# Phenotyping Reveals Targets of a Pseudo‐Natural‐Product Autophagy Inhibitor

**DOI:** 10.1002/anie.202000364

**Published:** 2020-04-21

**Authors:** Daniel J. Foley, Sarah Zinken, Dale Corkery, Luca Laraia, Axel Pahl, Yao‐Wen Wu, Herbert Waldmann

**Affiliations:** ^1^ Max Planck Institute of Molecular Physiology Dortmund Germany; ^2^ Current address: School of Physical and Chemical Sciences University of Canterbury Christchurch New Zealand; ^3^ Faculty of Chemistry and Chemical Biology Technical University Dortmund Dortmund Germany; ^4^ Department of Chemistry Umeå Centre for Microbial Research Umeå University Umeå Sweden; ^5^ Current address: Department of Chemistry Technical University of Denmark Copenhagen Denmark; ^6^ Compound Management and Screening Centre Dortmund Germany

**Keywords:** alkaloids, autophagy, chemical biology, inhibitors, natural products

## Abstract

Pseudo‐natural‐product (NP) design combines natural product fragments to provide unprecedented NP‐inspired compounds not accessible by biosynthesis, but endowed with biological relevance. Since the bioactivity of pseudo‐NPs may be unprecedented or unexpected, they are best evaluated in target agnostic cell‐based assays monitoring entire cellular programs or complex phenotypes. Here, the Cinchona alkaloid scaffold was merged with the indole ring system to synthesize indocinchona alkaloids by Pd‐catalyzed annulation. Exploration of indocinchona alkaloid bioactivities in phenotypic assays revealed a novel class of azaindole‐containing autophagy inhibitors, the azaquindoles. Subsequent characterization of the most potent compound, azaquindole‐1, in the morphological cell painting assay, guided target identification efforts. In contrast to the parent Cinchona alkaloids, azaquindoles selectively inhibit starvation‐ and rapamycin‐induced autophagy by targeting the lipid kinase VPS34.

## Graphical abstract

**The Cinchona alkaloid** scaffold was synthetically merged with the indole ring system, and the bioactivities of the products in phenotypic assays revealed a novel class of autophagy inhibitors, azaquindoles. Subsequent characterization of potent azaquindole‐1 in the morphological cell painting assay guided target identification efforts. In contrast to the parent Cinchona alkaloids, azaquindoles selectively inhibit starvation‐ and rapamycin‐induced autophagy by targeting the lipid kinase VPS34.

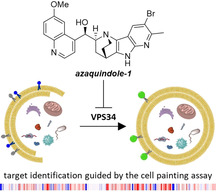

## Introduction

Natural products (NPs)^1^ occupy a definable area of chemical space,^2^ and are notable for their diverse frameworks and shapes, stereochemical complexity, heteroatom content, high fraction of sp^3^‐hybridized atoms, and diverse bioactivities. Significant focus has been placed on the design and preparation of biologically relevant small molecules endowed with NP‐like features.^3^, ^4^ Recently developed design strategies include complexity‐to‐diversity (CtD) approaches, in which alternative NPs^4^ or complex synthetic intermediates^5^ are modified or distorted; and biology‐oriented synthesis (BIOS),^3^ in which the NP structure is simplified to arrive at synthetically tractable NP‐derived scaffolds. We very recently introduced pseudo‐natural products as novel compounds with NP‐like structures and properties.^6^, ^7^, ^8^, ^9^, ^10^, ^11^ Pseudo‐NPs are obtained through the unprecedented combination of NP‐derived fragments and occupy areas of biologically relevant chemical space inaccessible to nature through biosynthesis.^12^ There is an urgent requirement for unbiased biological profiling of these new compounds since their possible biological activities are unknown.

**Figure 1:**
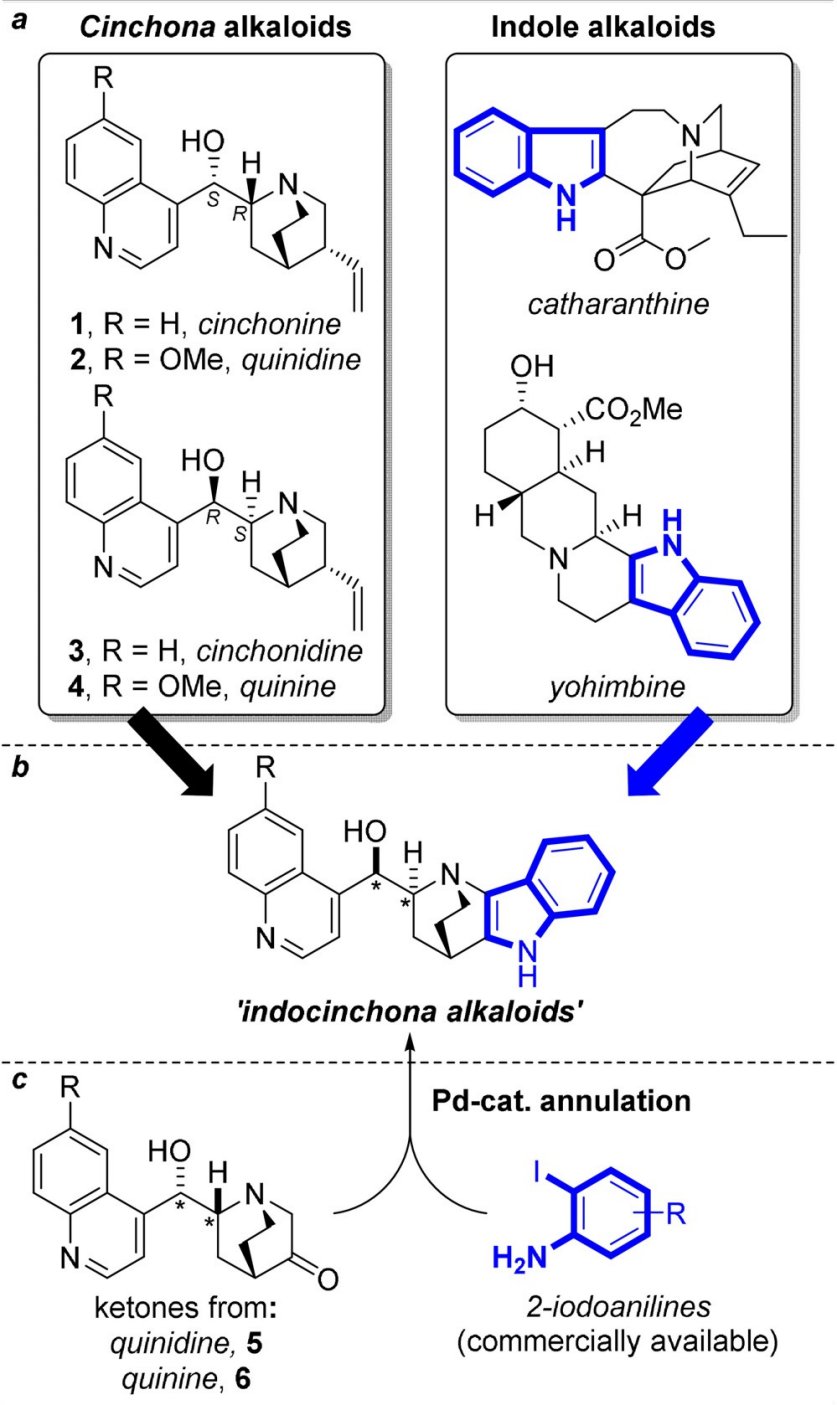
Proposed design strategy to derive novel indole alkaloids from the Cinchona alkaloids. a) The Cinchona alkaloids and representative 2,3‐fused indole alkaloids. b) The targeted unnatural indole alkaloids derived from Cinchona alkaloids. c) Proposed synthetic route to the pseudo‐NPs by harnessing a Pd‐catalyzed annulation between the quinidine/quinine‐derived ketones **5** and **6** and 2‐iodoanilines. *Stereochemistry relative to compounds **1** and **2** as appropriate.

For the design of a new pseudo‐NP class we were drawn to a biologically unprecedented fusion of the biosynthetically related^13^, ^14^ Cinchona alkaloids **1**–**4** and indole alkaloids promised to yield a new pseudo‐NP class with unexpected bioactivity. For efficient synthesis of an indocinchona alkaloid library (Figure 1 a,b) we envisioned combining the indole ring system, found in a variety of alkaloids (e.g. catharanthine, yohimbine),^13^ with the caged quinuclidine ring system of the Cinchona alkaloids in an edge‐on‐edge (2,3)‐fusion, which is biologically unprecedented. Here, we describe the expedient use of a Pd‐catalyzed annulation to prepare the envisioned indocinchona alkaloid library (Figure 1 b,c).^15^ Biological investigation of the collection, including phenotypic characterization of bioactivities in an unbiased, multiparametric cell‐painting assay revealed a new inhibitor of both starvation‐ and rapamycin‐induced autophagy, which targets the lipid kinase VPS34.

## Results and Discussion

For 2,3‐fusion of the indole ring system to Cinchona alkaloids, a unifying, operationally straightforward, and preferably one‐pot connective reaction was required. It was envisioned that the known^16^ Cinchona alkaloid derived ketones **5** and **6** would provide suitable coupling partners to undergo regioselective Pd‐catalyzed annulations with 2‐iodoanilines^15^ to furnish the target pseudo‐NPs in a single step (Figure 1 c).

**Figure 2:**
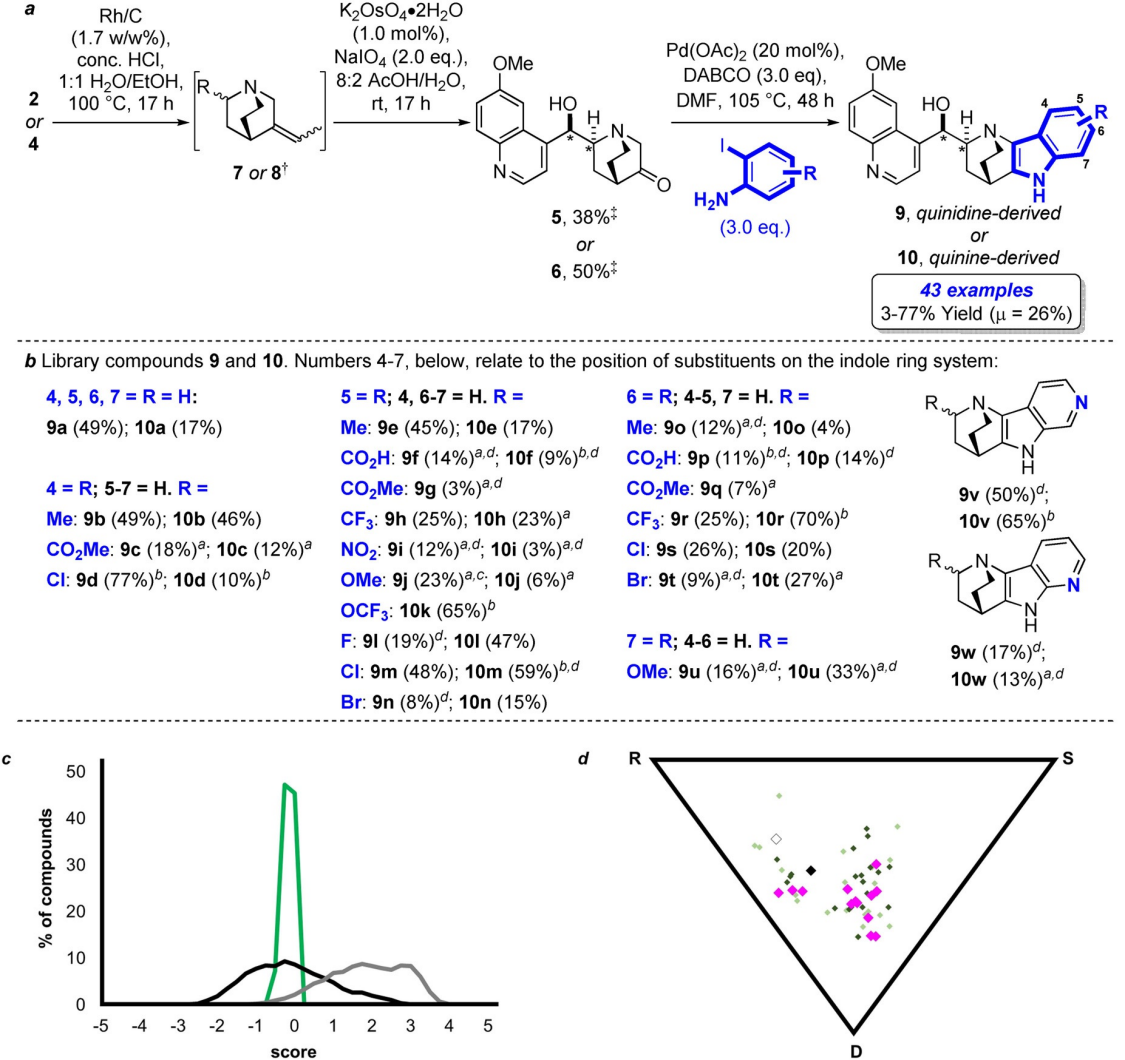
Preparation and chemoinformatic analysis of the novel indole alkaloids **9** and **10**. a) Synthetic route to prepare compounds **9** and **10**. b) Library members and yields from the Pd‐catalyzed annulation. c) NP‐likeness scores comparing the indocinchona library (green) with natural products (from ChEMBL, grey) and marketed drugs (DrugBank, black). The data are binned into histograms (0.25 units in width). N.b. the score for quinine/quinidine is +0.83. Scores were calculated using the method developed by Ertl,^18^ implemented in RDKit.^25^ d) Shape analysis (PMI) plot of the indocinchona alkaloids versus three idealised molecular shapes (R=rod; D=disk; S=sphere): — quinidine **2** (white); quinine **4** (black); indoquinidines **9 a**–**w** (light green); indoquinines **10 a**–**v** (dark green); 7‐azaindoquinines **10 w** to **10 w‐l** (pink). Generated by LLAMA.^21^ *Stereochemistry relative to compounds **1**–**2** as appropriate. †In each case a 2:1 mixture of *E*/*Z* alkenes was observed. ≠Yields given over two steps. [a] 30 mol % Pd(OAc)_2_, 72 h. [b] 10 mol % Pd(OAc)_2_, 24 h. [c] The iodoaniline HCl salt and 6.0 equiv. DABCO were used. [d] Isolated as the TFA salt. *μ*=mean. All yields are unoptimized.

For the syntheses of **5** and **6** the quinine/quinidine terminal alkenes were isomerized^17^ to the internal alkenes **7** and **8**, respectively (Figure 2 a,b). The alkenes were oxidatively cleaved under modified Lemieux–Johnson conditions^16^ to give **5** and **6** in a telescoped two‐step procedure in 38 and 50 % yields, respectively . For the envisioned Pd‐catalyzed Heck‐type annulation we employed the ligand‐free variant developed by Chen et al.^15^ and, pleasingly, exposure of **5** and **6** to 2‐iodoanilines in the presence of catalytic Pd(OAc)_2_ and DABCO provided the targeted pseudo‐NP compounds in viable yields and in multimilligram amounts. 2‐Iodoanilines bearing a range of polar functionalities, including nitro and carboxylic acid functional groups at the 3–6 positions were tolerated and the reaction protocol was successfully extended to the preparation of 6‐ and 7‐azaindoles (**9**–**10 v** and **9**–**10 w**). Overall, the indole synthesis proved to be very robust and yielded a collection of 61 indocinchona alkaloids in total (Figure 2 a,b; see Section 3 in the Supporting Information).

A substructure search in the dictionary of natural products (DNP) revealed that both the indocinchona alkaloid scaffold and the fused quinuclidine‐indole ring system were not found in NPs (see Figure S1 in the Supporting Information for substructure searches). Comparison of the NP‐likeness^18^ of the collection with the guiding Cinchona alkaloid NPs **1**–**4**, NPs from ChEMBL,^19^ and drugs from DrugBank (Figure 2 c)^20^ revealed that the indocinchona alkaloids display a narrow NP‐score distribution (μ[NP‐likeness score]=−0.04), and contain connectivity that is more synthetic‐compound‐like than both the guiding Cinchona alkaloids (+0.83), and NPs in general (*μ*=+1.95). The average scores for the indocinchona alkaloids are close to the scores for compounds in DrugBank (*μ*=−0.01, and +0.02, respectively).

Shape analysis of the compound collection by generating the two normalized principal moments of inertia values (Figure 2 d)^21^ indicated that the collection has a wide distribution of molecular shapes and a high three‐dimensional character. The indocinchona alkaloids also exhibit a high fraction of sp^3^‐hybridized carbon centers (μ[Fsp^3^]=0.30), a valuable feature for the successful progression of drug candidates.^22^ Overall the library has favorable properties for molecular discovery,^23^ with 96 % of the compounds falling within Lipinksi “Rule‐of‐5” space (see Figure S2).^24^

Since phenotypic screening enables identification of bioactivities of new small‐molecule classes in an unbiased manner,^26^, ^27^, ^28^ we subjected the compound collection to a range of cell‐based screens, including a phenotypic assay that monitors autophagic flux (see Table 1).^29^ Autophagy degrades and recycles both superfluous and damaged proteins and organelles by autophagosomes. Autophagy plays a crucial role in degenerative diseases and cancer, and novel small‐ molecule autophagy inhibitors may provide inspiration for new drug discovery programs.^30^, ^31^

To identify autophagy inhibitors we monitored puncta formation in MCF7 cells stably transfected with an EGFP‐tagged LC3 protein (MCF7‐EGFP‐LC3 cells) upon autophagy induction by amino‐acid starvation, or treatment with the mTOR inhibitor rapamycin, using high‐throughput automated image acquisition and analysis.^32^ Use of the autophagosome‐autolysosome fusion inhibitor chloroquine (CQ) enhanced the dynamic range of the assay.

**Figure 3:**
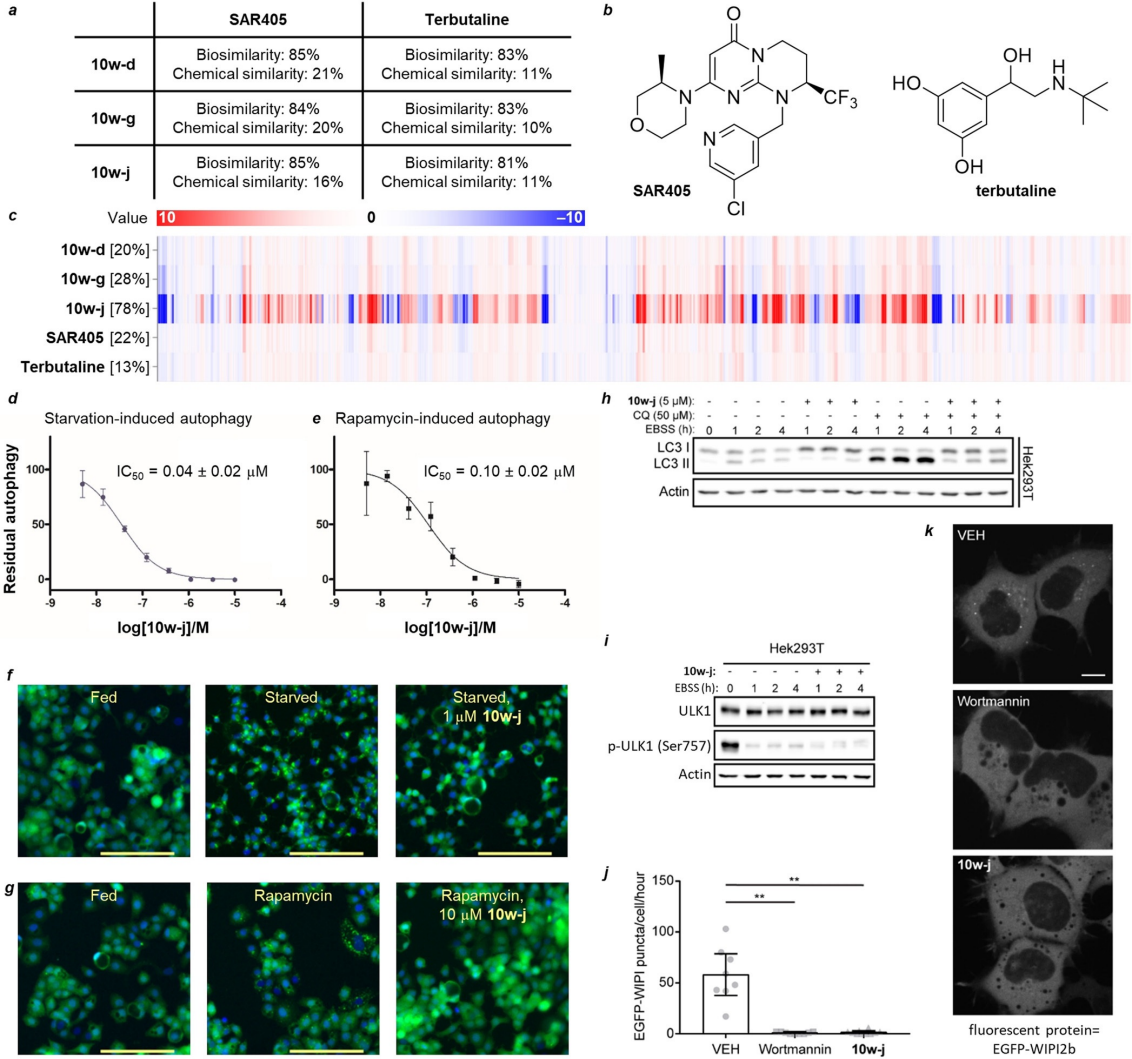
Biological evaluation of the lead compound **10 w‐j**. a) Bio and chemical similarities between selected **10 w** compounds and their common reference compounds^9^). Biosimilarity refers to the bioactivity similarity of the cell painting assay “fingerprint” profiles. Chemical similarity refers to the structural similarity of compounds (Tanimoto coefficient).^9^ b) Chemical structures of the reference compounds. c) Cell painting assay fingerprint profiles for selected **10 w** compounds and their biosimilar reference compounds. The percentages within the brackets refer to the induction, the fraction of parameters (in %) that underwent significant changes (median absolute deviation [MAD] value) upon compound treatment of at least ± threefold of the median determined for the DMSO controls (see Section 5.3).^9^ d) Dose‐dependent inhibition of EGFP‐LC3 accumulation in MCF7 cells induced by amino acid starvation by Azaquindole‐1 (**10 w‐j**). e) Dose‐dependent inhibition of rapamycin‐induced EGFP‐LC3 accumulation in MCF7 cells by Azaquindole‐1 (**10 w‐j**). f) Fluorescence microscopy images of the starvation‐induced autophagy screen. g) Fluorescence microscopy images of the rapamycin‐induced autophagy screen. Blue=Hoechst; green=EGFP‐LC3. Scale bars: 150 μm. Data are given as the mean ± SD, *n*≥3, representative graphs and images shown. h) Western blot analysis of LC3 lipidation in Hek293T cells undergoing starvation in the presence or absence of **10 w‐j** or chloroquine (CQ), as indicated. i) Western blot analysis of ULK1 phosphorylation status in starved Hek293T cells when treated with **10 w‐j**. j) Quantification of the total number of EGFP‐WIPI2b foci appearing during 1 h of EBSS treatment with or without **10 w‐j** in Hek293A cells stably expressing EGFP‐WIPI2b. Bars show mean ± SD from four biologically independent experiments. Data points represent individual cells pooled from independent experiments. Significance was determined from biological replicates using a two‐tailed, unpaired t‐test. **P≤0.01. k) Representative fluorescence images of Hek293A EGFP‐WIPI cells after 1 h of treatment as shown in (j). Scale bars, 10 μm. See Figures S6 and S7 for versions of western blots for (f) and (g) using alternative cell lines.

The compounds **10**, derived from quinine, inhibited starvation‐induced autophagy (Table 1, entries 1–9), but no inhibitory activity was observed for the quinidine‐derived indoles **9 a**–**w** at 10 μm. The compounds substituted at the 5‐ and 6‐positions of the indole ring (entries 1–7) were relatively weak inhibitors of starvation‐induced autophagy (IC_50_≈4.7–8.1 μm), and those substituted with polar functionalities at the 7‐position of the indole ring (entries 8 and 9) gave appreciably higher activities. Thus, the 7‐azaindole‐substituted compound **10 w** inhibited starvation‐induced autophagy with IC_50_=4.33±1.7 μm, whilst the 7‐methoxy‐substituted indole **10 u** gave the most active initial compound with IC_50_=2.46±0.6 μm. Notably, the latter two compounds (entries 8 and 9) also inhibited rapamycin‐induced autophagy, suggesting that they act either downstream or independently of mTOR. In general, 7‐azaindoles were potent, sub‐micromolar inhibitors of autophagy (entries 10–21. See Table S1 for synthetic yields). Introduction of chloride at the 4‐position of the 7‐azaindole (R=Cl, entry 10) gave an inhibitor of starvation‐ and rapamycin‐induced autophagy with sub‐micromolar activity. Introduction of substituents at the 5‐position of the 7‐azaindole ring system (entries 11–18) revealed that either polar (e.g. NO_2_; entry 14) or lipophilic (e.g. Me, CF_3_, Cl, Br, I; entries 11, 13, 16–18) substituents led to nanomolar activities in starvation‐induced autophagy, and low‐ or sub‐micromolar activities in rapamycin‐induced autophagy. Markedly, while electronic factors cannot be ruled out, the size of the substituent seems to significantly impact the activity. The small fluoride‐substituted 7‐azaindole **10 w‐f** (entry 15) is inactive, whilst nanomolar inhibition of starvation‐induced autophagy is observed for larger halogen substituents (entries 16–18). Furthermore, the aryl‐substituted analogue **10 w‐c** (entry 12) leads to diminished inhibitory activity versus the unfunctionalized 7‐azaindole (entry 9). Substitution at the 6‐position of the 7‐azaindole ring system does not appear to significantly improve inhibitory activity (entries 20 and 21). Surprisingly, however, substitution at both the 5‐ and 6‐positions (with Br and Me, respectively, entry 19) of the 7‐azaindole ring system gave **10 w‐j**, which had the highest observed inhibitory activity against both starvation‐ and rapamycin‐induced autophagy (0.04±0.02 μm and 0.10±0.02 μm, respectively, Figures 3 d–g). We termed the active, quinine‐derived 7‐azaindole compounds azaquindoles. The compound **10 w‐j** (azaquindole‐1) was chosen as a potent, representative member for further investigations.

**Table 1:** Identification of Cinchona‐alkaloid‐inspired inhibitors of starvation and/or rapamycin‐induced autophagy, derived from quinine.

Entry	Compound	R group and position	Starvation‐ Induced IC_50_ [μm]	Rapamycin‐ Induced IC_50_ [μm]
1	**10 h**	5‐CF_3_	7.86±0.8	n/a
2	**10 i**	5‐NO_2_	7.33±1.5	n/a
3	**10 k**	5‐OCF_3_	4.68±1.4	n/a
4	**10 m**	5‐Cl	5.54±2.5	n/a
5	**10 n**	5‐Br	6.78±1.1	n/a
6	**10 r**	6‐CF_3_	5.82±3.0	n/a
7	**10 s**	6‐Cl	8.12±1.5	n/a
8	**10 u**	7‐OMe	2.46±0.6	2.37±0.7
9	**10 w**	7‐azaindole	4.33±1.7	4.95±0.7
10	**10 w‐a**	4‐Cl‐7‐azaindole	0.52±0.20	0.65±0.35
11	**10 w‐b**	5‐Me‐7‐azaindole	0.31±0.09	0.86±0.26
12	**10 w‐c**	5‐Ar‐7‐azaindole	9.00±1.1	n/a
13	**10 w‐d**	5‐CF_3_‐7‐azaindole	0.12±0.03	0.77±0.29
14	**10 w‐e**	5‐NO_2_‐7‐azaindole	0.67±0.13	1.26±0.20
15	**10 w‐f**	5‐F‐7‐azaindole	n/a	n/a
16	**10 w‐g**	5‐Cl‐7‐azaindole	0.11±0.04	0.85±0.14
17	**10 w‐h**	5‐Br‐7‐azaindole	0.08±0.03	0.81±0.35
18	**10 w‐i**	5‐I‐7‐azaindole	0.08±0.02	1.24±0.20
19	**10 w‐j** (azaquindole‐1)	5‐Br‐6‐Me‐7‐azaindole	0.04±0.02	0.10±0.02
20	**10 w‐k**	6‐Me‐7‐azaindole	3.12±0.5	5.11±1.4
21	**10 w‐l**	6‐Cl‐7‐azaindole	3.06±0.9	6.21±2.2
22	**9 w**	7‐azaindole	n/a	nd
23	**9 w‐b**	5‐Me‐7‐azaindole	n/a	nd

All data are shown as mean ± SD of three independent experiments (*N*=3; *n*≥3). All compounds were initially assayed at a concentration of 10 μm. For hits reducing the number of LC3 puncta by more than 50 %, IC_50_ values were determined. n/a=inactive (no reduction of LC3 puncta at 10 μm). Ar=4‐Cl‐C_6_H_4_. nd=not determined.

Investigation of **10 w‐j** analogues, in which various molecular features were either masked or truncated (see Figures S3 and S4), in the autophagy assay determined that the key feature of the compound for inhibition of rapamycin‐ and starvation‐induced autophagy is the fused quinuclidine/azaindole ring system, while substituents at the azaindole 5‐position and the presence of the quinoline ring are key drivers of potency. Importantly, the corresponding quinidine‐derived 7‐azaindoles (**9 w**, **9 w‐b**, **9 w‐k**) were inactive in the autophagy assays, as were the parent Cinchona alkaloids (see Table S1 and Figure S5). The inhibition of autophagy was not an activity shared by simple indoles, 7‐azaindoles, quinuclidines, or quinolines (see Figure S5). Indole‐containing natural products (82 compounds) within our in‐house library were also found to be inactive against autophagy (see Table S2). Thus, the azaquindole **10 w** bioactivity is not shared by the individual NP fragments.

To gain insight into mode of action and point towards potential targets, the pseudo‐NP series **10 w** was subjected to a nonbiased phenotyping assay.^9^, ^33^, ^34^, ^35^, ^36^, ^37^ Using image‐based analysis, the “cell painting” morphological profiling assay measures and quantifies a large number of phenotypic changes to cells upon treatment with a compound of interest (COI), and then the changes are condensed in a fingerprint profile (see Section 5.3 for details). High similarity to profiles generated for reference compounds that have annotated biological activities (and/or modes of action) may indicate particular molecular targets. Investigation of the sub‐micromlar active 5‐substituted members of the **10 w** series in the cell painting assay revealed two common reference compounds: the VPS34 inhibitor, SAR405, and the β_2_ adrenergic receptor agonist, terbutaline (Figure 3 a–c). Investigation of the in vitro inhibitory activity of **10 w‐j** against the β_1_ and β_2_ adrenergic receptors revealed no agonistic activity against both receptors, and low antagonistic activity (8.6 % and 22 % inhibition for β_1_ and β_2_, respectively). The weak activity suggested that the β_1_ and β_2_ adrenergic receptors were unlikely to be relevant to the autophagy inhibition activity observed. In addition, **10 w‐d** showed no inhibitory activity against either receptor. Excitingly, however, VPS34 was inhibited by **10 w‐j** at 350 nm in vitro. Significantly, PI3K kinase PIK3C3/VPS34 plays an important role in autophagosome biogenesis downstream of mTOR.^38^

To determine the selectivity of **10 w‐j**, it was screened against the wider kinome (485 kinases), which revealed inhibition of seven additional kinases with IC_50_ values of less than 10 μm (see Table S3). Most notably, **10 w‐j** demonstrated inhibition of a number of PI3Ks (see the Supporting Information, Table S3, entries 2, 3, 5, 7, 8), and of CLK2 and CLK4 kinases. In light of the medium nanomolar cellular potency of azaquidole‐1 (see Table 1, entry 19) kinase inhibition with sub‐micromolar activity was deemed most relevant. Inhibition of CLK2 and CLK4 is likely not relevant since the CLK2/4 kinase inhibitor ML167, and the CLK2/3/4 inhibitor TG003, were inactive in the autophagy assay at 10 μm (see Table S4). We have previously shown that inhibition of PIK3C2G and PI4KB using several highly potent and selective inhibitors does not result in autophagy inhibition.^39^ Additionally, respective investigation of selective SPHK2 and PIK3CD inhibitors in the autophagy assay devalidated likely inhibitory roles for these kinases (see Table S4). Across the azaquindole class, inhibition of VPS34 activity correlated strongly with autophagy inhibitory activity (see Table S5) further strengthening the hypothesis that VPS34 is the primary target in relation to autophagy inhibition.

To further validate VPS34 as cellular target we treated four different cell lines with azaquindole‐1 and it resulted in very strong inhibition of LC3 lipidation, confirming its autophagy‐inhibiting activity (Figure 3 h; see Figure S7). In addition, ULK1 phosphorylation status in serum starved cells was investigated to confirm that signaling upstream of ULK‐1 is not inhibited by azaquindole‐1. ULK1 is phosphorylated by mTOR at Ser757 to prevent its interaction with, and activation by, AMPK.^40^ Under serum starvation, mTOR is inhibited and reduces ULK1 Ser757 phosphorylation. Dephosphorylated ULK1 interacts with AMPK and promotes VPS34 activation by Beclin‐1 phosphorylation.^41^ Western blot analysis suggested that ULK1 is dephosphorylated in the presence of azaquindole‐1, confirming mTOR inhibition (Figure 3 i; see Figure S7). VPS34 activity in cellulo was monitored by WIPI2b puncta formation. WIPI2b is recruited to the autophagosome initiation site in a PI3P‐dependent fashion.^42^ Hek293A cells stably expressing EGFP‐WIPI2b^43^ were treated with EBSS to induce autophagy in either the presence or absence of azaquindole‐1 and imaged every 15 seconds for 1 hour. Quantification of the number of WIPI2b foci that appeared over the 1 hour time period showed that azaquindole‐1 strongly inhibited WIPI2b puncta formation, similar to wortmannin, and consistent with VPS34 inhibition (Figure 3 j–k; see the Supporting Movies).

**Figure 4:**
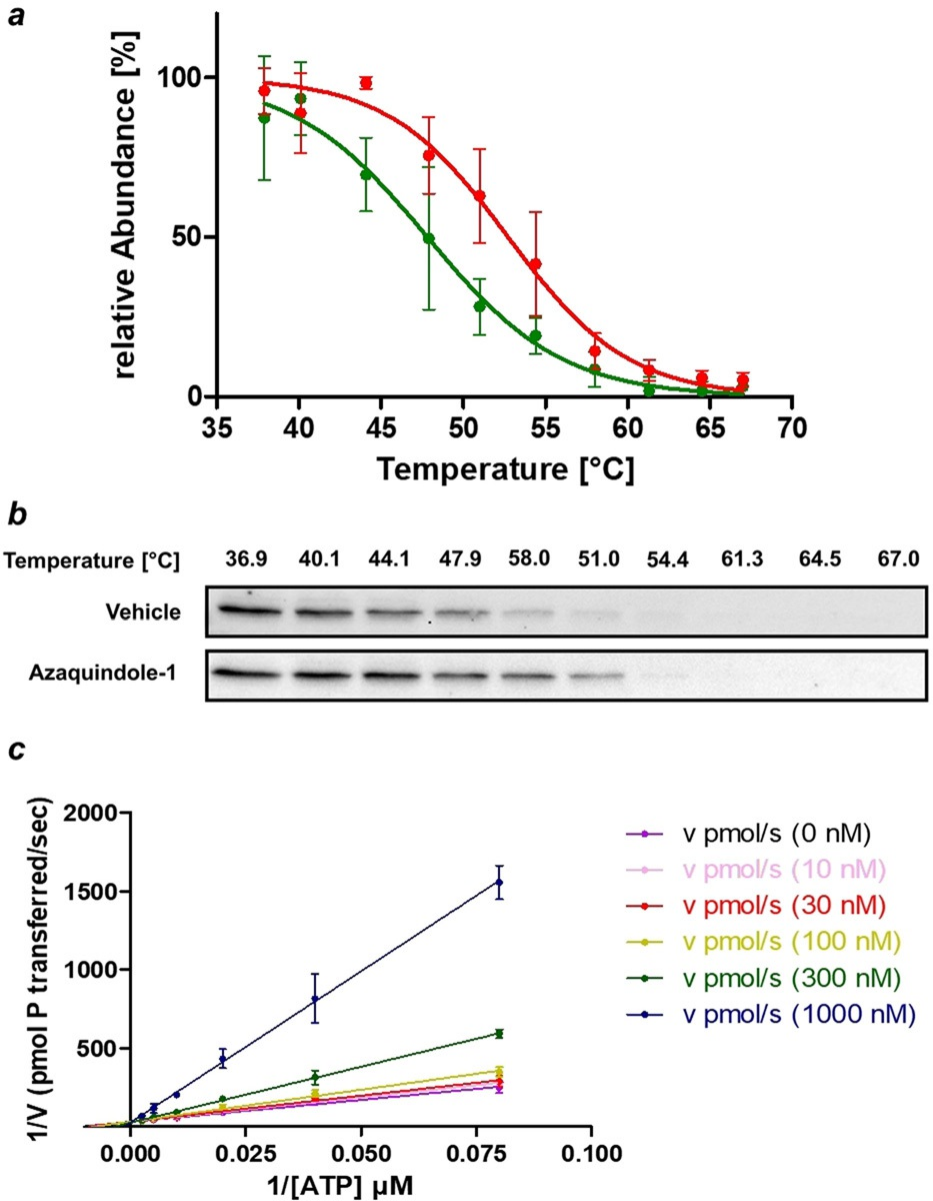
a) Cellular thermal shift assay (CETSA) for the binding of azaquindole‐1 (**10 w‐j**) to VPS34 in cell lysate (green line=VEH; red line=**10 w‐j**). Data is mean ± SD, *n*=3; b) Representative CETSA blot. See Section 5.8 in the Supporting Information for further details. c) Lineweaver–Burk plot for inhibition of VPS34 by **10 w‐j**. Data is mean ± SD, *n*=3.

In‐cell target engagement of VPS34 was demonstrated by means of a cellular thermal shift assay (CETSA).^44^ An average stabilization of 5.03±1.8 °C was observed in cell lysate (Figure 4 a,b). To determine the mode of VPS34 inhibition we investigated the kinetics for inhibition of VPS34 by azaquindole‐1 (**10 w‐j**), which indicated that it was an ATP competitive inhibitor (Figure 4 c).

We compared the cell painting profile of azaquindole‐1 against other recently identified autophagy inhibitors^11^, ^29^, ^38^, ^39^, ^45^, ^46^ (see Figures S10 and S11). Our analysis revealed that azaquindole‐1 (**10 w‐j**) was biosimilar to oxautin‐1, autoquin, and autophinib. Notably, autophinib is a known VPS34 inhibitor, and the cell painting analysis further validates this earlier finding. The ability of the cell painting assay data to suggest and subsequently identify molecular targets is therefore apparent. However, oxautin‐1 and autoquin do not inhibit VPS34. This outcome suggests that the profile may represent compound bioactivity in a broader sense. Thus, the cell painting assay may be a good experimental means to identify clusters of compounds that exhibit similar phenotypic outputs, but not necessarily through the same modes of action. These insights will be investigated more thoroughly in a forthcoming study.

## Conclusion

In conclusion, we have reported a novel approach to prepare a new class of pseudo‐natural‐product autophagy inhibitors by fusing the indole ring system with the cinchona alkaloid scaffold in a biologically unprecedented manner. The lead compound, azaquindole‐1 (**10 w‐j**), appears to suppress autophagy by inhibiting the lipid kinase VPS34, as identified by phenotypic profiling using the cell painting assay. These results highlight the potential of the cell painting assay as a target identification technique. Our synthetic strategy may now be extended to investigate alternative modes of indole fusion, and to harness alternative alkaloids (and other natural products) to make numerous new classes of pseudo‐natural products that are not available to nature through biosynthesis, and which may be endowed with unique biological activities.

## Conflict of interest

The authors declare no conflict of interest.

## Supporting information

As a service to our authors and readers, this journal provides supporting information supplied by the authors. Such materials are peer reviewed and may be re‐organized for online delivery, but are not copy‐edited or typeset. Technical support issues arising from supporting information (other than missing files) should be addressed to the authors.

Supplementary

Supplementary

Supplementary
