# Supplementary material for: Phenotyping Reveals Targets of a Pseudo‐Natural‐Product Autophagy Inhibitor
Source: Angew Chem Int Ed Engl. 2020 Apr 21;59(30):12470–6. doi: 10.1002/anie.202000364 (PMC7383971; doi:10.1002/anie.202000364)
Supplement: Supplementary file 1 — Supplementary [file ANIE-59-12470-s001.pdf]

Angewandte Chemie
Eine Zeitschrift der Gesellschaft Deutscher Chemiker

Supporting Information

Phenotyping Reveals Targets of a Pseudo-Natural-Product Autophagy Inhibitor

*Daniel J. Foley, Sarah Zinken, Dale Corkery, Luca Laraia, Axel Pahl, Yao-Wen Wu, and Herbert Waldmann**

anie_202000364_sm_miscellaneous_information.pdf
anie_202000364_sm_movie_10w-j.avi
anie_202000364_sm_movie_VEH.avi

Supporting Information

Contents:

1.0 Supporting Figures 3
1.1 Substructure search in the Dictionary of Natural Products 3
1.2 Molecular properties of the indocinchona alkaloids 3
1.3 SAR analysis and determination of the minimum required pharmacophore for azaquindole activity 4
1.4 Additional compounds tested for activity in the autophagy assay 5
1.5 LC3 lipidation in starved cells treated with azaquindole-1 6
1.6 ULK1 phosphorylation in starved cells treated with azaquindole-1 6
2.0 Supporting Tables 7
2.1 Synthetic yields and autophagy inhibition IC_{50} values for Cinchona alkaloid-derived 7-azaindoles 7
2.2 Activity data for indole-containing natural products screened in the autophagy assay 9
2.3 Kinases showing *in vitro* inhibition below 10 μM by azaquindole-1 14
2.4 Investigation of selective kinase inhibitors in the autophagy assay 15
2.5 *In vitro* IC_{50} measurements for azaquindoles and related structures against VPS34 16
3.0 Pd-catalysed indole formations: substrate scope summary 17
3.1 Substrate scope experiments: successful reactions 17
3.2 Substrate scope experiments: failed reactions 19
3.3 SAR investigations: successful reactions 21
3.4 SAR investigations: failed reactions 23
4.0 Computational analysis 25
4.1 Compounds in the analysis 25
4.2 NP-likeness scoring 28
4.3 Molecular properties analysis 28
4.4 Novelty assessment 29
5.0 Biological experimental 32
5.1 Cell culture for the autophagy assay 32
5.2 High-content screening for autophagy inhibitors 32
5.3 Cell painting assay 33
5.3.1 Discussion of the Induction Parameter 36
5.3.2 Availability of the cell painting assay code 37
5.3.3 Further discussion and interpretation of the cell painting data 37
5.3.4 Comparison of azaquindole-1 with other recently identified autophagy inhibitors 38
5.4 β-Adrenergic receptor screening 40

5.5 Kinase panel data 41
5.6 Immunoblotting 51
5.7 Live-cell microscopy 51
5.8 Cellular thermal shift assay (CETSA) in cell lysate 52
5.9 VPS34 kinetic experiments 53
5.9.1 Experimental protocol 53
5.9.2 Michaelis–Menten Saturation Curves 54
5.9.3 %inhibition versus [*10w-j*] plot 54
5.9.4 Lineweaver–Burk Plot 55
6.0 Synthetic experimental 56
6.1 General experimental 56
6.2 General procedures 57
6.3 Compound data 59
6.3.1 Preparation of the Cinchona alkaloid derived ketones 59
6.3.2 Preparation of the Cinchona alkaloid derived indoles 64
6.3.2.1 Preparation of the quinidine-derived indoles 65
6.3.2.2 Preparation of the quinidine-derived azaindoles 85
6.3.2.3 Preparation of the quinine-derived indoles 90
6.3.2.4 Preparation of the quinine-derived azaindoles 112
6.3.2.5 Preparation of compounds to investigate the minimum necessary pharmacophore 127
6.3.2.5.1 Preparation of compound S-1 127
6.3.2.5.2 Preparation of compound S-2 128
6.3.2.5.3 Preparation of compounds S-3 and S-4 132
6.3.2.5.4 Preparation of compound S-5 139
6.3.2.5.5 Preparation of compound S-6 146
7.0 Processed NMR Spectra 147
8.0 References 320

1.0 Supporting Figures

1.1 Substructure search in the Dictionary of Natural Products

The following substructures were investigated in the Dictionary of Natural Products (http://dnp.chemnetbase.com, accessed 28/05/2018, Figure S1):

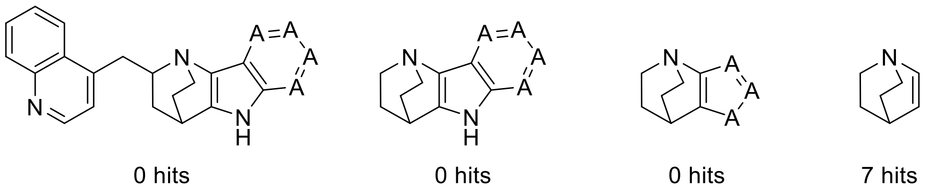

Figure S1 Substructure searches within the Dictionary of Natural Products. A = any atom.

1.2 Molecular properties of the indocinchona alkaloids

The indocinchona alkaloids have the following AlogP vs MW distribution (Figure S2).

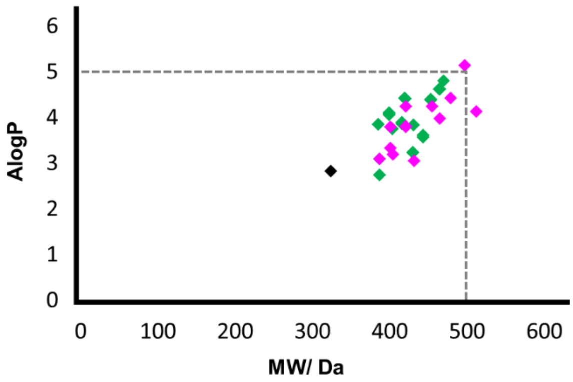

Figure S2 Molecular properties of the indocinchona alkaloids.
Indocinchona alkaloids – compounds **9a-w** and **10a-v** (green); azaquindoles – compounds **10w** to **10w-I** (pink); quinidine/quinine (black).

See Section 4.3 for further molecular property analyses.

1.3 SAR analysis and determination of the minimum required pharmacophore for azaquindole activity

The following compounds (Figure S3) were prepared to explore the structure-activity relationship (SAR) around azaquindole-1 (compound **10w-j**):

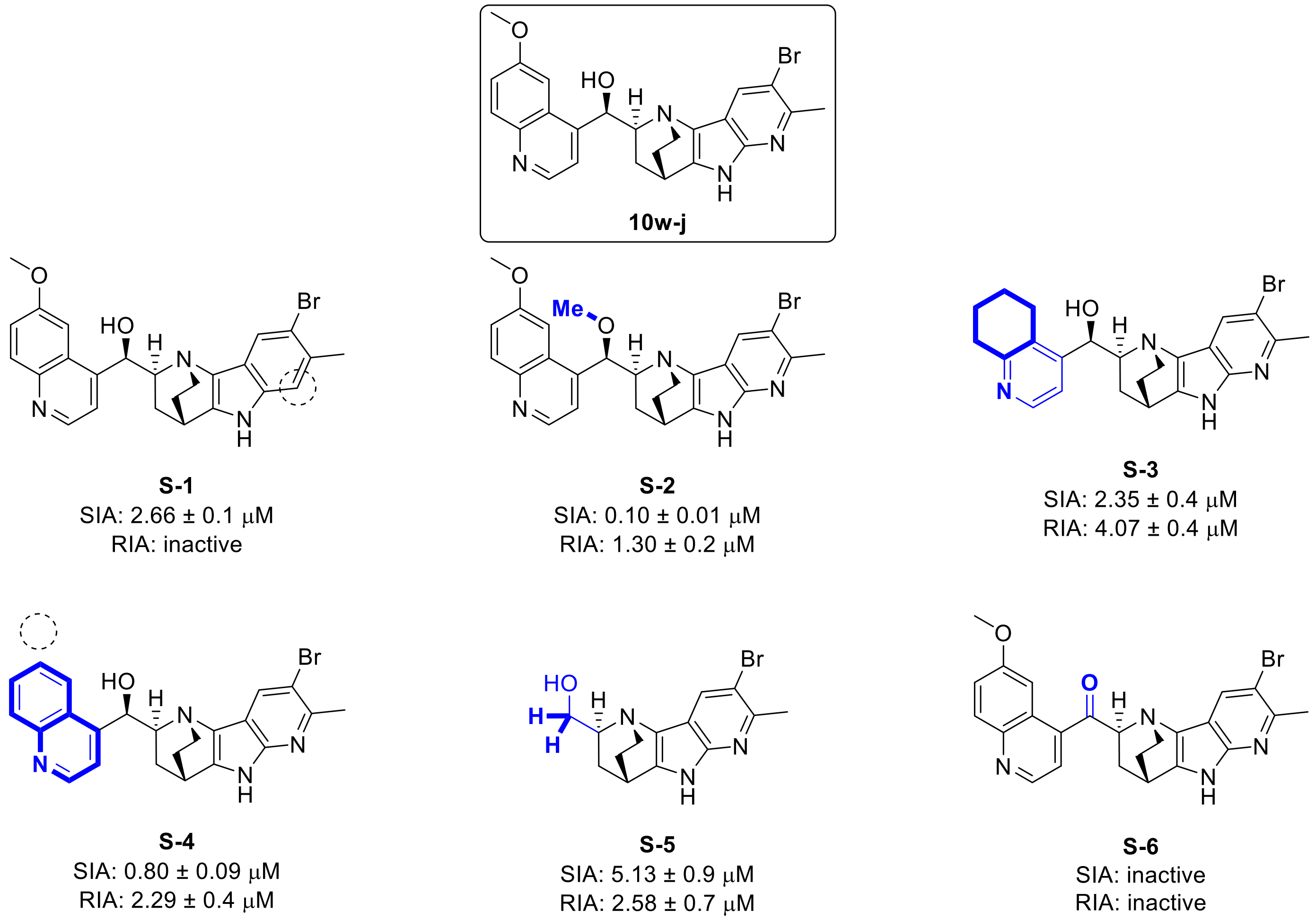

Figure S3 A Summary of the SAR explored for azaquindole-1.
SIA = starvation-induced autophagy. RIA= rapamycin-induced autophagy.

On the basis of the data above (Figure S3), we surmised that the following structural features were important for autophagy inhibition by indocinchona alkaloids (Figure S4):

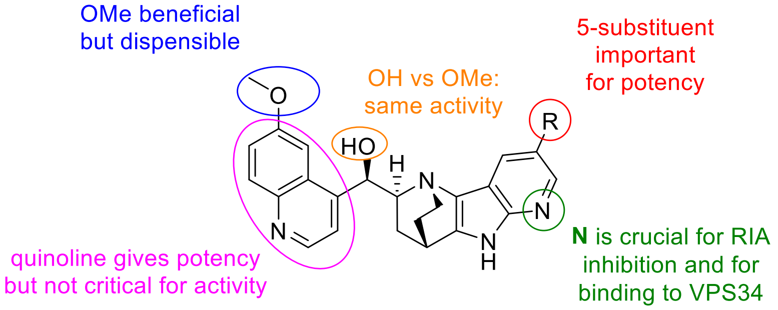

quinine-derived = active; *quinidine-derived = inactive*

Figure S4 A Summary of the key features required for autophagy inhibition. RIA= rapamycin-induced autophagy.

1.4 Additional compounds tested for activity in the autophagy assay

The following compounds, which were closely related to the lead compound **10w-j**, were investigated for activity in the autophagy assay and were found to be inactive at ≤10 µM (Figure S5):

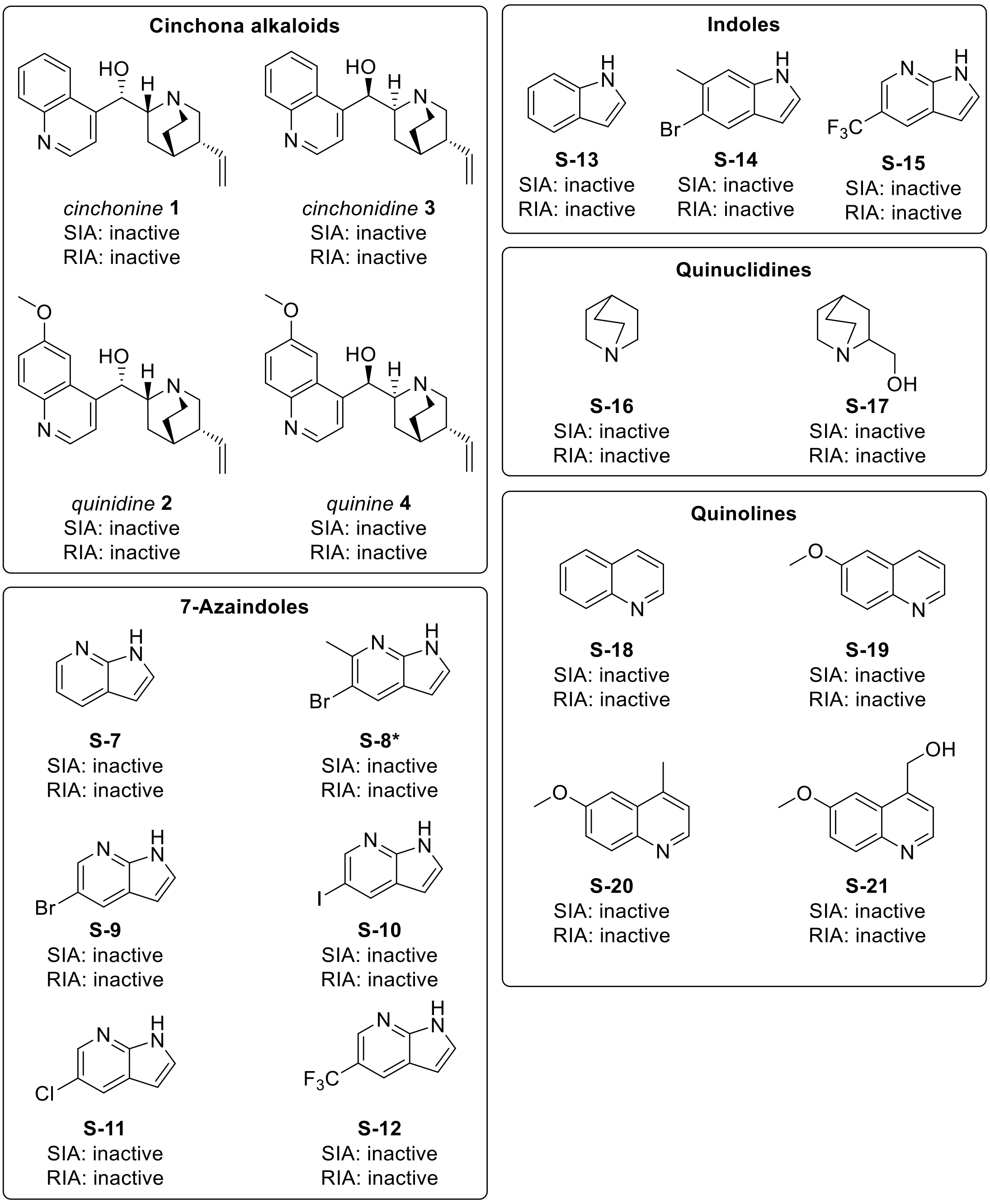

Figure S5 Several compounds that closely relate to the azaquindoles **10w** were found to be inactive in the autophagy assay. SIA = starvation-induced autophagy. RIA= rapamycin-induced autophagy.

*Compound **S-8** was found to inhibit hedgehog-induced osteogenesis in a cell-based assay,[1] with IC_{50} = 1.83 ± 0.44 µM (the assay serves as a proxy for identifying inhibitors of the hedgehog signalling pathway – for more details see Ref. [1], Fig. 1).

Overall, new bioactivity (autophagy inhibition) was realised through the combination of azaindoles with the quinine framework (n.b. azaquindoles **10w** were found to be inactive in the hedgehog-induced osteogenesis assay). None of the fragments above were found to be active in the cell painting assay.

1.5 LC3 lipidation in starved cells treated with azaquindole-1

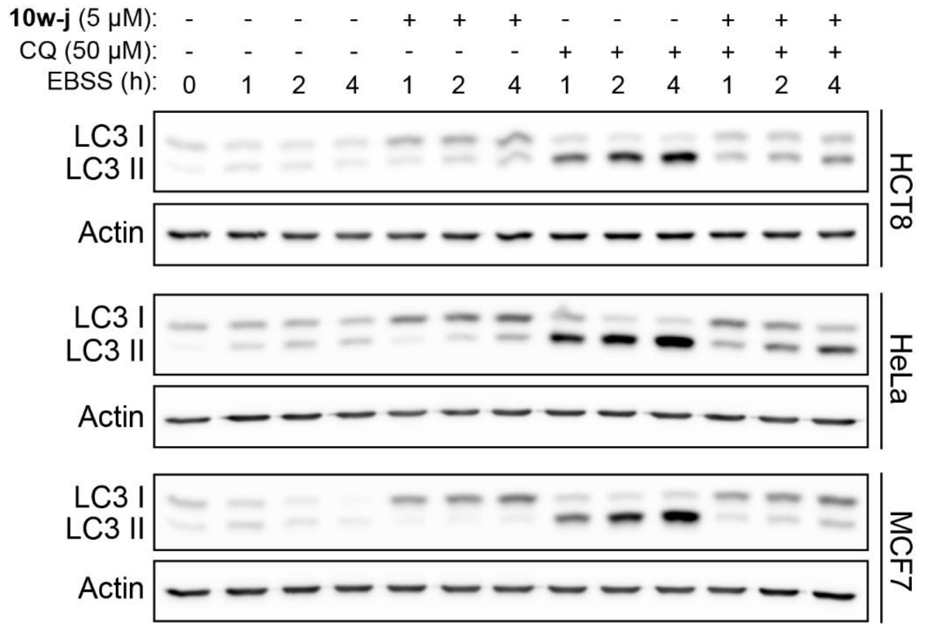

Figure S6 LC3 lipidation in cells (HCT8; HeLa; MCF7) undergoing starvation in the presence or absence of **10w-j** or chloroquine (CQ) as indicated.

1.6 ULK1 phosphorylation in starved cells treated with azaquindole-1

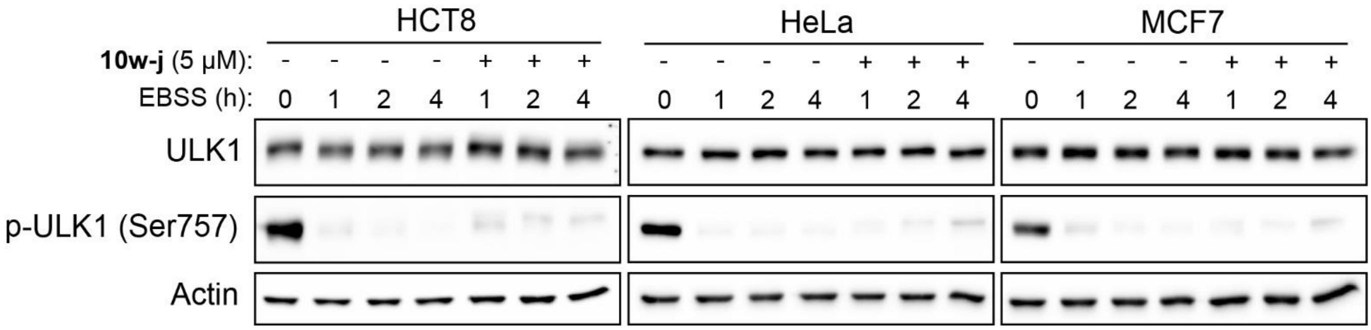

Figure S7 ULK1 phosphorylation incells (HCT8; HeLa; MCF7) undergoing starvation in the presence or absence of **10w-j**, as indicated.

2.0 Supporting Tables

2.1 Synthetic yields and autophagy inhibition IC_{50} values for Cinchona alkaloid-derived 7-azaindoles

Synthetic yields and autophagy IC_{50} values for the 7-azaindoles **9w** and **10w** (Table S1):

			From quinidine					From quinine				
**Entry**	**Functionalised at indole position…**	**R =**	**Nr**	**Yield /%**	**Starvation-induced IC_{50} /µM**	**Rapamycin-induced IC_{50} /µM**	**Notes**	**Nr**	**Yield /%**	**Starvation-induced IC_{50} /µM**	**Rapamycin-induced IC_{50} /µM**	**Notes**
**1**	n/a	n/a	**9w**	17	n/a	nd	*d*	**10w**	13	4.33 ± 1.7	4.95 ± 0.7	*a,e*
**2**	**4-R**-7-azaindole	Cl	–	–	–	–		**10w-a**	19	0.52 ± 0.20	0.65 ± 0.35	*a,d,g*
**3**	**5-R**-7-azaindole	Me	**9w-b**	51	n/a	n/a	*a*	**10w-b**	30	0.31 ± 0.09	0.86 ± 0.26	*a*
**4**	**5-R**-7-azaindole	4-Cl-(C_6H_5)-	–	–	–	–		**10w-c**	33	9.00 ± 1.1	n/a	*e,h*
**5**	**5-R**-7-azaindole	CF_3	–	–	–	–		**10w-d**	24	0.12 ± 0.03	0.77 ± 0.29	*e,g,h*
**6**	**5-R**-7-azaindole	NO_2	–	–	–	–		**10w-e**	10	0.67 ± 0.13	1.26 ± 0.20	*a,d,g*
**7**	**5-R**-7-azaindole	F	**9w-f**	38	n/a	n/a	*a*	**10w-f**	14	n/a	n/a	*a,e*
**8**	**5-R**-7-azaindole	Cl	–	–	–	–		**10w-g**	53	0.11 ± 0.04	0.85 ± 0.14	
**9**	**5-R**-7-azaindole	Br	–	–	–	–		**10w-h**	17	0.08 ± 0.03	0.81 ± 0.35	*a,e,g,h*
**10**	**5-R**-7-azaindole	I	–	–	–	–		**10w-i**	5	0.08 ± 0.02	1.24 ± 0.20	*a,g*
**11**	**5-Br-6-Me-azaindole**	N/A	–	–	–	–		**10w-j**	38	0.04 ± 0.02	0.10 ± 0.02	*e,g,h*
**12**	**6-R**-7-azaindole	Me	**9w-k**	13	n/a	n/a	*a,e*	**10w-k**	3	3.12 ± 0.5	5.11 ± 1.4	*a,e*
**13**	**6-R**-7-azaindole	Cl	–	–	–	–		**10w-l**	41	3.06 ± 0.9	6.21 ± 2.2	*e,h*

Table S1 A summary of the successful 7-azaindole formations using the Pd-catalysed annulation. Yields are unoptimised. Autophagy inhibition data are shown as the mean ± SD of three independent experiments (N = 3; $n \geq 3$). All compounds were initially assayed at a concentration of 10 µM. For hits reducing the number of LC3 puncta by more than 50%, IC_{50} values were determined. n/a = inactive (no reduction of LC3 puncta at 10 µM).

Standard conditions**:** ketone **5** or **6** (1.0 eq.), 2-iodoaniline (3.0 eq.), $Pd(OAc)_2$ (20 mol%), DABCO (3.0 eq.), DMF (0.3 M), 105 °C, 24 h. All reactions performed using 100 mg (0.32 mmol) ketone **5** or **6**. See General Procedure **B** for more details. See the Experimental Section for full details of any deviations denoted.

Notes: [a]30 mol% Pd(OAc)$_2$ used (3 x 10 mol% over 72 h). [b]10 mol% Pd(OAc)$_2$ used over 24 h. [c]The 2-iodoaniline HCl salt and 6.0 eq. DABCO were used. [d]Purified by mass-directed preparative HPLC to give the product as the TFA salt. [e]Purified by column chromatography followed by by mass-directed preparative HPLC to give the product as the TFA salt. [f]Purified by mass-directed preparative HPLC followed by column chromatography. [g]1.5 eq. MgSO$_4$ was added after 24 h. [h]Net yield from initial column before subsequent purification using mass-directed preparative HPLC.

2.2 Activity data for indole-containing natural products screened in the autophagy assay

Table S2 Activity data for indole-containing natural products screened in the the autophagy assay. All compounds were initially assayed at a concentration of 10 μM. No compounds reduced the number of LC3 puncta by more than 50%, and therefore IC_{50} values were not determined.

Entry	Trivial name	%activity (mean)	SMILES	Molecular Formula	CAS Registry No.	COMAS ID
1	Chondramide A; Demethoxy	104	C/C1=C/[C@@H](C)[C@H](C)OC(=O)C[C@H](c2ccc(O)cc2)NC(=O)[C@@H](Cc2c[nH]c3ccccc23)N(C)C(=O)[C@H](C)NC(=O)[C@H](C)C1	C35H44N4O6	172430-62-5	101415
2	Mozamide A; Deoxy	88	CC[C@H](C)[C@H](NC(=O)N[C@@H]1CCCCNC(=O)C(Cc2ccccc2)NC(=O)[C@H](Cc2c[nH]c3ccccc23)N(C)C(=O)[C@H](CC(C)C)NC(=O)[C@H](C(C)C)NC1=O)C(=O)O	C45H64N8O8	909119-50-2	104794
3	Koeniginequinone A	84	COc1ccc2c3c([nH]c2c1)C(=O)C=C(C)C3=O	C14H11NO3	110519-58-9	106116
4	Koeniginequinone A; 6-Methoxy	97	COc1cc2[nH]c3c(c2cc1OC)C(=O)C(C)=CC3=O	C15H13NO4	211183-78-7	106117
5	Carbazomycin G	96	COC1=C(C)C(C)(O)c2[nH]c3ccccc3c2C1=O	C15H15NO3	115920-44-0	106120
6	Carbazomycin G; 6-Methoxy	75	COC1=C(C)C(C)(O)c2[nH]c3ccc(OC)cc3c2C1=O	C16H17NO4	115920-42-8	106121
7	Dihydrogambirtannine; (S)-form, De(methoxycarbonyl)	92	c1ccc2c(c1)C[C@H]1c3[nH]c4ccccc4c3CCN1C2	C19H18N2	15026-41-2	106132
8	Rutaecarpine	97	O=c1c2ccccc2nc2n1CCc1c-2[nH]c2ccccc12	C18H13N3O	84-26-4	108014
9	Reserpic acid; 3-Epimer,O-(3,4,5-trimethoxycinnamoyl), Me ester	100	COC(=O)[C@H]1[C@H]2C[C@@H]3c4[nH]c5cc(OC)ccc5c4CCN3C[C@H]2C[C@@H](OC(=O)/C=C/c2cc(OC)c(OC)c(OC)c2)[C@@H]1OC	C35H42N2O9	187082-77-5	108015
10	Nitrarine; (±)-form	91	c1ccc2c3c([nH]c2c1)[C@H]1[C@@H]2CC[C@@H]([C@@H]4CCCN[C@@H]42)N1CC3	C20H25N3		108018
11	Ajmalicine; (-)-form	108	COC(=O)C1=CO[C@@H](C)[C@@H]2CN3CCc4c([nH]c5ccccc45)C3C[C@H]12	C21H24N2O3	483-04-5	108019
12	Corynantheine; 3,20-Diepimer, 18,19-dihydro	101	CC[C@H]1CN2CCc3c([nH]c4ccccc34)[C@@H]2C[C@@H]1/C(=C\OC)C(=O)OC	C22H28N2O3	7729-22-8	108027

Entry	Trivial name	%activity (mean)	SMILES	Molecular Formula	CAS Registry No.	COMAS ID
13	Yohimbine	101	COC(=O)C1[C@@H](O)CC[C@@H]2CN3CCc4c([nH]c5ccccc45)[C@@H]3C[C@H]12	C21H26N2O3	146-48-5	108028
14	Vincamine; (+)-form	97	CC[C@]12CCCN3CCc4c(n(c5ccccc45)[C@@](O)(C(=O)OC)C1)[C@@H]32	C21H26N2O3	1617-90-9	108029
15	2,3,4,9-Tetrahydro-1-methyl-1H-pyrido[3,4-b]indole-3-carboxylic acid; (1R,3S)-form	101	CC1N[C@H](C(=O)O)Cc2c1[nH]c1ccccc21	C13H14N2O2	42438-72-2	108032
16	Reserpine; (-)-form	112	COC(=O)[C@H]1[C@H]2C[C@H]3c4[nH]c5cc(OC)ccc5c4CCN3C[C@H]2C[C@@H](OC(=O)c2cc(OC)c(OC)c(OC)c2)[C@@H]1OC	C33H40N2O9	50-55-5	108033
17	1,2,3,4-Tetrahydro-6-hydroxy-β-carboline; Me ether	107	COc1ccc2[nH]c3c(c2c1)CCNC3	C12H14N2O	20315-68-8	108034
18	Evodiamine; (S)-form	103	CN1c2ccccc2C(=O)N2CCc3c([nH]c4ccccc34)C21	C19H17N3O	518-17-2	108040
19	Ergocornine	81	CC(C)[C@H]1C(=O)N2CCC[C@H]2[C@]2(O)O[C@](NC(=O)[C@@H]3C=C4c5cccc6[nH]cc(c56)C[C@H]4N(C)C3)(C(C)C)C(=O)N12	C31H39N5O5	564-36-3	108044
20	5-Hydroxytryptamine	99	NCCc1c[nH]c2ccc(O)cc12	C10H12N2O	50-67-9	108123
21	2,3,4,9-Tetrahydro-1H-pyrido[3,4-b]indole-3-carboxylic acid; (S)-form	106	O=C(O)C1Cc2c([nH]c3ccccc23)CN1	C12H12N2O2	42438-90-4	116230
22	1H-Indole-3-carboxylic acid	110	O=C(O)c1c[nH]c2ccccc12	C9H7NO2	771-50-6	124320
23	1H-Indole-3-propanoic acid; Methylamide	117	CNC(=O)CCc1c[nH]c2ccccc12	C12H14N2O	69397-85-9	127141
24	1H-Indol-3-ol; OH-form, O-Ac	108	CC(=O)Oc1c[nH]c2ccccc12	C10H9NO2	608-08-2	129257
25	1H-Indole-3-methanol	101	OCc1c[nH]c2ccccc12	C9H9NO	700-06-1	132775
26	Aplysinopsin; 3'-Deimino, 3'-oxo, $N^{2'}N^{4'}$-di-de-Me	87	O=C1NC(=O)/C(=C\c2c[nH]c3ccccc23)N1	C12H9N3O2		164577
27	Tryptamine	89	NCCc1c[nH]c2ccccc12	C10H12N2	61-54-1	165364
28	3,6-Diiodo-9H-carbazole	79	Ic1ccc2[nH]c3ccc(I)cc3c2c1	C12H7I2N	57103-02-3	166754
29	2,3,4,9-Tetrahydro-1,1-dimethyl-1H-pyrido[3,4-b]indole-3-carboxylic acid; (S)-form	95	CC1(C)NC(C(=O)O)Cc2c1[nH]c1ccccc21	C14H16N2O2	73198-03-5	172534
30	1H-Indole-3-butanoic acid	100	O=C(O)CCCc1c[nH]c2ccccc12	C12H13NO2	133-32-4	174746
31	1,2,3,4-Tetrahydro-1-methyl-β-carboline; (±)-form	85	CC1NCCc2c1[nH]c1ccccc21	C12H14N2	525-40-6	179383

Entry	Trivial name	%activity (mean)	SMILES	Molecular Formula	CAS Registry No.	COMAS ID
32	1H-Indole-3-acetic acid	100	O=C(O)Cc1c[nH]c2ccccc12	C10H9NO2	87-51-4	200918
33	Tryptophan; (S)-form	98	NC(Cc1c[nH]c2ccccc12)C(=O)O	C11H12N2O2	73-22-3	200975
34	Tryptophan; (S)-form, N^{α}-Ac	86	CC(=O)NC(Cc1c[nH]c2ccccc12)C(=O)O	C13H14N2O3	1218-34-4	201023
35	Staurosporine	98	CN[C@@H]1C[C@H]2O[C@@](C)([C@@H]1OC)n1c3ccccc3c3c4c(c5c6ccccc6n2c5c31)C(=O)NC4	C28H26N4O3	62996-74-1	245396
36	5-Methoxytryptamine; N^{b}-Ac	93	COc1ccc2[nH]cc(CCNC(C)=O)c2c1	C13H16N2O2	73-31-4	245497
37	Goniomitine	105	CC[C@@]12CCCN[C@@H]1n1c(c(CCO)c3ccccc31)CC2	C19H26N2O	109794-95-8	245755
38	7-Hydroxy-1-methyl-β-carboline; 3,4-Dihydro, Me ether	97	COc1ccc2c3c([nH]c2c1)C(C)=NCC3	C13H14N2O	304-21-2	283249
39	7-Hydroxy-1-methyl-β-carboline; 3,4-Dihydro	93	CC1=NCCc2c1[nH]c1cc(O)ccc21	C12H12N2O	525-57-5	246351
40	1-Methyl-β-carboline	85	Cc1nccc2c1[nH]c1ccccc12	C12H10N2	486-84-0	246352
41	7-Hydroxy-1-methyl-β-carboline; Me ether	81	COc1ccc2c(c1)[nH]c1c(C)nccc12	C13H12N2O	442-51-3	277591
42	7-Hydroxy-1-methyl-β-carboline	81	Cc1nccc2c1[nH]c1cc(O)ccc12	C12H10N2O	487-03-6	246354
43	6-Hydroxy-1-methyl-β-carboline; 3,4-Dihydro, Me ether	89	COc1ccc2[nH]c3c(c2c1)CCN=C3C	C13H14N2O	3589-73-9	246371
44	Catharanthine	86	CCC1=C[C@@H]2CN3CCc4c([nH]c5ccccc45)[C@@](C(=O)OC)(C2)[C@@H]13	C21H24N2O2	2468-21-5	246642
45	Eburnamine; (+)-form, 16-Ketone	86	CCC12CCCN3CCc4c(n(c5ccccc45)C(=O)C1)[C@@H]32	C19H22N2O	4880-88-0	284614
46	Yohimbine; Parent acid	104	O=C(O)[C@H]1[C@@H](O)CC[C@H]2CN3CCc4c([nH]c5ccccc45)[C@@H]3C[C@@H]21	C20H24N2O3	522-87-2	247974
47	Mahanine; (±)-form, Deoxy	98	CC(C)=CCCC1(C)C=Cc2c(c(C)cc3c2[nH]c2ccccc23)O1	C23H25NO	24948-14-9	277601
48	Reserpinine	93	COC(=O)C1=CO[C@@H](C)[C@@H]2CN3CCc4c([nH]c5cc(OC)ccc45)[C@@H]3C[C@H]12	C22H26N2O4	482-96-2	277617
49	Brevicarine	98	CNCCCCc1cnc(C)c2[nH]c3ccccc3c12	C17H21N3	25978-39-6	277645
50	Cyclo(tryptophyltryptophyl); (3R,6S)-form	97	O=C1N[C@@H](Cc2c[nH]c3ccccc23)C(=O)N[C@H]1Cc1c[nH]c2ccccc12	C22H20N4O2	175414-35-4	277679
51	1-(8-Quinolinyl)-β-carboline	94	c1cnc2c(-c3nccc4c3[nH]c3ccccc34)cccc2c1	C20H13N3	62209-25-0	277708
52	Vinblastine	133	CC[C@]1(O)C[C@@H]2CN(CCc3c([nH]c4ccccc34)[C@@](C(=O)OC)(c3cc4c(cc3OC)N(C)[C@H]3[C@@](O)(C(=O)OC)[C@H](OC	C46H58N4O9	865-21-4	278431

Entry	Trivial name	%activity (mean)	SMILES	Molecular Formula	CAS Registry No.	COMAS ID
			(C)=O)[C@]5(CC)C=CCN6CC[C@]43[C@@H]65)C2)C1			
53	Tombozine; N^4-Me	103	C/C=C1\C[N@@+]2(C)[C@H]3C[C@@H]1C(CO)[C@@H]2Cc1c3[nH]c2ccccc12	C20H25N2O	6792-07-0	278563
54	Agroclavine; (-)-form	145	CC1=C[C@@H]2c3cccc4[nH]cc(c34)C[C@H]2N(C)C1	C16H18N2	548-42-5	279968
55	β-Carboline-3-carboxylic acid; Me ester	101	COC(=O)c1cc2c(cn1)[nH]c1ccccc12	C13H10N2O2	69954-48-9	279993
56	Ergocristine	91	CC(C)[C@@]1(NC(=O)[C@@H]2C=C3c4cccc5[nH]cc(c45)C[C@H]3N(C)C2)O[C@@]2(O)[C@@H]3CCCN3C(=O)[C@H](Cc3ccccc3)N2C1=O	C35H39N5O5	511-08-0	279996
57	5-Hydroxytryptamine; N^b-Ac	99	CC(=O)NCCc1c[nH]c2ccc(O)cc12	C12H14N2O2	1210-83-9	280251
58	Tryptamine; N^b-Ac	101	CC(=O)NCCc1c[nH]c2ccccc12	C12H14N2O	1016-47-3	280288
59	Ellipticine	326	Cc1c2ccncc2c(C)c2c1[nH]c1ccccc12	C17H14N2	519-23-3	280538
60	5,6-Dihydroxytryptamine; 5-Me ether, N^b-Ac	123	COc1cc2c(CCNC(C)=O)c[nH]c2cc1O	C13H16N2O3	2208-41-5	280590
61	5-Hydroxy-1H-indole-3-acetic acid	94	O=C(O)Cc1c[nH]c2ccc(O)cc12	C10H9NO3	54-16-0	280613
62	5-Hydroxytryptophan; (S)-form	103	N[C@@H](Cc1c[nH]c2ccc(O)cc12)C(=O)O	C11H12N2O3	8/09/4350	280615
63	5-Hydroxytryptamine; N^b-Me	94	CNCCc1c[nH]c2ccc(O)cc12	C11H14N2O	1134-01-6	280703
64	Talopeptin	124	CC(C)CC(NP(=O)(O)O[C@@H]1O[C@@H](C)[C@H](O)[C@@H](O)[C@H]1O)C(=O)NC(Cc1c[nH]c2ccccc12)C(=O)O	C23H34N3O10P	84235-60-9	280895
65	Vincristine	145	CCC1(O)CC2CN(CCc3c([nH]c4ccccc34)[C@@](C(=O)OC)(c3cc4c(cc3OC)N(C=O)C3[C@@](O)(C(=O)OC)[C@H](OC(C)=O)[C@]5(CC)C=CCN6CC[C@]43[C@@H]65)C2)C1	C46H56N4O10	57-22-7	281038
66	Ergometrine	102	C[C@@H](CO)NC(=O)C1C=C2c3cccc4[nH]cc(c34)C[C@H]2N(C)C1	C19H23N3O2	60-79-7	282116
67	β-Carboline	84	c1ccc2c(c1)[nH]c1cnccc12	C11H8N2	244-63-3	282790
68	N-Methyltryptophan; (R)-form	107	CNC(Cc1c[nH]c2ccccc12)C(=O)O	C12H14N2O2	862504-05-0	282822
69	2,3,4,9-Tetrahydro-1H-pyrido[3,4-b]indol-1-one; N^2-(2-Methylaminobenzoyl)	81	CNc1ccccc1C(=O)N1CCc2c([nH]c3ccccc23)C1=O	C19H17N3O2	526-43-2	282896
70	Thienodolin	95	NC(=O)c1cc2c([nH]c3cc(Cl)ccc32)s1	C11H7ClN2OS	149127-27-5	283324
71	Telomycin	78	CC1NC(=O)C(C(C)O)NC(=O)C(NC(=O)C(CO)NC(=O)CC(N)C(=O)O)C(C)OC(=O)C2C(	C59H77N13O19	19246-24-3	283392

Entry	Trivial name	%activity (mean)	SMILES	Molecular Formula	CAS Registry No.	COMAS ID
			O)CCN2C(=O)C(C(O)C(C)C)NC(=O)C(C(C)c2c[nH]c3ccccc23)NC(=O)/C(=C/c2c[nH]c3ccccc23)NC(=O)C2C(O)CCN2C(=O)CNC1=O			
72	3,4-Dihydroxy-1,2-dimethylcarbazole; 3-Me ether	78	COc1c(C)c(C)c2[nH]c3ccccc3c2c1O	C15H15NO2	75139-38-7	283710
73	3-Greenwayodendrinol; 3α-form	62	CC1(C)C(O)CCC2(C)C1CCC1(C)C2Cc2cc3ccccc3n21	C23H31NO	85027-87-8	283728
74	Bis(7-hydroxygirinimbine A); 6,6'-Dimethoxy	96	COc1cc2c([nH]c3c4c(c(C)cc32)OC(C)(C)C=C4)c(-c2c(O)c(OC)cc3c2[nH]c2c4c(c(C)cc23)OC(C)(C)C=C4)c1O	C38H36N2O6	477890-82-7	283782
75	5-Carboxystrictosidine	116	C=C[C@H]1[C@H](O[C@@H]2O[C@H](CO)[C@@H](O)[C@H](O)[C@H]2O)OC=C(C(=O)OC)[C@H]1C[C@@H]1N[C@H](C(=O)O)Cc2c1[nH]c1ccccc21	C28H34N2O11	34371-11-4	283886
76	Sarpagine	102	C/C=C1/CN2C3CC1C(CO)C2Cc1c3[nH]c2ccc(O)cc12	C19H22N2O2	482-68-8	283899
77	Girinimbine; 8-Methoxy	98	COc1ccc2[nH]c3c4c(c(C)cc3c2c1)OC(C)(C)C=C4	C19H19NO2	21087-98-9	284383
78	Mahanimbicine; (+)-form, 7-Hydroxy	82	CC(C)=CCCC1(C)C=Cc2c(ccc3c2[nH]c2cc(O)c(C)cc23)O1	C23H25NO2	138876-26-3	284384
79	Fumitremorgin C	102	COc1ccc2c3c([nH]c2c1)C(C=C(C)C)N1C(=O)C2CCCN2C(=O)C1C3	C22H25N3O3	118974-02-0	284423
80	4,5-Dihydroxycanthin-6-one; Di-Me ether	75	COc1c(OC)c2nccc3c4ccccc4n(c1=O)c23	C16H12N2O3	18110-87-7	284476
81	1-Acetyl-β-carboline	80	CC(=O)c1nccc2c1[nH]c1ccccc12	C13H10N2O	50892-83-6	336423
82	3,6-Dibromo-9H-carbazole	160	Brc1ccc2[nH]c3ccc(Br)cc3c2c1	C12H7Br2N	6825-20-3	393546

2.3 Kinases showing *in vitro* inhibition below 10 μM by azaquindole-1

The following kinases were inhibited <10 μM by azaquindole-1 (compound **10w-j**, Table S3):

**Entry**	**Kinase**	**[ATP]** /μM	**IC$_{50}$** /nM
1	CLK4	n/a	93.9
2	PIK3C3 (VPS34)	Apparent K_M	350
3	PIK3C2G*	Apparent K_M	497
4	CLK2	Apparent K_M	572
5	PIK3C2B	100	2160
6	SPHK2	10	2770
7	PIK3CD/PIK3R1	Apparent K_M	4790
8	PI4KB*	Apparent K_M	5160

Table S3 Kinases inhibited at <10 μM by **10w-j**. *Potential roles in autophagy inhibition devalidated previously.[2]

For the complete list of kinases investigated in the study see Section 5.5.

2.4 Investigation of selective kinase inhibitors in the autophagy assay

To investigate any potential roles for the kinases shown in Table S3 in autophagy inhibition, we investigated potent and selective kinase inhibitors associated with each target in the autophagy assay (Table S4). The following kinases were inhibited <10 µM by azaquindole-1 (compound **10w-j**, see also Table S3):

Entry	Kinase(s)	Inhibitor	Short description	Starvation-induced IC_{50} /µM	Rapamycin-induced IC_{50} /µM	Inference
1	CLK4; CLK2	ML167	Inhibitor of CLK4 with IC_{50} = 136 nM, >10-fold selectivity over related related kinases CLK1 (1.52 µM), CLK2 (1.65 µM), CLK3 (>10 µM), DYRK1A (>10 µM), and DYRK1B (4.4 µM).[3,4]	n/a	n/a	Inhibition of CLK4 and/or CLK2 is not likely to be relevant to autophagy inhibition.
		TG003	Inhibitor of CLKs with IC_{50} = 15, 20 and 200 nM for CLK4, 1 and 2, respectively, and >10 µM for CLK3.[5] Also inhibits DYRK1A/B (IC_{50} = 24 and 34 nM respectively).[6]	n/a	n/a	
2	PIK3C2B	–	No selective inhibitors were commercially available.	–	–	–
3	SPHK2	ABC294640	Inhibitor of SPHK2 with IC_{50} = 60 µM, and >1.5 selectivity over SPHK1.[7]	n/a	n/a	Inhibition of SPHK2 is not likely to be relevant to autophagy inhibition.
		PF-543	Inhibitor of SPHK1 with IC_{50} = 2.7 nM. Inhibits SPHK2 with IC_{50} = 356 nM.[8]	n/a	n/a	
4	PIK3CD	IC-87114	Inhibitor of PI3Kδ with IC_{50} = 0.5 µM, >50-fold selectivity over PI3Kγ and PI3Kβ (IC_{50} = 29 and 75 µM, respectively) and >200-fold over PI3Kα (>100 µM).[9]	n/a	n/a	Inhibition of PIK3CD is not likely to be relevant to autophagy inhibition.
		Idelalisib (CAL-101)	Inhibitor of PI3Kδ with IC_{50} = 2.5nM, >30-fold selectivity over PI3Kγ (89 nM) and >200-fold over PI3Kα (820 nM), PI3Kβ (565 nM). ~400-fold activity over VPS34 (978 nM).[10]	5.34 ± 3.5	4.82 ± 2.4	
		Nemiralisib	Inhibitor of PI3Kδ with pK_i = 9.9, 1,000-fold selective over PI3Kα, β, and γ isoforms.[11]	n/a	n/a	

Table S4 Investigation of selective kinase inhibitors in the autophagy assay. Autophagy inhibition data are shown as the mean ± SD of three independent experiments (N = 3; $n \geq 3$). All compounds were initially assayed at a concentration of 10 µM. For hits reducing the number of LC3 puncta by more than 50%, IC_{50} values were determined. n/a = inactive (no reduction of LC3 puncta at 10 µM).

2.5 *In vitro* IC_{50} measurements for azaquindoles and related structures against VPS34

The following compounds were assessed for inhibition (<10 μM) of VPS34 (Table S5):

**Entry**	**Compound No.**	**Compound Structure**	**[ATP]** /μM	IC_{50} /nM
1	**10w-j**	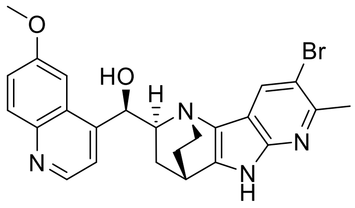 **10w-j**	Apparent K_M	350
2	**10w-i**	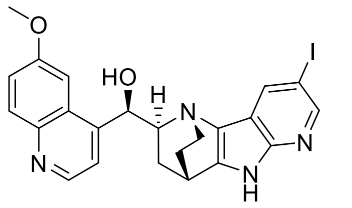 **10w-i**	Apparent K_M	627
3	**10w-b**	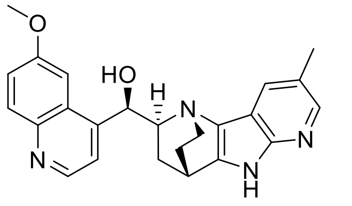 **10w-b**	Apparent K_M	656
4	**10w-k**	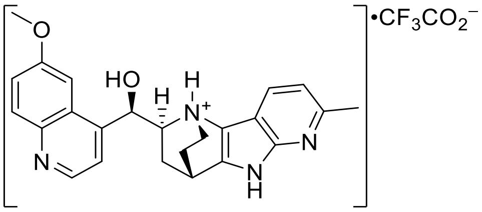 **10w-k**	Apparent K_M	3280
5	**S-1**	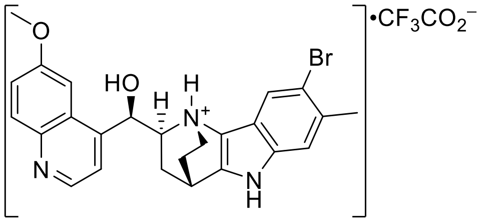 **S-1**	Apparent K_M	>10000

Table S5 Kinases inhibited at <10 μM by compounds **10w** and related structures.

3.0 Pd-catalysed indole formations: substrate scope summary

In initial investigations (manuscript Figure 2), 43/62 (69%) of Pd-catalysed indole formations attempted were successful (see Section 3.2 for failed reaction scope). In the subsequent SAR investigations around the initial hit compounds **10u** and **10w**, 3/6 (50%) and 15/22 (68%), of the additional reactions attempted were successful respectively (see Section 3.4 for failed reaction scope). Overall 61/90 (68%) of the attempted indole formations using the Pd-catalysed annulation were successful (excluding the compounds from Figure S3).

3.1 Substrate scope experiments: successful reactions

The yields for the successful reactions are summarised below in Table S6.

			From quinidine			From quinine		
**Entry**	**Functionalised at indole position…**	**R =**	**Nr**	**Yield /%**	**Notes**	**Nr**	**Yield /%**	**Notes**
1	**4/5/6/7 = R**	H	**9a**	49		**10a**	17	*b,f*
2	**4 = R**; 5/6/7 = H	Me	**9b**	49		**10b**	46	
3	**4 = R**; 5/6/7 = H	CO_2Me	**9c**	18	*a*	**10c**	12	*a*
4	**4 = R**; 5/6/7 = H	Cl	**9d**	77	*b*	**10d**	10	*b*
5	**5 = R**; 4/6/7 = H	Me	**9e**	45		**10e**	17	*f*
6	**5 = R**; 4/6/7 = H	CO_2H	**9f**	14	*a,d,f*	**10f**	9	*b,d*
7	**5 = R**; 4/6/7 = H	CO_2Me	**9g**	3	*a,e*	–	0	
8	**5 = R**; 4/6/7 = H	CF_3	**9h**	25		**10h**	23	*a*
9	**5 = R**; 4/6/7 = H	NO_2	**9i**	12	*a,e*	**10i**	3	*a,e*
10	**5 = R**; 4/6/7 = H	OMe	**9j**	23	*a,c*	**10j**	6	*a*
11	**5 = R**; 4/6/7 = H	OCF_3	–	nd		**10k**	65	*b*
12	**5 = R**; 4/6/7 = H	F	**9l**	19	*d*	**10l**	47	
13	**5 = R**; 4/6/7 = H	Cl	**9m**	48		**10m**	59	*b,d*
14	**5 = R**; 4/6/7 = H	Br	**9n**	8	*e*	**10n**	15	
15	**6 = R**; 4/5/7 = H	Me	**9o**	12	*a,e*	**10o**	4	
16	**6 = R**; 4/5/7 = H	CO_2H	**9p**	11	*b,d*	**10p**	14	*d*
17	**6 = R**; 4/5/7 = H	CO_2Me	**9q**	7	*a*		0	
18	**6 = R**; 4/5/7 = H	CF_3	**9r**	25		**10r**	70	*b*
19	**6 = R**; 4/5/7 = H	Cl	**9s**	26		**10s**	20	
20	**6 = R**; 4/5/7 = H	Br	**9t**	9	*a,e*	**10t**	27	*a*
21	**7 = R**; 4/5/6 = H	OMe	**9u**	16	*a,e*	**10u**	33	
22	**4-azaindole**	N/A	–	0		–	nd	
23	**5-azaindole**	N/A	–	0		–	nd	
24	**6-azaindole**	N/A	**9v**	50	*d*	**10v**	65	*b*
25	**7-azaindole**	N/A	**9w**	17	*d*	**10w**	13	*a,e*

Table S6 A summary of the successful substrate scope and yields for the indole formations using the Pd-catalysed annulation. Some notable failed substrates are included (Entries 7, 17, 22 and 23. See also Section 3.2). Yields are unoptimised.

Standard conditions: ketone **5** or **6** (1.0 eq.), 2-iodoaniline (3.0 eq.), $Pd(OAc)_2$ (20 mol%), DABCO (3.0 eq.), DMF (0.3 M), 105 °C, 24 h. All reactions performed using 100 mg (0.32 mmol) ketone **5** or **6** See General Procedure **B** for more details. See the Experimental Section for full details of any deviations denoted.

Notes: [a]30 mol% $Pd(OAc)_2$ used (3 x 10 mol% over 72 h). [b]10 mol% $Pd(OAc)_2$ used over 24 h. [c]The 2-iodoaniline HCl salt and 6.0 eq. DABCO were used. [d]Purified by mass-directed preparative HPLC to give the product as the TFA salt. [e]Purified by column chromatography followed by by mass-directed preparative HPLC to give the product as the TFA salt. [f]Purified by mass-directed preparative HPLC followed by column chromatography. N/A = not applicable. nd = not determined (reaction not carried out).

3.2 Substrate scope experiments: failed reactions

The following reactions failed during investigation of the substrate scope of the Pd-catalysed indole formation:

Entry	2-Iodoaniline	Ketone	Notes
**1**	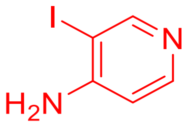 4-amino-3-iodopyridine	**5**	not observed by LCMS after 24 h
**2**	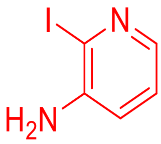 3-amino-2-iodopyridine	**5**	not observed by LCMS after 24 h
**3**	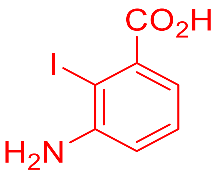 3-amino-2-iodobenzoic acid	**6**	not observed by LCMS after 24 h
**4**	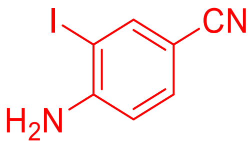 4-amino-3-iodobenzonitrile	**6**	not observed by LCMS after 24 h
**5**	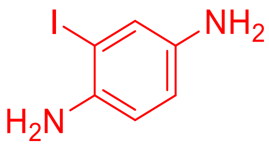 2-iodo-1,4-benzenediamine	**6**	not observed by LCMS after 24 h
**6**	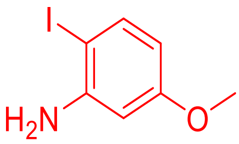 2-iodo-5-methoxyaniline	**6**	not observed by LCMS after 24 h

Entry	2-Iodoaniline	Ketone	Notes
**7**	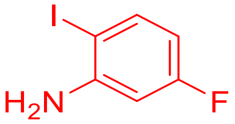<br>5-fluoro-2-iodoaniline	**5**	trace by LCMS (<5%) after 72 h (30 mol% Pd(OAc)2, unable to isolate cleanly
**8**	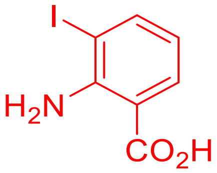<br>2-amino-3-iodobenzoic acid	**6**	not observed by LCMS after 24 h
**9**	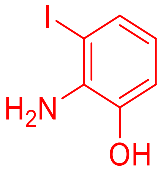<br>2-amino-3-iodo-phenol	**6**	not observed by LCMS after 24 h
**10**	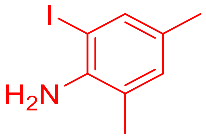<br>2-iodo-4,6-dimethylaniline	**5**	not observed by LCMS after 24 h
**11**	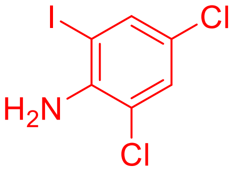<br>2,4-dichloro-6-iodoaniline	**6**	trace by LCMS (<10%) after 72 h (30 mol% Pd(OAc)2, unable to isolate cleanly

Table S7 Failed reactions in substrate scope studies. *Conditions:* ketone **5** or **6** (1.0 eq.), 2-iodoaniline (3.0 eq.), Pd(OAc)2 (20 mol%), DABCO (3.0 eq.), DMF (0.3 M), 105 °C, 24 h). All reactions performed using 100 mg (0.32 mmol) ketone **5** or **6**.

3.3 SAR investigations: successful reactions

In SAR investigations around compound **10u**, 3/6 (50%) additional reactions attempted were successful (Table S8; see Section 3.4 for the failed reaction scope):

			From quinine		
**Entry**	**Functionalised at indole position…**	**R =**	**Nr**	**Yield /%**	**Notes**
1	**7 = R**; 4/5/6 = H	F	**10x**	18	*a*
2	**7 = R**; 4/5/6 = H	Cl	**10y**	53	*b*
3	**7-OMe-5-(CO_2Me)-indole**	N/A	**10z**	1	*a,c,d*

Table S8 A summary of the successful 7-subsituted indole formations using the Pd-catalysed annulation. Yields are unoptimised.

Standard conditions: ketone **5** or **6** (1.0 eq.), 2-iodoaniline (3.0 eq.), Pd$(OAc)_2$ (20 mol%), DABCO (3.0 eq.), DMF (0.3 M), 105 °C, 24 h. All reactions performed using 100 mg (0.32 mmol) ketone **5** or **6**. See General Procedure **B** for more details. See the Experimental Section for full details of any deviations denoted.

Notes: [a]30 mol% Pd$(OAc)_2$ used (3 x 10 mol% over 72 h). [b]10 mol% Pd$(OAc)_2$ used over 24 h. [c]1.5 eq. $MgSO_4$ was added after 24 h. [d]Purified by column chromatography followed by by mass-directed preparative HPLC to give the product as the TFA salt.

The biological activities of the above compounds in the autophagy assay were as follows:

Entry	Compound	R-group and position	Starvation-induced IC_{50} /μM	Rapamycin-induced IC_{50} /μM
1	**10x**	7-F	n/a	n/a
2	**10y**	7-Cl	n/a	n/a
3	**10z**	7-OMe-5-(CO_2Me)-	2.25 ± 0.1	2.33 ± 0.5

Table S9 Inhibition of starvation and/or rapamycin-induced autophagy by analogues of compound **10u**. All data are shown as mean ± SD of three independent experiments (N = 3; n = 3). All compounds were initially assayed at a concentration of 10 μM. For hits reducing the number of LC3 puncta by more than 50%, IC_{50} values were determined. n/a = inactive (no reduction of LC3 puncta at 10 μM). Ar = 4-Cl-(C_6H_4).

In SAR investigations around compound **10w**, 15/22 (68%) of reactions attempted were successful (Table S10; see Section 3.4 for failed reaction scope):

			From quinidine			From quinine		
**Entry**	**Functionalised at indole position…**	**R =**	**Nr**	**Yield /%**	**Notes**	**Nr**	**Yield /%**	**Notes**
**1**	**4-R**-7-azaindole	Cl				**10w-a**	19	*a,d,g*
**2**	**5-R**-7-azaindole	Me	**9w-b**	51	*a*	**10w-b**	30	*a*
**3**	**5-R**-7-azaindole	4-Cl-(C_6H_5)-	–	–		**10w-c**	33	*e,h*
**4**	**5-R**-7-azaindole	CF_3	–	–		**10w-d**	24	*e,g,h*
**5**	**5-R**-7-azaindole	NO_2	–	–		**10w-e**	10	*a,d,g*
**6**	**5-R**-7-azaindole	F	**9w-f**	38	*a*	**10w-f**	14	*a,e*
**7**	**5-R**-7-azaindole	Cl	–	–		**10w-g**	53	
**8**	**5-R**-7-azaindole	Br	–	–		**10w-h**	17	*a,e,g,h*
**9**	**5-R**-7-azaindole	I	–	–		**10w-i**	5	*a,g*
**10**	**5-Br-6-Me-azaindole**	N/A	–	–		**10w-j**	38	*e,g,h*
**11**	**6-R**-7-azaindole	Me	**9w-k**	13	*a,e*	**10w-k**	3	*a,e*
**12**	**6-R**-7-azaindole	Cl	–	–		**10w-l**	41	*e,h*

Table S10 A summary of the successful 7-azaindole formations using the Pd-catalysed annulation. Yields are unoptimised.

***Standard conditions*:** ketone **5** or **6** (1.0 eq.), 2-iodoaniline (3.0 eq.), $Pd(OAc)_2$ (20 mol%), DABCO (3.0 eq.), DMF (0.3 M), 105 °C, 24 h). All reactions performed using 100 mg (0.32 mmol) ketone **5** or **6**. See General Procedure **B** for more details. See the Experimental Section for full details of any deviations denoted.

Notes: [a]30 mol% $Pd(OAc)_2$ used (3 x 10 mol% over 72 h). [b]10 mol% $Pd(OAc)_2$ used over 24 h. [c]The 2-iodoaniline HCl salt and 6.0 eq. DABCO were used. [d]Purified by mass-directed preparative HPLC to give the product as the TFA salt. [e]Purified by column chromatography followed by by mass-directed preparative HPLC to give the product as the TFA salt. [f]Purified by mass-directed preparative HPLC followed by column chromatography. [g]1.5 eq. $MgSO_4$ was added after 24 h. [h]Net yield from column before subsequent purification using mass-directed preparative HPLC.

3.4 SAR investigations: failed reactions

The following reactions failed during SAR studies to investigate the preparation of analogues of compound **10u** using the Pd-catalysed indole formation (note that Entries 1 and 3 were investigated in the initial substrate scoping experiments [see Table S7] and are included here for completeness):

Entry	2-Iodoaniline
**1***	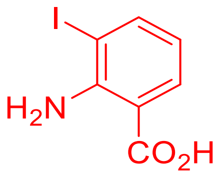<br>2-amino-3-iodobenzoic acid
**2**	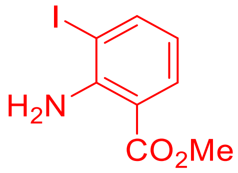<br>methyl 2-amino-3-iodobenzoate
**3***	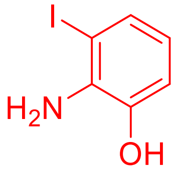<br>2-amino-3-iodo-phenol
**4**	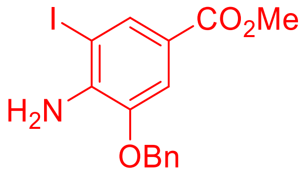<br>methyl 4-amino-3-(benzyloxy)-5-iodobenzenecarboxylate
**5**	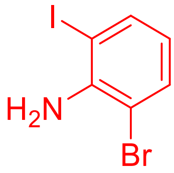<br>2-bromo-6-iodoaniline

Table S11 Failed reactions in SAR studies. *Conditions:* ketone **6** (1.0 eq.), 2-iodoaniline (3.0 eq.), $Pd(OAc)_2$ (20 mol%), DABCO (3.0 eq.), DMF (0.3 M). When no conversion was observed by LCMS after 24 h, additional $Pd(OAc)_2$ (10 mol%) and $MgSO_4$ (1.5 eq.) were added. The reaction mixture was degassed with Ar (10 min), then heated at 105 °C for a further 24 h. All reactions performed using 100 mg (0.32 mmol) ketone **6**. All reactions were monitored by LCMS. *Investigated in original substrate scoping reactions, see Table S7.

The following reactions failed during SAR studies to investigate the preparation of analogues of compound **10w** using the Pd-catalysed indole formation:

Entry	2-Iodoaniline
**1**	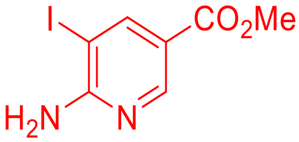<br>methyl 6-amino-5-iodopyridine-3-carboxylate
**2**	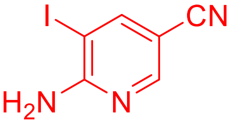<br>6-amino-5-iodonicotinonitrile
**3**	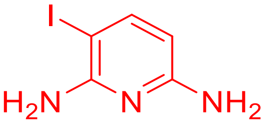<br>3-iodopyridine-2,6-diamine
**4**	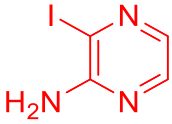<br>2-amino-3-iodopyrazine
**5**	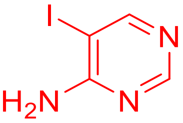<br>4-amino-5-iodopyrimidine
**6**	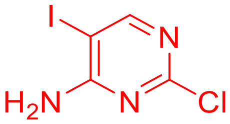<br>4-amino-2-chloro-5-iodopyrimidine
**7**	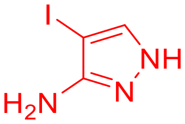<br>3-Amino-4-iodo-1H-pyrazole

Table S12 Failed reactions in SAR studies. *Conditions:* ketone **6** (1.0 eq.), 2-iodoaniline (3.0 eq.), $Pd(OAc)_2$ (20 mol%), DABCO (3.0 eq.), DMF (0.3 M). When no conversion was observed by LCMS after 24 h, additional $Pd(OAc)_2$ (10 mol%) and $MgSO_4$ (1.5 eq.) were added. The reaction mixture was degassed with Ar (10 min), then heated at 105 °C for a further 24 h. All reactions performed using 100 mg (0.32 mmol) ketone **6**. All reactions were monitored by LCMS.

4.0 Computational analysis

4.1 Compounds in the analysis

The following compounds were used to construct Figures 2c-d in the manuscript along with the molecular property analysis in Figure S2. A total of 55 compounds were included in the study:- indoquinidines (22 compounds, black); indoquinines (20 compounds, blue); azaquindoles (13 compounds, pink).

indoquinidines 9a-w

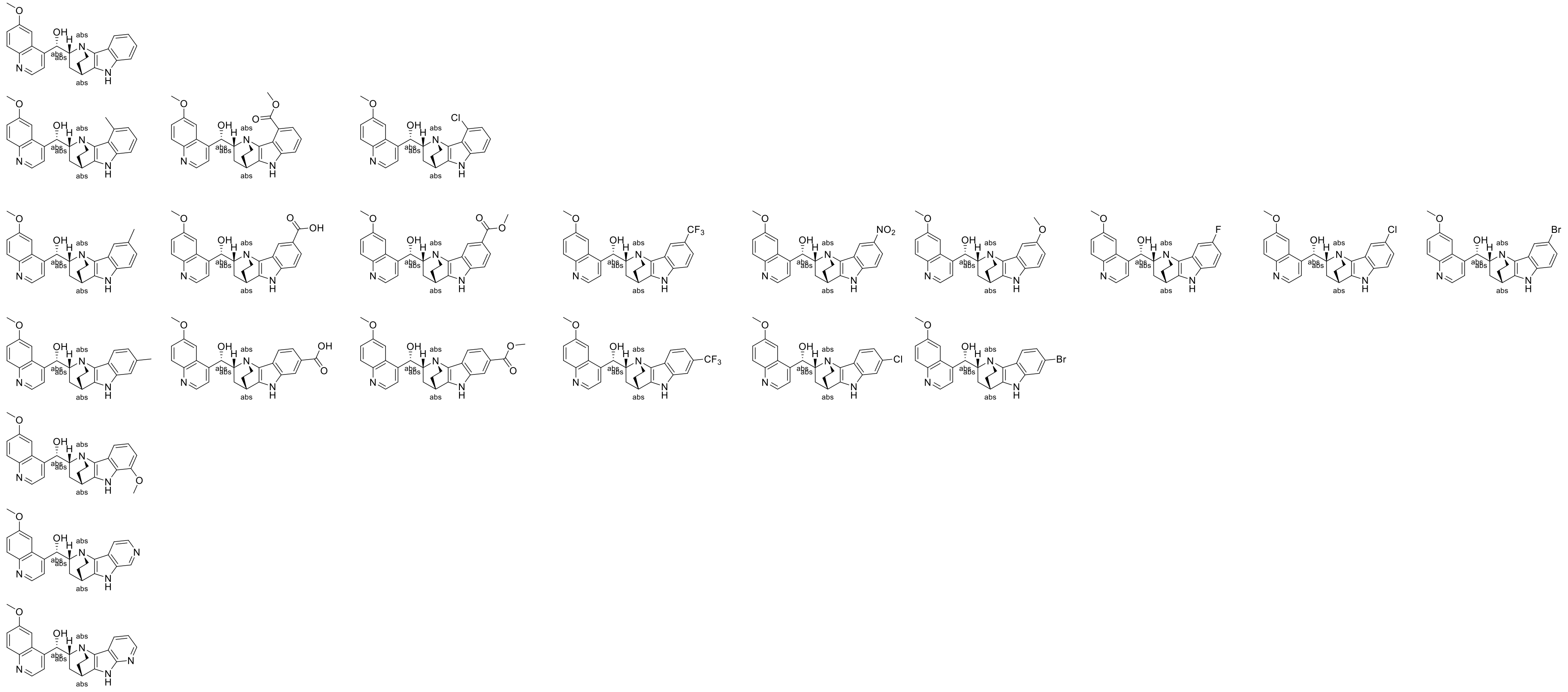

Indoquinines 10a-v

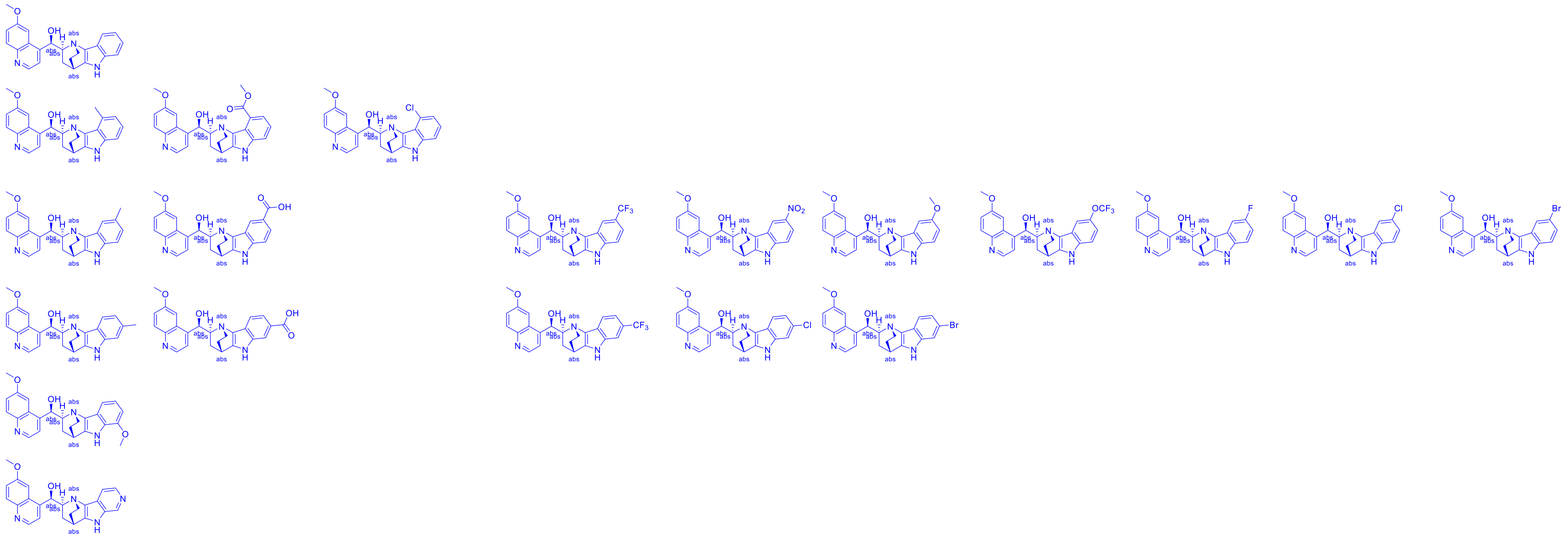

7-azaindoquinines 10w to 10w-l

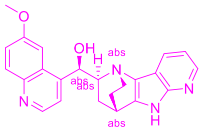

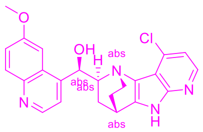

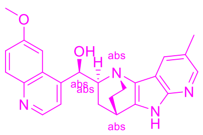

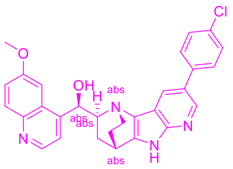

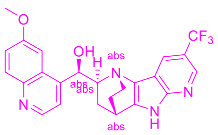

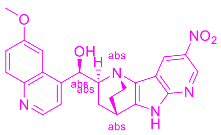

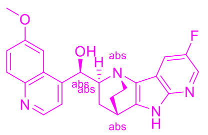

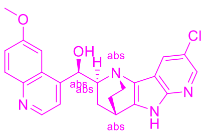

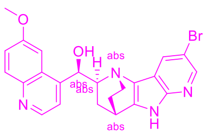

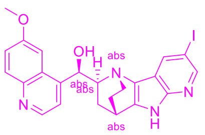

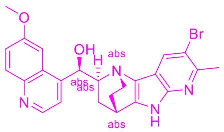

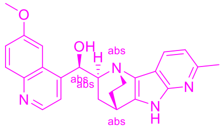

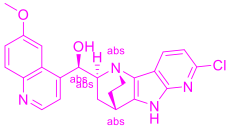

4.2 NP-likeness scoring

Natural product likeness scores were calculated using the method of Ertl *et al*.[12] using the implementation in RDKit v2017.09.1 (Greg Landrum; Open Source Cheminformatics Software; http://www.rdkit.org; last accessed 28/05/2018).

For the preparation of Figure 2d, the following databases were used:

- Drugbank Approved + Experimental v5.1.0 (7360 compounds [2325 + 5035], https://www.drugbank.ca/releases/latest#structures; downloaded 28/05/2018).

- ChEMBL v23 (35k unique compounds with *J. Nat. Prod.* as source document, release date May 2017, downloaded 28/05/2018).

4.3 Molecular properties analysis

The following data were calculated using LLAMA.[13]

See also Figure S2, Section 1.2.

A summary of the molecular properties of the cinchona alkaloids, quinidine and quinine, is given below:

Entry	Property	Quinidine	Quinine
1	**MW /Da**	324	324
2	**ALogP**	2.73	2.73
3	**tPSA**	45.6	45.6
4	**Fsp^3**	0.45	0.45

Table S13 Molecular properties of the Cinchona alkaloids.

A summary of the molecular properties of the indocinchona library, including azaindoles **10w**, is given below:

Entry	Property	Range	Average
1	**MW /Da**	385-512	427
2	**ALogP**	2.74-5.14	3.94
3	**tPSA**	61-120	73
4	**Fsp^3**	0.24-0.33	0.30

Table S14 Molecular properties of the indocinchona alkaloid library.
Compounds included are detailed in Section 4.1 above (55 compounds in total).

A summary of the molecular properties of the 7-azaindoquinines **10w** to **10w-j** is given below:

Entry	Property	Range	Average
1	**MW /Da**	386-512	438
2	**ALogP**	3.06-5.14	3.86
3	**tPSA**	74-120	78
4	**Fsp**^{3}	0.24-0.33	0.31

Table S15 Molecular properties of the 7-azaindoquinines **10w**.
Compounds **10w** to **10w-j** are detailed in Section 4.1 above (13 compounds in total).

4.4 Novelty assessment

See Section 1.1, Figure S1, for substructure searches within the Dictionary of Natural Products.

A Chemical Abstracts Service (CAS) search using SciFinder (https://scifinder.cas.org, accessed 28/05/2018), and a Reaxys search (www.reaxys.com, accessed 28/05/2018), revealed that the indocinchona scaffold is unknown (Figure S8). One simple example of a 1,9 diazatetracyclo[9.2.2.02,10.03,8]pentadeca-2(10),3,5,7-tetraene (fused indole/quinuclidine) ring-system was found in the scientific literature, whilst eight simple examples were found in the patent literature (references given below in Table S16). To the best of our knowledge no activity against kinases has been reported.

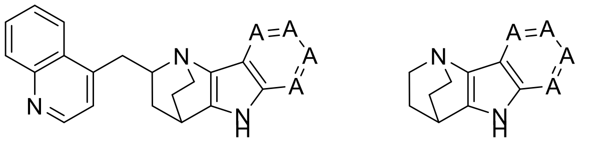

0 hits

9 hits
(see the Table below)

Figure S8 Substructure searches within CAS.

Entry	Structure	Reference(s)
**1**	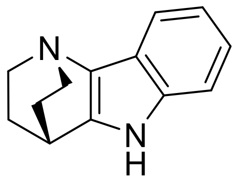	1. C. Chen, D. R. Lieberman, R. D. Larsen, T. R. Verhoeven, P. J. Reider, *J. Org. Chem.* **1997**, *62*, 2676–2677.<br>2. C. Chen, R. D. Larsen, inventors; Merck & Co., assignee. Palladium catalyzed indolization. *US Patent 5,811,551*, **1998**. Filed Aug 7 1997, issued Sept 22 1998.<br>3. M. R. Schrimpf, C.-H. Lee, T. Li, G. A. Gfesser, K. H. Mortell, R. Faghih, D. L. Nersesian, K. B.

Entry	Structure	Reference(s)
		Sippy, W. H. Bunnelle, M. Scanio, L. Shi, M. Gopalakrishnan, D. L. Donnelly-Roberts, M. Hu, inventors; AbbVie Inc., assignee. Indole and indoline derivatives and methods of use thereof. *US Patent 9,625,475 (B2)*, **2017**. Filed March 19 2010, issued April 18 2017.
**2**	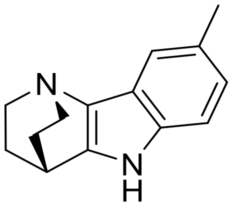	(see Entry 1, Ref. 3)
**3**	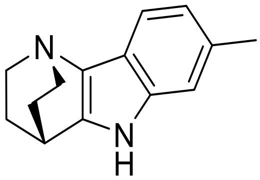	(see Entry 1, Ref. 3)
**4**	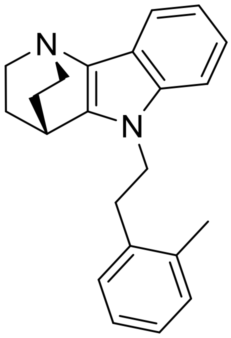	(see Entry 1, Ref. 3)
**5**	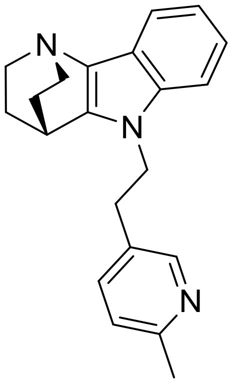	(see Entry 1, Ref. 3)

Entry	Structure	Reference(s)
**6**	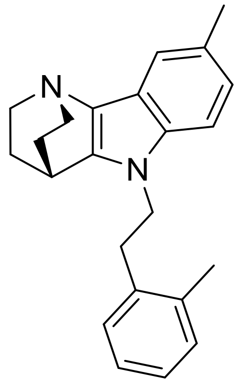	(see Entry 1, Ref. 3)
**7**	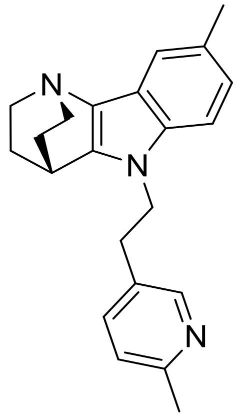	(see Entry 1, Ref. 3)
**8**	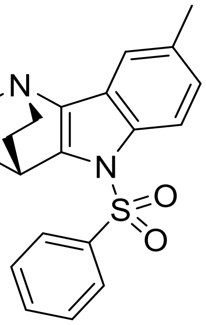	(see Entry 1, Ref. 3)
**9**	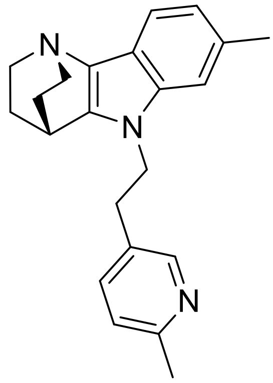	(see Entry 1, Ref. 3)

Table S16 Hits for the substructure search (Figure S8) within Reaxys and SciFinder.

5.0 Biological experimental

5.1 Cell culture for the autophagy assay

MCF7 cells stably transfected with eGFP-LC3 (MCF7-GFP-LC3) were cultured at 37 °C with 5% CO_2 using Eagle's MEM (Gibco cat# 21090-022) containing 10% FBS (Invitrogen cat# 10500-084), 1% L-Glutamine (Invitrogen cat# 25030-081), 1% sodium pyruvate (PAN Biotech cat# P04-43100), 1% NEAA (PAN Biotech cat# P08-32100), 0.01 mg/mL bovine insulin (Sigma Aldrich cat# I9278) and 200 µg/ml G418 as the medium.

MCF7 wt (#2011) cells were cultured in standard DMEM (PAN Biotech, cat# P04-03500) supplemented with 10% FBS (Invitrogen cat# 10500-084), 1% sodium pyruvate (PAN Biotech, cat# P04-43100), 1% NEAA (PAN Biotech, cat# P08-32100) and 0.01 mg/mL bovine insulin (Sigma Aldrich cat# I9278) at 37 °C with 5% CO_2.

HeLa, Hek293T and Hek293A EGFP-WIPI2b cells were cultured in standard DMEM (PAN Biotech, cat# P04-03500) supplemented with 10% FBS (Invitrogen cat# 10500-084), 1% sodium pyruvate (PAN Biotech, cat# P04-43100) and 1% NEAA (PAN Biotech, cat# P08-32100) at 37 °C with 5% CO_2.

HCT8 cells were cultured in Roswell Park Memorial Institute (RPMI) 1640 medium (Sigma-Aldrich, cat# R8758) supplemented with 10% FBS(Invitrogen cat# 10500-084) and 1% NEAA (PAN Biotech, cat# P08-32100) at 37°C with 5% CO_2.

5.2 High-content screening for autophagy inhibitors

For the phenotypic autophagy assay, 4000 MCF7-GFP-LC3 cells per well were seeded in 25 µL medium in a 384 well Greiner µclear plate (cat# 781080, lid cat# 656191) and incubated (37 °C, 5% CO_2) overnight. Cells were then washed by a plate washer (Biotek, ELx405) three times with 1X PBS followed by a final aspiration of the washing buffer. The addition of 25 nL of compound solution (10 mM stock solution in DMSO) was then carried out with an echo dispenser (Labcyte, Echo 520 dispenser). Addition of medium to induce autophagy was carried out with a Multidrop Combi (Thermo Scientific). 25 µL Earle's Balanced Salt Solution (EBSS, Sigma Aldrich, cat# E3024-500mL) containing 50 µM Chloroquine (Sigma Aldrich, cat# C6628-25g) was used for starvation-induced autophagy and 25 µL medium containing 50 µl Chloroquine and 100 nM Rapamycin (Biomol, cat# Cay13346)-1 was used for rapamycin-induced autophagy screening. After incubation (37 °C, 5% CO_2) for three hours cells were fixed by addition of 25 µL 1:4 formaldehyde in 1X PBS + 1:500 Hoechst (stock: 1 mg/mL, Sigma Aldrich cat# B2261-25 mg) and incubation for 20 min at

room temperature. Cells were then washed three times with 1X PBS. Four images per well were taken with ImageXpress Micro XL (Molecular Devices) at 20x. Automated image analysis was performed using the granularity setting of MetaXpress Software (Molecular Devices).

5.3 Cell painting assay

The described assay closely follows the method described by Bray *et al.*[14]

Initially, 5 µl U2OS medium were added to each well of a 384-well plate (PerkinElmer CellCarrier-384 Ultra). Subsequently, U2OS cell were seeded with a density of 1600 cells per well in 20 µl medium. The plate was incubated for 10 min at the ambient temperature, followed by an additional 4 h incubation (37 °C, 5% CO2). Compound treatment was performed with the Echo 520 acoustic dispenser (Labcyte) at final concentrations of 10 µM, 3 µM or 1 µM. Incubation with compound was performed for 20 h (37 °C, 5% CO2). Subsequently, mitochondria were stained with Mito Tracker Deep Red (Thermo Fisher Scientific, Cat. No. M22426). The Mito Tracker Deep Red stock solution (1 mM) was diluted to a final concentration of 100 nM in prewarmed medium. The medium was removed from the plate leaving 10 µl residual volume and 25 µl of the Mito Tracker solution were added to each well. The plate was incubated for 30 min in darkness (37 °C, 5% CO2). To fix the cells 7 µl of 18.5 % formaldehyde in PBS were added, resulting in a final formaldehyde concentration of 3.7 %. Subsequently, the plate was incubated for another 20 min in darkness (RT) and washed three times with 70 µl of PBS. (Biotek Washer Elx405). Cells were permeabilized by addition of 25 µl 0.1% Triton X-100 to each well, followed by 15 min incubation (RT) in darkness. The cells were washed three times with PBS leaving a final volume of 10 µl. To each well 25 µl of a staining solution were added, which contains 1% BSA, 50 µl Phalloidin (Alexa594 conjugate, Thermo Fisher Scientific, A12381), 25 µg/ml Concanavalin A (Alexa488 conjugate, Thermo Fisher Scientific, Cat. No. C11252), 50 µl/ml Hoechst 33342 (Sigma, Cat. No. B2261-25mg), 15 µl/ml WGA-Alexa594 conjugate (Thermo Fisher Scientific, Cat. No. W11262) and 0.3 µl/ml SYTO 14 solution (Thermo Fisher Scientific, Cat. No. S7576). The plate is incubated for 30 min (RT) in darkness and washed three times with 70 µl PBS. After the final washing step the PBS was not aspirated. The plates were sealed and centrifuged for 1 min at 500 rpm.
The plates were prepared in triplicates with shifted layouts to reduce plate effects and imaged using a Micro XL High-Content Screening System (Molecular Devices) in 5 channels (DAPI: Ex350-400/ Em410-480; FITC: Ex470-500/ Em510-540; Spectrum Gold: Ex520-545/ Em560-585; TxRed: Ex535-585/ Em600-650; Cy5: Ex605-650/ Em670-715) with 9 sites per well and 20x magnification (binning 2).

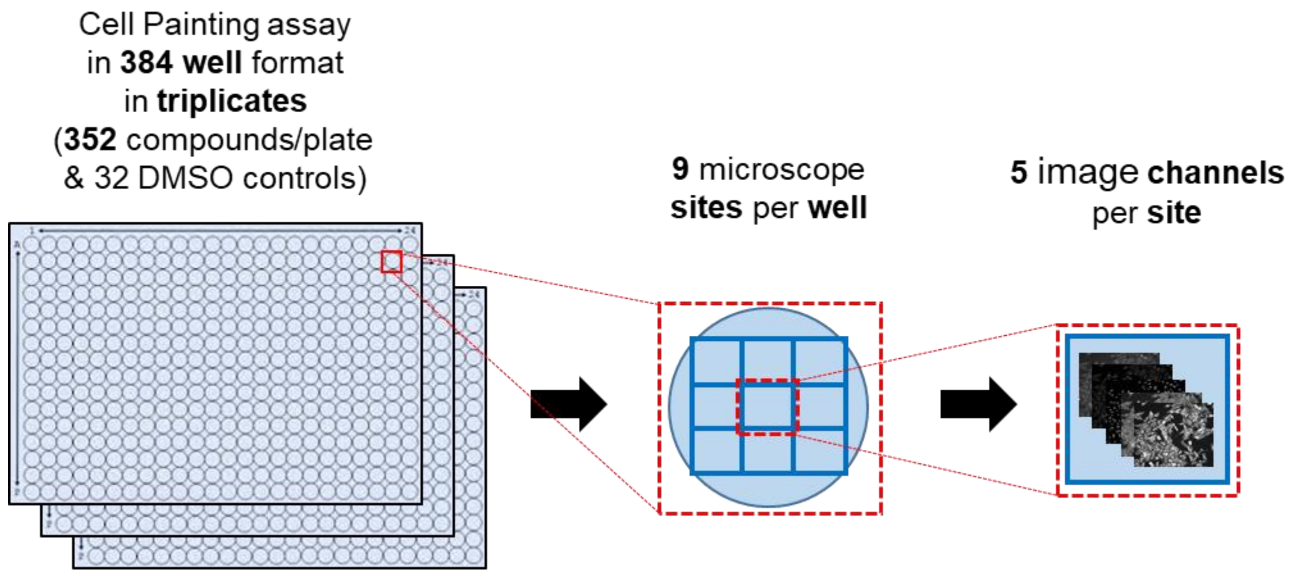

The generated images were processed with the CellProfiler package (https://cellprofiler.org/, version 3.0.0, Git commit 235f8251bd8a39b04bc68fa1511d5b5dccf828d5) on a computing cluster of the Max Planck Society to extract 1716 cell features (parameters) per microscope site. The data was then further aggregated as medians per well (9 sites -> 1 well), then over the three replicates.
Further analysis was performed with custom Python (https://www.python.org/) scripts using the Pandas (https://pandas.pydata.org/) and Dask (https://dask.org/) data processing libraries (separate publication to follow).

From the total set of 1716 parameters a subset of highly reproducible and robust parameters was determined using the procedure described by Woehrmann *et al*.[15] in the following way: Two biological repeats of one plate containing reference compounds were analysed. For every parameter, its full profile over each whole plate was calculated. If the profiles from the two repeats showed a similarity >= 0.8 (see below), the parameter was added to the set.
This procedure was only performed once and resulted in a set of 579 robust parameters out of the total of 1716 that was used for all further analyses.

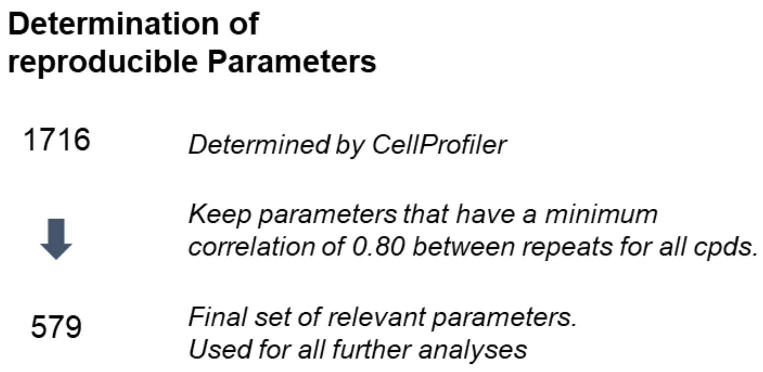

To determine the phenotypic profiles for each test compound Z-scores were then calculated for each parameter as how many times the Median Absolute Deviation (MAD) of the controls the measured parameter value of a test compound deviates from the Median of the controls:

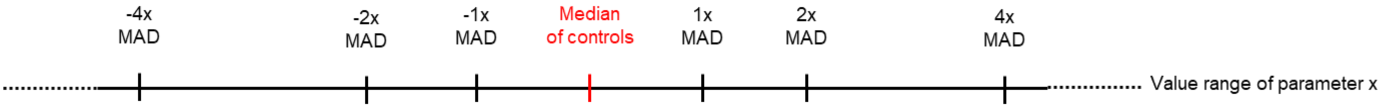

$$z\_score = \frac{value_{meas.} - Median_{Controls}}{MAD_{Controls}}$$

The phenotypic compound profile is then determined as the list of z-scores of all parameters for one compound.

In addition to the phenotypic profile, an induction value was determined for each compound as the fraction of significantly changed parameters, in percent:

$$Induction\ [\%] = \frac{number\ of\ parameters\ with\ abs.\ values > 3}{total\ number\ of\ parameters}$$

Similarities of phenotypic profiles were calculated from the correlation distances between two profiles (https://docs.scipy.org/doc/scipy/reference/generated/scipy.spatial.distance.correlation.html; Similarity = 1 - Correlation Distance) and the compounds with the most similar profiles were determined from a set of 3000 reference compounds that was also measured in the assay.

An example for two compounds with highly similar profiles (96% similarity):

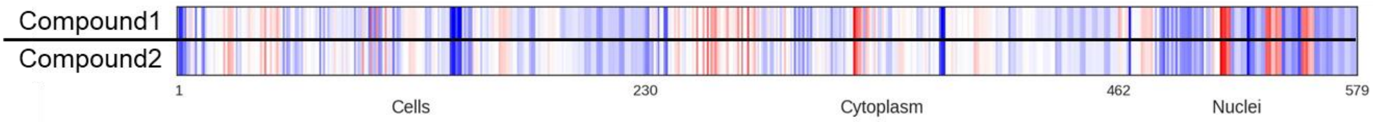

An example for two compounds with low similarity profiles (0% similarity):

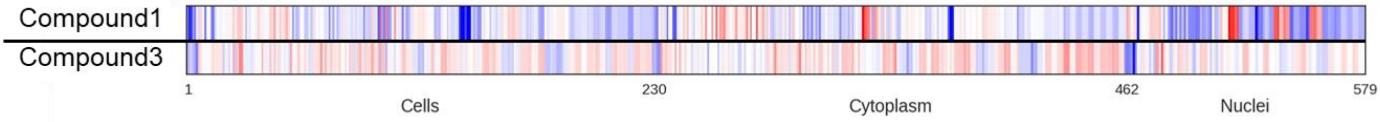

Each colored band represents one Z-score of a parameter.

5.3.1 Discussion of the Induction Parameter

The Cell Painting assay is under active development in the department since the beginning of 2017 and the induction was introduced into the analysis as a measure of the extent of the phenotypic effect. The assay is currently being developed in its third iteration.

The first iteration used a very sparse profile of only about hundred features with categorical values (0 – reduced, 1 unchanged, 2 – increased compared to control). The induction was introduced at this stage. The code for this first iteration was described[16] and open-sourced[17] in 2017.

The second iteration already used a much larger profile with continuous values but was based on single point measurements.

The current third iteration uses 579 features calculated from triplicate measurements with shifted layouts for reduced plate effects. It now uses induction as a measure of how many features of the profile have been significantly changed compared to DMSO controls (induction = 0%, calculation see above), making it a robust and easy to calculate parameter in our hands.

It should be noted that the induction is not a measure of activity per se, but rather of the extent of the observed phenotypic effect.

In our opinion, this is an important distinction to make, since the extent of the effect is not necessarily correlated to compound activity. It rather depends on the compound’s target(s) and the extent to which the modulation of these target(s) induce phenotypic effects. In other words, a compound can have a high biological activity on a target that does cause only minor phenotypic changes in the cell and will therefore only have a small induction value. For this case, equating the low induction with low activity would be misleading.

In case by case situations, where compounds have the same target(s) the induction might be used as a proxy parameter for activity and in this context we are using the it e.g. for finding concentrations at which the compounds are having a comparable extent of the phenotypic changes.

We also use the induction as a cutoff parameter to decide whether a comparison of a profile to other profiles would make sense. A profile with an induction < 5% we consider being too sparse for a meaningful comparison, whereas a profile with an induction >= 80% is usually too crowded for a good comparison. In the first case, we re-test the compounds in question at higher doses, in the latter case, we reduce the dose.

A more detailed comparison to other measures of the extent of phenotypic effects, like the Mahalanobis distance to DMSO controls will be part of a larger methodology paper to be written. At that time the code of the current iteration will also be made available.

5.3.2 Availability of the cell painting assay code

The code and data analysis are still undergoing method development. A separate Cell Painting methodology paper detailing the finalised code will follow this manuscript. We are willing to share the code and data in its current form upon request.

5.3.3 Further discussion and interpretation of the cell painting data

Cut-off for common reference compounds

In order for reference compounds to be considered biosimilar in the cell painting assay, we considered the following parameters:

- ≥75% biosimilarity in the cell painting profile.
- The following induction range: 5% < % < 80% (see also the discussion in Section 5.3.1).

Comparison of terbutaline and SAR405

a

	Biosimilarity	Chemical similarity
SAR405 / Terbutaline	83%	10%

b

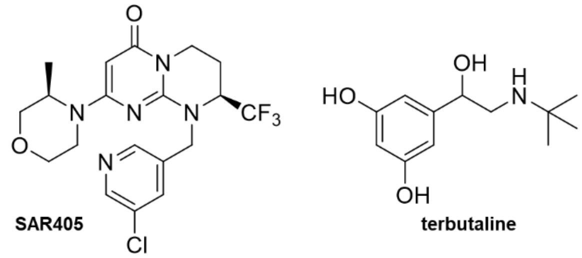

c

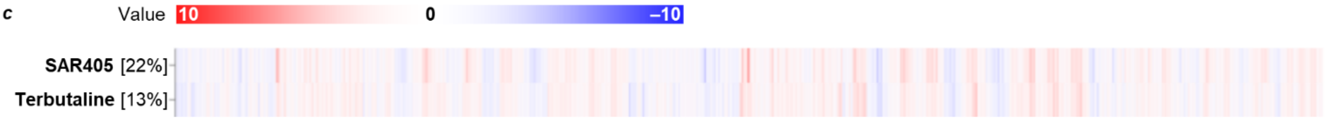

Figure S9 Comparison of Terbutaline versus SAR405 in the cell painting assay.

The profiles of terbutaline and SAR405 were found to be 83% biosimilar in the cell painting assay, however, terbutaline was inactive in our autophagy screen (see Section 5.2), whereas SAR405 was inhibited both starvation-induced autophagy (2.51 ± 2.3 μM) and rapamycin-induced autophagy (1.09 ± 0.8 μM). While terbutaline is inactive in autophagy, this does not rule out the possibility that it is a weak VPS34 inhibitor. We also note that similar phenotypes can be induced in the cell painting assay by interference with different biological processes.

5.3.4 Comparison of azaquindole-1 with other recently identified autophagy inhibitors

We compared the cell painting fingerprint of azaquindole-1 (**10w-j**) against other autophagy inhibitors that were recently identified in the Waldmann laboratory. The compounds in the study are summarised in Figure S10, and the profiles of the compounds that showed biosimilarity to azaquindole-1 are shown in Figure S11.

structure	name	mode of action	autophagy inhibition
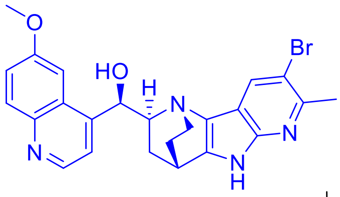	**azaquindole-1** (*from quinine*)	VPS34 inhibitor	SIA: 0.04 ± 0.02 µM<br>RIA:0.10 ± 0.02 µM<br>VPS34 IC_{50}: 0.35 µM
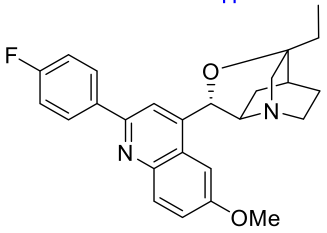	**oxautin-1** (*from quinidine*)	lysosomotropic and Fe^{2+} sequestration	SIA: 0.65 ± 0.22 µM<br>RIA: inactive
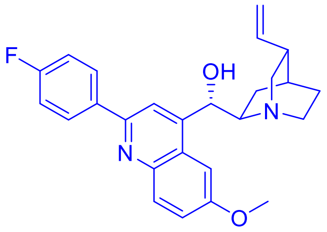	**autoquin** (*from quinidine*)	lysosomotropic and Fe^{2+} sequestration	SIA: 0.56 ± 0.15 µM<br>RIA: inactive
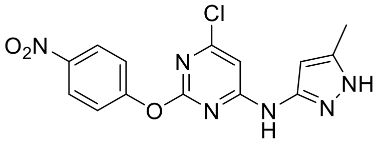	**autophinib**	VPS34 inhibitor	SIA: 0.09 ± 0.04 µM<br>RIA: 0.04 ± 0.01 µM<br>VPS34 IC_{50}: 0.019 µM
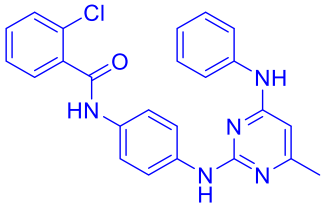	**aumitin**	inhibits mitochondrial complex I	SIA: 0.12 ± 0.07 µM<br>RIA: 0.24 ± 0.20 µM
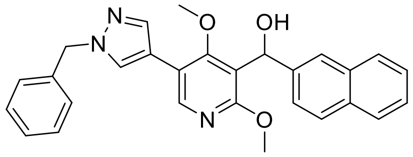	**DMP-1**	tbd	SIA: 1.3 ± 0.5 µM<br>RIA: inactive
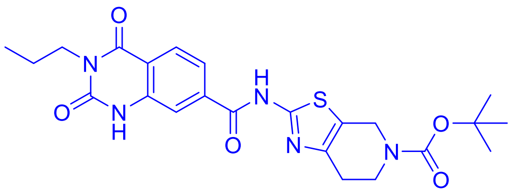	**autogramin-1**	GRAMD1A inhibitor	SIA: 0.17 ± 0.05 µM<br>RIA: 0.44 ± 0.30 µM

Figure S10 Autophagy inhibitors identified in the Waldmann group, including: azaquindole-1 (this work), oxautin-1[18], autoquin[19], autophinib,[20] aumitin,[2] DMP-1,[21] autogramin-1[22]. SIA= starvation-induced autophagy. RIA= rapamycin-induced autophagy.

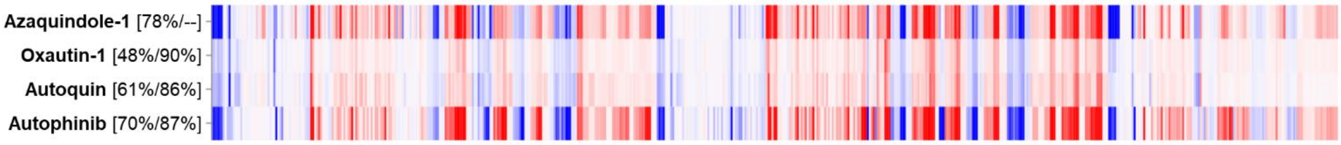

Figure S11 Comparison of the cell painting fingerprint of azaquindole-1 (**10w-j**) with the remaining compounds shown in Figure **S10**. In the square parentheses are detailed firstly the induction, and secondly (after the forward slash), the biosimilarity of the profile to azaquindole-1.

Our analysis revealed that the profile of azaquindole-1 (**10w-j**) is similar to the profiles of oxautin-1, autoquin and autophinib. Since these compounds emerged from our internal research programmes, they were not included in the original reference compound collection used to identify the target of azaquindole-1.

Notably, autophinib is an inhibitor of VPS34. The cell painting analysis therefore further validates our earlier finding that autophinib targets VPS34. This finding also highlights the ability of the cell painting assay data to suggest and subsequently identify molecular targets. However, oxautin-1 and autoquin do not inhibit VPS34. This suggests that in these cases the profile represents the bioactivity and mode of action in a broader sense. Thus, the cell painting assay may be a good experimental means to identify clusters of compounds with similar modes of action, but not necessarily the same targets. We intend to investigate these insights more thoroughly to determine whether such clusters exist in autophagy in a wider sense, and in other areas of biology.

5.4 β-Adrenergic receptor screening

Detection of the functional inhibition or activation of beta-1 and beta-2 adrenoreceptors was carried out by Eurofins CEREP SA (France). A functional, cell-based assay with Homogeneous Time Resolved Fluorescence (HTRF) read out was performed in agonist and antagonist mode at a concentration of 10 μM of azaquindole-1 (**10w-j**) or **10w-d**.

Information on how each assay was completed can be found using the following link (or otherwise contact the authors of this manuscript):

- https://www.eurofinsdiscoveryservices.com/catalogmanagement/viewitem/beta1-Human-Adrenoceptor-GPCR-Cell-Based-Agonist-cAMP-Assay-Cerep/1605
- https://www.eurofinsdiscoveryservices.com/catalogmanagement/viewitem/beta1-Human-Adrenoceptor-GPCR-Cell-Based-Antagonist-cAMP-Assay-Cerep/1606
- https://www.eurofinsdiscoveryservices.com/catalogmanagement/viewitem/beta2-Human-Adrenoceptor-GPCR-Cell-Based-Agonist-cAMP-Assay-Cerep/1976
- https://www.eurofinsdiscoveryservices.com/catalogmanagement/viewitem/beta2-Human-Adrenoceptor-GPCR-Cell-Based-Antagonist-cAMP-Assay-Cerep/1977

(all last accessed 12/16/2019)

Table S17 *In vitro* inhibition of β Adrenergic receptors by azaquindoles. Compounds screened at 10 μM in all assays.

Assay	Compound	Measurement	Value /%
beta 1 (h) (agonist effect)	**10w-j**	Mean %Effect	-7.43E-01
beta 1 (h) (agonist effect)	**10w-d**	Mean %Effect	-1.20E+00
beta 1 (h) (antagonist effect)	**10w-j**	Mean %Inhibition	8.60183
beta 1 (h) (antagonist effect)	**10w-d**	Mean %Inhibition	-1.42E+01
beta 2 (h) (agonist effect)	**10w-j**	Mean %Effect	2.83625
beta 2 (h) (agonist effect)	**10w-d**	Mean %Effect	3.5587
beta 2 (h) (antagonist effect)	**10w-j**	Mean %Inhibition	22.0743
beta 2 (h) (antagonist effect)	**10w-d**	Mean %Inhibition	0.10949

5.5 Kinase panel data

Detection of the biochemical inhibition of 485 kinases was carried out by Life Technologies Ltd (United Kingdom). The screen was performed in three different assays formats: Adapta (activity-based), Z-Lyte (activity-based) and Lantha (binding-based) at a concentration of 10 μM of azaquindole-1 (**10w-j**).

Information on how each assay was completed can be found using the following link (or otherwise contact the authors of this manuscript): https://www.thermofisher.com/nz/en/home/products-and-services/services/custom-services/screening-and-profiling-services/selectscreen-profiling-service/selectscreen-kinase-profiling-service.html (last accessed 07/06/2019).

For kinases showing >40% inhibition, an IC_{50} was determined.

Table S18 *In vitro* inhibition of kinases by azaquindole-1.

Entry	Kinase	Technology	ATP	% Inhibition 1	% Inhibition 2	% Inhibition mean	IC50 / nM
1	AAK1	Lantha	n/a	3	6	4	
2	ABL1	ZLYTE	Km app	2	2	2	
3	ABL1 E255K	ZLYTE	Km app	7	8	8	
4	ABL1 F317I	ZLYTE	Km app	1	1	1	
5	ABL1 F317L	ZLYTE	Km app	7	0	3	
6	ABL1 G250E	ZLYTE	Km app	2	1	2	
7	ABL1 H396P	Lantha	n/a	-7	2	-3	
8	ABL1 M351T	Lantha	n/a	9	16	13	
9	ABL1 Q252H	Lantha	n/a	-1	-20	-11	
10	ABL1 T315I	ZLYTE	Km app	5	3	4	
11	ABL1 Y253F	ZLYTE	Km app	2	1	2	
12	ABL2 (Arg)	ZLYTE	Km app	2	2	2	
13	ACVR1 (ALK2)	Lantha	n/a	3	5	4	
14	ACVR1 (ALK2) R206H	Lantha	n/a	38	44	41	>10000
15	ACVR1B (ALK4)	ZLYTE	Km app	-8	-3	-5	
16	ACVR2A	Lantha	n/a	-33	-8	-20	
17	ACVR2B	Lantha	n/a	-7	6	0	
18	ACVRL1 (ALK1)	Lantha	n/a	-3	-13	-8	
19	ADCK3	Lantha	n/a	33	34	33	
20	ADRBK1 (GRK2)	ZLYTE	Km app	1	-7	-3	
21	ADRBK2 (GRK3)	ZLYTE	Km app	8	7	7	
22	AKT1 (PKB alpha)	ZLYTE	Km app	55	60	58	>10000
23	AKT2 (PKB beta)	ZLYTE	Km app	13	17	15	
24	AKT3 (PKB gamma)	ZLYTE	Km app	12	9	11	
25	ALK	ZLYTE	Km app	10	-3	4	
26	ALK C1156Y	Lantha	n/a	-3	1	-1	
27	ALK F1174L	Lantha	n/a	-13	-1	-7	
28	ALK L1196M	Lantha	n/a	-1	-18	-9	
29	ALK R1275Q	Lantha	n/a	10	10	10	
30	ALK T1151_L1152insT	Lantha	n/a	-13	-10	-12	

Entry	Kinase	Technology	ATP	% Inhibition 1	2	% Inhibition mean	IC50 / nM
31	AMPK (A1/B1/G2)	Lantha	n/a	4	-2	1	
32	AMPK (A1/B1/G3)	Lantha	n/a	0	-4	-2	
33	AMPK (A1/B2/G1)	Lantha	n/a	-3	3	0	
34	AMPK (A1/B2/G2)	ZLYTE	Km app	-1	3	1	
35	AMPK (A1/B2/G3)	ZLYTE	Km app	-2	6	2	
36	AMPK (A2/B1/G2)	ZLYTE	Km app	6	3	5	
37	AMPK (A2/B1/G3)	ZLYTE	Km app	8	12	10	
38	AMPK (A2/B2/G1)	Lantha	n/a	-2	-4	-3	
39	AMPK (A2/B2/G2)	Lantha	n/a	1	2	1	
40	AMPK (A2/B2/G3)	ZLYTE	Km app	8	8	8	
41	AMPK A1/B1/G1	ZLYTE	Km app	22	24	23	
42	AMPK A2/B1/G1	ZLYTE	Km app	14	15	15	
43	ANKK1	Lantha	n/a	5	11	8	
44	AURKA (Aurora A)	ZLYTE	Km app	3	5	4	
45	AURKB (Aurora B)	ZLYTE	Km app	3	6	4	
46	AURKC (Aurora C)	ZLYTE	Km app	8	5	6	
47	AXL	ZLYTE	Km app	3	3	3	
48	AXL R499C	Lantha	n/a	-29	-34	-31	
49	BLK	ZLYTE	Km app	0	4	2	
50	BMPR1A (ALK3)	Lantha	n/a	16	17	16	
51	BMPR1B (ALK6)	Lantha	n/a	1	-3	-1	
52	BMPR2	Lantha	n/a	0	3	2	
53	BMX	ZLYTE	Km app	-7	-6	-7	
54	BRAF	ZLYTE	100	27	19	23	
55	BRAF	Lantha	n/a	-5	-2	-3	
56	BRAF V599E	ZLYTE	100	-2	-9	-5	
57	BRAF V599E	Lantha	n/a	4	6	5	
58	BRSK1 (SAD1)	ZLYTE	Km app	22	23	23	
59	BRSK2	Lantha	n/a	-14	-36	-25	
60	BTK	ZLYTE	Km app	6	6	6	
61	CAMK1 (CaMK1)	Adapta	10	-11	-9	-10	
62	CAMK1D (CaMKI delta)	ZLYTE	Km app	6	6	6	
63	CAMK1G (CAMKI gamma)	ZLYTE	Km app	0	0	0	
64	CAMK2A (CaMKII alpha)	ZLYTE	Km app	4	4	4	
65	CAMK2B (CaMKII beta)	ZLYTE	Km app	-5	-4	-5	
66	CAMK2D (CaMKII delta)	ZLYTE	Km app	7	9	8	
67	CAMK2G (CaMKII gamma)	Lantha	n/a	-5	-11	-8	
68	CAMK4 (CaMKIV)	ZLYTE	Km app	4	6	5	
69	CAMKK1 (CAMKKA)	Lantha	n/a	-8	-8	-8	
70	CAMKK2 (CaMKK beta)	Lantha	n/a	15	17	16	
71	CASK	Lantha	n/a	-5	-2	-4	
72	CDC42 BPA (MRCKA)	ZLYTE	Km app	4	4	4	
73	CDC42 BPB (MRCKB)	ZLYTE	Km app	2	1	1	
74	CDC42 BPG (MRCKG)	ZLYTE	Km app	-2	3	1	
75	CDC7/DBF4	Lantha	n/a	7	3	5	
76	CDK1/cyclin B	ZLYTE	Km app	1	-1	0	
77	CDK11 (Inactive)	Lantha	n/a	14	8	11	
78	CDK11/cyclin C	Lantha	n/a	4	-7	-2	
79	CDK13/cyclin K	Lantha	n/a	-2	8	3	
80	CDK14 (PFTK1)/cyclin Y	Lantha	n/a	-11	-23	-17	
81	CDK16 (PCTK1)/cyclin Y	Lantha	n/a	-26	-5	-15	
82	CDK17/cyclin Y	ZLYTE	Km app	1	3	2	

Entry	Kinase	Technology	ATP	% Inhibition 1	2	% Inhibition mean	IC50 / nM
83	CDK18/cyclin Y	ZLYTE	Km app	-11	8	-1	
84	CDK2/cyclin A	ZLYTE	Km app	0	5	2	
85	CDK2/cyclin A1	Lantha	n/a	0	1	1	
86	CDK2/cyclin E1	Lantha	n/a	1	-1	0	
87	CDK2/cyclin O	Lantha	n/a	-1	3	1	
88	CDK3/cyclin E1	Lantha	n/a	-9	-8	-9	
89	CDK4/cyclin D1	Adapta	10	-13	-12	-13	
90	CDK4/cyclin D3	Adapta	10	11	8	10	
91	CDK5 (Inactive)	Lantha	n/a	-7	-11	-9	
92	CDK5/p25	ZLYTE	Km app	-4	7	1	
93	CDK5/p35	ZLYTE	Km app	-1	6	3	
94	CDK6/cyclin D1	Adapta	10	-14	-20	-17	
95	CDK7/cyclin H/MNAT1	Adapta	Km app	12	6	9	
96	CDK8/cyclin C	Lantha	n/a	14	15	14	
97	CDK9 (Inactive)	Lantha	n/a	-7	-15	-11	
98	CDK9/cyclin K	Lantha	n/a	-3	6	2	
99	CDK9/cyclin T1	Adapta	Km app	20	21	20	
100	CDKL5	ZLYTE	Km app	5	11	8	
101	CHEK1 (CHK1)	ZLYTE	Km app	-1	-8	-4	
102	CHEK2 (CHK2)	ZLYTE	Km app	21	11	16	
103	CHUK (IKK alpha)	Adapta	Km app	9	6	7	
104	CLK1	ZLYTE	Km app	15	12	13	
105	CLK2	ZLYTE	Km app	51	44	47	572
106	CLK3	ZLYTE	Km app	2	4	3	
107	CLK4	Lantha	n/a	78	83	81	93.9
108	CSF1R (FMS)	ZLYTE	Km app	12	7	10	
109	CSK	ZLYTE	Km app	-8	2	-3	
110	CSNK1A1 (CK1 alpha 1)	ZLYTE	Km app	-9	-5	-7	
111	CSNK1A1L	ZLYTE	Km app	1	3	2	
112	CSNK1D (CK1 delta)	ZLYTE	Km app	9	5	7	
113	CSNK1E (CK1 epsilon)	ZLYTE	Km app	7	3	5	
114	CSNK1E (CK1 epsilon) R178C	ZLYTE	Km app	4	4	4	
115	CSNK1G1 (CK1 gamma 1)	ZLYTE	Km app	-3	7	2	
116	CSNK1G2 (CK1 gamma 2)	ZLYTE	Km app	10	11	11	
117	CSNK1G3 (CK1 gamma 3)	ZLYTE	Km app	25	5	15	
118	CSNK2A1 (CK2 alpha 1)	ZLYTE	Km app	8	4	6	
119	CSNK2A2 (CK2 alpha 2)	ZLYTE	Km app	9	1	5	
120	DAPK1	Adapta	Km app	12	7	9	
121	DAPK2	Lantha	n/a	-34	-21	-27	
122	DAPK3 (ZIPK)	ZLYTE	Km app	1	-1	0	
123	DCAMKL1 (DCLK1)	ZLYTE	Km app	6	6	6	
124	DCAMKL2 (DCK2)	ZLYTE	Km app	6	3	4	
125	DDR1	Lantha	n/a	-3	0	-1	
126	DDR2	Lantha	n/a	10	20	15	
127	DDR2 N456S	Lantha	n/a	-32	-9	-20	
128	DDR2 T654M	Lantha	n/a	14	16	15	
129	DMPK	Lantha	n/a	5	5	5	
130	DNA-PK	ZLYTE	Km app	1	9	5	
131	DYRK1A	ZLYTE	Km app	24	23	24	
132	DYRK1B	ZLYTE	Km app	11	16	13	
133	DYRK2	Lantha	n/a	1	3	2	
134	DYRK3	ZLYTE	Km app	-9	-10	-10	

Entry	Kinase	Technology	ATP	% Inhibition 1	2	% Inhibition mean	IC50 / nM
135	DYRK4	ZLYTE	Km app	5	3	4	
136	EEF2K	ZLYTE	Km app	1	2	2	
137	EGFR (ErbB1)	ZLYTE	Km app	-11	4	-4	
138	EGFR (ErbB1) C797S	ZLYTE	Km app	1	-1	0	
139	EGFR (ErbB1) d746-750	Lantha	n/a	-11	-2	-6	
140	EGFR (ErbB1) d747-749 A750P	Lantha	n/a	-7	-14	-11	
141	EGFR (ErbB1) G719C	ZLYTE	Km app	-3	-5	-4	
142	EGFR (ErbB1) G719S	ZLYTE	Km app	-3	0	-2	
143	EGFR (ErbB1) L858R	ZLYTE	Km app	-16	4	-6	
144	EGFR (ErbB1) L861Q	ZLYTE	Km app	4	0	2	
145	EGFR (ErbB1) T790M	ZLYTE	Km app	3	1	2	
146	EGFR (ErbB1) T790M C797S L858R	ZLYTE	Km app	-2	-1	-2	
147	EGFR (ErbB1) T790M L858R	ZLYTE	Km app	-1	1	0	
148	EIF2AK2 (PKR)	Lantha	n/a	-9	3	-3	
149	EPHA1	ZLYTE	Km app	7	-1	3	
150	EPHA2	ZLYTE	Km app	-6	-6	-6	
151	EPHA3	Lantha	n/a	-5	-1	-3	
152	EPHA4	ZLYTE	Km app	10	14	12	
153	EPHA5	ZLYTE	Km app	-1	2	0	
154	EPHA6	Lantha	n/a	-2	10	4	
155	EPHA7	Lantha	n/a	-2	12	5	
156	EPHA8	ZLYTE	Km app	3	5	4	
157	EPHB1	ZLYTE	Km app	-1	5	2	
158	EPHB2	ZLYTE	Km app	-1	-4	-3	
159	EPHB3	ZLYTE	Km app	10	8	9	
160	EPHB4	ZLYTE	Km app	0	-3	-1	
161	ERBB2 (HER2)	ZLYTE	Km app	3	1	2	
162	ERBB4 (HER4)	ZLYTE	Km app	-8	-4	-6	
163	ERN1	Lantha	n/a	-5	-20	-13	
164	ERN2	Lantha	n/a	4	3	4	
165	FER	ZLYTE	Km app	6	9	8	
166	FES (FPS)	ZLYTE	Km app	8	10	9	
167	FGFR1	ZLYTE	Km app	-3	-4	-4	
168	FGFR1 V561M	Lantha	n/a	10	7	9	
169	FGFR2	ZLYTE	Km app	-10	-11	-11	
170	FGFR2 N549H	ZLYTE	Km app	1	-4	-2	
171	FGFR3	ZLYTE	Km app	-2	-4	-3	
172	FGFR3 G697C	Lantha	n/a	-22	-21	-21	
173	FGFR3 K650E	ZLYTE	Km app	-6	-4	-5	
174	FGFR3 K650M	Lantha	n/a	6	4	5	
175	FGFR3 V555M	ZLYTE	Km app	-11	8	-1	
176	FGFR4	ZLYTE	Km app	9	3	6	
177	FGR	ZLYTE	Km app	15	15	15	
178	FLT1 (VEGFR1)	ZLYTE	Km app	-3	2	0	
179	FLT3	ZLYTE	Km app	12	13	13	
180	FLT3 D835Y	ZLYTE	Km app	5	5	5	
181	FLT3 ITD	Lantha	n/a	-52	-59	-55	
182	FLT4 (VEGFR3)	ZLYTE	Km app	-6	1	-2	
183	FRAP1 (mTOR)	ZLYTE	Km app	11	5	8	
184	FRK (PTK5)	ZLYTE	Km app	5	3	4	
185	FYN	ZLYTE	Km app	3	0	2	
186	FYN A	Lantha	n/a	2	9	6	

Entry	Kinase	Technology	ATP	% Inhibition 1	2	% Inhibition mean	IC50 / nM
187	GAK	Lantha	n/a	-3	16	6	
188	GRK1	Lantha	n/a	7	-4	2	
189	GRK4	ZLYTE	Km app	9	8	9	
190	GRK5	ZLYTE	Km app	3	-4	0	
191	GRK6	ZLYTE	Km app	2	7	5	
192	GRK7	ZLYTE	Km app	3	-5	-1	
193	GSG2 (Haspin)	Adapta	Km app	23	24	24	
194	GSK3A (GSK3 alpha)	ZLYTE	Km app	5	3	4	
195	GSK3B (GSK3 beta)	ZLYTE	Km app	12	11	12	
196	HCK	ZLYTE	Km app	1	9	5	
197	HIPK1 (Myak)	ZLYTE	Km app	0	0	0	
198	HIPK2	ZLYTE	Km app	3	3	3	
199	HIPK3 (YAK1)	ZLYTE	Km app	1	3	2	
200	HIPK4	ZLYTE	Km app	-5	-9	-7	
201	HUNK	Lantha	n/a	-10	1	-4	
202	ICK	Lantha	n/a	-2	16	7	
203	IGF1R	ZLYTE	Km app	-7	-7	-7	
204	IKBKB (IKK beta)	ZLYTE	Km app	-1	0	0	
205	IKBKE (IKK epsilon)	ZLYTE	Km app	8	15	12	
206	INSR	ZLYTE	Km app	4	4	4	
207	INSRR (IRR)	ZLYTE	Km app	4	10	7	
208	IRAK1	Adapta	Km app	16	19	17	
209	IRAK3	Lantha	n/a	-10	-3	-7	
210	IRAK4	ZLYTE	Km app	4	5	4	
211	ITK	ZLYTE	Km app	6	2	4	
212	JAK1	ZLYTE	Km app	-7	-6	-6	
213	JAK2	ZLYTE	Km app	10	1	5	
214	JAK2 JH1 JH2	ZLYTE	Km app	-15	-19	-17	
215	JAK2 JH1 JH2 V617F	ZLYTE	Km app	1	-5	-2	
216	JAK3	ZLYTE	Km app	3	-1	1	
217	KDR (VEGFR2)	ZLYTE	Km app	9	11	10	
218	KIT	ZLYTE	Km app	7	13	10	
219	KIT A829P	Lantha	n/a	-8	-11	-10	
220	KIT D816H	Lantha	n/a	-8	4	-2	
221	KIT D816V	Lantha	n/a	-7	-6	-6	
222	KIT D820E	Lantha	n/a	8	9	9	
223	KIT N822K	Lantha	n/a	-1	0	-1	
224	KIT T670E	Lantha	n/a	-5	-4	-4	
225	KIT T670I	ZLYTE	Km app	-4	-14	-9	
226	KIT V559D	ZLYTE	Km app	-9	-2	-6	
227	KIT V559D T670I	Lantha	n/a	3	-12	-5	
228	KIT V559D V654A	ZLYTE	Km app	12	-5	4	
229	KIT V560G	ZLYTE	Km app	-6	-6	-6	
230	KIT V654A	Lantha	n/a	-1	0	0	
231	KIT Y823D	Lantha	n/a	-4	-6	-5	
232	KSR2	ZLYTE	Km app	8	11	9	
233	LATS2	Lantha	n/a	-5	3	-1	
234	LCK	ZLYTE	Km app	-16	-3	-10	
235	LIMK1	Lantha	n/a	4	0	2	
236	LIMK2	Lantha	n/a	-3	4	1	
237	LRRK2	Adapta	Km app	-8	-4	-6	
238	LRRK2 FL	Adapta	Km app	-1	-5	-3	

Entry	Kinase	Technology	ATP	% Inhibition 1	% Inhibition 2	% Inhibition mean	IC50 / nM
239	LRRK2 G2019S	Adapta	Km app	10	14	12	
240	LRRK2 G2019S FL	Adapta	Km app	6	-3	2	
241	LRRK2 I2020T	Adapta	Km app	1	1	1	
242	LRRK2 R1441C	Adapta	Km app	1	2	2	
243	LTK (TYK1)	ZLYTE	Km app	-1	2	0	
244	LYN A	ZLYTE	Km app	10	13	11	
245	LYN B	ZLYTE	Km app	3	2	2	
246	MAP2K1 (MEK1)	ZLYTE	100	1	5	3	
247	MAP2K1 (MEK1)	Lantha	n/a	-2	-7	-5	
248	MAP2K1 (MEK1) S218D S222D	Lantha	n/a	8	2	5	
249	MAP2K2 (MEK2)	ZLYTE	100	7	9	8	
250	MAP2K2 (MEK2)	Lantha	n/a	-3	2	-1	
251	MAP2K4 (MEK4)	Lantha	n/a	9	18	13	
252	MAP2K5 (MEK5)	Lantha	n/a	5	4	5	
253	MAP2K6 (MKK6)	ZLYTE	100	3	6	5	
254	MAP2K6 (MKK6)	Lantha	n/a	12	15	13	
255	MAP2K6 (MKK6) S207E T211E	Lantha	n/a	14	15	14	
256	MAP3K10 (MLK2)	Lantha	n/a	8	4	6	
257	MAP3K11 (MLK3)	Lantha	n/a	-2	3	0	
258	MAP3K14 (NIK)	Lantha	n/a	8	-2	3	
259	MAP3K19 (YSK4)	ZLYTE	Km app	-7	-3	-5	
260	MAP3K2 (MEKK2)	Lantha	n/a	1	10	6	
261	MAP3K3 (MEKK3)	Lantha	n/a	-11	-13	-12	
262	MAP3K5 (ASK1)	Lantha	n/a	4	0	2	
263	MAP3K7/MAP3K7IP1 (TAK1-TAB1)	Lantha	n/a	11	7	9	
264	MAP3K8 (COT)	ZLYTE	100	6	4	5	
265	MAP3K9 (MLK1)	ZLYTE	Km app	21	21	21	
266	MAP4K1 (HPK1)	Lantha	n/a	16	16	16	
267	MAP4K2 (GCK)	ZLYTE	Km app	3	18	10	
268	MAP4K3 (GLK)	Lantha	n/a	-1	-2	-1	
269	MAP4K4 (HGK)	ZLYTE	Km app	9	8	8	
270	MAP4K5 (KHS1)	ZLYTE	Km app	15	18	16	
271	MAPK1 (ERK2)	ZLYTE	Km app	-6	-18	-12	
272	MAPK10 (JNK3)	ZLYTE	100	1	0	1	
273	MAPK10 (JNK3)	Lantha	n/a	3	7	5	
274	MAPK11 (p38 beta)	ZLYTE	Km app	-4	-5	-5	
275	MAPK12 (p38 gamma)	ZLYTE	Km app	8	14	11	
276	MAPK13 (p38 delta)	ZLYTE	Km app	8	9	8	
277	MAPK14 (p38 alpha)	ZLYTE	100	13	11	12	
278	MAPK14 (p38 alpha) Direct	ZLYTE	Km app	-16	-20	-18	
279	MAPK15 (ERK7)	Lantha	n/a	7	2	4	
280	MAPK3 (ERK1)	ZLYTE	Km app	0	0	0	
281	MAPK7 (ERK5)	ZLYTE	Km app	10	11	10	
282	MAPK8 (JNK1)	ZLYTE	100	-8	-9	-8	
283	MAPK8 (JNK1)	Lantha	n/a	6	4	5	
284	MAPK9 (JNK2)	ZLYTE	100	6	11	9	
285	MAPK9 (JNK2)	Lantha	n/a	11	13	12	
286	MAPKAPK2	ZLYTE	Km app	8	-8	0	
287	MAPKAPK3	ZLYTE	Km app	19	12	15	
288	MAPKAPK5 (PRAK)	ZLYTE	Km app	1	7	4	
289	MARK1 (MARK)	ZLYTE	Km app	3	6	5	
290	MARK2	ZLYTE	Km app	-6	-5	-5	

Entry	Kinase	Technology	ATP	% Inhibition 1	% Inhibition 2	% Inhibition mean	IC50 / nM
291	MARK3	ZLYTE	Km app	19	17	18	
292	MARK4	ZLYTE	Km app	16	12	14	
293	MASTL	Lantha	n/a	-22	-1	-11	
294	MATK (HYL)	ZLYTE	Km app	1	-6	-3	
295	MELK	ZLYTE	Km app	22	24	23	
296	MERTK (cMER)	ZLYTE	Km app	1	1	1	
297	MERTK (cMER) A708S	Lantha	n/a	-3	-5	-4	
298	MET (cMet)	ZLYTE	Km app	6	7	6	
299	MET (cMet) Y1235D	ZLYTE	Km app	-5	3	-1	
300	MET D1228H	Lantha	n/a	-10	-1	-6	
301	MET M1250T	ZLYTE	Km app	-3	0	-2	
302	MINK1	ZLYTE	Km app	-6	-1	-4	
303	MKNK1 (MNK1)	ZLYTE	Km app	4	-4	0	
304	MKNK2 (MNK2)	Lantha	n/a	-13	11	-1	
305	MLCK (MLCK2)	Lantha	n/a	8	14	11	
306	MLK4	Lantha	n/a	-38	-16	-27	
307	MST1R (RON)	ZLYTE	Km app	4	-2	1	
308	MST4	ZLYTE	Km app	5	-10	-2	
309	MUSK	ZLYTE	Km app	1	-2	-1	
310	MYLK (MLCK)	Lantha	n/a	-1	2	0	
311	MYLK2 (skMLCK)	ZLYTE	Km app	3	1	2	
312	MYLK4	Lantha	n/a	9	3	6	
313	MYO3A (MYO3 alpha)	Lantha	n/a	-6	-6	-6	
314	MYO3B (MYO3 beta)	Lantha	n/a	0	-4	-2	
315	NEK1	ZLYTE	Km app	4	-6	-1	
316	NEK2	ZLYTE	Km app	-6	2	-2	
317	NEK4	ZLYTE	Km app	3	5	4	
318	NEK6	ZLYTE	Km app	16	6	11	
319	NEK8	Lantha	n/a	-18	-17	-18	
320	NEK9	ZLYTE	Km app	-2	0	-1	
321	NIM1K	ZLYTE	Km app	2	2	2	
322	NLK	Lantha	n/a	8	12	10	
323	NTRK1 (TRKA)	ZLYTE	Km app	-33	-10	-21	
324	NTRK2 (TRKB)	ZLYTE	Km app	-1	-3	-2	
325	NTRK3 (TRKC)	ZLYTE	Km app	2	-6	-2	
326	NUAK1 (ARK5)	Adapta	Km app	10	5	8	
327	NUAK2	Lantha	n/a	10	17	14	
328	PAK1	ZLYTE	Km app	-1	3	1	
329	PAK2 (PAK65)	ZLYTE	Km app	2	6	4	
330	PAK3	ZLYTE	Km app	17	12	14	
331	PAK4	ZLYTE	Km app	-8	-9	-8	
332	PAK6	ZLYTE	Km app	-13	3	-5	
333	PAK7 (KIAA1264)	ZLYTE	Km app	-14	7	-4	
334	PASK	ZLYTE	Km app	8	17	12	
335	PDGFRA (PDGFR alpha)	ZLYTE	Km app	-2	4	1	
336	PDGFRA D842V	ZLYTE	Km app	-1	-8	-4	
337	PDGFRA T674I	ZLYTE	Km app	-26	-38	-32	
338	PDGFRA V561D	ZLYTE	Km app	0	0	0	
339	PDGFRB (PDGFR beta)	ZLYTE	Km app	-12	-3	-8	
340	PDK1	ZLYTE	100	-1	3	1	
341	PDK1 Direct	ZLYTE	Km app	-4	6	1	
342	PEAK1	ZLYTE	Km app	-4	-3	-3	

Entry	Kinase	Technology	ATP	% Inhibition 1	2	% Inhibition mean	IC50 / nM
343	PHKG1	ZLYTE	Km app	12	7	10	
344	PHKG2	ZLYTE	Km app	4	8	6	
345	PI4K2A (PI4K2 alpha)	Adapta	Km app	-3	-3	-3	
346	PI4K2B (PI4K2 beta)	Adapta	Km app	-8	-3	-6	
347	PI4KA (PI4K alpha)	Adapta	10	11	14	12	
348	PI4KB (PI4K beta)	Adapta	Km app	78	78	78	5160
349	PIK3C2A (PI3K-C2 alpha)	Adapta	Km app	11	16	13	
350	PIK3C2B (PI3K-C2 beta)	Adapta	10	77	76	76	2160
351	PIK3C2G (PI3K-C2 gamma)	Adapta	Km app	86	86	86	497
352	PIK3C3 (hVPS34)	Adapta	Km app	97	97	97	350
353	PIK3CA E542K/PIK3R1 (p110 alpha E542K/p85 alpha)	Adapta	10	26	24	25	
354	PIK3CA E545K/PIK3R1 (p110 alpha E545K/p85 alpha)	Adapta	Km app	0	2	1	
355	PIK3CA/PIK3R1 (p110 alpha/p85 alpha)	Adapta	Km app	12	14	13	
356	PIK3CA/PIK3R3 (p110 alpha/p55 gamma)	Adapta	Km app	10	12	11	
357	PIK3CB/PIK3R1 (p110 beta/p85 alpha)	Adapta	Km app	0	-3	-1	
358	PIK3CB/PIK3R2 (p110 beta/p85 beta)	Adapta	Km app	-11	-8	-9	
359	PIK3CD/PIK3R1 (p110 delta/p85 alpha)	Adapta	Km app	78	79	79	4790
360	PIK3CG (p110 gamma)	Adapta	Km app	-4	-10	-7	
361	PIM1	ZLYTE	Km app	1	3	2	
362	PIM2	ZLYTE	Km app	5	4	5	
363	PIM3	ZLYTE	Km app	5	-5	0	
364	PIP4K2A	Adapta	10	7	-2	2	
365	PIP5K1A	Adapta	10	3	-4	0	
366	PIP5K1B	Adapta	10	4	0	2	
367	PIP5K1C	Adapta	10	-23	-21	-22	
368	PKMYT1	Lantha	n/a	6	10	8	
369	PKN1 (PRK1)	ZLYTE	Km app	5	0	2	
370	PKN2 (PRK2)	Lantha	n/a	-6	-4	-5	
371	PLK1	ZLYTE	Km app	7	6	7	
372	PLK2	ZLYTE	Km app	8	5	6	
373	PLK3	ZLYTE	Km app	19	5	12	
374	PLK4	Lantha	n/a	14	11	13	
375	PRKACA (PKA)	ZLYTE	Km app	6	8	7	
376	PRKACB (PRKAC beta)	Lantha	n/a	11	5	8	
377	PRKACG (PRKAC gamma)	Lantha	n/a	16	12	14	
378	PRKCA (PKC alpha)	ZLYTE	Km app	11	10	11	
379	PRKCB1 (PKC beta I)	ZLYTE	Km app	0	1	1	
380	PRKCB2 (PKC beta II)	ZLYTE	Km app	3	13	8	
381	PRKCD (PKC delta)	ZLYTE	Km app	-9	-3	-6	
382	PRKCE (PKC epsilon)	ZLYTE	Km app	-6	10	2	
383	PRKCG (PKC gamma)	ZLYTE	Km app	6	23	15	
384	PRKCH (PKC eta)	ZLYTE	Km app	1	14	7	
385	PRKCI (PKC iota)	ZLYTE	Km app	5	0	2	
386	PRKCN (PKD3)	ZLYTE	Km app	3	10	7	
387	PRKCQ (PKC theta)	ZLYTE	Km app	4	-6	-1	

Entry	Kinase	Technology	ATP	% Inhibition 1	2	% Inhibition mean	IC50 / nM
388	PRKCZ (PKC zeta)	ZLYTE	Km app	3	3	3	
389	PRKD1 (PKC mu)	ZLYTE	Km app	4	1	2	
390	PRKD2 (PKD2)	ZLYTE	Km app	2	1	2	
391	PRKG1	ZLYTE	Km app	7	11	9	
392	PRKG2 (PKG2)	ZLYTE	Km app	12	14	13	
393	PRKX	ZLYTE	Km app	5	14	10	
394	PTK2 (FAK)	ZLYTE	Km app	-2	-2	-2	
395	PTK2B (FAK2)	ZLYTE	Km app	-1	1	0	
396	PTK6 (Brk)	ZLYTE	Km app	-2	-2	-2	
397	RAF1 (cRAF) Y340D Y341D	ZLYTE	100	19	20	20	
398	RAF1 (cRAF) Y340D Y341D	Lantha	n/a	-3	-2	-3	
399	RET	ZLYTE	Km app	-2	-1	-2	
400	RET A883F	ZLYTE	Km app	-6	-4	-5	
401	RET G691S	Lantha	n/a	-17	-24	-20	
402	RET M918T	Lantha	n/a	-6	-25	-15	
403	RET S891A	ZLYTE	Km app	-4	-6	-5	
404	RET V804E	ZLYTE	Km app	0	-5	-3	
405	RET V804L	ZLYTE	Km app	1	-1	0	
406	RET V804M	Lantha	n/a	-15	-18	-16	
407	RET Y791F	ZLYTE	Km app	6	7	7	
408	RIPK2	Lantha	n/a	7	9	8	
409	RIPK3	Lantha	n/a	-48	-48	-48	
410	ROCK1	ZLYTE	Km app	1	-4	-1	
411	ROCK2	ZLYTE	Km app	10	8	9	
412	ROS1	ZLYTE	Km app	5	0	2	
413	RPS6KA1 (RSK1)	ZLYTE	Km app	14	6	10	
414	RPS6KA2 (RSK3)	ZLYTE	Km app	-8	9	0	
415	RPS6KA3 (RSK2)	ZLYTE	Km app	13	12	13	
416	RPS6KA4 (MSK2)	ZLYTE	Km app	4	6	5	
417	RPS6KA5 (MSK1)	ZLYTE	Km app	3	15	9	
418	RPS6KA6 (RSK4)	ZLYTE	Km app	5	16	11	
419	RPS6KB1 (p70S6K)	ZLYTE	Km app	-1	-1	-1	
420	RPS6KB2 (p70S6Kb)	ZLYTE	Km app	-2	0	-1	
421	SBK1	ZLYTE	Km app	3	4	4	
422	SGK (SGK1)	ZLYTE	Km app	10	22	16	
423	SGK2	ZLYTE	Km app	14	10	12	
424	SGKL (SGK3)	ZLYTE	Km app	0	6	3	
425	SIK1	Lantha	n/a	-4	1	-2	
426	SIK3	Lantha	n/a	7	-4	2	
427	SLK	Lantha	n/a	-9	-7	-8	
428	SNF1LK2	ZLYTE	Km app	4	1	2	
429	SPHK1	Adapta	Km app	22	27	24	
430	SPHK2	Adapta	10	75	74	75	2770
431	SRC	ZLYTE	Km app	-8	-2	-5	
432	SRC N1	ZLYTE	Km app	6	8	7	
433	SRMS (Srm)	ZLYTE	Km app	10	17	13	
434	SRPK1	ZLYTE	Km app	12	8	10	
435	SRPK2	ZLYTE	Km app	-7	4	-2	
436	STK16 (PKL12)	Lantha	n/a	-21	-11	-16	
437	STK17A (DRAK1)	Lantha	n/a	9	0	4	
438	STK17B (DRAK2)	Lantha	n/a	7	-11	-2	
439	STK22B (TSSK2)	ZLYTE	Km app	9	14	11	

Entry	Kinase	Technology	ATP	% Inhibition 1	2	% Inhibition mean	IC50 / nM
440	STK22D (TSSK1)	ZLYTE	Km app	4	1	2	
441	STK23 (MSSK1)	ZLYTE	Km app	2	4	3	
442	STK24 (MST3)	ZLYTE	Km app	12	8	10	
443	STK25 (YSK1)	ZLYTE	Km app	2	27	15	
444	STK3 (MST2)	ZLYTE	Km app	-3	-7	-5	
445	STK32B (YANK2)	Lantha	n/a	11	11	11	
446	STK32C (YANK3)	Lantha	n/a	-6	13	4	
447	STK33	Lantha	n/a	8	9	8	
448	STK38 (NDR)	Lantha	n/a	2	17	9	
449	STK38L (NDR2)	Lantha	n/a	-6	-8	-7	
450	STK39 (STLK3)	Lantha	n/a	8	-16	-4	
451	STK4 (MST1)	ZLYTE	Km app	-1	-3	-2	
452	SYK	ZLYTE	Km app	6	9	7	
453	TAOK1	Lantha	n/a	-1	-2	-1	
454	TAOK2 (TAO1)	ZLYTE	Km app	-6	1	-2	
455	TAOK3 (JIK)	Lantha	n/a	-5	6	1	
456	TBK1	ZLYTE	Km app	4	5	5	
457	TEC	Lantha	n/a	-9	-4	-7	
458	TEK (Tie2)	ZLYTE	Km app	8	10	9	
459	TEK (TIE2) R849W	Lantha	n/a	-14	-20	-17	
460	TEK (TIE2) Y1108F	Lantha	n/a	-14	-10	-12	
461	TEK (TIE2) Y897S	ZLYTE	Km app	7	3	5	
462	TESK1	Lantha	n/a	0	14	7	
463	TESK2	Lantha	n/a	-25	-7	-16	
464	TGFBR1 (ALK5)	Lantha	n/a	4	5	5	
465	TGFBR2	Lantha	n/a	-13	-15	-14	
466	TLK1	Lantha	n/a	-3	-6	-5	
467	TLK2	Lantha	n/a	-3	-27	-15	
468	TNIK	Lantha	n/a	7	9	8	
469	TNK1	ZLYTE	Km app	-9	9	0	
470	TNK2 (ACK)	Lantha	n/a	11	4	7	
471	TTK	Lantha	n/a	17	13	15	
472	TXK	ZLYTE	Km app	1	10	5	
473	TYK2	ZLYTE	Km app	0	3	1	
474	TYRO3 (RSE)	ZLYTE	Km app	1	0	1	
475	ULK1	Lantha	n/a	-2	-11	-7	
476	ULK2	Lantha	n/a	7	10	9	
477	ULK3	Lantha	n/a	-14	-4	-9	
478	VRK2	Lantha	n/a	4	0	2	
479	WEE1	Lantha	n/a	-3	14	5	
480	WNK1	Lantha	n/a	-32	-24	-28	
481	WNK2	Lantha	n/a	29	9	19	
482	WNK3	Lantha	n/a	-2	-12	-7	
483	YES1	ZLYTE	Km app	5	5	5	
484	ZAK	Lantha	n/a	0	2	1	
485	ZAP70	ZLYTE	Km app	-3	-7	-5	

5.6 Immunoblotting

Cells were lysed in ice cold lysis buffer (20 mM Tris-HCl pH 8, 300 mM KCl, 10% Glycerol, 0.25% Nonidet P-40, 0.5 mM EDTA, 0.5 mM EGTA, 1 mM PMSF, 1x complete protease inhibitor) and passed 5X trough a 21G needle. After clearance by centrifugation protein concentrations were determined via Bradford assay (Bio-Rad Protein Reagent, cat# 5000006) and lysates normalized. Lysates were mixed with 4X sample buffer, boiled for 10 min, proteins separated by SDS-PAGE, then transferred to nitrocellulose membrane (Bio-Rad, cat# 1704159) using a Trans-Blot Turbo transfer system (Bio-Rad). Membranes were blocked with 5% skim milk (in TBST) and incubated with primary antibody overnight at 4°C. Membranes were washed with TBST and incubated with an HRP-conjugated secondary antibody diluted in 5% skim milk for 1 h at room temperature. After washing, protein detection was carried out using chemiluminescence and imaged using a ChemiDoc imaging system (Bio-Rad).

5.7 Live-cell microscopy

For live-cell imaging of Hek293A EGFP-WIPI2b cells, cells were seeded on poly-l-lysine coated 8-well cover glass bottom chamber slides (Sarstedt, cat# 94.6190.802) and incubated for 24 h. Imaging was performed on a Zeiss Cell Observer spinning disk confocal (ANDOR iXon Ultra) (Carl Zeiss) equipped with a 63x immersion oil objective lens (Plan-Apochromat 1.40 Oil DIC M27) and a temperature-controlled hood maintained at 37°C and 5% CO_2. Quantification of puncta was performed using the spot tracking function of the open-source bioimage processing software, Icy.[23]

5.8 Cellular thermal shift assay (CETSA) in cell lysate

Procedure:

Two T75 cell culture Flasks were seeded with each $6x10^5$ MCF7#2011 cells in 12 mL DMEM and incubated at 37°C and 5% CO_2 for three days. Cells were detached, transferred to two separate tubes and washed three times with PBS. Then 0.6 mL PBS containing 0.04% NP-40 Alternative were added to each tube and cells were lysed by means of freeze and thaw. Either 50 µM of Azaquindole-1 or DMSO were added to the lysates, samples were mixed and incubated for 10 min. Treated and non-treated lysates were divided into ten aliquots, each 50 µL in PCR tubes. The aliquots were individually heated at different temperatures (*Eppendorf Mastercycler ep Gradient S*). After the heat treatment the cell lysates were completely transferred to polycarbonate tubes and centrifuged (*Beckman Optima MAX-TL*) at 100,000 g, 4°C for 25 min. 16 µL of each supernatant was added with 4 µL of 5x loading buffer and incubated for 5 min at 95°C, before samples were analyzed by Immunoblotting. Proteins were separated by SDS-PAGE, and transferred to PDVF membrane using wet transfer. The membranes were blocked with Odyssey Blocking Buffer (TBS; *Li-Cor*) for 1 h and incubated with the primary antibody (PI3 Kinase Class III (D4E2) Rabbit mAb #3358 Cell Signalling in Odyssey blocking buffer containing 0.2% Tween-20) at 4°C over night. After washing with TBS-T (TBS containing 0.1 % Tween) the membrane was incubated with the secondary antibody coupled to IRDye® 800CW (Donkey anti-Rabbit IgG, *Li-COR*) for 1 h, in Odyssey blocking buffer containing 0.2% Tween-20 and 0.1 % SDS at room temperature. Membranes were washed with TBS-T, then TBS before images were taken (*Bio-Rad ChemiDoc™ MP Imaging System*).

Analysis: Images were analyzed with ImageJ (FUJI). Normalisation and all calculations were done with PRISM. (Curves: IC_{50} variable slope → turning points correspond to melting temperature in obtained curves).

5.9 VPS34 kinetic experiments

5.9.1 Experimental protocol

Determination of the kinetic parameters of **10w-j** against VPS34 was carried out by SignalChem (Kanada). VPS34 was cloned, expressed and purified at SignalChem using proprietary methods. Quality control testing is routinely performed on each of the SignalChem targets to ensure compliance to acceptable standards. The protein kinase assays were performed using the ADP-GloTM assay kit purchased from Promega. The assay conditions for the kinases were optimized to yield acceptable enzymatic activity. In addition, the assays were optimized to give high signal-to-noise ratio. The optimized conditions are as follows:

The protein kinase assays were performed in duplicate at room temperature for 40 min in a final volume of 5 µL in 384-well plates according to the following assay reaction recipe:

Component 1. 1 µL of diluted active protein kinase
Component 2. 1 µL of peptide substrate
Component 3. 1 µL kinase assay buffer
Component 4. 1 µL compound (various concentrations) or 10% DMSO
Component 5. 1µL of ATP stock solution (final µM as below)

The following final ATP concentrations were attained in the experiment: 12.5, 25, 50, 100, 200, 400 µM. The assay was started by incubating the reaction mixture in a 384-well plate at room temperature for 40 minutes. After the incubation period, the assay was terminated by the addition of 5 µl of ADP-GloTM Reagent (Promega). The plate was shaken and then incubated for 40 minutes at ambient temperature. Then 10 µL of Kinase Detection Reagent was added, the plate shaken and then incubated for further 30 min at ambient temperature. Readout of the Luminescence signal was performed using the ADP-GloTM Luminescence Protocol on a GloMax plate reader. A Blank control was set up that included all the assay components except the addition of the enzyme (replace with equal volume of kinase assay buffer). The corrected activity for protein kinase targets were determined by removing the blank control value. A nonlinear regression (curve fit) – mixed model inhibition analysis was run on GraphPad Prism using the obtained corrected data. Given Curves were generated from results of three independent runs (n = 3).

5.9.2 Michaelis–Menten Saturation Curves

Plotted below are the mean data ± SD (*n* = 3):

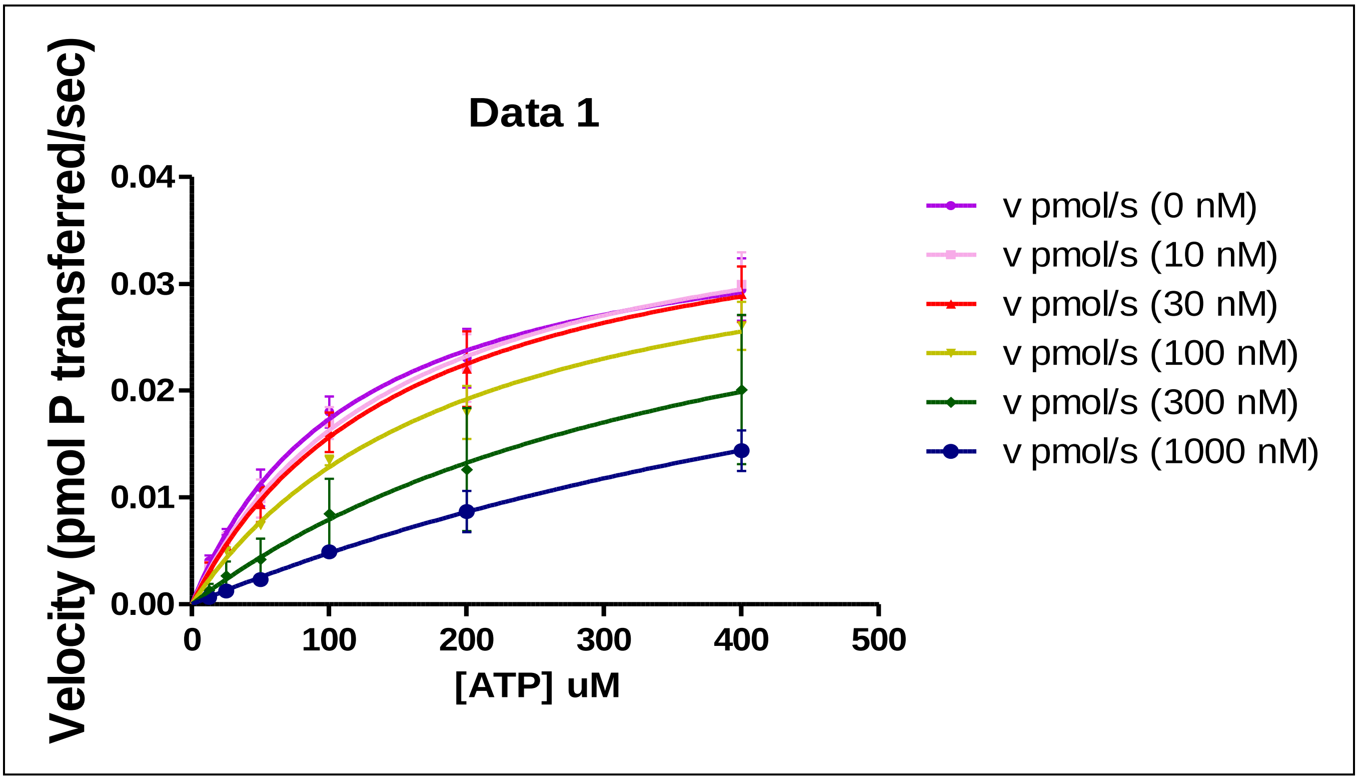

5.9.3 %inhibition versus [*10w-j*] plot

Plotted below are the mean data ± SD (*n* = 3):

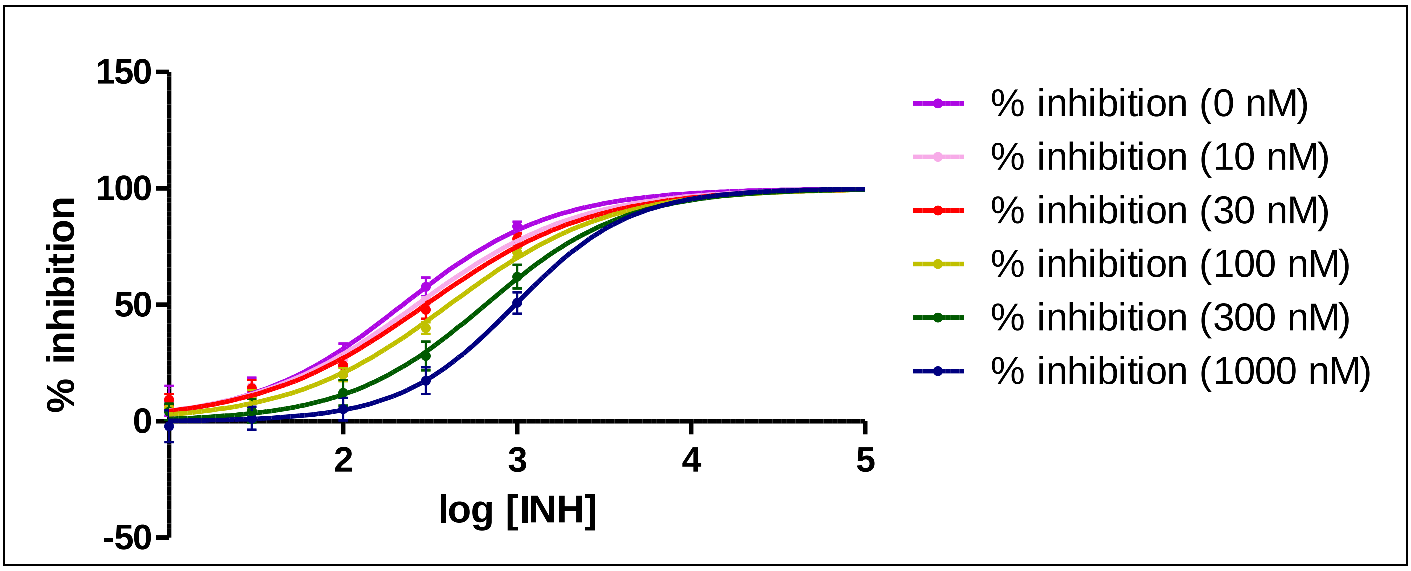

5.9.4 Lineweaver–Burk Plot

Plotted below are the mean data ± SD (*n* = 3):

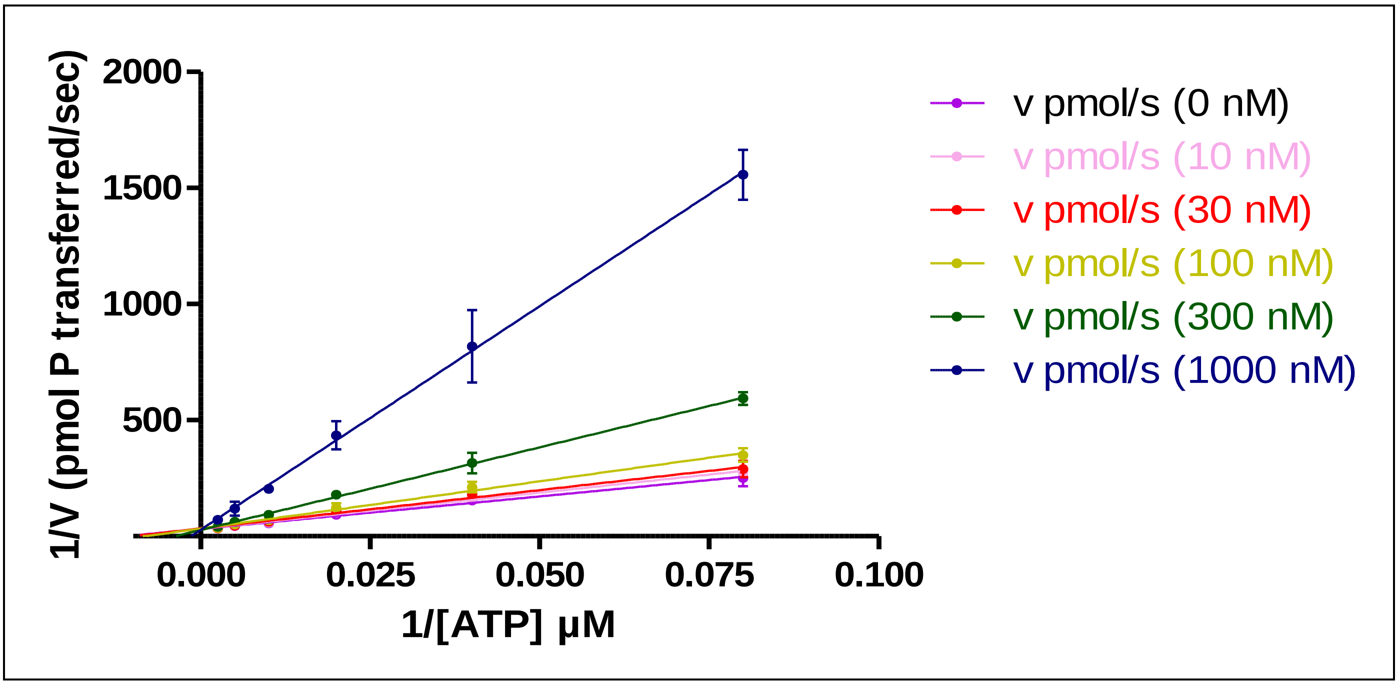

	v pmol/s (0 nM)	v pmol/s (10 nM)	v pmol/s (30 nM)	v pmol/s (100 nM)	v pmol/s (300 nM)	v pmol/s (1000 nM)
V_{max}	0.037786667	0.041013333	0.04029	0.040976667	0.04354667	0.043653333
SD	0.003809544	0.005810074	0.004234745	0.004145362	0.00339038	0.003843491
K_m	117.7666667	153.6	158.6333333	193.6666667	322.5	815.3333333
SD	9.90633916	38.91760527	24.38309979	17.88748787	42.1369988	44.04409407

6.0 Synthetic experimental

6.1 General experimental

All non-aqueous reactions were performed under an atmosphere of N_2 unless otherwise stated. Dry solvents were received from Sigma-Aldrich or Acros in anhydrous quality and used without further purification. All other reaction solvents were HPLC grade. Milli-Q grade water was used in all experiments. Commercially available starting materials were obtained from Acros, Alfa Aesar, Enamine, Fluorochem, Key Organics, Sigma-Aldrich and TCI, and were used without purification.

Thin layer chromatography (TLC) was performed on Merck silica gel aluminium plate with F-254 indicator. Visualisation of the plates was achieved using an ultraviolet lamp (λ_{max} = 254 nm) and/or $KMnO_4$ staining (1.5 g in 400 mL H_2O, 5 g $NaHCO_3$). Flash chromatography was carried out using silica gel (particle size 40-60 μm, 230-400 mesh) from Acros Organics. Chromatography solvents were technical grade.

Mass-directed preparative HPLC purifications were carried out using an Agilent 1100/LC/MSD VL system equipped with a C18 column (flow 20.0 mL/min, solvent A: 0.1% TFA in H_2O, solvent B: 0.1% TFA in MeCN).

Chemical yields refer to pure isolated substances. Optical rotations were measured on a Schmitd + Hansch Polartronic HH8. $[\alpha]_D^{20}$ values are given in [° mL g^{-1} dm^{-1}].

High resolution mass spectra (HRMS) were recorded on a LTQ Orbitrap mass spectrometer coupled to an Accela HPLC-System (HPLC column: Hypersyl GOLD, 50 mm x 1 mm, particle size 1.9 μm, ionisation method: electron spray ionisation).

Proton (^{1}H) and carbon (^{13}C) NMR spectral data were collected on *Bruker AVANCE NEO* (500 MHz), *Bruker AVANCE HD-III* (600 MHz) and *Bruker AVANCE HDX-III* (700 MHz)spectrometers. Chemical shifts (δ) are quoted in parts per million (ppm) and referenced to the residual solvent peak. Coupling constants (*J*) are quoted in Hertz (Hz) and splitting patterns reported in an abbreviated manner: app. (apparent), br. (broad), s (singlet), d (doublet), t (triplet), q (quartet), m (multiplet). All fully characterised products were assigned with the aid of COSY and HSQC experiments. Compounds are numbered with respect to their IUPAC names.

6.2 General procedures

General procedure A: Preparation of the ketones

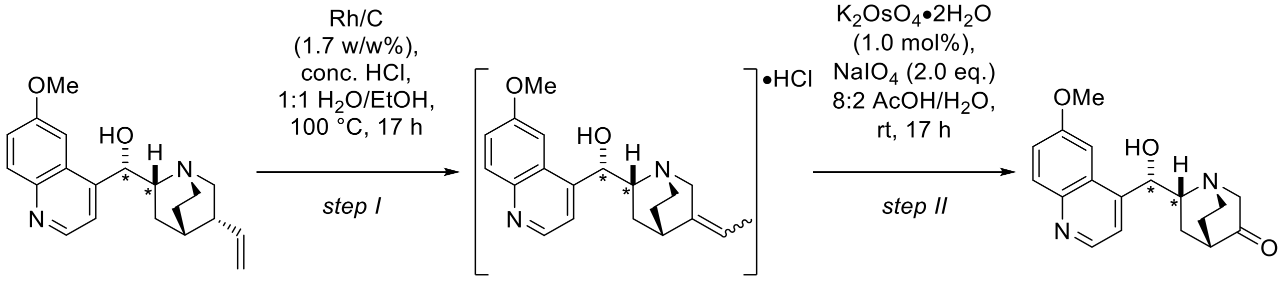

2, *quinidine* *or* **4**, *quinine*; **7** *or* **8**; **5**, 38% (2 steps) *or* **6**, 50% (2 steps)

Step I: Isomerisation of the Cinchona alkaloid double bond:

Following a modification of a procedure by Portlock *et al.*,[24] in two batches, quinidine or quinine (2 × 6.0 g, 2 × 18.4 mmol [18.4 mmol in each batch, 36.8 mmol over 2 batches], 1.0 eq) was dissolved in 1:1 H_2O/EtOH (2 × 120 mL, [120 mL in each batch, 240 mL overall]) and concentrated HCl (2 × 15.6 mL, 181 mmol [181 mmol in each batch, 362 mmol over 2 batches], 10 eq.) was added. Rh/C (5 wt%, 2 × 100 mg [100 mg in each batch, 200 mg overall], 1.7 w/w%) was added and the reaction mixtures were heated at reflux (101.5°C) under Ar for 17 h. The reaction mixtures were cooled to rt and the two batches were combined and filtered through celite, flushing through with MeOH.* The filtrate was concentrated *in vacuo*.† To the resulting oil was added H_2O (100 mL). Lyophilisation (72 h) gave the internal alkenes **7** or **8** as the hydrochloride salts, which were carried forward to the next step without further purification.

Step II: Oxidative cleavage of the alkene to the ketone:

Following a modification of a procedure by Carroll *et al.*,[25] the relevant hydrochloride salt **7** or **8** (5.0 mmol) was dissolved in 8:2 AcOH/H2O (24 mL) and $K_2OsO_4 \cdot 2H_2O$ (18 mg, 0.05 mmol, 1.0 mol%) was added. The reaction mixture was stirred at rt for 10 min, then cooled to 0 °C. $NaIO_4$ (2.14 g, 10.0 mmol, 2.0 eq.) was added. The mixture was warmed to rt and stirred for 17 h. The mixture obtained was quenched with aqueous NaOH (10 M, ~50 mL) at 0°C until it was basic (tested with pH indicator paper). Sat. aq. Na_2SO_3 solution (10 mL) was added to destroy OsO_4. The resulting solution was extracted with 9:1 $CHCl_3$/MeOH (4 x 50 mL) and 8:2 $CHCl_3$/MeOH (2 x 50 mL).

* Analysis of the crude reaction products by ^{1}H NMR spectroscopy showed complete conversion to a 2:1 mixture of *Z*/*E* alkenes.
† The HCl was scrubbed using 2.0 M NaOH (200 mL).

The combined organic fractions were dried over Na_2SO_4, filtered, and concentrated *in vacuo*. Flash column chromatography gave the ketones **5** or **6**.

General Procedure B: Pd-catalysed indole formation

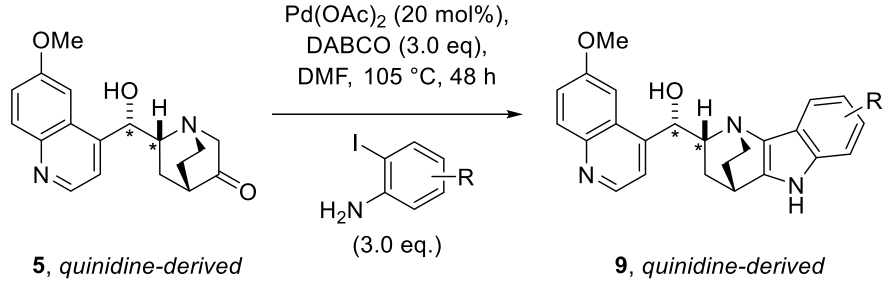

5, *quinidine-derived*
or
6, *quinine-derived*

9, *quinidine-derived*
or
10, *quinine-derived*

Following a modification of a procedure by Chen *et al.*,[26] ketone **5** or **6** (100 mg, 0.32 mmol, 1.0 eq.), 2-iodoaniline (0.96 mmol, 3.0 eq.), DABCO (108 mg, 0.96 mmol, 3.0 eq.) and $Pd(OAc)_2$ (7 mg, 10 mol%) were added to a 7.0 mL reaction vial equipped with a screw-cap lid bearing a PTFE/silicone septum.‡ The reactants were dissolved in anhydrous DMF (1.0 mL, 0.3 M) and degassed under a stream of Argon for 10 min. The lid was additionally sealed with parafilm and transferred to a pre-heated heating block at 105 °C (the solvent level in the vial was submerged below level of the heating block). The reaction progress was monitored every 24 h by HPLC-MS. In case of incomplete conversion,§ the reaction mixture was cooled to rt, and additional $Pd(OAc)_2$ (7 mg, 10 mol%) was added. The resulting solution was degassed under a steam of argon for 10 min and returned to the heating block for additional 24 h. Following complete conversion of starting ketone (or after a maximum 72 h reaction time) the reaction mixture was cooled to rt, diluted in MeOH (5 mL) and passed through a syringe filter**. After removal of the solvents *in vacuo* the products **9** or **10** were purified by column chromatography and/or by mass-directed preparative HPLC (MeCN + 0.1% TFA/H_2O +0.1%TFA).

‡ Supelco-27151 vials and Supelco-27019 lids purchased from Sigma-Aldrich
§ As judged by the presence of the mass (MH^+) of any unreacted starting ketone by LCMS. In cases where the conversion was deemed to be particularly poor (~5-10%), $MgSO_4$ (1.5 eq.) was added to the reaction mixture after 24 h, as stipulated.
** Chromafil® PET-45/15 MS (729023).

6.3 Compound data

6.3.1 Preparation of the Cinchona alkaloid derived ketones

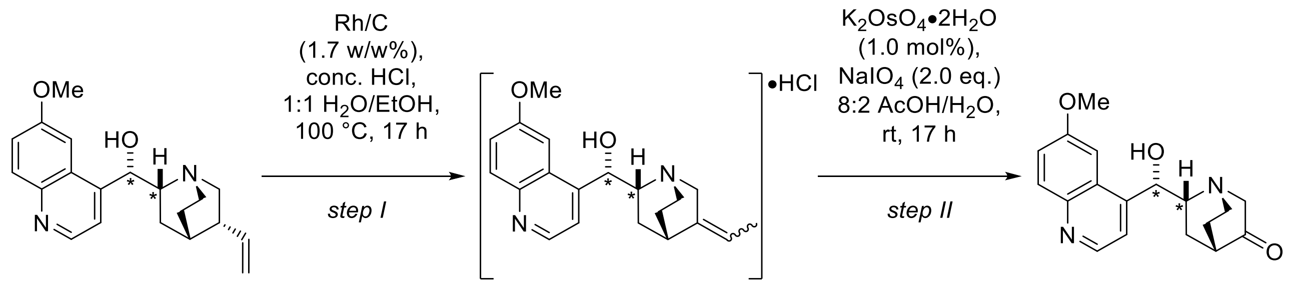

1, *quinidine*
or
4, *quinine*

7
or
8

5, 38% (2 steps)
or
6, 50% (2 steps)

(1*S*,2*R*,4*S*)-5-Ethylidene-2-[(*S*)-hydroxy(6-methoxyquinolin-4-yl)methyl]-1-azabicyclo[2.2.2]octan-1-ium chloride 7

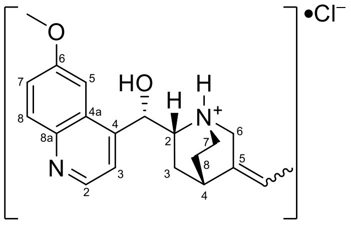

7

General procedure A, Part I, was followed using quinidine in two batches (2 × 6.0 g, 2 × 18.4 mmol [18.4 mmol in each batch, 36.8 mmol over 2 batches]) to give the title product **7** (15.2 g††) as a pale yellow solid which was carried forward to the next step without further purification. **^{1}H NMR** (500 MHz, CD_3OD, 2:1 mixture of Z/E alkenes, NH^+ and OH not observed): δ 9.05-9.01 (1H, m, quinoline 2-H), 8.31-8.28 (1H, m, quinoline 3-H), 8.22 (1H, d, *J* 9.3, quinoline 8-H), 7.91-7.84 (2H, m, quinoline 5-H and 7-H), 6.67 (0.67H, s, major C*H*(OH)), 6.65 (0.33H, s, minor CH(OH)), 5.58-5.52 (0.67H, m, major C=C*H*CH_3), 5.51-5.44 (0.33H, m, minor C=C*H*CH_3), 5.01-4.95 (1H, m, 6-H_A), 4.19 (2H, s, major OCH_3), 4.18 (1H, s, minor OCH_3), 4.09 (0.67H, d, *J* 15.9, major 6-H_B), 3.98-3.86 (1.33H, m, 2-H and minor 6-H_B), 3.64-3.56 (1H, m, 7-H_A), 3.37-3.25 (1H, m, 7-H_B), 3.13 (0.33H, s, minor 4-H), 2.68 (0.67H, s, major 4-H), 2.35 (1H, app. t, *J* 9.7, 3-H_A), 1.99-1.84 (2H, m, 8-H_A and 8-H_B), 1.72 (1H, d, *J* 6.9, minor C=CHC*H*$_3$), 1.63 (2H, d, *J* 6.9, major C=CHC*H*$_3$), 1.60-1.49 (1H, m, 3-H_B). **^{13}C NMR** (125 MHz, CD_3OD, 2:1 mixture of Z/E alkenes): δ 162.6 (2 peaks, Ar-C_q), 158.2 (2 peaks, Ar-C_q), 141.8 (quinoline 2-C), 134.9 (2 peaks, Ar-C_q), 132.6 (major 5-C), 131.7 (minor 5-C), 129.6 (quinoline 7-C), 129.2 (2 peaks, Ar-C_q), 123.9 (quinoline 8-C), 121.3 (quinoline 3-C), 119.5 (minor C=*C*HCH_3), 119.4 (major C=*C*HCH_3), 103.6 (2 peaks, quinoline 5-C), 68.3 (CH(OH)), 60.7 (major 2-C), 60.5 (minor 2-C), 58.5 (major OCH_3), 58.4 (minor OCH_3), 53.1 (minor 6-C), 51.7 (7-C), 51.4 (major 6-C), 32.9 (major 4-C), 26.0 (minor 4-C), 24.6 (2 peaks, major 3-C or major 8-C), 23.9 (minor 3-C or minor 8-C), 23.6 (minor 3-C or minor 8-C), 12.8 (minor C=CH*C*H_3), 12.7 (major C=CH*C*H_3).

†† Maximum theoretical yield = 13.3 g.

(1*S*,2*S*,4*S*)-5-Ethylidene-2-[(*R*)-hydroxy(6-methoxyquinolin-4-yl)methyl]-1-azabicyclo[2.2.2]octan-1-ium chloride 8

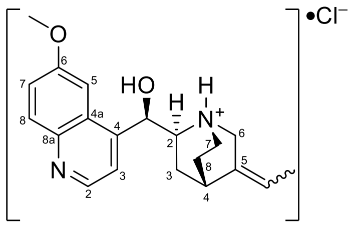

8

General procedure A, Part I, was followed using quinine in two batches (2 × 6.0 g, 2 × 18.4 mmol [18.4 mmol in each batch, 36.8 mmol over 2 batches]) to give the title product **8** (14.9 g‡‡) as a pale yellow solid which was carried forward to the next step without further purification. **^{1}H NMR** (500 MHz, CD_3OD, 2:1 mixture of Z/E alkenes, NH^+ and OH not observed): δ 9.08 (1H, d, *J* 3.4, quinoline 2-H), 8.38 (1H, d, *J* 3.4, quinoline 3-H), 8.26 (1H, d, *J* 8.8, quinoline 8-H), 7.90 (0.67H, s, major quinoline 5-H), 7.88 (0.33H, s, minor quinoline 5-H), 7.83 (1H, d, *J* 8.8, quinoline 7-H), 6.71 (1H, s, C*H*(OH)), 5.56-5.49 (0.67H, m, major C=C*H*CH_3), 5.49-5.43 (0.33H, m, minor C=C*H*CH_3), 4.47-4.39 (1H, m, 7-H_A), 4.23 (1H, d, *J* 16.1, 6-H_A), 4.14-4.01 (1H, m, 6-H_B), 3.85-3.77 (0.67H, m, major 2-H), 3.77-3.67 (0.33H, s, minor 2-H), 3.47-3.38 (1H, m, 7-H_B), 3.17 (0.33H, s, minor 4-H), 2.71 (0.67H, s, major 4-H), 2.52-2.41 (1H, m, 3-H_A), 2.31-2.21 (1H, m, 8-H_A), 2.02-1.91 (1H, m, 8-H_B), 1.63 (1H, d, *J* 4.8, minor C=CHCH_3), 1.57 (2H, d, *J* 5.9, major C=CHCH_3), 1.53-1.41 (1H, m, 3-H_B). **^{13}C NMR** (125 MHz, CD_3OD, 2:1 mixture of Z/E alkenes): δ 162.5 (Ar-C_q), 158.3 (major Ar-C_q), 158.2 (minor Ar-C_q), 141.7 (2 peaks, quinoline 2-C), 134.8 (2 peaks, Ar-C_q), 131.9 (major 5-C), 131.0 (minor 5-C), 129.6 (major Ar-C_q), 129.5 (minor Ar-C_q), 129.1 (quinoline 7-C), 124.0 (minor quinoline 8-C), 123.9 (major quinoline 8-C), 121.4 (quinoline 3-C), 121.2 (minor C=CHCH_3), 120.9 (major C=CHCH_3), 103.6 (major quinoline 5-C), 103.5 (minor quinoline 5-C), 68.1 (minor CH(OH)), 68.0 (major CH(OH)), 61.7 (major 2-C), 61.5 (minor 2-C), 58.7 (major OCH_3), 58.6 (minor OCH_3), 58.2 (minor 6-C), 56.5 (major 6-C), 46.0 (major 7-C), 45.8 (minor 7-C), 32.6 (major 4-C), 25.8 (minor 4-C), 25.6 (major 8-C), 24.7 (major 3-C), 24.5 (minor 8-C), 23.9 (minor 3-C), 13.0 (minor C=CHCH_3), 12.8 (major C=CHCH_3).

‡‡ Maximum theoretical yield = 13.3 g.

(*1S*,*4S*,*6R*)-6-[(*S*)-Hydroxy(6-methoxyquinolin-4-yl)methyl]-1-azabicyclo[2.2.2]octan-3-one 5

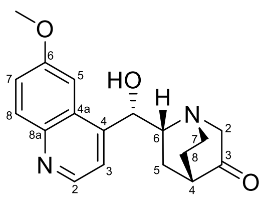

5

General Procedure A, Part II, was followed using compound **7** (2.0 g, assume 4.9 mmol). Flash column chromatography eluting with 5-35% (50:80:1 CH_2Cl_2:EtOH:$NH_4OH_{(sat.\ aq.)}$) in CH_2Cl_2 gave the title product **5** (591 mg,[§§] 1.89 mmol, 38%) as a colourless solid. **^{1}H NMR** (500 MHz, CD_3OD, OH not observed): δ 8.71 (1H, d, *J* 4.6, quinoline 2-H), 8.00 (1H, d, *J* 9.0, quinoline 8-H), 7.72 (1H, d, *J* 4.6, quinoline 3-H), 7.50-7.44 (2H, m, quinoline 5-H and 7-H), 5.76 (1H, d, *J* 2.4, C*H*(OH)), 4.37 (1H, app. dd, *J* 18.7, 1.9, quinuclidine 2-H_A), 4.04 (3H, s, OCH_3), 3.41-3.36 (1H, m, quinuclidine 6-H), 3.26 (1H, d, *J* 18.7, quinuclidine 2-H_B), 3.20-3.10 (1H, m, quinuclidine 7-H_A), 2.99 (1H, ddd, *J* 13.9, 10.2, 6.8, quinuclidine 7-H_B), 2.50-2.38 (2H, m, quinuclidine 4-H and 5-H_A), 2.06-1.89 (2H, m, quinuclidine 8-H), 1.74-1.65 (1H, m, quinuclidine 5-H_B). **^{13}C NMR** (125 MHz, CD_3OD, quinuclidine 2-C not observed due to deuterium exchange): δ 220.5 (quinuclidine 3-C), 159.8 (quinoline 6-C), 150.0 (Ar-C_q), 148.2 (quinoline 2-C), 144.7 (Ar-C_q), 131.4 (quinoline 8-C), 127.9 (Ar-C_q), 123.4 (quinoline 7-C), 119.8 (quinoline 3-C), 102.2 (quinoline 5-C), 71.8 (*C*H(OH)), 59.6 (6-C), 56.4 (OCH_3), 50.9 (quinuclidine 7-C), 42.2 (quinuclidine 4-C), 25.3 (quinuclidine 5-C or 8-C), 25.2 (quinuclidine 5-C or 8-C). **HRMS** (ESI): $C_{18}H_{21}N_2O_3$ $[M+H]^+$; calculated: 313.1549, found: 313.1547. $[\alpha]_{20}^{D}$ = +122 (c. 1.0, MeOH).

[§§] Average yield from 8 reaction attempts.

(1*S*,4*S*,6*S*)-6-[(*R*)-Hydroxy(6-methoxyquinolin-4-yl)methyl]-1-azabicyclo[2.2.2]octan-3-one 6

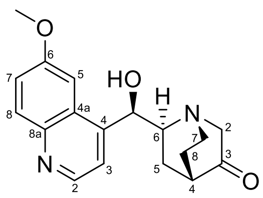

6

General Procedure A, Part II, was followed using compound **8** (2.0 g, assume 5.1 mmol). Flash column chromatography eluting with 5-35% {50:80:1 CH_2Cl_2:EtOH:$NH_4OH_{(sat.\ aq.)}$} in CH_2Cl_2 gave the title product **6** (788 mg,*** 2.52 mmol, 50%) as a colourless solid. **^{1}H NMR** (500 MHz, CD_3OD, OH not observed): δ 8.67 (1H, d, *J* 4.6, quinoline 2-H), 7.94 (1H, d, *J* 9.2, quinoline 8-H), 7.71 (1H, d, *J* 4.6, quinoline 3-H), 7.46 (1H, d, *J* 2.7, quinoline 5-H), 7.42 (1H, dd, *J* 9.2, 2.7, quinoline 7-H), 5.69 (1H, d, *J* 3.7, C*H*(OH)), 3.98 (3H, s, OCH_3), 3.90-3.82 (1H, m, quinuclidine 7-H_A), 3.37-3.23 (3H, m, quinuclidine 2-H_A and 2-H_B and quinuclidine 6-H), 2.88-2.80 (1H, m, quinuclidine 7-H_B), 2.49-2.38 (2 H, m, quinuclidine 4-H and quinuclidine 5-H_A), 2.27-2.19 (1H, m, quinuclidine 8-H_A), 1.97-1.86 (1H, m, quinuclidine 8-H_B), 1.74-1.65 (1H, m, quinuclidine 5-H_B). **^{13}C NMR** (125 MHz, CD_3OD): δ 220.2 (quinuclidine 3-C), 159.7 (quinoline 6-C), 150.5 (Ar-C_q), 148.2 (quinoline 2-C), 144.8 (Ar-C_q), 131.3 (quinoline 8-C), 128.2 (Ar-C_q), 123.4 (quinoline 7-C), 120.0 (quinoline 3-C), 102.6 (quinoline 5-C), 71.9 (CH(OH)), 65.5 (2-C), 61.4 (quinuclidine 6-C), 56.4 (OCH_3), 43.6 (quinuclidine 7-C), 41.9 (quinuclidine 4-C), 26.1 (quinuclidine 5-C), 25.8 (quinuclidine 8-C). **HRMS** (ESI): $C_{18}H_{21}N_2O_3$ $[M+H]^+$; calculated: 313.1547, found: 313.1548. $[\alpha]_{20}^{D}$ = −147 (c. 0.1, MeOH).

*** Average yield from 10 reaction attempts.

6.3.2 Preparation of the Cinchona alkaloid derived indoles

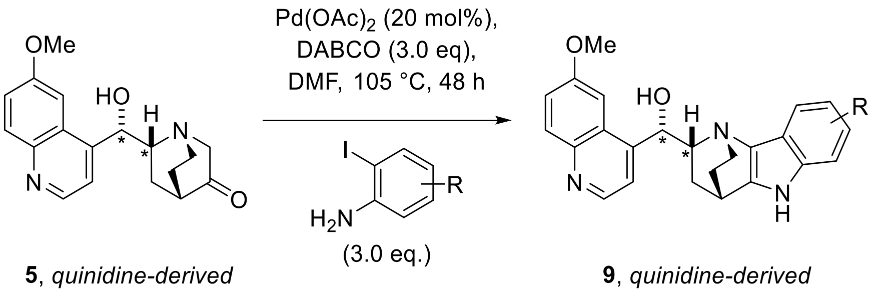

5, *quinidine-derived* *or* **6**, *quinine-derived*

9, *quinidine-derived* *or* **10**, *quinine-derived*

4, 5, 6, 7 = R = H:
9a (49%); **10a** (17%)

4 = R; 5-7 = H. R =
Me: **9b** (49%); **10b** (46%)
CO_2Me: **9c** (18%)[a]; **10c** (12%)[a]
Cl: **9d** (77%)[b]; **10d** (10%)[b]

5 = R; 4, 6-7 = H. R =
Me: **9e** (45%); **10e** (4%)
CO_2H: **9f** (14%)[a,d]; **10f** (9%)[b,d]
CO_2Me: **9g** (3%)[a,d]
CF_3: **9h** (25%); **10h** (23%)[a]
NO_2: **9i** (12%)[a,d]; **10i** (3%)[a,d]
OMe: **9j** (23%)[a,c]; **10j** (6%)[a]
OCF_3: **10k** (65%)[b]
F: **9l** (19%)[d]; **10l** (47%)
Cl: **9m** (48%); **10m** (59%)[b,d]
Br: **9n** (8%)[d]; **10n** (15%)

6 = R; 4-5, 7 = H. R =
Me: **9o** (12%)[a,d]; **10o** (4%)
CO_2H: **9p** (11%)[b,d]; **10p** (14%)[d]
CO_2Me: **9q** (7%)[a]
CF_3: **9r** (25%); **10r** (70%)[b]
Cl: **9s** (26%); **10s** (20%)
Br: **9t** (9%)[a,d]; **10t** (27%)[a]

7 = R; 4-6 = H. R =
OMe: **9u** (16%)[a,d]; **10u** (33%)[a,d]

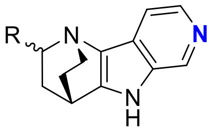

9v (50%)[d]; **10v** (65%)[b]

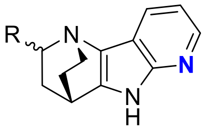

9w (17%)[d]; **10w** (13%)[a,d]

6.3.2.1 Preparation of the quinidine-derived indoles

(*S*)-(*11S*,*13R*)-1,9-Diazatetracyclo[9.2.2.0^{2},^{10}.0^{3,8}]pentadeca-2(10),3(8),4,6-tetraen-13-yl(6-methoxyquinolin-4-yl)methanol 9a

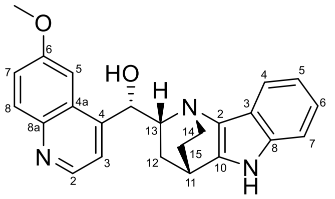

9a

Prepared according to General Procedure B using ketone **5** and 20 mol% $Pd(OAc)_2$ (48 h). Flash column chromatography eluting with 20% {50:80:1 CH_2Cl_2:EtOH:NH_4OH} in CH_2Cl_2 gave the *title compound* **9a** (60 mg, 0.16 mmol, 49%) as a pale brown solid. **^{1}H NMR** (500 MHz, CD_3OD, NH and OH not observed): δ 8.70 (1H, d, *J* 4.6, quinoline 2-H), 7.95 (1H, d, *J* 9.2, quinoline 8-H), 7.53 (1H, d, *J* 4.6, quinoline 3-H), 7.39-7.34 (2H, m, 7-H and quinoline 7-H), 7.16 (1H, d, *J* 2.3, quinoline 5-H), 7.03-6.97 (1H, m, 6-H), 6.85-6.76 (2H, m, 4-H and 5-H), 5.47 (1H, s, OH), 4.55 (1H, d, *J* 9.0, C*H*(OH)), 4.01-3.90 (1H, m, 13-H), 3.52-3.48 (1H, m, 11-H), 3.42 (3H, s, OCH_3), 3.23-3.13 (1H, m, 14-H_A), 2.46 (1H, app. td, *J* 11.1, 5.1, 14-H_B), 2.27 (1H, ddd, *J* 12.4, 8.2, 3.0, 12-H_A), 2.00-1.92 (1H, m, 15-H_A), 1.91-1.84 (1H, m, 12-H_B), 1.55-1.46 (1H, m, 15-H_B). **^{13}C NMR** (125 MHz, CD_3OD, 1 × Ar-C_q not observed): δ 159.0 (quinoline 6-C), 150.2 (Ar-C_q), 148.2 (quinoline 2-C), 145.2 (Ar-C_q), 142.7 (Ar-C_q), 135.9 (Ar-C_q), 131.0 (quinoline 8-C), 129.2 (Ar-C_q), 124.0 (Ar-C_q), 123.6 (quinoline 7-C), 121.5 (quinoline 3-C), 121.1 (6-C), 120.2 (4-C or 5-C), 117.6 (4-C or 5-C), 112.6 (7-C), 103.4 (quinoline 5-C), 74.0 (CH(OH)), 66.4 (13-C), 55.6 (OCH_3), 54.8 (14-C), 35.2 (12-C), 29.2 (11-C), 28.9 (15-C). **HRMS** (ESI): $C_{24}H_{24}N_3O_2$ $[M+H]^+$; calculated: 386.1869, found: 386.1866. $[\boldsymbol{\alpha}]_{\mathbf{20}}^{\boldsymbol{D}}$ = +116 (c. 0.2, MeOH).

(*S*)-(6-Methoxyquinolin-4-yl)[(*11S*,*13R*)-4-methyl-1,9-diazatetracyclo[9.2.2.0^{2,10}.0^{3,8}]pentadeca-2(10),3(8),4,6-tetraen-13-yl]methanol 9b

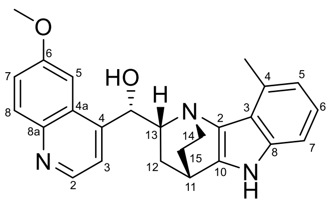

9b

Prepared according to General Procedure B using ketone **5** and 20 mol% Pd(OAc)$_2$ (48 h). Flash column chromatography eluting with 5-35% {50:80:1 CH_2Cl_2:EtOH:NH_4OH} in CH_2Cl_2 gave the *title compound* **9b** (63 mg, 0.16 mmol, 49%) as a brown solid. **^{1}H NMR** (500 MHz, CD_3OD, NH and OH not observed): δ 8.67 (1H, d, *J* 4.7, quinoline 2-H), 7.88 (1H, d, *J* 9.2, quinoline 8-H), 7.65 (1H, d, *J* 4.7, quinoline 3-H), 7.29 (1H, dd, *J* 9.2, 2.6, quinoline 7-H), 7.10 (1H, d, *J* 8.1, 7-H), 6.88 (1H, d, *J* 2.6, quinoline 5-H), 6.80 (1H, app. t, *J* 7.6, 6-H), 6.40 (1H, d, *J* 7.1, 5-H), 4.59 (1H, d, *J* 8.9, C*H*(OH)), 3.86 (1H, app. td, *J* 8.5, 5.2, 13-H), 3.43-3.39 (1H, m, 11-H), 3.26-3.21 (4H, m, includes 14-H_A; and at δ 3.26: 3H, s, OCH_3), 2.52 (1H, ddd, *J* 12.4, 10.8, 4.9, 14-H_B), 2.21-2.14 (1H, m, 15-H_A), 1.92-1.82 (2H, m, 12-H_A and 15-H_B), 1.47-1.38 (4H, m, includes 12-H_B; and at δ 1.44: 3H, s, CH_3). **^{13}C NMR** (125 MHz, CD_3OD): δ 159.2 (quinoline 6-C), 150.2 (Ar-C_q), 148.1 (quinoline 2-C), 145.0 (Ar-C_q), 142.5 (Ar-C_q), 135.7 (Ar-C_q), 130.8 (quinolone 8-C), 129.8 (Ar-C_q), 129.2 (Ar-C_q), 124.0 (Ar-C_q), 123.9 (Ar-C_q), 123.7 (quinolone 7-C), 121.0 (5-C or 6-C), 120.9 (5-C or 6-C), 120.6 (quinoline 3-C), 109.8 (7-C), 102.3 (quinoline 5-C), 72.2 (CH(OH)), 65.5 (13-C), 55.5 (OCH_3), 54.1 (14-C), 35.2 (15-C), 29.2 (11-C and 12-C), 17.9 (C_qCH_3). **HRMS** (ESI): $C_{25}H_{26}N_3O_2$ $[M+H]^+$; calculated: 400.2020, found: 400.2010. $[\alpha]_{20}^{D}$ = +317 (c. 0.1, MeOH).

Methyl (*11S*,*13R*)-13-[(*S*)-hydroxy(6-methoxyquinolin-4-yl)methyl]-1,9-diazatetracyclo[9.2.2.0^{2,10}.0^{3,8}]pentadeca-2(10),3(8),4,6-tetraene-4-carboxylate 9c

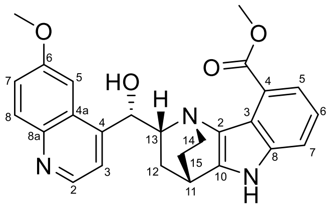

9c

Prepared according to General Procedure B using ketone **5** and 30 mol% $Pd(OAc)_2$ (72 h). Flash column chromatography eluting with 5-20% {50:80:1 CH_2Cl_2:EtOH:NH_4OH} in CH_2Cl_2 gave the *title compound* **9c** (25 mg, 56 µmol, 18%) as a pale brown solid. **^{1}H NMR** (700 MHz, CD_3OD, NH and OH not observed): δ 8.75 (1H, d, *J* 4.4, quinoline 2-H), 8.15 (1H, app. s, 5-H), 8.01 (1H, d, *J* 9.1, quinoline 8-H), 7.57 (1H, d, *J* 8.3, 7-H), 7.55 (1H, d, *J* 4.4, quinoline 3-H), 7.43 (1H, dd, *J* 9.1, 2.3, quinoline 7-H), 7.20 (1H, s, quinoline 5-H), 6.85 (1H, app. d, *J* 8.2, 6-H), 4.58 (1H, d, *J* 8.6, C*H*(OH)), 4.02-3.94 (1H, m, 13-H), 3.94 (3H, s, CO_2CH_3), 3.57 (1H, app. s, 11-H), 3.49 (3H, s, $ArOCH_3$), 3.27-3.21 (1H, m, 14-H_A), 2.54-2.48 (1H, m, 14-H_B), 2.35-2.30 (1H, m, 15-H_A), 2.07-2.00 (1H, m, 12-H_A), 1.95-1.89 (1H, m, 15-H_B), 1.60-1.53 (1H, m, 12-H_B). **^{13}C NMR** (125 MHz, CD_3OD): δ 170.1 ($\mathit{C}O_2CH_3$), 159.1 (quinoline 6-C), 150.3 (quinoline 2-C), 148.2 (Ar-C_q), 147.7 (Ar-C_q), 145.2 (Ar-C_q), 135.0 (Ar-C_q), 131.1 (quinoline 8-C), 129.1 (Ar-C_q), 127.5 (Ar-C_q), 123.5 (quinoline 7-C), 123.1 (Ar-C_q), 122.3 (Ar-C_q), 121.4 (2 peaks, 7-C and quinoline 3-C), 117.0 (6-C), 115.1 (5-C), 103.4 (quinoline 5-C), 74.0 (CH(OH)) 66.2 (13-C), 55.5 ($ArOCH_3$), 54.3 (14-C), 52.4 ($CO_2\mathit{C}H_3$), 34.9 (15-C), 29.4 (11-C), 28.7 (12-C). **HRMS** (ESI): $C_{26}H_{26}N_3O_4$ $[M+H]^+$; calculated: 444.1918, found: 444.1916. $[\alpha]_{20}^{D}$ = +56.5 (c. 0.2, MeOH).

(*S*)-[(*11S*,*13R*)-4-Chloro-1,9-diazatetracyclo[9.2.2.0^{2,10}.0^{3,8}]pentadeca-2(10),3(8),4,6-tetraen-13-yl](6-methoxyquinolin-4-yl)methanol 9d

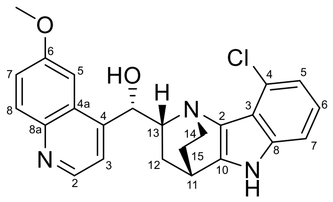

9d

Prepared according to General Procedure B using ketone **5** and 10 mol% $Pd(OAc)_2$ (24 h). Flash column chromatography eluting with 5-35% {50:80:1 CH_2Cl_2:EtOH:NH_4OH} in CH_2Cl_2 gave the *title compound* **9d** (103 mg, 0.25 mmol, 77%) as a brown solid. **^{1}H NMR** (500 MHz, CD_3OD, NH and OH not observed): δ 8.65 (1H, d, *J* 4.7, quinoline 2-H), 7.91 (1H, d, *J* 9.2, quinoline 8-H), 7.60 (1H, d, *J* 4.7, quinoline 3-H), 7.34 (1H, dd, *J* 9.2, 2.6, quinoline 7-H), 7.29 (1H, d, *J* 8.1, 5-H), 7.18 (1H, d, *J* 2.6, quinoline 5-H), 6.91 (1H, app. t, *J* 7.8, 6-H), 6.76 (1H, d, *J* 7.5, 7-H), 4.83 (1H, d, *J* 7.5, C*H*(OH)), 3.86 (1H, app. dd, *J* 13.4, 7.8, 13-H), 3.54 (3H, s, OCH_3), 3.50-3.44 (1H, m, 11-H), 3.30-3.26 (1H, m, 14-H_A), 2.60 (1H, ddd, *J* 12.5, 10.8, 5.1, 14-H_B), 2.11-2.04 (1H, m, 15-H_A), 1.96-1.85 (2H, m, 12-H_A and 15-H_B), 1.54-1.45 (1H, m, 12-H_B). **^{13}C NMR** (125 MHz, CD_3OD): δ 159.3 (quinoline 6-C), 150.0 (Ar-C_q), 148.0 (quinoline 2-C), 145.0 (Ar-C_q), 144.4 (Ar-C_q), 136.9 (Ar-C_q), 130.9 (quinoline 8-C), 129.6 (Ar-C_q), 124.4 (Ar-C_q), 123.4 (quinoline 7-C), 122.8 (Ar-C_q), 122.3 (Ar-C_q), 121.3 (6-C), 120.7 (7-C and quinoline 3-C), 111.3 (5-C), 102.7 (quinoline 5-C), 72.7 (CH(OH)), 65.3 (13-C), 55.8 (OCH_3), 54.3 (14-C), 33.9 (15--C), 29.3 (11-C), 28.8 (12-C). **HRMS** (ESI): $C_{24}H_{23}{}^{35}ClN_3O_2$ $[M+H]^+$; calculated: 420.1473, found: 420.1473. $[\alpha]_{20}^{D}$ = +186 (c. 0.1, MeOH).

(*S*)-(6-Methoxyquinolin-4-yl)[(*11S*,*13R*)-5-methyl-1,9-diazatetracyclo[9.2.2.0^{2,10}.0^{3,8}]pentadeca-2(10),3(8),4,6-tetraen-13-yl]methanol 9e

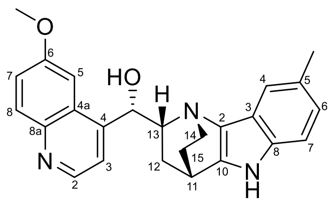

9e

Prepared according to General Procedure B using ketone **5** and 20 mol% $Pd(OAc)_2$ (48 h). Flash column chromatography eluting with 5-20% {50:80:1 CH_2Cl_2:EtOH:NH_4OH} in CH_2Cl_2 gave the *title compound* **9e** (58 mg, 0.15 mmol, 45%) as a pale brown solid. **^{1}H NMR** (700 MHz, CD_3OD, NH and OH not observed): δ 8.77 (1H, d, *J* 4.6, quinoline 2-H), 8.01 (1H, d, *J* 9.1, quinoline 8-H), 7.64 (1H, d, *J* 4.6, quinoline 3-H), 7.41 (1H, dd, *J* 9.1, 2.5, quinolone 7-H), 7.27 (1H, d, *J* 8.2, 7-H), 7.01 (1H, d, *J* 2.5, quinoline 5-H), 6.86 (1H, dd, *J* 8.2, 1.0, 6-H), 6.38 (1H, s, 4-H), 4.58 (1H, d, *J* 9.3, C*H*(OH)), 3.93 (1H, td, *J* 8.5, 5.2, 13-H), 3.52-3.49 (1H, m, 11-H), 3.37 (3H, s, OCH_3), 3.20 (1H, ddd, *J* 12.6, 8.7, 4.0, 14-H_A), 2.50-2.44 (1H, m, 14-H_B), 2.34 (1H, ddd, *J* 12.6, 8.1, 3.0, 12-H_A), 2.21 (3H, s, CH_3), 2.02-1.97 (1H, m, 15-H_A), 1.91-1.87 (1H, m, 12-H_B), 1.57-1.51 (1H, m, 15-H_B). **^{13}C NMR** (175 MHz, CD_3OD): δ 158.9 (quinoline 6-C), 150.6 (Ar-C_q), 148.3 (quinoline 2-C), 145.4 (Ar-C_q), 142.9 (Ar-C_q), 134.3 (Ar-C_q), 130.9 (quinolone 8-C), 129.3 (Ar-C_q), 129.2 (Ar-C_q), 124.2 (Ar-C_q), 123.7 (quinoline 7-C), 122.6 (6-C), 121.9 (Ar-C_q), 121.1 (quinoline 3-C), 117.8 (4-C), 112.2 (7-C), 103.2 (quinoline 5-C), 73.2 (CH(OH)), 66.8 (13-C), 55.4 (OCH_3), 54.2 (14-C), 35.6 (12-C), 29.2 (11-C or 15-C), 29.1 (11-C or 15-C), 21.6 (CH_3). **HRMS** (ESI): $C_{25}H_{26}N_3O_2$ [M+H]$^+$; calculated: 400.2020, found: 400.2017. $[\alpha]_{20}^{D}$ = −12 (c. 0.1, MeOH).

(*1R*,*11S*,*13R*)-5-Carboxy-13-[(*S*)-hydroxy(6-methoxyquinolin-4-yl)methyl]-1,9-diaza-tetracyclo[9.2.2.0^{2,10}.0^{3,8}]pentadeca-2(10),3(8),4,6-tetraen-1-ium trifluoroacetate 9f

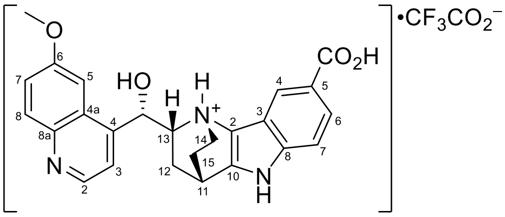

9f

Prepared according to General Procedure B using ketone **5** and 30 mol% $Pd(OAc)_2$ (72 h). Purification by mass-directed preparative HPLC (MeCN in H_2O + 0.1% TFA; Rt = 6.2 min) followed by flash column chromatography eluting with 1:9 MeOH/CH_2Cl_2 gave the *title compound* **9f** (24 mg, 45 μmol, 14%) as a colourless solid. **^{1}H NMR** (600 MHz, CD_3OD, CO_2H, NH^+, NH and OH not observed): δ 8.93 (1H, d, *J* 5.4, quinoline 2-H), 8.17 (1H, d, *J* 9.4, quinoline 8-H), 8.03 (1H, d, *J* 5.5, quinoline 3-H), 7.92-7.87 (2H, m, indole 4-H and indole 6-H), 7.75 (1H, dd, *J* 9.4, 2.5, quinoline 7-H), 7.58 (1H, d, *J* 8.6, indole 7-H), 7.39 (1H, d, *J* 2.5, quinoline 5-H), 5.56 (1H, d, *J* 6.0, C*H*(OH)), 4.65-4.60 (1H, m, 13-H), 4.00-3.93 (1H, m, 14-H_A), 3.87-3.83 (1H, m, 11-H), 3.69 (3H, s, OCH_3), 3.19-3.11 (1H, m, 14-H_B), 2.38-2.32 (1H, m, 12-H_A), 2.31-2.22 (1H, m, 15-H_A), 2.15-2.10 (1H, m, 12-H_B), 1.91-1.83 (1H, m, 15-H_B). **^{13}C NMR** (125 MHz, CD_3OD, *C*$F_3CO_2^-$ not observed): δ 170.4 (CO_2H), 170.0 (indole 5-C), 163.1 (q, J_{CF} 38.1, CF_3*C*O_2^-), 161.6 (quinoline 6-C), 150.2 (Ar-C_q), 143.1 (quinoline 2-C), 141.6 (Ar-C_q), 138.2 (Ar-C_q), 129.4 (Ar-C_q), 128.5 (quinoline 7-C), 125.3 (quinoline 8-C), 124.5 (indole 4-C or indole 6-C), 124.5 (Ar-C_q), 122.4 (Ar-C_q), 122.1 8 (quinoline 3-C), 120.4 (indole 4-C or indole 6-C), 114.1 (Ar-C_q), 113.2 (indole 7-C), 103.4 (quinoline 5-C), 69.7 (CH(OH)), 68.1 (13-C), 57.1 (14-C), 56.6 (OCH_3), 30.6 (12-C), 28.6 (11-C), 25.4 (15-C). **HRMS** (ESI): $C_{25}H_{24}O_4N_3$ $[M+H]^+$; calculated: 430.17613, found: 430.17615. $[\boldsymbol{\alpha}]_{\mathbf{20}}^{\boldsymbol{D}}$ = +42 (c. 0.1, MeOH).

(*1R*,*11S*,*13R*)-13-[(*S*)-hydroxy(6-methoxyquinolin-4-yl)methyl]-5-(methoxycarbonyl)-1,9-diazatetracyclo[9.2.2.0^{2,10}.0^{3,8}]pentadeca-2(10),3(8),4,6-tetraen-1-ium trifluoroacetate 9g

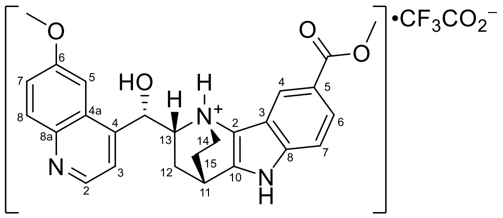

9g

Prepared according to General Procedure B using ketone **5** and 30 mol% Pd(OAc)$_2$ (72 h). Flash column chromatography eluting with 1:9 to 1:4 ({50:80:1 CH_2Cl_2:EtOH:NH_4OH}/CH_2Cl_2) followed by mass-directed preparative HPLC (MeCN in H_2O + 0.1% TFA; Rt = 12 min) gave the *title compound* **9g** (6 mg, 10 μmol, 3%) as a yellow solid. **^{1}H NMR** (600 MHz, CD_3OD, NH^+, NH and OH not observed): δ 8.86 (1H, d, *J* 5.0, quinoline 2-H), 8.12 (1H, d, *J* 9.3, quinoline 8-H), 7.89 (1H, dd, *J* 8.6, 1.4, indole 6-H), 7.86 (1H, d, *J* 5.0, quinoline 2-H), 7.73 (1H, s, indole 4-H), 7.61 (1H, dd, *J* 9.3, 2.6, quinoline 7-H), 7.58 (1H, d, *J* 8.6, indole 7-H), 7.19 (1H, d, *J* 2.3, quinoline 5-H), 5.28 (1H, d, *J* 6.7, C*H*(OH)), 4.59-4.55 (1H, m, 13-H), 3.94-3.88 (4H, m, CO_2CH_3 or OCH_3 and 14-H_A), 3.86-3.83 (1H, m, 11-H), 3.56 (3H, s, CO_2CH_3 or OCH_3), 3.11-3.04 (1H, m, 14-H_B), 2.45-2.39 (1H, m, 12-H_A), 2.32-2.26 (1H, m, 15-H_A), 2.13-2.09 (1H, m, 12-H_B), 1.88-1.81 (1H, m, 15-H_B). **^{13}C NMR** (150 MHz, CD_3OD, *C*$F_3CO_2^-$ not observed): δ 169.1 (*C*O_2CH_3), 163.2 (q, J_{CF} 37.2, CF_3*C*O_2^-), 160.7 (quinoline 6-C), 145.6 (quinoline 2-C), 142.1 (Ar-C_q), 141.9 (Ar-C_q), 138.2 (Ar-C_q), 128.9 (Ar-C_q), 128.2 (quinolone 8-C), 126.5 (quinoline 7-C), 124.2 (Ar-C_q), 124.0 (Ar-C_q), 123.8 (indole 6-C), 121.8 (quinoline 3-C), 120.2 (Ar-C_q), 119.9 (Ar-C_q), 113.3 (indole 7-C), 102.9 (quinoline 5-C), 70.0 (CH(OH)), 68.4 (13-C), 56.9 (14-C), 56.2 (CO_2*C*H_3 or OCH_3), 52.4 (CO_2*C*H_3 or OCH_3), 31.3 (12-C), 28.7 (11-C), 25.7 (15-C). **HRMS** (ESI): $C_{26}H_{26}O_4N_3$ $[M+H]^+$; calculated: 444.1918, found: 444.1918; $C_{26}H_{25}O_4NaN_3$ $[M+Na]^+$; calculated: 466.1737, found: 466.1738. $[\alpha]_{20}^{D}$ = +150 (c. 0.1, MeOH).

(*S*)-(6-Methoxyquinolin-4-yl)[(*11S*,*13R*)-5-(trifluoromethyl)-1,9-diazatetracyclo[9.2.2.0^{2,10}.0^{3,8}]pentadeca-2(10),3(8),4,6-tetraen-13-yl]methanol 9h

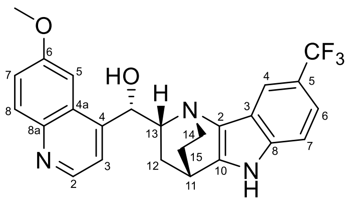

9h

Prepared according to General Procedure B using ketone **5** and 20 mol% Pd(OAc)$_2$ (48 h). Flash column chromatography eluting with 1:9 to 1:4 ({50:80:1 CH_2Cl_2:EtOH:NH_4OH}/CH_2Cl_2) gave the *title compound* **9h** (36 mg, 80 μmol, 25%) as a yellow solid. **^{1}H NMR** (500 MHz, CD_3OD): δ 8.71 (1H, d, *J* 4.6, quinoline 2-H), 7.97 (1H, d, *J* 9.3, quinoline 8-H), 7.54-7.49 (2H, m, quinoline 3-H and indole 7-H), 7.38 (1H, dd, *J* 9.3, 2.7, quinoline 7-H), 7.27 (1H, dd, *J* 8.6, 1.7, indole 6-H), 7.10 (1H, d, *J* 2.7, quinoline 5-H), 7.07 (1H, s, indole 4-H), 4.56 (1H, d, *J* 8.8, C*H*(OH)), 3.95 (1H, app. td, *J* 8.5, 5.3, 13-H), 3.57-3.54 (1H, m, 11-H), 3.41 (3 H, s, OCH_3), 3.26-3.20 (1H, m, 14-H_A), 2.51-2.44 (1H, m, 14-H_B), 2.35-2.28 (1H, m, 12-H_A), 2.01-1.98 (1H, m, 15-H_A), 1.94-1.84 (1H, m, 12-H_B), 1.58-1.46 (1H, m, 15-H_B). **^{13}C NMR** (125 MHz, CD_3OD): δ 159.1 (quinoline 6-C), 150.2 (Ar-C_q), 145.3 (quinoline 2-C), 145.2 (Ar-C_q), 137.1 (Ar-C_q), 130.9 (quinoline 8-C), 129.0 (Ar-C_q), 128.0 (Ar-C_q), 126.9 (q, J_{CF} 270.7, CF_3), 125.8 (Ar-C_q), 123.8 (quinoline 7-C), 123.3 (Ar-C_q), 122.5 (q, J_{CF} 31.3, indole 5-C), 121.3 (quinoline 3-C), 117.5 (q, J_{CF} 3.8, indole 6-C), 115.1 (q, J_{CF} 4.6, indole 4-C), 113.1 (indole 7-C), 102.9 (quinoline 5-C), 73.8 (CH(OH)), 66.2 (13-C), 55.4 (OCH_3), 54.3 (14-C), 35.1 (12-C), 29.3 (11-C), 28.9 (15-C). **HRMS** (ESI): $C_{25}H_{23}O_2N_3F_3$ $[M+H]^+$; calculated: 454.1737, found: 454.1734. $[\alpha]_{20}^{D}$ = +174 (c. 0.1, MeOH).

(*1R*,*11S*,*13R*)-13-[(*S*)-Hydroxy(6-methoxyquinolin-4-yl)methyl]-5-nitro-1,9-diazatetracyclo[9.2.2.0^{2,10}.0^{3,8}]pentadeca-2(10),3(8),4,6-tetraen-1-ium trifluoroacetate 9i

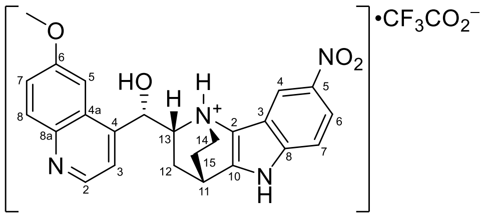

9i

Prepared according to General Procedure B using ketone **5** and 30 mol% $Pd(OAc)_2$ (96 h). Flash column chromatography eluting with 1:9 to 1:3 ({50:80:1 CH_2Cl_2:EtOH:NH_4OH}/CH_2Cl_2) followed by mass-directed preparative HPLC (MeCN in H_2O + 0.1% TFA; Rt = 9.0 min) gave the *title compound* **9i** (20 mg, 38 µmol, 12%) as a yellow solid. **^{1}H NMR** (500 MHz, CD_3OD, NH^+, NH and OH not observed): δ 8.93 (1H, d, *J* 5.5, quinoline 2-H), 8.18 (1H, d, *J* 9.4, quinoline 8-H), 8.16 (1H, d, *J* 2.4, indole 4-H), 8.11 (1H, dd, *J* 9.1, 2.4, indole 6-H), 8.01 (1H, d, *J* 5.5, quinoline 3-H), 7.73 (1H, dd, *J* 9.4, 2.6, quinoline 7-H), 7.65 (1H, d, *J* 9.1, indole 7-H), 7.43 (1H, d, *J* 2.6, quinoline 5-H), 5.70 (1H, d, *J* 5.4, C*H*(OH)), 4.55-4.50 (1H, m, 13-H), 3.97-3.92 (1H, m, 14-H_A), 3.87-3.83 (1H, m, 11-H), 3.74 (3 H, s, OCH_3), 3.17-3.10 (1H, m, 14-H_B), 2.29-2.21 (2 H, m, 12-H_A and 15-H_A), 2.12-2.06 (1H, m, 15-H_B), 1.90-1.81 (1H, m, 12-H_B). **^{13}C NMR** (125 MHz, CD_3OD): δ 162.7 (d, J_{CF} 36.5, $CF_3CO_2^-$), 161.5 (quinoline 6-C), 155.8 (Ar-C_q), 144.0 (Ar-C_q), 143.5 (Ar-C_q), 143.3 (quinoline 2-C), 138.6 (Ar-C_q), 137.3 (Ar-C_q), 129.3 (Ar-C_q), 128.3 (quinoline 7-H), 125.5 (quinoline 8-C), 121.8 (quinoline 3-C), 119.9 (Ar-C_q), 118.0 (q, J_{CF} 291.5, $CF_3CO_2^-$), 117.9 (indole 6-C), 116.2 (Ar-C_q), 114.7 (indole 4-C), 113.7 (indole 7-C), 103.3 (quinoline 5-C), 69.5 (CH(OH)), 67.6 (13-C), 56.7 (OCH_3), 56.7 (14-C), 30.5 (12-C), 28.8 (11-C), 25.4 (15-C). **HRMS** (ESI): $C_{24}H_{23}O_4N_4$ $[M+H]^+$; calculated: 431.1714 found: 431.1709. $[\alpha]_{20}^{D}$ = +199 (c. 0.1, MeOH).

(*S*)-[(*11S*,*13R*)-5-Methoxy-1,9-diazatetracyclo[9.2.2.0^{2,10}.0^{3,8}]pentadeca-2(10),3(8),4,6-tetraen-13-yl](6-methoxyquinolin-4-yl)methanol 9j

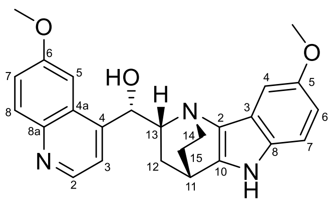

9j

Prepared according to General Procedure B using ketone **5** and 6.0 eq. DABCO (added in one portion at the start of the reaction) and 30 mol% $Pd(OAc)_2$ (72 h). Flash column chromatography eluting with 5-50% {50:80:1 CH_2Cl_2:EtOH:NH_4OH} in CH_2Cl_2 gave the *title compound* **9j** (31 mg, 74 μmol, 23%) as a colourless solid. **^{1}H NMR** (500 MHz, CD_3OD, NH and OH not observed): δ 8.76 (1H, d, *J* 4.6, quinoline 2-H), 8.00 (1H, d, *J* 9.2, quinoline 8-H), 7.64 (1H, d, *J* 4.6, quinoline 3-H), 7.42 (1H, dd, *J* 9.2, 2.5, quinoline 7-H), 7.30 (1H, d, *J* 8.8, 7-H), 7.08 (1H, d, *J* 2.5, quinoline 5-H), 6.72 (1H, dd, *J* 8.8, 2.1, 6-H), 6.25 (1H, d, *J* 2.1, 4-H), 4.74 (1H, d, *J* 8.3, C*H*(OH)), 4.28-4.18 (1H, m, 13-H), 3.67-3.58 (1H, m, 11-H), 3.52 (3H, s, OCH_3), 3.51-3.42 (4H, m, includes 14-H_A; and at δ 3.44 (3H, s, OCH_3), 2.76-2.64 (1H, m, 14-H_B), 2.44-2.35 (1H, m, 12-H_A), 2.16-2.06 (1H, m, 15-H_A), 2.02-1.95 (1H, m, 12-H_B), 1.69-1.58 (1H, m, 15-H_B). **^{13}C NMR** (125 MHz, CD_3OD, 3 × Ar-C_q not observed): δ 159.3 (quinoline 6-C), 155.6 (Ar-C_q), 148.3 (quinoline 2-C), 145.2 (Ar-C_q), 131.1 (quinoline 8-C), 130.8 (Ar-C_q), 129.0 (Ar-C_q), 124.0 (quinoline 7-C), 121.5 (quinoline 3-C), 113.7 (7-C), 112.1 (6-C), 102.9 (quinoline 5-C), 99.0 (4-C), 72.1 (CH(OH)), 67.3 (13-C), 55.6 (2 peaks, 2 × OCH_3), 55.4 (14-C), 33.9 (15-C), 29.0 (11-C), 27.8 (12-C). **HRMS** (ESI): $C_{25}H_{26}O_3N_3$ $[M+H]^+$; calculated: 416.1969, found: 416.1966. $[\alpha]_{20}^{D}$ = +372 (c. 0.1, MeOH).

(*1R*,*11S*,*13R*)-5-Fluoro-13-[(*S*)-hydroxy(6-methoxyquinolin-4-yl)methyl]-1,9-diazatetracyclo[9.2.2.0^{2,10}.0^{3,8}]pentadeca-2(10),3(8),4,6-tetraen-1-ium trifluoroacetate 9l

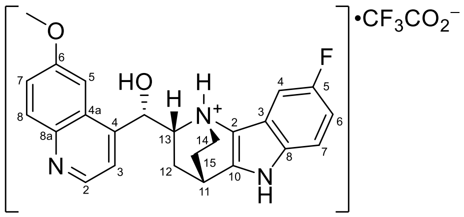

9l

Prepared according to General Procedure B using ketone **5** and 20 mol% $Pd(OAc)_2$ (48 h). Purification by mass-directed preparative HPLC (MeCN in H_2O + 0.1% TFA) gave the *title compound* **9l** (32 mg, 62 μmol, 19%) as a colourless solid. **^{1}H NMR** (500 MHz, CD_3OD, NH^+, NH and OH not observed): δ 8.91 (1H, d, *J* 4.6, quinoline 2-H), 8.17 (1H, d, *J* 8.9, quinoline 8-H), 8.01-7.92 (1H, m, quinolone 3-H), 7.76 (1H, d, *J* 8.9, quinoline 7-H), 7.52-7.45 (2H, m, includes quinoline 5-H; and at δ 7.48: 1H, dd, *J* 8.8, 4.2, 7-H), 7.01-6.89 (2H, m, 4-H and 6-H), 5.69 (1H, s, C*H*(OH)), 4.59 (1H, app. dt, *J* 8.6, 5.9, 13-H), 4.02-3.93 (1H, m, 14-H_A), 3.84-3.79 (4H, m, includes 11-H; and at δ 3.82: 3H, s, OCH_3), 3.14 (1H, td, *J* 11.5, 5.5, 14-H_B), 2.30-2.21 (2H, m, 12-H_A and 15-H_A), 2.12-2.06 (1H, m, 15-H_B), 1.88-1.80 (1H, m, 12-H_B). **^{13}C NMR** (125 MHz, CD_3OD, $CF_3CO_2^-$ not observed): δ 161.6 (quinoline 6-C), 159.7 (d, *J* 235.3, 5-C), 143.4 (quinoline 2-C), 141.4 (Ar-C_q), 132.3 (Ar-C_q), 129.3 (Ar-C_q), 128.1 (quinoline 7-C), 126.1 (quinoline 8-C), 122.0 (quinoline 3-C), 120.3 (d, *J* 11.4, Ar-C_q), 118.2 (Ar-C_q), 118.0 (Ar-C_q), 114.5 (d, *J* 9.1, 7-C), 113.4 (Ar-C_q), 111.2 (d, *J* 26.2, 6-C), 103.4 (quinoline 5-C), 102.7 (d, *J* 24.8, 4-C), 69.6 (CH(OH)), 67.6 (13-C), 57.2 (14-C), 56.8 (OCH_3), 30.1 (15-C), 28.7 (11-C), 25.2 (12-C). **HRMS** (ESI): $C_{24}H_{23}FN_3O_2$ [M+H]$^+$; calculated: 404.1769, found: 404.1765. $[\alpha]_{20}^{D}$ = +69.3 (c. 0.8, MeOH).

(*S*)-[(*11S*,*13R*)-5-Chloro-1,9-diazatetracyclo[9.2.2.0^{2,10}.0^{3,8}]pentadeca-2(10),3(8),4,6-tetraen-13-yl](6-methoxyquinolin-4-yl)methanol 9m

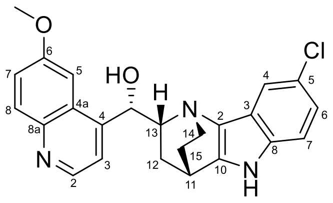

9m

Prepared according to General Procedure B using ketone **5** and 20 mol% $Pd(OAc)_2$ (48 h). Flash column chromatography eluting with 5-20% {50:80:1 CH_2Cl_2:EtOH:NH_4OH} in CH_2Cl_2 gave the *title compound* **9m** (64 mg, 0.16 mmol, 48%) as a colourless solid. **^{1}H NMR** (500 MHz, CD_3OD, NH and OH not observed): δ 8.73 (1H, d, *J* 4.6, quinoline 2-H), 7.99 (1H, d, *J* 9.2, quinoline 8-H), 7.55 (1H, d, *J* 4.6, quinoline 3-H), 7.42 (1H, dd, *J* 9.2, 2.5, quinolone 7-H), 7.33 (1H, d, *J* 8.6, 7-H), 7.10 (1H, d, *J* 2.5, quinoline 5-H), 6.97 (1H, dd, *J* 8.6, 1.9, 6-H), 6.63 (1H, d, *J* 1.9, 4-H), 4.56 (1H, d, *J* 8.9, C*H*(OH)), 3.97-3.89 (1H, m, 13-H), 3.53-3.49 (1H, m, 11-H), 3.47 (3H, s, OCH_3), 3.23-3.16 (1H, m, 14-H_A), 2.49-2.42 (1H, m, 14-H_B), 2.33-2.26 (1H, m, 12-H_A), 2.01-1.94 (1H, m, 15-H_A), 1.90-1.83 (1H, m, 12-H_B), 1.56-1.48 (1H, m, 15-H_B). **^{13}C NMR** (125 MHz, CD_3OD): δ 159.1 (quinoline 6-C), 150.4 (Ar-C_q), 148.2 (Ar-C_q), 145.2 (quinolone 2-C), 144.8 (Ar-C_q), 134.2 (Ar-C_q), 130.9 (quinoline 8-C), 129.1 (Ar-C_q), 126.1 (Ar-C_q), 125.0 (Ar-C_q), 123.8 (quinoline 7-C), 122.1 (Ar-C_q), 121.2 (quinoline 3-C), 121.0 (6-C), 117.2 (4-C), 113.7 (7-C), 103.1 (quinoline 5-C), 73.6 (CH(OH)), 66.4 (13-C), 55.5 (OCH_3), 54.3 (14-C), 35.2 (15-C), 29.3 (11-C), 28.9 (12-C). **HRMS** (ESI): $C_{24}H_{23}{}^{35}ClN_3O_2$ $[M+H]^+$; calculated: 420.1473, found: 420.1476. $[\alpha]_{20}^{D}$ = +158 (c. 1.0, MeOH).

(*1R*,*11S*,*13R*)-5-Bromo-13-[(*S*)-hydroxy(6-methoxyquinolin-4-yl)methyl]-1,9-diazatetracyclo[9.2.2.0^{2,10}.0^{3,8}]pentadeca-2(10),3(8),4,6-tetraen-1-ium trifluoroacetate 9n

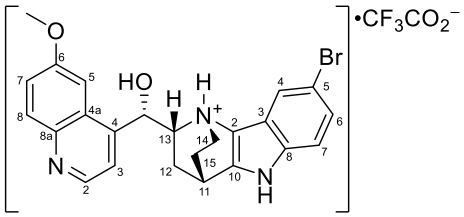

9n

Prepared according to General Procedure B using ketone **5** and 20 mol% Pd(OAc)$_2$ (48 h). Flash column chromatography eluting with 1:9 to 1:4 ({50:80:1 CH_2Cl_2:EtOH:NH_4OH}/CH_2Cl_2) followed by mass-directed preparative HPLC (MeCN in H_2O + 0.1% TFA; Rt = 10.3 min) gave the *title compound* **9n** (16 mg, 27 μmol, 8%) as a yellow solid. **^{1}H NMR** (500 MHz, CD_3OD, NH^+, NH and OH not observed): δ 8.90 (1H, d, *J* 5.3, quinoline 2-H), 8.16 (1H, d, *J* 9.3, quinoline 8-H), 7.97 (1H, d, *J* 5.3, quinoline 3-H), 7.75 (1H, dd, *J* 9.2, 2.5, quinoline 7-H), 7.43 (1H, d, *J* 8.7, indole 7-H), 7.38 (1H, d, *J* 2.4, quinoline 5-H), 7.30 (1H, dd, *J* 8.7, 1.8, indole 6-H), 7.27 (1H, d, *J* 1.8, indole 4-H), 5.58 (1H, d, *J* 5.8, C*H*(OH)), 4.57 (1H, dt, *J* 8.6, 5.8, 13-H), 4.00-3.91 (1H, m, 14-H_A), 3.85-3.79 (1H, m, 11-H), 3.72 (3H, s, OCH_3), 3.16-3.08 (1H, m, 14-H_B), 2.34-2.22 (2H, m, 12-H_A and 15-H_A), 2.13-2.06 (1H, m, 12-H_B), 1.90-1.78 (1H, m, 15-H_B). **^{13}C NMR** (125 MHz, CD_3OD, *C*$F_3CO_2^-$ not observed): δ 162.6 (q, J_{CF} 35.4, CF_3*C*O_2^-), 161.4 (quinoline 6-C), 154.2 (Ar-C_q), 143.9 (quinoline 2-C), 141.1 (Ar-C_q), 138.4 (Ar-C_q), 134.4 (Ar-C_q), 129.2 (Ar-C_q), 127.9 (quinoline 8-C), 126.2 (quinolone 7-C), 125.8 (indole 6-C), 121.9 (quinoline 3-C), 121.6 (Ar-C_q), 120.1 (indole 4-C), 117.0 (Ar-C_q), 115.0 (indole 7-C), 114.6 (Ar-C_q), 103.1 (quinoline 5-C), 69.5 (CH(OH)), 67.9 (13-C), 57.0 (14-C), 56.6 (OCH_3), 30.5 (12-C), 28.6 (11-C), 25.4 (15-C). **HRMS** (ESI): $C_{24}H_{23}BrN_3O_2$ $[M+H]^+$; calculated: 466.0948, found: 466.0945. $[\alpha]_{20}^{D}$ = +127 (c. 0.1, MeOH).

(*1R*,*11S*,*13R*)-13-[(*S*)-Hydroxy(6-methoxyquinolin-4-yl)methyl]-6-methyl-1,9-diazatetracyclo[9.2.2.0^{2,10}.0^{3,8}]pentadeca-2(10),3(8),4,6-tetraen-1-ium trifluoroacetate 9o

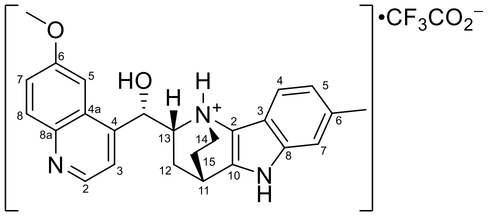

9o

Prepared according to General Procedure B using ketone **5** and 30 mol% $Pd(OAc)_2$ (72 h). Flash column chromatography eluting with 1:9 to 1:4 ({50:80:1 CH_2Cl_2:EtOH:NH_4OH}/CH_2Cl_2) followed by mass-directed preparative HPLC (MeCN in H_2O + 0.1% TFA; Rt = 9.5 min) gave the *title compound* **9o** (21 mg, 40 μmol, 12%) as a yellow solid. **^{1}H NMR** (500 MHz, CD_3OD, NH^+, NH and OH not observed): δ 8.82 (1H, d, *J* 5.1, quinoline 2-H), 8.10 (1H, d, *J* 9.3, quinoline 8-H), 7.83 (1H, d, *J* 5.1, quinoline 3-H), 7.64 (1H, dd, *J* 9.3, 2.6, quinoline 7-H), 7.32-7.29 (2 H, m, quinoline 5-H and indole 7-H), 6.96 (1H, d, *J* 8.2, indole 4-H), 6.91 (1H, d, *J* 8.2, indole 5-H), 5.29 (1H, d, *J* 6.8, C*H*(OH)), 4.65-4.60 (1H, m, 13-H), 3.95-3.89 (1H, m, 14-H_A), 3.83-3.79 (1H, m, 11-H), 3.66 (3H, s, OCH_3), 3.11-3.03 (1H, m, 14-H_B), 2.46 (3H, s, CH_3), 2.44-2.38 (1H, m, 12-H_A), 2.31-2.23 (1H, m, 15-H_A), 2.14-2.08 (1H, m, 12-H_B), 1.85-1.78 (1H, m, 15-H_B). **^{13}C NMR** (125 MHz, CD_3OD, $\mathit{C}F_3\mathit{C}O_2^-$ not observed): δ 160.8 (quinoline 6-C), 151.2 (Ar-C_q), 145.3 (quinoline 2-C), 140.6 (Ar-C_q), 138.8 (Ar-C_q), 136.2 (Ar-C_q), 133.3 (Ar-C_q), 129.1 (Ar-C_q), 128.0 (quinoline 8-C), 126.5 (quinoline 7-C), 123.6 (indole 5-C), 122.2 (quinoline 3-C), 117.9, 116.9 (indole 4-C), 113.3 (indole 7-C), 112.5 (Ar-C_q), 103.2 (quinoline 5-C), 70.4 (CH(OH)), 68.4 (13-C), 57.2 (14-C), 56.2 (OCH_3), 31.0 (12-C), 28.5 (11-C), 25.6 (15-C), 21.7 (CH_3). **HRMS** (ESI): $C_{25}H_{26}O_2N_3$ $[M+H]^+$; calculated: 400.2020, found: 400.2020. $[\boldsymbol{\alpha}]_{\mathbf{20}}^{\boldsymbol{D}}$ = +115 (c. 0.1, MeOH).

(*1R*,*11S*,*13R*)-6-Carboxy-13-[(*S*)-hydroxy(6-methoxyquinolin-4-yl)methyl]-1,9-diaza-tetracyclo[9.2.2.0^{2,10}.0^{3,8}]pentadeca-2(10),3(8),4,6-tetraen-1-ium trifluoroacetate 9p

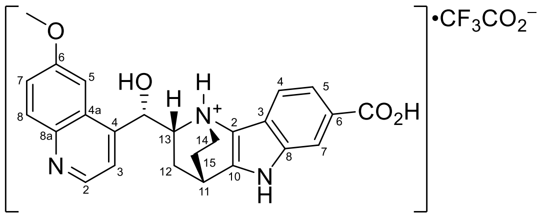

9p

Prepared according to General Procedure B using ketone **5** and 10 mol% $Pd(OAc)_2$ (24 h). Purification by mass-directed preparative HPLC (MeCN in H_2O + 0.1% TFA; Rt = 6.0 min) gave the *title compound* **9p** (20 mg, 47 μmol, 11%) as a yellow solid. **^{1}H NMR** (500 MHz, CD_3OD, CO_2H, NH^+, NH and OH not observed): δ 8.83 (1H, d, *J* 5.2, quinoline 2-H), 8.24 (1H, s, indole 7-H), 8.11 (1H, d, *J* 9.3, quinoline 8-H), 7.82 (1H, d, *J* 5.2, quinoline 3-H), 7.78 (1H, dd, *J* 8.5, 1.3, indole 5-H), 7.67 (1H, dd, *J* 9.3, 2.4, quinoline 7-H), 7.42 (1H, d, *J* 2.4, quinoline 5-H), 7.30 (1H, d, *J* 8.4, indole 4-H), 5.52 (1H, d, *J* 5.5, C*H*(OH)), 4.61-4.55 (1H, m, 13-H), 4.01-3.92 (1H, m, 14-H_A), 3.87-3.83 (1H, m, 11-H), 3.78 (3H, s, OCH_3), 3.18-3.09 (1H, m, 14-H_B), 2.33-2.23 (2H, m, 12 H_A and 15-H_A), 2.13-2.06 (1H, m, 12-H_B), 1.90-1.82 (1H, m, 15-H_B). **^{13}C NMR** (125 MHz, CD_3OD, $\mathit{C}F_3CO_2^-$ not observed): δ 170.5 (CO_2H), 162.9 (q, J_{CF} 33.4, $CF_3\mathit{C}O_2^-$), 162.8 (Ar-C_q), 161.0 (quinoline 6-C), 145.0 (quinoline 2-C), 143.3 (Ar-C_q), 140.0 (Ar-C_q), 135.1 (Ar-C_q), 131.0 (Ar-C_q), 128.9 (Ar-C_q), 127.7 (quinoline 8-C), 126.6 (quinoline 7-C), 125.2 (Ar-C_q), 123.4 (Ar-C_q), 123.1 (indole 5-C), 121.8 (quinoline 3-C), 116.8 (indole 4-C), 115.9 (indole 7-C), 103.2 (quinoline 5-C), 70.0 (CH(OH)), 67.7 (13-C), 57.1 (14-C), 56.5 (OCH_3), 30.1 (12-C), 28.7 (11-C), 25.3 (15-C). **HRMS** (ESI): $C_{25}H_{24}O_4N_3$ $[M+H]^+$; calculated: 430.1761, found: 430.1765. $[\alpha]_{20}^{D}$ = +73 (c. 0.1, MeOH).

Methyl (*11S*,*13R*)-13-[(*S*)-hydroxy(6-methoxyquinolin-4-yl)methyl]-1,9-diazatetracyclo [9.2.2.0^{2,10}.0^{3,8}]pentadeca-2(10),3(8),4,6-tetraene-6-carboxylate 9q

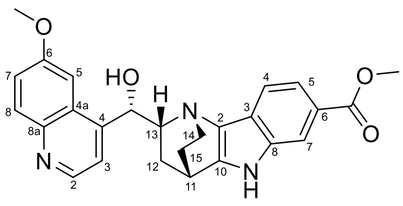

9q

Prepared according to General Procedure B using ketone **5** and 30 mol% $Pd(OAc)_2$ (72 h). Flash column chromatography eluting with 1:9 to 1:4 ({50:80:1 CH_2Cl_2:EtOH:NH_4OH}/CH_2Cl_2) gave the *title compound* **9q** (11 mg, 24 μmol, 7%) as a yellow solid. **^{1}H NMR** (500 MHz, CD_3OD, NH and OH not observed): δ 8.72 (1H, d, *J* 4.6, quinoline 2-H), 8.13 (1H, d, *J* 1.4, indole 7-H), 7.98 (1H, d, *J* 9.2, quinoline 8-H), 7.55 (1H, dd, *J* 8.4, 1.4, indole 5-H), 7.52 (1H, d, *J* 4.6, quinoline 3-H), 7.40 (1H, dd, *J* 9.2, 2.7, quinoline 7-H), 7.18 (1H, d, *J* 2.7, quinoline 5-H), 6.83 (1H, d, *J* 8.3, indole 4-H), 4.55 (1H, d, *J* 8.7, C*H*(OH)), 3.97-3.93 (1H, m, 13-H), 3.92 (3H, s, $ArOCH_3$), 3.56-3.53 (1H, m, 11-H), 3.47-3.45 (3H, s, CO_2CH_3), 3.25-3.18 (1H, m, 14-H_A), 2.52-2.44 (1H, m, 14-H_B), 2.33-2.27 (1H, m, 12-H_A), 2.03-1.97 (1H, m, 15-H_A), 1.92-1.86 (1H, m, 12-H_B), 1.57-1.50 (1H, m 15-H_B). **^{13}C NMR** (125 MHz, CD_3OD): δ 170.1 (*C*O_2CH_3), 159.0 (quinoline 6-C), 150.2 (Ar-C_q), 148.2 (Ar-C_q), 147.7 (quinoline 2-C), 145.2 (Ar-C_q), 135.1 (Ar-C_q), 131.1 (quinoline 8-H), 129.1 (Ar-C_q), 127.5 (Ar-C_q), 123.5 (quinoline 7-C), 123.2 (Ar-C_q), 122.3 (Ar-C_q), 121.4 (indole 5-H or quinoline 3-H), 121.4 (indole 5-H or quinoline 3-H), 117.0 (indole 4-C), 115.1 (indole 7-C), 103.4 (quinoline 5-C), 74.0 (CH(OH)), 66.2 (13-C), 55.6 ($ArOCH_3$), 54.2 (14-C), 52.3 (CO_2*C*H_3), 34.9 (12-C), 29.4 (11-C), 28.7 (15-C). **HRMS** (ESI): $C_{26}H_{26}O_4N_3$ $[M+H]^+$; calculated: 444.1918, found: 444.1917. $[\alpha]_{20}^{D}$ = +94 (c. 0.1, MeOH).

(*S*)-(6-Methoxyquinolin-4-yl)[(*11S*,*13R*)-6-(trifluoromethyl)-1,9-diazatetracyclo [9.2.2.0^{2,10}.0^{3,8}]pentadeca-2(10),3(8),4,6-tetraen-13-yl]methanol 9r

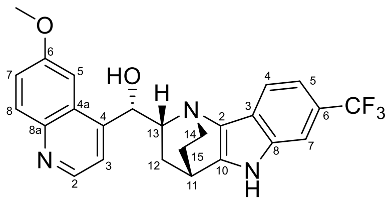

9r

Prepared according to General Procedure B using ketone **5** and 20 mol% Pd(OAc)$_2$ (48 h). Flash column chromatography eluting with 1:9 to 1:4 ({50:80:1 CH_2Cl_2:EtOH:NH_4OH}/CH_2Cl_2) gave the *title compound* **9r** (34 mg, 76 μmol, 25%) as a yellow solid. **^{1}H NMR** (500 MHz, CD_3OD, NH and OH not observed): δ 8.74 (1H, d, *J* 4.7, quinoline 2-H), 7.99 (1H, d, *J* 9.3, quinoline 8-H), 7.70 (1H, s, indole 7-H), 7.56 (1H, d, *J* 4.7, quinoline 3-H), 7.41 (1H, dd, *J* 9.3, 2.6, quinoline 7-H), 7.10-7.05 (2H, m, quinoline 5-H and indole 5-H), 6.84 (1H, d, *J* 8.4, indole 4-H), 4.53 (1H, d, *J* 8.9, C*H*(OH)), 3.98-3.90 (1H, m, 13-H), 3.58-3.53 (1H, m, 11-H), 3.39 (3H, s, OCH_3), 3.27-3.18 (1H, m, 14-H_A), 2.53-2.43 (1H, m, 14-H_B), 2.36-2.28 (1H, m, 12-H_A), 2.04-1.97 (1H, m, 15-H_A), 1.93-1.86 (1H, m, 12-H_B), 1.58-1.49 (1H, m, 15-H_B). **^{13}C NMR** (125 MHz, CD_3OD): δ 159.0 (quinoline 6-C), 150.3 (Ar-C_q), 148.3 (quinoline 2-C), 146.6 (Ar-C_q), 145.2 (Ar-C_q), 134.6 (Ar-C_q), 131.1 (quinoline 8-C), 129.1 (Ar-C_q), 127.0 (q, J_{CF} 270.5, CF_3), 126.2 (Ar-C_q), 123.6 (quinoline 7-C), 122.8 (Ar-C_q), 122.7 (q, J_{CF} 31.2, indole 6-C), 121.3 (quinoline 3-C), 117.9 (indole 4-C), 116.6 (q, J_{CF} 3.5, indole 5-C), 110.0 (q, J_{CF} 4.3, indole 7-C), 103.2 (quinoline 5-C), 73.7 (CH(OH)), 66.3 (13-C), 55.5 (OCH_3), 54.2 (14-C), 35.1 (12-C), 29.3 (11-C), 28.8 (15-C). **HRMS** (ESI): $C_{25}H_{23}O_2N_3F$ [M+H]$^+$; calculated: 454.1737, found: 454.1737. $[\alpha]_{20}^{D}$ = +129 (c. 0.1, MeOH).

(*S*)-[(*11S*,*13R*)-6-Chloro-1,9-diazatetracyclo[9.2.2.0^{2,10}.0^{3,8}]pentadeca-2(10),3(8),4,6-tetraen-13-yl](6-methoxyquinolin-4-yl)methanol 9s

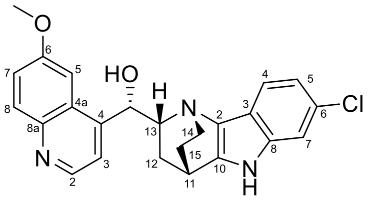

9s

Prepared according to General Procedure B using ketone **5** and 20 mol% $Pd(OAc)_2$ (48 h). Flash column chromatography eluting with 5-20% {50:80:1 CH_2Cl_2:EtOH:NH_4OH} in CH_2Cl_2 gave the *title compound* **9s** (35 mg, 83 μmol, 26%) as a pale brown solid. **^{1}H NMR** (700 MHz, CD_3OD, NH and OH not observed): δ 8.72 (1H, d, *J* 4.5, quinoline 2-H), 7.98 (1H, d, *J* 9.2, quinoline 8-H), 7.53 (1H, d, *J* 4.5, quinoline 3-H), 7.40 (1H, dd, *J* 9.2, 2.6, quinoline 7-H), 7.40 (1H, d, *J* 1.8, 7-H), 7.14 (1H, s, quinoline 5-H), 6.81 (1H, dd, *J* 8.4, 1.8, 5-H), 6.69 (1H, d, *J* 8.4, 4-H), 4.53 (1H, d, *J* 8.9, C*H*(OH)), 3.96-3.86 (1H, m, 13-H), 3.52-3.49 (1H, m, 11-H), 3.48 (3H, s, OCH_3), 3.22-3.14 (1H, m, 14-H_A), 2.51-2.42 (1H, m, 14-H_B), 2.33-2.23 (1H, m, 12-H_A), 2.02-1.94 (1H, m, 15-H_A), 1.91-1.83 (1H, m, 12-H_B), 1.54-1.47 (1H, m, 15-H_B). **^{13}C NMR** (175 MHz, CD_3OD): δ 159.0 (quinoline 6-C), 150.3 (Ar-C_q), 148.3 (quinoline 2-C), 145.3 (Ar-C_q), 144.0 (Ar-C_q), 136.2 (Ar-C_q), 131.1 (quinoline 8-C), 129.1 (Ar-C_q), 126.7 (Ar-C_q), 123.5 (quinoline 7-C), 122.8 (Ar-C_q), 122.6 (Ar-C_q), 121.4 (quinoline 3-C), 120.6 (5-C), 118.5 (4-C), 112.5 (7-C), 103.4 (quinoline 5-C), 73.9 (CH(OH), 66.3 (13-C), 55.5 (OCH_3), 54.2 (14-C), 35.1 (12-C), 29.2 (15-C), 28.9 (11-C). **HRMS** (ESI): $C_{24}H_{23}{}^{37}ClN_3O_2$ $[M+H]^+$; calculated: 422.1444, found: 422.1444. $[\boldsymbol{\alpha}]_{\mathbf{20}}^{\boldsymbol{D}}$ = +142 (c. 0.1, MeOH).

(*1R*,*11S*,*13R*)-6-Bromo-13-[(*S*)-hydroxy(6-methoxyquinolin-4-yl)methyl]-1,9-diaza-tetracyclo[9.2.2.0^{2,10}.0^{3,8}]pentadeca-2(10),3(8),4,6-tetraen-1-ium trifluoroacetate 9t

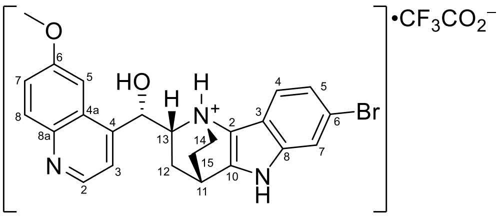

9t

Prepared according to General Procedure B using ketone **5** and 30 mol% $Pd(OAc)_2$ (72 h). Flash column chromatography eluting with 1:9 to 1:4 ({50:80:1 CH_2Cl_2:EtOH:NH_4OH}/CH_2Cl_2) followed by mass-directed preparative HPLC (MeCN in H_2O + 0.1% TFA; Rt = 10.6 min) gave the *title compound* **9t** (16 mg, 27 µmol, 9%) as a yellow solid. **^{1}H NMR** (600 MHz, CD_3OD, NH^+, NH and OH not observed): δ 8.89 (1H, d, *J* 5.3, quinoline 2-H), 8.15 (1H, d, *J* 9.4, quinoline 8-H), 7.92 (1H, d, *J* 5.3, quinolone 3-H), 7.73 (1H, dd, *J* 9.3, 2.6, quinoline 7-H), 7.68 (1H, d, *J* 1.3, indole 7-H), 7.47 (1H, d, *J* 2.6, quinoline 5-H), 7.23-7.16 (2 H, m, indole 4-H and indole 5-H), 5.62 (1H, d, *J* 5.5, C*H*(OH)), 4.61-4.55 (1H, m, 13-H), 4.00-3.92 (1H, m, 14-H_A), 3.81 (4 H, s, OCH_3 and 11-H), 3.16-3.10 (1H, m ,14-H_B), 2.29-2.18 (2 H, m, 12-H_A and 15-H_A), 2.13-2.05 (1H, m, 12-H_B), 1.89-1.79 (1H, m, 15-H_B). **^{13}C NMR** (125 MHz, CD_3OD, $\mathit{C}F_3CO_2^-$ not observed): δ 162.61 (q, J_{CF} 35.7, $CF_3\mathit{C}O_2^-$), 161.4 (quinoline 6-C), 154.1 (Ar-C_q), 143.9 (quinoline 2-C), 140.4 (Ar-C_q), 138.3 (Ar-C_q), 136.5 (Ar-C_q), 129.2 (Ar-C_q), 127.7 (quinoline 7-C), 126.3 (quinoline 8-C), 124.9 (indole 4-C or indole 5-C), 122.0 (quinoline 3-C), 119.2 (indole 4-C or indole 5-C), 118.8 (Ar-C_q), 116.3 (indole 7-C), 116.1 (Ar-C_q), 113.5 (Ar-C_q), 103.3 (quinoline 5-C), 69.7 (CH(OH)), 67.6 (13-C), 57.1 (14-C), 56.6 (OCH_3), 30.1 (12-C), 28.6 (11-C), 25.2 (15-C). **HRMS** (ESI): $C_{24}H_{23}O_2N_3{}^{79}Br$ $[M+H]^+$; calculated: 464.0968, found: 464.0967; $C_{24}H_{23}O_2N_3{}^{81}Br$ $[M+H]^+$; calculated: 466.0948, found: 466.0946. $[\alpha]_{20}^{D}$ = +69 (c. 0.1, MeOH).

(*1R*,*11S*,*13R*)-13-[(*S*)-Hydroxy(6-methoxyquinolin-4-yl)methyl]-7-methoxy-1,9-diaza-tetracyclo[9.2.2.0^{2,10}.0^{3,8}]pentadeca-2(10),3(8),4,6-tetraen-1-ium trifluoroacetate 9u

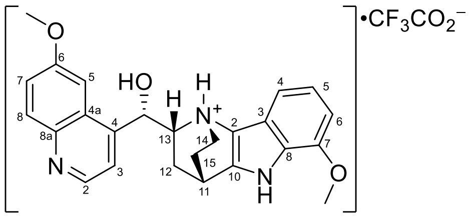

9u

Prepared according to General Procedure B using ketone **5** and 30 mol% $Pd(OAc)_2$ (72 h). Flash column chromatography eluting with 1:9 to 1:4 ({50:80:1 CH_2Cl_2:EtOH:NH_4OH}/CH_2Cl_2) followed by mass-directed preparative HPLC (MeCN in H_2O + 0.1% TFA; Rt = 9 min) gave the *title compound* **9u** (27 mg, 49 μmol, 16%) as a yellow solid. **^{1}H NMR** (500 MHz, CD_3OD, NH^+, NH and OH not observed): δ 8.85 (1H, d, *J* 5.3, quinoline 2-H), 8.13 (1H, d, *J* 9.3, quinoline 8-H), 7.88 (1H, d, *J* 5.3, quinoline 3-H), 7.69 (1H, dd, *J* 9.3, 2.5, quinoline 7-H), 7.42 (1H, d, *J* 2.5, quinoline 5-H), 7.01 (1H, t, *J* 7.9, indole 5-H), 6.77 (2H, d, *J* 7.9, indole 4-H and indole 6-H), 5.41 (1H, d, *J* 6.4, C*H*(OH)), 4.66-4.59 (1H, m, 13-H), 4.00 (3H, s, OCH_3), 3.97-3.91 (1H, m, 14-H_A), 3.83-3.80 (1H, m, 11-H), 3.74 (3 H, s, OCH_3), 3.13-3.06 (1H, m, 14-H_B), 2.41-2.35 (1H, m, 12-H_A), 2.31-2.23 (1H, m, 15-H_A), 2.13-2.06 (1H, m, 12-H_B), 1.85-1.78 (1H, m, 15-H_B). **^{13}C NMR** (125 MHz, CD_3OD, $CF_3CO_2^-$ not observed): δ 162.5 (q, J_{CF} 35.1, $CF_3CO_2^-$), 161.1 (quinoline 2-C), 152.7 (Ar-C_q), 148.4 (Ar-C_q), 144.4 (quinoline 2-C), 139.2 (Ar-C_q), 138.9 (Ar-C_q), 129.2 (Ar-C_q), 127.2 (quinoline 7-C), 126.9 (quinoline 8-C), 125.9 (Ar-C_q), 122.7 (indole 5-C), 122.3 (quinoline 3-C), 121.1 (Ar-C_q), 113.1 (Ar-C_q), 109.9 (indole 4-C or indole 6-C), 103.4 (quinoline 5-C), 103.4 (indole 4-C or indole 6-C), 70.3 (CH(OH)), 68.2 (13-C), 57.2 (14-C), 56.5 (OCH_3), 56.0 (OCH_3), 30.7 (12-C), 28.4 (11-C), 25.5 (15-C). **HRMS** (ESI): $C_{25}H_{26}O_3N_3$ [M+H]$^+$; calculated: 416.1969, found: 416.1967. $[\alpha]_D^{20}$ = +98 (c. 0.1, MeOH).

6.3.2.2 Preparation of the quinidine-derived azaindoles

(*1R*,*11S*,*13R*)-13-[(*S*)-Hydroxy(6-methoxyquinolin-4-yl)methyl]-1,6,9-triazatetracyclo[9.2.2.0^{2,10}.0^{3,8}]pentadeca-2(10),3(8),4,6-tetraen-1-ium trifluoroacetate 9v

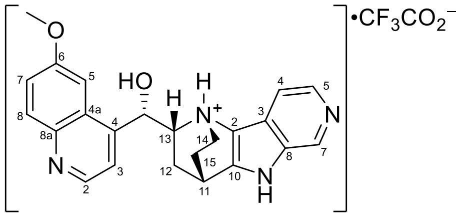

9v

Prepared according to General Procedure B using ketone **5** and 20 mol% $Pd(OAc)_2$ (48 h). Purification by mass-directed preparative HPLC (MeCN in H_2O + 0.1% TFA) gave the *title compound* **9v** (80 mg, 0.16 mmol, 50%) as a colourless solid. **^{1}H NMR** (700 MHz, CD_3OD, NH^+ and OH not observed): δ 9.05 (1H, s, 7-H), 8.95 (1H, d, *J* 5.5, quinoline 2-H), 8.20 (1H, d, *J* 9.2, quinoline 8-H), 8.13 (1H, d, *J* 6.5, 5-H), 7.92 (1H, d, *J* 5.5, quinoline 3-H), 7.78 (1H, dd, *J* 9.2, 2.6, quinoline 7-H), 7.60 (1H, d, *J* 2.6, quinoline 5-H), 7.54 (1H, d, *J* 6.5, 4-H), 5.26 (1H, d, *J* 5.9, CH(OH)), 4.11 (1H, app. dd, *J* 14.5, 6.2, 13-H), 3.87 (3H, s, OCH_3), 3.82-3.79 (1H, m, 11-H), 3.57-3.52 (1H, m, 14-H_A), 2.76-2.69 (1H, m, 14-H_B), 2.27-2.22 (1H, m, 15-H_A), 2.18-2.13 (1H, m, 12-H_A), 2.02-1.97 (1H, m, 15-H_B), 1.72-1.66 (1H, m, 12-H_B). **^{13}C NMR** (175 MHz, CD_3OD): δ 162.6 (q, *J* 35.7, $CF_3\mathit{C}O_2^-$), 161.4 (quinoline 6-C), 158.9 (Ar-C_q), 158.5 (Ar-C_q), 143.1 (quinoline 2-C), 137.1 (Ar-C_q), 131.1 (Ar-C_q), 131.0 (Ar-C_q), 129.8 (5-C or Ar-C_q), 129.5 (5-C or Ar-C_q), 128.2 (7-C), 127.9 (quinolone 7-C), 125.4 (quinoline 8-C), 123.5 (Ar-C_q), 121.5 (quinoline 3-C), 117.9 (q, *J* 292, $\mathit{C}F_3CO_2^-$), 113.6 (4-C), 104.1 (quinoline 5-C), 72.4 (CH(OH)), 65.7 (13-C), 56.8 (OCH_3), 54.6 (14-C), 31.9 (15-C), 29.9 (11-C), 26.8 (12-C). **HRMS** (ESI): $C_{23}H_{23}N_4O_2$ $[M+H]^+$; calculated: 387.1816, found: 387.1817. $[\alpha]_{20}^{D}$ = +44 (c. 1.5, MeOH).

(*1R*,*11S*,*13R*)-13-[(*S*)-Hydroxy(6-methoxyquinolin-4-yl)methyl]-1,7,9-triazatetracyclo[9.2.2.0^{2,10}.0^{3,8}]pentadeca-2(10),3(8),4,6-tetraen-1-ium trifluoroacetate 9w

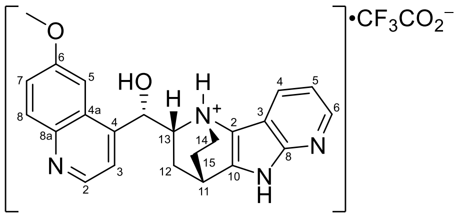

9w

Prepared according to General Procedure B using ketone **5** and 20 mol% $Pd(OAc)_2$ (48 h). Purification by mass-directed preparative HPLC (MeCN in H_2O + 0.1% TFA) gave the *title compound* **9w** (28 mg, 56 μmol, 17%) as a pale yellow solid. **^{1}H NMR** (700 MHz, CD_3OD, NH^+ and OH not observed): δ 8.91 (1H, d, *J* 5.4, quinoline 2-H), 8.30 (1H, d, *J* 3.9, 6-H), 8.18 (1H, d, *J* 9.2, quinoline 8-H), 7.95 (1H, d, *J* 5.4, quinoline 3-H), 7.91 (1H, d, *J* 7.9, 4-H), 7.78 (1H, dd, *J* 9.2, 2.5, quinoline 7-H), 7.65-7.58 (1H, m, quinoline 5-H), 7.25 (1H, dd, *J* 7.9, 4.9, 5-H), 5.83 (1H, br. s, C*H*(OH)), 4.55-4.50 (1H, m, 13-H), 4.13-4.08 (1H, m, OH), 4.00-3.94 (1H, m, 14-H_A), 3.93 (3H, s, OCH_3), 3.86-3.82 (1H, m, 11-H), 3.16 (1H, td, *J* 11.3, 5.5, 14-H_B), 2.28-2.22 (1H, m, 15-H_A), 2.20-2.13 (1H, m, 12-H_A), 2.11-2.05 (1H, m, 12-H_B), 1.92-1.85 (1H, m, 15-H_B). **^{13}C NMR** (125 MHz, CD_3OD, 3 × Ar-C_q not observed): δ 162.2 (q, *J* 36.4, $CF_3\mathit{C}O_2^-$), 161.7 (quinoline 6-C), 146.3 (Ar-C_q), 143.5 (quinoline 2-C), 142.7 (6-C), 141.4 (Ar-C_q), 129.2 (Ar-C_q), 127.9 (quinoline 7-C), 127.2 (4-C), 125.9 (quinoline 8-C), 121.7 (quinoline 3-C), 118.0 (5-C), 117.8 (q, 288.7, $\mathit{C}F_3CO_2^-$), 103.4 (quinoline 5-C), 69.8 (CH(OH)), 67.1 (13-C), 56.9 (2 peaks, 14-C and OCH_3), 29.9 (12-C), 28.5 (11-C), 25.2 (15-C). **HRMS** (ESI): $C_{23}H_{23}N_4O_2$ $[M+H]^+$; calculated: 387.1816, found: 387.1818. $[\alpha]_{20}^{D}$ = +64 (c. 0.2, MeOH).

(*S*)-(6-Methoxyquinolin-4-yl)[(*11S*,*13R*)-5-methyl-1,7,9-triazatetracyclo[9.2.2.0^{2,10}.0^{3,8}]pentadeca-2(10),3(8),4,6-tetraen-13-yl]methanol 9w-b

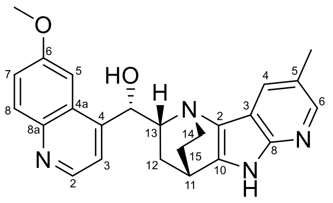

9w-b

Prepared according to General Procedure B using ketone **5** and 30 mol% $Pd(OAc)_2$ (72 h). Flash column chromatography eluting with 5-35% {50:80:1 CH_2Cl_2:EtOH:NH_4OH} in CH_2Cl_2 gave the *title compound* **9w-b** (65 mg, 0.16 mmol, 51%) as a colourless solid. **^{1}H NMR** (500 MHz, CD_3OD, NH and OH not observed): δ 8.70 (1H, d, *J* 4.6, quinoline 2-H), 7.94 (1H, d, *J* 9.2, quinoline 8-H), 7.85 (1H, d, *J* 1.3, 6-C), 7.56 (1H, d, *J* 4.6, quinoline 3-H), 7.35 (1H, dd, *J* 9.2, 2.3, quinoline 7-H), 6.90 (1H, d, *J* 2.3, quinoline 5-H), 6.65 (1H, s, 4-H), 4.51 (1H, d, *J* 9.0, CH(OH)), 3.88-3.79 (1H, m, 13-H), 3.51-3.45 (1H, m, 11-H), 3.28 (3H, s, OCH_3), 3.17-3.10 (1H, m, 14-H_A), 2.39 (1H, ddd, *J* 12.3, 10.9, 5.1, 14-H_B), 2.32-2.26 (1H, m, 15-H_A), 2.17 (3H, s, Py-CH_3), 1.97-1.90 (1H, m, 12-H_A), 1.87-1.81 (1H, m, 15-H_B), 1.52-1.43 (1H, m, 12-H_B). **^{13}C NMR** (125 MHz, CD_3OD): δ 158.9 (quinoline 6-C), 150.6 (Ar-C_q), 148.4 (quinoline 2-C), 145.9 (Ar-C_q), 145.2 (Ar-C_q), 144.9 (Ar-C_q), 141.8 (6-C), 131.1 (quinoline 8-C), 129.1 (Ar-C_q), 126.7 (4-C), 126.0 (Ar-C_q), 123.6 (quinoline 7-C), 121.1 (quinoline 3-C), 120.7 (Ar-C_q), 117.3 (Ar-C_q), 103 (quinoline 5-C), 73.0 (CH(OH)), 66.7 (13-C), 55.4 (OCH_3), 54.1 (14-C), 35.5 (15-C), 29.0 (11-C and 12-C), 18.4 (Py-CH_3). **HRMS** (ESI): $C_{24}H_{25}N_4O_2$ $[M+H]^+$; calculated: 401.1972, found: 401.1965. $[\boldsymbol{\alpha}]_{\mathbf{20}}^{\boldsymbol{D}}$ = +353 (c. 0.1, MeOH).\

(*S*)-[(*11S*,*13R*)-5-Fluoro-1,9-diazatetracyclo[9.2.2.0^{2,10}.0^{3,8}]pentadeca-2(10),3(8),4,6-tetra-en-13-yl](6-methoxyquinolin-4-yl)methanol 9w-f

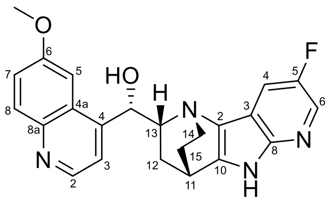

9w-f

Prepared according to General Procedure B using ketone **5** and 30 mol% $Pd(OAc)_2$ (72 h). Flash column chromatography eluting with 1:9 to 1:3 ({50:80:1 CH_2Cl_2:EtOH:NH_4OH}/CH_2Cl_2) gave the *title compound* **9w-f** (49 mg, 121 μmol, 38%) as yellow solid. **^{1}H NMR** (500 MHz, CD_3OD, NH and OH not observed): δ 8.73 (1H, d, *J* 4.6, quinoline 2-H), 8.05-7.97 (2H, m, quinoline 8-H and indole 6-H), 7.54 (1H, d, *J* 4.6, quinoline 3-H), 7.43 (1H, dd, *J* 9.2, 2.7, quinolone 7-H), 7.16 (1H, d, *J* 2.7, quinoline 5-H), 6.85 (1H, dd, *J* 9.3, 2.7, indole 4-H), 4.65 (1H, d, *J* 8.2, CH(OH)), 3.93-3.86 (1H, m, 13-H), 3.59 (3H, s, OCH_3), 3.56-3.48 (1H, m, 11-H), 3.27-3.18 (1H, m, 14-H_A), 2.53-2.45 (1H, m, 14-H_B), 2.31-2.24 (1H, m, 12-H_A), 2.05-1.96 (1H, m, 15-H_A), 1.94-1.88 (1H, m, 12-H_B), 1.64-1.50 (1H, m, 15-H_B). **^{13}C NMR** (125 MHz, CD_3OD): δ 159.1 (quinolone 6-C), 157.0 (d, J_{CF} 240.1, indole 5-C), 150.3 (Ar-C_q), 148.3 (quinoline 2-C), 147.6 (Ar-C_q), 145.1 (Ar-C_q), 144.4 (Ar-C_q), 131.2 (quinoline 8-C), 129.2 (d, J_{CF} 30.4, indole 6-C), 128.9 (Ar-C_q), 123.3 (quinoline 7-C), 121.4 (d, J_{CF} 4.0, indole 8-C) 121.2 (quinoline 3-C), 117.4 (d, J_{CF} 7.0, indole 3-C), 111.6 (d, J_{CF} 21.9, indole 4-C), 103.3 (quinoline 5-C), 73.6 (CH(OH)), 66.2 (13-C), 55.7 (OCH_3), 54.3 (14-C), 34.8 (12-C), 29.1 (11-C), 28.7 (15-C). **HRMS** (ESI): $C_{23}H_{22}O_2N_4F$ $[M+H]^+$; calculated: 405.1721, found: 405.1721. $[\alpha]_{20}^{D}$ = +168 (c. 0.1, MeOH).

(1*R*,11*S*,13*R*)-13-[(*S*)-Hydroxy(6-methoxyquinolin-4-yl)methyl]-6-methyl-1,7,9-triaza-tetracyclo[9.2.2.0^{2,10}.0^{3,8}]pentadeca-2(10),3(8),4,6-tetraen-1-ium trifluoroacetate 9w-k

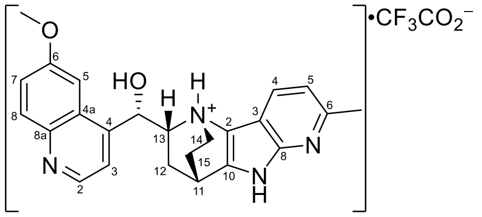

9w-k

Prepared according to General Procedure B using ketone **5** and 30 mol% $Pd(OAc)_2$ (72 h). Flash column chromatography eluting with 1:9 to 1:4 ({50:80:1 CH_2Cl_2:EtOH:NH_4OH}/CH_2Cl_2) followed by mass-directed preparative HPLC (MeCN in H_2O + 0.1% TFA; Rt = 6 min) gave the *title compound* **9w-k** (21 mg, 42 μmol, 13%) as yellow solid. **^{1}H NMR** (500 MHz, CD_3OD, NH^+, NH and OH not observed): δ 8.90 (1H, d, *J* 5.4, quinoline 2-H), 8.16 (1H, d, *J* 9.3, quinoline 8-H), 7.93 (1H, d, *J* 5.4, quinoline 3-H), 7.83 (1H, d, *J* 8.1, indole 4-H), 7.76 (1H, dd, *J* 9.3, 2.4, quinoline 7-H), 7.59 (1H, d, *J* 2.4, quinoline 5-H), 7.16 (1H, d, *J* 8.1, indole 5-H), 5.78 (1H, d, *J* 4.4 C*H*(OH)), 4.52-4.45 (1H, m, 13-H), 3.95-3.92 (1H, m, 14-H_A), 3.91 (3H, s, OCH_3), 3.83-3.79 (1H, m, 11-H), 3.14-3.08 (1H, m, 14-H_B), 2.67 (3H, s, Py-CH_3), 2.26-2.18 (1H, m, 15-H_A), 2.17-2.11 (1H, m, 12-H_A), 2.08-2.02 (1H, m, 12-H_B), 1.88-1.81 (1H, m, 15-H_B). **^{13}C NMR** (125 MHz, CD_3OD, $\mathit{C}F_3CO_2^-$ not observed): δ 162.7 (d, J_{CF} 36.3, $CF_3CO_2^-$), 161.6 (quinoline 6-C), 155.3 (Ar-C_q), 151.6 (Ar-C_q), 145.0 (Ar-C_q), 143.5 (quinoline 2-H), 140.8 (Ar-C_q), 137.6 (Ar-C_q), 129.2 (Ar-C_q), 128.6 (indole 4-C), 128.0 (quinoline 7-C), 125.9 (quinoline 8-C), 121.7 (quinoline 3-C), 118.1 (indole 5-C), 114.0 (Ar-C_q), 113.1 (Ar-C_q), 103.4 (quinoline 5-C), 69.8 (CH(OH)), 67.0 (13-C), 56.8 (OCH_3), 56.6 (14-C), 30.0 (12-C), 28.5 (11-C), 25.4 (15-C), 23.0 (Py-CH_3). **HRMS** (ESI): $C_{24}H_{25}O_2N_4$ $[M+H]^+$; calculated: 401.1972, found: 401.1972. $[\boldsymbol{\alpha}]_{\mathbf{20}}^{\boldsymbol{D}}$ = +52 (c. 0.1, MeOH).

6.3.2.3 Preparation of the quinine-derived indoles

(*R*)-[(*11S*,*13S*)-1,9-Diazatetracyclo[9.2.2.0^{2,10}.0^{3,8}]pentadeca-2(10),3(8),4,6-tetraen-13-yl](6-methoxyquinolin-4-yl)methanol 10a

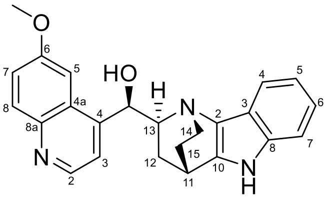

10a

Prepared according to General Procedure B using ketone **6** and 10 mol% $Pd(OAc)_2$ (24 h). Flash column chromatography eluting with 1:9 to 1:4 ({50:80:1 CH_2Cl_2:EtOH:NH_4OH}/CH_2Cl_2) gave the *title compound* **10a** (21 mg, 55 μmol, 17%) as a yellow solid. **^{1}H NMR** (500 MHz, CD_3OD, NH and OH not observed) δ 8.63 (1H, d, *J* 4.7, quinoline 2-H), 7.84 (1H, d, *J* 9.2, quinoline 8-H), 7.75 (1H, d, *J* 4.7, quinoline 3-H), 7.67-7.62 (1H, m, indole 4-H), 7.37 (1H, d, *J* 2.4 Hz, quinoline 5-H), 7.32-7.26 (2H, m, quinoline 7-H and indole 7-H), 7.05-6.98 (2H, m, indole 5-H and 6-H), 6.04 (1H, app. s, C*H*(OH)), 4.26 (1H, ddd, *J* 12.4, 8.9, 3.9 Hz 14-H_A), 3.97 (3H, s, OCH_3), 3.40 (1H, s, 11-H), 3.10-3.04 (1H, m, 13-H), 2.67 (1H, app. td, *J* 11.2, 4.2, 14-H_B), 2.38-2.32 (1H, m, 12-H_A), 2.21-2.13 (1H, m, 15-H_A), 1.68-1.60 (1H, m 15-H_B), 1.18-1.11 (1H, m, 12-H_B). **^{13}C NMR** (125 MHz, CD_3OD) δ 159.7 (quinoline 6-C), 151.0 (Ar-C_q), 148.1 (quinoline 2-C), 144.5 (Ar-C_q), 142.2 (Ar-C_q), 135.6 (Ar-C_q), 131.2 (quinoline 8-C), 127.7 (Ar-C_q), 127.2 (Ar-C_q), 123.7 (quinoline 7-C), 122.0 (Ar-C_q) 121.2 (indole 5-C or indole 6-C), 120.5 (indole 5-C or indole 6-C), 119.3 (quinoline 3-C), 116.7 (indole 4-C), 112.8 (indole 7-C), 101.8 (quinoline 5-C), 71.4 (CH(OH)), 67.1 (13-C), 56.6 (OCH3), 49.2[§] (14-C), 30.3 (15-C), 29.5 (11-C), 27.4 (12-C) ppm. **HRMS** (ESI): $C_{24}H_{24}O_2N_3$ $[M+H]^+$; calculated: 386.1863, found: 386.1863. $[\alpha]^{D}_{20}$ = +76 (c. 0.1, MeOH).

[§] Inferred from HSQC analysis.

(*R*)-(6-Methoxyquinolin-4-yl)[(*11S*,*13S*)-4-methyl-1,9-diazatetracyclo[9.2.2.0^{2,10}.0^{3,8}] pentadeca-2(10),3(8),4,6-tetraen-13-yl]methanol 10b

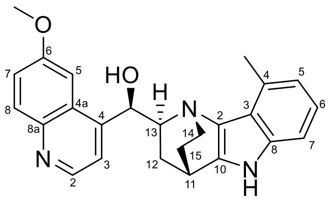

10b

Prepared according to General Procedure B using ketone **6** and 20 mol% Pd(OAc)$_2$ (48 h). Flash column chromatography eluting with 1:9 to 1:4 ({50:80:1 CH_2Cl_2:EtOH:NH_4OH}/CH_2Cl_2) gave the *title compound* **10b** (59 mg, 147 μmol, 46%) as a yellow solid. **^{1}H NMR** (500 MHz, CD_3OD, NH and OH not observed): δ 8.65 (1H, d, *J* 4.6, quinoline 2-H), 7.93 (1H, d, *J* 9.2, quinoline 8-H), 7.71 (1H, d, *J* 4.6, quinoline 3-H), 7.63 (1H, d, *J* 2.6, quinoline 5-H), 7.38 (1H, dd, *J* 9.2, 2.6, quinoline 7-H), 7.08 (1H, d, *J* 8.1, indole 7-H), 6.82 (1H, app. t, *J* 7.6, indole 6-H), 6.62 (1H, d, *J* 7.1, indole 5-H), 5.71 (1H, d, *J* 6.5, C*H*(OH)), 3.92 (3H, s, OCH_3), 3.78-3.69 (1H, m, 14-H_A), 3.39-3.35 (1H, m, 11-H), 3.21-3.15 (1H, m, 13-H), 2.47-2.40 (1H, m, 14-H_B), 2.25 (3H, s, $ArCH_3$), 2.18-2.11 (1H, m, 12-H_A), 2.11-2.03 (1H, m, 15-H_A), 1.69-1.62 (1H, m, 12-H_B), 1.57-1.51 (1H, m, 15-H_B). **^{13}C NMR** (125 MHz, CD_3OD): δ 159.3 (quinoline 6-C), 151.6 (Ar-C_q), 148.1 (quinoline 2-C), 144.9 (Ar-C_q), 142.1 (Ar-C_q), 135.3 (Ar-C_q), 131.1 (quinoline 8-C), 130.3 (Ar-C_q), 128.9 (Ar-C_q), 128.8 (Ar-C_q), 123.0 (quinoline 7-C), 122.0 (Ar-C_q), 120.8 (indole 5-C), 120,7 (indole 6-C), 120.6 (quinoline 3-C), 110.0 (indole 7-C), 103.4 (quinoline 5-C), 73.5 (CH(OH)), 67.7 (13-C), 56.2 (OCH_3), 47.2 (14-C), 31.7 (12-C), 31.2 (15-C), 29.5 (11-C), 18.7 (CH_3). **HRMS** (ESI): $C_{25}H_{26}O_2N_3$ [M+H]$^+$; calculated: 400.2020, found: 400.2020. $[\alpha]_{20}^{D}$ = +48 (c. 0.1, MeOH).

Methyl (*11S*,*13S*)-13-[(*R*)-hydroxy(6-methoxyquinolin-4-yl)methyl]-1,9-diazatetracyclo [9.2.2.0^{2,10}.0^{3,8}]pentadeca-2(10),3(8),4,6-tetraene-4-carboxylate 10c

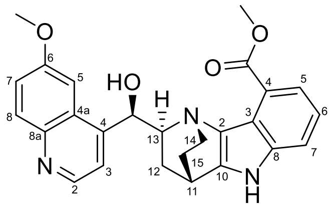

10c

Prepared according to General Procedure B using ketone **6** and 30 mol% $Pd(OAc)_2$ (72 h). Flash column chromatography eluting with 1:9 to 1:4 ({50:80:1 CH_2Cl_2:EtOH:NH_4OH}/CH_2Cl_2) gave the *title compound* **10c** (17 mg, 38 μmol, 12%) as a yellow solid. **^{1}H NMR** (500 MHz, CD_3OD, NH and OH not observed): δ 8.64 (1H, d, *J* 4.6, quinoline 2-H), 8.06 (1H, app. s, indole 5-H), 7.86 (1H, d, *J* 9.2, quinoline 8-H), 7.76 (1H, d, *J* 4.6, quinoline 3-H), 7.72 (1H, dd, *J* 8.4, 1.4, indole 6-H), 7.67 (1H, d, *J* 8.4, indole 7-H), 7.39 (1H, d, *J* 2.7, quinoline 5-H), 7.31 (1H, dd, *J* 9.2, 2.7, quinoline 7-H), 6.02 (1H, s, C*H*(OH)), 4.28-4.22 (1H, m, 14-H_A), 3.98 (3H, s, CO_2CH_3 or OCH_3), 3.88 (3H, s, CO_2CH_3 or OCH_3), 3.45 (1H, s, 11-H), 3.08-3.02 (1H, m, 13-H), 2.65 (1H, app. td, *J* 11.1, 4.0, 14-H_B), 2.41-2.35 (1H, m, 12-H_A), 2.24-2.17 (1H, m, 15-H_A), 1.68-1.61 (1H, m, 12-H_B), 1.22-1.15 (1H, m, 12-H_B). **^{13}C NMR** (125 MHz, CD_3OD): δ 170.0 (CO_2CH_3), 159.7 (quinolone 6-C), 151.0 (quinoline 2-C), 148.2 (Ar-C_q), 147.4 (Ar-C_q), 144.6 (Ar-C_q), 134.7 (Ar-C_q), 131.3 (quinoline 8-C), 128.3 (Ar-C_q), 127.8 (Ar-C_q), 125.5 (Ar-C_q), 123.6 (quinolone 7-C), 122.3 (Ar-C_q), 121.8 (indole 6-C), 119.4 (quinoline 3-C), 116.1 (indole 7-C), 115.3 (indole 5-C), 101.9 (quinoline 5-C), 71.6 (CH(OH)), 66.8 (13-C), 56.6 (CO_2CH_3 or OCH_3), 52.3 (CO_2CH_3 or OCH_3), 48.4§ (14-C), 30.2 (15-C), 29.7 (11-C), 27.4 (12-C). **HRMS** (ESI): $C_{26}H_{26}O_4N_3$ $[M+H]^+$; calculated: 444.1918, found: 444.1917. $[\alpha]_{20}^{D}$ = +64 (c. 0.1, MeOH).

§ Inferred from HSQC analysis.

(*R*)-[(*11S*,*13S*)-4-Chloro-1,9-diazatetracyclo[9.2.2.0^{2,10}.0^{3,8}]pentadeca-2(10),3(8),4,6-tetra-en-13-yl](6-methoxyquinolin-4-yl)methanol 10d

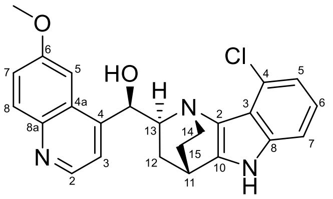

10d

Prepared according to General Procedure B using ketone **6** and 10 mol% $Pd(OAc)_2$ (24 h). Flash column chromatography eluting with 1:9 to 1:4 ({50:80:1 CH_2Cl_2:EtOH:NH_4OH}/CH_2Cl_2) gave the *title compound* **10d** (14 mg, 34 μmol, 10%) as a yellow solid. **^{1}H NMR** (500 MHz, CD_3OD, NH and OH not observed): δ 8.65 (1H, d, *J* 4.6, quinoline 2-H), 7.87 (1H, d, *J* 9.3, quinoline 8-H), 7.78-7.73 (2 H, m, quinoline 3-H, indole 6-H), 7.65-7.61 (1H, m, indole 5-H), 7.41 (1H, d, *J* 2.7, quinoline 5-C), 7.32 (1H, dd, *J* 9.3, 2.7, quinoline 7-H), 7.28 (1H, d, *J* 8.3, indole 7-H), 6.01 (1H, app. s, C*H*(OH)), 4.27-4.19 (1H, m, 14-H_A), 3.98 (3 H, s, OCH_3), 3.47-3.43 (1H, m, 11-H), 3.07-3.02 (1H, m, 13-H), 2.66-2.59 (1H, m, 14-H_B), 2.41-2.35 (1H, m, 12-H_A), 2.24-2.16 (1H, m, 15-H_A), 1.68-1.60 (1H, m, 15-H_B), 1.24-1.17 (1H, m, 12-H_B). **^{13}C NMR** (125 MHz, CD_3OD): δ 159.7 (quinoline 6-C), 151.1 (Ar-C_q), 148.2 (quinoline 2-C), 146.4 (Ar-C_q), 144.6 (Ar-C_q), 134.3 (Ar-C_q), 131.3 (quinoline 8-C), 128.4 (Ar-C_q), 127.8 (Ar-C_q), 124.4 (Ar-C_q), 123.6 (quinoline 7-C), 122.7 (Ar-C_q), 119.4 (quinoline 3-C), 117.0 (indole 6-C), 117.0 (indole 7-C), 110.2 (indole 5-C), 102.0 (quinoline 5-C), 71.7 (CH(OH)), 66.8 (13-C), 56.6 (OCH_3), 48.4 (14-C), 30.4 (15-C), 29.7 (11-C), 27.7 (12-C). **HRMS** (ESI): $C_{24}H_{23}O_2N_3{}^{35}Cl$ $[M+H]^+$; calculated: 420.1473, found: 420.1471. $C_{24}H_{23}O_2N_3{}^{37}Cl$ $[M+H]^+$; calculated: 422.1443, found: 422.1443. $[\alpha]_{20}^{D}$ = +80 (c. 0.1, MeOH).

(*R*)-(6-Methoxyquinolin-4-yl)[(*11S*,*13S*)-5-methyl-1,9-diazatetracyclo[9.2.2.0^{2,10}.0^{3,8}] pentadeca-2(10),3(8),4,6-tetraen-13-yl]methanol 10e

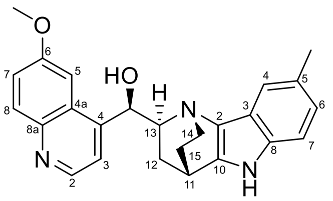

10e

Prepared according to General Procedure B using ketone **6** and 10 mol% Pd(OAc)$_2$ (24 h). Flash column chromatography eluting with 1:9 to 1:4 ({50:80:1 CH_2Cl_2:EtOH:NH_4OH}/CH_2Cl_2) gave the *title compound* **10e** (21 mg, 53 µmol, 17%) as a yellow solid. **^{1}H NMR** (500 MHz, CD_3OD): δ 8.65 (1H, d, *J* 4.6 Hz, quinoline 2-H), 7.87 (1H, d, *J* 9.3 Hz, quinolone 8-H), 7.76 (1H, d, *J* 4.7 Hz, quinoline 3-H), 7.44 (1H, s, indole 4-H), 7.39 (1H, d, *J* 2.6 Hz, quinoline 5-H), 7.32 (1H, dd, *J* 9.3, 2.6 Hz, quinoline 7-H), 7.19 (1H, d, *J* 8.2 Hz, indole 6-H or indole 7-H), 6.85 (1H, dd, *J* 8.2, 1.1 Hz, indole 6-H or indole 7-H), 6.03 (1H, app. s, C*H*(OH)), 4.29-4.22 (1H, m, 14-H$_A$), 4.00 (3H, s, OCH_3), 3.39 (1H, s, 11-H), 3.06 (1H, app. t, *J* 8.3, 13-H), 2.68 (1H, app. dt, *J* 4.2, 9.2 Hz, 14-H$_B$), 2.41 (3H, s, CH_3), 2.36-2.32 (1H, m, 12-H$_A$), 2.21-2.14 (1H, m, 15-H$_A$), 1.70-1.61 (1H, m, 15-H$_B$), 1.13 (1H, t, *J* 11.1 Hz, 12-H$_B$). **^{13}C NMR** (125 MHz, CD_3OD): δ 159.7 (quinoline 6-C), 151.1 (quinoline 4-C), 148.2 (quinoline 2-C), 144.6 (Ar-C$_q$), 142.4 (Ar-C$_q$), 134.0 (Ar-C$_q$), 131.3 (quinoline 8-C), 129.6 (Ar-C$_q$), 127.8 (Ar-C$_q$), 123.9 (Ar-C$_q$), 123.7 (quinolone 7-C), 122.7 (indole 6-C or indole 7-C), 122.3 (Ar-C$_q$), 119.3 (quinoline 3-C), 116.4 (indole 4-C), 112.5 (indole 6-C or indole 7-C), 101.9 (quinoline 5-C), 71.5 (CH(OH)), 67.2 (13-C), 56.5 (OCH_3), 48.5[§] (14-C), 30.4 (15-C), 29.5 (11-C), 27.4 (12-C), 21.7 (CH_3). **HRMS** (ESI): $C_{25}H_{26}O_2N_3$ $[M+H]^+$; calculated: 400.2020, found: 400.2019. $[\boldsymbol{\alpha}]_{\mathbf{20}}^{\boldsymbol{D}}$ = +70 (c. 0.1, MeOH).

[§] Inferred from HSQC analysis.

(*1R*,*11S*,*13S*)-5-Carboxy-13-[(*R*)-hydroxy(6-methoxyquinolin-4-yl)methyl]-1,9-diazatetracyclo[9.2.2.0^{2,10}.0^{3,8}]pentadeca-2(10),3(8),4,6-tetraen-1-ium trifluoroacetate 10f

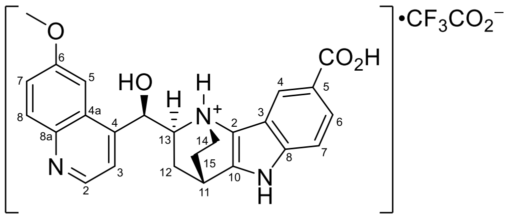

10f

Prepared according to General Procedure B using ketone **6** and 10 mol% $Pd(OAc)_2$ (24 h). Purification by mass-directed preparative HPLC (MeCN in H_2O + 0.1% TFA) gave the *title compound* **10f** (15 mg, 28 μmol, 9%) as a colourless solid. **^{1}H NMR** (500 MHz, CD_3OD, CO_2H, NH^+ and OH not observed): δ 8.91 (1H, d, *J* 5.3, quinoline 2-H), 8.19 (1H, d, *J* 5.3, quinoline 3-H), 8.17 (1H, s, 4-H), 8.05 (1H, d, *J* 9.3, quinoline 8-H), 7.87 (1H, d, *J* 8.5, 6-H), 7.84 (1H, d, *J* 8.5, 7-H), 7.68 (1H, d, *J* 2.2, quinoline 5-H), 7.62 (1H, dd, *J* 9.3, 2.2, quinoline 7-H), 6.63 (1H, app. s, CH(OH)), 4.88-4.81 (1H, m, 14-H_A), 4.08 (3H, s, OCH_3), 3.84-3.80 (1H, m, 11-H), 3.75 (1H, dd, *J* 10.0, 6.8, 13-H), 3.36-3.28 (1H, m, 14-H_B), 2.71-2.63 (1H, m, 12-H_A), 2.60-2.45 (1H, m, 15-H_A) 2.04-1.95 (1H, m, 15-H_B), 1.52-1.43 (1H, m, 12-H_B). **^{13}C NMR** (125 MHz, CD_3OD, $\mathit{C}F_3CO_2^-$ not observed): δ 170.4 (CO_2H), 162.9 (dd, *J* 34.6, $CF_3\mathit{C}O_2^-$), 161.7 (quinoline 6-C), 154.6 (Ar-C_q), 144.2 (quinolone 2-C), 143.7 (Ar-C_q), 138.5 (Ar-C_q), 134.9 (Ar-C_q), 128.5 (Ar-C_q), 127.4 (quinolone 7-C), 126.8 (quinoline 8-C), 125.4 (Ar-C_q), 123.4 (6-C), 121.6 (Ar-C_q), 120.5 (quinoline 3-C), 118.3 (Ar-C_q), 116.5 (7-C), 116.1 (4-C), 102.3 (quinoline 5-C), 69.5 (13-C), 68.0 (CH(OH)), 57.3 (OCH_3), 51.5 (14-C), 29.0 (11-C), 26.9 (15-C), 24.8 (12-C). **HRMS** (ESI): $C_{25}H_{24}N_3O_4$ $[M+H]^+$; calculated: 430.1761, found: 430.1759. $[\alpha]_{20}^{D}$ = −4.0 (c. 0.1, MeOH).

(*R*)-(6-Methoxyquinolin-4-yl)[(*11S*,*13S*)-5-(trifluoromethyl)-1,9-diazatetracyclo [9.2.2.0^{2,10}.0^{3,8}]pentadeca-2(10),3(8),4,6-tetraen-13-yl]methanol 10h

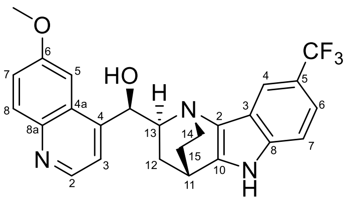

10h

Prepared according to General Procedure B using ketone **6** and 30 mol% $Pd(OAc)_2$ (72 h). Flash column chromatography eluting with 1:9 to 1:4 ({50:80:1 CH_2Cl_2:EtOH:NH_4OH}/CH_2Cl_2) gave the *title compound* **10h** (34 mg, 75 μmol, 23%) as a yellow solid. **^{1}H NMR** (500 MHz, CD_3OD, NH and OH not observed): δ 8.64 (1H, d, *J* 4.7, quinoline 2-H), 8.00 (1H, s, indole 4-H), 7.85 (1H, d, *J* 9.2, quinoline 8-H), 7.76 (1H, d, *J* 4.7, quinoline 3-H), 7.45 (1H, d, *J* 8.5, indole 7-H), 7.37 (1H, d, *J* 2.6, quinoline 5-H), 7.33-7.25 (2H, m, indole 6-H, quinoline 7-H), 6.02 (1H, app. s, C*H*(OH)), 4.29-4.22 (1H, m, 14-H_A), 3.96 (3H, s, OCH_3), 3.44 (1H, app. s, 11-H), 3.06-3.00 (1H, m, 13-H), 2.67 (1H, app. td, *J* 11.1, 3.7, 14-H_B), 2.40-2.34 (1H, m, 12-H_A), 2.23-2.16 (1H, m, 15-H_A), 1.70-1.63 (1H, m, 15-H_B), 1.17-1.10 (1H, m, 12-H_B). **^{13}C NMR** (125 MHz, CD_3OD): δ 159.7 (quinoline 6-C), 151.1 (Ar-C_q), 148.1 (quinoline 2-C), 145.2 (Ar-C_q), 144.5 (Ar-C_q), 136.9 (Ar-C_q), 131.3 (quinoline 8-C), 127.2 (q, J_{CF} 270.4, CF_3), 128.7 (Ar-C_q), 127.7 (Ar-C_q), 123.8 (quinoline 7-C), 122.8 (q, J_{CF} 31.3, indole 5-C), 121.6 (Ar-C_q), 119.2 (quinoline 3-C), 117.5 (q, J_{CF} 3.4, indole 6-C), 114.2 (q, J_{CF} 4.3, indole 4-C), 113.2 (indole 7-C), 101.8 (quinoline 5-C), 71.7 (CH(OH)), 67.1 (13-C), 56.5 (OCH_3), 48.3 (14-C), 30.5 (15-C), 29.7 (11-C), 27.3 (12-C). **HRMS** (ESI): $C_{25}H_{23}O_2N_3F$ $[M+H]^+$; calculated: 454.1737, found: 454.1733. $[\alpha]_{20}^{D}$ = +94 (c. 0.1, MeOH).

(*1R*,*11S*,*13S*)-13-[(*R*)-hydroxy(6-methoxyquinolin-4-yl)methyl]-5-nitro-1,9-diazatetracyclo[9.2.2.0^{2,10}.0^{3,8}]pentadeca-2(10),3(8),4,6-tetraen-1-ium trifluoroacetate 10i

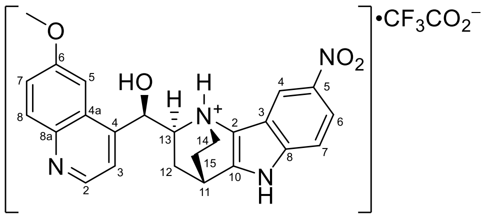

10i

Prepared according to General Procedure B using ketone **6** and 30 mol% $Pd(OAc)_2$ (72 h). Flash column chromatography eluting with 1:9 to 1:4 ({50:80:1 CH_2Cl_2:EtOH:NH_4OH}/CH_2Cl_2) followed by mass-directed preparative HPLC (MeCN in H_2O + 0.1% TFA) gave the *title compound* **10i** (5 mg, 8 μmol, 3%) as a yellow solid. **^{1}H NMR** (500 MHz, CD_3OD, NH^+, NH and OH not observed): δ 8.90 (1H, d, *J* 5.3, quinoline 2-H), 8.83 (1H, d, *J* 2.2, indole 4-H), 8.17 (1H, d, *J* 5.3, quinoline 3-H), 8.09 (1H, dd, *J* 9.0, 2.2, indole 6-H), 8.04 (1H, d, *J* 9.3, quinoline 8-H), 7.71 (1H, d, *J* 2.4, quinoline 5-H), 7.61 (1H, dd, *J* 9.3, 2.4, quinoline 7-H), 7.58 (1H, d, *J* 9.0, indole 7-H), 6.63 (1H, app. s, C*H*(OH)), 4.85-4.79 (1H, m, 14-H_A), 4.10 (3H, s, OCH_3), 3.84-3.80 (1H, m, 11-H), 3.76-3.71 (1H, m, 13-H), 2.71-2.64 (1H, m, 12-H_A), 2.54-2.47 (1H, m, 15-H_A), 2.05-1.96 (1H, m, 15-H_B), 1.50-1.43 (1H, m, 12-H_B). **^{13}C NMR** (125 MHz, CD_3OD, $\mathit{C}F_3CO_2^-$ not observed): δ 163.0 (q, J_{CF} 30.9, $CF_3\mathit{C}O_2^-$), 161.6 (quinoline 6-C), 144.6 (Ar-C_q), 144.3 (Ar-C_q), 144.0 (quinoline 2-C), 143.1 (Ar-C_q), 140.5 (Ar-C_q), 138.7 (Ar-C_q), 138.5 (Ar-C_q), 128.4 (Ar-C_q), 127.3 (quinoline 7-C), 126.9 (quinolone 8-C), 120.4 (quinoline 3-C), 118.2 (Ar-C_q), 118.2 (indole 6-C), 114.2 (indole 4-C), 113.8 (indole 7-C), 102.43 (quinoline 5-C), 69.5 (13-C), 68.1 (CH(OH)), 57.4 (OCH_3), 51.2 (14-C), 29.1 (11-C), 27.1 (15-C), 24.9 (12-C). **HRMS** (ESI): $C_{24}H_{23}O_4N_4$ $[M+H]^+$; calculated: 431.1714, found: 431.1709. $[\alpha]_{20}^{D}$ = +52 (c. 0.1, MeOH).

(*R*)-[(*11S*,*13S*)-5-Methoxy-1,9-diazatetracyclo[9.2.2.0^{2,10}.0^{3,8}]pentadeca-2(10),3(8),4,6-tetraen-13-yl](6-methoxyquinolin-4-yl)methanol 10j

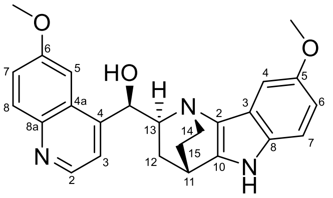

10j

Prepared according to General Procedure B using ketone **6** and 30 mol% $Pd(OAc)_2$ (72 h). Flash column chromatography eluting with 1:9 to 1:3 ({50:80:1 CH_2Cl_2:EtOH:NH_4OH}/CH_2Cl_2) gave the *title compound* **10j** (8 mg, 20 μmol, 6%) as a yellow solid. **^{1}H NMR** (600 MHz, CD_3OD, NH and OH not observed): δ 8.64 (1H, d, *J* 4.7, quinoline 2-H), 7.87 (1H, d, *J* 9.2, quinoline 8-H), 7.76 (1H, d, *J* 4.7, quinoline 3-H), 7.41 (1H, d, *J* 2.6, quinoline 5-H), 7.32 (1H, dd, *J* 9.2, 2.6, quinoline 7-H), 7.21-7.16 (2H, m, indole 4-H and indole 7-H), 6.66 (1H, dd, *J* 8.8, 2.4, indole 6-H), 6.01 (1H, app. s, C*H*(OH)), 4.26-4.20 (1H, m, 14-H_A), 4.01 (3H, s, OCH_3), 3.82 (3H, s, OCH_3), 3.38-3.34 (1H, m, 11-H), 3.09-3.02 (1H, m, 13-H), 2.68-2.62 (1H, m, 14-H_B), 2.37-2.31 (1H, m, 12-H_A), 2.19-2.13 (1H, m, 15-H_A), 1.66-1.60 (1H, m, 15-H_B), 1.16-1.10 (1H, m, 12-H_B). **^{13}C NMR** (150 MHz, CD_3OD): δ 159.6 (quinoline 6-C), 155.6 (indole 5-C), 151.3 (Ar-C_q), 148.2 (quinoline 2-C), 144.6 (Ar-C_q), 143.2 (Ar-C_q), 131.3 (Ar-C_q), 130.8 (quinoline 8-C), 127.9 (Ar-C_q), 127.8 (Ar-C_q), 123.7 (quinoline 7-C), 122.5 (Ar-C_q), 119.3 (quinoline 3-C), 113.3 (indole 4-C or indole 7-C), 110.9 (indole 6-C), 102.0 (quinoline 5-C), 99.1 (indole 4-C or indole 7-C), 71.8 (CH(OH)), 67.0 (13-C), 56.7 (OCH_3), 56.2 (OCH_3), 48.5[§] (14-C), 30.5 (15-C), 29.6 (11-C), 27.6 (12-C). **HRMS** (ESI): $C_{25}H_{26}O_3N_3$ $[M+H]^+$; calculated: 416.1969, found: 416.1972. $[\alpha]_{20}^{D}$ = −110 (c. 0.1, MeOH).

[§] Inferred from HSQC analysis.

(*R*)-(6-methoxyquinolin-4-yl)[(*11S*,*13S*)-5-(trifluoromethoxy)-1,9-diazatetracyclo[9.2.2.0^{2,10}.0^{3,8}]pentadeca-2(10),3(8),4,6-tetraen-13-yl]methanol 10k

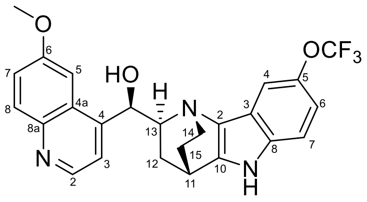

10k

Prepared according to General Procedure B using ketone **6** and 10 mol% Pd(OAc)$_2$ (24 h). Flash column chromatography eluting with 5-20% {50:80:1 CH_2Cl_2:EtOH:NH_4OH} in CH_2Cl_2 gave the *title compound* **10k** (98 mg, 0.21 mmol, 65%) as a pale brown solid. **^{1}H NMR** (400 MHz, CD_3OD, NH and OH not observed): δ 8.56 (1H, d, *J* 4.7, quinoline 2-H), 7.73 (1H, d, *J* 9.2, quinoline 8-H), 7.69 (1H, d, *J* 4.7, quinoline 3-H), 7.53 (1H, d, *J* 1.1, indole 4-H), 7.30-7.26 (2H, m, quinoline 5-H and indole 7-H), 7.13 (1H, dd, *J* 9.2, 2.7, quinoline 7-H), 6.85 (1H, ddd, *J* 8.8, 2.3, 0.8, indole 6-H), 5.94 (1H, s, app. s, C*H*(OH)), 4.16 (1H, ddd, *J* 12.7, 8.8, 4.1, 14-H$_A$), 3.88 (3H, s, OCH_3), 3.33-3.27 (1H, m, 11-H), 3.01-2.94 (1H, m, 13-H), 2.59-2.50 (1H, m, 14-H$_B$), 2.27 (1H, ddd, *J* 12.0, 6.6, 2.0, m, 12-H$_A$), 2.12-2.03 (1H, m, 15-H$_A$), 1.58-1.47 (1H, m, 15-H$_B$), 1.11-1.02 (1H, m, 12-H$_B$). **^{13}C NMR** (175 MHz, CD_3OD): δ 159.5 (quinolone 6-C), 151.1 (Ar-C$_q$), 148.0 (quinoline 2-C), 145.3 (Ar-C$_q$), 144.5 (Ar-C$_q$), 144.4 (Ar-C$_q$), 133.8 (Ar-C$_q$), 131.2 (quinoline 8-C), 128.5 (Ar-C$_q$), 127.6 (Ar-C$_q$), 123.6 (quinolone 7-C), 122.3 (Ar-C$_q$), 122.2 (q, J_{CF} 253.5, CF_3), 119.2 (quinoline 3-C), 114.5 (indole 6-C), 113.4 (indole 7-C), 109.0 (indole 4-C), 101.7 (quinoline 5-C), 71.7 (CH(OH)), 66.8 (13-C), 56.5 (OCH_3), 48.4 (14-C), 30.4 (15-C), 29.6 (11-C), 27.4 (12-C). **HRMS** (ESI): $C_{25}H_{23}O_3N_3F_3$ [M+H]$^+$; calculated: 470.1686, found: 470.1680. $[\alpha]_{20}^{D}$ = +33 (c. 0.1, MeOH).

(*R*)-[(*11S*,*13S*)-5-Fluoro-1,9-diazatetracyclo[9.2.2.0^{2,10}.0^{3,8}]pentadeca-2(10),3(8),4,6-tetraen-13-yl](6-methoxyquinolin-4-yl)methanol 10l

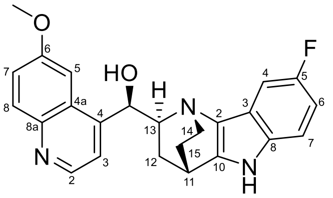

10l

Prepared according to General Procedure B using ketone **6** and 20 mol% $Pd(OAc)_2$ (48 h). Flash column chromatography eluting with 1:9 to 1:4 ({50:80:1 CH_2Cl_2:EtOH:NH_4OH}/CH_2Cl_2) gave the *title compound* **10l** as a yellow solid (61 mg, 151 μmol, 47%). **^{1}H NMR** (500 MHz, CD_3OD, NH and OH not observed): δ 8.65 (1H, d, *J* 4.7, quinoline 2-H), 7.86 (1H, d, *J* 9.2, quinoline 8-H), 7.76 (1H, d, *J* 4.7, quinoline 3-H), 7.39 (1H, d, *J* 2.6, quinoline 5-H), 7.32 (1H, dd, *J* 2.6, 9.2, quinoline 7-H), 7.29 (1H, dd, *J* 9.9, 2.5, indole 4-H), 7.25 (1H, dd, *J* 8.9, 4.4, indole 7-H), 6.76 (1H, app. td, *J* 9.2, 2.5, indole 6-H), 6.00 (1H, app. s, C*H*(OH)), 4.26-4.19 (1H, m, 14-H_A), 3.99 (3H, s, OCH_3), 3.40-3.37 (1H, m, 11-H), 3.06-3.00 (1H, m, H-13), 2.64 (1H, app. td, *J* 11.5, 4.1, 14-H_B), 2.37-2.32 (1H, m, 12-H_A), 2.21-2.13 (1H, m, 15-H_A), 1.67-1.59 (1H, m, 15-H_B), 1.15 (1H, t, *J* 11.1, 12-H_B). **^{13}C NMR** (125 MHz, CD_3OD): δ 159.7 (quinoline 6-C), 159.5 (d, J_{CF} 232.6, indole 5-C), 151.2 (Ar-C_q), 148.2 (quinoline 2-C), 144.8 (Ar-C_q), 144.6 (Ar-C_q), 132.2 (Ar-C_q), 131.3 (quinoline 8-C), 127.8 (Ar-C_q), 123.7 (quinoline 7-C), 122.4 (Ar-C_q), 122.3 (Ar-C_q), 119.3 (quinoline 3-C), 113.4 (d, J_{CF} 9.9, indole 7-C), 108.8 (d, J_{CF} 26.5, indole 6-C), 101.9 (quinolone 5-C), 101.5 (d, J_{CF} 24.6, indole 4-C), 71.7 (CH(OH)), 66.9 (13-C), 56.6 (OCH_3), 48.5[§] (14-C), 30.4 (15-C), 29.7 (11-C), 27.6 (12-C). **HRMS** (ESI): $C_{24}H_{23}O_2N_3F$ $[M+H]^+$; calculated: 404.1769, found: 404.1769. $[\alpha]_{20}^{D}$ = +64 (c. 0.1, MeOH).

[§] Inferred from HSQC analysis.

(*R*)-[(*11S*,*13S*)-5-Chloro-1,9-diazatetracyclo[9.2.2.0^{2,10}.0^{3,8}]pentadeca-2(10),3(8),4,6-tetraen-13-yl](6-methoxyquinolin-4-yl)methanol 10m

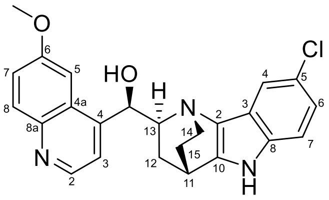

10m

Prepared according to General Procedure B using ketone **6** and 10 mol% Pd(OAc)$_2$ (24 h). Flash column chromatography eluting with 1:9 to 1:4 ({50:80:1 CH_2Cl_2:EtOH:NH_4OH}/CH_2Cl_2) gave the *title compound* **10m** (80 mg, 191 μmol, 59%) as a yellow solid. **^{1}H NMR** (500 MHz, CD_3OD): δ 8.64 (1H, d, *J* 4.6, quinoline 2-H), 7.85 (1H, d, *J* 9.3, quinoline 8-H), 7.75 (1H, d, *J* 4.6, quinoline 3-H), 7.61 (1H, d, *J* 2.0, indole 4-H), 7.37 (1H, d, *J* 2.6, quinoline 5-H), 7.30 (1H, dd, *J* 9.3, 2.6, quinoline 7-H), 7.26 (1H, d, *J* 8.5, indole 7-H), 6.96 (1H, dd, *J* 8.5, 2.0, indole 6-H), 5.99 (1H, app. s, C*H*OH), 4.25-4.18 (1H, m 14-H_A), 3.99 (3H, s, OCH_3), 3.39 (1H, app. s, 11-H), 3.03-2.98 (1H, m, 13-H), 2.63 (1H, td, *J* 11.1, 3.7 Hz 14-H_B), 2.37-2.31 (1H, m, 12-H_A), 2.19-2.15 (1H, m CH2 15-H_A), 1.67-1.59 (1H, m, 15-H_B), 1.16-1.09 (1H, m, 12-H_B). **^{13}C NMR** (125 MHz, CD_3OD): 159.7 (quinoline 6-C), 151.2 (Ar-C_q), 148.1 (quinoline 2-C), 144.6 (Ar-C_q), 144.5 (Ar-C_q), 133.9 (Ar-C_q), 131.3 (quinoline 8-C), 127.8 (Ar-C_q), 127.7 (Ar-C_q), 126.3 (Ar-C_q), 123.7 (quinoline 7-C), 123.2 (Ar-C_q), 121.0 (indole 6-C or indole 7-C), 119.3 (quinolone 3-C), 116.2 (indole 4-C), 113.9 (indole 6-C or indole 7-C), 101.8 (quinoline 5-C), 71.7 (CH(OH)), 66.9 (13-C), 56.5 (OCH_3), 47.1[§] (C-14), 30.5 (15-C), 29.6 (11-C), 27.5 (12-C). **HRMS** (ESI): $C_{24}H_{23}O_2N_3Cl^{35}$ $[M+H]^+$; calculated: 420.1473, found: 420.1475. $[\alpha]_{20}^{D} = -19$ (c. 0.1, MeOH).

[§] Inferred from HSQC analysis.

(*R*)-[(*11S*,*13S*)-5-Bromo-1,9-diazatetracyclo[9.2.2.0^{2,10}.0^{3,8}]pentadeca-2(10),3(8),4,6-tetraen-13-yl](6-methoxyquinolin-4-yl)methanol 10n

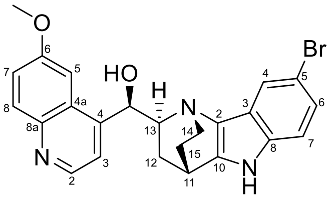

10n

Prepared according to General Procedure B using ketone **6** and 20 mol% $Pd(OAc)_2$ (48 h). Flash column chromatography eluting with 1:9 to 1:4 ({50:80:1 CH_2Cl_2:EtOH:NH_4OH}/CH_2Cl_2) the *title compound* **10n** (22 mg, 48 μmol, 15%) as a yellow solid. **^{1}H NMR** (600 MHz, CD_3OD, NH and OH not observed): δ 8.64 (1H, d, *J* 4.7, quinoline 2-H), 7.86 (1H, d, *J* 9.2, quinoline 8-H), 7.77 (1H, d, *J* 1.8, indole 4-H), 7.75 (1H, d, *J* 4.7, quinoline 3-H), 7.38 (1H, d, *J* 2.6, quinoline 5-H), 7.32 (1H, dd, *J* 9.2, 2.7, quinoline 7-H), 7.22 (1H, d, *J* 8.5, indole 7-H), 7.09 (1H, dd, *J* 8.5, 1.8, indole 6-H), 5.99 (1H, app. s, C*H*(OH)), 4.25-4.19 (1H, m, 14-H_A), 4.00 (3H, s, OCH_3), 3.41-3.38 (1H, m, 11-H), 3.05-2.99 (1H, m, 13-H), 2.68-2.61 (1H, m, 14-H_B), 2.37-2.32 (1H, m, 12-H_A), 2.21-2.15 (1H, m, 15-H_A), 1.68-1.61 (1H, m, 15-H_B), 1.17-1.10 (1H, m, 12-H_B).**^{13}C NMR** (150 MHz, CD_3OD): δ 159.7 (quinoline 6-C), 151.1 (Ar-C_q), 148.2 (quinoline 2-C), 144.6 (Ar-C_q), 144.5 (Ar-C_q), 134.3 (Ar-C_q), 131.3 (quinoline 8-C), 127.8 (Ar-C_q), 127.5 (Ar-C_q), 123.9 (Ar-C_q), 123.7 (quinoline 7-C), 123.7 (indole 6-C), 119.3 (indole 4-C), 119.3 (quinoline 3-C), 114.3 (indole 7-C), 113.9 (Ar-C_q), 101.9 (quinoline 5-C), 71.7 (CH(OH)), 67.1 (13-C), 56.6 (OCH_3), 48.3 (14-C), 30.5 (15-C), 29.7 (11-C), 27.5 (12-C). **HRMS** (ESI): $C_{24}H_{23}O_2N_3{}^{79}Br$ $[M+H]^+$; calculated: 464.0968, found: 464.0965. $C_{24}H_{23}O_2N_3{}^{81}Br$ $[M+H]^+$; calculated: 466.0948, found: 466.0945. $[\alpha]_{20}^{D}$ = +164 (c. 0.1, MeOH).

(*R*)-(6-Methoxyquinolin-4-yl)[(*11S*,*13S*)-6-methyl-1,9-diazatetracyclo[9.2.2.0^{2,10}.0^{3,8}] pentadeca-2(10),3(8),4,6-tetraen-13-yl]methanol 10o

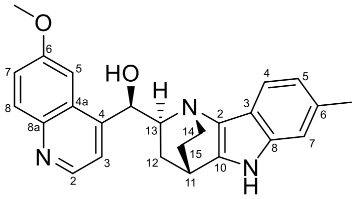

10o

Prepared according to General Procedure B using ketone **6** and 20 mol% Pd(OAc)$_2$ (48 h). Flash column chromatography eluting with 1:9 to 1:4 ({50:80:1 CH_2Cl_2:EtOH:NH_4OH}/CH_2Cl_2) gave the *title compound* **10o** (5 mg, 12 μmol, 4%) as a yellow solid. **^{1}H NMR** (500 MHz, CD_3OD, NH and OH not observed): δ 8.65 (1H, d, *J* 4.7, quinoline 2-H), 7.87 (1H, d, *J* 9.2, quinoline 8-H), 7.76 (1H, d, *J* 4.7, quinoline 3-H), 7.51 (1H, d, *J* 8.0, indole 4-H), 7.39 (1H, d, *J* 2.4, quinoline 5-H), 7.33 (1H, dd, *J* 9.2, 2.4, quinoline 7-H), 7.11 (1H, s, indole 7-H), 6.87 (1H, d, *J* 8.0, indole 5-H), 6.02 (1H, app. s, C*H*(OH)), 4.27-4.21 (1H, m, 14-H_A), 3.99 (3H, s, OCH_3), 3.40-3.36 (1H, m, 11-H), 3.08-3.02 (1H, m, 13-H), 2.70-2.62 (1H, m, 14-H_B), 2.39 (3H, s, CH_3) 2.37-2.31 (1H, m, 12-H_A), 2.21-2.13 (1H, m, 15-H_A), 1.68-1.60 (1H, m, 15-H_B), 1.16-1.09 (1H, m, 12-H_B). **^{13}C NMR** (125 MHz, CD_3OD): δ 159.7 (quinoline 6-C), 151.2 (Ar-C_q), 148.1 (quinolone 2-C), 144.5 (Ar-C_q), 141.5 (Ar-C_q), 136.0 (Ar-C_q), 131.2 (quinoline 8-C), 130.7 (Ar-C_q), 127.8 (Ar-C_q), 123.7 (quinoline 7-C), 122.1 (indole 5-C), 120.2 (Ar-C_q) 120.1 (Ar-C_q), 119.3 (quinoline 3-C), 116.4 (indole 4-C), 112.7 (indole 7-C), 101.9 (quinoline 5-C), 71.6 (CH(OH)), 67.0 (13-C), 56.6 (OCH_3), 46.3 (14-C), 30.5 (15-C), 29.5 (11-C), 27.6 (12-C), 21.8 (CH_3). **HRMS** (ESI): $C_{25}H_{26}O_2N_3$ [M+H]$^+$; calculated: 400.2020, found: 400.2019. $[\alpha]_{20}^{D}$ = −31 (c. 0.1, MeOH).

(*1R*,*11S*,*13S*)-6-Carboxy-13-[(*R*)-hydroxy(6-methoxyquinolin-4-yl)methyl]-1,9-diaza-tetracyclo[9.2.2.0^{2,10}.0^{3,8}]pentadeca-2(10),3(8),4,6-tetraen-1-ium trifluoroacetate 10p

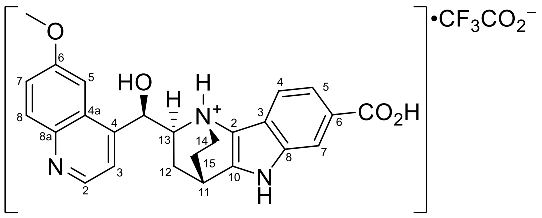

10p

Prepared according to General Procedure B using ketone **6** and 20 mol% $Pd(OAc)_2$ (48 h). Purification by mass-directed preparative HPLC (MeCN in H_2O + 0.1% TFA; Rt = 7.2 min) gave the *title compound* **10p** (25 mg, 46 μmol, 14%) as a yellow solid. **^{1}H NMR** (500 MHz, CD_3OD, CO_2H, NH^+, NH and OH not observed): δ 8.87 (1H, d, *J* 5.3, quinoline 2-H), 8.24 (1H, d, *J* 1.4, indole 7-H), 8.14 (1H, d, *J* 9.3, quinoline 8-H), 7.89 (1H, d, *J* 5.3, quinoline 3-H), 7.78 (1H, dd, *J* 8.5, 1.4, indole 5-H), 7.72 (1H, dd, *J* 9.3, 2.5, quinoline 7-H), 7.48 (1H, d, *J* 2.5, quinoline 5-H), 7.33 (1H, d, *J* 8.5, indole 4-H), 5.62 (1H, d, *J* 5.4, C*H*(OH)), 4.60 (1H, app. dt, *J* 8.6, 5.9, 13-H), 4.03-3.94 (1H, m, 14-H_A), 3.88-3.82 (1H, m, 11-H), 3.81 (3H, s, OCH_3), 3.19-3.12 (1H, m, 14-H_B), 2.31-2.23 (2 H, m, 12-H_A and 15-H_A), 2.14-2.05 (1H, m, 12-H_B), 1.93-1.81 (1H, m, 15-H_B). **^{13}C NMR** (125 MHz, CD_3OD, *C*$F_3CO_2^-$ not observed): δ 170.5 (CO_2H), 162.6 (q, J_{CF} 35.6, CF_3*C*O_2^-), 161.3 (quinoline 6-C), 153.5 (Ar-C_q), 144.2 (quinoline 2-C), 143.2 (Ar-C_q), 138.8 (Ar-C_q), 135.1 (Ar-C_q), 129.1 (Ar-C_q), 127.3 (quinoline 7-C), 126.8 (quinoline 8-C), 125.2 (Ar-C_q), 123.4 (Ar-C_q), 123.1 (indole 5-C), 121.9 (quinoline 3-C), 116.8 (Ar-C_q), 115.9 (indole 7-C), 113.9 (indole 4-C), 103.3 (quinoline 5-C), 69.9 (CH(OH)), 67.6 (13-C), 57.1 (14-C), 56.6 (OCH_3), 30.1 (12-C), 28.7 (11-C), 25.2 (15-C). **HRMS** (ESI): $C_{25}H_{24}O_4N_3$ $[M+H]^+$; calculated: 430.1761, found: 430.1759. $[\alpha]_{20}^{D}$ = +71 (c. 0.1, MeOH).

(*R*)-(6-Methoxyquinolin-4-yl)[(*11S*,*13S*)-6-(trifluoromethyl)-1,9-diazatetracyclo [9.2.2.0^{2,10}.0^{3,8}]pentadeca-2(10),3(8),4,6-tetraen-13-yl]methanol 10r

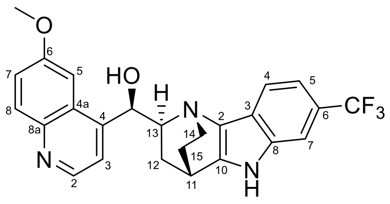

10r

Prepared according to General Procedure B using ketone **6** and 10 mol% Pd(OAc)$_2$ (24 h). Flash column chromatography eluting with 1:9 to 1:4 ({50:80:1 CH_2Cl_2:EtOH:NH_4OH}/CH_2Cl_2) gave the *title compound* **10r** (101 mg, 0.22 mmol, 70%) as a yellow solid. **^{1}H NMR** (500 MHz, CD_3OD, NH and OH not observed): δ 8.65 (1H, d, *J* 4.7, quinoline 2-H), 7.87 (1H, d, *J* 9.2, quinoline 8-H), 7.78-7.74 (2 H, m, quinoline 3-H indole 5-H), 7.63 (1H, s, indole 7-H), 7.40 (1H, d, *J* 2.6, quinoline 5-H), 7.32 (1H, dd, *J* 9.2, 2.6, quinoline 7-H), 7.28 (1H, d, *J* 7.6, indole 4-H), 6.01 (1H, app. s, C*H*(OH)), 4.27-4.21 (1H, m, 14-H_A), 3.98 (3H, s, OCH_3), 3.47-3.43 (1H, m, 11-H), 3.07-3.02 (1H, m, 13-H), 2.67-2.60 (1H, m, 14-H_B), 2.41-2.35 (1H, m, 12-H_A), 2.24-2.17 (1H, m, 15-H_A), 1.68-1.61 (1H, m, 15-H_B), 1.22-1.15 (1H, m, 12-H_B). **^{13}C NMR** (125 MHz, CD_3OD): δ 159.7 (quinoline 6-C), 151.1 (Ar-C_q), 148.2 (quinoline 2-C), 146.4 (Ar-C_q), 144.6 (Ar-C_q), 134.3 (Ar-C_q), 131.3 (quinoline 8-C), 128.3 (Ar-C_q), 127.8 (Ar-C_q), 126.5 (q, J_{CF} 271.5, CF_3), 124.4 (Ar-C_q), 123.7 (quinoline 7-C), 122.8 (q, J_{CF} 31.7, indole 6-C), 117.0 (q, J_{CF} 2.2, indole 5-C), 117.0 (indole 4-C), 110.2 (q, J_{CF} 5.2, indole 7-C), 101.9 (quinolone 5-C), 71.7 (CH(OH)), 66.8 (13-C), 56.6 (OCH_3), 48.4 (14-C), 30.3 (15-C), 29.7 (11-C), 27.6 (12-C). **HRMS** (ESI): $C_{25}H_{23}O_2N_3F_3$ $[M+H]^+$; calculated: 454.1737, found: 454.1735. $[\boldsymbol{\alpha}]_{\mathbf{20}}^{\boldsymbol{D}}$ = +20 (c. 0.1, MeOH).

(*R*)-[(*11S*,*13S*)-6-Chloro-1,9-diazatetracyclo[9.2.2.0^{2,10}.0^{3,8}]pentadeca-2(10),3(8),4,6-tetraen-13-yl](6-methoxyquinolin-4-yl)methanol 10s

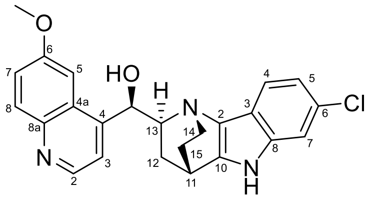

10s

Prepared according to General Procedure B using ketone **6** and 20 mol% $Pd(OAc)_2$ (48 h). Flash column chromatography eluting with 1:9 to 1:4 ({50:80:1 CH_2Cl_2:EtOH:NH_4OH}/CH_2Cl_2) gave the *title compound* **10s** (28 mg, 66 μmol, 20%) as a yellow solid. **^{1}H NMR** (500 MHz, CD_3OD): δ 8.64 (1H, d, *J* 4.7, quinoline 2-H), 7.87 (1H, d, *J* 9.2, quinoline 8-H), 7.75 (1H, d, *J* 4.7, quinoline 3-H), 7.57 (1H, d, *J* 8.5, indole 4-H), 7.39 (1H, d, *J* 2.5, quinoline 5-H), 7.33-7.29 (2H, m, indole 7-H and quinoline 7-H), 7.00 (1H, dd, *J* 8.5, 1.8, indole 5-H), 5.99 (1H, s, C*H*(OH)), 4.21 (1H, ddd, *J* 12.7, 8.8, 4.1, 14-H_A), 3.97 (3H, s, OCH3), 3.41-3.38 (1H, m, 11-H), 3.05-2.99 (1H, m, 13-H), 2.61 (1H, td, *J* 11.1, 4.2, 14-H_B), 2.37-2.31 (1H, m, 12-H_A), 2.20-2.15 (1H, m, 15-H_A), 1.62 (1H, td, *J* 11.1, 3.4, 15-H_B), 1.19-1.13 (1H, m, 12-H_B). **^{13}C NMR** (150 MHz, CD_3OD): δ 159.7 (quinoline 6-C), 151.1 (Ar-C_q), 148.2 (quinolone 2-C), 144.6 (Ar-C_q), 143.7 (Ar-C_q), 135.9 (Ar-C_q), 131.3 (quinoline 8-C), 128.1 (Ar-C_q), 127.8 (Ar-C_q), 126.7 (Ar-C_q), 123.6 (quinoline 7-C), 121.0 (Ar-C_q), 120.9 (indole 5-C), 119.4 (quinoline 3-C), 117.6 (indole 4-C), 112.6 (indole 7-C), 102,0 (quinoline 5-H), 71.7 (CH(OH)), 66.9 (13-C), 56.6 (OCH_3), 48.5[§] (14-C) 30.5 (15-C), 29.6 (11-C), 27.7 (12-C). **HRMS** (ESI): $C_{24}H_{23}O_2N_3{}^{35}Cl$ $[M+H]^+$; calculated: 420.1473, found: 420.1472. $C_{24}H_{23}O_2N_3{}^{37}Cl$ $[M+H]^+$; calculated: 422.1444, found: 422.1443. $[\alpha]_{20}^{D}$ = +56 (c. 0.1, MeOH).

§ Inferred from HSQC analysis.

(*S*)-[(*11S*,*13S*)-6-Bromo-1,9-diazatetracyclo[9.2.2.0^{2,10}.0^{3,8}]pentadeca-2(10),3(8),4,6-tetraen-13-yl](6-methoxyquinolin-4-yl)methanol 10t

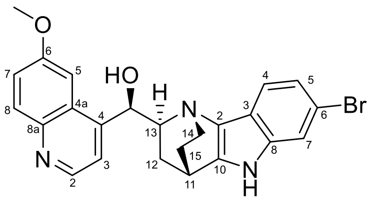

10t

Prepared according to General Procedure B using ketone **6** and 30 mol% $Pd(OAc)_2$ (72 h). Flash column chromatography eluting with 1:9 to 1:4 ({50:80:1 CH_2Cl_2:EtOH:NH_4OH}/CH_2Cl_2) gave the *title compound* **10t** (26 mg, 55 μmol, 27%) as a yellow solid. **^{1}H NMR** (600 MHz, CD_3OD, NH and OH not observed): δ 8.64 (1H, d, *J* 4.6, quinoline 2-H), 7.86 (1H, dd, *J* 9.2, quinoline 8-H), 7.75 (1H, d, *J* 4.6, quinoline 3-H), 7.52 (1H, d, *J* 8.4, indole 4-H), 7.48 (1H, d, *J* 1.7, indole 7-H) 7.39 (1H, d, *J* 2.3, quinoline 5-H), 7.33-7.29 (1H, m, quinoline 7-H), 7.12 (1H, dd, *J* 8.4, 1.7, indole 5-H), 6.01 (1H, app. s, C*H*(OH)), 4.27-4.18 (1H, m, 14-H_A), 3.97 (3H, s, OCH_3), 3.41-3.38 (1 H, m, 11-H), 3.10-2.97 (1H, m, 13-H), 2.68-2.58 (1H, m, 14-H_B), 2.38-2.31 (1H, m, 12-H_A), 2.21-2.14 (1H, m, 15-H_A), 1.67-1.58 (1H, m, 15-H_B), 1.19-1.12 (1H, m, 12-H_B). **^{13}C NMR** (125 MHz, CD_3OD): δ 159.7 (quinoline 6-C), 151.1 (Ar-C_q), 148.2 (quinoline 2-C), 144.6 (Ar-C_q), 143.7 (Ar-C_q), 136.3 (Ar-C_q), 131.3 (quinoline 8-C), 127.8 (Ar-C_q), 123.6 (quinoline 7-C), 123.5 (indole 5-C), 121.3 (Ar-C_q), 121.1 (Ar-C_q), 119.4 (quinoline 3-C), 118.0 (indole 4-C), 115.6 (indole 7-C), 114.0 (Ar-C_q), 102.0 (quinoline 5-C), 71.7 (CH(OH)), 66.9 (13-C), 56.6 (OCH_3), 48.6 (14-C) 30.4 (15-C), 29.6 (11-C), 27.7 (12-C). **HRMS** (ESI): $C_{24}H_{23}O_2N_3{}^{81}Br$ [M+H]$^+$; calculated: 466.0948, found: 466.0950. $[\alpha]_{20}^{D}$ = +22 (c. 0.1, MeOH).

(*R*)-[(*11S*,*13S*)-7-Methoxy-1,9-diazatetracyclo[9.2.2.0^{2,10}.0^{3,8}]pentadeca-2(10),3(8),4,6-tetraen-13-yl](6-methoxyquinolin-4-yl)methanol 10u

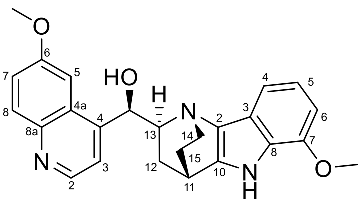

10u

Prepared according to General Procedure B using ketone **6** and 30 mol% $Pd(OAc)_2$ (72 h). Flash column chromatography eluting with 5-20% {50:80:1 CH_2Cl_2:EtOH:NH_4OH} in CH_2Cl_2 gave the *title compound* **10u** (45 mg, 0.11 mmol, 33%) as a colourless solid. **^{1}H NMR** (500 MHz, CD_3OD, NH and OH not observed): δ 8.66 (1H, d, *J* 4.7, quinoline 2-H), 7.88 (1H, d, *J* 9.2, quinoline 8-H), 7.77 (1H, d, *J* 4.7, quinoline 3-H), 7.37 (1H, d, *J* 2.5, quinoline 5-H), 7.34 (1H, dd, *J* 9.2, 2.5, quinoline 7-H), 7.25 (1H, d, *J* 8.0, 6-H), 6.99 (1H, app. t, *J* 7.9, 5-H), 6.62 (1H, d, *J* 7.8, 4-H), 6.07 (1H, s, CH(OH)), 4.44-4.30 (1H, m, 14-H_A), 3.98 (3H, s, OCH_3), 3.92 (3H, s, OCH_3), 3.51-3.46 (1H, m, 11-H), 3.23-3.14 (1H, m, 13-H), 2.84-2.74 (1H, m, 14-H_B), 2.41 (1H, dd, *J* 10.8, 6.5, 12-H_A), 2.28-2.18 (1H, m, 15-H_A), 1.75-1.65 (1H, m, 15-H_B), 1.25-1.16 (1H, m, 12-H_B). **^{13}C NMR** (125 MHz, CD_3OD, 3 × Ar-C_q not observed): δ 159.8 (quinolone 6-C), 148.2 (2 peaks, quinoline 2-C and Ar-C_q), 144.6 (Ar-C_q), 141.3 (Ar-C_q), 131.4 (quinoline 8-C), 127.7 (Ar-C_q), 125.4 (Ar-C_q), 123.7 (quinoline 7-C), 121.4 (5-C), 119.4 (quinoline 3-C), 109.7 (6-C), 102.1 (4-C), 101.8 (quinoline 5-C), 70.9 (CH(OH)), 67.7 (13-C), 56.6 (OCH_3), 55.8 (OCH_3), 51.5 (14-C), 29.7 (11-C), 29.3 (15-C), 27.0 (12-C). **HRMS** (ESI): $C_{25}H_{26}N_3O_3$ $[M+H]^+$; calculated: 416.1969, found: 416.1970. $[\alpha]_{20}^{D}$ = −52 (c. 0.1, MeOH).

(11*S*,13*S*)-7-Fluoro-13-[(1*R*)-1-(6-methoxyquinolin-4-yl)ethyl]-1,9-diazatetracyclo [9.2.2.0^{2,10}.0^{3,8}]pentadeca-3,5,7-triene 10x

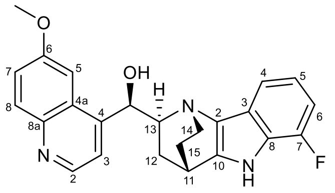

10x

Prepared according to General Procedure B using 30 mol% Pd(OAc)$_2$ (72 h). Flash column chromatography eluting with 10-25% {50:80:1 CH_2Cl_2:EtOH:NH_4OH} in CH_2Cl_2 gave the *title compound* **10x** (24 mg, 59 μmol, 18%) as brown solid. **^{1}H NMR** (500 MHz, CD_3OD, NH and OH not observed): δ 8.62 (1H, d, *J* 4.6, quinoline 2-H), 7.84 (1H, d, *J* 9.2, quinolone 8-H), 7.74 (1H, d, *J* 4.6, quinoline 3-H), 7.41 (1H, d, *J* 8.1, indole 4-H), 7.37 (1H, d, *J* 2.4, quinoline 5-H), 7.28 (1H, dd, *J* 9.2, 2.4, quinoline 7-H), 6.99-6.89 (1H, m, indole 5-H), 6.73 (1H, dd, *J* 11.5, 8.1, indole 6-H), 5.99 (1H, s, C*H*(OH)), 4.25-4.18 (1H, m, 14-H_A), 3.96 (3H, s, OCH_3), 3.41 (1H, s, 11-H), 3.08-2.99 (1H, m, 13-H), 2.66-2.58 (1H, m, 14-H_B), 2.39-2.31 (1H, m, 12-H_A), 2.20-2.13 (1H, m, 15-H_A), 1.66-1.57 (1H, m, 15-H_B), 1.17-1.11 (1H, m, 12-H_B). **^{13}C NMR** (126 MHz, CD_3OD): δ 159.6 (quinoline 6-C), 151.4 (d, J_{CF} 241.9, indole 7-C) 151.2 (Ar-C_q), 148.1 (quinolone 2-C), 144.5 (Ar-C_q), 143.8 (Ar-C_q), 131.2 (quinoline 8-C), 128.8 (Ar-C_q), 127.8 (Ar-C_q), 126.1 (Ar-C_q), 123.6 (quinoline 7-C), 123.2, (Ar-C_q) 120.6 (d, J_{CF} 6.6, indole 5-C), 119.3 (quinolone 3-C), 112.7 (d, J_{CF} 3.1, indole 4-C), 105.8 (d, J_{CF} 16.7, indole 6-C), 101.8 (quinolone 5-C), 71.6 (*C*H(OH)), 66.8 (13-C), 56.6 (OCH_3), 48.4 (14-C), 30.4 (15-C), 29.6 (11-C), 27.6 (12-C). **HRMS** (ESI): $C_{24}H_{23}O_2N_3F$ [M+H]$^+$; calculated: 404.1769, found: 404.1769 $[\alpha]_D^{20}$ = −8 (c. 0.1, MeOH).

(11*S*,13*S*)-7-chloro-13-[(1*R*)-1-(6-methoxyquinolin-4-yl)ethyl]-1,9-diazatetracyclo [9.2.2.0^{2,10}.0^{3,8}]pentadeca-3,5,7-triene 10y

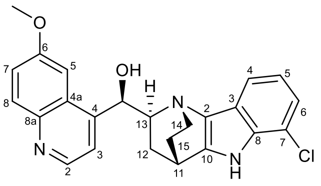

10y

Prepared according to General Procedure B using 10 mol% $Pd(OAc)_2$ (24 h). Flash column chromatography eluting with 10-25% {50:80:1 CH_2Cl_2:EtOH:NH_4OH} in CH_2Cl_2 gave the *title compound* **10y** (71 mg, 169 μmol, 53%) as yellow solid. **^{1}H NMR** (500 MHz, CD_3OD, NH and OH not observed): δ 8.58 (1H, d, *J* 4.6, quinoline 2-H), 7.80 (1H, d, *J* 9.2, quinoline 8-H), 7.76 (1H, d, *J* 4.6, quinoline 3-H), 7.30 (1H, d, *J* 2.3, quinoline 5-H), 7.29-7.26 (1H, m, indole 4-H), 7.24 (1H, dd, *J* 9.2, 2.3, quinoline 7-H), 6.98-6.95 (2H, m, indole 5-H and indole 6-H), 6.37 (1H, app. s, C*H*(OH)), 4.51 (1H, app. s, 14-H_A), 3.95 (3H, s, OCH_3), 3.63-3.56 (1H, m, 11-H)[†††], 3.40 (1H, s, 13-H), 2.95 (1H, app. s, 14-H_B), 2.54-2.45 (1H, m, 12-H_A), 2.35-2.26 (1H, m, 15-H_A), 1.82-1.68 (1H, m, 15-H_B), 1.35 (1H, app. t, *J* 11.2, 12-H_B). **^{13}C NMR** (126 MHz, CD_3OD, 1 × Ar-C_q not observed): δ 159.8 (quinoline 6-C), 149.1 (Ar-C_q), 148.0 (quinoline 2-C), 144.3 (Ar-C_q), 142.9 (Ar-C_q), 136.3 (Ar-C_q), 131.3 (quinoline 8-C), 127.6 (Ar-C_q), 123.7 (quinoline 7-C), 122.9 (indole 6-C), 122.7 (Ar-C_q), 121.4 (indole 5-C), 119.9 (quinoline 3-C), 118.6 (Ar-C_q), 112.1 (indole 4-C), 101.4 (quinoline 5-C), 69.5 (13-C), 68.9 (*C*H(OH)), 56.7 (OCH_3), 51.6 (14-C), 28.8 (11-C), 27.8 (15-C), 26.5 (12-C). **HRMS** (ESI): $C_{24}H_{23}O_2N_3{}^{35}Cl$ $[M+H]^+$; calculated: 420.1473, found: 420.1471; $C_{24}H_{23}O_2N_3{}^{37}Cl$ $[M+H]^+$; calculated: 422.1443, found: 422.1442. $[\alpha]_D^{20}$ = +42 (c. 0.1, MeOH).

[†††] Determined *via* COSY.

(*1R*,*11S*,*13S*)-13-[(*R*)-Hydroxy(6-methoxyquinolin-4-yl)methyl]-7-methoxy-5-methoxycarbonyl)-1,9-diazatetracyclo[9.2.2.0^{2,10}.0^{3,8}]pentadeca-2(10),3(8),4,6-tetraen-1-ium trifluoroacetate 10z

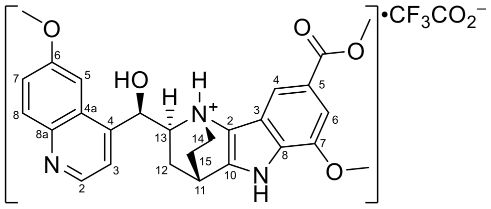

10z

Prepared according to General Procedure B using ketone **6**, $MgSO_4$ (1.5 eq.) and 30 mol% $Pd(OAc)_2$ (72 h). Flash column chromatography eluting with 5-20% {50:80:1 CH_2Cl_2:EtOH:NH_4OH} in CH_2Cl_2, followed by further purification using mass-directed HPLC (MeCN in H_2O + 0.1% TFA) gave the *title compound* **10z** (3 mg, 4.7 µmol, 1%) as an orange oil. **^{1}H NMR** (500 MHz, CD_3OD, NH and OH not observed): δ 8.90 (1H, d, *J* 5.5, quinoline 2-H), 8.23 (1H, d, *J* 1.3, indole 4-H or 6-H), 8.21 (1H, d, *J* 5.5, quinoline 3-H), 8.04 (1H, d, *J* 9.3, quinoline 8-H), 7.71 (1H, d, *J* 2.6, quinoline 5-H), 7.63 (1H, dd, *J* 9.3, 2.6, quinoline 7-H), 7.34 (1H, d, *J* 1.3, indole 4-H or 6-H), 6.66 (1H, s, C*H*(OH)), 4.78 (1H, ddd, *J* 11.8, 9.3, 4.3, 14-H_A), 4.08 (3H, s, OCH_3), 3.98 (3H, s, OCH_3), 3.92 (3H, s, OCH_3), 3.78-3.75 (1H, m, 11-H), 3.72-3.67 (1H, m, 13-H), 3.36-3.29 (1H, 14-H_B)[§], 2.61 (1H, ddd, *J* 12.2, 6.6, 2.2, 12-H_A), 2.50-2.43 (1H, m, 15-H_A), 2.02-1.93 (1H, m, 15-H_B), 1.45-1.37 (1H, m, 12-H_B). **^{13}C NMR** (125 MHz, CD_3OD, $\mathit{C}F_3\mathit{C}O_2^-$ and 2 × Ar-C_q not observed): 169.2 (CO_2Me), 162.0 (quinoline 6-C), 148.0 (Ar-C_q), 143.5 (quinoline 2-C), 141.4 (Ar-C_q), 128.7 (Ar-C_q), 128.5 (Ar-C_q), 128.2 (quinoline 7-C), 126.0 (quinoline 8-C), 125.2 (Ar-C_q), 120.5 (quinolone 3-C), 119.5 (Ar-C_q), 119.2 (Ar-C_q), 113.5 (indole 4-C or 6-C), 103.7 (indole 4-C or 6-C), 102.5 (quinoline 5-C), 69.8 (13-C), 68.0 (CH(OH)), 57.6 (OCH_3), 56.2 (OCH_3), 52.6 (OCH_3), 51.2 (14-C), 28.8 (11-C), 27.0 (12-C), 24.7 (15-C). **HRMS** (ESI): $C_{27}H_{28}O_5N_3$ $[M+H]^+$; calculated: 474.2024, found: 474.2020.

[§] Inferred from HSQC analysis.

6.3.2.4 Preparation of the quinine-derived azaindoles

(*R*)-(6-Methoxyquinolin-4-yl)[(*11S*,*13S*)-1,6,9-triazatetracyclo[9.2.2.0^{2,10}.0^{3,8}]pentadeca-2(10),3(8),4,6-tetraen-13-yl]methanol 10v

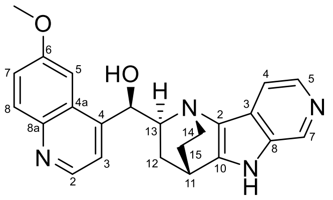

10v

Prepared according to General Procedure B using ketone **6** and 10 mol% Pd(OAc)$_2$ (24 h). Flash column chromatography eluting with 1:9 to 1:4 ({50:80:1 CH$_2$Cl$_2$:EtOH:NH$_4$OH}/CH$_2$Cl$_2$) gave the *title compound* **10v** as a yellow solid (81 mg, 209 μmol, 65%). **^{1}H NMR** (600 MHz, CD$_3$OD, NH and OH not observed): δ 8.65 (1H, d, *J* 4.7, quinoline 2-H), 8.59 (1H, s, indole 7-H), 8.04 (1H, d, *J* 5.7, indole 5-H), 7.87 (1H, d, *J* 9.2, quinoline 8-H), 7.76 (1H, d, *J* 4.7, quinoline 3-H), 7.63 (1H, d, *J* 5.7, indole 4-H), 7.41 (1H, d, *J* 2.6, quinoline 5-H), 7.32 (1H, dd, *J* 9.2, 2.6, quinoline 7-H), 5.99 (1H, app. s, C*H*(OH)), 4.26-4.20 (1H, m, 14-H$_A$), 3.98 (3H, s, OCH$_3$), 3.49 (1H, s, 11-H), 3.05-3.00 (1H, m, 13-H), 2.65-2.59 (1H, m, 14-H$_B$), 2.44-2.39 (1H, m, 12-H$_A$), 2.26-2.20 (1H, m, 15-H$_A$), 1.68-1.61 (1H, m, 15-H$_B$), 1.27-1.20 (1H, m, 12-H$_B$). **^{13}C NMR** (150 MHz, CD$_3$OD): δ 159.7 (quinoline 6-C), 151.0 (Ar-C$_q$), 149.9 (Ar-C$_q$), 148.2 (quinoline 2-C), 144.6 (Ar-C$_q$), 138.1 (indole 5-C), 134.4 (indole 7-C), 132.5 (Ar-C$_q$), 131.3 (quinoline 8-C), 127.9 (Ar-C$_q$), 127.8 (Ar-C$_q$), 126.8 (Ar-C$_q$), 123.6 (quinoline 7-C), 119.4 (quinoline 3-C), 111.9 (indole 4-C), 102.0 (quinoline 5-C), 71.7 (CH(OH)), 66.7 (13-C), 56.6 (O-CH$_3$), 48.3 (14-C), 30.1 (15-C), 29.9 (11-C), 27.6 (12-C). **HRMS** (ESI): C$_{23}$H$_{23}$O$_2$N$_4$ [M+H]$^+$; calculated: 387.1816, found: 387.1816. $[\boldsymbol{\alpha}]_{\mathbf{20}}^{\boldsymbol{D}}$ = +58 (c. 0.1, MeOH).

(*1R*,*11S*,*13S*)-13-[(*R*)-Hydroxy(6-methoxyquinolin-4-yl)methyl]-1,7,9-triazatetracyclo [9.2.2.0^{2,10}.0^{3,8}]pentadeca-2(10),3(8),4,6-tetraen-1-ium trifluoroacetate 10w

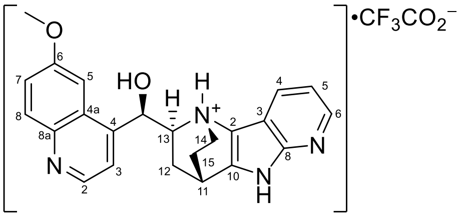

10w

Prepared according to General Procedure B using ketone **6** and 30 mol% $Pd(OAc)_2$ (72 h). Flash column chromatography eluting with 1:9 to 1:4 ({50:80:1 CH_2Cl_2:EtOH:NH_4OH}/CH_2Cl_2) followed by mass-directed preparative HPLC (MeCN in H_2O + 0.1% TFA; Rt = 10.2 min) gave the *title compound* **10w** (21 mg, 43 μmol, 13%) as a colourless solid. **^{1}H NMR** (500 MHz, CD_3OD, NH^+, NH and OH not observed): δ 8.94 (1H, d, *J* 5.5, quinoline 2-H), 8.28-8.23 (3 H, m, indole 4-H, indole 6-H, quinoline 3-H), 8.08 (1H, d, *J* 9.3, quinoline 8-H), 7.74 (1H, d, *J* 2.3, quinoline 5-H), 7.67 (1H, dd, *J* 9.3, 2.3, quinoline 7-H), 7.28 (1H, dd, *J* 7.9, 5.0, indole 5-H), 6.67 (1H, app. s, C*H*(OH)), 4.85-4.78 (1H, m, 14-H_A), 4.08 (3H, s, OCH_3), 3.82 (1H, s, 11-H), 3.79-3.73 (1H, m, 13-H), 3.29-3.26 (1H, m, 14-H_B), 2.68-2.64 (1H, m, 12-H_A), 2.55-2.47 (1H, m, 15-H_A), 2.04-1.94 (1H, m, 15-H_B), 1.52-1.45 (1H, m, 12-H_B). **^{13}C NMR** (125 MHz, CD_3OD, $\mathit{C}F_3CO_2^-$ not observed): δ 162.8 (q, J_{CF} 36.7, $CF_3\mathit{C}O_2^-$). 161.9 (quinoline 6-C), 156.0 (Ar-C_q), 146.5 (Ar-C_q), 143.6 (indole 6-C), 143.4 (quinoline 2-C), 141.6 (Ar-C_q), 137.4 (Ar-C_q), 128.7 (Ar-C_q), 128.1 (quinoline 7-C), 126.6 (indole 4-C), 125.9 (quinoline 8-C), 120.7 (quinoline 3-C), 118.4 (indole 5-C), 116.8 (Ar-C_q), 112.4 (Ar-C_q), 102.5 (quinoline 5-C), 69.2 (13-C), 67.9 (CH(OH)), 57.4 (OCH_3), 51.3 (14-C), 28.6 (11-C), 27.0 (15-C), 24.9 (12-C). **HRMS** (ESI): $C_{23}H_{23}O_2N_4$ $[M+H]^+$; calculated: 387.1816, found: 387.1816. $[\alpha]_{20}^{D}$ = −37 (c. 0.1, MeOH).

(*1R*,*11S*,*13S*)-4-Chloro-13-[(*R*)-hydroxy(6-methoxyquinolin-4-yl)methyl]-1,7,9-triazatetracyclo[9.2.2.0^{2,10}.0^{3,8}]pentadeca-2(10),3(8),4,6-tetraen-1-ium trifluoroacetate 10w-a

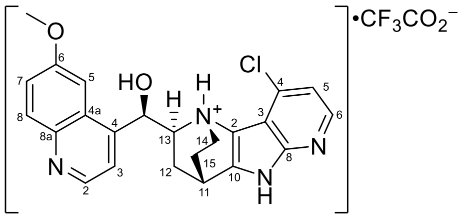

10w-a

Prepared according to General Procedure B using ketone **6**, $MgSO_4$ (1.5 eq.) and 30 mol% $Pd(OAc)_2$ (72 h). Purification by mass-directed HPLC (MeCN in H_2O + 0.1% TFA) gave the *title compound* **10w-a** (32 mg, 59 μmol, 19%) as a pale brown solid. **^{1}H NMR** (700 MHz, CD_3OD, NH^+, NH and OH not observed): δ 8.96 (1H, d, *J* 5.5, quinoline 2-H), 8.27 (1H, d, *J* 5.5, quinoline 3-H), 8.18 (1H, d, *J* 5.3, indole 6-H), 8.12 (1H, d, *J* 9.2, quinoline 8-H), 7.75-7.70 (2H, m, quinoline 5-H and 7-H), 7.31 (1H, d, *J* 5.3, indole 5-H), 6.78 (1H, app. s, C*H*(OH)), 4.83-4.76 (1H, m, 14-H_A), 4.13 (3H, s, OCH_3), 3.88-3.80 (2H, m, 11-H and 13-H), 3.38-3.33 (1H, m, 14-H_B), 2.73 (1H, ddd, *J* 12.3, 6.2, 1.9, 12-H_A), 2.58-2.50 (1H, m, 15-H_A), 2.04-1.97 (1H, m, 15-H_B), 1.54-1.49 (1H, m, 12-H_B). **^{13}C NMR** (176 MHz, CD_3OD, *C*$F_3CO_2^-$ not observed): δ 162.4 (q, *J* 36.2, CF_3*C*O_2^-), 161.7 (quinoline 6-C), 147.5 (Ar-C_q), 144.1 (quinoline 3-C), 143.9 (quinoline-2-C), 143.5 (Ar-C_q), 138.0 (Ar-C_q), 135.8 (Ar-C_q), 133.3 (Ar-C_q), 128.7 (Ar-C_q), 127.6 (quinoline 7-C), 126.4 (quinoline 8-C), 120.7 (indole 6-C), 118.5 (indole 5-C), 114.8 (Ar-C_q), 111.9 (Ar-C_q), 102.5 (quinoline 5-C), 70.2 (13-C), 68.0 (*C*H(OH)), 57.2 (OCH_3), 52.8 (14-C), 28.4 (11-C), 26.6 (15-C), 25.4 (12-C). **HRMS** (ESI): $C_{23}H_{22}O_2N_4{}^{35}Cl$ $[M+H]^+$; calculated: 421.1426, found: 421.1423. $[\alpha]_{20}^{D}$ = +34 (c. 0.1, MeOH).

(*R*)-(6-Methoxyquinolin-4-yl)[(*11S*,*13S*)-5-methyl-1,7,9-triazatetracyclo[9.2.2.0^{2,10}.0^{3,8}] pentadeca-2(10),3(8),4,6-tetraen-13-yl]methanol 10w-b

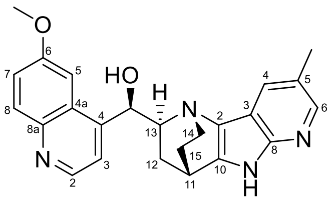

10w-b

Prepared according to General Procedure B using ketone **6** and 30 mol% Pd(OAc)$_2$ (72 h). Flash column chromatography eluting with 1:9 to 1:4 ({50:80:1 CH_2Cl_2:EtOH:NH_4OH}/CH_2Cl_2) gave the *title compound* **10w-b** (39 mg, 97 μmol, 30%) as a yellow solid. **^{1}H NMR** (500 MHz, CD_3OD, NH and OH not observed): δ 8.65 (1H, d, *J* 4.7, quinoline 2-H), 7.91 (1H, s, indole 6-H), 7.87 (1H, d, *J* 9.2, quinoline 8-H), 7.83 (1H, s, indole 4-H), 7.76 (1H, d, *J* 4.7, quinolone 3-H), 7.41 (1H, d, *J* 2.7, quinoline 5-H), 7.32 (1H, dd, *J* 9.2, 2.7, quinoline 7-H), 5.97 (1H, app. s, C*H*(OH)), 4.24-4.17 (1H, m, 14-H_A), 3.99 (3H, s, OCH_3), 3.46-3.42 (1H, m, 11-H), 3.06-2.99 (1H, m, 13-H), 2.68-2.57 (1H, m, 14-H_B), 2.42 (3H, s, Py-CH_3), 2.40-2.35 (1H, m, 12-H_A), 2.23-2.16 (1H, m, 15-H_A), 1.69-1.62 (1H, m, 15-H_B), 1.27-1.19 (1H, m, 12-H_B). **^{13}C NMR** (125 MHz, CD_3OD): δ 159.7 (quinoline 6-C), 151.1 (Ar-C_q), 148.2 (quinoline 2-C), 145.7 (Ar-C_q), 144.7 (Ar-C_q), 144.6 (Ar-C_q), 141.9 (indole 6-C), 131.3 (quinoline 8-C), 127.8 (Ar-C_q), 126.3 (Ar-C_q), 126.1 (Ar-C_q), 125.5 (indole 4-C), 123.6 (quinoline 7-C), 119.4 (quinoline 3-C), 115.6 (Ar-C_q), 101.9 (quinoline 5-C), 71.7 (CH(OH)), 66.9 (13-C), 56.5 (OCH_3), 48.4 (14-C), 30.5 (15-C), 29.3 (11-C), 27.9 (12-C), 18.6 (Py-CH_3). **HRMS** (ESI): $C_{24}H_{25}O_2N_4$ $[M+H]^+$; calculated: 401.1972, found: 401.1972. $[\alpha]_{20}^{D}$ = +46 (c. 0.1, MeOH).

(*R*)-[(*11S*,*13S*)-5-(4-Chlorophenyl)-1,7,9-triazatetracyclo[9.2.2.0^{2,10}.0^{3,8}]pentadeca-2(10),3(8),4,6-tetraen-13-yl](6-methoxyquinolin-4-yl)methanol trifluoroacetate 10w-c

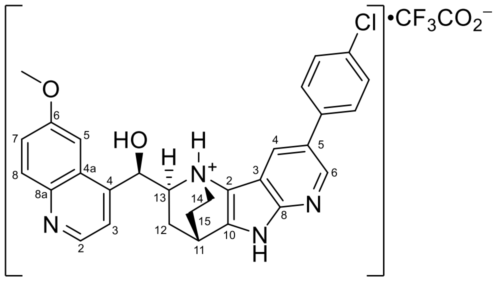

10w-c

Prepared according to General Procedure B using ketone **6** and 20 mol% $Pd(OAc)_2$ (48 h). Flash column chromatography eluting with 5-35% {50:80:1 CH_2Cl_2:EtOH:NH_4OH} in CH_2Cl_2 gave 71 mg of partially purified material (63:37 mixture of target product:starting ketone[‡‡‡] containing 0.10 mmol product, 33% yield). Further purification by mass-directed HPLC (MeCN in H_2O + 0.1% TFA) gave the *title compound* **10w-c** (18 mg, 29 μmol, 9%) as a colourless solid. **^{1}H NMR** (500 MHz, CD_3OD, NH^+, NH and OH not observed): δ 8.95 (1H, d, *J* 5.5, quinoline 2-H), 8.52 (1H, d, *J* 2.1, indole 6-H), 8.46 (1H, d, *J* 2.1, indole 4-H), 8.27 (1H, d, *J* 5.5, quinoline 3-H), 8.09 (1H, d, *J* 9.3, quinoline 8-H), 7.76 (1H, d, *J* 2.6, quinoline 5-H), 7.69-7.62 (3H, m, quinoline 7-H and phenyl 2-H or 3-H), 7.51 (2H, d, *J* 8.4, phenyl 2-H or 3-H), 6.69 (1H, app. s, C*H*(OH)), 4.87-4.81 (1H, m, 14-H_A[§§§]), 4.05 (3H, s, OCH_3), 3.86-3.83 (1H, m, 11-H), 3.81 (1H, dd, *J* 10.5, 6.7, 13-H), 3.39-3.33 (1H, m, 14-H_B[****]), 2.70 (1H, ddd, *J* 12.3, 6.6, 2.3, 12-H_A), 2.58-2.51 (1H, m, 15-H_A), 2.07-1.98 (1H, m, 15-H_B), 1.57-1.50 (1H, m, 12-H_B). **^{13}C NMR** (125 MHz, CD_3OD, $CF_3CO_2^-$ not observed): δ 161.9 (quinoline 6-C), 146.5 (Ar-C_q), 143.5 (quinoline 2-C), 142.7 (indole 6-C), 142.5 (Ar-C_q), 138.6 (Ar-C_q), 134.9 (Ar-C_q), 131.1 (Ar-C_q), 130.3 (phenyl 2-C or 3-C), 129.6 (phenyl 2-C or 3-C), 128.7 (Ar-C_q), 128.0 (quinoline 7-C), 126.0 (quinoline 8-C), 124.3 (indole 4-C), 120.7 (quinoline 3-C), 117.0 (Ar-C_q), 112.2 (Ar-C_q), 102.6 (quinoline 5-C), 69.3 (13-C), 68.0 (CH(OH)), 57.3 (OCH_3), 51.3 (14-C), 28.8 (11-C), 27.1 (15-C), 24.9 (12-C). **HRMS** (ESI): $C_{29}H_{26}O_2N_4{}^{35}Cl$ $[M+H]^+$; calculated: 497.1739, found: 497.1734. $[\alpha]_{20}^{D}$ = +189 (c. 0.1, MeOH).

‡‡‡ As judged by analysis of the partially purified reaction product by ^{1}H NMR spectroscopy at 300 MHz.
§§§ Inferred from HSQC analysis.
**** Inferred from HSQC analysis.

(*1R*,*11S*,*13S*)-13-[(*R*)-Hydroxy(6-methoxyquinolin-4-yl)methyl]-5-(trifluoromethyl)-1,7,9-triazatetracyclo[9.2.2.0^{2,10}.0^{3,8}]pentadeca-2(10),3(8),4,6-tetraen-1-ium trifluoroacetate 10w-d

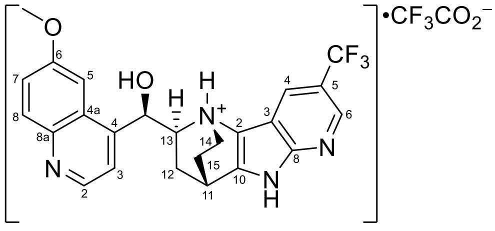

10w-d

Prepared according to General Procedure B using ketone **6**, $MgSO_4$ (1.5 eq.) and 20 mol% $Pd(OAc)_2$ (48 h). Flash column chromatography eluting with 5-20% {50:80:1 CH_2Cl_2:EtOH:NH_4OH} in CH_2Cl_2 gave 51 mg of partially purified material (61:39 mixture of target product:starting ketone†††† containing 76 μmol product, 24% yield). Further purification by mass-directed HPLC (MeCN in H_2O + 0.1% TFA) gave the *title compound* **10w-d** (18 mg, 32 μmol, 10%). **^{1}H NMR** (500 MHz, CD_3OD, NH^+, NH and OH not observed): δ 8.97 (1H, d, *J* 5.7, quinoline 2-H), 8.56-8.51 (2H, m, indole 4-H and 6-H), 8.31 (1H, d, *J* 5.7, quinoline 3-H), 8.10 (1H, d, *J* 9.3, quinolone 8-H), 7.76 (1H, d, *J* 2.5, quinoline 5-H), 7.69 (1H, dd, *J* 9.3, 2.5, quinoline 7-H), 6.73 (1H, app. s, C*H*(OH)), 4.84-4.79 (1H, m, 14-H_A), 3.87-3.83 (1H, m, 11-H), 3.78 (1H, dd, *J* 10.4, 6.8, 13-H), 3.37-3.35 (1H, m, 14-H_B), 2.67 (1H, ddd, *J* 12.4, 6.7, 2.2, 12-H_A), 2.57-2.47 (1H, m, 15-H_A), 2.07-1.96 (2H, m, 15-H_B), 1.53-1.44 (1H, m, 12-H_B). **^{13}C NMR** (125 MHz, CD_3OD): δ 163.1 (q, *J* 35.8, $CF_3\mathit{C}O_2^-$), 162.1 (quinoline 6-C), 156.6 (Ar-C_q), 148.5 (Ar-C_q), 144.3 (Ar-C_q), 143.1 (quinoline 2-C), 140.7 (q, *J* 3.7, indole 6-C), 136.9 (Ar-C_q), 128.8 (Ar-C_q), 128.5 (quinoline 7-C), 126.0 (q, *J* 269.8, $ArCF_3$), 125.4 (quinoline 8-C), 123.7 (q, *J* 3.7, indole 4-C), 121.5 (q, *J* 32.4, indole 5-C), 120.7 (quinoline 3-C), 118.0 (q, *J* 291.3, $\mathit{C}F_3CO_2^-$), 117.4 (Ar-C_q), 111.1 (Ar-C_q), 102.5 (quinoline 5-C), 69.2 (13-C), 68.0 (CH(OH)), 57.4 (OCH_3), 50.9 (14-C), 28.8 (11-C), 27.0 (15-C), 24.7 (12-C). **HRMS** (ESI): $C_{24}H_{22}O_2N_4F_3$ $[M+H]^+$; calculated: 455.1689, found: 455.1685. $[\alpha]_{20}^{D}$ = +17 (c. 0.1, MeOH).

†††† As judged by analysis of the partially purified reaction product by ^{1}H NMR spectroscopy at 300 MHz.

(*1R*,*11S*,*13S*)-13-[(*R*)-Hydroxy(6-methoxyquinolin-4-yl)methyl]-5-nitro-1,7,9-triazatetracyclo[9.2.2.0^{2,10}.0^{3,8}]pentadeca-2(10),3(8),4,6-tetraen-1-ium trifluoroacetate 10w-e

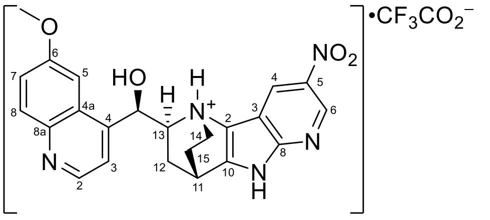

10w-e

Prepared according to General Procedure B using ketone **6**, $MgSO_4$ (1.5 eq.) and 30 mol% $Pd(OAc)_2$ (72 h). Purification by mass-directed HPLC (MeCN in H_2O + 0.1% TFA) gave the *title compound* **10w-e** (18 mg, 33 μmol, 10%). **^{1}H NMR** (700 MHz, CD_3OD, NH^+, NH and OH not observed): δ 9.14 (1H, d, *J* 2.5, indole 6-H), 9.06 (1H, d, *J* 2.5, indole 4-H), 8.96 (1H, d, *J* 5.6, quinoline 2-H), 8.28 (1H, d, *J* 5.6, quinoline 3-H), 8.10 (1H, d, *J* 9.3, quinoline 8-H), 7.78 (1H, d, *J* 2.6, quinoline 5-H), 7.70 (1H, dd, *J* 9.3, 2.5, quinoline 7-H), 6.69-6.62 (1H, m, CH(OH)), 4.81-4.75 (1H, m, 14-H_A), 4.11 (3H, s, OCH_3), 3.86-3.82 (1H, m, 11-H), 3.78-3.73 (1H, m, 13-H), 3.30-3.24 (1H, m, 14-H_B), 2.69 (1H, ddd, *J* 12.4, 6.6, 2.3, 12-H_A), 2.55-2.49 (1H, m, 15-H_A), 2.05-1.98 (1H, m, 15-H_B), 1.56-1.49 (1H, m, 12-H_B). **^{13}C NMR** (175 MHz, CD_3OD, $CF_3CO_2^-$ not observed): δ 162.0 (2 peaks, 2 × Ar-C_q), 149.1 (Ar-C_q), 146.2 (Ar-C_q), 143.3 (quinoline 2-C), 141.3 (Ar-C_q), 139.9 (indole 6-C), 137.1 (Ar-C_q), 128.8 (2 peaks, 2 × Ar-C_q), 128.3 (quinoline 7-C), 125.6 (quinoline 8-C), 122.0 (indole 4-C), 120.7 (quinoline 3-C), 117.7 ($\mathit{C}F_3CO_2^-$), 111.4 (Ar-C_q), 102.7 (quinoline 5-C), 68.9 (13-C), 68.4 (CH(OH)), 57.5 (OCH_3), 50.8 (14-C), 29.0 (11-C), 27.3 (15-C), 25.2 (12-C). **HRMS** (ESI): $C_{23}H_{22}O_4N_5$ $[M+H]^+$; calculated: 432.1666, found: 432.1661. $[\alpha]^D_{20}$ = +59 (c. 0.1, MeOH).

(*1R*,*11S*,*13S*)-5-Fluoro-13-[(*R*)-hydroxy(6-methoxyquinolin-4-yl)methyl]-1,7,9-triaza-tetracyclo[9.2.2.0^{2,10}.0^{3,8}]pentadeca-2(10),3(8),4,6-tetraen-1-ium trifluoroacetate 10w-f

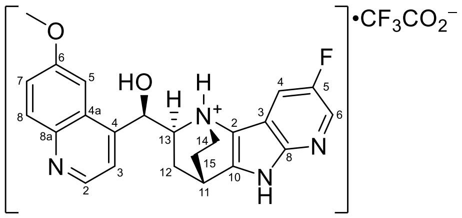

10w-f

Prepared according to General Procedure B using ketone **6** and 30 mol% Pd(OAc)$_2$ (72 h). Flash column chromatography eluting with 1:9 to 1:4 ({50:80:1 CH_2Cl_2:EtOH:NH_4OH}/CH_2Cl_2) followed by mass-directed preparative HPLC (MeCN in H_2O + 0.1% TFA; Rt = 6 min) gave the *title compound* **10w-f** (23 mg, 44 μmol, 14%) as a yellow solid. **^{1}H NMR** (500 MHz, CD_3OD, NH^+, NH and OH not observed): δ 8.90 (1H, d, *J* 5.4, quinoline 2-H), 8.18 (1H, d, *J* 9.1, indole 6-H), 8.16 (1H, d, *J* 9.3, quinoline 8-H), 7.93 (1H, d, *J* 5.4, quinoline 3-H), 7.76 (1H, dd, *J* 9.3, 2.4, quinoline 7-H), 7.59 (1H, d, *J* 2.4, quinoline 5-H), 7.56 (1H, dd, *J* 9.1, 2.4, indole 4-H), 5.87 (1H, d, *J* 4.1, C*H*(OH)), 4.53-4.46 (1H, m, 13-H), 3.99-3.95 (1H, m, 14-H_A), 3.94 (3H, s, OCH_3), 3.82-3.77 (1H, m, 11-H), 3.20-3.13 (1H, m, 14-H_B), 2.27-2.18 (1H, m, 15-H_A), 2.14-2.03 (2H, m, 12-H_A and 12-H_B), 1.91-1.84 (1H, m, 15-H_B). **^{13}C NMR** (125 MHz, CD_3OD): δ 162.72 (q, J_{CF} 38.6, $CF_3CO_2^-$) 161.6 (quinoline 6-C), 157.4 (d, J_{CF} 242.1, indole 5-C), 155.0 (Ar-C_q), 144.1 (Ar-C_q), 143.7 (Ar-C_q), 143.4 (quinoline 2-C), 137.9 (Ar-C_q), 131.8 (d, J_{CF} 30.0, indole 6-C), 131.7 (Ar-C_q), 129.0 (Ar-C_q), 127.8 (quinoline 7-C), 126.1 (quinoline 8-C), 121.6 (quinoline 3-C), 120.1 (q, J_{CF} 265.6, $CF_3CO_2^-$), 113.3 (Ar-C_q), 112.11 (d, J_{CF} 23.1, indole 4-C), 103.3 (quinoline 5-C), 69.4 (CH(OH)), 66.9 (13-C), 56.9 (OCH_3), 56.7 (14-C), 29.7 (12-C), 28.7 (11-C), 25.1 (15-C). **HRMS** (ESI): $C_{23}H_{22}O_2N_4F$ $[M+H]^+$; calculated: 405.1721, found: 405.1723. $[\alpha]_{20}^{D}$ = +83 (c. 0.1, MeOH).

(*R*)-[(*11S*,*13S*)-5-Chloro-1,7,9-triazatetracyclo[9.2.2.0^{2,10}.0^{3,8}]pentadeca-2(10),3(8),4,6-tetraen-13-yl](6-methoxyquinolin-4-yl)methanol 10w-g

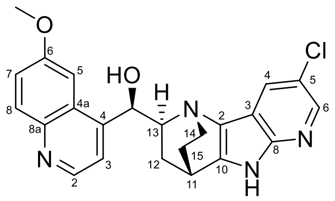

10w-g

Prepared according to General Procedure B using ketone **6** and 20 mol% $Pd(OAc)_2$ (72 h). Flash column chromatography eluting with 5-20% {50:80:1 CH_2Cl_2:EtOH:NH_4OH} in CH_2Cl_2 gave the *title compound* **10w-g** (71 mg, 0.17 mmol, 53%). **^{1}H NMR** (400 MHz, CD_3OD, NH and OH not observed): δ 8.59 (1H, d, *J* 4.7, quinoline 2-H), 7.97-7.92 (2H, m, indole 4-H and 6-H), 7.76 (1H, d, *J* 9.2, quinoline 8-H), 7.70 (1H, d, *J* 4.7, quinoline 3-H), 7.33 (1H, d, *J* 2.7, quinoline 5-H), 7.18 (1H, dd, *J* 9.2, 2.6, quinoline 7-H), 5.92 (1H, s, C*H*(OH)), 4.15 (1H, ddd, *J* 12.8, 8.7, 4.1, 14-H_A), 3.91 (3H, s, OCH_3), 3.41-3.37 (1H, m, 11-H), 3.01-2.96 (1H, m, 13-H), 2.60-2.52 (1H, m, 14-H_B), 2.33 (1H, ddd, *J* 12.3, 6.7, 2.1, 12-H_A), 2.19-2.10 (1H, m, 15-H_A), 1.64-1.55 (1H, m, 15-H_B), 1.27-1.16 (1H, m, 12-H_B). **^{13}C NMR** (175 MHz, CD_3OD): δ 159.6 (quinolone 6-C), 150.8 (Ar-C_q), 148.1 (quinoline 2-C), 146.9 (Ar-C_q), 145.5 (Ar-C_q), 144.5 (Ar-C_q), 139.7 (indole 6-C), 131.2 (quinoline 8-C), 127.8 (Ar-C_q), 126.1 (Ar-C_q), 125.0 (Ar-C_q), 124.3 (indole 4-C), 123.4 (quinoline 7-C), 119.4 (quinoline 3-C), 116.1 (Ar-C_q), 101.9 (quinolone 5-C), 71.6 (CH(OH)), 66.8 (13-C), 56.5 (OCH_3), 48.2 (14-C), 30.4 (15-C), 29.4 (11-C), 27.8 (12-C). **HRMS** (ESI): $C_{23}H_{22}O_2N_4{}^{35}Cl$ $[M+H]^+$; calculated: 421.1426, found: 421.1423. $[\boldsymbol{\alpha}]_{\mathbf{20}}^{\boldsymbol{D}}$ = +74 (c. 0.1, MeOH).

(*1R*,*11S*,*13S*)-5-Bromo-13-[(*R*)-hydroxy(6-methoxyquinolin-4-yl)methyl]-1,7,9-triazatetracyclo[9.2.2.0^{2,10}.0^{3,8}]pentadeca-2(10),3(8),4,6-tetraen-1-ium trifluoroacetate 10w-h

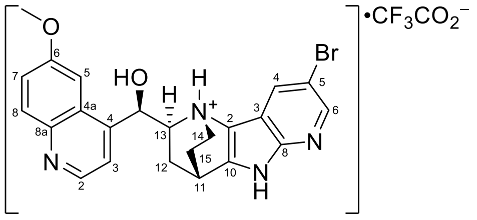

10w-h

Prepared according to General Procedure B using ketone **6**, $MgSO_4$ (1.5 eq.) and 30 mol% $Pd(OAc)_2$ (72 h). Flash column chromatography eluting with 5-20% {50:80:1 CH_2Cl_2:EtOH:NH_4OH} in CH_2Cl_2 gave 43 mg of partially purified material (56:54 mixture of target product:starting ketone‡‡‡‡ containing 56 μmol product, 17% yield). Further purification by mass-directed HPLC (MeCN in H_2O + 0.1% TFA) gave the *title compound* **10w-h** (19 mg, 33 μmol, 10%). **^{1}H NMR** (700 MHz, CD_3OD, NH^+, NH and OH not observed): δ 8.97 (1H, d, *J* 5.7, quinoline 2-H), 8.36 (1H, d, *J* 2.2, indole 4-H), 8.32 (1H, d, *J* 5.7, quinoline 3-H), 8.27 (1H, d, *J* 2.2, indole 6-H), 8.11 (1H, d, *J* 9.3, quinoline 8-H), 7.77 (1H, d, *J* 2.5, quinoline 5-H), 7.71 (1H, dd, *J* 9.3, 2.5, quinoline 7-H), 6.71 (1H, s, CH(OH)), 4.79 (1H, ddd, *J* 11.8, 9.3, 4.3, 14-H_A), 4.09 (3H, s, OCH_3), 3.82-3.78 (1H, m, 11-H), 3.79-3.73 (1H, m, 13-H), 3.33-3.30 (1H, m, 14-H_B), 2.65 (1H, ddd, *J* 12.3, 6.7, 2.4, 12-H_A), 2.53-2.47 (1H, m, 15-H_A), 2.02-1.96 (1H, m, 15-H_B), 1.50-1.44 (1H, m, 12-H_B). **^{13}C NMR** (175 MHz, CD_3OD): δ 162.3 (quinolone 6-C), 162.2 (q, *J* 36.7, $CF_3CO_2^-$), 157.6 (Ar-C_q), 145.6 (Ar-C_q), 144.5 (indole 6-C), 143.2 (Ar-C_q), 142.5 (quinoline 2-C), 135.9 (Ar-C_q), 129.03 (quinoline 7-C), 129.0 (Ar-C_q), 128.3 (indole 4-C), 124.7 (quinoline 8-C), 120.8 (quinoline 3-C), 117.6 (q, *J* 289.3, $CF_3CO_2^-$), 115.9 (Ar-C_q), 114.0 (Ar-C_q), 113.3 (Ar-C_q), 102.7 (quinoline 5-C), 69.1 (13-C), 67.9 (CH(OH)), 57.5 (OCH_3), 51.2 (14-C), 28.7 (11-C), 26.9 (15-C), 24.7 (12-C). **HRMS** (ESI): $C_{23}H_{22}O_2N_4{}^{79}Br$ $[M+H]^+$; calculated: 465.0921, found: 465.0918; $C_{23}H_{22}O_2N_4{}^{81}Br$ $[M+H]^+$; calculated: 467.0900, found: 467.0898. $[\alpha]_{20}^{D}$ = +59 (c. 0.1, MeOH).

‡‡‡‡ As judged by analysis of the partially purified reaction product by ^{1}H NMR spectroscopy at 300 MHz.

(*R*)-[(*11S*,*13S*)-5-Iodo-1,7,9-triazatetracyclo[9.2.2.0^{2,10}.0^{3,8}]pentadeca-2(10),3(8),4,6-tetraen-13-yl](6-methoxyquinolin-4-yl)methanol 10w-i

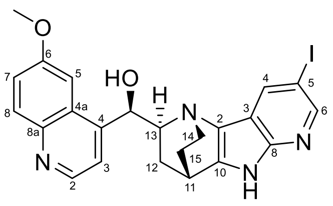

10w-i

Prepared according to General Procedure B using ketone **6**, $MgSO_4$ (1.5 eq.) and 30 mol% $Pd(OAc)_2$ (72 h). Flash column chromatography eluting with 5-20% {50:80:1 CH_2Cl_2:EtOH:NH_4OH} in CH_2Cl_2 gave the *title compound* **10w-i** (9 mg, 18 μmol, 5%). **^{1}H NMR** (700 MHz, CD_3OD, NH and OH not observed): δ 9.14 (1H, d, *J* 2.5, indole 6-H), 9.06 (1H, d, *J* 2.5, indole 4-H), 8.96 (1H, d, *J* 5.6, quinoline 2-H), 8.28 (1H, d, *J* 5.6, quinoline 3-H), 8.10 (1H, d, *J* 9.3, quinoline 8-H), 7.78 (1H, d, *J* 2.6, quinoline 5-H), 7.70 (1H, dd, *J* 9.3, 2.5, quinoline 7-H), 6.65 (1H, br. s, CH(OH)), 4.80-4.74 (1H, m, 14-H_A), 4.11 (3H, s, OCH_3), 3.86-3.81 (1H, m, 11-H), 3.79-3.73 (1H, m, 13-H), 3.30-3.26 (1H, m, 14-H_B), 2.69 (1H, ddd, *J* 12.4, 6.6, 2.3, 12-H_A), 2.58-2.47 (1H, m, 15-H_A), 2.04-1.98 (1H, m, 15-H_B), 1.56-1.47 (1H, m, 12-H_B). **^{13}C NMR** (175 MHz, CD_3OD, 1 × Ar-C_q not observed): δ 162.0 (quinoline 6-C), 149.1 (Ar-C_q), 146.2 (Ar-C_q), 143.3 (quinoline 2-C), 141.3 (Ar-C_q), 139.9 (indole 6-H), 137.1 (Ar-C_q), 128.8 (Ar-C_q), 128.3 (2 peaks, quinoline 7-C and Ar-C_q), 125.6 (quinoline 8-C), 122.0 (indole 4-C), 120.7 (quinoline 3-C), 111.4 (Ar-C_q), 102.7(quinoline 5-C), 68.9 (13-C), 68.4 (CH(OH)), 57.5 (OCH_3), 50.8 (14-C), 29.0 (11-C), 27.3 (15-C), 25.2 (12-C). **HRMS** (ESI): $C_{23}H_{22}O_2N_4I$ $[M+H]^+$; calculated: 513.0782, found: 513.0779. $[\alpha]_{20}^{D}$ = +179 (c. 0.1, MeOH).

(*R*)-[(*11S*,*13S*)-5-Bromo-6-methyl-1,7,9-triazatetracyclo[9.2.2.0^{2,10}.0^{3,8}]pentadeca-2(10),3(8),4,6-tetraen-13-yl](6-methoxyquinolin-4-yl)methanol 10w-j

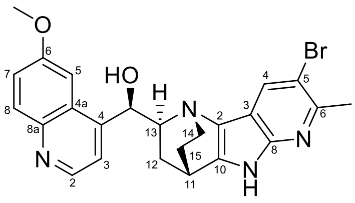

10w-j

Method A: Prepared according to General Procedure B using ketone **6** and 20 mol% $Pd(OAc)_2$ (48 h). Flash column chromatography eluting with 5-35% {50:80:1 CH_2Cl_2:EtOH:NH_4OH} in CH_2Cl_2 gave the *title compound* **10w-j** (29 mg, 60 μmol, 19%). **Method B:** Prepared according to General Procedure B using ketone **6**, $MgSO_4$ (1.5 eq.) and 20 mol% $Pd(OAc)_2$ (48 h). Flash column chromatography eluting with 5-20% {50:80:1 CH_2Cl_2:EtOH:NH_4OH} in CH_2Cl_2 gave the *title compound* **10w-j** (59 mg, 122 μmol, 38%). This material was subjected to mass-directed HPLC to give the *title compound* (51 mg, 86 μmol, 27%) as the TFA salt, which was a yellow oil. **^{1}H NMR** (700 MHz, CD_3OD, free base form, NH and OH not observed): δ 8.65 (1H, d, *J* 4.6, quinoline 2-H), 8.14 (1H, s, indole 4-H), 7.87 (1H, d, *J* 9.3, quinoline 8-H), 7.75 (1H, d, *J* 4.6, quinoline 3-H), 7.40 (1H, s, quinoline 5-H), 7.32 (1H, d, *J* 9.3, quinoline 7-H), 5.96 (1H, s, CH(OH)), 4.22-4.17 (1H, m, 14-H_A), 3.99 (3H, s, OCH_3), 3.47-3.42 (1H, m, 11-H), 3.05-3.00 (1H, m, 13-H), 2.66-2.59 (4H, m, includes 14-H_B and at δ 2.62: 3H, s, $ArCH_3$), 2.37 (1H, dd, *J* 12.1, 6.5, 12-H_A), 2.22-2.17 (1H, m, 15-H_A), 1.70-1.63 (1H, m, 15-H_B), 1.26-1.20 (1H, m, 12-H_B). **^{13}C NMR** (175 MHz, CD_3OD, free base form): δ 159.7 (quinoline 6-C), 151.0 (Ar-C_q), 148.2 (quinoline 2-C), 148.1 (Ar-C_q), 145.8 (Ar-C_q), 145.4 (Ar-C_q), 144.7 (Ar-C_q), 131.6 (quinoline 8-C), 128.7 (indole 4-C), 127.8 (Ar-C_q), 125.8 (Ar-C_q), 123.6 (quinoline 7-C), 119.4 (quinoline 3-C), 115.3 (Ar-C_q), 113.6 (Ar-C_q), 102.0 (quinoline 5-C), 71.7 (CH(OH)), 66.9 (13-C), 56.5 (OCH_3), 48.2 (14-C), 30.5 (15-C), 29.4 (11-C), 27.8 (12-C), 24.7 ($ArCH_3$). **HRMS** (ESI): $C_{24}H_{24}O_2N_4{}^{79}Br$ $[M+H]^+$; calculated: 479.1077, found: 479.1074; $C_{24}H_{24}O_2N_4{}^{81}Br$ $[M+H]^+$; calculated: 481.1057, found: 481.1053. $[\alpha]_{20}^{D}$ = +78 (c. 0.1, MeOH).

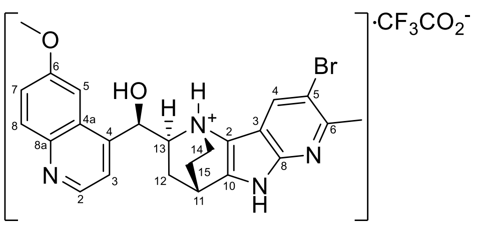

10w-j

^{1}H NMR (500 MHz, CD_3OD, **TFA salt form**, NH^+, NH and OH not observed): δ 8.93 (1H, d, *J* 5.7, quinoline 2-H), 8.31 (1H, s, indole 4-H), 8.27 (1H, d, *J* 5.7, quinoline 3-H), 8.07 (1H, d, *J* 9.3, quinoline 8-H), 7.72 (1H, d, *J* 2.6, quinoline 5-H), 7.68 (1H, dd, *J* 9.3, 2.6, quinoline 7-H), 6.65 (1H, s, CH(OH)), 4.75 (1H, ddd, *J* 11.8, 9.3, 4.3, 14-H_A), 4.06 (3H, s, OCH_3), 3.78-3.66 (2H, m, 11-H and 13-H), 3.32-3.22 (14-H_B)[§], 2.64-2.58 (4H, m, includes 12-H_A, and at δ 2.62: 3H, s, $ArCH_3$), 2.49-2.42 (1H, m, 15-H_A), 2.00-1.89 (1H, m, 15-H_B), 1.47-1.38 (1H, m, 12-H_B). **^{13}C NMR** (175 MHz, CD_3OD, TFA salt form, one Ar-C_q not observed): δ 160.9 (q, *J* 36.5, $CF_3CO_2^-$), 160.8 (quinoline 6-C), 155.9 (Ar-C_q), 149.8 (Ar-C_q), 144.2 (Ar-C_q), 141.2 (quinoline 2-C), 140.6 (Ar-C_q), 134.8 (Ar-C_q), 128.1 (indole 4-C),

[§] Inferred from HSQC analysis.

127.5 (quinoline 7-C), 123.5 (quinoline 8-C), 119.4 (quinoline 3-C), 116.3 (q, *J* 290, $\mathit{C}F_3CO_2^-$), 114.2 (Ar-C_q), 113.5 (Ar-C_q), 110.2 (Ar-C_q), 101.3 (quinoline 5-C), 67.8 (13-C), 66.5 (CH(OH)), 56.1 (OCH_3), 49.8 (14-C), 27.3 (11-C), 25.6 (15-C), 23.7 ($ArCH_3$), 23.3 (12-C).

(*1R*,*11S*,*13S*)-13-[(*R*)-Hydroxy(6-methoxyquinolin-4-yl)methyl]-6-methyl-1,7,9-triaza-tetracyclo[9.2.2.0^{2,10}.0^{3,8}]pentadeca-2(10),3(8),4,6-tetraen-1-ium trifluoroacetate 10w-k

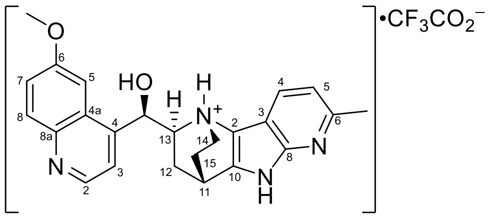

10w-k

Prepared according to General Procedure B using ketone **6** and 30 mol% Pd(OAc)$_2$ (72h). Flash column chromatography eluting with 1:9 to 1:4 ({50:80:1 CH_2Cl_2:EtOH:NH_4OH}/CH_2Cl_2), followed by mass-directed preparative HPLC (MeCN in H_2O + 0.1% TFA; Rt = 8.4 min) gave the *title compound* **10w-k** (5 mg, 10 μmol, 3%) as a yellow solid. **^{1}H NMR** (700 MHz, CD_3OD, NH^+, NH and OH not observed): δ 8.84 (1H, d, *J* 5.2, quinoline 2-H), 8.09 (1H, d, *J* 8.2, quinoline 8-H), 8.05 (1H, d, *J* 5.2, quinoline 3-H), 8.02 (1H, d, *J* 8.8, indole 4-H), 7.60-7.56 (2H, m, quinoline 5-H and indole 5-H), 7.17 (1H, d, *J* 8.2, quinoline 7-H), 6.46 (1H, app. s, C*H*(OH)), 4.80-4.74 (1H, m, 14-H_A), 4.03 (3H, s, OCH_3), 3.80-3.77 (1H, m, 11-H), 3.72-3.67 (1H, m, 13-H), 3.27-3.20 (1H, m, 14-H_B), 2.69-2.61 (1H, m, 12-H_A), 2.59 (3H, s, Py-CH_3), 2.51-2.45 (1H, m, 15-H_A), 1.98-1.92 (1H, m, 15-H_B), 1.52-1.47 (1H, m, 12-H_B). **^{13}C NMR** (125 MHz, CD_3OD, *C*$F_3CO_2^-$ not observed): δ 162.7 (q, J_{CF} 35.5, CF_3*C*O_2^-), 161.1 (quinoline 6-C), 145.6 (quinoline 2-C), 140.7 (Ar-C_q), 128.5 (Ar-C_q), 128.1 (indole 4-C), 126.8 (quinoline 8-C), 126.0 (indole 6-C), 120.4 (quinoline 3-C), 118.8 (Ar-C_q), 118.4 (quinoline 7-C), 117.2 (Ar-C_q), 116.8 (Ar-C_q), 110.0 (Ar-C_q), 102.3 (quinoline 5-C), 69.5 (11-C), 68.3 (CH(OH)), 57.0 (OCH_3), 51.5 (14-C), 28.6 (11-C), 27.3 (15-C), 25.3 (12-C), 23.6 (Py-CH_3). **HRMS** (ESI): $C_{24}H_{25}O_2N_4$ $[M+H]^+$; calculated: 401.1972, found: 401.1972. $[\alpha]_{20}^{D}$ = −48 (c. 0.1, MeOH).

(*1R*,*11S*,*13S*)-6-Chloro-13-[(*R*)-hydroxy(6-methoxyquinolin-4-yl)methyl]-1,7,9-triazatetracyclo[9.2.2.0^{2,10}.0^{3,8}]pentadeca-2(10),3(8),4,6-tetraen-1-ium trifluoroacetate 10w-l

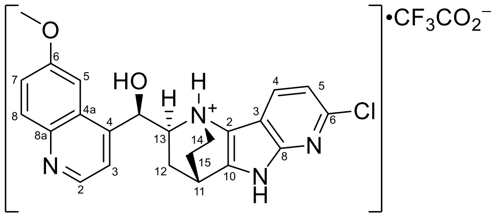

10w-l

Prepared according to General Procedure B using ketone **6** and 20 mol% $Pd(OAc)_2$ (48 h). Flash column chromatography eluting with 15-35% {50:80:1 CH_2Cl_2:EtOH:NH_4OH} in CH_2Cl_2 gave the *title compound* **10w-l** (56 mg, 0.13 mmol, 41%, ~90% purity[§§§§]). Further purification of this material by mass-directed HPLC (MeCN in H_2O + 0.1% TFA) gave the *title compound* **10w-l** (25 mg, 47 μmol, 15%) as the TFA salt. **^{1}H NMR** (700 MHz, CD_3OD, NH^+, NH and OH not observed): δ 8.96 (1H, d, *J* 5.6, quinoline 2-H), 8.27 (1H, d, *J* 5.6, quinoline 3-H), 8.18 (1H, d, *J* 5.3, indole 4-H), 8.12 (1H, d, *J* 9.1, quinoline 8-H), 7.75-7.69 (2H, m, quinoline 5-H and 7-H), 7.31 (1H, d, *J* 5.3, indole 5-H), 6.78 (1H, s, CH(OH)), 4.82-4.76 (1H, m, 14-H_A), 4.13 (3H, s, OCH_3), 3.87-3.79 (2H, m, 11-H and 13H), 3.39-3.33 (1H, m, 14-H_B), 2.73 (1H, ddd, *J* 12.7, 6.3, 2.6, 12-H_A), 2.57-2.50 (1H, m, 15-H_A), 2.04-1.97 (1H, m, 15-H_B), 1.56-1.46 (1H, m, 12-H_B). **^{13}C NMR** (175 MHz, CD_3OD, $\mathit{C}F_3\mathit{C}O_2^-$ and 1 × Ar-C_q not observed): δ 161.9 (quinoline 6-C), 147.5 (Ar-C_q), 144.2 (indole 4-C), 143.4 (2 peaks, quinoline 2-C and Ar-C_q), 137.2 (Ar-C_q), 133.3 (Ar-C_q), 128.8 (Ar-C_q), 128.1 (quinoline 7-C), 125.8 (2 peaks, quinoline 8-C)), 120.7 (quinoline 3-C), 118.6 (indole 5-C), 114.8 (Ar-C_q), 111.8 (Ar-C_q), 102.6 (quinoline 5-C), 70.2 (13-C), 67.9 (CH(OH)), 57.3 (OCH_3), 53.0 (14-C), 28.4 (11-C), 26.4 (15-C), 25.2 (12-C). **HRMS** (ESI): $C_{23}H_{22}O_2N_4{}^{35}Cl$ [M+H]$^+$; calculated: 421.1426, found: 421.1422. $[\boldsymbol{\alpha}]_{\mathbf{20}}^{\boldsymbol{D}}$ = −25 (c. 0.1, MeOH).

[§§§§] As judged by analysis of the product by ^{1}H NMR spectroscopy at 300 MHz.

6.3.2.5 Preparation of compounds to investigate the minimum necessary pharmacophore

6.3.2.5.1 Preparation of compound S-1

(*R*)-[(13*S*)-5-Bromo-6-methyl-1,9-diazatetracyclo[9.2.2.0^{2,10}.0^{3,8}]pentadeca-2(10),3,5,7-tetraen-13-yl](6-methoxyquinolin-4-yl)methyl S-1

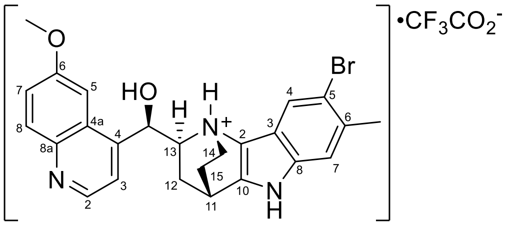

S-1

Prepared according to General Procedure B using ketone **6** and 20 mol% $Pd(OAc)_2$ (48 h). Flash column chromatography eluting with 20% {50:80:1 CH_2Cl_2:EtOH:NH_4OH} in CH_2Cl_2 followed by mass-directed preparative HPLC (MeCN in H_2O + 0.1% TFA; Rt = 15.0 min) gave the *title compound* **S-1** (36 mg, 75 μmol, 23%) as a yellow solid. **^{1}H NMR** (700 MHz, CD_3OD, NH^+, NH and OH not observed) δ 8.89 (d, *J* 5.4, 1H, quinoline 2-H), 8.16 (d, *J* 5.4, 1H, quinoline 3-H), 8.05 (d, *J* 9.3, 1H, quinoline 8-H), 8.00 (s, 1H, indole 4-H), 7.66 (d, *J* 2.3, 1H, quinoline 5-H), 7.61 (dd, *J* 9.3, 2.3, 1H, quinoline 7-H), 7.36 (s, 1H, indole 7-H), 6.60 (s, 1H, app. s, C*H*(OH)), 4.82-4.77 (m, 1H, 14-H_A), 4.09 (s, 3H, OCH_3), 3.75 (s, 1H, app. s, 11-H), 3.70-3.65 (m, 1H, 13-H), 3.30-3.27 (m, 1H, 14-H_B), 2.64-2.60 (m, 1H, 12-H_A), 2.50-2.43 (m, 4H, CH_3 and 15-H_A), 2.00-1.93 (m, 1H, 15-H_B), 1.44-1.39 (m, 1H, 12-H_B). **^{13}C NMR** (176 MHz, CD_3OD, *C*$F_3CO_2^-$ not observed) δ 162.9 (q, J_{CF} 35.8, CF_3*C*O_2^-), 161.6 (quinoline 6-C), 154.4 (Ar-C_q), 144.3 (quinoline 2-C), 141.0 (Ar-C_q), 138.7 (Ar-C_q), 135.2 (Ar-C_q), 132.3 (Ar-C_q), 128.4 (Ar-C_q), 127.4 (quinoline 7-C), 127.0 (quinoline 8-C), 120.4 (indole 4-C or quinoline 3-C), 120.3 (indole 4-C or quinoline 3-C), 118.5 (Ar-C_q), 118.3 (Ar-C_q), 117.1 (Ar-C_q), 114.9 (indole 7-C), 102.3 (quinoline 5-C), 69.8 (13-C), 68.0 (*C*H(OH)), 57.2 (O*C*H_3), 51.3 (14-C), 28.9 (11-C), 27.1 (15-C), 24.8 (12-C), 23.6 (*C*H_3). **HRMS** (ESI); $C_{25}H_{24}O_2N_3{}^{79}Br$ [M+H]$^+$; calculated: 478.1125, found: 478.1122; $C_{25}H_{24}O_2N_3{}^{81}Br$ [M+H]$^+$; calculated: 480.1104, found: 478.1100. $[\alpha]_D^{20}$ −10 (c. 0.1, MeOH).

6.3.2.5.2 Preparation of compound S-2

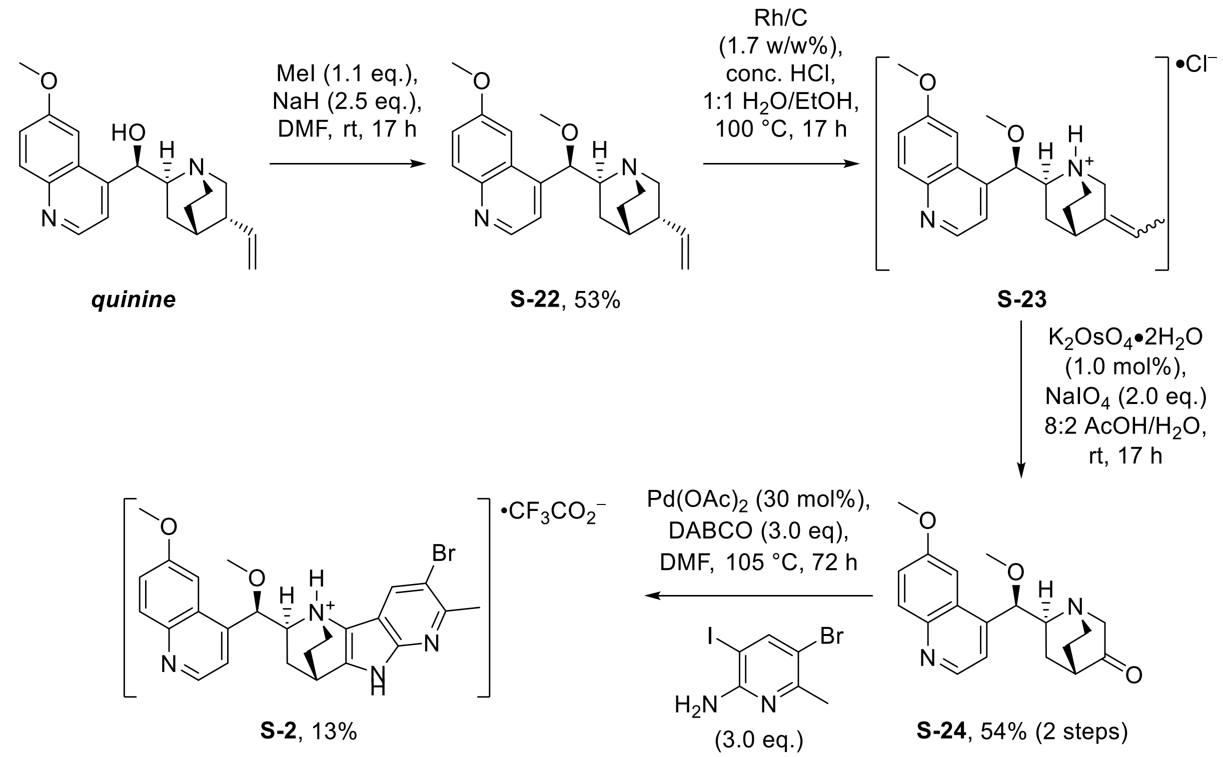

quinine **S-22**, 53% **S-23** **S-24**, 54% (2 steps) **S-2**, 13%

4-[(*R*)-[(*1S*,*2S*,*4S*,*5R*)-5-Ethenyl-1-azabicyclo[2.2.2]octan-2-yl](methoxy)methyl]-6-methoxyquinoline S-22

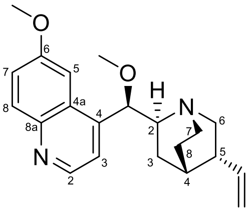

S-22

To a stirred solution of quinine (2.0 g, 6.2 mmol, 1.0 eq.) in anhydrous DMF (20 mL) was added portionwise NaH (60% dispersion in mineral oil, 618 mg, 15.5 mmol, 2.5 eq.) at rt. The reaction mixture was stirred for 1 h, then MeI (430 µl, 6.9 mmol, 1.1 eq.) was added dropwise. The reaction mixture was stirred for 17 h, then quenched with sat. aq. brine solution (20 mL). The resulting solution was extracted with EtOAc (3 × 50 mL), then the combined organics were dried over $MgSO_4$, filtered, and concentrated *in vacuo*. Flash column chromatography eluting with 9:1 EtOAc–MeOH gave the title product as an off-white solid (1.11 g, 3.3 mmol, 53%). **^{1}H NMR** (500 MHz, CD_3OD): δ 8.65 (1H, d, *J* 4.6, quinoline 2-H), 7.94 (1H, d, *J* 9.8, quinoline 8-H), 7.54 (1H, d, *J* 4.6, quinoline 3-H), 7.44-7.38 (2H, m, quinoline 5-H and 7-H), 5.70 (1H, ddd, *J* 17.1, 10.4, 7.5, CHC*H*=CH_2), 5.10 (1H, d, *J* 3.0, C*H*(OCH_3)), 4.93 (1H, dt, *J* 17.2, 1.5, CHCH=$CH_{cis}H_{trans}$), 4.88-4.83 (1H, m, CHCH=$CH_{cis}H_{trans}$), 3.95 (3H, s, OCH_3), 3.52-3.45 (1H, m, 6-H_A), 3.31 (OCH_3), 3.13-3.04 (2H, m, 2-H and 7-H_A), 2.78-2.69 (1H, m, 6-H_B), 2.64 (1H, ddd, *J* 13.6, 5.0, 2.6, 7-H_B), 2.36-2.29 (1H, m, 5-H), 1.87-1.74 (3H, m, 3-H_A, 4-H, and 8-H_A), 1.62-1.54 (1H, m, 8-H_B), 1.51-1.44 (1H, m, 3-H_B). **^{13}C NMR** (125 MHz, CD_3OD): δ 159.9 (quinoline 6-C), 148.2 (quinoline 2-C), 146.4 (Ar-C_q), 145.1 (Ar-C_q), 142.5 (*C*H=CH_2), 131.6 (quinoline 8-C), 128.9 (Ar-C_q), 123.6 (quinoline 7-C), 120.3 (quinoline 3-C), 115.1 (CH=*C*H_2), 102.3 (quinoline 5-C), 83.2 (*C*H(OCH_3)), 61.0 (2-C), 57.5 (7-C or OCH_3), 57.4 (7-C or OCH_3), 56.5 (OCH_3), 44.2 (6-C), 40.8 (5-C), 29.1 (4-C), 28.1 (8-C), 22.3 (3-C). **HRMS** (ESI): $C_{21}H_{27}O_2N_2$ $[M+H]^+$; calculated: 339.2067, found: 339.2067. $[\alpha]_{20}^{D}$ = −209 (c. 0.1, MeOH).

(*1S*,*4S*,*6S*)-6-[(*R*)-Methoxy(6-methoxyquinolin-4-yl)methyl]-1-azabicyclo[2.2.2]octan-3-one S-24

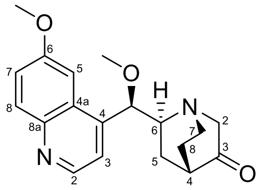

S-24

General procedure A, Part I, was followed using compound **S-22** (1.10 g, 3.25 mmol, 1.0 eq.) to give **S-23** (1.3 g[*****]) as a pale yellow solid which was carried forward to the next step without further purification. General Procedure A, Part II, was followed using compound **S-23** (1.3 g, assume 3.25 mmol). Flash column chromatography eluting with 5-35% {50:80:1 CH_2Cl_2:EtOH:$NH_4OH_{(sat. aq.)}$} in CH_2Cl_2 gave the *title product* **S-24** (568 mg, 1.74 mmol, 54%) as a yellow solid. **^{1}H NMR** (500 MHz, CD_3OD, not observed): δ 8.62 (1H, d, *J* 4.6, quinoline 2-H), 7.89 (1H, d, *J* 9.2, quinoline 8-H), 7.50 (1H, d, *J* 4.6, quinoline 3-H), 7.41 (1H, d, *J* 2.7, quinoline 5-H), 7.34 (1H, dd, *J* 9.3, 2.7, quinoline 7-H), 5.14 (1H, d, *J* 4.1, C*H*(OCH_3)), 3.92 (3H, s, OCH_3), 3.61-3.53 (1H, m, 7-H_A), 3.29 (3H, s, OCH_3), 3.22-3.10 (2H, m, 2-H_A and 6-H), 2.84-2.58 (2H, m, 2-H_B and 7-H_B), 2.31-2.23 (2H, m, 4-H and 5-H_A), 2.12-2.02 (1H, m, 8-H_A), 1.89-1.71 (1H, m, 8-H_B), 1.69-1.57 (1H, m, 5-H_B). **^{13}C NMR** (125 MHz, CD_3OD, 3-C not observed): δ 159.7 (quinoline 6-C), 148.2 (quinoline 2-C), 146.5 (Ar-C_q), 145.0 (Ar-C_q), 131.5 (quinoline 8-C), 128.9 (Ar-C_q), 123.6 (quinoline 7-C), 120.2 (quinoline 3-C), 102.5 (quinoline 5-C), 82.9 (*C*H(OCH_3)), 65.3 (2-C), 61.1 (6-C), 57.5 (OCH_3), 56.4 (OCH_3), 43.4 (7-C), 41.6 (4-C), 26.8 (5-C), 25.7 (8-C). **HRMS** (ESI): $C_{19}H_{23}O_3N_2$ $[M+H]^+$; calculated: 327.1703, found: 327.1700. $[\alpha]_{20}^{D}$ = −125 (c. 0.1, MeOH).

[*****] Maximum theoretical yield = 1.22 g.

(*1R*,*11S*,*13S*)-5-Bromo-13-[(*R*)-methoxy(6-methoxyquinolin-4-yl)methyl]-6-methyl-1,7,9-triazatetracyclo[9.2.2.0^{2,10}.0^{3,8}]pentadeca-2(10),3(8),4,6-tetraen-1-ium trifluoroacetate S-2

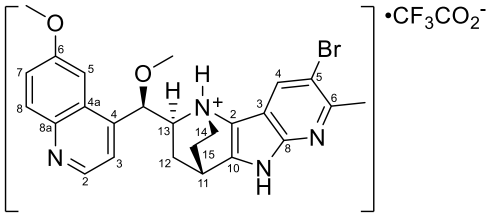

S-2

Prepared according to General Procedure B using ketone **S-24** and 30 mol% Pd(OAc)$_2$ (72h). Flash column chromatography eluting with 1:19 to 1:4 ({50:80:1 CH_2Cl_2:EtOH:NH_4OH}/CH_2Cl_2), followed by mass-directed preparative HPLC (MeCN in H_2O + 0.1% TFA; Rt = 14 min) gave the *title compound* **S-2** (20 mg, 40 µmol, 13%) as a yellow solid. **^{1}H NMR** (700 MHz, CD_3OD, NH and NH^+ not observed) δ 8.93 (1H, d, *J* 5.4, quinoline 2-H), 8.41 (1H, indole 4-H), 8.08 (1H, d, *J* 9.2, quinoline 8-H), 8.06 (1H, d, *J* 5.4, quinoline 3-H), 7.71 (1H, d, 2.3, quinoline 5-H), 7.67 (1H, dd, *J* 9.2, 2.3, quinoline 7-H), 6.30 (1H, app. s, C*H*(OCH_3)), 4.54-4.46 (1H, m, 14-H_A), 4.10 (3H, s, OCH_3), 3.77 (1H, app. s, 11-H), 3.72-3.68 (1H, m, 13-H), 3.62 (3H, s, OCH_3), 3.30-3.24 (1H, m, 14-H_B), 2.66 (3H, s, CH_3), 2.65-2.61 (1H, m, 12-H_A), 2.48-2.42 (1H, m, 15-H_A), 2.37-2.33 (1H, m, 15-H_B), 1.98 (1H, d, *J* 11.7, 12-H_B). **^{13}C NMR** (176 MHz, CD_3OD, *C*$F_3CO_2^-$ not observed) δ 162.5 (q, J_{CF} 35.6, CF_3*C*O_2^-), 161.9 (quinoline 6-C), 151.1 (Ar-C_q), 145.7 (Ar-C_q), 144.1 (quinoline 2-C), 141.9 (Ar-C_q), 138.6 (Ar-C_q), 129.6 (indole 4-C), 127.8 (quinoline 7-C), 126.7 (quinoline 8-C), 120.4 (quinoline 3-C), 116.9 (Ar-C_q), 114.8 (Ar-C_q), 111.7 (Ar-C_q), 109.1 (Ar-C_q), 102.6 (quinoline 5-C), 93.6 (Ar-C_q), 78.16 (*C*H(OCH_3)), 68.7 (13-C), 58.1 (OCH_3), 57.5 (OCH_3), 51.1, (14-C), 28.6 (11-C), 27.1 (15-C), 25.5 (12-C), 25.1 (CH_3). **HRMS** (ESI): $C_{25}H_{26}O_2N_4{}^{79}Br$ $[M+H]^+$; calculated: 493.1234, found: 493.1229; $C_{25}H_{26}O_2N_4{}^{81}Br$ $[M+H]^+$; calculated: 495.1213, found: 495.1208. $[\alpha]_{20}^{D}$ −10 (c. 0.1, MeOH).

6.3.2.5.3 Preparation of compounds S-3 and S-4

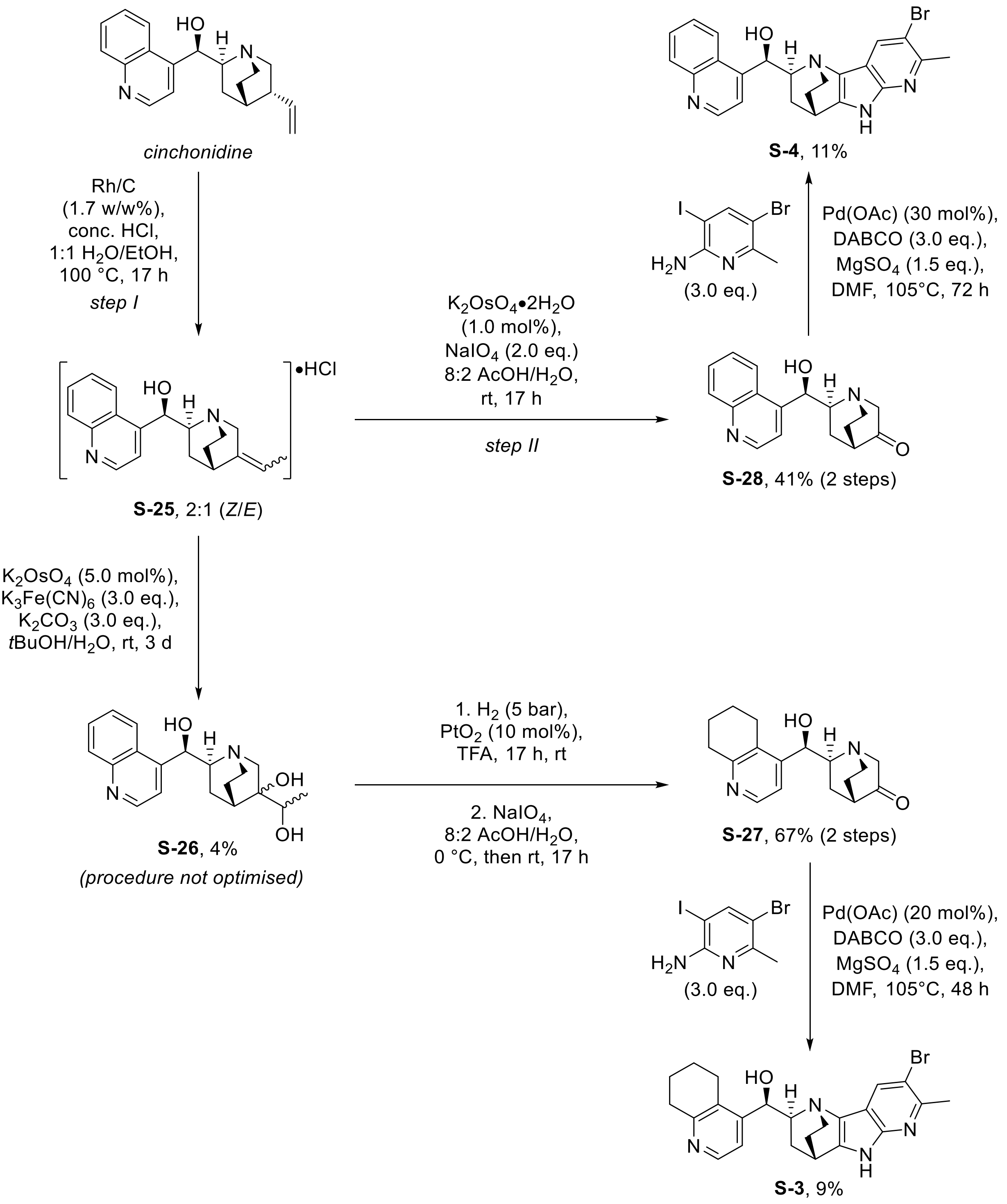

(1*S*,2*S*,4*S*)-5-Ethylidene-2-[(*R*)-hydroxy(quinolin-4-yl)methyl]-1-azabicyclo[2.2.2]octan-1-ium chloride S-25

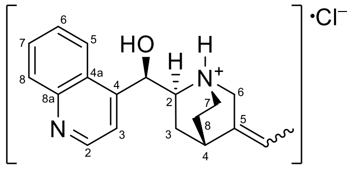

S-25

General procedure A, Part I, was followed using cinchonidine (6.0 g, 20.4 mmol) to give the title product **S-25** (8.1 g†††††) as a pale yellow solid which was carried forward to the next step without further purification.**^{1}H NMR** (700 MHz, CD_3OD, 2:1 mixture of Z/E alkenes, NH^+ and OH not observed) δ 9.28 (1H, app. s, quinoline 2-H), 8.91 (0.66H, d, *J* 8.0, major quinoline 8-H), 8.88 (0.33H, d, *J* 8.0, minor quinoline 8-H), 8.42 (1H, app. s, quinoline 3-H), 8.36 (1H, d, *J* 8.0, quinoline 5-H), 8.24 (1H, app. s, quinoline 6-H), 8.13-8.06 (1H, m, quinoline 7-H), 6.62 (1H, d, *J* 5.4, C*H*(OH)), 5.50 (0.66H, d, *J* 5.6, major C=C*H*CH_3), 5.44 (0.33H, d, *J* 4.9, minor C=C*H*CH_3), 4.40 (1H, app. s, quinuclidine 7-H_A), 4.28-3.99 (2H, m, quinuclidine 6-H_A and quinuclidine 6-H_B), 3.82 (0.66H, app. s, major quinuclidine 2-H), 3.76 (0.33H, app. s, minor quinuclidine 2-H), 3.43 (1H, d, *J* 9.3, quinuclidine 7-H_B), 3.15 (0.66H, s, minor quinuclidine 4-H), 2.70 (0.33H, s, major quinuclidine 4-H), 2.45 (1H, s, quinuclidine 3-H_A), 2.25 (1H, d, *J* 12.0, quinuclidine 8-H_A), 1.96 (1H, d, *J* 12.0, quinuclidine 8-H_B), 1.61 (2H, d, *J* 4.9, minor CH_3), 1.54 (1H, d, *J* 5.6, major CH_3), 1.48 (1H, app. s, quinuclidine 3-H_B). **^{13}C NMR** (176 MHz, CD_3OD, 2:1 mixture of Z/E alkenes): δ 160.7 (Ar-C_q), 160.6 (Ar-C_q), 145.6 (quinoline 2-C), 138.9 (Ar-C_q), 138.9 (Ar-C_q), 136.3 (quinoline 6-C), 132.1 (quinoline 7-C), 131.8 (Ar-C_q), 130.9 (Ar-C_q), 127.2 (major quinoline 8-C), 127.2 (minor quinoline 8-C), 126.3 (Ar-C_q), 126.2 (Ar-C_q), 122.5 (minor quinoline 5-C), 122.5 (major quinoline 5-C), 121.3 (minor quinoline 3-C), 121.2 (major quinoline 3-C), 120.9 (C=*C*HCH_3), 68.4 (minor *C*H(OH)), 68.4 (major *C*H(OH)), 62.4 (major quinuclidine 2-C), 62.2 (minor quinuclidine 2-C), 58.4 (minor quinuclidine 6-C), 56.7 (major quinuclidine 6-C), 46.2 (major quinuclidine 7-C), 46.1 (minor quinuclidine 7-C), 32.6 (major quinuclidine 4-C), 25.8 (minor quinuclidine 4-C), 25.5 (minor quinuclidine 8-C), 24.8 (major quinuclidine 8-C), 24.5 (minor quinuclidine 3-C), 24.1 (major quinuclidine 3-C), 13.0 (minor CH_3), 12.8 (major CH_3). **HRMS** (ESI): calc. for $[M+H]^+$ $C_{19}H_{23}ON_2$: 295.1849 found 295.18039.

††††† Maximum theoretical yield = 6.8 g.

(*1S*,*4S*,*6S*)-6-[(*R*)-Hydroxy(quinolin-4-yl)methyl]-3-(1-hydroxyethyl)-1-azabicyclo[2.2.2]octan-3-ol S-26

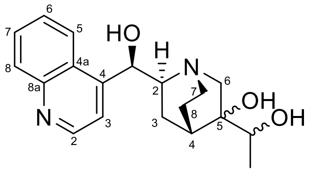

S-26

To a stirred solution of compound **S-25** (free base form,‡‡‡‡‡ 2.0 g, 6.8 mmol) in ^{t}BuOH/H_2O (36 mL, 0.2 M) was added was K_2CO_3 (2.9 g, 20.4 mmol, 3.0 eq.) and $K_3Fe(CN)_6$ (6.7 g, 20.4 mmol, 3.0 eq.) The mixture was stirred for 45 min, then OsO_4 (4.0% in H_2O, 432 μL, 68 μmol, 1.0 mol%) was added. The reaction mixture was stirred for 6 h. Due to the poor conversion observed,§§§§§ K_2OsO_4 (75 mg, 0.20 mmol, 4.0 mol%) was added. The reaction mixture was stirred for an additional 3 days. The reaction mixture was quenched by the addition of $Na_2S_2O_5$ (1.0 g). Sat. aq. $NaHCO_3$ solution (100 mL) was added and the reaction mixture was extracted with 9:1 $CHCl_3$/MeOH (4 × 100 mL). The combined organics were dried, filtered, and concentrated *in vacuo*. Flash column chromatography eluting with 50-100% {50:8:1 CH_2Cl_2:EtOH:NH_4OH} in CH_2Cl_2 gave the *title compound* **S-26** (97 mg, 0.30 mmol, 4%, mixture of 4 diastereomers) as a pale brown oil. **^{1}H NMR** (700 MHz, CD_3OD, **characteristic peaks given**, see Section 7.0 for the processed NMR): δ 8.78-8.73 (1H, m, Ar-H), 8.17 (1H, d, *J* 8.4, Ar-H), 7.99 (1H, d, *J* 8.4, Ar-H), 7.73-7.65 (2H, m, Ar-H), 7.61-7.54 (1H, m, Ar-H), 5.67-5.60 (1H, m, ArC*H*(OH)), 1.05 (0.33H, d, *J* 6.4, diastereomer-1, CH_3), 1.01 (0.33H, d, *J* 6.3, diastereomer-2, CH_3), 1.00 (0.17H, d, *J* 6.3, diastereomer-3, CH_3), 0.99 (0.17H, d, *J* 6.4, diastereomer-4, CH_3). **^{13}C NMR** (175 MHz, CD_3OD): δ 152.4 (2 peaks), 152.3 (2 peaks), 150.90 (2 peaks), 150.8 (2 peaks), 148.8, 148.7 (2 peaks), 130.6 (3 peaks), 130.0 (2 peaks), 129.9 (2 peaks), 128.1 (2 peaks), 128.0, 127.1 (3 peaks), 124.6 (2 peaks), 124.5, 119.9 (2 peaks), 119.8 (2 peaks), 74.4, 74.2, 74.1, 73.9, 72.7, 72.6, 72.1, 72.3, 72.1, 72.0, 71.5, 71.3, 70.9, 69.8, 65.9, 65.1, 63.5, 63.1, 61.4, 61.1, 60.5, 60.4, 43.8, 43.7 (2 peaks), 43.5, 31.7, 31.3, 30.5, 30.1, 23.7, 23.6, 23.5, 23.4, 22.8, 22.6, 22.2 (2 peaks), 17.6, 17.5, 16.2, 16.0. **HRMS** (ESI): $C_{19}H_{25}O_3N_2$ $[M+H]^+$; calculated: 329.1860, found: 329.1860.

‡‡‡‡‡ Prepared by passing a sample of compound **S-25** through a SiO_2 plug eluting with 1:1 (50:8:1 CH_2Cl_2:EtOH:NH4OH):CH_2Cl_2.
§§§§§ As judged by analysis of the crude reaction mixture using LCMS.

(*1S*,*4S*,*6S*)-6-[(*R*)-Hydroxy(5,6,7,8-tetrahydroquinolin-4-yl)methyl]-1-azabicyclo[2.2.2]octan-3-one S-27

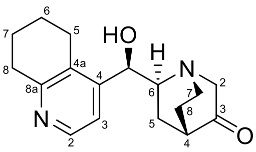

S-27

Compound **S-26** (70 mg, 0.21 mmol) was dissolved in TFA (2.0 mL) and PtO_2 (5 mg, 21 μmol, 10 mol%) was added. The reaction mixture was stirred under an atmosphere of H_2 (5 bar) for 17 h. The reaction mixture was filtered through celite, flushing through with CH_2Cl_2, and concentrated *in vacuo*. The crude reaction product was diluted in 8:2 AcOH/H_2O (1.0 mL). $NaIO_4$ (90 mg, 0.42 mmol, 2.0 eq.) was added at 0 °C. The reaction mixture was warmed to rt, then stirred overnight. The reaction mixture was cooled to 0 °C, then quenched with 10 M solution NaOH (~1.5 mL) until it was basic. The reaction mixture was transferred to a separating funnel and extracted with 9:1 $CHCl_3$/MeOH (5 x 10 mL). The combined organics were dried over Na_2SO_4, filtered, and concentrated *in vacuo*. Flash column chromatography eluting with 40% {50:8:1 CH_2Cl_2:EtOH:NH_4OH} in CH_2Cl_2 gave the *title product* **S-27** (40 mg, 0.14 mmol, 67% over 2 steps) as a colourless amorphous solid. **^{1}H NMR** (500 MHz, CD_3OD, OH not observed, spectrum complicated by deuterium exchange: only 1 of 2 protons at 2-C observed): δ 8.25 (1H, d, *J* 5.2, pyridine 2-H), 7.39 (1H, d, *J* 5.2, pyridine 3-H), 5.14 (1H, d, *J* 4.9, C*H*(OH)), 3.67-3.60 (1H, m, quinuclidine 7-H_A), 3.30-3.25 (1H, m, 2-H), 3.11-3.04 (1H, m, quinuclidine 6-H), 2.96-2.88 (3H, m, C$H_A$$H_B$ and CH_2), 2.84-2.73 (2H, m, C$H_A$$H_B$ and quinuclidine 7-H_B), 2.46-2.42 (1H, m, quinuclidine 4-H), 2.37 (1H, ddd, *J* 13.4, 8.0, 2.2, quinuclidine 5-H_A), 2.19-2.12 (1H, m, quinuclidine 8-H_A), 1.97-1.80 (6H, m, includes 5-H_B, 8-H_B and 2 × CH_2). **^{13}C NMR** (125 MHz, CD_3OD): complicated by deuterium exchange at 2-C, see Section 7.0 for the processed NMR. **HRMS** (ESI): $C_{17}H_{23}O_2N_2$ $[M+H]^+$; calculated: 287.1754, found: 287.1751. $[\alpha]^D_{20}$ = −79 (c. 0.1, MeOH).

(*1R*,*11S*,*13S*)-5-Bromo-13-[(*R*)-hydroxy(5,6,7,8-tetrahydroquinolin-4-yl)methyl]-6-methyl-1,7,9-triazatetracyclo[9.2.2.0^{2,10}.0^{3,8}]pentadeca-2(10),3(8),4,6-tetraen-1-ium trifluoroacetate S-3

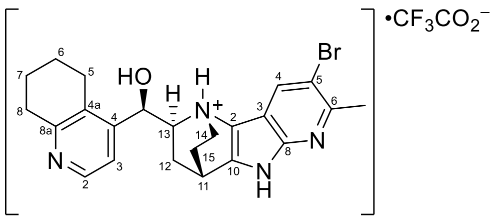

S-3

Prepared according to General Procedure B using ketone **S-27** (39 mg, 0.14 mmol), $MgSO_4$ (1.5 eq.) and 30 mol% $Pd(OAc)_2$ (72 h). Flash column chromatography eluting with 5-20% {50:80:1 CH_2Cl_2:EtOH:NH_4OH} in CH_2Cl_2 gave the *title compound* **S-3** (7 mg, 12 μmol, 9%) as a brown oil. **^{1}H NMR** (500 MHz, CD_3OD, OH not observed): δ 8.52 (1H, d, *J* 6.2, Py 2-H), 8.34 (1H, s, indole 4-H), 8.08 (1H, d, *J* 6.2, Py 3-H), 5.96 (1H, app. s, C*H*(OH)), 4.65 (1H, ddd, *J* 11.7, 9.2, 4.1, 14-H_A), 3.80-3.76 (1H, m, 11-H), 3.59 (1H, dd, *J* 10.3, 6.6, 13-H), 3.21-3.14 (1H, m, 14-H_B), 3.12-2.96 (3H, m, CH_AH_B and CH_2), 2.81-2.73 (1H, m, CH_AH_B), 2.68-2.62 (4H, m, includes 12-H_A, and at δ 2.65: 3H, s, CH_3), 2.47-2.39 (1H, m, 15-H_A), 1.99-1.80 (5H, m, 15-H_B and 2 × CH_2), 1.58-1.50 (1H, m, 12-H_B). **^{13}C NMR** (125 MHz, CD_3OD): δ 160.7 (Ar-C_q), 154.1 (Ar-C_q), 151.2 (Ar-C_q), 145.5 (Ar-C_q), 141.8 (Ar-C_q), 139.7 (Py 2-C), 136.1 (Ar-C_q), 129.8 (indole 4-C), 123.2 (Py 3-C), 115.4 (Ar-C_q), 114.9 (Ar-C_q), 111.6 (Ar-C_q), 67.8 (13-C), 67.5 (CH(OH)), 51.7 (14-C), 29.0 (CH_2), 28.5 (11-C), 26.8 (15-C), 25.7 (CH_2), 25.1 (CH_3), 24.7 (12-C), 22.1 (CH_2), 21.3 (CH_2). **HRMS** (ESI): $C_2H_{26}ON_4{}^{79}Br$ [M+H]$^+$; calculated: 453.1285, found: 453.1282; $C_2H_{26}ON_4{}^{81}Br$ [M+H]$^+$; calculated: 455.1264, found: 455.1262.

(*1S*,*4S*,*6S*)-6-[(1*R*)-1-(Quinolin-4-yl)ethyl]-1-azabicyclo[2.2.2]octan-3-one S-28

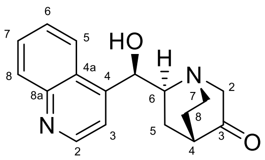

S-28

General Procedure A, Part II, was followed using compound **S-25** (2.0 g, 6.1 mmol). Flash column chromatography eluting with 5-20% {50:80:1 CH_2Cl_2:EtOH:$NH_4OH_{(sat. aq.)}$} in CH_2Cl_2 gave the *title product* **S-28** (791 mg, 2.80 mmol, 41%) as a yellow solid. **^{1}H NMR** (600 MHz, CD_3OD, OH not observed): δ 8.83 (1H, d, *J* 4.6, quinoline 2-H), 8.27 (1H, d, *J* 8.4 quinoline 8-H), 8.06 (1H, d, *J* 8.7, quinoline 5-H), 7.80-7.76 (1H, m, quinoline 6-H), 7.75 (1H, d, *J* 4.6, quinoline 3-H), 7.68-7.65 (1H, m, quinoline 7-H), 5.74 (1H, d, *J* 4.3, C*H*(OH)), 3.82-3.76 (1H, m, quinuclidine 7-H_A), 3.29-3.21 (3H, m, quinuclidine 6-H, quinuclidine 2-H_A and quinuclidine 2-H_B), 2.84-2.78 (1H, m, quinuclidine 7-H_B), 2.46-2.41 (2H, m, quinuclidine 4-H and quinuclidine 5-H_A), 2.25-2.18 (1H, m, quinuclidine 8-H_A), 1.95-1.88 (1H, m, quinuclidine 8-H_B), 1.80-1.74 (1H, m, quinoline 5-H_B). **^{13}C NMR** (151 MHz, CD_3OD): δ 220.3 (quinuclidine 3-C), 152.1 (Ar-C_q), 151.0 (quinoline 2-C), 148.8 (Ar-C_q), 130.7 (quinoline 6-C), 130.0 (quinoline 5-C), 128.2 (quinoline 7-C), 127.2 (Ar-C_q), 124.6 (quinolone 8-C), 119.9 (quinoline 3-C), 71.9 (CH(OH)), 65.5 (quinuclidine 2-C), 61.9 (quinuclidine 6-C), 43.4 (quinuclidine 7-C), 41.8 (quinuclidine 4-C), 26.6 (quinuclidine 5-C), 25.8 (quinuclidine 8-C). **HRMS** (ESI): calc. for $[M+H]^+$ $C_{17}H_{19}O_2N_2$: 283.1441, found: 283.1440. $[\alpha]_D^{20}$ = −128 (MeOH).

(*1R*,*11S*,*13S*)-5-Bromo-13-[(*R*)-hydroxy(quinolin-4-yl)methyl]-6-methyl-1,7,9-triazatetracyclo[9.2.2.02,10.03,8]pentadeca-2(10),3,5,7-tetraen-1-ium trifluoroacetate S-4

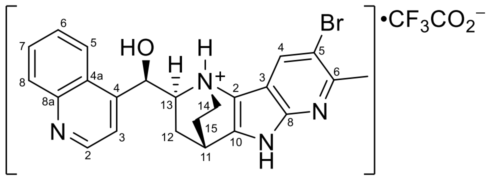

S-4

Prepared according to General Procedure B using ketone **S-28** (100 mg, 0.35 mmol, 1.0 eq.) and 20 mol% $Pd(OAc)_2$ (24 h). Flash column chromatography eluting with 5-20% {50:80:1 CH_2Cl_2:EtOH:NH_4OH} in CH_2Cl_2 followed by mass-directed preparative HPLC (MeCN in H_2O + 0.1% TFA; Rt = 11.4 min) gave the *title compound* **S-4** (18.3 mg, 40.7 µmol, 11%) as a yellow solid. **^{1}H NMR** (700 MHz, CD_3OD, NH^+, NH, and OH not observed): δ 9.04 (1H, d, *J* 5.2, quinoline 2-H), 8.49 (1H, d, app. *J* 8.5, quinoline 8-H), 8.34 (1H, s, indole 4-H), 8.16-8.13 (2H, m, quinoline 3-H and quinoline 5-H), 7.96 (1H, app. t, *J* 7.7, quinoline 6-H), 7.86 (1H, app. t, *J* 7.7, quinoline 7-H), 6.52 (1H, app. s, C*H*(OH)), 4.84-4.81 (1H, m, 14-H_A) 3.81-3.75 (2H, m, 11-H and 13-H), 3.25 (1H, td, *J* 11.3, 5.0, 14-H_B), 2.69 (1H, ddd, *J* 12.3, 6.4, 2.0, 12-H_A), 2.54-2.48 (1H, m, 15-H_A), 2.67 (3H, s, CH_3), 1.97-1.91 (1H, m, 15-H_B), 1.56-1.50 (1H, m, 12-H_B). **^{13}C NMR** (176 MHz, CD_3OD, *C*$F_3CO_2^-$ not observed): δ 162.5 (q, J_{CF} 35.6, CF_3*C*O_2^-), 154.2 (Ar-C_q), 151.1 (Ar-C_q), 148.7 (quinoline 2-C), 145.5 (Ar-C_q), 144.5 (Ar-C_q), 141.9 (Ar-C_q), 133.4 (quinolone 6-C), 130.3 (quinoline 7-C), 129.6 (indole 4-C), 126.9 (quinoline 3-C), 126.7 (Ar-C_q), 124.5 (quinoline 8-C), 120.4 (quinoline 5-C), 115.6 (Ar-C_q), 114.8 (Ar-C_q), 111.5 (Ar-C_q), 69.7 (13-C), 68.0 (CH(OH)), 52.1 (14-C), 28.6 (11-C), 26.9 (15-C), 25.2 (12-C), 25.1 (CH_3). **HRMS** (ESI): $C_{23}H_{22}ON_4{}^{79}Br$ $[M+H]^+$; calculated: 449.0972, found 449.09675; $C_{23}H_{22}ON_4{}^{81}Br$ $[M+H]^+$; calculated: 451.0951, found 451.0943. $[\alpha]_D^{20}$ = +123 (c. 0.1, MeOH).

6.3.2.5.4 Preparation of compound S-5

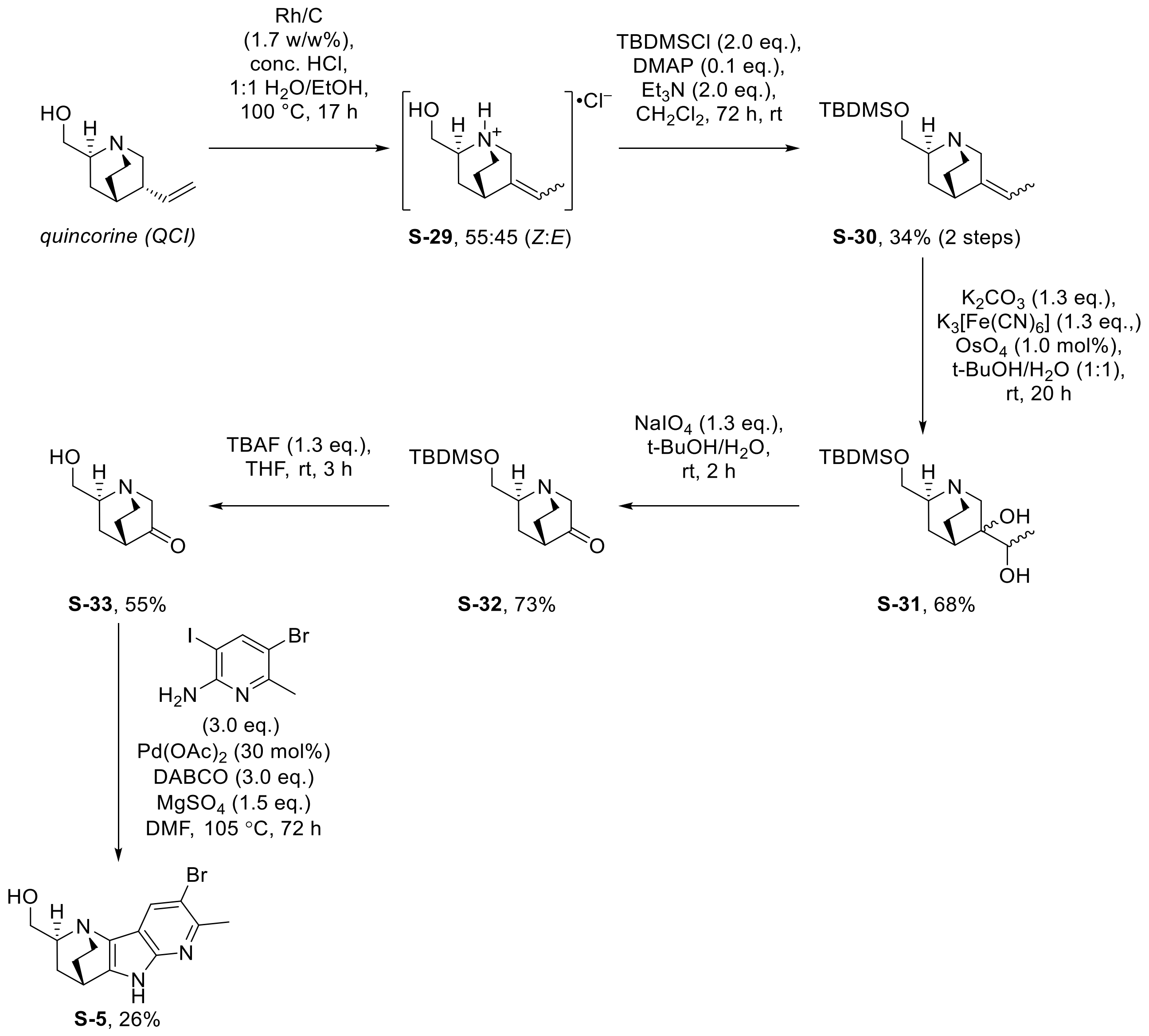

(*1S*,*2S*,*4S*)-5-Ethylidene-2-(hydroxymethyl)-1-azabicyclo[2.2.2]octan-1-ium chloride S-29

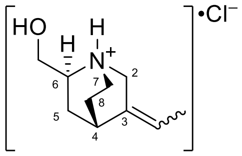

S-29

General procedure A, Part I, was followed using quincorine (1.52 g, 9.1 mmol). Flash column chromatography eluting with 1:4 ({50:80:1 DCM:EtOH:NH4OH}/DCM) gave the *title compound* **S-29** (2.1 g******) as yellow oil. **^{1}H NMR** (500 MHz, CD_3OD, 55:45 mixture of Z/E alkenes, NH^+ and OH not observed): δ 5.48 (0.55H, d, *J* 6.8, major C=C*H*CH_3), 5.42 (0.45H, d, *J* 6.8, minor C=C*H*CH_3), 4.09-3.91 (2H, m, 2-H_A and 2-H_B), 3.84-3.78 (1H, m C*H*$_A$$H_B$OH), 3.77-3.69 (1H, m CH_A*H*$_B$OH), 3.67-3.56 (2H, 6-H, 7-H_A), 3.29-3.21 (1H, m, 7-H_B), 3.09 (0.45H, s, minor 4-H), 2.63 (0.55H, s, major 4-H), 2.07-1.95 (2H, m, 5-H_A and 8-H_A), 1.95-1.86 (1H, m, 5-H_B), 1.71 (1.35H, d, *J* 6.8, minor CH_3), 1.61 (1.65H, d, *J* 6.8, major CH_3), 1.58-1.51 (1H, m, 8-H_B). **^{13}C NMR** (126 MHz, CD_3OD): δ 132.2 (minor 3-C), 131.4 (major 3-C), 120.8 (minor C=*C*HCH_3), 120.6 (major C=*C*HCH_3), 61.6 (minor 6-C), 61.4 (major 6-C), 61.6 (minor *C*H_2OH), 61.2 (major *C*H_2OH), 56.9 (minor 2-C), 55.1 (major 2-C), 42.8 (minor 7-C), 42.6 (major 7-C), 31.9 (minor 4-C), 28.4 (minor 8-C), 27.5 (major 8-C), 25.4 (minor 5-C), 25.1 (major 4-C), 24.5 (major 5-C), 12.9 (minor *C*H_3), 12.8 (major *C*H_3). **HRMS** (ESI): $C_{10}H_{18}ON$ $[M+H]^+$; calculated: 168.1383, found 168.1379.

****** Maximum theoretical yield = 1.2 g.

(*1S*,*2S*,*4S*)-2-{[(*tert*-Butyldimethylsilyl)oxy]methyl}-5-ethylidene-1-azabicyclo[2.2.2]octane S-30

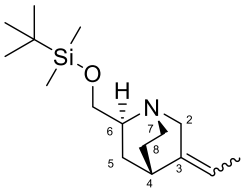

S-30

To a stirred solution of compound **S-29** (1.52 g, 9.1 mmol) in CH_2Cl_2 (30 mL, 0.3 M) was added Et_3N (3.8 mL, 27.2 mmol, 3.0 eq.). After the solution was stirred under Ar for 15 min, DMAP (111 mg, 0.9 mmol, 0.1 eq.) and TBDMSCl (4.11 g, 27.2 mmol, 3.0 eq.) were added at 0 °C. The reaction mixture was stirred for 48 h at rt until the reaction showed complete consumption of the starting material (monitored by LCMS). The reaction mixture was washed with saturated aqueous $NaHCO_3$ (50 mL) and the resulting solution was extracted with CH_2Cl_2 (4 x 50 mL). The combined organic layers were dried over $MgSO_4$, filtered and concentrated *in vacuo*. The crude product was purified by flash column chromatography eluting with 1:9 ({50:80:1 CH_2Cl_2:EtOH:NH_4OH}/ CH_2Cl_2) followed by removal of solvents under reduced pressure to give the *title compound* **S-30** (860 mg, 3.06 mmol, 34%) as yellow oil. **^{1}H NMR** (700 MHz, CD_3OD, 1:1 mixture of *Z*/*E* alkenes): δ 5.31-5.19 (1H, m, C=C*H*CH_3), 3.76-3.68 (2H, m, C*H*$_2$OTBDMS), 3.51-3.36 (2H, m, 2-H_A and 2-H_B), 3.22-3.14 (1H, m, 7-H_A), 2.88-2.82 (1H, m, 6-H), 2.81 (0.5H, s, 4-H^a), 2.72-2.63 (1H, m, 7-H_B), 2.32 (0.5H, s, 4-H^b), 1.78-1.66 (2H, m, 5-H_A and 8-H_A), 1.64-1.52 (5H, m, 5-H_B, 8-H_B and C=CHC*H*$_3$), 0.92 (9H, s, Si$(CH_3)_2$C(C*H*$_3$)$_3$), 0.10 (3H, app, d, *J* 1.3, Si(CH^a_3)$_2$C$(CH_3)_3$), 0.08 (3H, app. d, *J* 1.3, Si(CH^b_3)$_2$C$(CH_3)_3$). **^{13}C NMR** (176 MHz, CD_3OD): δ 141.6 (3-C^a), 140.5 (3-C^b), 115.7 (C^aHCH_3), 115.4 (C^bHCH_3), 66.5 ($C^a$$H_2$OTBDMS), 66.4 ($C^b$$H_2$OTBDMS), 59.5 (6-$C^a$), 59.2 (6-$C^b$), 58.9 (2-$C^a$), 56.6 (2-$C^b$), 43.7 (7-$C^a$), 43.6 (7-$C^b$), 34.4 (4-$C^a$), 32.0 (8-$C^a$), 31.1 (8-$C^b$), 28.7 (5-$C^a$), 27.5 (5-$C^b$), 26.8 (4-$C^b$), 26.4 (Si$(CH_3)_2$C($C^a$$H_3$)$_3$), 26.4 (Si$(CH_3)_2$C($C^b$$H_3$)$_3$), 19.2 (Si$(CH_3)_2$$C^a$$(CH_3)_3$), 19.2 (Si$(CH_3)_2$$C^b$$(CH_3)_3$), 12.8 (C=CH$C^a$$H_3$), 12.4 (C=CH$C^b$$H_3$), −5.3 (Si(*C*$H_3$)$_2C(CH_3)_3$). **HRMS** (ESI): $C_{16}H_{32}ONSi$ $[M+H]^+$; calculated: 282.2248 found 282.2247.

(*1S*,*4S*,*6S*)-6-{[(*tert*-Butyldimethylsilyl)oxy]methyl}-3-(1-hydroxyethyl)-1-azabicyclo[2.2.2]octan-3-ol S-31

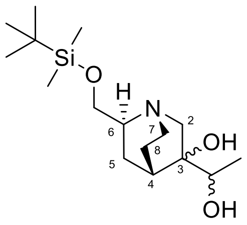

S-31

Compound **S-30** (860 mg, 3.05 mmol) was added to a two phase system of K_2CO_3 (1.28 g, 9.15 mmol, 3.0 eq.). and $K_3Fe(CN)_6$ (3.01 g, 9.15 mmol, 3.0 eq.) in 1:1 *t*BuOH/H_2O (0.3 M, 10.2 mL). The reaction mixture was stirred for 45 min, then 4% OsO_4 in H_2O (194 µL, 30.6 µmol, 1.0 mol%) was added at rt. After 20 h the reaction mixture was quenched with a saturated aqueous solution $Na_2S_2O_3$ (2 mL) and washed with saturated aqueous $NaHCO_3$ (50 mL) and the resulting solution was extracted with CH_2Cl_2 (4 x 50 mL). The combined organic layers were dried over $MgSO_4$, filtered and concentrated *in vacuo*. The obtained crude was purified by flash column chromatography eluting with 1:9 ({50:80:1 CH_2Cl_2:EtOH:NH_4OH}/CH_2Cl_2) followed by removal of solvents under reduced pressure gave the *title compound* **S-31** (655 mg, 2.08 mmol, 68 %) as white solid. **^{1}H NMR** (700 MHz, CD_3OD, **characteristic peaks given**, see Section 7.0 for the processed NMR): δ 3.92-3.78 (1H, m, C*H*(OH)CH_3), 3.75-3.67 (2H, m), 3.19-2.98 (2H, m), 2.81-2.42 (3H, m), 2.23-1.98 (1H, m), 1.88-1.47 (2H, m), 1.41-1.18 (2H, m), 1.17-1.10 (3H, m, CH(OH)C*H*$_3$), 0.93 (9H, s, Si$(CH_3)_2$C(C*H*$_3$)$_3$), 0.12-0.04 (6H, m, Si(C*H*$_3$)$_2$C$(CH_3)_3$). **^{13}C NMR** (176 MHz, CD_3OD): δ 74.5, 74.4, 74.1, 74.0, 73.8, 72.9, 72.6, 70.8, 70.0, 66.6, 66.5, 66.4, 65.1, 64.5, 62.7, 62.4, 58.3, 58.0, 57.8, 57.7, 42.7, 42.7, 42.6, 42.4, 31.3, 31.0, 30.1, 29.8, 27.0, 26.9, 26.4, 26.4, 26.3, 26.2, 23.6, 23.5, 22.5, 22.4, 19.2, 17.6, 16.2, 16.0, −5.3. **HRMS** (ESI): $C_{16}H_{34}O_3NSi$ $[M+H]^+$; calculated: 316.2302, found: 316.2301.

(*1S*,*4S*,*6S*)-6-{[(*tert*-Butyldimethylsilyl)oxy]methyl}-1-azabicyclo[2.2.2]octan-3-one S-32

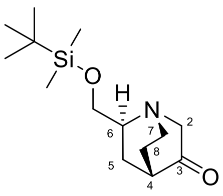

S-32

Compound **S-31** (655 mg, 2.08 mmol) was dissolved in *t*BuOH (7 mL, 0.3 M). A saturated solution of $NaIO_4$ (578 mg, 2.70 mmol, 1.3 eq.) in H_2O (6.35 mL) was added. The reaction mixture was stirred at rt for 2 h, treated with aqueous $NaHCO_3$ (50 mL) and extracted with CH_2Cl_2 (4 x 50 mL). The combined organic layers were dried over $MgSO_4$, filtered and concentrated *in vacuo*. Flash column chromatography eluting with 1:9 ({50:80:1 CH_2Cl_2:EtOH:NH_4OH}/CH_2Cl_2) gave the *title product* **S-32** (412 mg, 1.53 mmol, 73%) as white solid. **^{1}H NMR** (600 MHz, CD_3OD): δ 3.82 (1H, s, C*H*$_A$H$_B$OTBDMS), 3.81 (1H, s, CH$_A$H$_B$OTBDMS), 3.38-3.32 (1H, m, 7-H$_A$), 3.08-2.95 (1H, m, 6-H), 2.84-2.73 (1H, m, 7-H$_B$), 2.44-2.36 (1H, m, 4-H), 2.11-1.96 (2H, m, 5-H$_A$ and 8-H$_A$), 1.95-1.80 (2H, m, 5-H$_B$ and 8-H$_B$), 0.94 (9H, s, Si(CH$_3$)$_2$C(C*H*$_3$)$_3$), 0.12 (6H, d, *J* 3.5, Si(C*H*$_3$)$_2$C(CH$_3$)$_3$). **^{13}C NMR** (151 MHz, CD_3OD): δ 220.2 (3-C), 66.1 (*C*H$_2$OTBDMS), 58.8 (6-C), 42.5 (7-C), 41.6 (4-C), 29.1 (8-C), 26.4 (Si(CH$_3$)$_2$C(*C*H$_3$)$_3$), 25.9 (5-C), 19.2 (Si(CH$_3$)$_2$*C*(CH$_3$)$_3$), −5.4 (Si(*C*H$_3$)$_2$C(*C*H$_3$)$_3$). **HRMS** (ESI): $C_{14}H_{28}O_2NSi$ [M+H]$^+$; calculated: 270.1884, found 270.1884. $[\boldsymbol{\alpha}]_{\mathbf{D}}^{\mathbf{20}}$ = −21 (c. 0.1, MeOH).

(*1S*,*4S*,*6S*)-6-(Hydroxymethyl)-1-azabicyclo[2.2.2]octan-3-one S-33

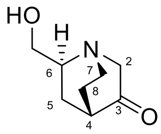

S-33

TBAF (1.0 M in THF, 2.9 mL, 1.3 eq.) was added to compound **S-32** (600 mg, 2.23 mmol, 1.0 eq.) in THF (4.5 mL) at 0 °C. The resulting mixture was stirred at rt for 20 h, then a 1:1 sat aq. brine / sat. aq. $NaHCO_3$ mixture was added (10 mL). The mixture was extracted with $CHCl_3$ (1 × 20 mL), $CHCl_3$:MeOH (9:1, 3 × 20 mL), and $CHCl_3$:MeOH (8:2, 3 × 20 mL). The combined organics were dried over $MgSO_4$, filtrated and concentrated *in vacuo*. Flash column chromatography eluting with 5% to 10% {50:80:1 CH_2Cl_2:EtOH:NH_4OH} in CH_2Cl_2, gave the *title compound* **S-33** as white solid (191 mg, 1.23 mmol, 55%). **^{1}H NMR** (600 MHz, CD_3OD, OH, 2-H not observed): δ 3.71 (dd, *J* 11.5, 8.2 Hz, 1H, C*H*$_A$H$_B$OH), 3.61 (dd, *J* 11.5, 6.0 Hz, 1H, CH$_A$*H*$_B$OH), 3.23 (ddd, *J* 14.3, 10.2, 5.5 Hz, 1H, quinuclidine 7-H$_A$), 3.07-2.98 (m, 1H, quinuclidine 6-H), 2.84-2.74 (m, 1H, quinuclidine 7-H$_B$), 2.39-2.35 (m, 1H, quinuclidine 4-H) , 2.11-2.03 (m, 1H, quinuclidine 5-H$_A$), 2.00-1.94 (m, 1H, quinuclidine 8-H$_A$), 1.94-1.88 (m, 1H, quinuclidine 8-H$_B$), 1.62 (ddd, *J* 13.5, 7.5, 2.3 Hz, 1H, quinuclidine 5-H$_B$). **^{13}C NMR** (151 MHz, CD_3OD): δ 220.5 (quinuclidine 3-C), 98.2 (quinuclidine 2-C), 64.0 (*C*H_2OH), 58.8 (quinuclidine 6-C), 41.5 (quinuclidine 4-C), 41.2 (quinuclidine 7-C), 29.7 (quinuclidine 5-C), 26.0 (quinuclidine 8-C). **HRMS** (ESI): $C_8H_{14}O_2N$ $[M+H]^+$; calculated: 156.1019, found: 156.1016. $[\alpha]_D^{20}$ = −27 (c. 0.1, MeOH).

[(*11S*,*13S*)-5-Bromo-6-methyl-1,7,9-triazatetracyclo[9.2.2.02,10.03,8]pentadeca-2(10),3,5,7-tetraen-13-yl]methanol S-5

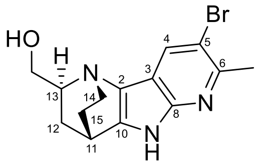

S-5

Prepared according to General Procedure B using ketone **S-33** (100 mg, 0.35 mmol, 1.0 eq.) and 20 mol% $Pd(OAc)_2$. Flash column chromatography eluting with 5-15% {50:80:1 CH_2Cl_2:EtOH:NH_4OH} in CH_2Cl_2 gave the *title compound* **S-5** (53 mg, 0.17 mmol, 26%) as a yellow solid. **^{1}H NMR** (600 MHz, CD_3OD, NH and OH not observed): δ 8.16 (1H, s, 4-H), 3.85 (1H, dd, *J* 11.4, 8.1, CH_AH_BOH), 3.77 (1H, dd, *J* 11.4, 6.1 Hz, CH_AH_BOH), 3.50-3.44 (1H, m, 14-H_A), 3.42-3.39 (1H, m, 11-H), 2.89-2.82 (1H, m, 13-H), 2.65 (3H, s, CH_3), 2.53-2.44 (1H, m, 14-H_B), 1.96-1.90 (1H, m, 15-H_A), 1.84-1.77 (1H, m, 12-H_A), 1.62-1.55 (1H, m, 15-H_B), 1.55-1.50 (1H, m, 12-H_B). **^{13}C NMR** (151 MHz, CD_3OD): δ 148.0 (Ar-C_q), 145.8 (Ar-C_q), 145.4 (Ar-C_q), 129.2 (4-C), 125.1 (Ar-C_q), 115.6 (Ar-C_q), 113.6 (Ar-C_q), 64.6 (CH_2OH), 64.4 (13-H), 45.7 (14-C), 33.5 (12-C), 30.3 (15-C), 28.9 (11-C), 24.7 (CH_3). **HRMS** (ESI): $C_{14}H_{17}ON_3{}^{79}Br$ [M+H]+; calculated: 322.0549, found: 322.0554; $C_{14}H_{17}ON_3{}^{81}Br$ $[M+H]^+$; calculated: 324.0529, found: 324.0527. $[\alpha]_D^{20}$ = −36 (c. 0.1, MeOH).

6.3.2.5.5 Preparation of compound S-6

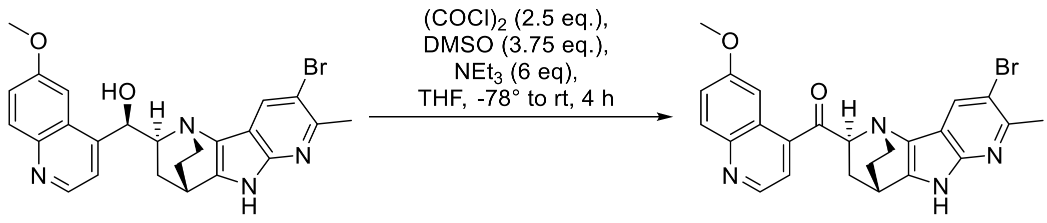

10w-j **S-34**, 19%

(13*S*)-5-Bromo-13-(6-methoxyquinoline-4-carbonyl)-6-methyl-1,9-diazatetracyclo [9.2.2.0^{2,10}.0^{3,8}]pentadeca-2(10),3,5,7-tetraene S-6

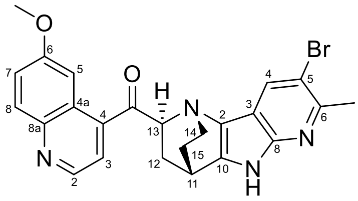

S-34

DMSO (22.2 µL, 313 µmol, 3 eq.) in THF (800 µL) was cooled to −78°C. Oxalylchloride (17.9 µL 0.21 mmol, 2.5 eq.) was added slowly and the mixture was stirred for 0.5 h at −78°C. Compound **10w-j** (Azaquindole-1, 40 mg, 83 µmol) was dissolved in THF (0.1 M, 800 µL) and added dropwise. The mixture was stirred for 0.5 h at −78°C, then Et_3N (69.8 µL, 0.50 mmol, 6.0 eq.) was added dropwise. The mixture was stirred at −78°C for 0.5 h, at 0°C for 0.5 h and at rt for 2 h. Purification by mass-directed preparative HPLC (MeCN in H_2O + 0.1% TFA; Rt = 14 min) followed by column chromatography eluting with 10-50% {50:80:1 CH_2Cl_2:EtOH:NH_4OH} in CH_2Cl_2 gave the *title compound* **S-34** as an orange solid (7.4 mg, 15.5 µmol, 19%). **^{1}H NMR** (600 MHz, Cl_3CD): δ 8.95 (1H, d, *J* 4.4, quinoline 2-H), 8.87 (1H, s, NH), 8.04 (1H, d, *J* 9.2, quinoline 8-H), 7.69 (1H, d, *J* 4.4, quinoline 3-H), 7.31 (1H, dd, *J* 9.2, 2.8, quinolone 7-H), 6.55 (1H, s, indole 4-H), 6.30 (1H, d, *J* 2.9, quinoline 5-H), 4.66 (1H, dd, *J* 8.4, 4.2, 13-H), 3.60-3.57 (1H, m, 11-H), 3.44-3.38 (1H, m, 14-H_A), 3.24 (3H, s, OCH_3), 2.73-2.67 (1H, m, 14-H_B), 2.66-2.62 (1H, m, 12-H_A), 2.61 (3H, s, CH_3), 2.22-2.17 (1H, m, 12-H_B), 2.09-2.03 (1H, m, 15-H_A), 1.66-1.59 (1H, m, 15-H_B). **^{13}C NMR** (126 MHz, Cl_3CD): δ 202.2 (*C*=O), 158.5 (quinoline 6-C), 148.0 (Ar-C_q), 146.8 (quinoline 2-C), 145.6 (Ar-C_q), 144.5 (Ar-C_q), 143.6 (Ar-C_q), 142.0 (Ar-C_q), 131.3 (quinolone 8-C), 128.4 (indole 4-C), 125.4 (Ar-C_q), 123.1 (quinoline 7-C), 120.8 (Ar-C_q), 119.5 (quinoline 3-C), 115.2 (Ar-C_q), 113.5 (Ar-C_q), 102.2 (quinoline 5-C), 67.6 (13-C), 54.8 (OCH_3), 51.7 (14-C), 31.0 (12-C), 29.6 (15-C), 28.2 (11-C), 24.9 (CH_3). **HRMS** (ESI): $C_{24}H_{22}O_2N_4{}^{79}Br$ $[M+H]^+$; calculated: 477.0921; found: 477.0907; $C_{24}H_{22}O_2N_4{}^{81}Br$ $[M+H]^+$; calculated: 479.0900, found: 479.0887. $[\alpha]_D^{20}$ +48 (c 0.1, MeCN).

7.0 Processed NMR Spectra

Compounds are listed in order of appearance within the Supporting Information.

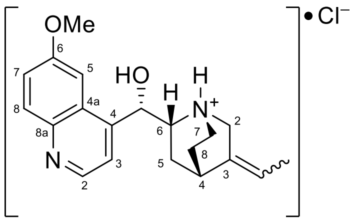
OMe
HO
H
H
N+
N
•Cl−

7

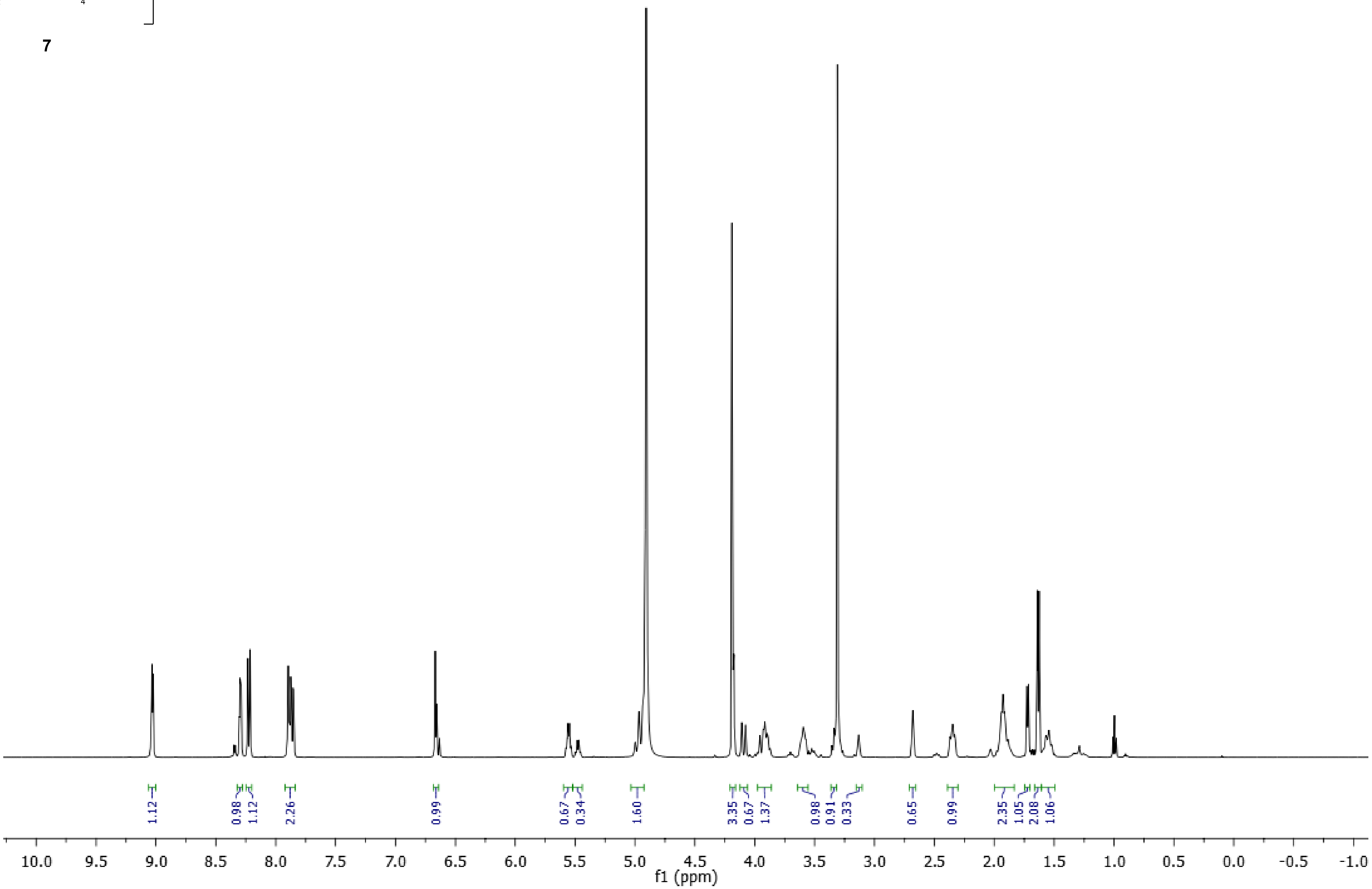
1.12
0.98
1.12
2.26
0.99
0.67
0.34
1.60
3.35
0.67
1.37
0.98
0.91
0.33
0.65
0.99
2.35
1.05
2.08
1.06
10.0
9.5
9.0
8.5
8.0
7.5
7.0
6.5
6.0
5.5
5.0
4.5
4.0
3.5
3.0
2.5
2.0
1.5
1.0
0.5
0.0
-0.5
-1.0
f1 (ppm)

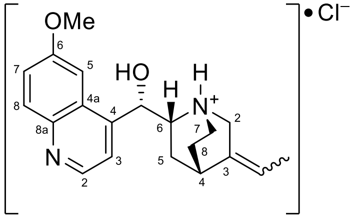
OMe
HO
H
H
N+
Cl−

7

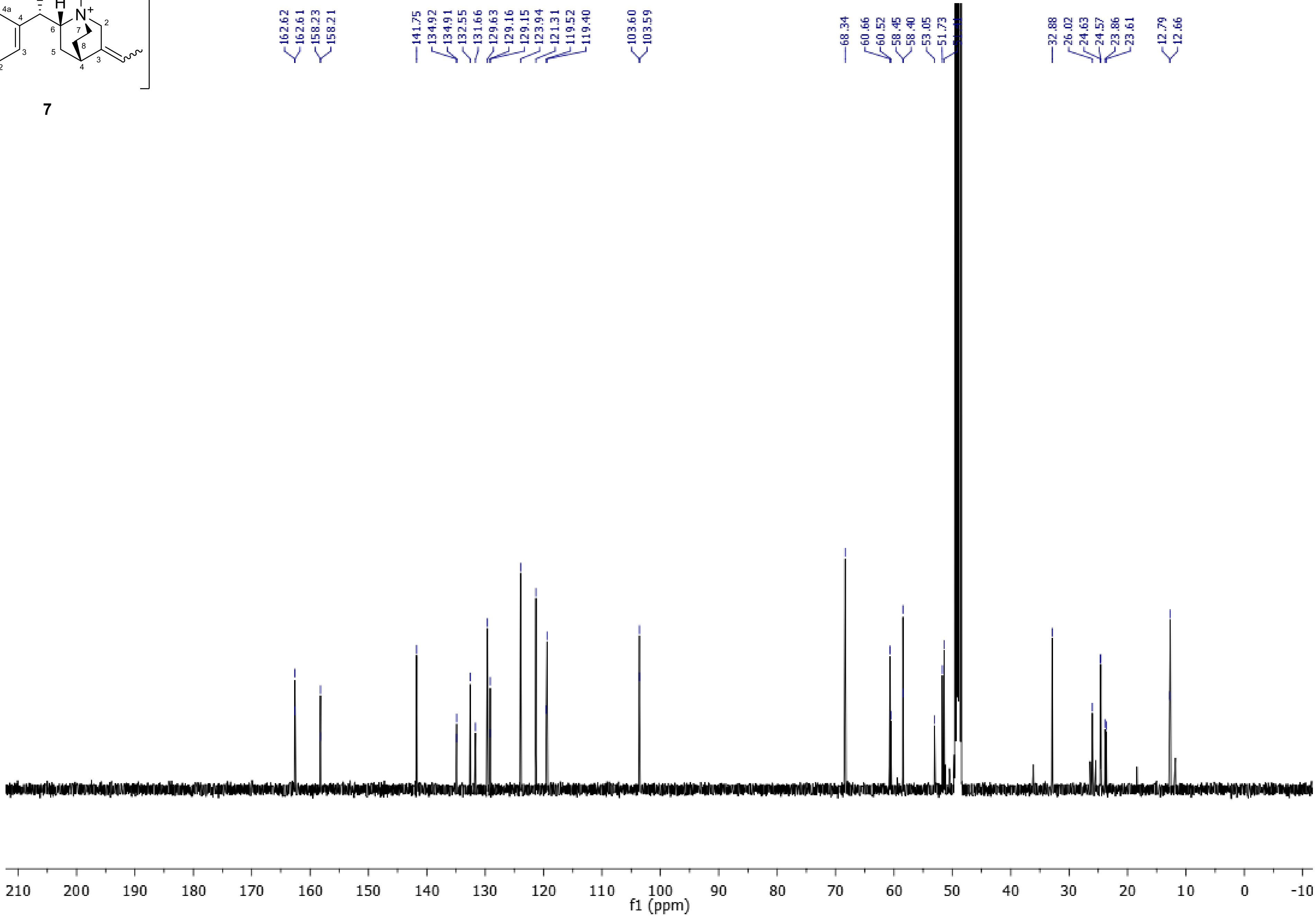
162.62
162.61
158.23
158.21
141.75
134.92
134.91
132.55
131.66
129.63
129.16
129.15
123.94
121.31
119.52
119.40
103.60
103.59
68.34
60.66
60.52
58.45
58.40
53.05
51.73
32.88
26.02
24.63
24.57
23.86
23.61
12.79
12.66
210 200 190 180 170 160 150 140 130 120 110 100 90 80 70 60 50 40 30 20 10 0 -10
f1 (ppm)

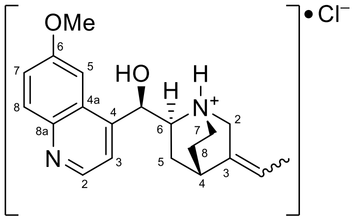
OMe
HO
H
H
N+
N
•Cl⁻

8

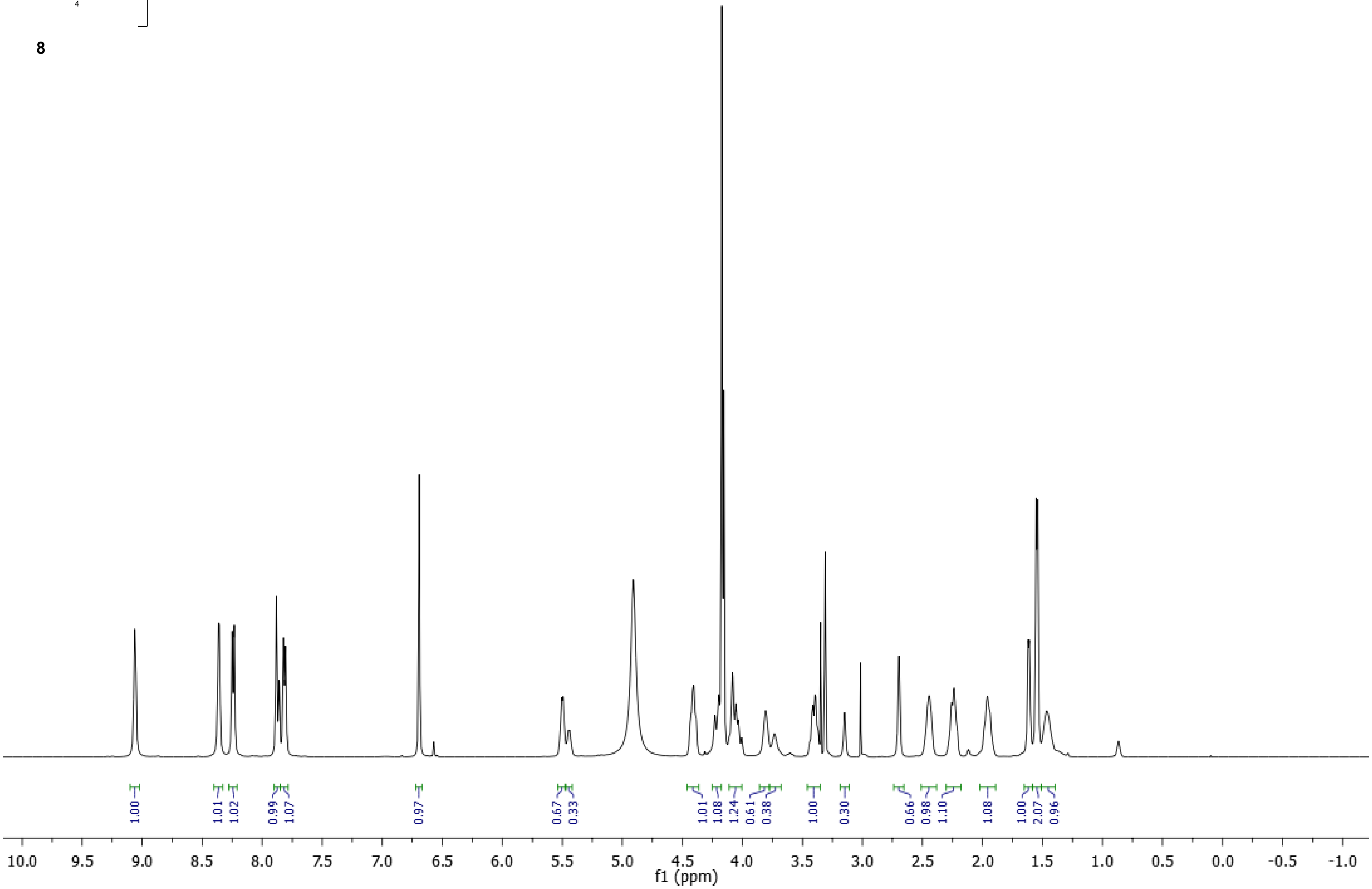
1.00
1.01
1.02
0.99
1.07
0.97
0.67
0.33
1.01
1.08
1.24
0.61
0.38
1.00
0.30
0.66
0.98
1.10
1.08
1.00
2.07
0.96
10.0 9.5 9.0 8.5 8.0 7.5 7.0 6.5 6.0 5.5 5.0 4.5 4.0 3.5 3.0 2.5 2.0 1.5 1.0 0.5 0.0 -0.5 -1.0
f1 (ppm)

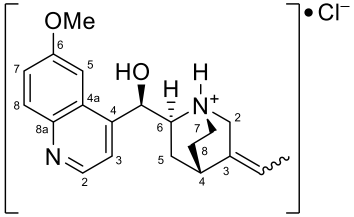
OMe
HO
H
N+
•Cl−
N

8

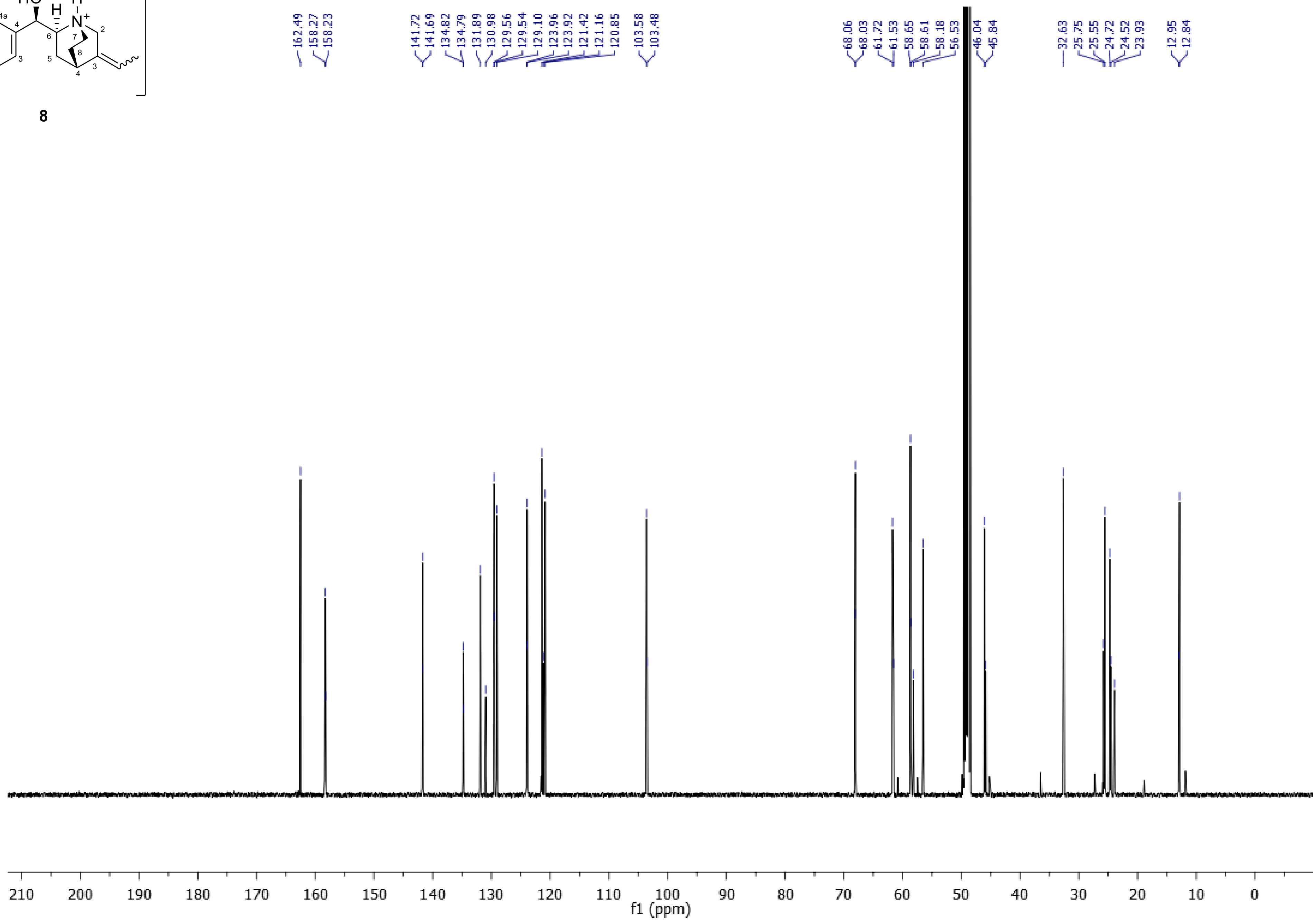
162.49
158.27
158.23
141.72
141.69
134.82
134.79
131.89
130.98
129.56
129.54
129.10
123.96
123.92
121.42
121.16
120.85
103.58
103.48
68.06
68.03
61.72
61.53
58.65
58.61
58.18
56.53
46.04
45.84
32.63
25.75
25.55
24.72
24.52
23.93
12.95
12.84
210 200 190 180 170 160 150 140 130 120 110 100 90 80 70 60 50 40 30 20 10 0
f1 (ppm)

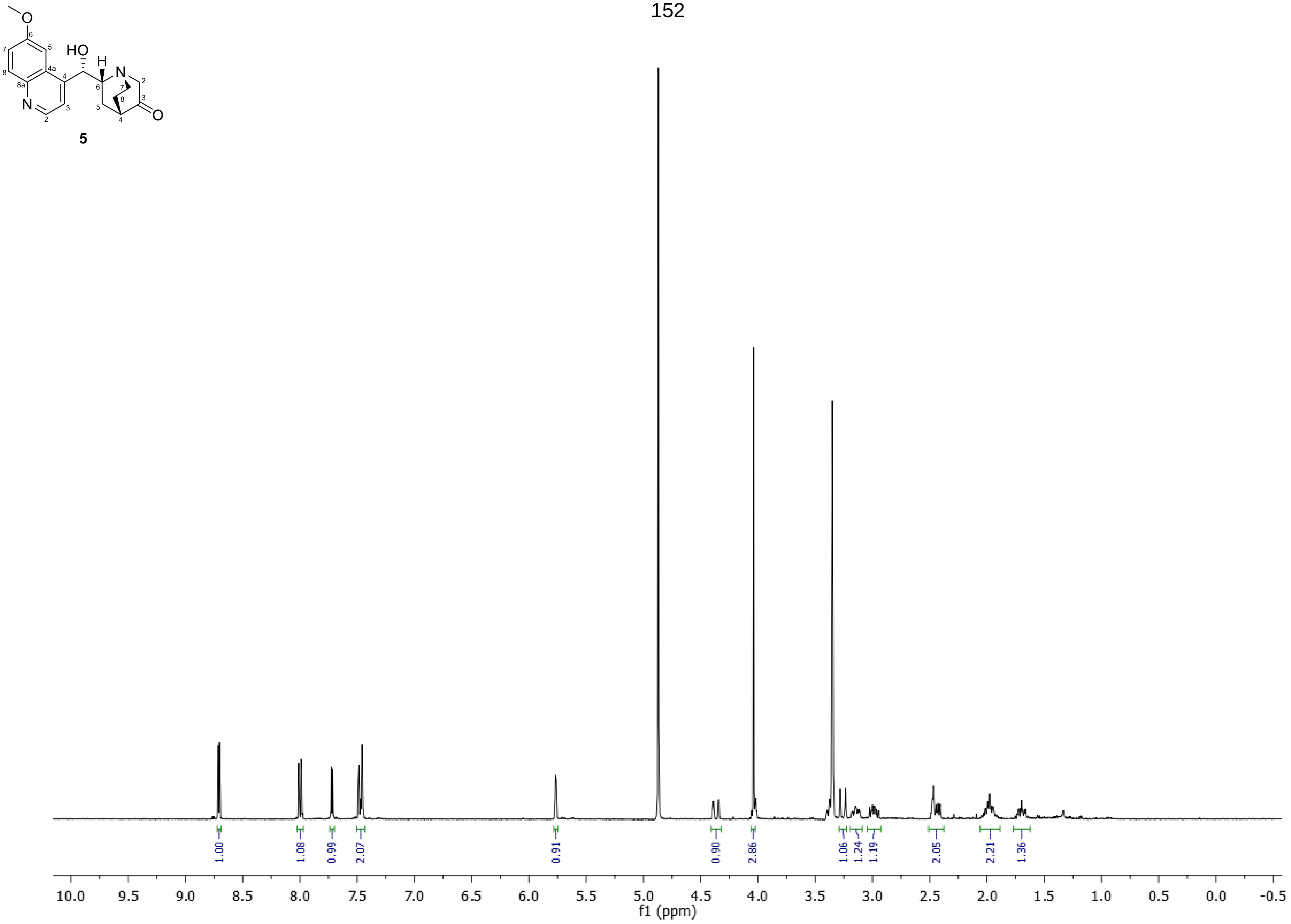

HO
H
N
O
5
1.00
1.08
0.99
2.07
0.91
0.90
2.86
1.06
1.24
1.19
2.05
2.21
1.36
10.0 9.5 9.0 8.5 8.0 7.5 7.0 6.5 6.0 5.5 5.0 4.5 4.0 3.5 3.0 2.5 2.0 1.5 1.0 0.5 0.0 -0.5
f1 (ppm)

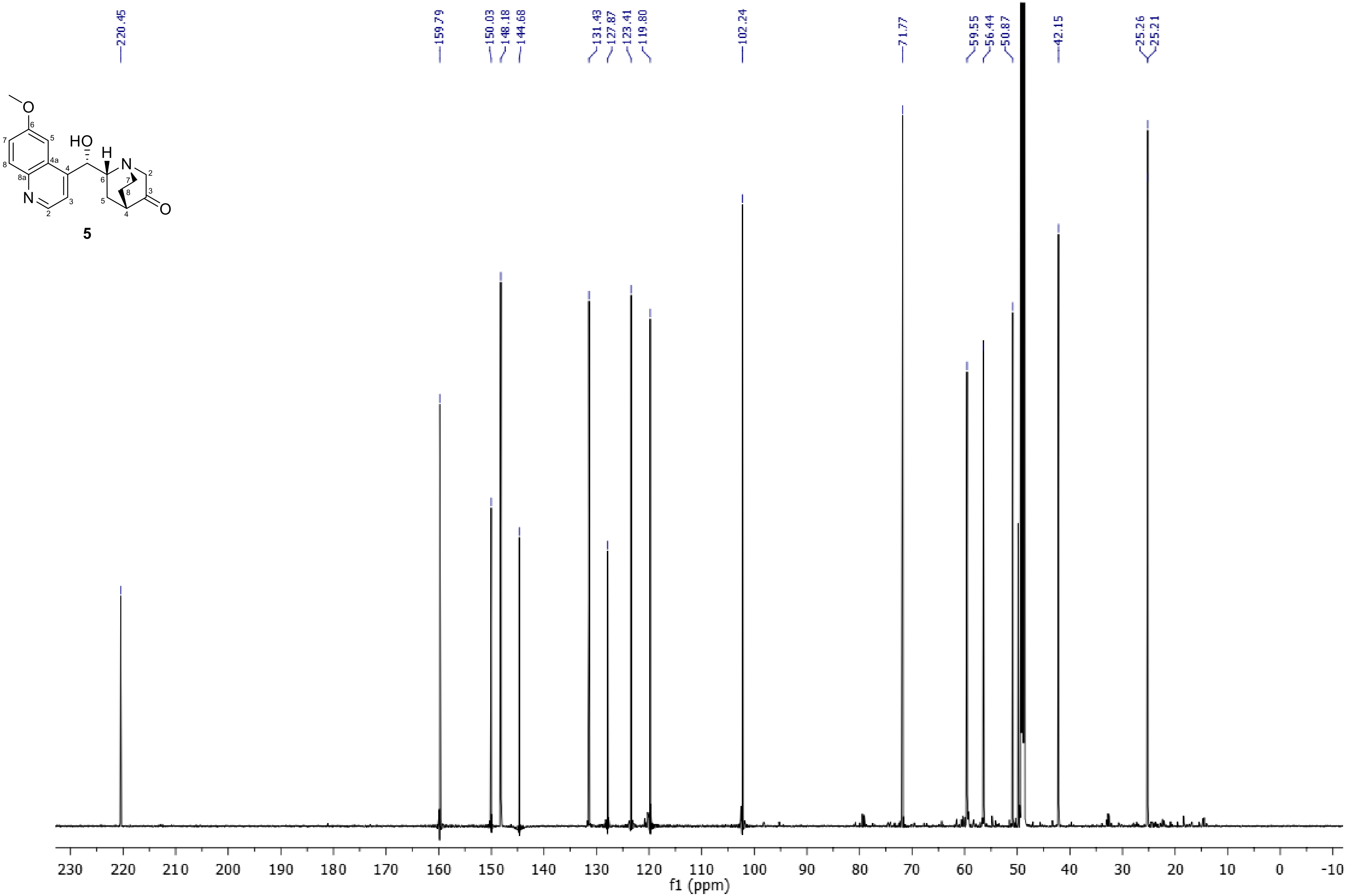
O
6
7
5
HO
H
8
4a
4
N
2
8a
6
7
3
N
3
8
O
5
2
3
4
5
220.45
159.79
150.03
148.18
144.68
131.43
127.87
123.41
119.80
102.24
71.77
59.55
56.44
50.87
42.15
25.26
25.21
230 220 210 200 190 180 170 160 150 140 130 120 110 100 90 80 70 60 50 40 30 20 10 0 -10
f1 (ppm)

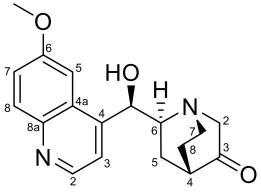

6

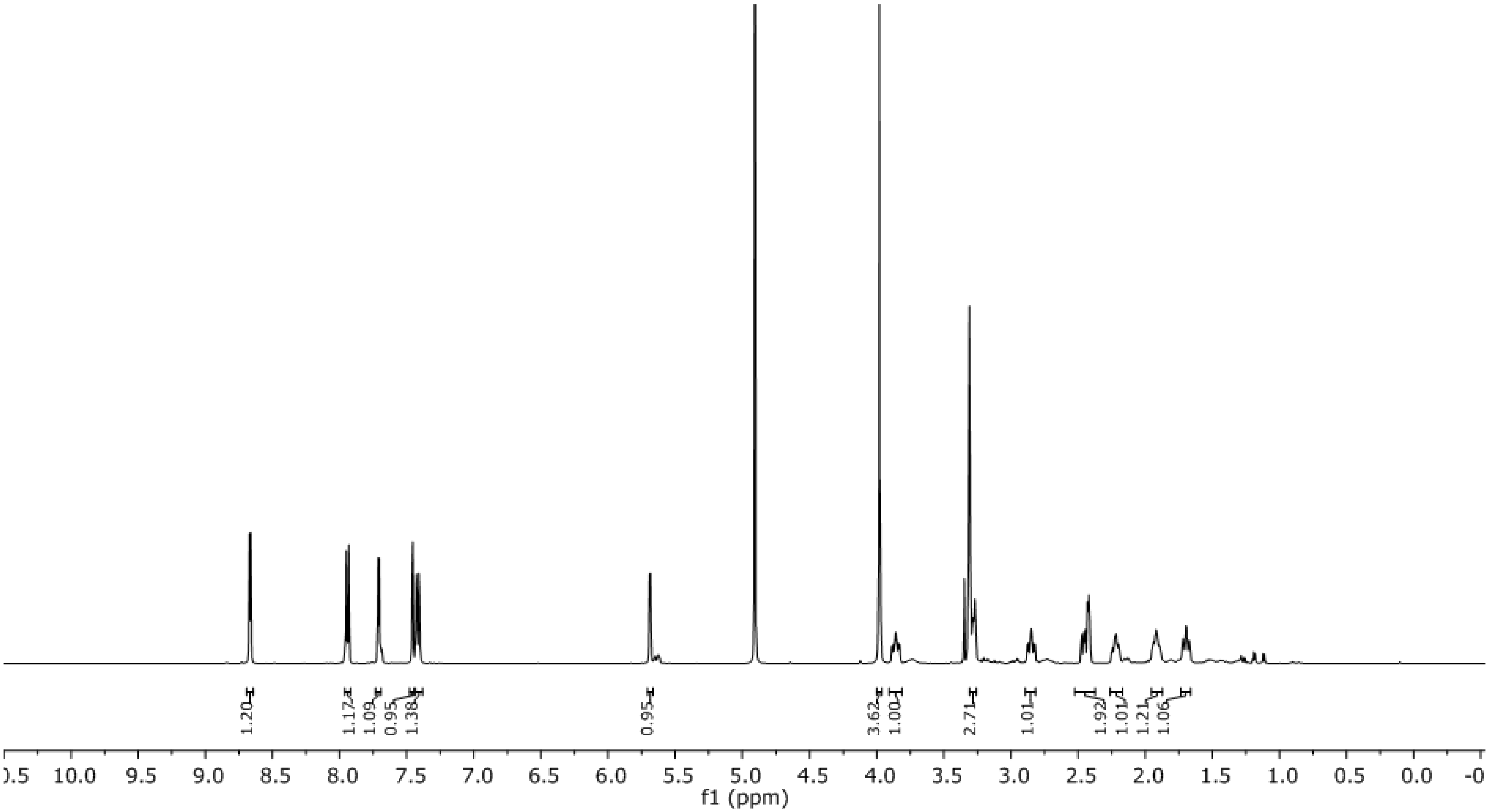

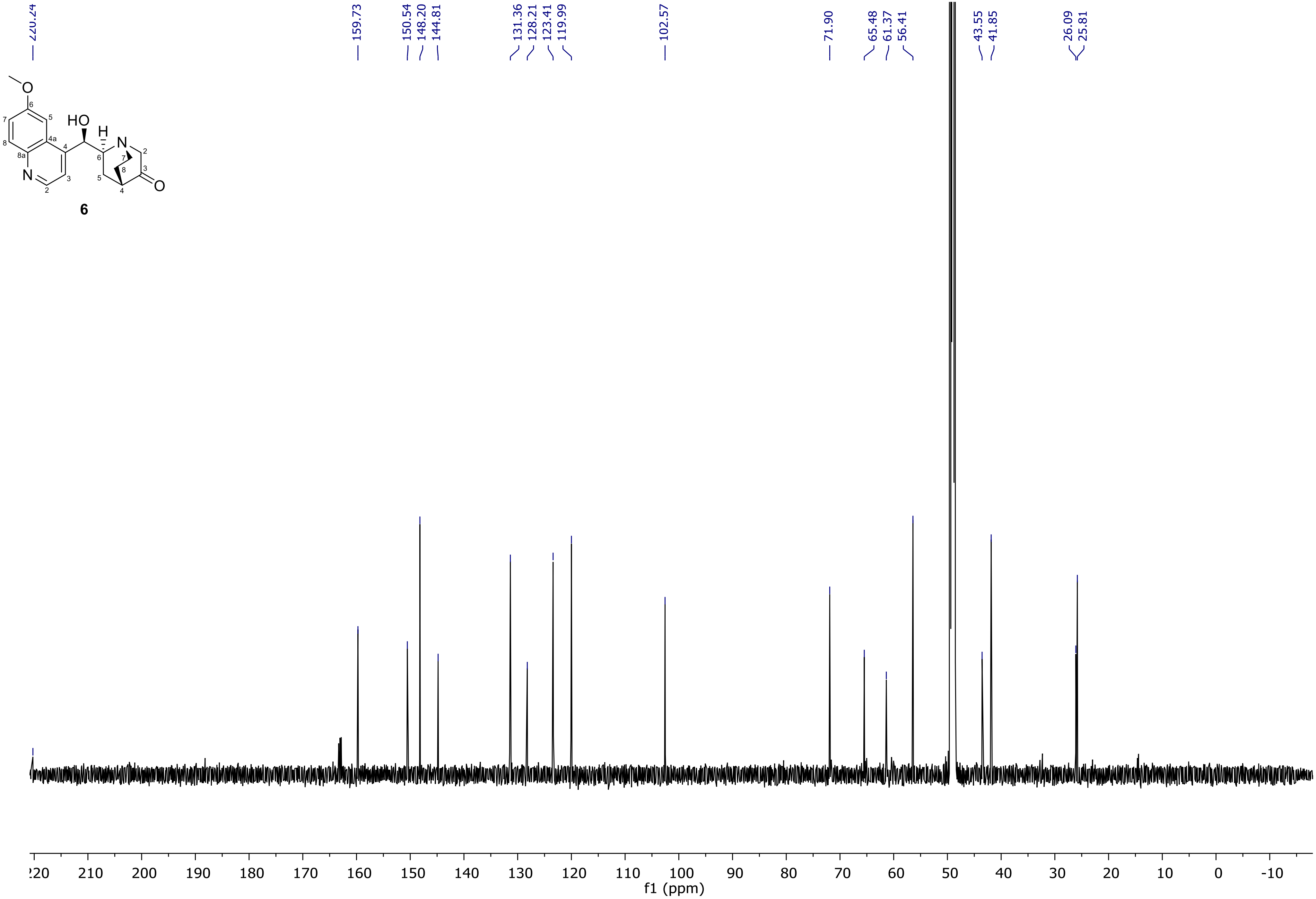
220.24
159.73
150.54
148.20
144.81
131.36
128.21
123.41
119.99
102.57
71.90
65.48
61.37
56.41
43.55
41.85
26.09
25.81
HO
H
N
O
6
f1 (ppm)

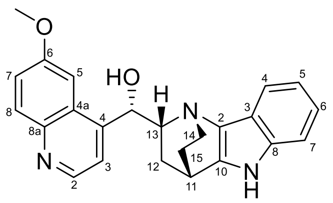
HO
H
N
N
H

9a

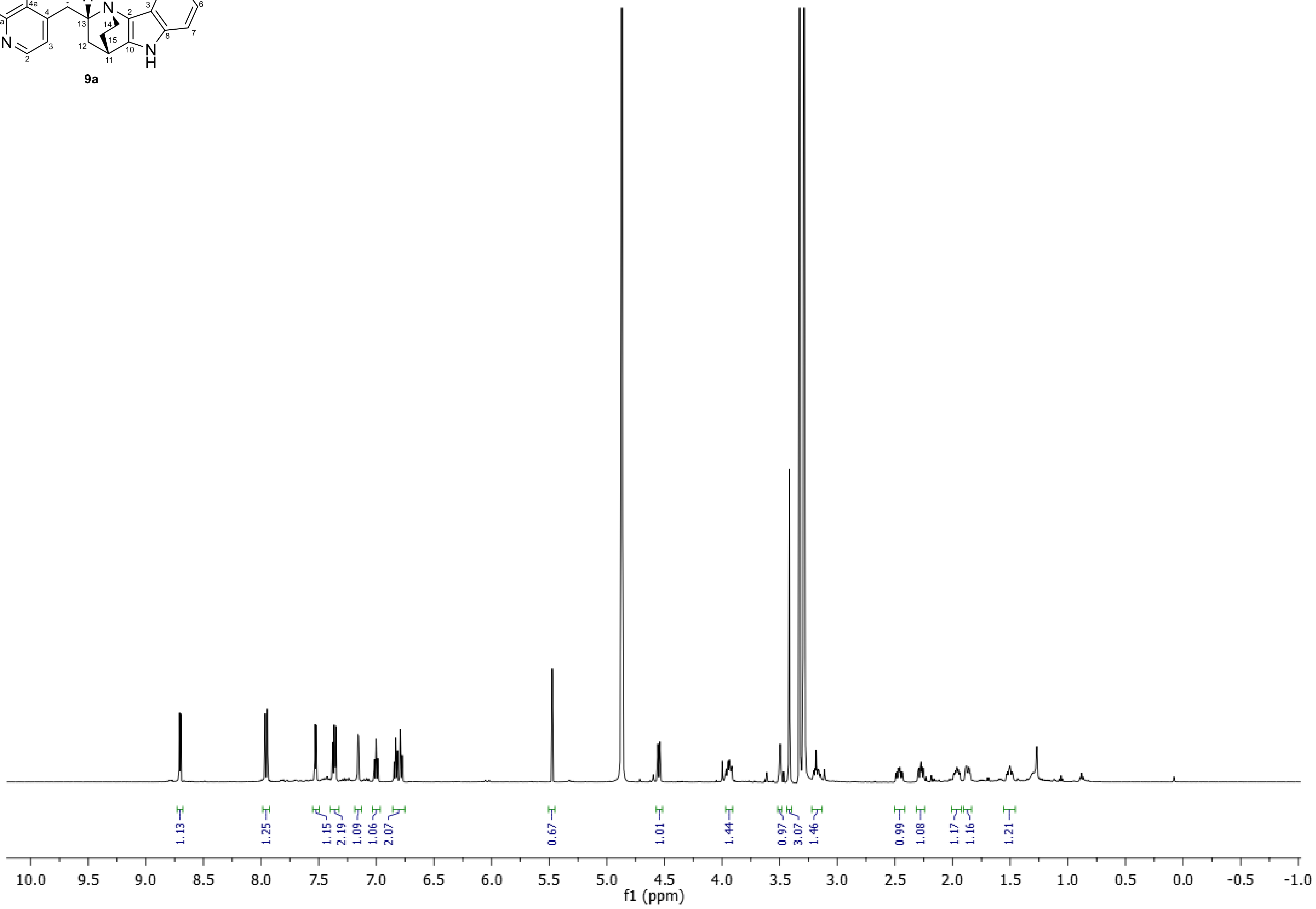
1.13
1.25
1.15
2.19
1.09
1.06
2.07
0.67
1.01
1.44
0.97
3.07
1.46
0.99
1.08
1.17
1.16
1.21
10.0 9.5 9.0 8.5 8.0 7.5 7.0 6.5 6.0 5.5 5.0 4.5 4.0 3.5 3.0 2.5 2.0 1.5 1.0 0.5 0.0 -0.5 -1.0
f1 (ppm)

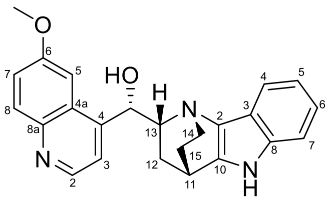

HO
H
N
NH

9a

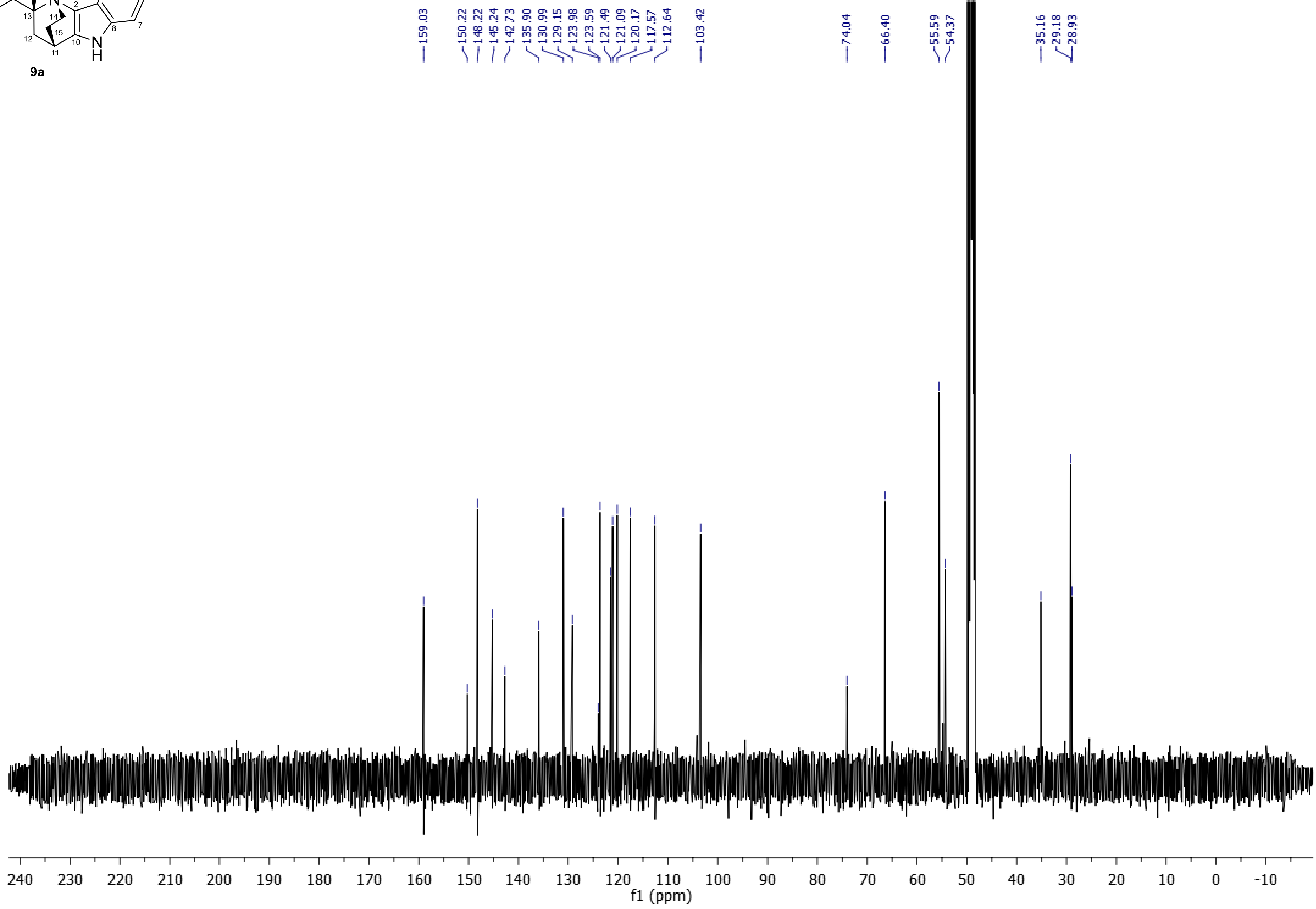

159.03
150.22
148.22
145.24
142.73
135.90
130.99
129.15
123.98
123.59
121.49
121.09
120.17
117.57
112.64
103.42
74.04
66.40
55.59
54.37
35.16
29.18
28.93
240 230 220 210 200 190 180 170 160 150 140 130 120 110 100 90 80 70 60 50 40 30 20 10 0 -10
f1 (ppm)

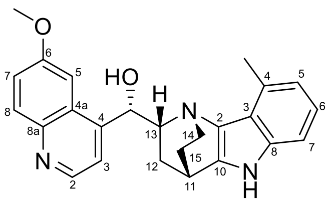
HO
H
N
NH
O

9b

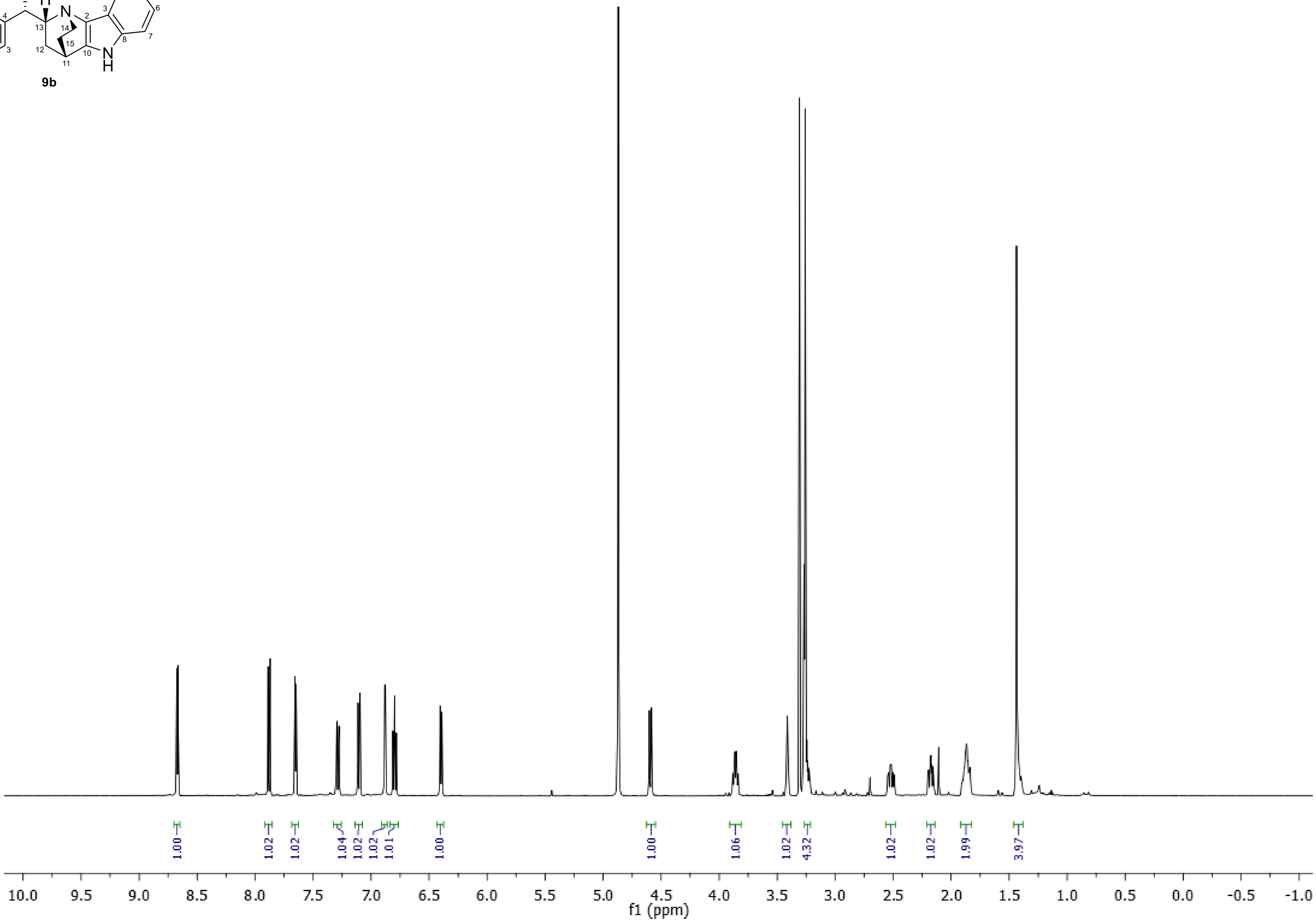
1.00
1.02
1.02
1.04
1.02
1.02
1.01
1.00
1.00
1.06
1.02
4.32
1.02
1.02
1.99
3.97
10.0 9.5 9.0 8.5 8.0 7.5 7.0 6.5 6.0 5.5 5.0 4.5 4.0 3.5 3.0 2.5 2.0 1.5 1.0 0.5 0.0 -0.5 -1.0
f1 (ppm)

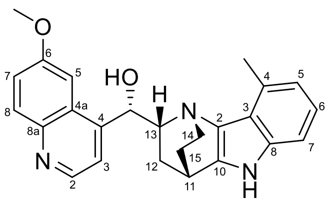
HO
H
N
NH
N
O

9b

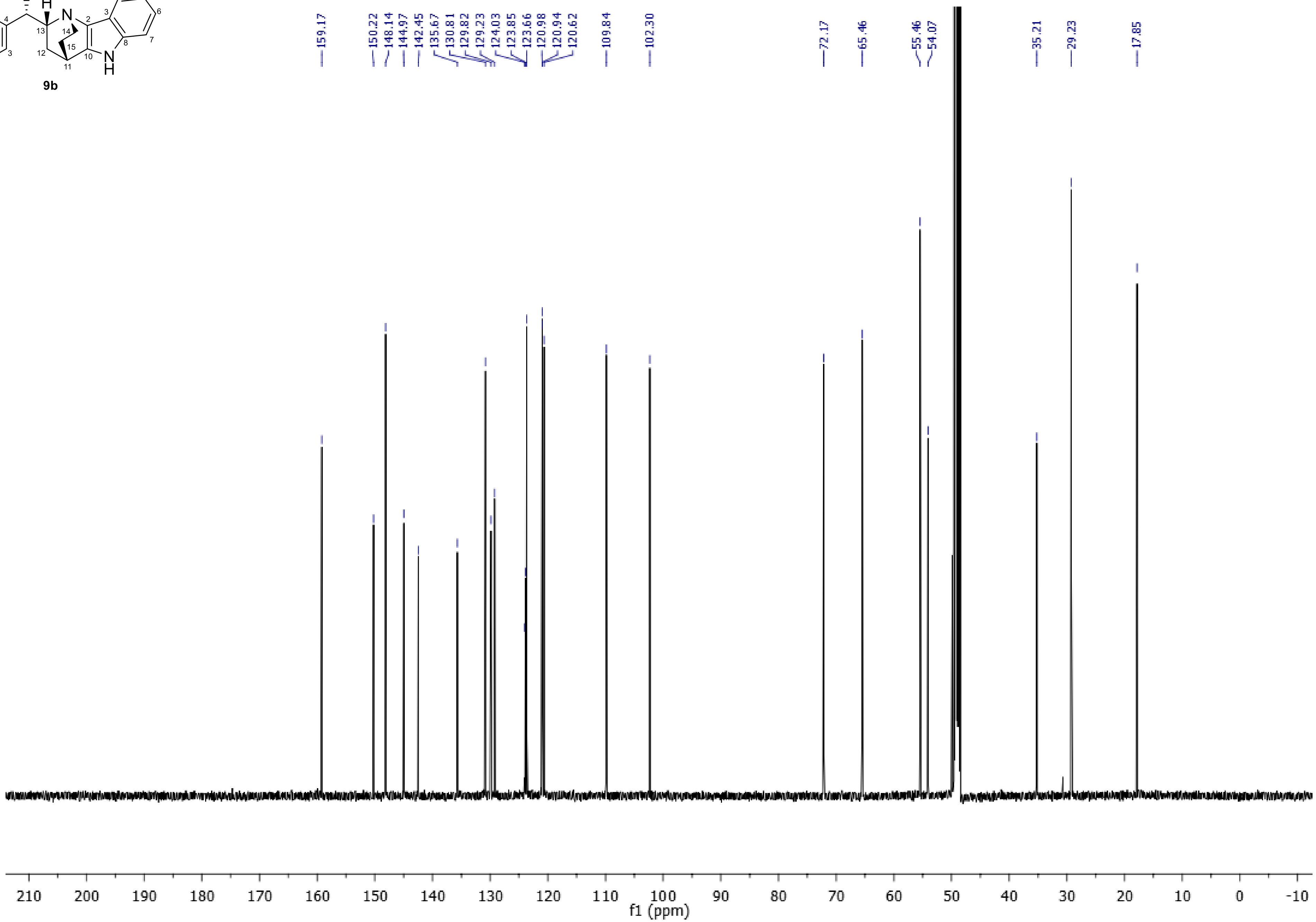
159.17
150.22
148.14
144.97
142.45
135.67
130.81
129.82
129.23
124.03
123.85
123.66
120.98
120.94
120.62
109.84
102.30
72.17
65.46
55.46
54.07
35.21
29.23
17.85
210 200 190 180 170 160 150 140 130 120 110 100 90 80 70 60 50 40 30 20 10 0 -10
f1 (ppm)

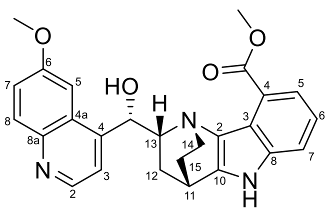

9c

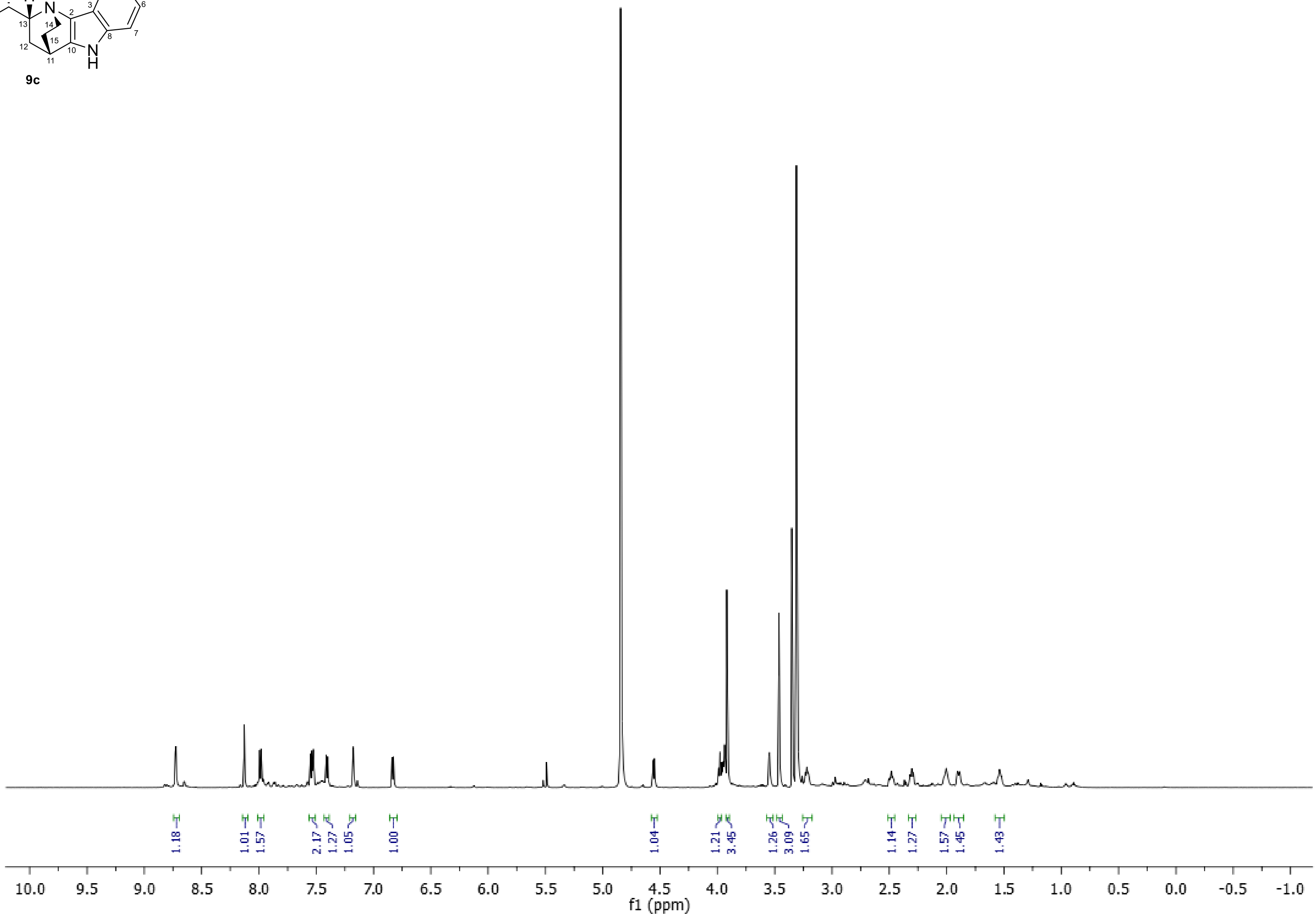
1.18
1.01
1.57
2.17
1.27
1.05
1.00
1.04
1.21
3.45
1.26
3.09
1.65
1.14
1.27
1.57
1.45
1.43
10.0 9.5 9.0 8.5 8.0 7.5 7.0 6.5 6.0 5.5 5.0 4.5 4.0 3.5 3.0 2.5 2.0 1.5 1.0 0.5 0.0 -0.5 -1.0
f1 (ppm)

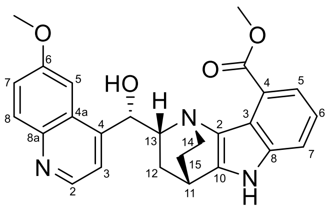

9c

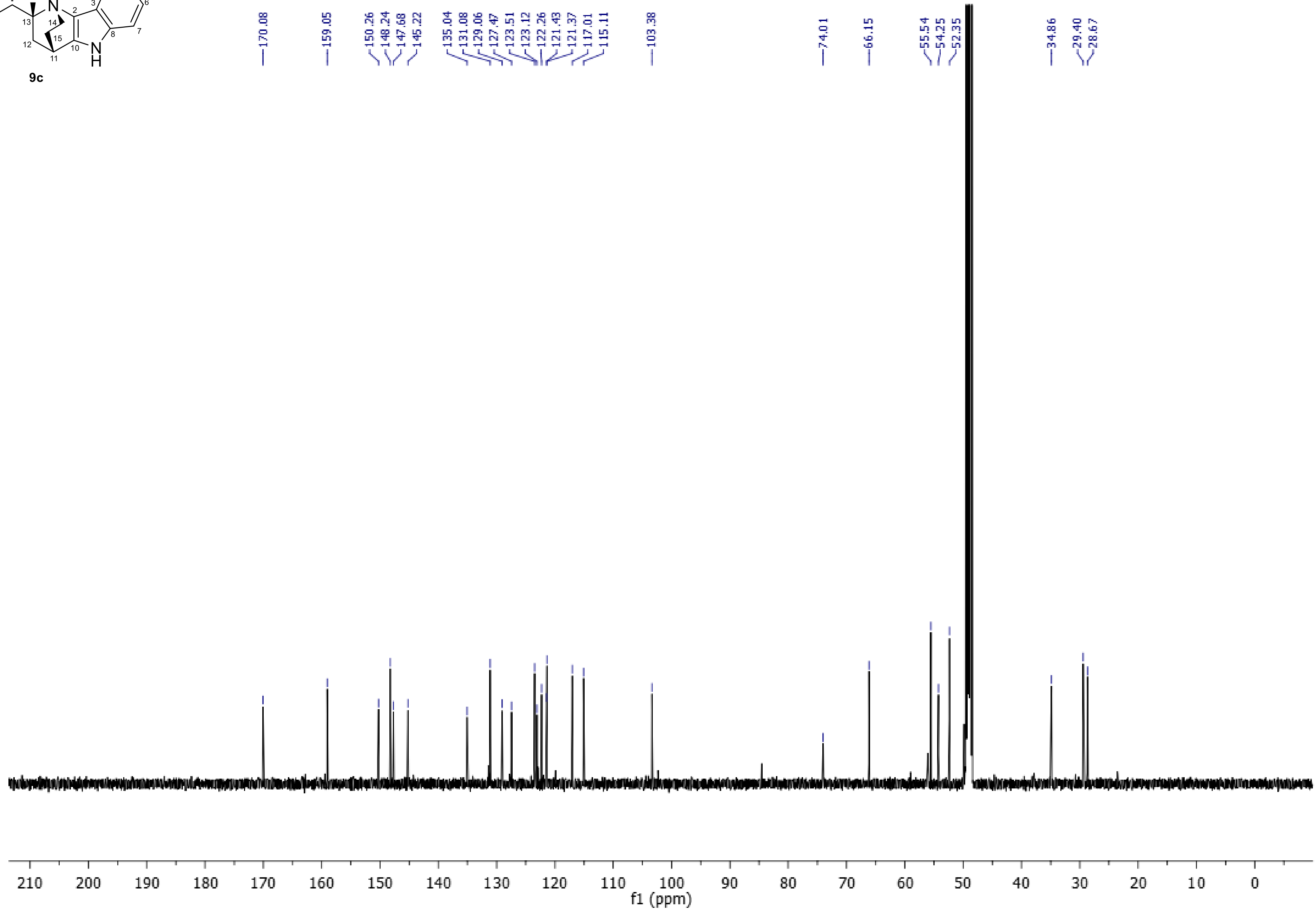
170.08
159.05
150.26
148.24
147.68
145.22
135.04
131.08
129.06
127.47
123.51
123.12
122.26
121.43
121.37
117.01
115.11
103.38
74.01
66.15
55.54
54.25
52.35
34.86
29.40
28.67
210 200 190 180 170 160 150 140 130 120 110 100 90 80 70 60 50 40 30 20 10 0
f1 (ppm)

162

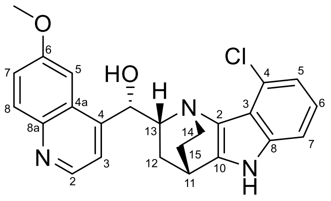

9d

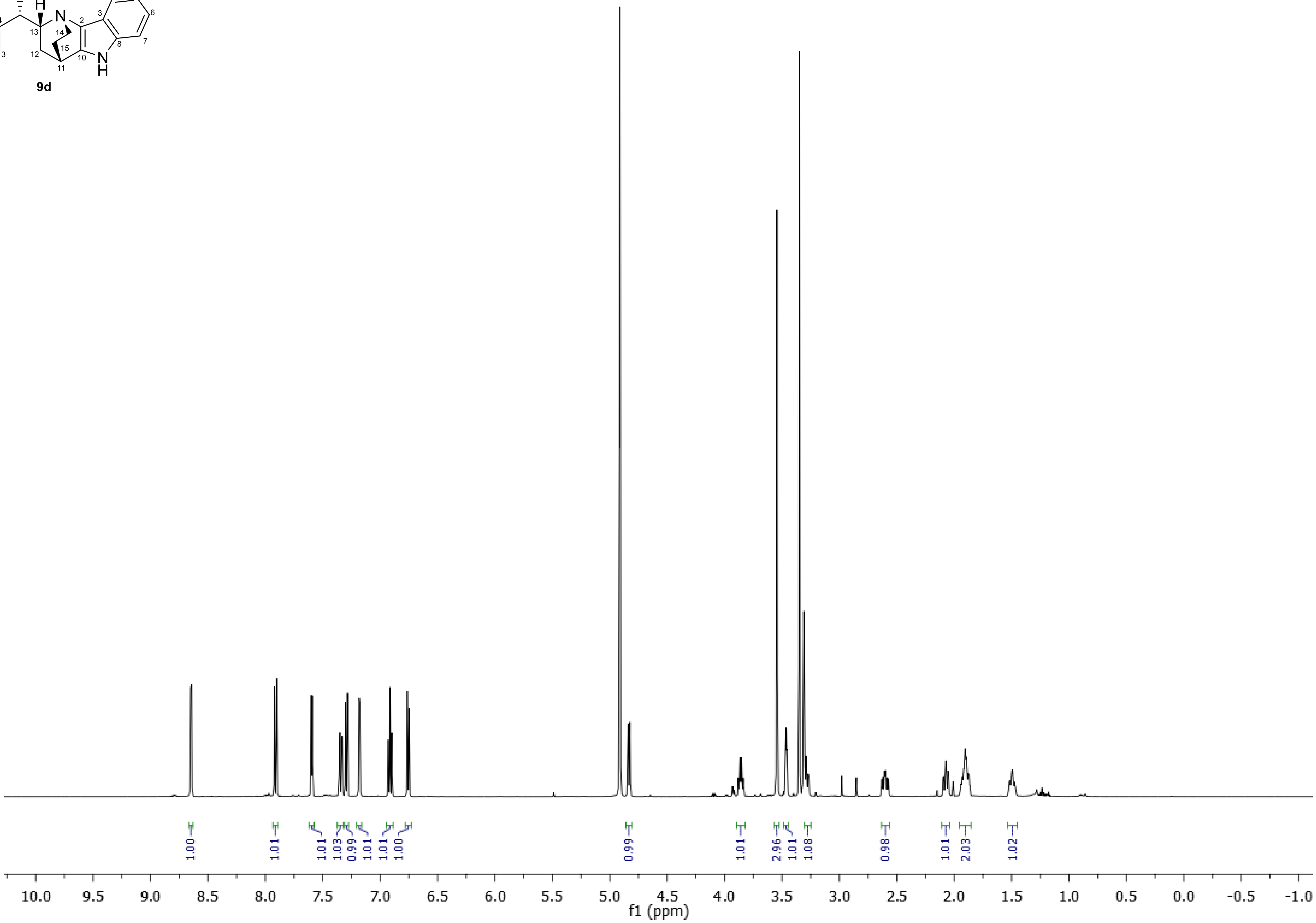
1.00
1.01
1.01
1.03
0.99
1.01
1.01
1.00
0.99
1.01
2.96
1.01
1.08
0.98
1.01
2.03
1.02
10.0
9.5
9.0
8.5
8.0
7.5
7.0
6.5
6.0
5.5
5.0
4.5
4.0
3.5
3.0
2.5
2.0
1.5
1.0
0.5
0.0
-0.5
-1.0
f1 (ppm)

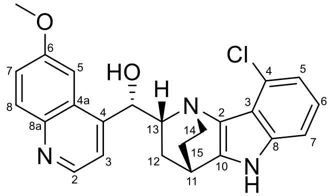

9d

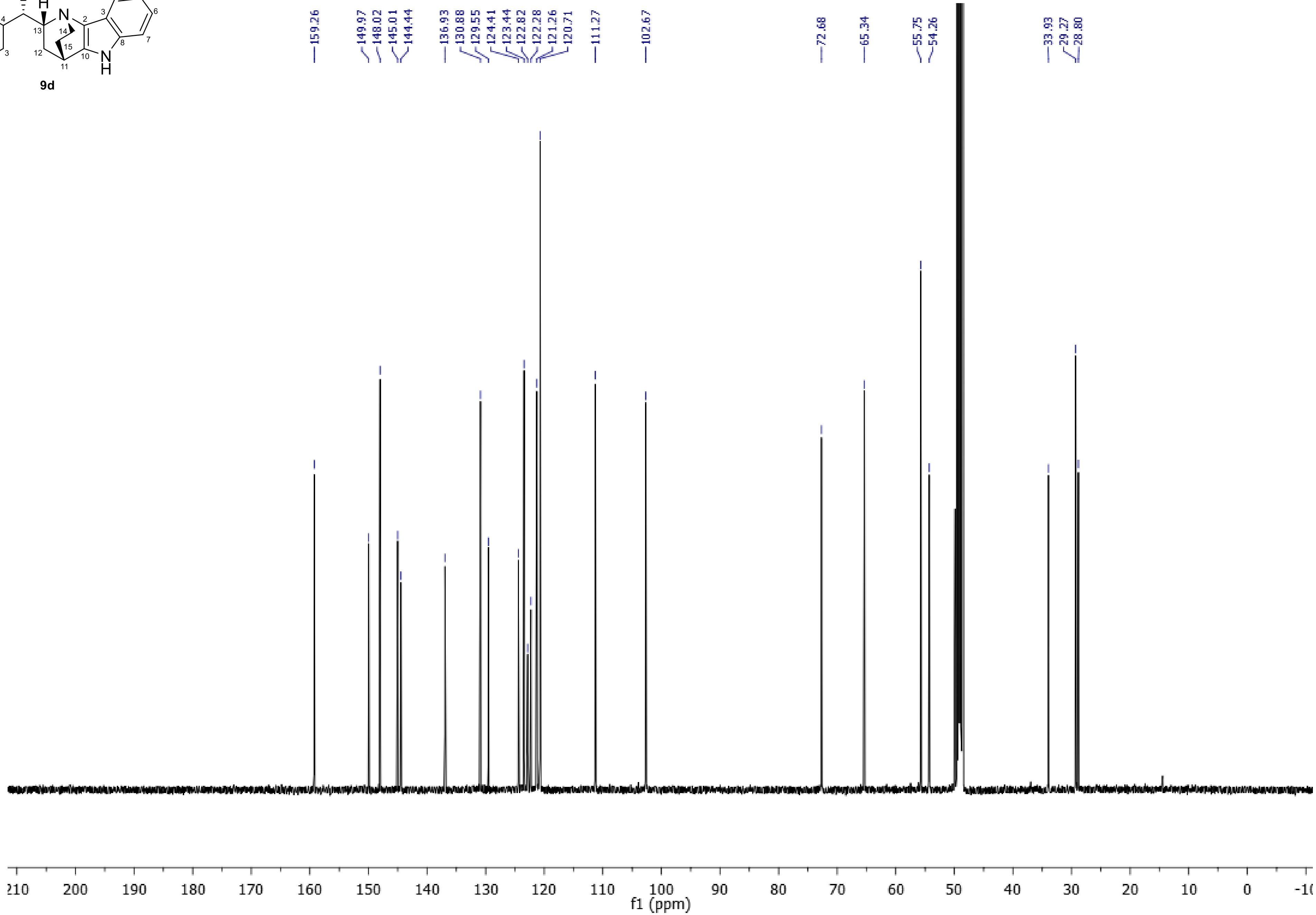
159.26
149.97
148.02
145.01
144.44
136.93
130.88
129.55
124.41
123.44
122.82
122.28
121.26
120.71
111.27
102.67
72.68
65.34
55.75
54.26
33.93
29.27
28.80
f1 (ppm)

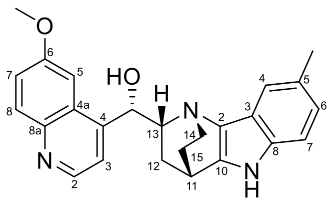
HO
H
N
N
H
O
6
5
7
8
8a
4a
4
3
2
13
14
12
15
11
10
2
3
4
5
6
7
8

9e

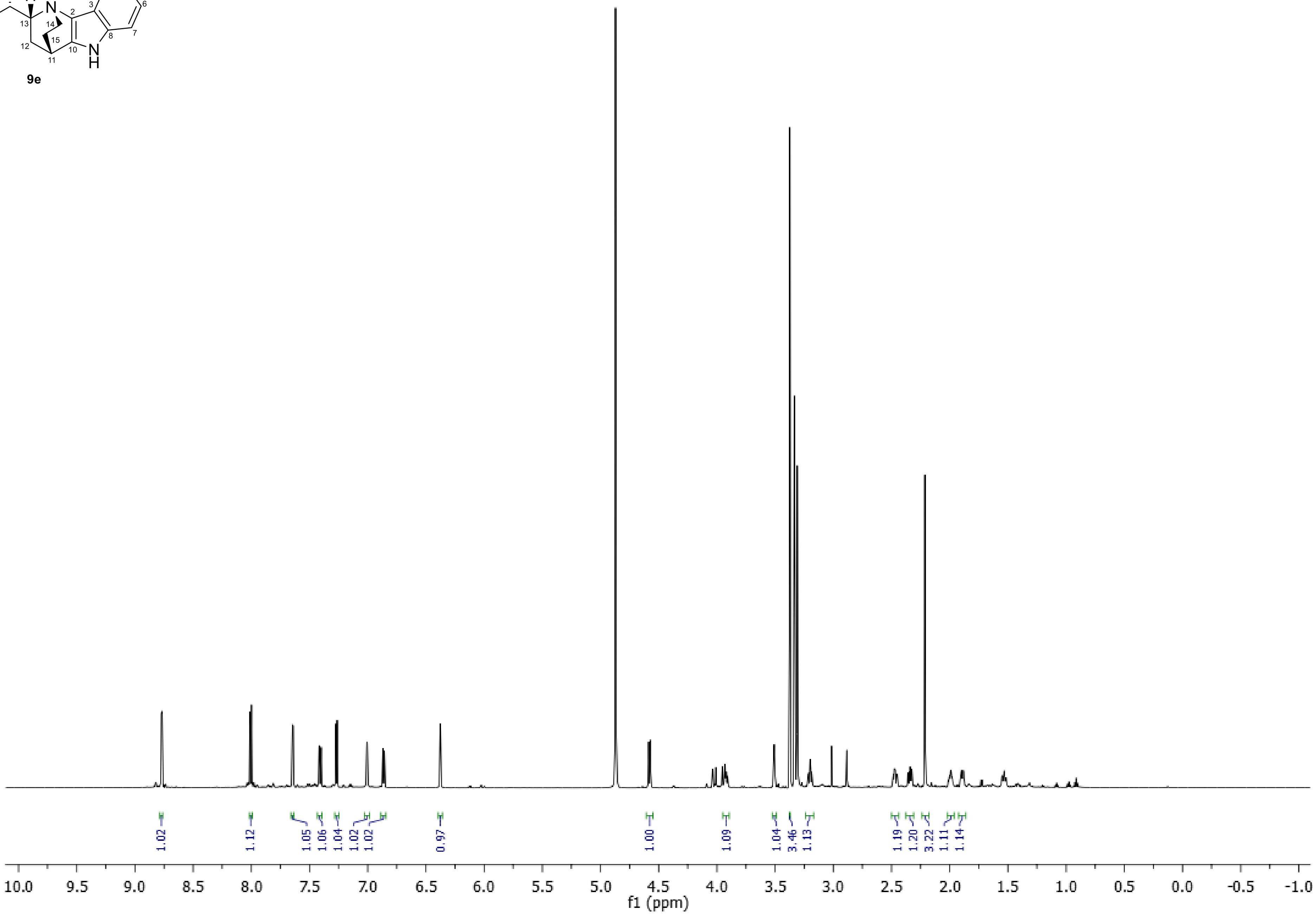
1.02
1.12
1.05
1.06
1.04
1.02
1.02
0.97
1.00
1.09
1.04
3.46
1.13
1.19
1.20
3.22
1.11
1.14
10.0
9.5
9.0
8.5
8.0
7.5
7.0
6.5
6.0
5.5
5.0
4.5
4.0
3.5
3.0
2.5
2.0
1.5
1.0
0.5
0.0
-0.5
-1.0
f1 (ppm)

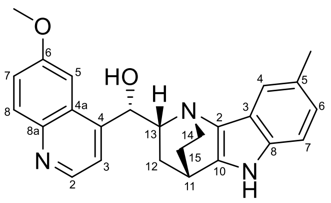

9e

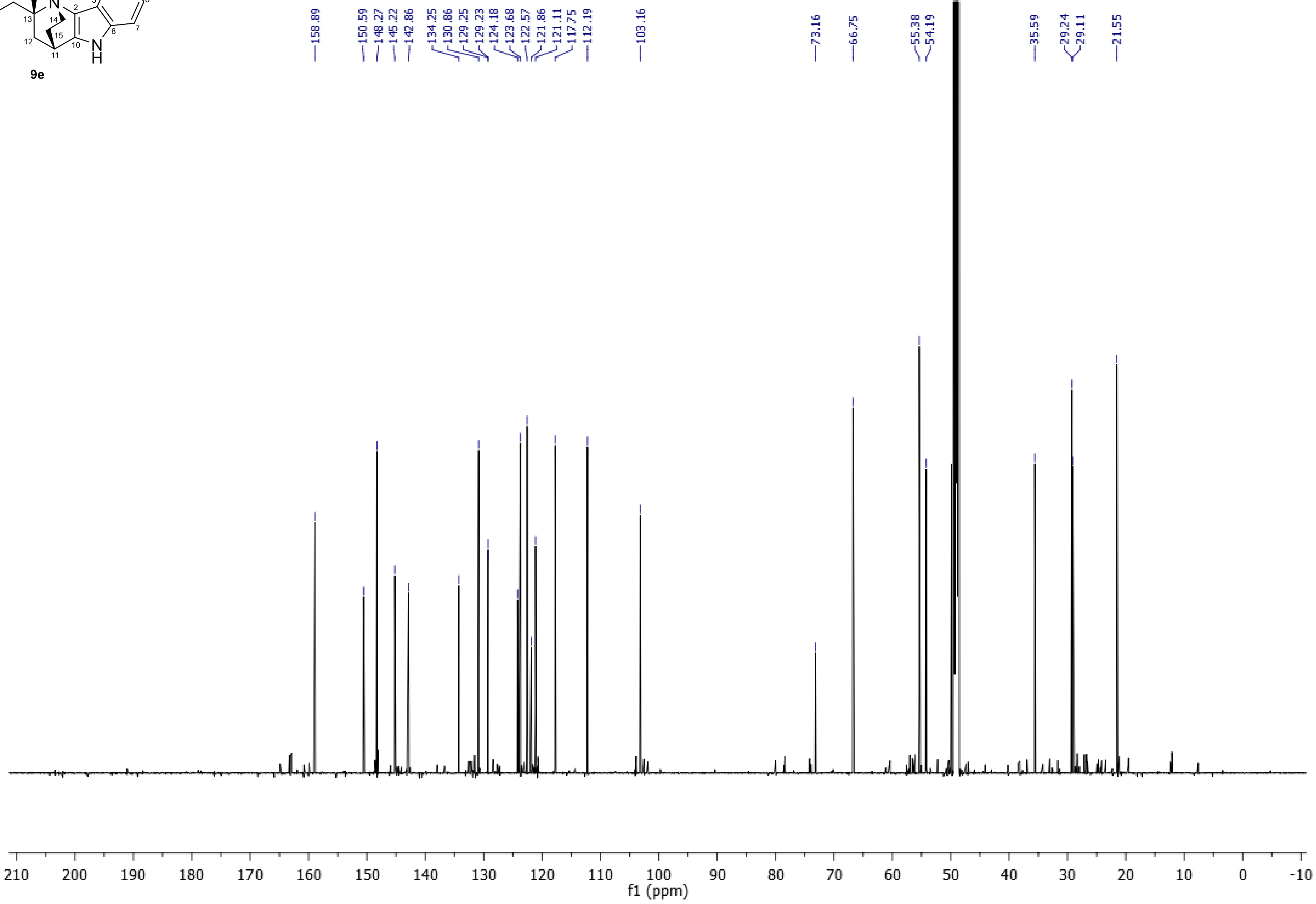
158.89
150.59
148.27
145.22
142.86
134.25
130.86
129.25
129.23
124.18
123.68
122.57
121.86
121.11
117.75
112.19
103.16
73.16
66.75
55.38
54.19
35.59
29.24
29.11
21.55
210 200 190 180 170 160 150 140 130 120 110 100 90 80 70 60 50 40 30 20 10 0 -10
f1 (ppm)

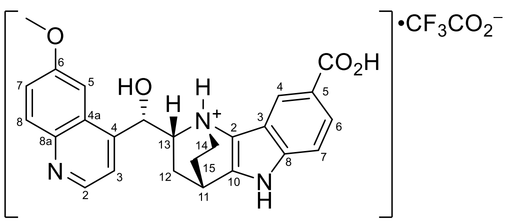
•CF3CO2−
CO2H
HO
H
N+
N
H

9f

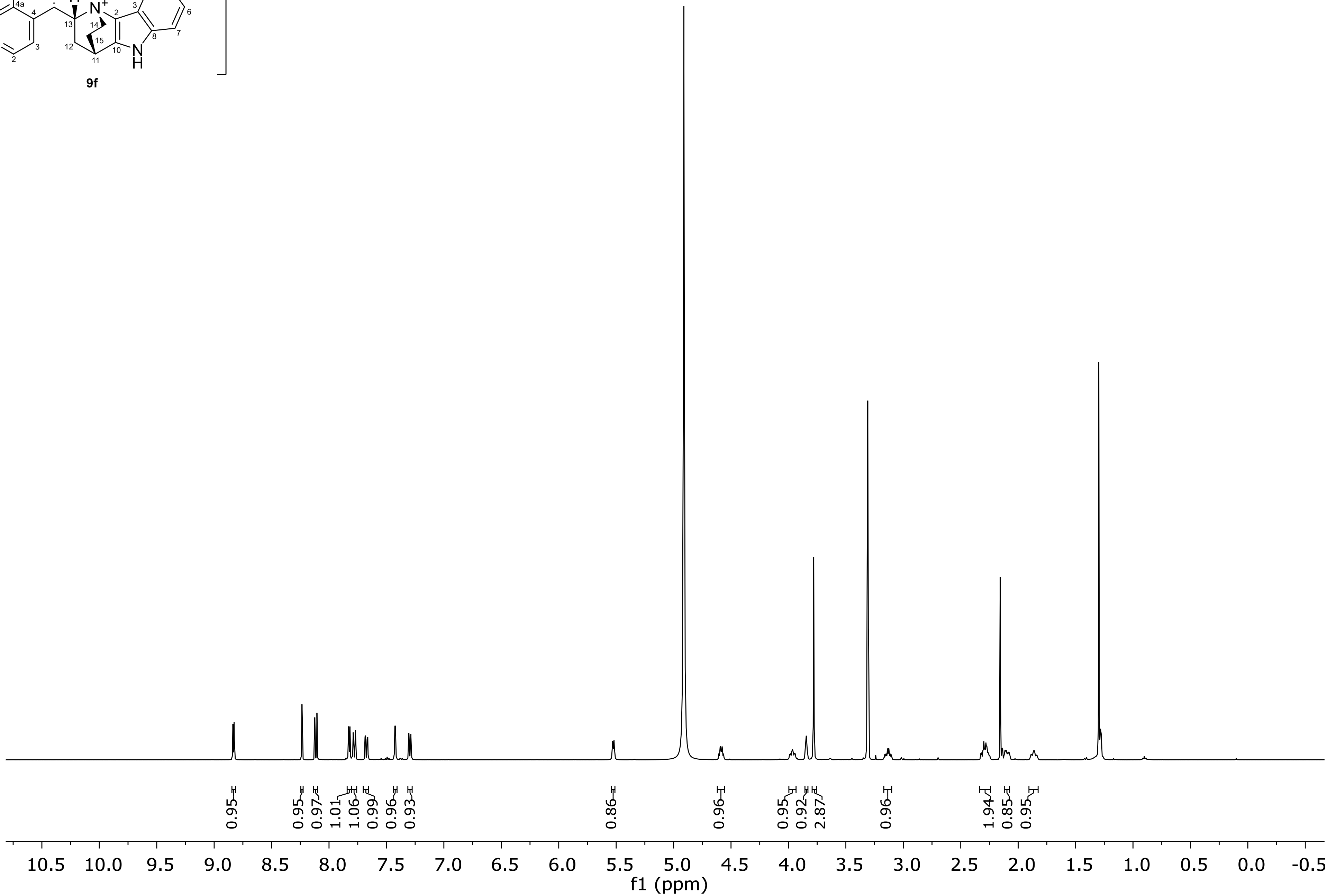
0.95
0.95
0.97
1.01
1.06
0.99
0.96
0.93
0.86
0.96
0.95
0.92
2.87
0.96
1.94
0.85
0.95
10.5 10.0 9.5 9.0 8.5 8.0 7.5 7.0 6.5 6.0 5.5 5.0 4.5 4.0 3.5 3.0 2.5 2.0 1.5 1.0 0.5 0.0 -0.5
f1 (ppm)

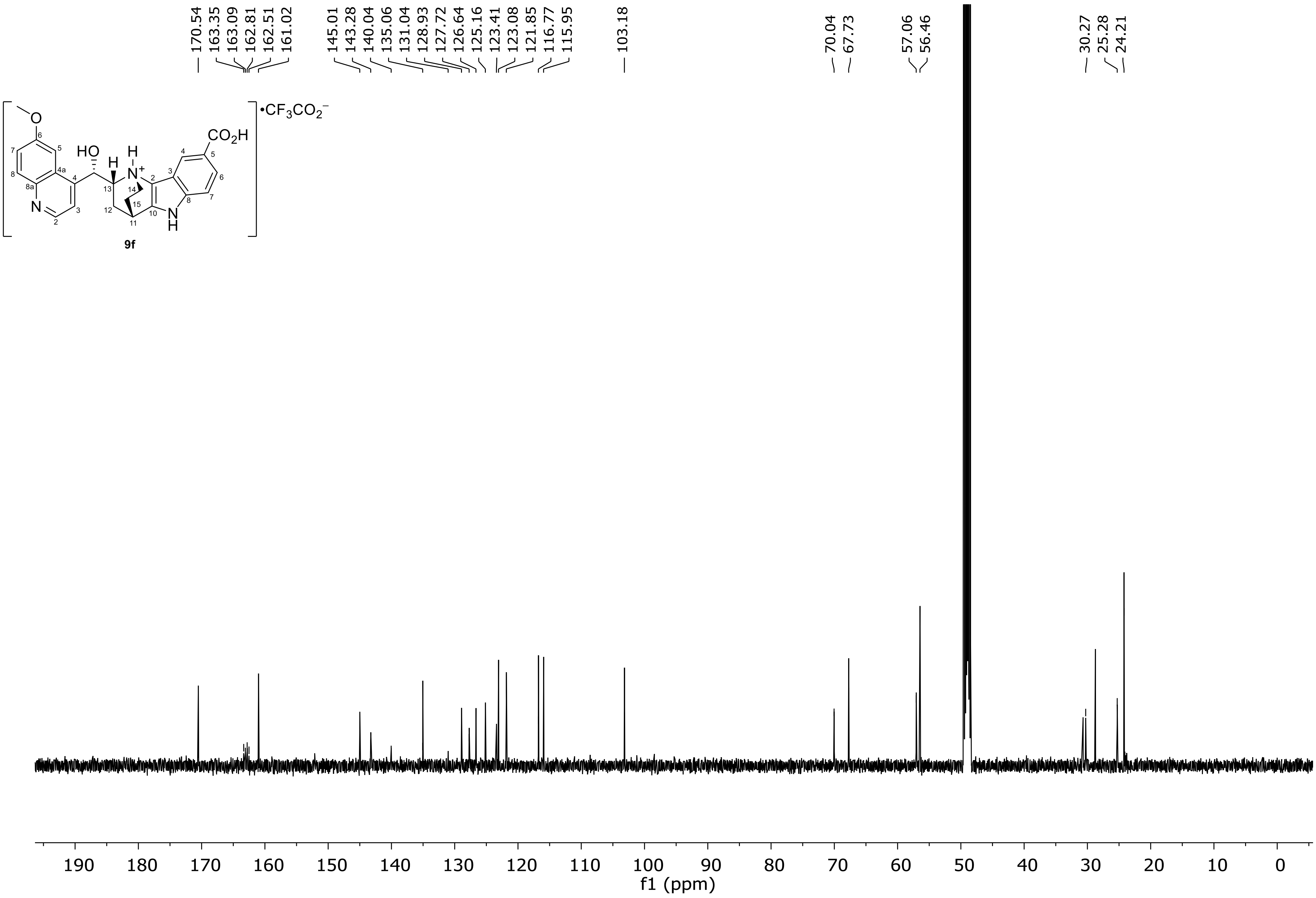

170.54
163.35
163.09
162.81
162.51
161.02
145.01
143.28
140.04
135.06
131.04
128.93
127.72
126.64
125.16
123.41
123.08
121.85
116.77
115.95
103.18
70.04
67.73
57.06
56.46
30.27
25.28
24.21
•$CF_3CO_2^-$
CO_2H
HO
H
N+
N
H
9f
190 180 170 160 150 140 130 120 110 100 90 80 70 60 50 40 30 20 10 0
f1 (ppm)

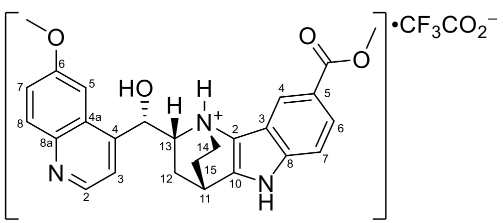

9g

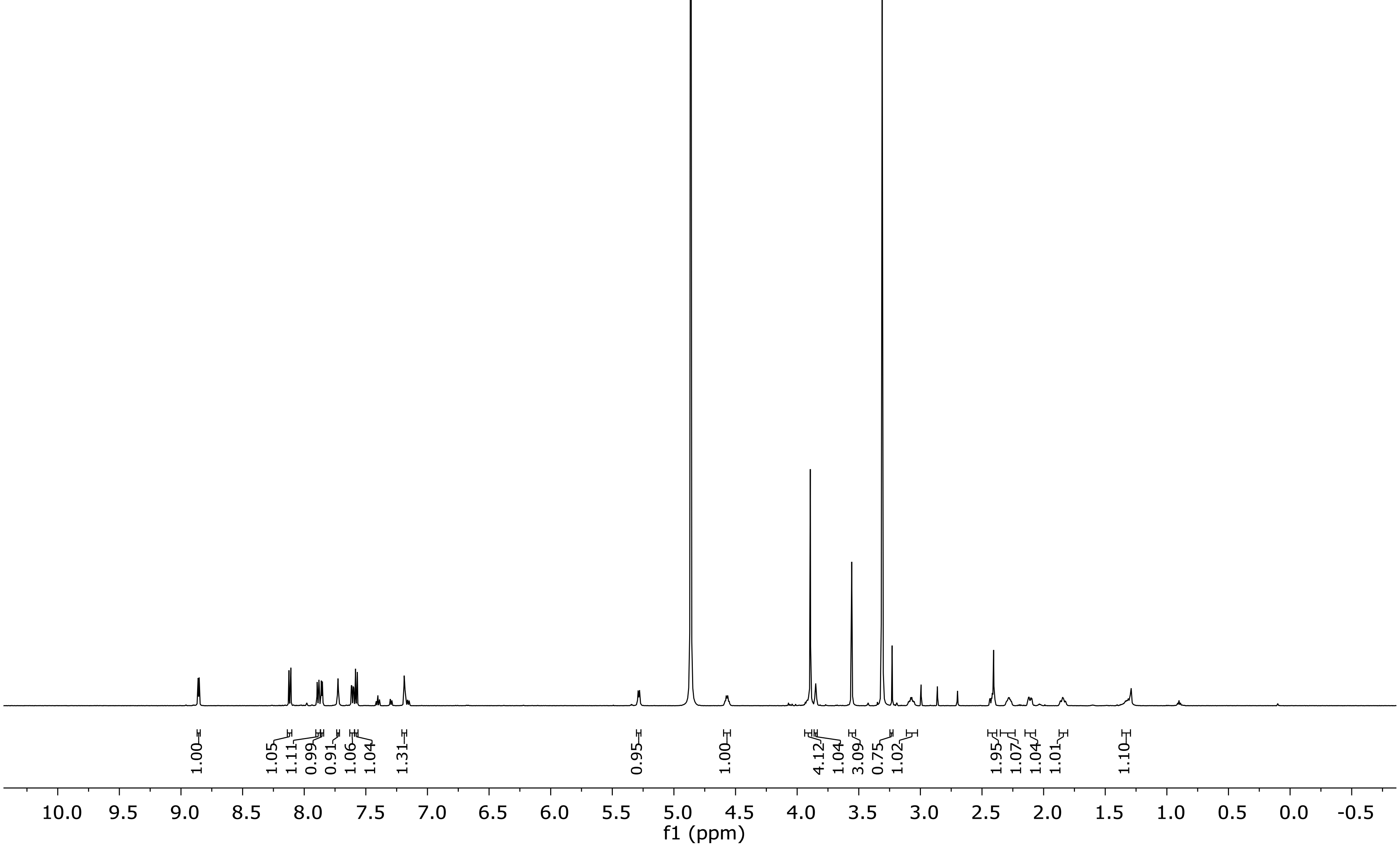
1.00
1.05
1.11
0.99
0.91
1.06
1.04
1.31
0.95
1.00
4.12
1.04
3.09
0.75
1.02
1.95
1.07
1.04
1.01
1.10
10.0 9.5 9.0 8.5 8.0 7.5 7.0 6.5 6.0 5.5 5.0 4.5 4.0 3.5 3.0 2.5 2.0 1.5 1.0 0.5 0.0 -0.5
f1 (ppm)

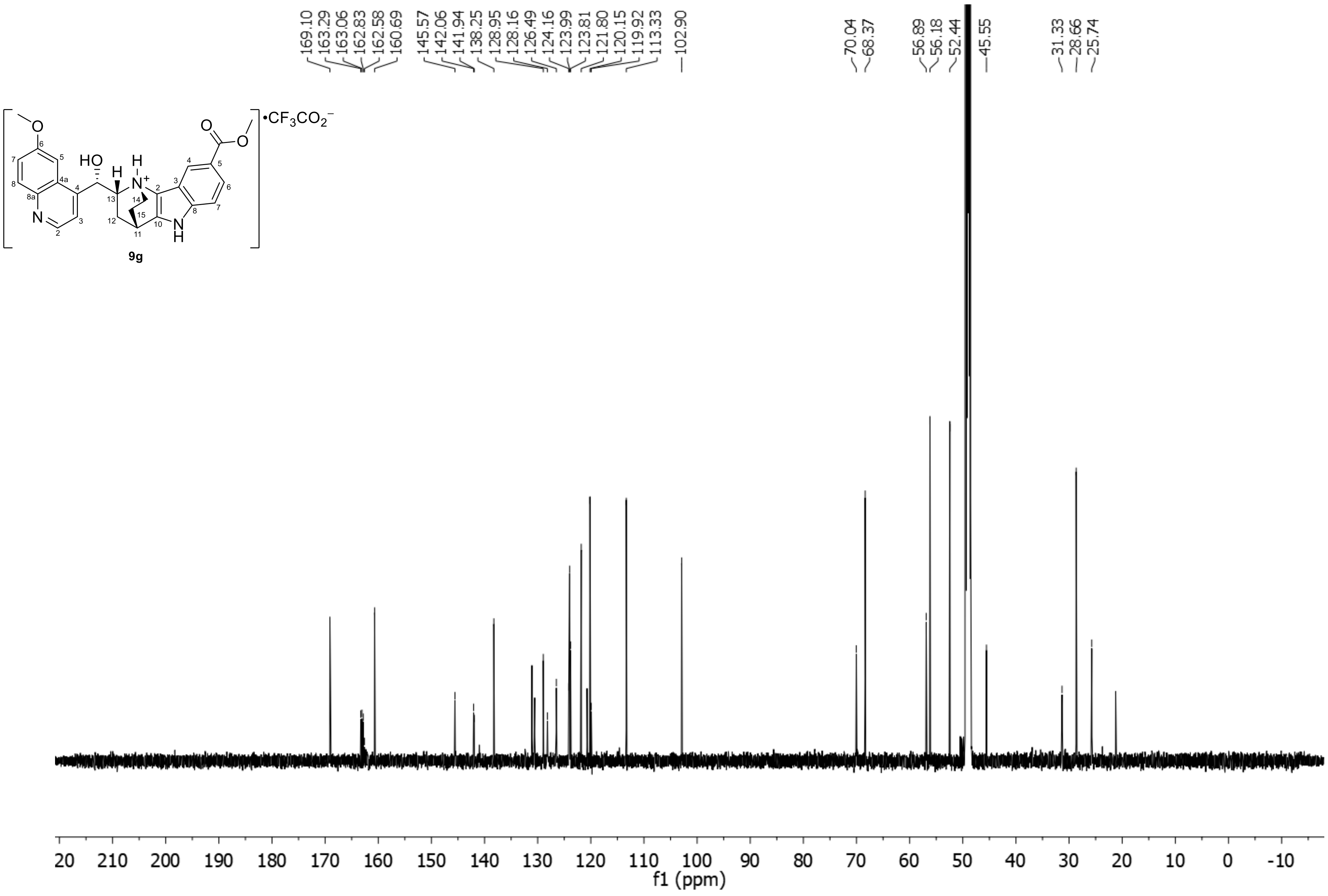
169.10
163.29
163.06
162.83
162.58
160.69
145.57
142.06
141.94
138.25
128.95
128.16
126.49
124.16
123.99
123.81
121.80
120.15
119.92
113.33
102.90
70.04
68.37
56.89
56.18
52.44
45.55
31.33
28.66
25.74
•$CF_3CO_2^-$
9g
f1 (ppm)

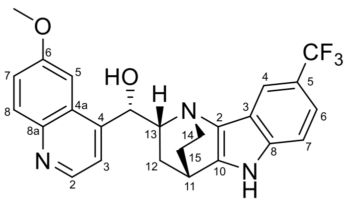
HO
H
N
CF3
N
H

9h

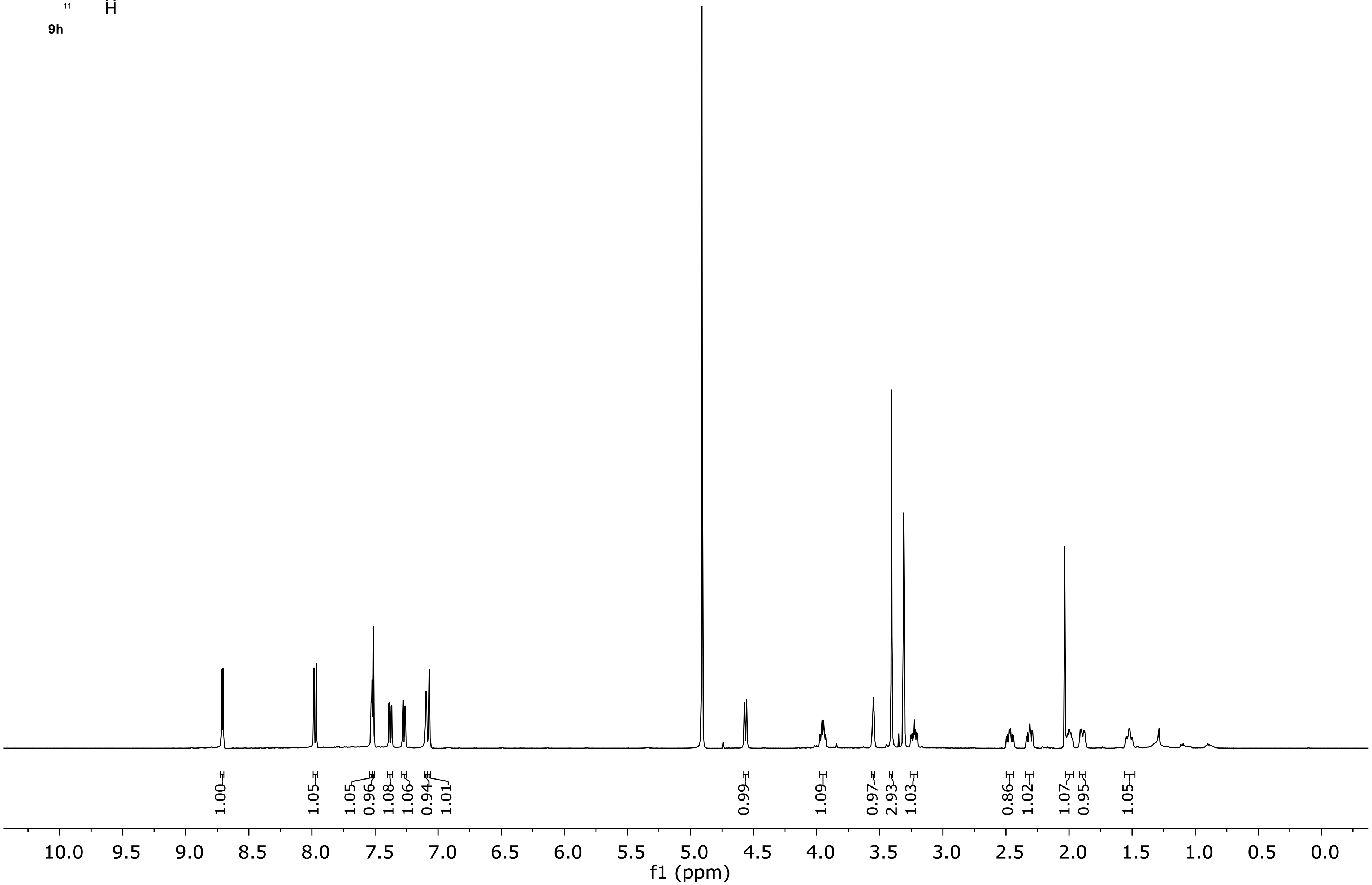
1.00
1.05
1.05
0.96
1.08
1.06
0.94
1.01
0.99
1.09
0.97
2.93
1.03
0.86
1.02
1.07
0.95
1.05
10.0 9.5 9.0 8.5 8.0 7.5 7.0 6.5 6.0 5.5 5.0 4.5 4.0 3.5 3.0 2.5 2.0 1.5 1.0 0.5 0.0
f1 (ppm)

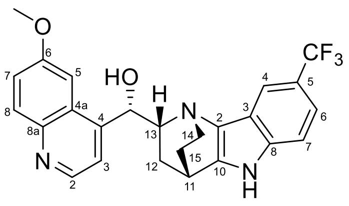

CF3
HO
H
N
H

9h

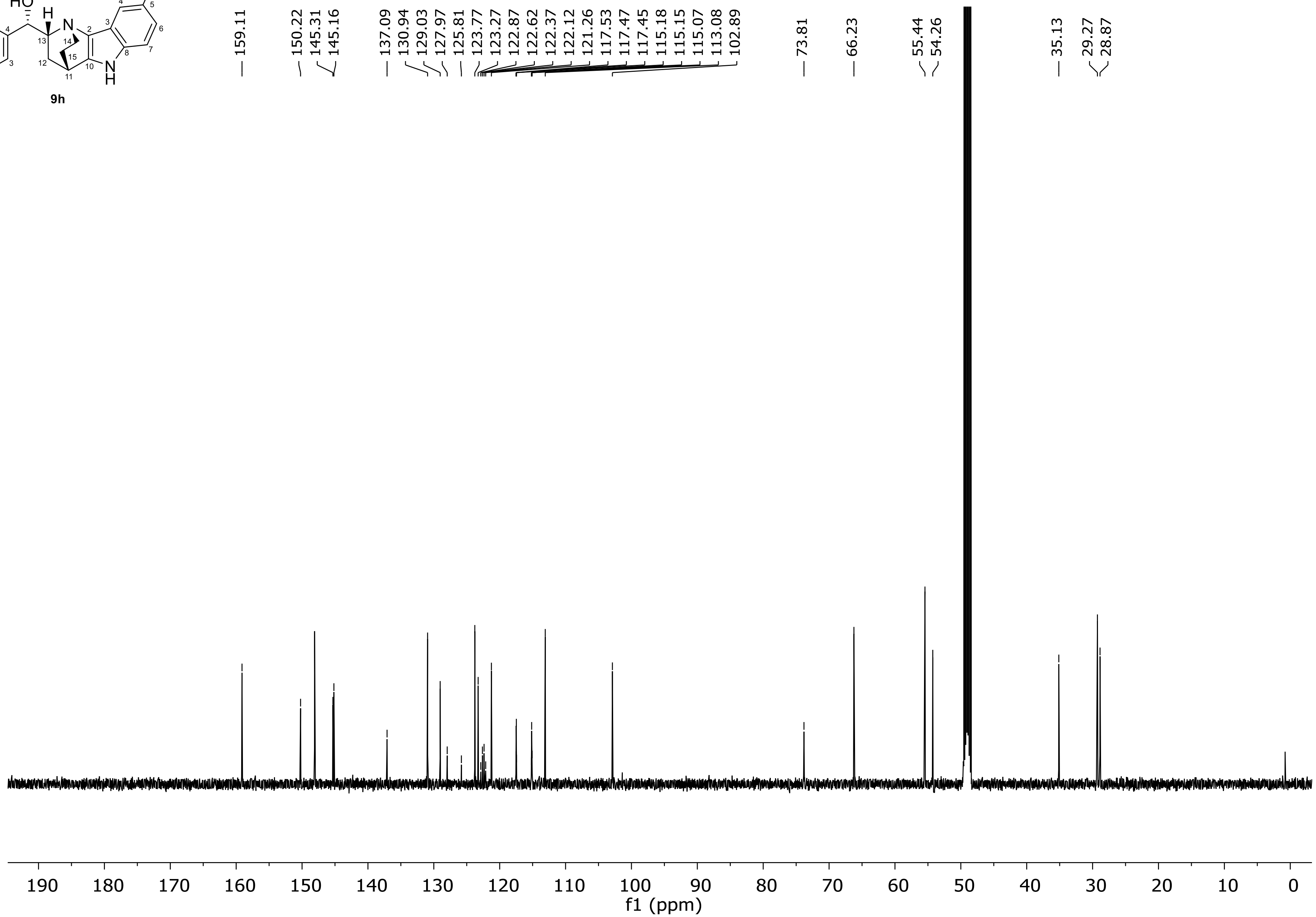

159.11
150.22
145.31
145.16
137.09
130.94
129.03
127.97
125.81
123.77
123.27
122.87
122.62
122.37
122.12
121.26
117.53
117.47
117.45
115.18
115.15
115.07
113.08
102.89
73.81
66.23
55.44
54.26
35.13
29.27
28.87
f1 (ppm)

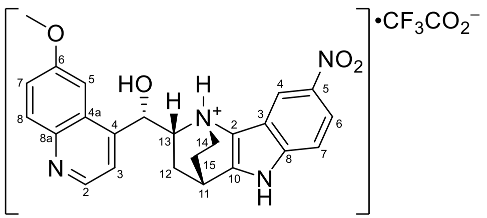

9i

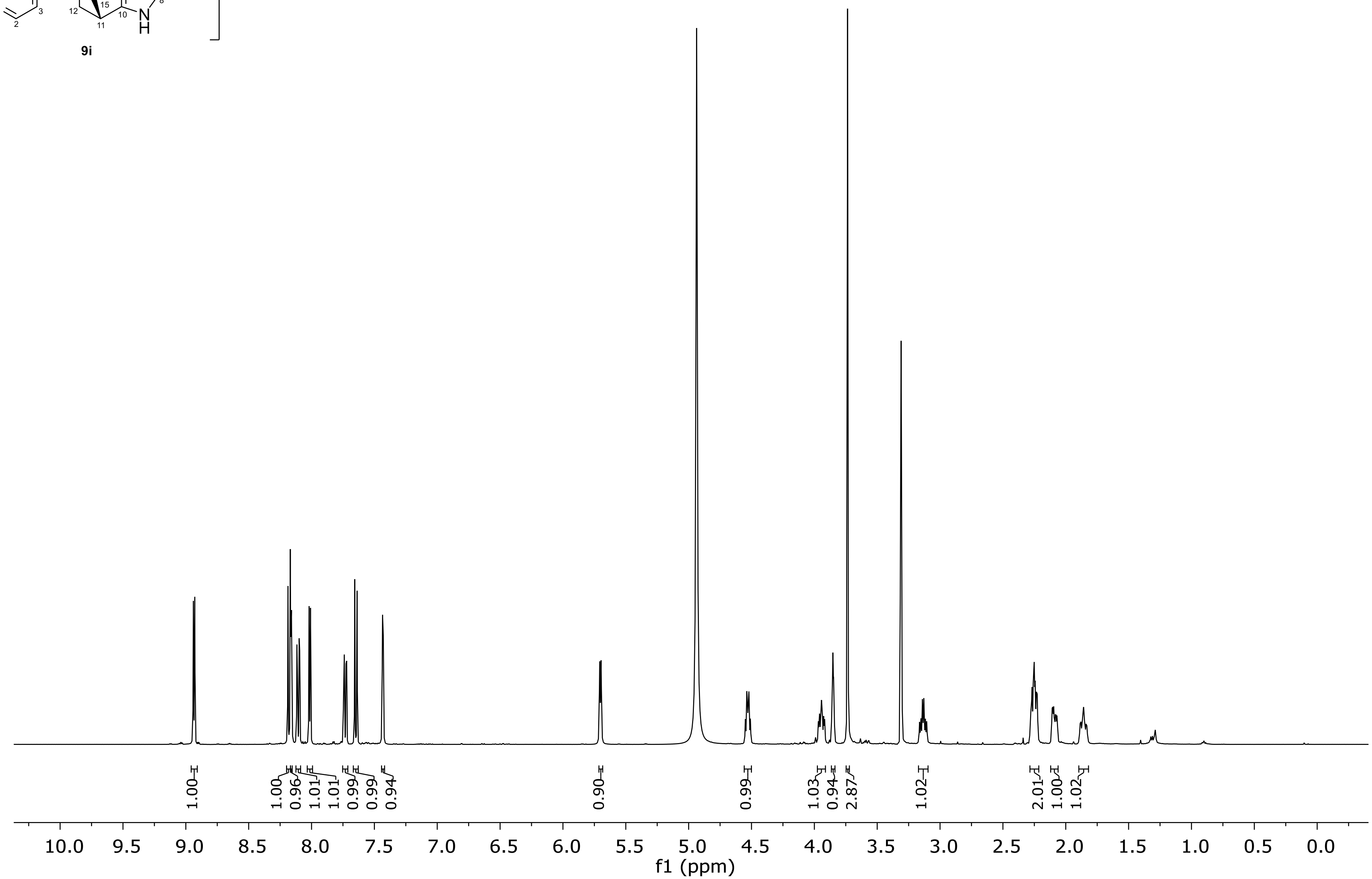
1.00
1.00
0.96
1.01
1.01
0.99
0.99
0.94
0.90
0.99
1.03
0.94
2.87
1.02
2.01
1.00
1.02
10.0 9.5 9.0 8.5 8.0 7.5 7.0 6.5 6.0 5.5 5.0 4.5 4.0 3.5 3.0 2.5 2.0 1.5 1.0 0.5 0.0
f1 (ppm)

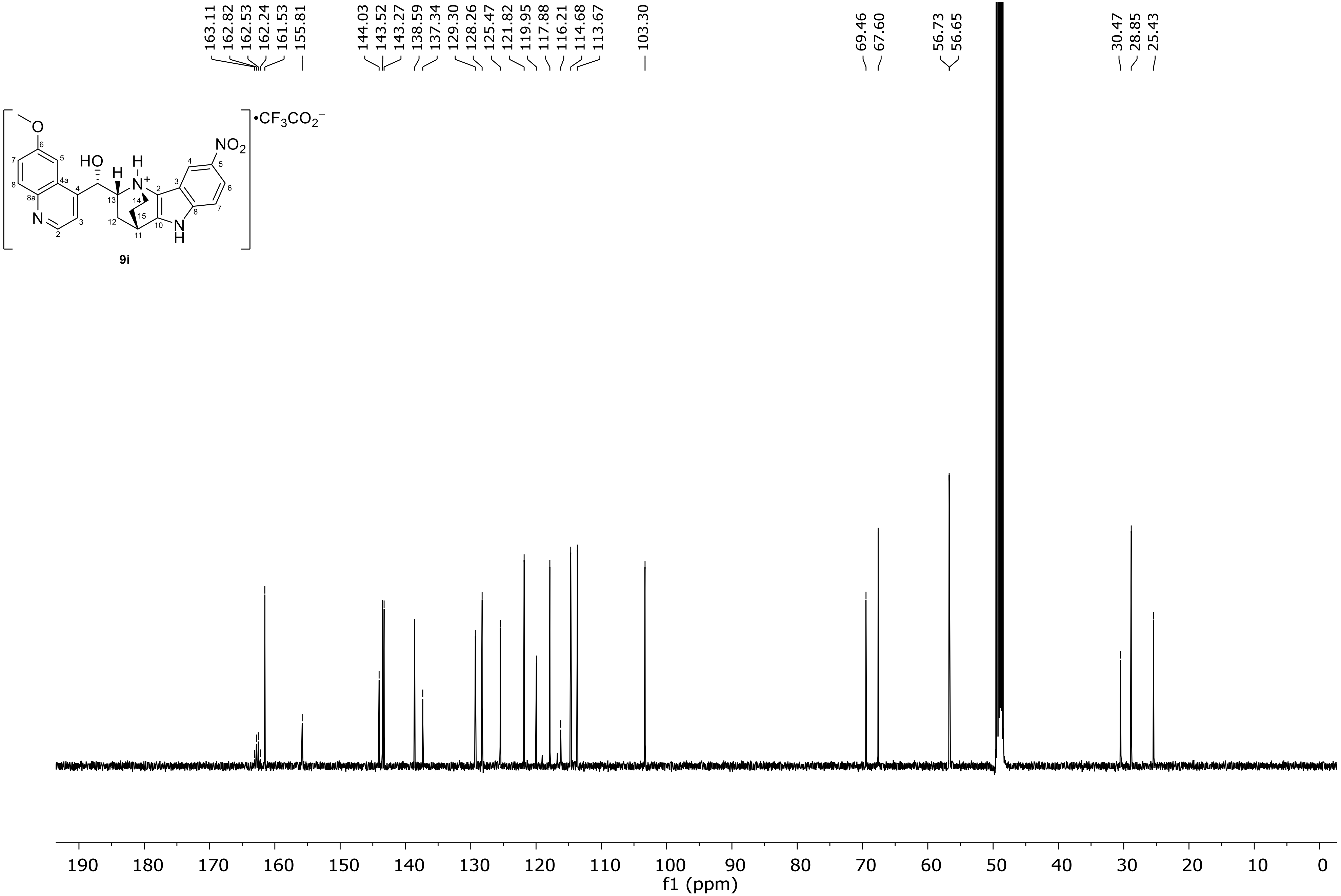
163.11
162.82
162.53
162.24
161.53
155.81
144.03
143.52
143.27
138.59
137.34
129.30
128.26
125.47
121.82
119.95
117.88
116.21
114.68
113.67
103.30
69.46
67.60
56.73
56.65
30.47
28.85
25.43
•CF3CO2−
NO2
HO
H
N+
NH
9i
190 180 170 160 150 140 130 120 110 100 90 80 70 60 50 40 30 20 10 0
f1 (ppm)

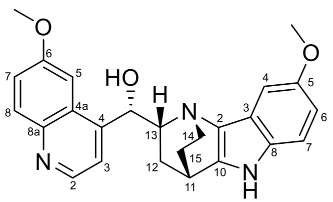
HO
H
N
N
H
O
O

9j

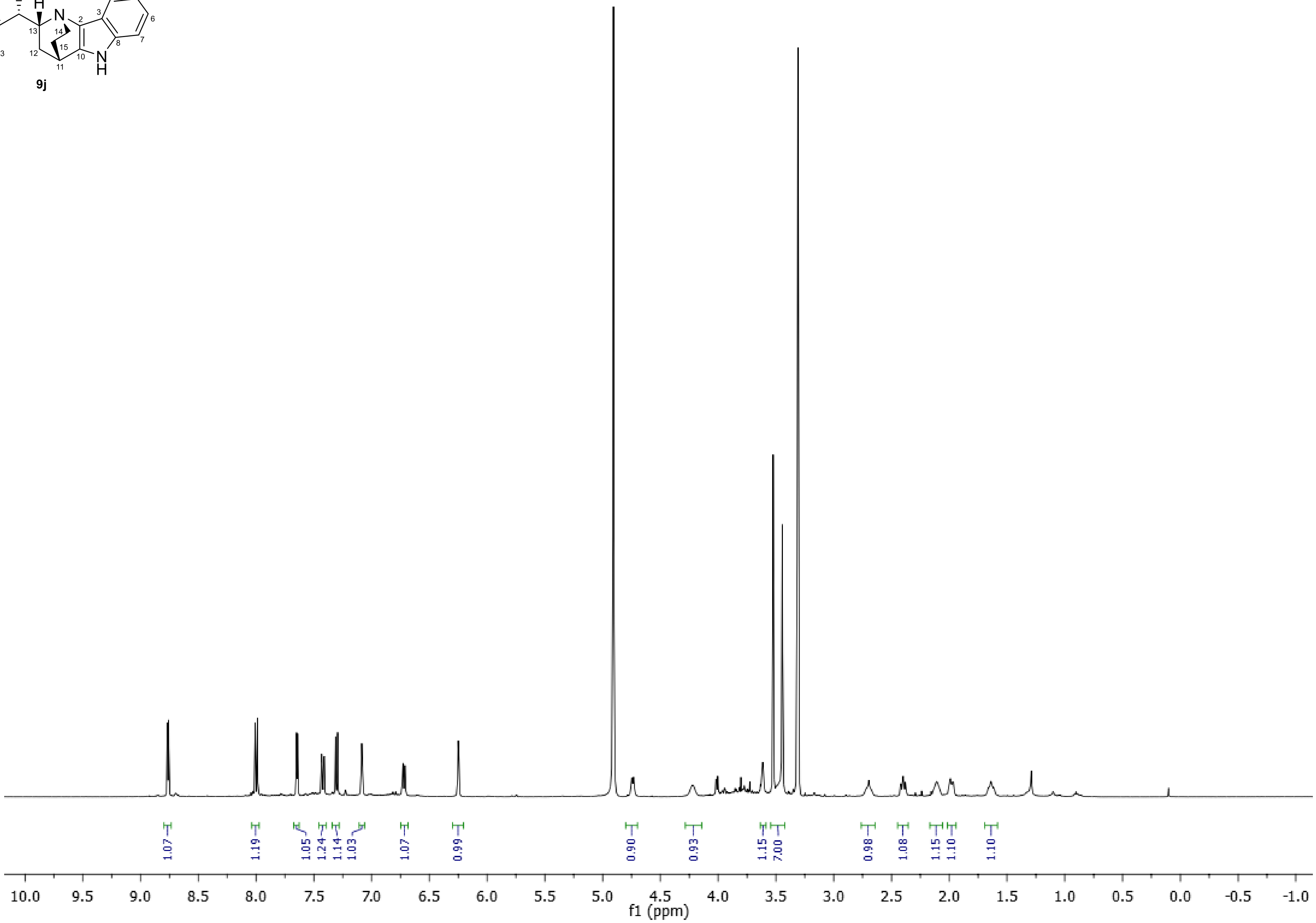
1.07
1.19
1.05
1.24
1.14
1.03
1.07
0.99
0.90
0.93
1.15
7.00
0.98
1.08
1.15
1.10
1.10
10.0 9.5 9.0 8.5 8.0 7.5 7.0 6.5 6.0 5.5 5.0 4.5 4.0 3.5 3.0 2.5 2.0 1.5 1.0 0.5 0.0 -0.5 -1.0
f1 (ppm)

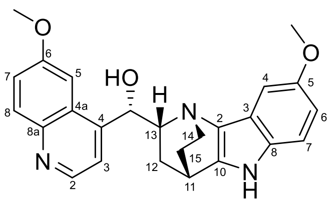
HO
H
N
NH
O
O

9j

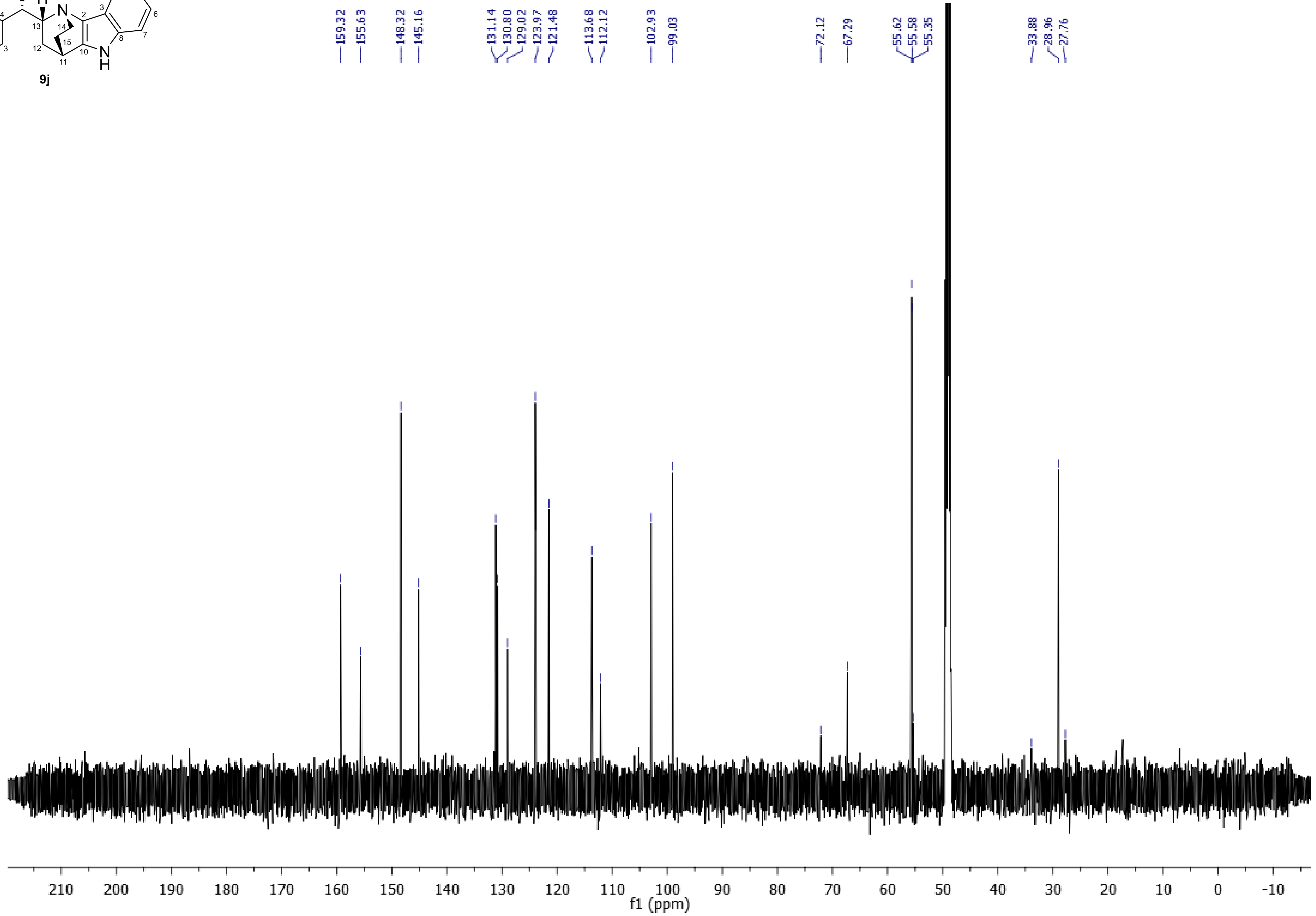
159.32
155.63
148.32
145.16
131.14
130.80
129.02
123.97
121.48
113.68
112.12
102.93
99.03
72.12
67.29
55.62
55.58
55.35
33.88
28.96
27.76
210 200 190 180 170 160 150 140 130 120 110 100 90 80 70 60 50 40 30 20 10 0 -10
f1 (ppm)

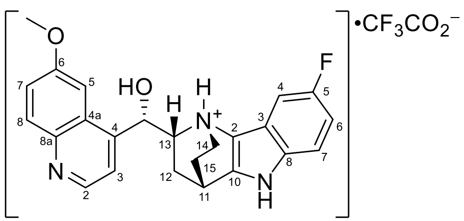

9l

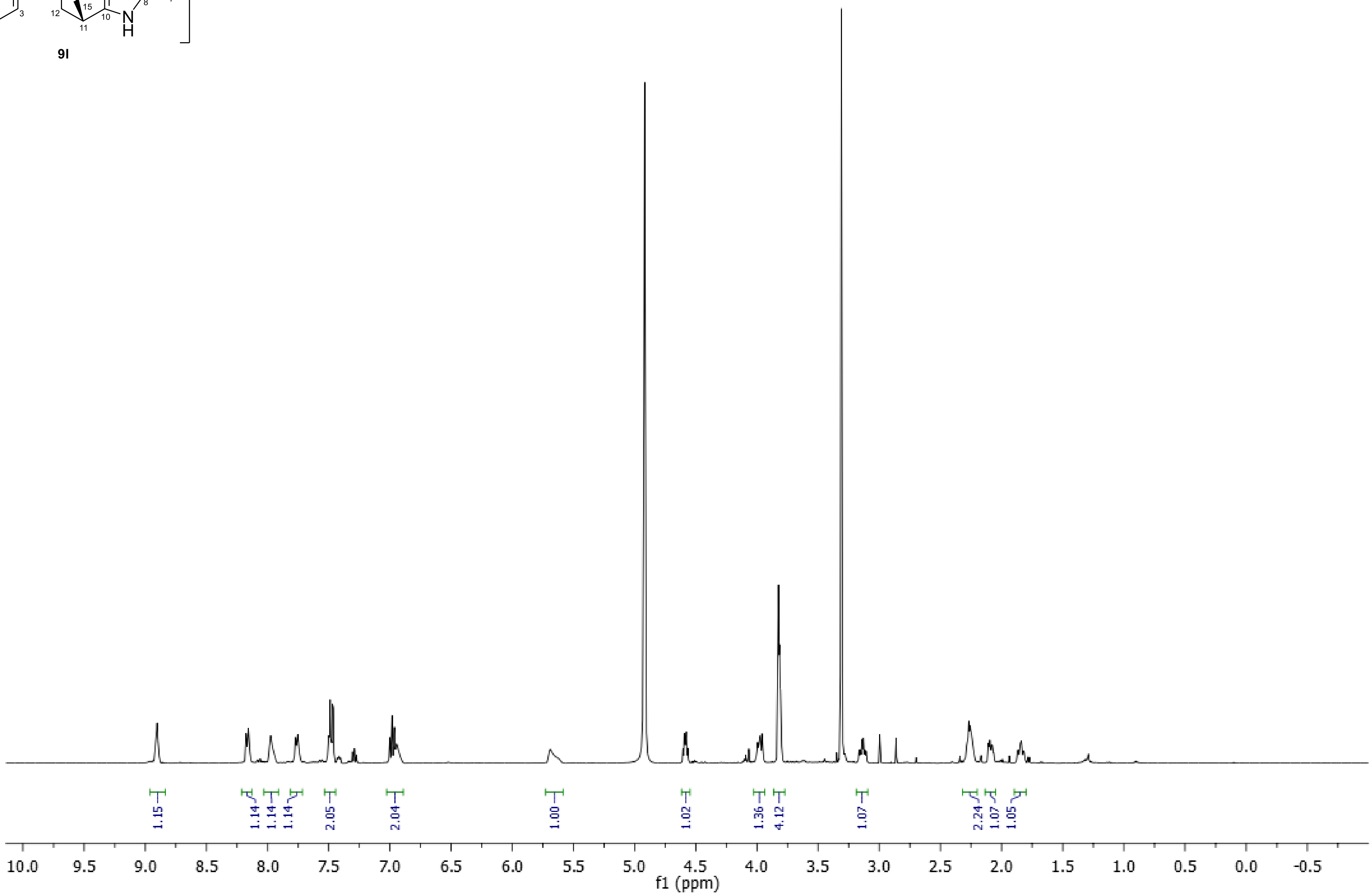

1.15
1.14
1.14
1.14
2.05
2.04
1.00
1.02
1.36
4.12
1.07
2.24
1.07
1.05
10.0 9.5 9.0 8.5 8.0 7.5 7.0 6.5 6.0 5.5 5.0 4.5 4.0 3.5 3.0 2.5 2.0 1.5 1.0 0.5 0.0 -0.5
f1 (ppm)

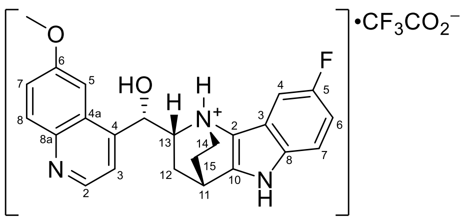
•$CF_3CO_2^-$
HO
H
N+
F
N
NH

9l

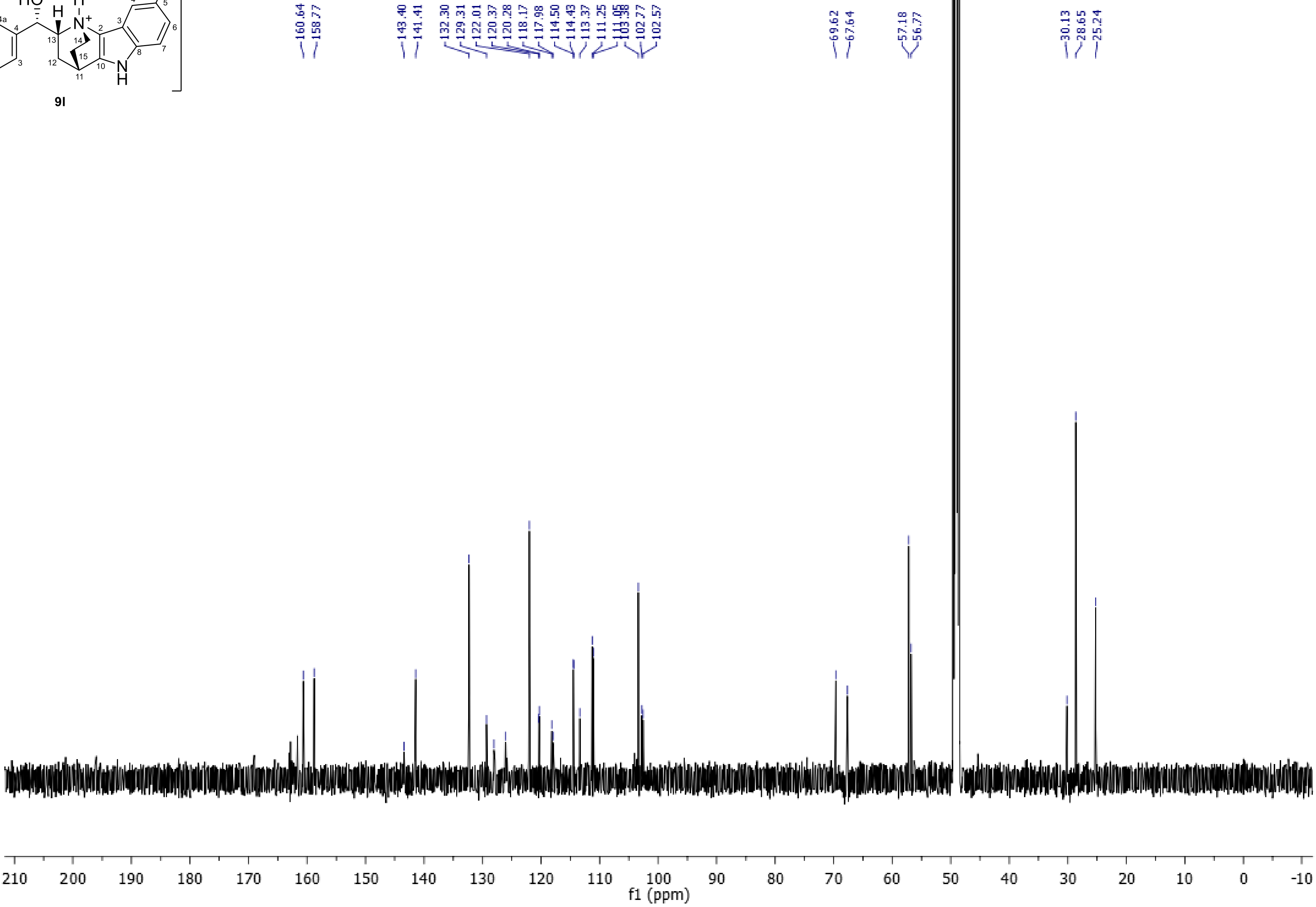
160.64
158.77
143.40
141.41
132.30
129.31
122.01
120.37
120.28
118.17
117.98
114.50
114.43
113.37
111.25
111.05
103.38
102.77
102.57
69.62
67.64
57.18
56.77
30.13
28.65
25.24
210 200 190 180 170 160 150 140 130 120 110 100 90 80 70 60 50 40 30 20 10 0 -10
f1 (ppm)

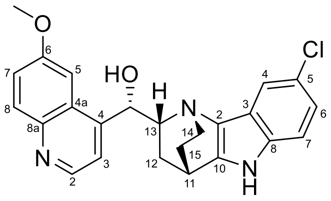

9m

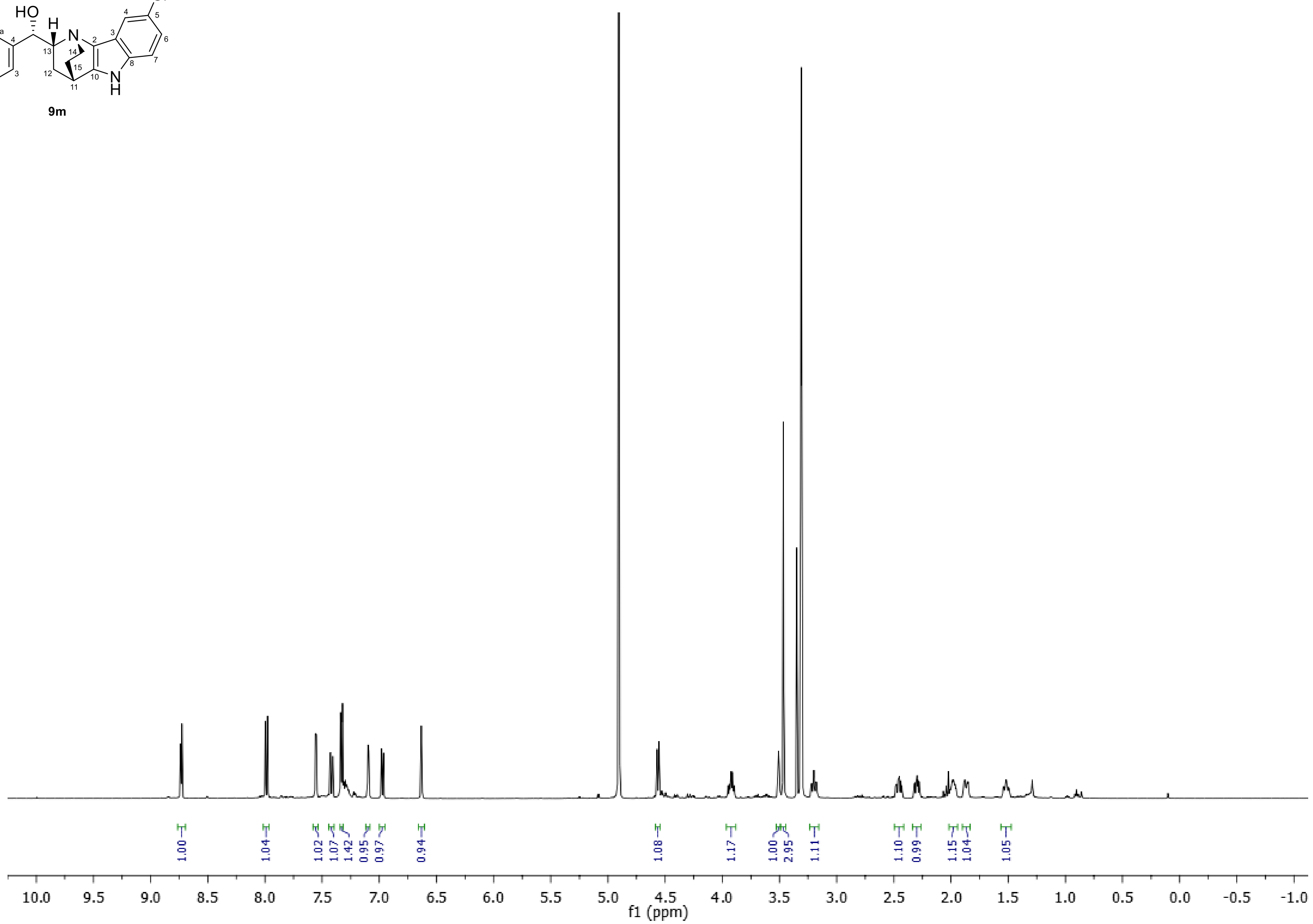
1.00
1.04
1.02
1.07
1.42
0.95
0.97
0.94
1.08
1.17
1.00
2.95
1.11
1.10
0.99
1.15
1.04
1.05
10.0
9.5
9.0
8.5
8.0
7.5
7.0
6.5
6.0
5.5
5.0
4.5
4.0
3.5
3.0
2.5
2.0
1.5
1.0
0.5
0.0
-0.5
-1.0
f1 (ppm)

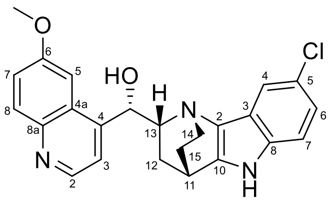
HO
H
N
Cl
N
H

9m

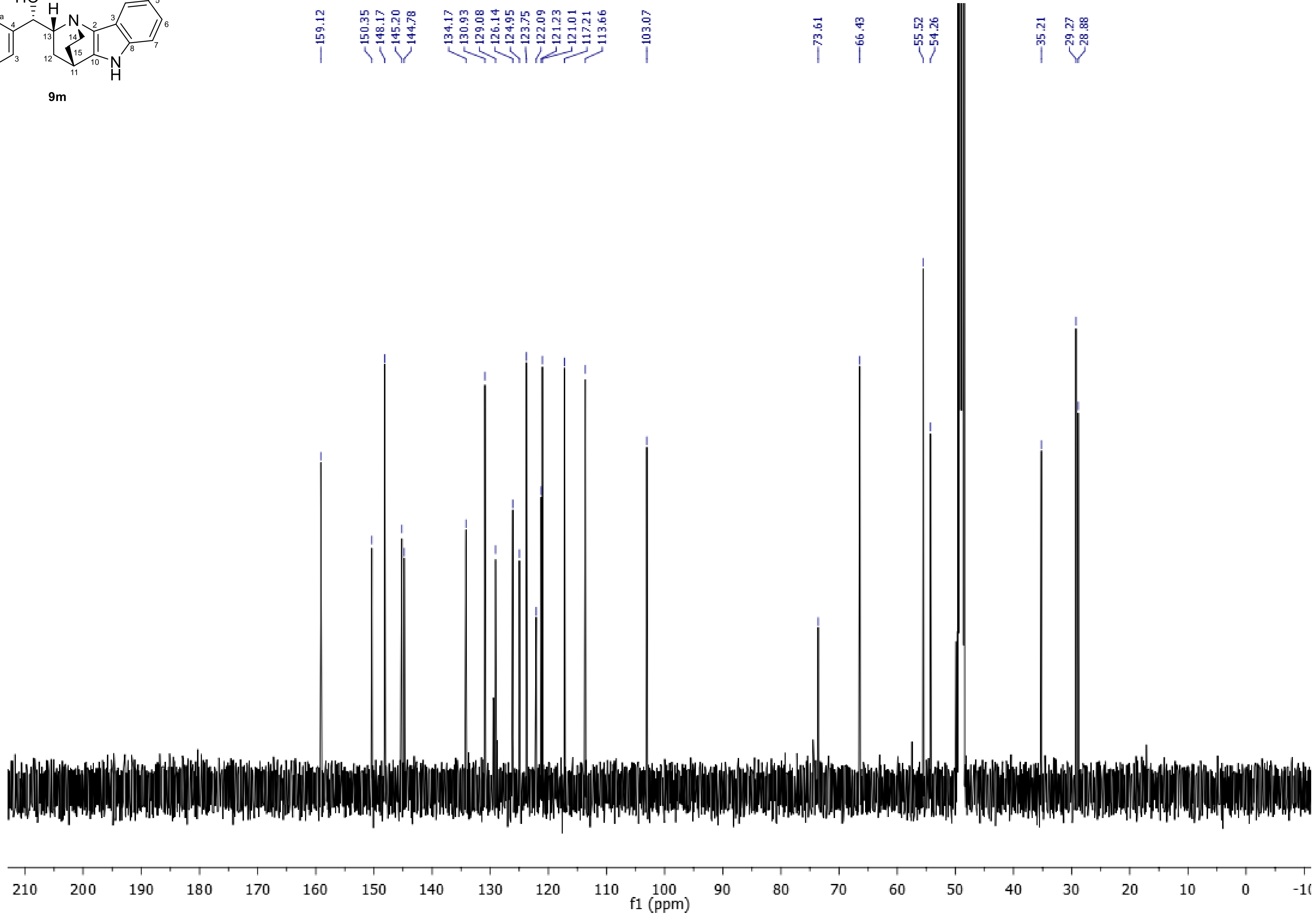
159.12
150.35
148.17
145.20
144.78
134.17
130.93
129.08
126.14
124.95
123.75
122.09
121.23
121.01
117.21
113.66
103.07
73.61
66.43
55.52
54.26
35.21
29.27
28.88
f1 (ppm)

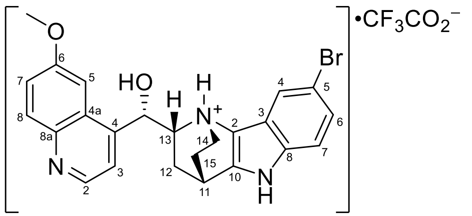
•CF3CO2−
HO
H
N+
Br
N
H
N

9n

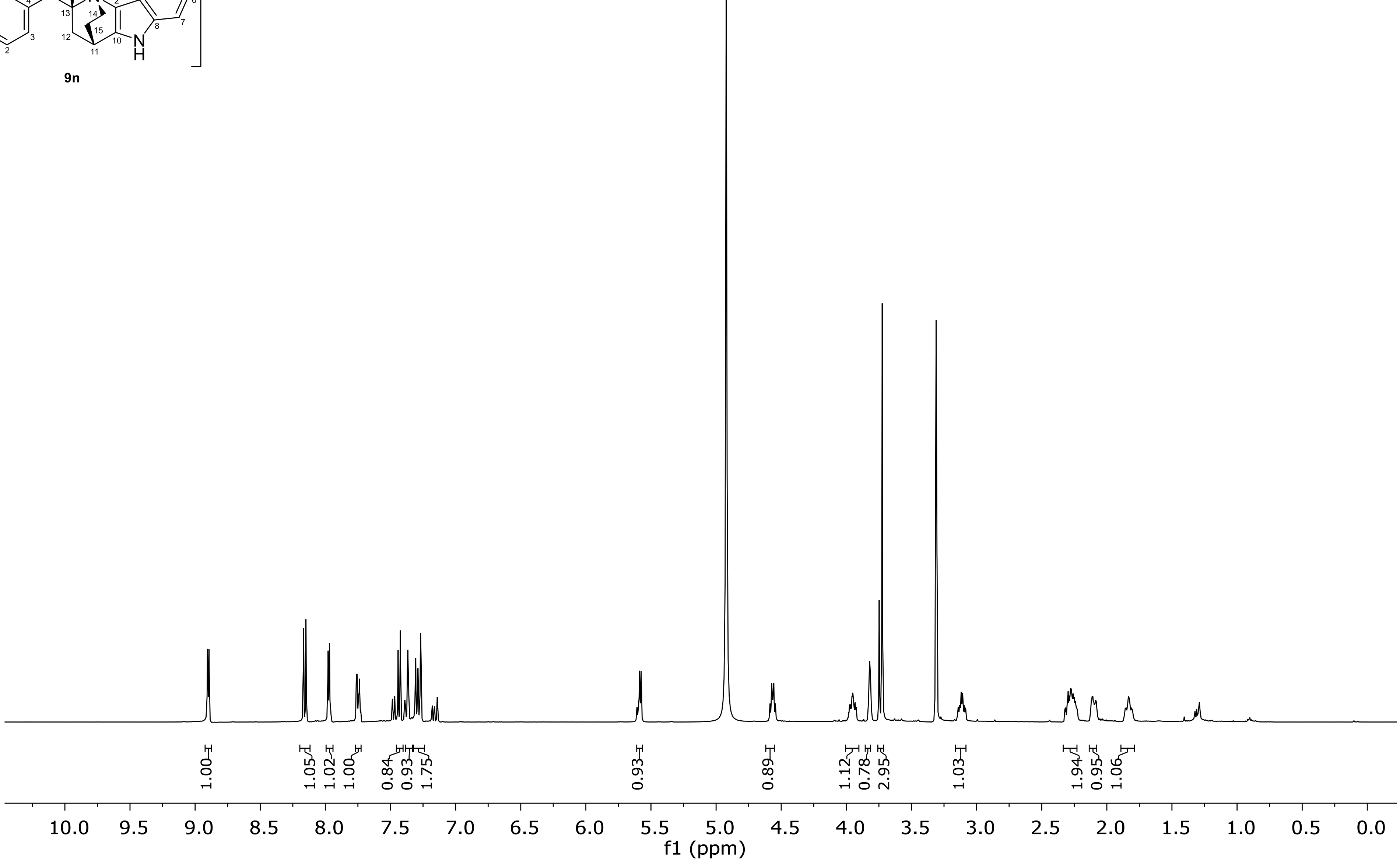
1.00
1.05
1.02
1.00
0.84
0.93
1.75
0.93
0.89
1.12
0.78
2.95
1.03
1.94
0.95
1.06
10.0 9.5 9.0 8.5 8.0 7.5 7.0 6.5 6.0 5.5 5.0 4.5 4.0 3.5 3.0 2.5 2.0 1.5 1.0 0.5 0.0
f1 (ppm)

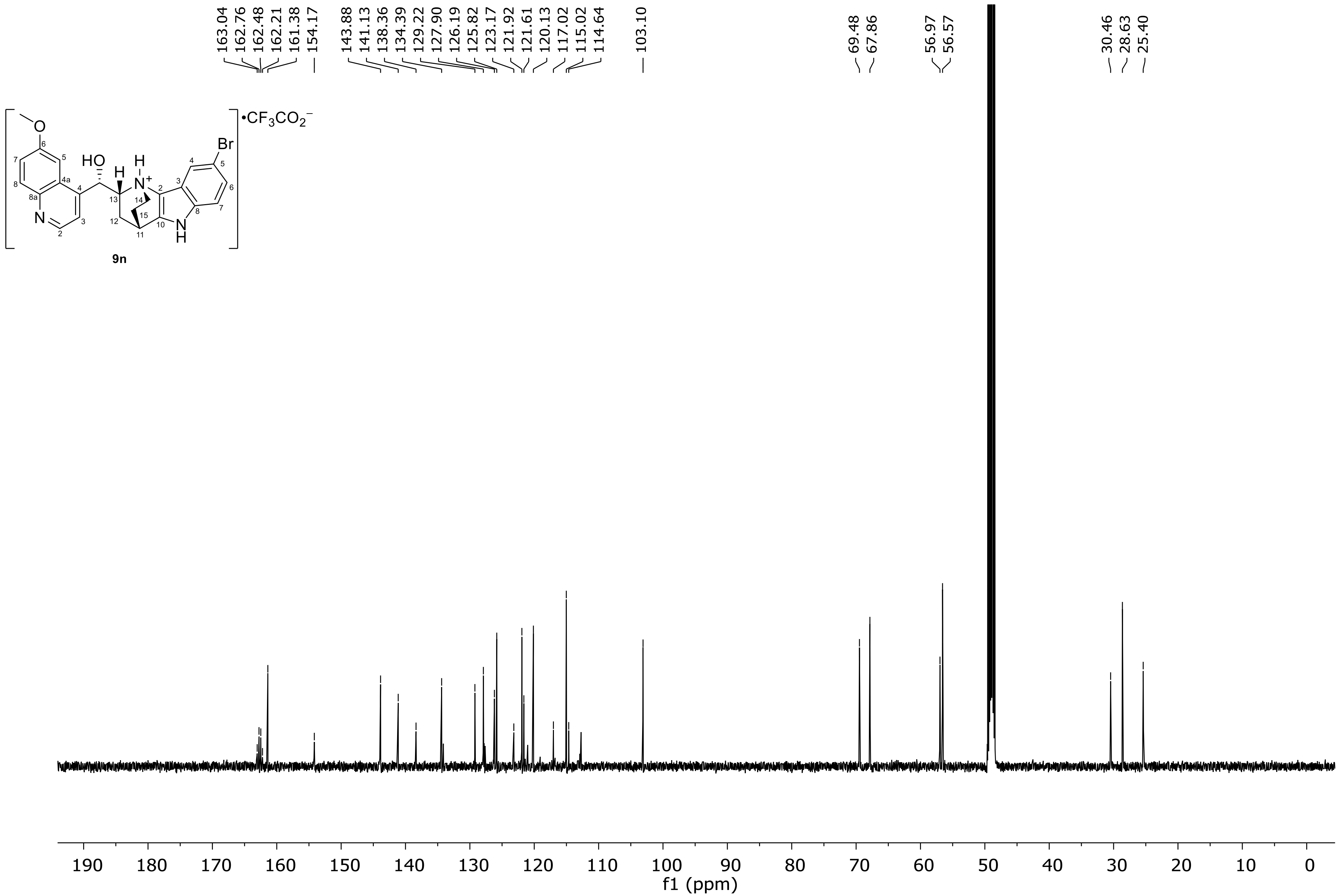
163.04
162.76
162.48
162.21
161.38
154.17
143.88
141.13
138.36
134.39
129.22
127.90
126.19
125.82
123.17
121.92
121.61
120.13
117.02
115.02
114.64
103.10
69.48
67.86
56.97
56.57
30.46
28.63
25.40
•$CF_3CO_2^-$
HO
H
N+
Br
NH
N
9n
190 180 170 160 150 140 130 120 110 100 90 80 70 60 50 40 30 20 10 0
f1 (ppm)

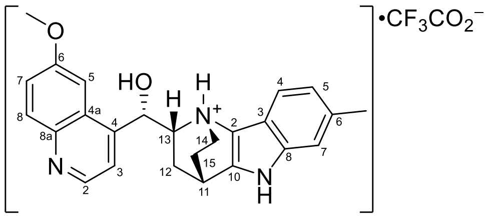
•CF3CO2−
HO
H
N+
H
N
H
N
O
2
3
4
4a
5
6
7
8
8a
13
12
11
14
15
10
8
7
6
5
4
3
2

9o

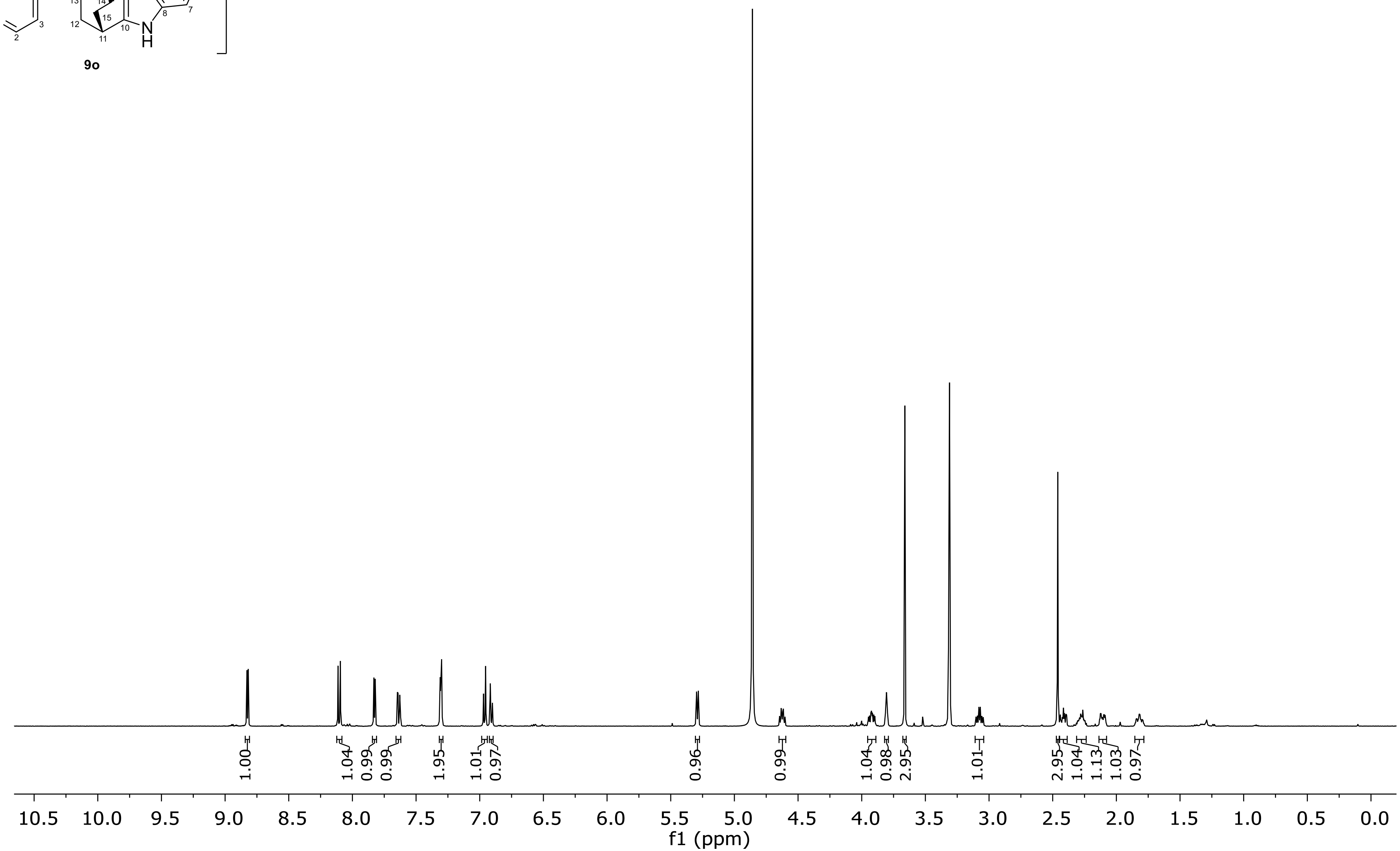
1.00
1.04
0.99
0.99
1.95
1.01
0.97
0.96
0.99
1.04
0.98
2.95
1.01
2.95
1.04
1.13
1.03
0.97
10.5 10.0 9.5 9.0 8.5 8.0 7.5 7.0 6.5 6.0 5.5 5.0 4.5 4.0 3.5 3.0 2.5 2.0 1.5 1.0 0.5 0.0
f1 (ppm)

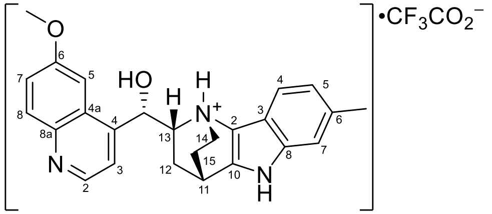
•CF3CO2−
HO
H
N+
NH

9o

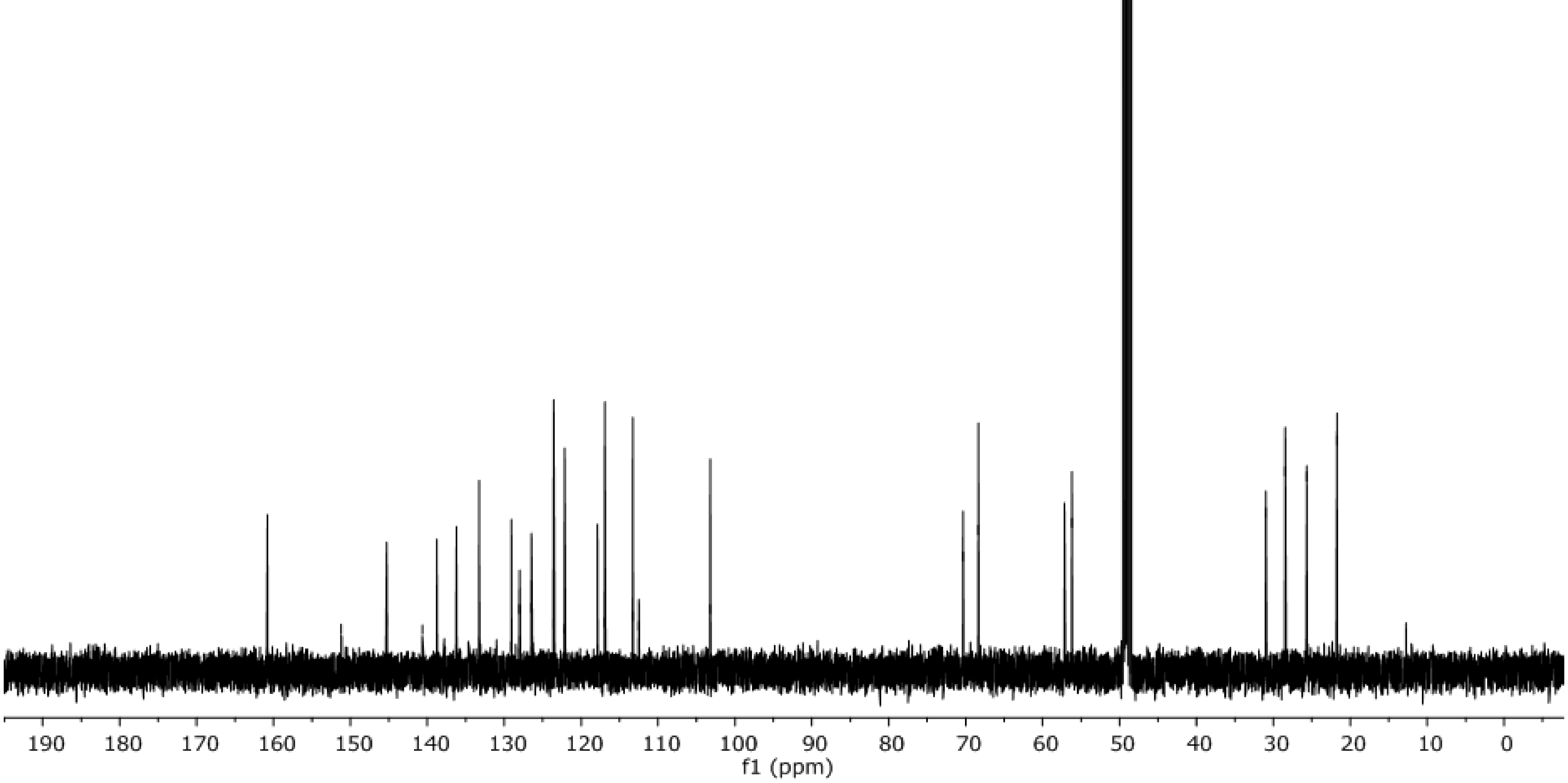
160.81
151.17
145.30
140.63
138.79
136.21
133.26
129.07
128.02
126.48
123.59
122.15
117.87
116.91
113.27
112.51
103.23
70.37
68.36
57.15
56.17
49.51
30.98
28.47
25.63
21.70
190 180 170 160 150 140 130 120 110 100 90 80 70 60 50 40 30 20 10 0
f1 (ppm)

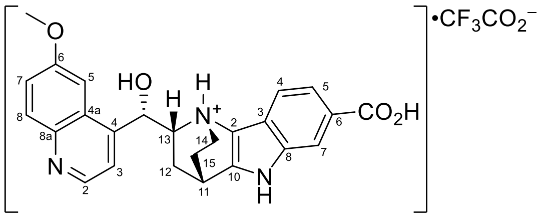
•CF3CO2−
HO
H
N+
CO2H
N
H

9p

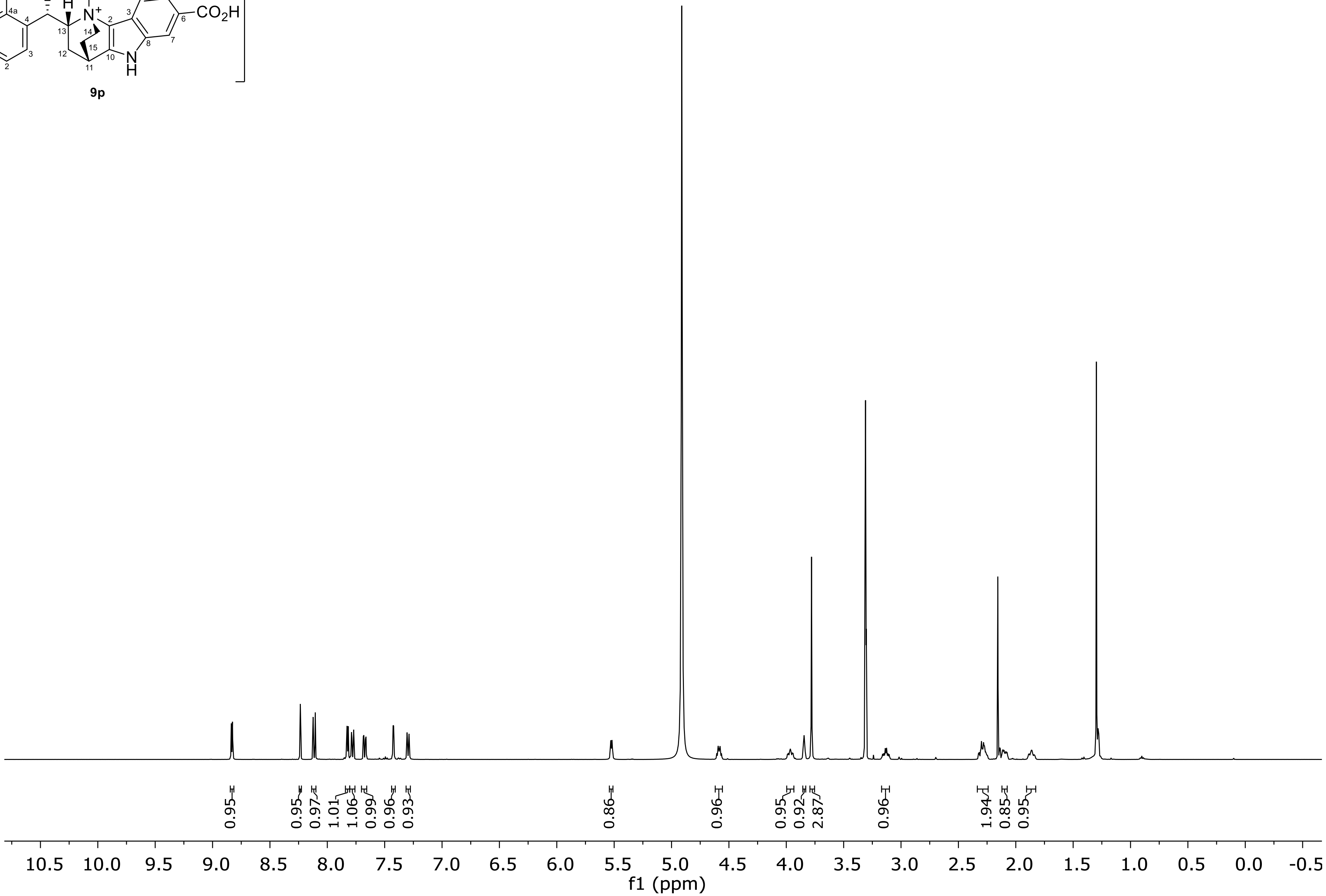
0.95
0.95
0.97
1.01
1.06
0.99
0.96
0.93
0.86
0.96
0.95
0.92
2.87
0.96
1.94
0.85
0.95
10.5 10.0 9.5 9.0 8.5 8.0 7.5 7.0 6.5 6.0 5.5 5.0 4.5 4.0 3.5 3.0 2.5 2.0 1.5 1.0 0.5 0.0 -0.5
f1 (ppm)

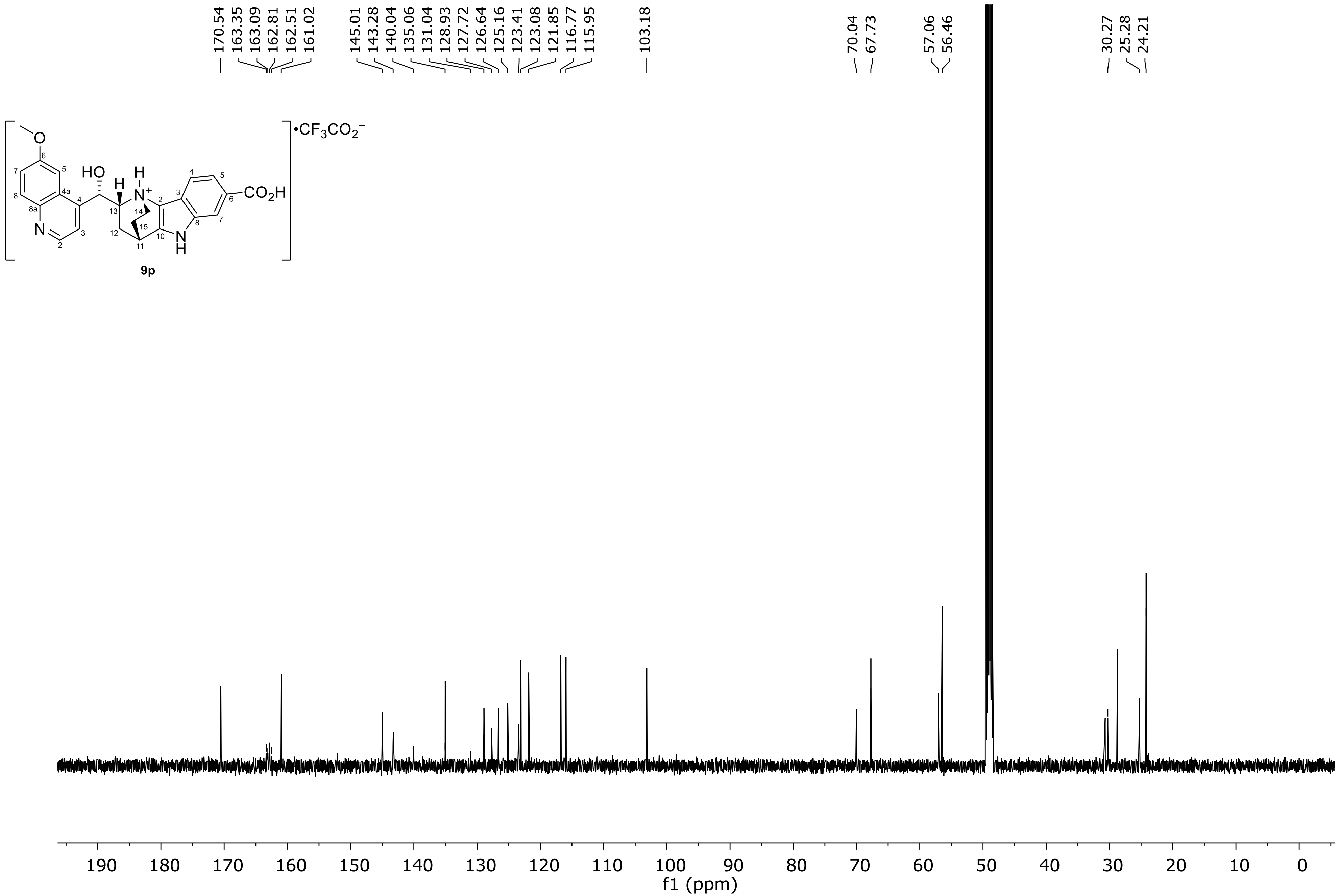
170.54
163.35
163.09
162.81
162.51
161.02
145.01
143.28
140.04
135.06
131.04
128.93
127.72
126.64
125.16
123.41
123.08
121.85
116.77
115.95
103.18
70.04
67.73
57.06
56.46
30.27
25.28
24.21
•CF3CO2−
CO2H
HO
9p
f1 (ppm)

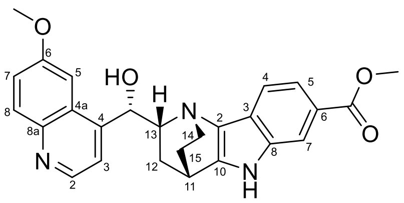

9q

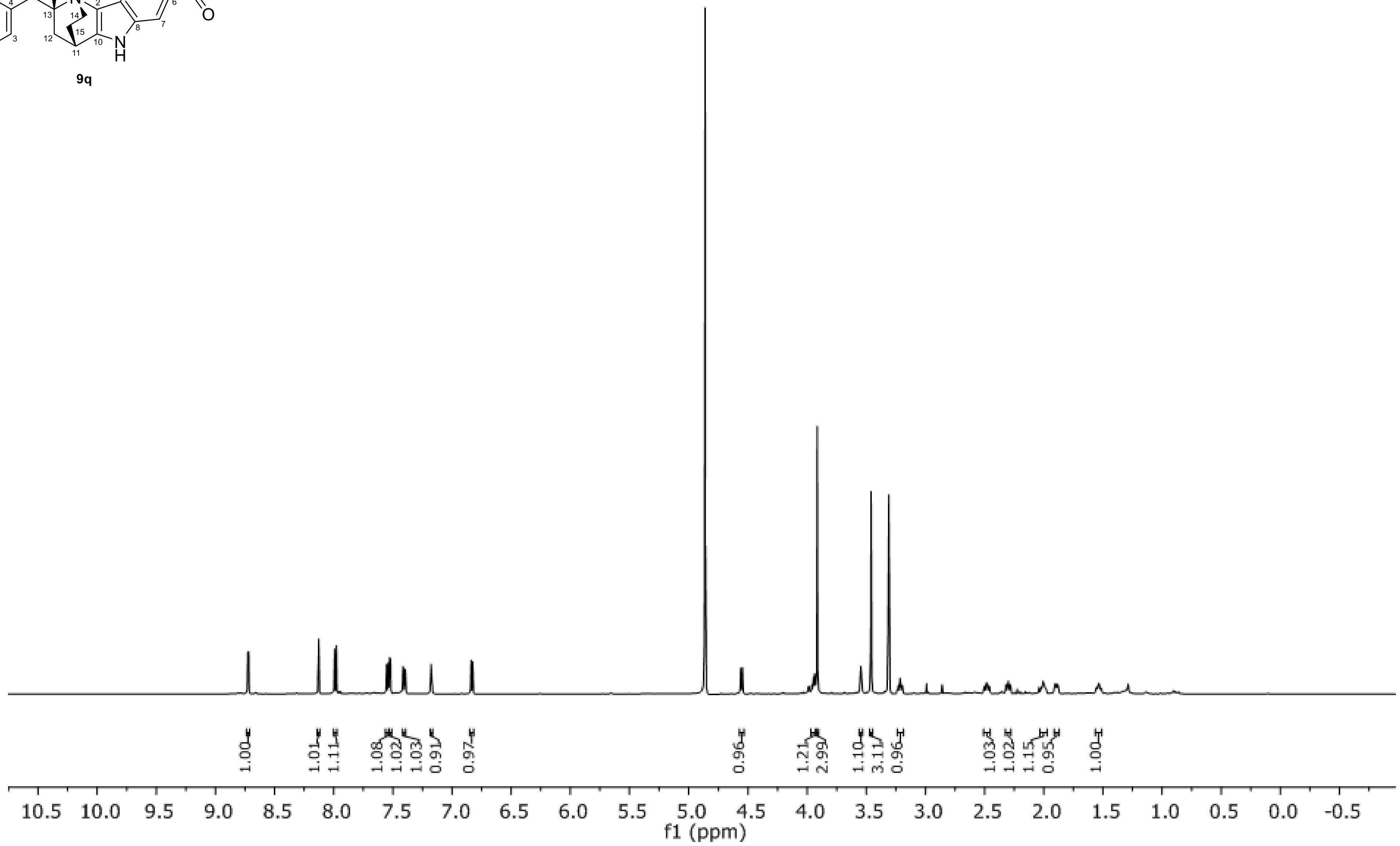
1.00
1.01
1.11
1.08
1.02
1.03
0.91
0.97
0.96
1.21
2.99
1.10
3.11
0.96
1.03
1.02
1.15
0.95
1.00
10.5 10.0 9.5 9.0 8.5 8.0 7.5 7.0 6.5 6.0 5.5 5.0 4.5 4.0 3.5 3.0 2.5 2.0 1.5 1.0 0.5 0.0 -0.5
f1 (ppm)

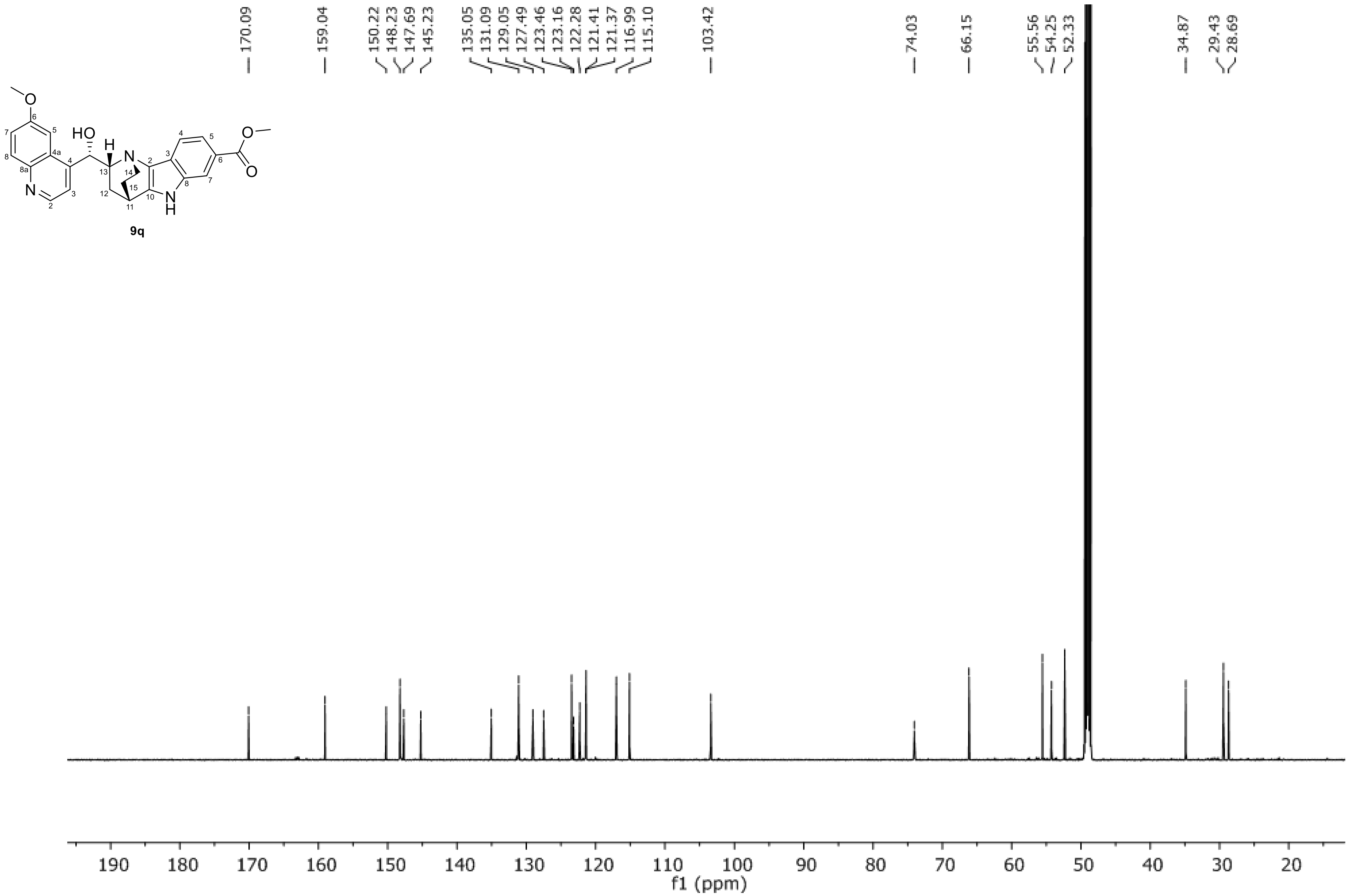
HO
H
N
NH
O
1, 2, 3, 4, 4a, 5, 6, 7, 8, 8a, 10, 11, 12, 13, 14, 15
9q
170.09
159.04
150.22
148.23
147.69
145.23
135.05
131.09
129.05
127.49
123.46
123.16
122.28
121.41
121.37
116.99
115.10
103.42
74.03
66.15
55.56
54.25
52.33
34.87
29.43
28.69
f1 (ppm)

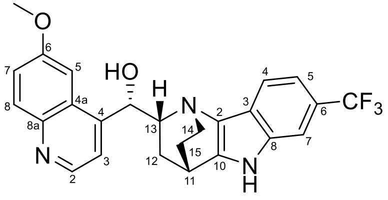

9r

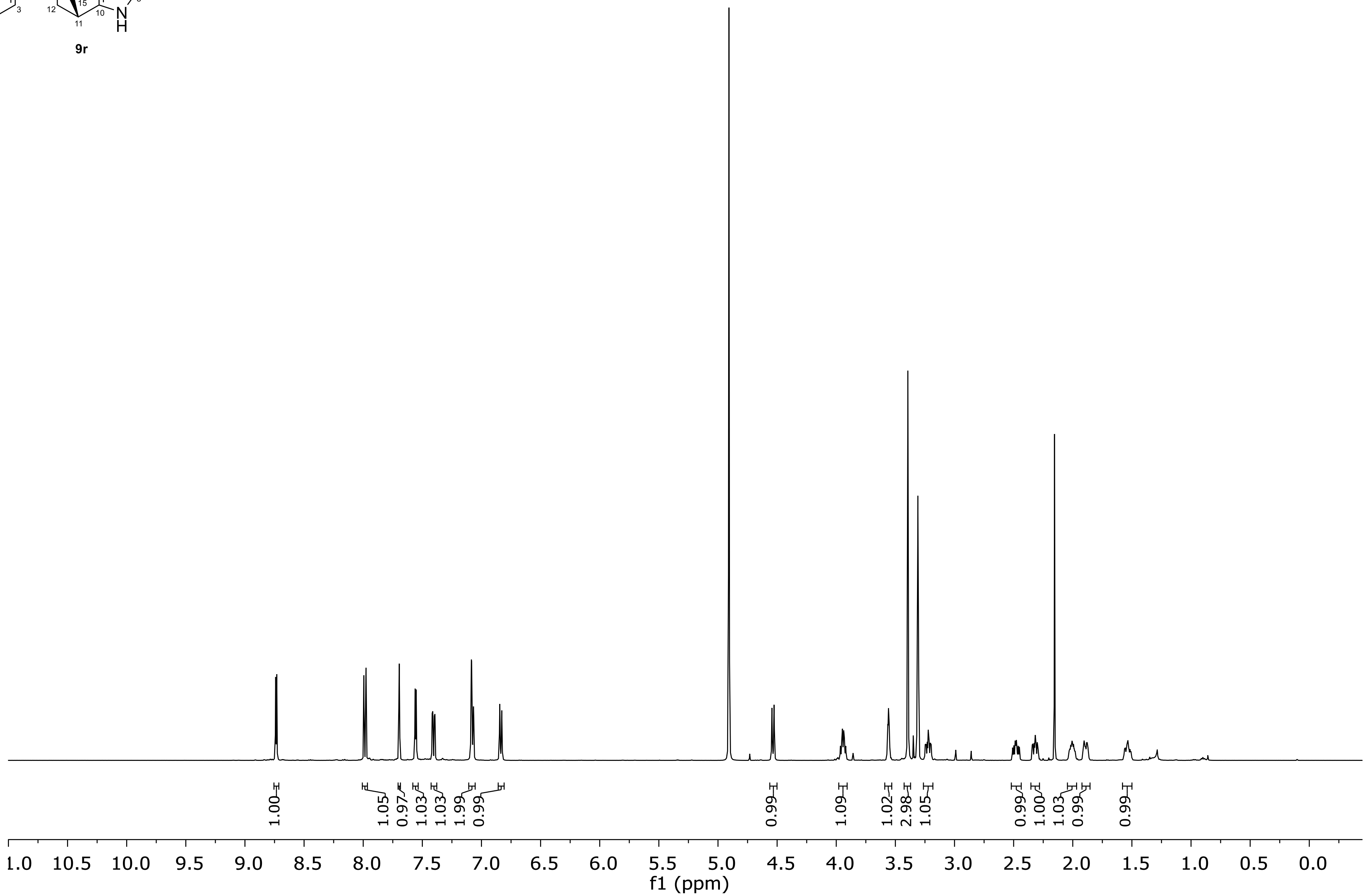
1.00
1.05
0.97
1.03
1.03
1.99
0.99
0.99
1.09
1.02
2.98
1.05
0.99
1.00
1.03
0.99
0.99
1.0 10.5 10.0 9.5 9.0 8.5 8.0 7.5 7.0 6.5 6.0 5.5 5.0 4.5 4.0 3.5 3.0 2.5 2.0 1.5 1.0 0.5 0.0
f1 (ppm)

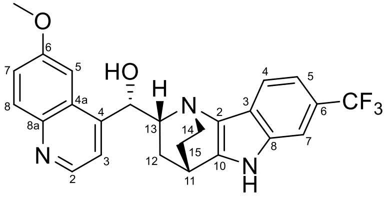

9r

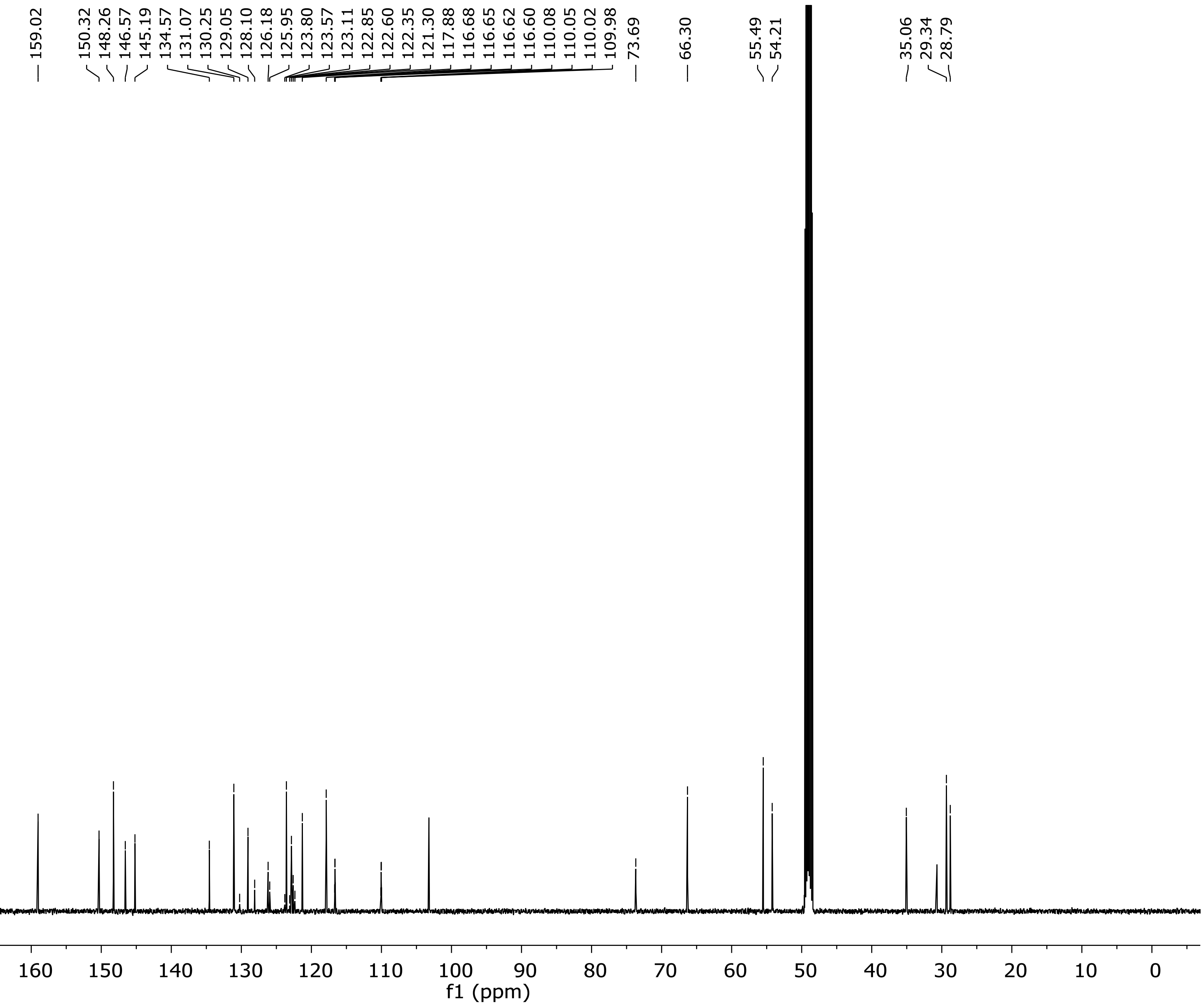

159.02
150.32
148.26
146.57
145.19
134.57
131.07
130.25
129.05
128.10
126.18
125.95
123.80
123.57
123.11
122.85
122.60
122.35
121.30
117.88
116.68
116.65
116.62
116.60
110.08
110.05
110.02
109.98
73.69
66.30
55.49
54.21
35.06
29.34
28.79
f1 (ppm)

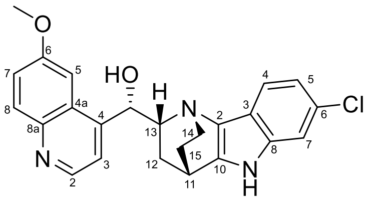

HO
H
N
N
H
Cl
O

9s

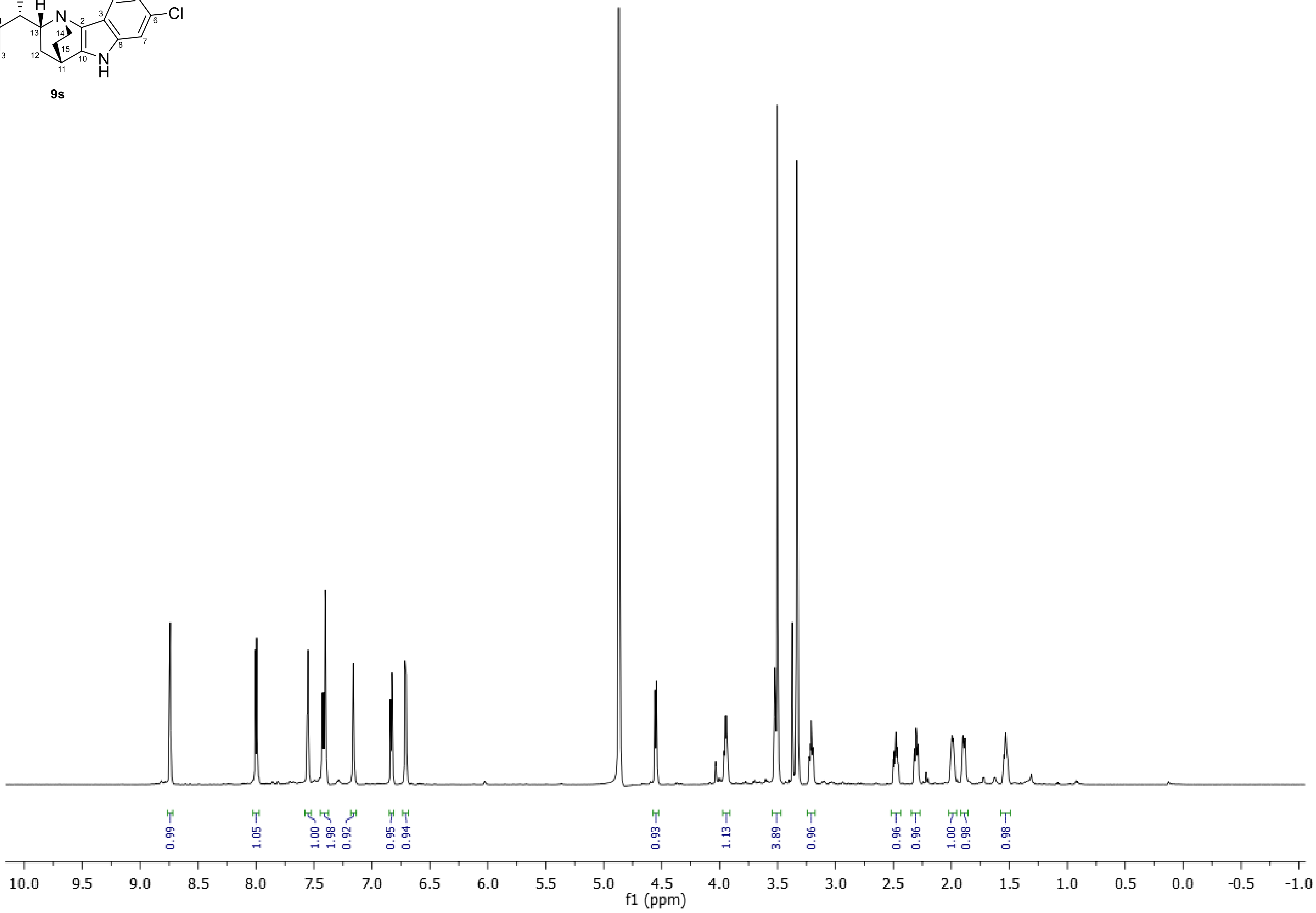

0.99
1.05
1.00
1.98
0.92
0.95
0.94
0.93
1.13
3.89
0.96
0.96
0.96
1.00
0.98
0.98
10.0 9.5 9.0 8.5 8.0 7.5 7.0 6.5 6.0 5.5 5.0 4.5 4.0 3.5 3.0 2.5 2.0 1.5 1.0 0.5 0.0 -0.5 -1.0
f1 (ppm)

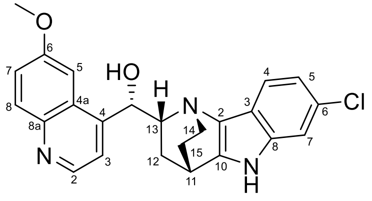
HO
H
N
NH
Cl
O
N
9s

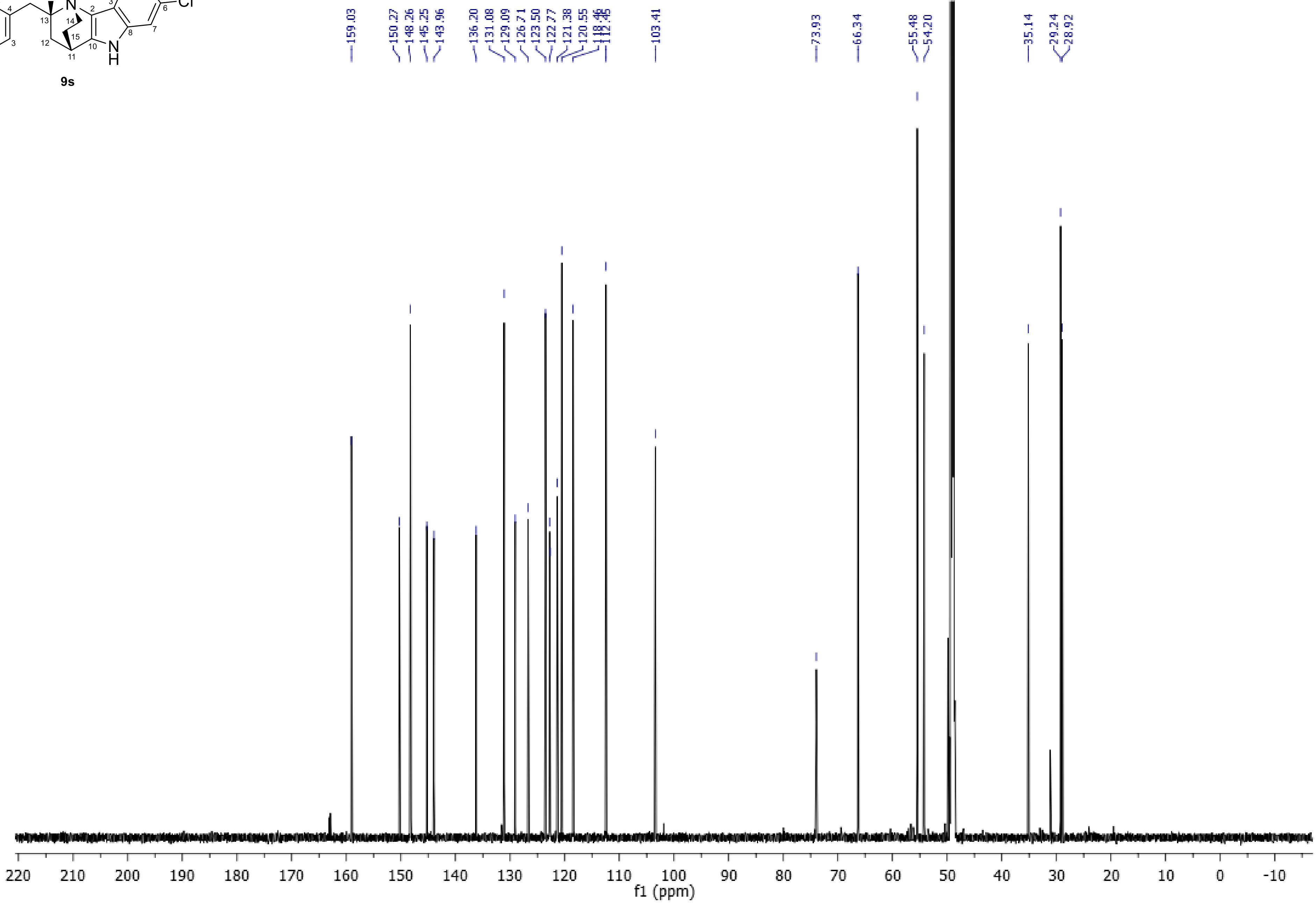
159.03
150.27
148.26
145.25
143.96
136.20
131.08
129.09
126.71
123.50
122.77
121.38
120.55
112.45
103.41
73.93
66.34
55.48
54.20
35.14
29.24
28.92
220 210 200 190 180 170 160 150 140 130 120 110 100 90 80 70 60 50 40 30 20 10 0 -10
f1 (ppm)

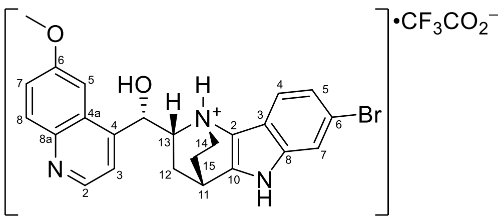
•CF3CO2−
HO
Br

9t

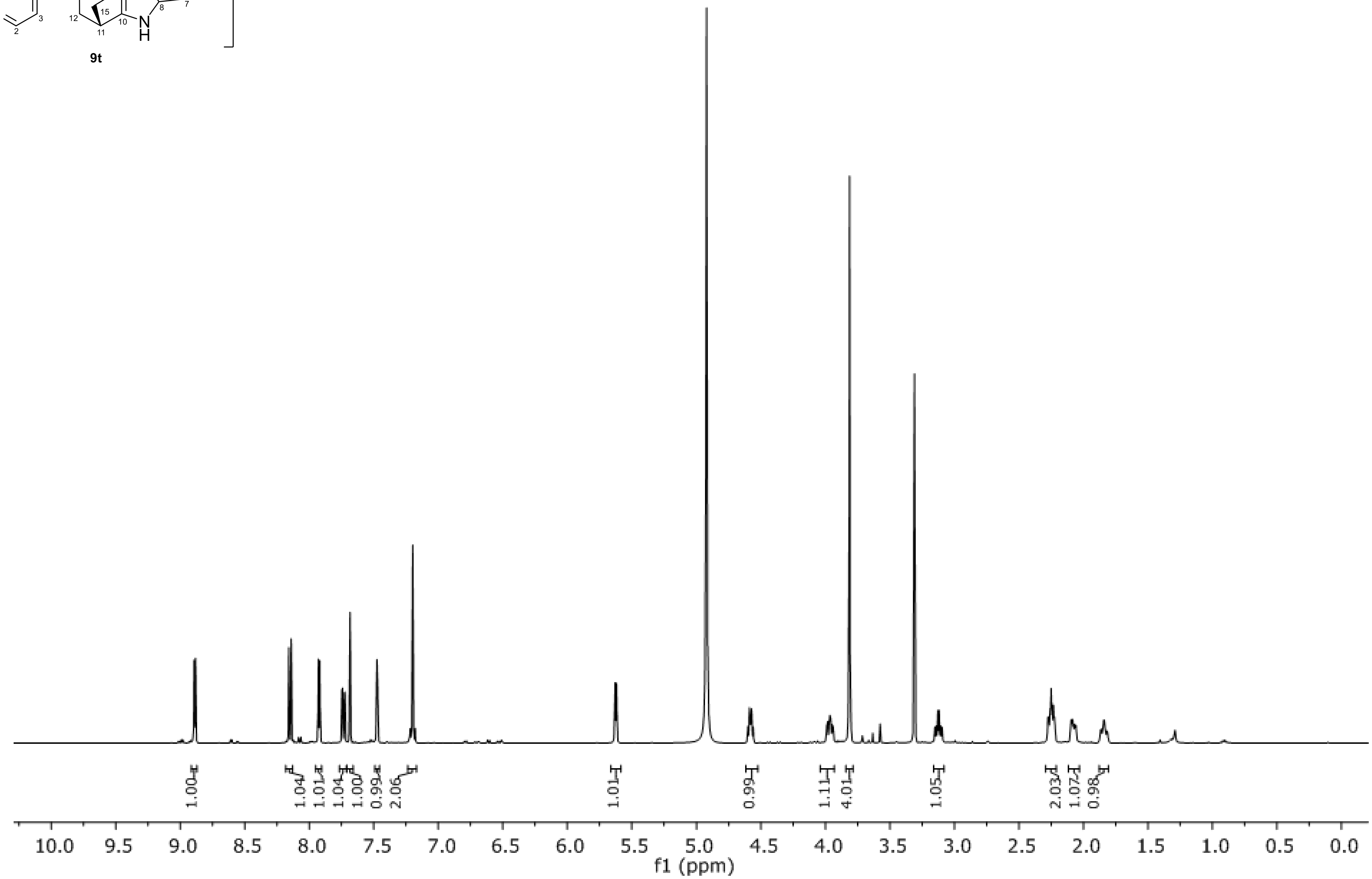
1.00
1.04
1.01
1.04
1.00
0.99
2.06
1.01
0.99
1.11
4.01
1.05
2.03
1.07
0.98
10.0 9.5 9.0 8.5 8.0 7.5 7.0 6.5 6.0 5.5 5.0 4.5 4.0 3.5 3.0 2.5 2.0 1.5 1.0 0.5 0.0
f1 (ppm)

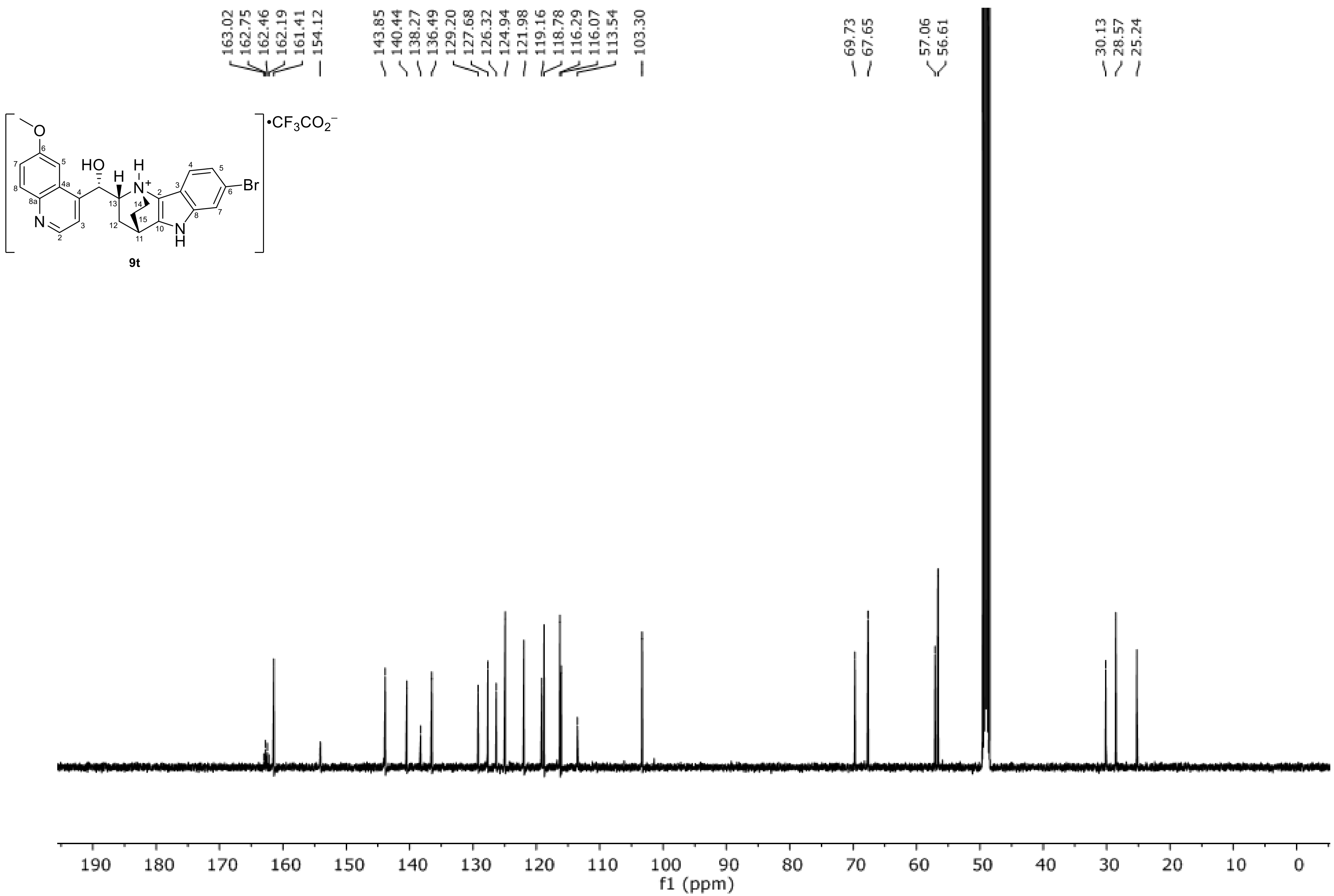
163.02
162.75
162.46
162.19
161.41
154.12
143.85
140.44
138.27
136.49
129.20
127.68
126.32
124.94
121.98
119.16
118.78
116.29
116.07
113.54
103.30
69.73
67.65
57.06
56.61
30.13
28.57
25.24
•CF3CO2−
HO
H
N+
Br
NH
N
f1 (ppm)

9t

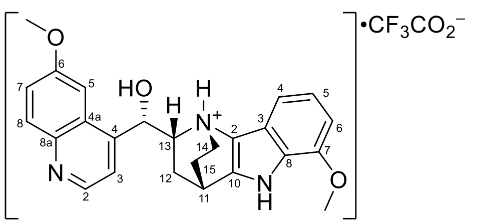
•CF3CO2−
HO
H
H
N+
N
H
O
O
N
2
3
4
4a
5
6
7
8
8a
12
13
14
15
11
10
8
7
6
5
4
3
2

9u

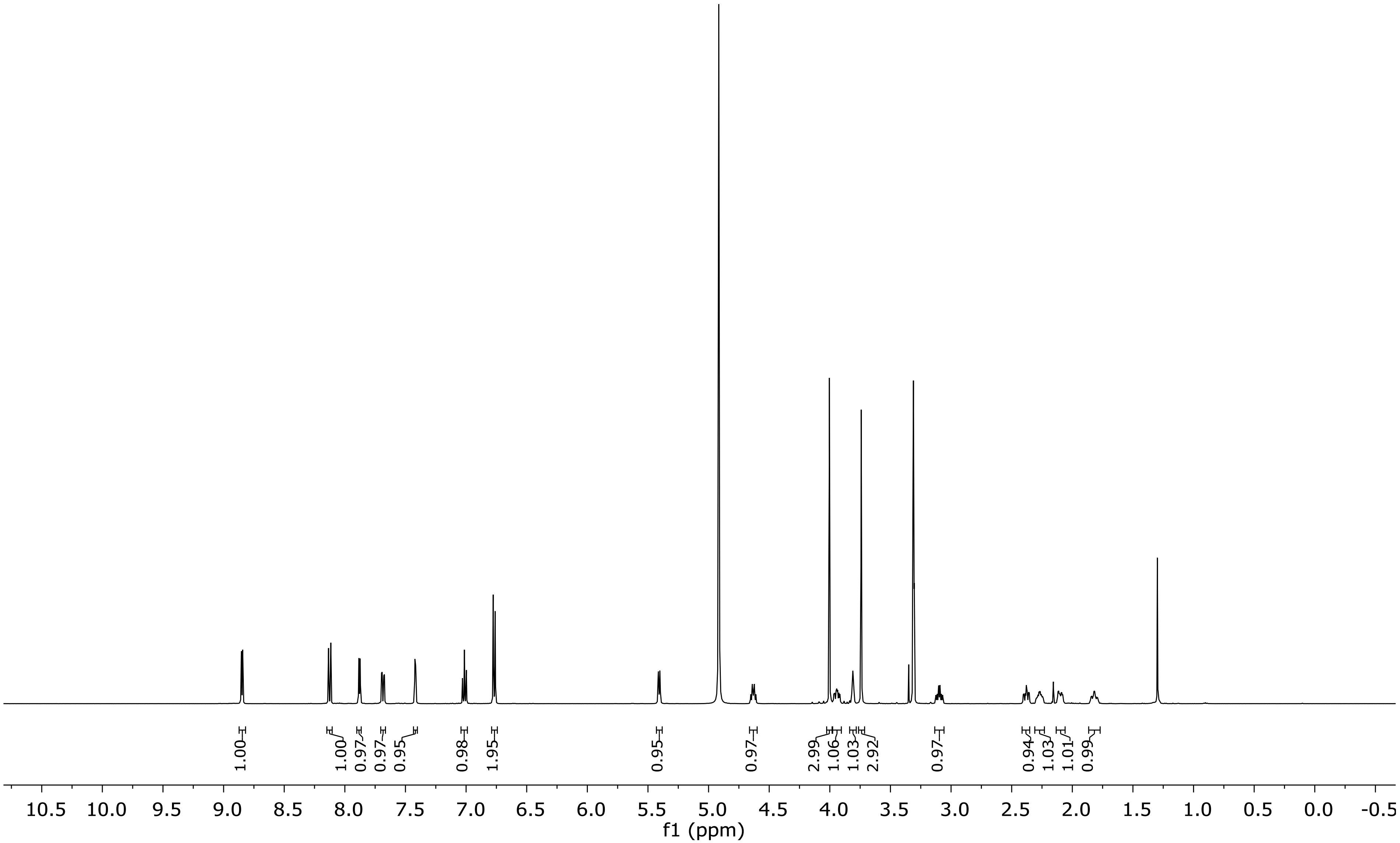
1.00
1.00
0.97
0.97
0.95
0.98
1.95
0.95
0.97
2.99
1.06
1.03
2.92
0.97
0.94
1.03
1.01
0.99
10.5
10.0
9.5
9.0
8.5
8.0
7.5
7.0
6.5
6.0
5.5
5.0
4.5
4.0
3.5
3.0
2.5
2.0
1.5
1.0
0.5
0.0
-0.5
f1 (ppm)

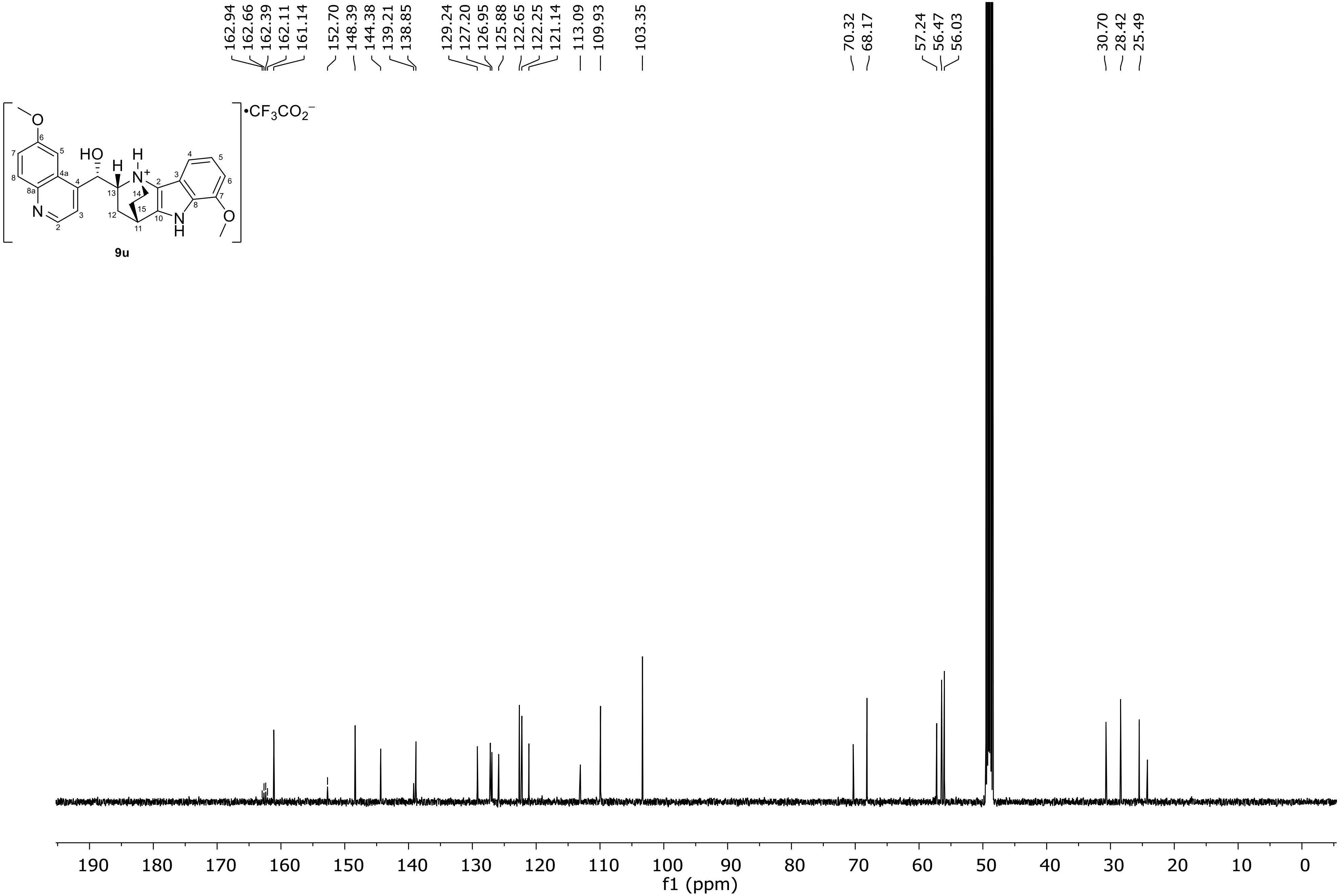

162.94
162.66
162.39
162.11
161.14
152.70
148.39
144.38
139.21
138.85
129.24
127.20
126.95
125.88
122.65
122.25
121.14
113.09
109.93
103.35
70.32
68.17
57.24
56.47
56.03
30.70
28.42
25.49
•CF3CO2−
190 180 170 160 150 140 130 120 110 100 90 80 70 60 50 40 30 20 10 0
f1 (ppm)

9u

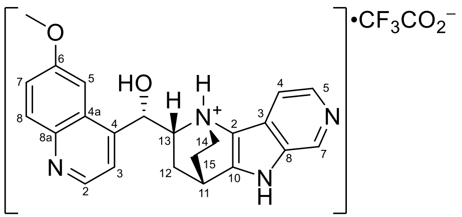

9v

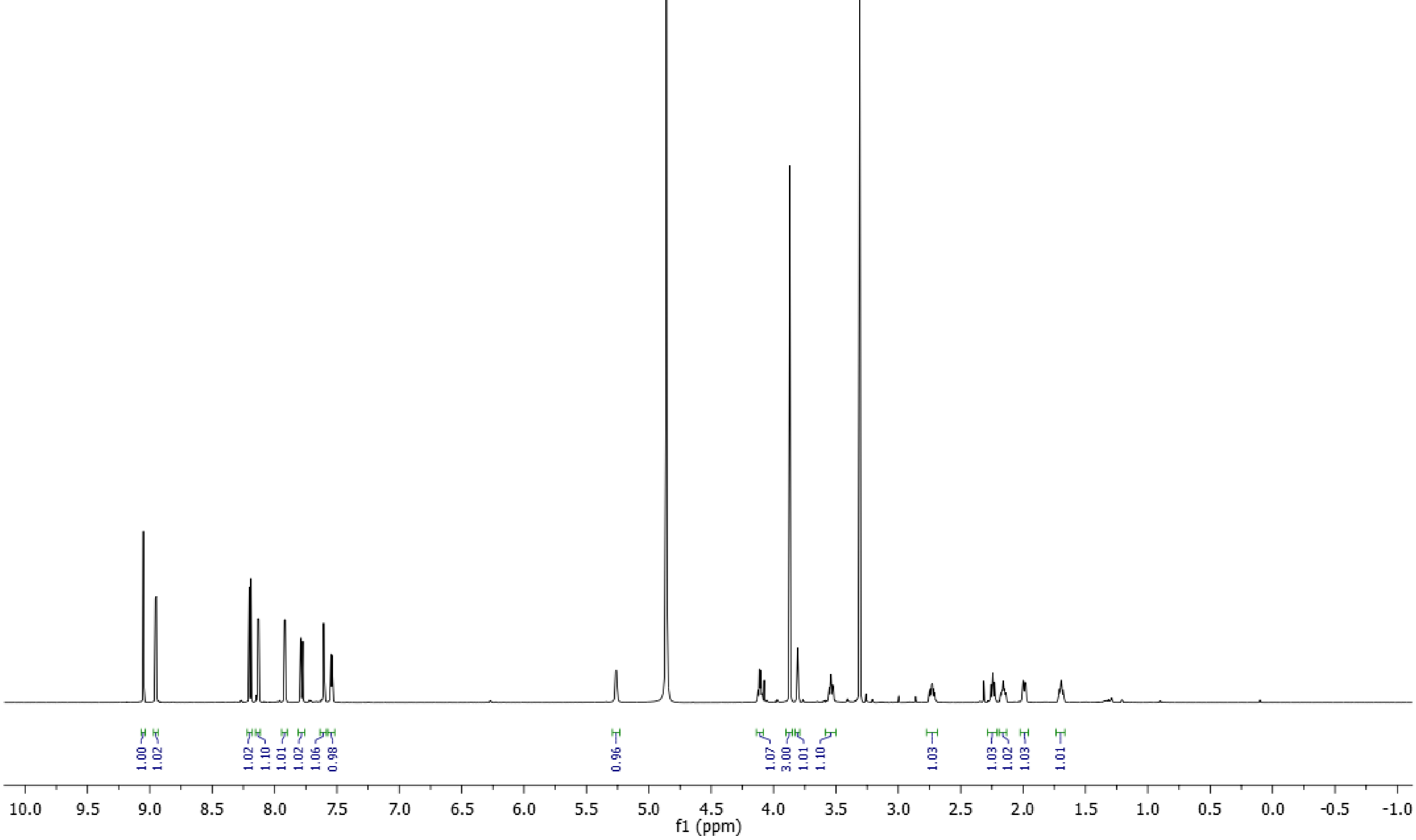
1.00
1.02
1.02
1.10
1.01
1.02
1.06
0.98
0.96
1.07
3.00
1.01
1.10
1.03
1.03
1.02
1.03
1.01
10.0 9.5 9.0 8.5 8.0 7.5 7.0 6.5 6.0 5.5 5.0 4.5 4.0 3.5 3.0 2.5 2.0 1.5 1.0 0.5 0.0 -0.5 -1.0
f1 (ppm)

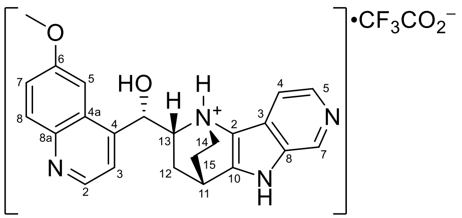
•CF3CO2–
HO
H
H
N+
N
N
H
O
N

9v

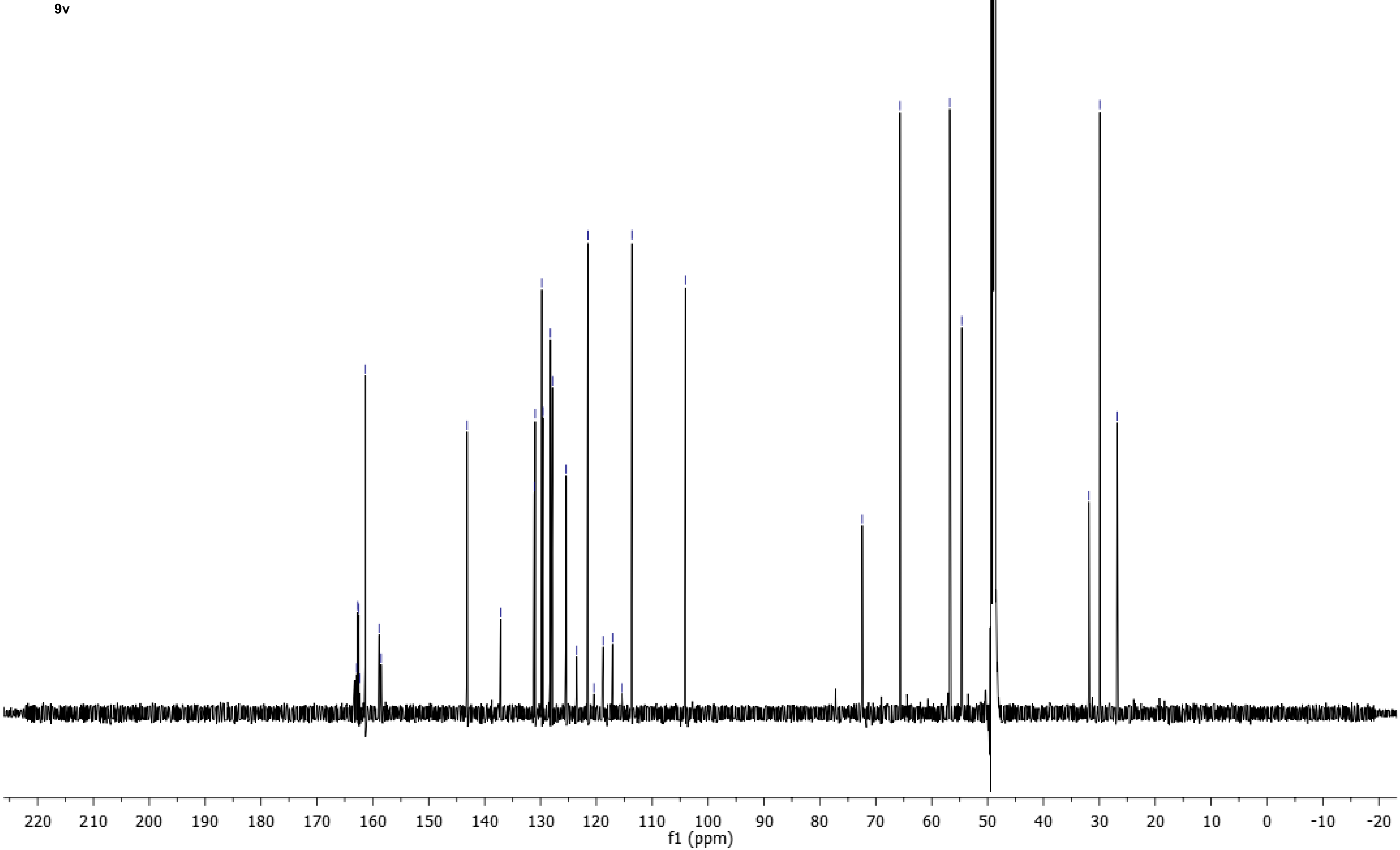
162.91
162.71
162.50
162.30
161.35
158.88
158.52
143.13
137.14
131.10
130.97
129.76
129.54
128.21
127.86
125.43
121.50
117.11
113.62
104.05
72.44
65.69
56.78
54.64
31.89
29.93
26.80
f1 (ppm)

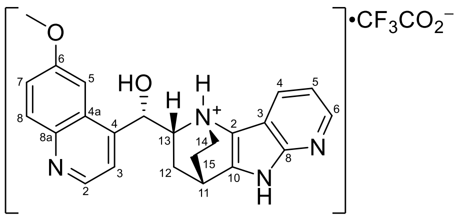

9w

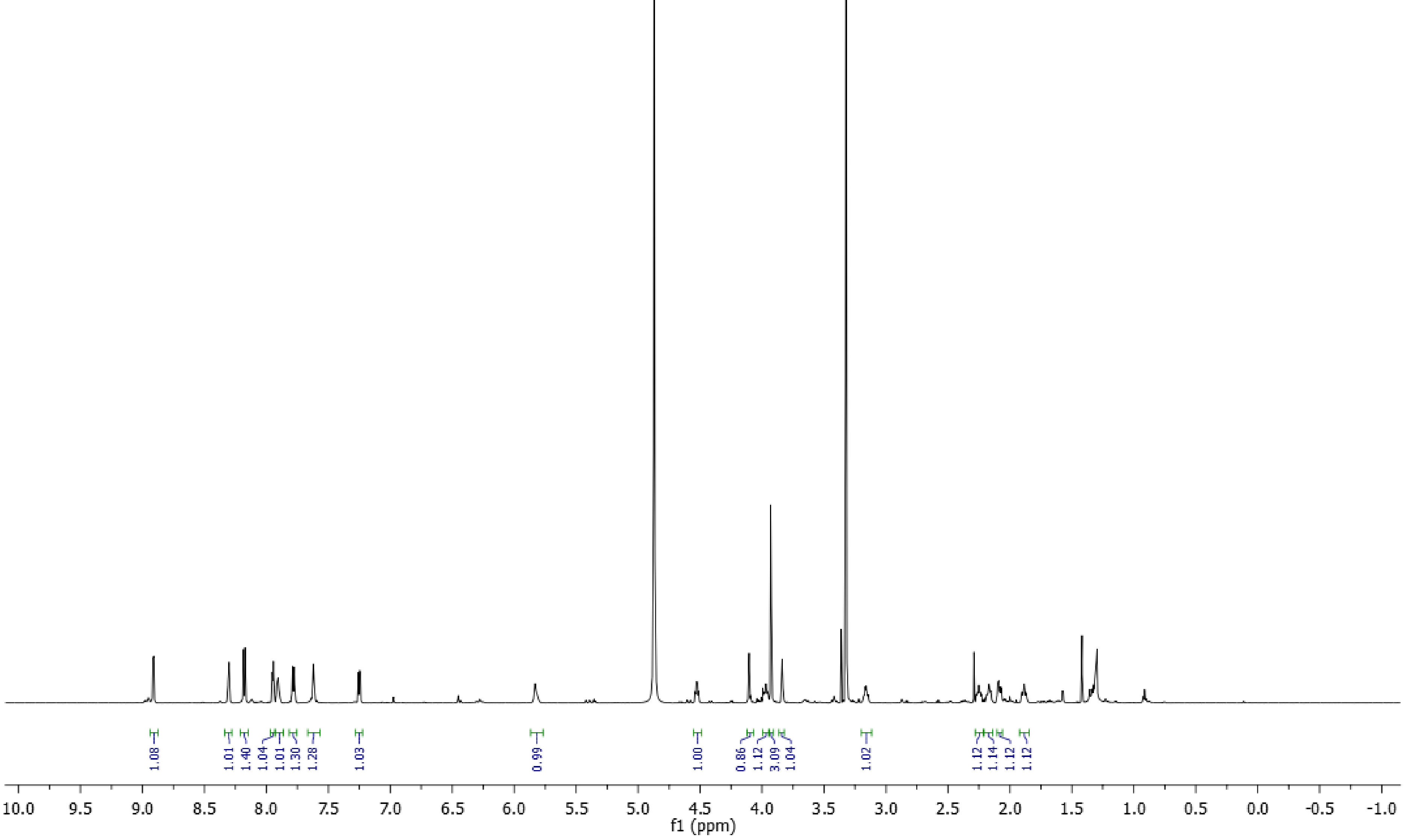

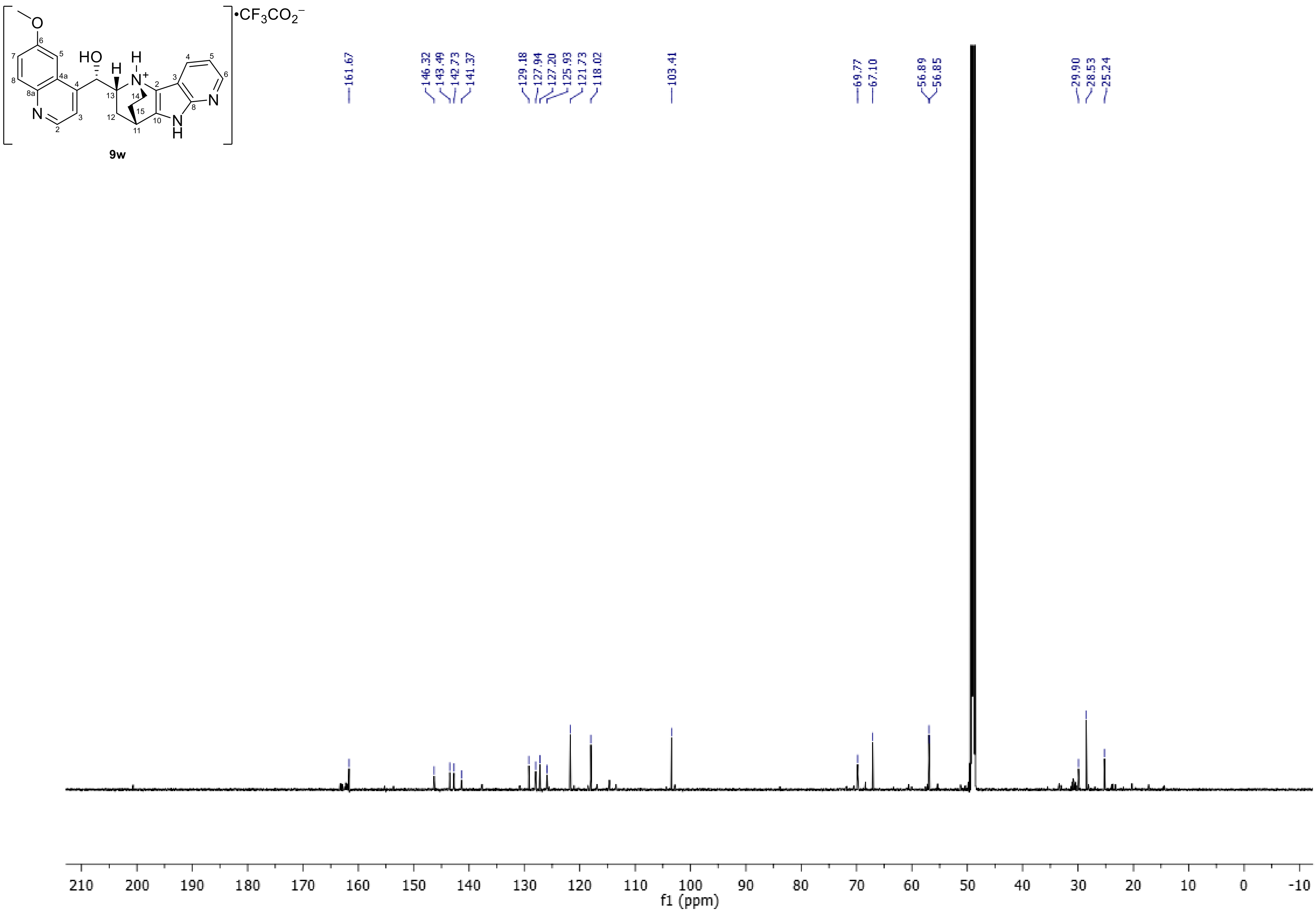
•$CF_3CO_2^-$
HO
H
N+
N
N
NH
O
9w
161.67
146.32
143.49
142.73
141.37
129.18
127.94
127.20
125.93
121.73
118.02
103.41
69.77
67.10
56.89
56.85
29.90
28.53
25.24
f1 (ppm)

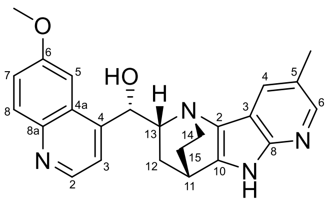

HO
H
N
N
NH

9w-b

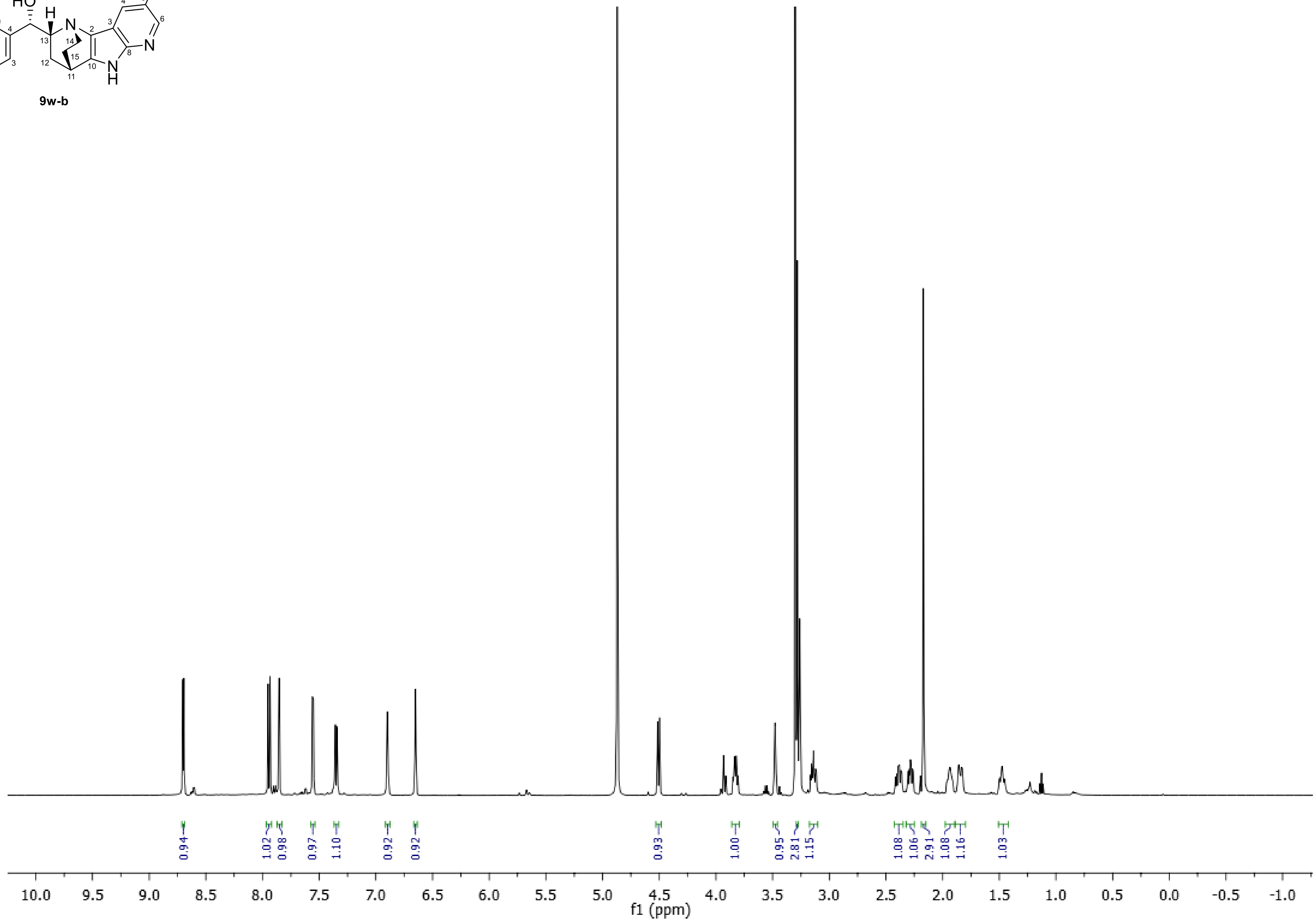

0.94
1.02
0.98
0.97
1.10
0.92
0.92
0.93
1.00
0.95
2.81
1.15
1.08
1.06
2.91
1.08
1.16
1.03
10.0 9.5 9.0 8.5 8.0 7.5 7.0 6.5 6.0 5.5 5.0 4.5 4.0 3.5 3.0 2.5 2.0 1.5 1.0 0.5 0.0 -0.5 -1.0
f1 (ppm)

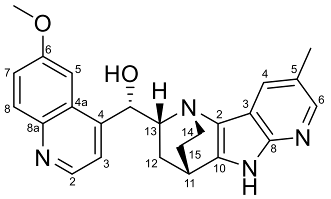

9w-b

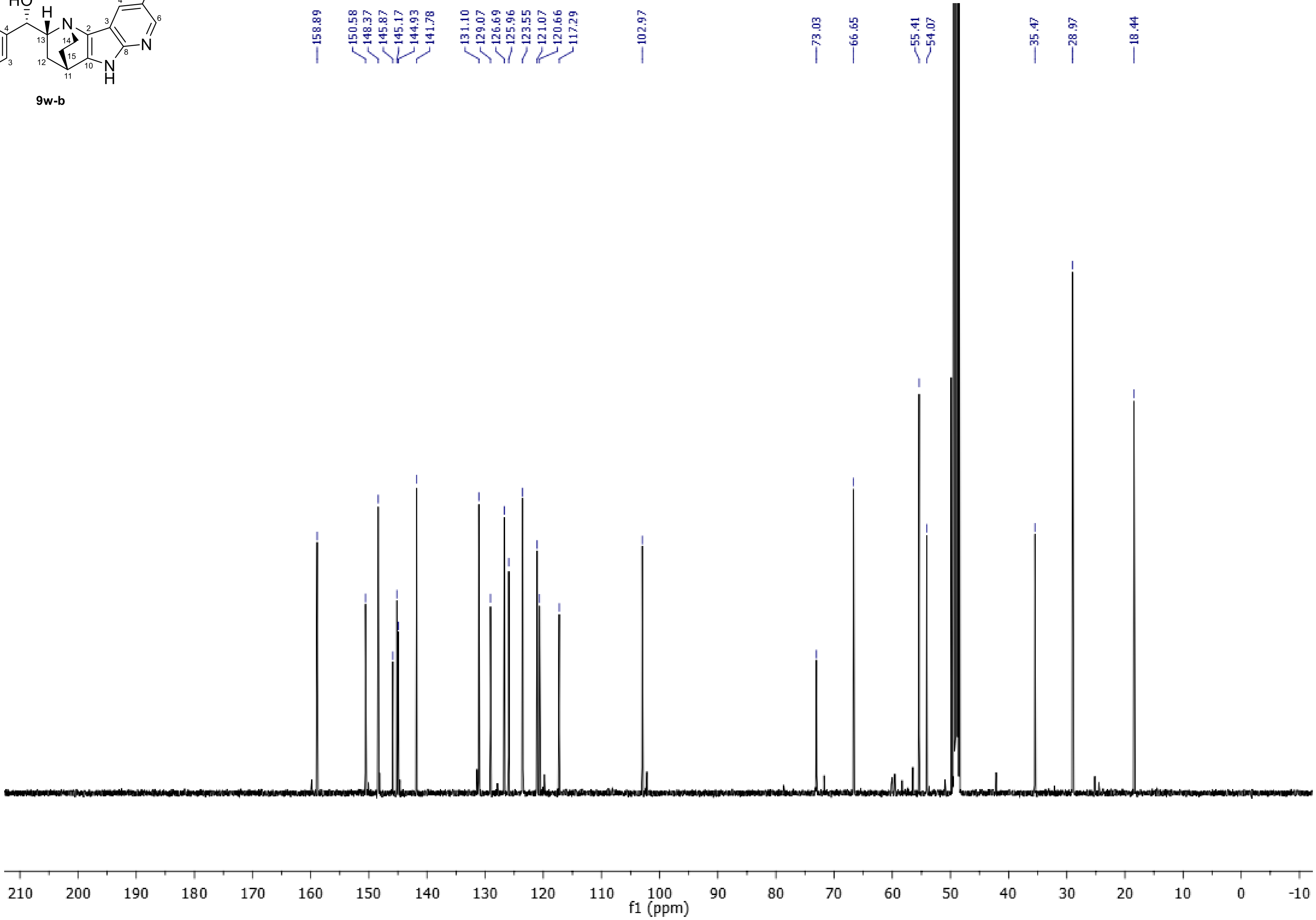
158.89
150.58
148.37
145.87
145.17
144.93
141.78
131.10
129.07
126.69
125.96
123.55
121.07
120.66
117.29
102.97
73.03
66.65
55.41
54.07
35.47
28.97
18.44
210 200 190 180 170 160 150 140 130 120 110 100 90 80 70 60 50 40 30 20 10 0 -10
f1 (ppm)

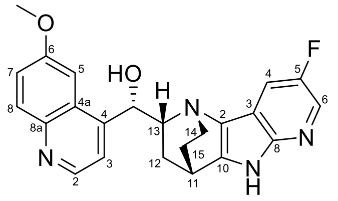

O
HO
H
N
F
N
N
H

9w-f

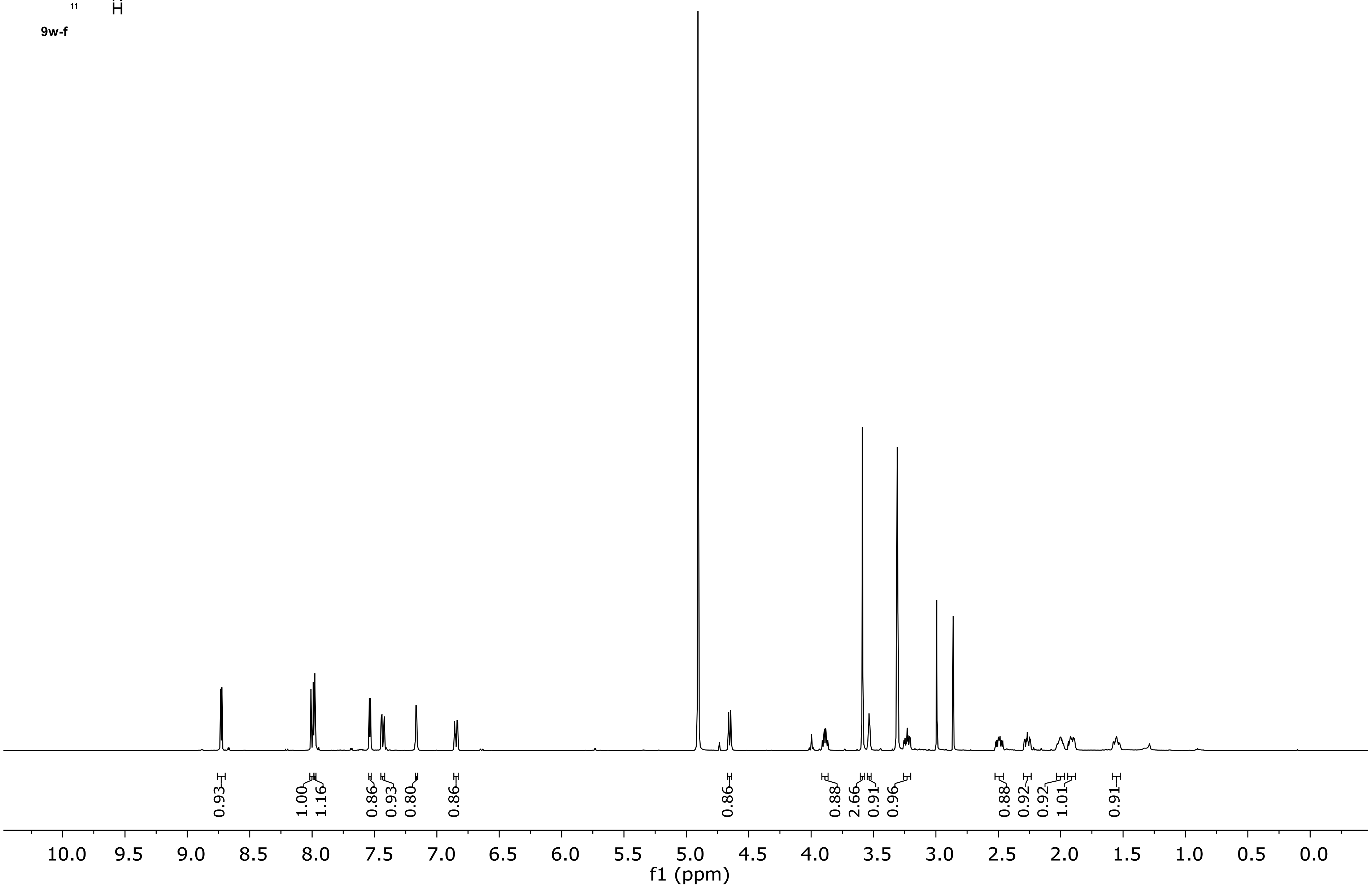

0.93
1.00
1.16
0.86
0.93
0.80
0.86
0.86
0.88
2.66
0.91
0.96
0.88
0.92
0.92
1.01
0.91
10.0 9.5 9.0 8.5 8.0 7.5 7.0 6.5 6.0 5.5 5.0 4.5 4.0 3.5 3.0 2.5 2.0 1.5 1.0 0.5 0.0
f1 (ppm)

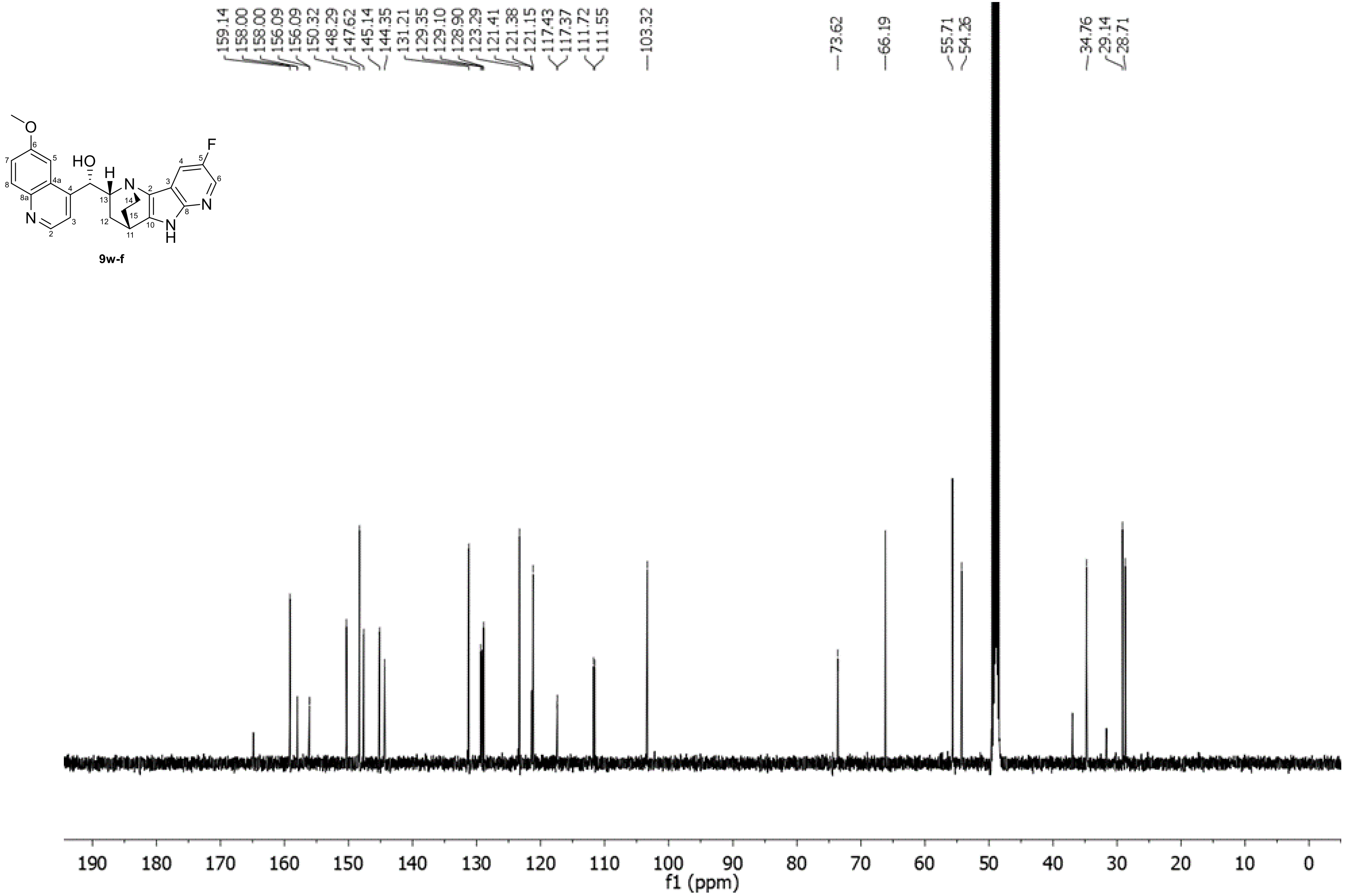
9w-f
159.14
158.00
158.00
156.09
156.09
150.32
148.29
147.62
145.14
144.35
131.21
129.35
129.10
128.90
123.29
121.41
121.38
121.15
117.43
117.37
111.72
111.55
103.32
73.62
66.19
55.71
54.26
34.76
29.14
28.71
190 180 170 160 150 140 130 120 110 100 90 80 70 60 50 40 30 20 10 0
f1 (ppm)

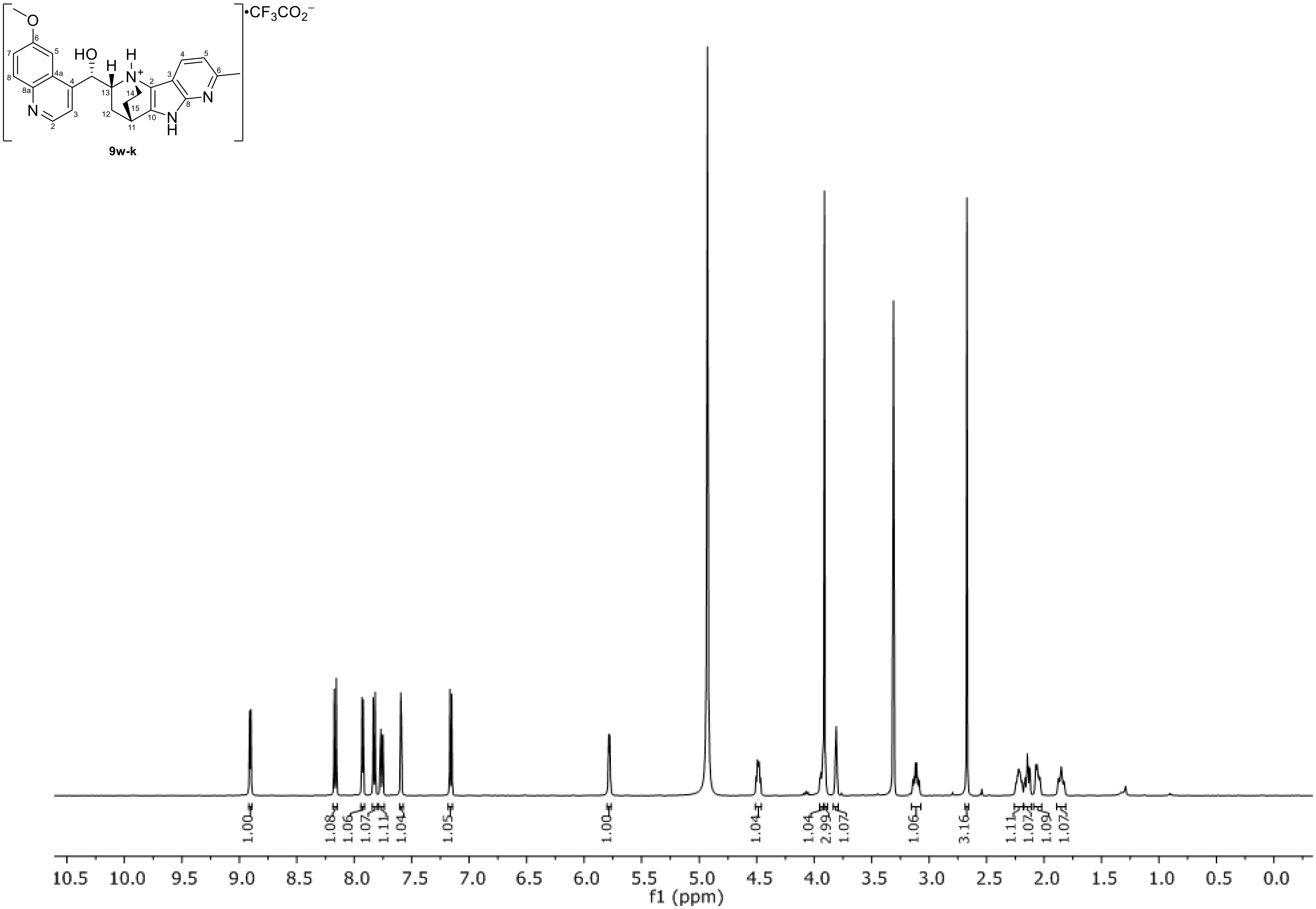
HO
H
N+
H
N
N
H
N
•CF3CO2−

9w-k

1.00
1.08
1.06
1.07
1.11
1.04
1.05
1.00
1.04
1.04
2.99
1.07
1.06
3.16
1.11
1.07
1.09
1.07
10.5 10.0 9.5 9.0 8.5 8.0 7.5 7.0 6.5 6.0 5.5 5.0 4.5 4.0 3.5 3.0 2.5 2.0 1.5 1.0 0.5 0.0
f1 (ppm)

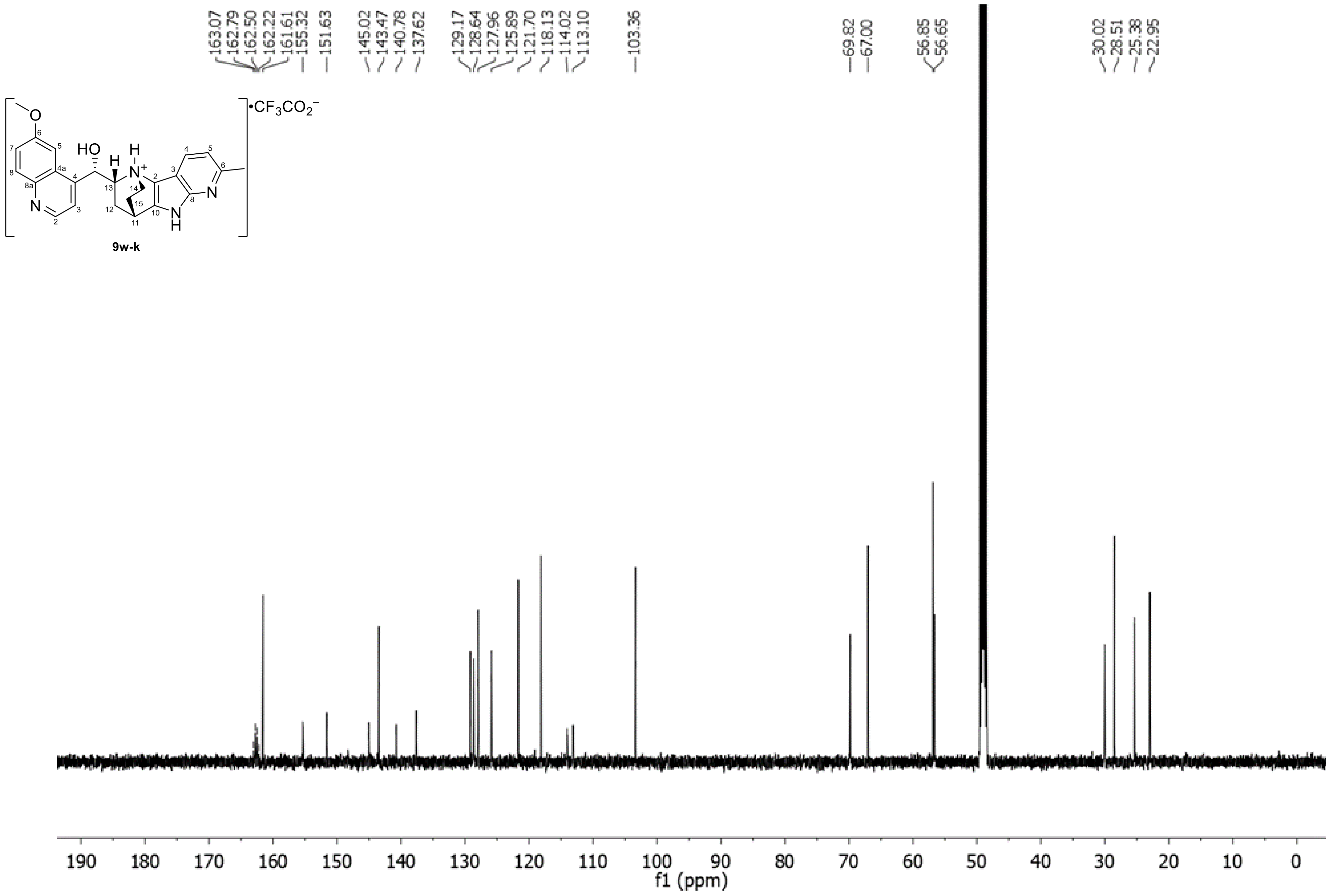
163.07
162.79
162.50
162.22
161.61
155.32
151.63
145.02
143.47
140.78
137.62
129.17
128.64
127.96
125.89
121.70
118.13
114.02
113.10
103.36
69.82
67.00
56.85
56.65
30.02
28.51
25.38
22.95
•CF3CO2−
HO
H
N+
N
N
H
O
9w-k
f1 (ppm)

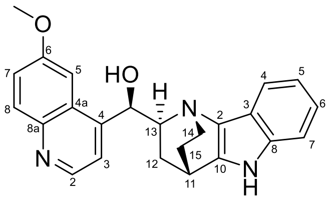

O
6
7
5
HO
H
N
4a
8
4
13
2
3
4
5
6
8a
14
N
12
15
10
8
7
3
11
N
H
2

10a

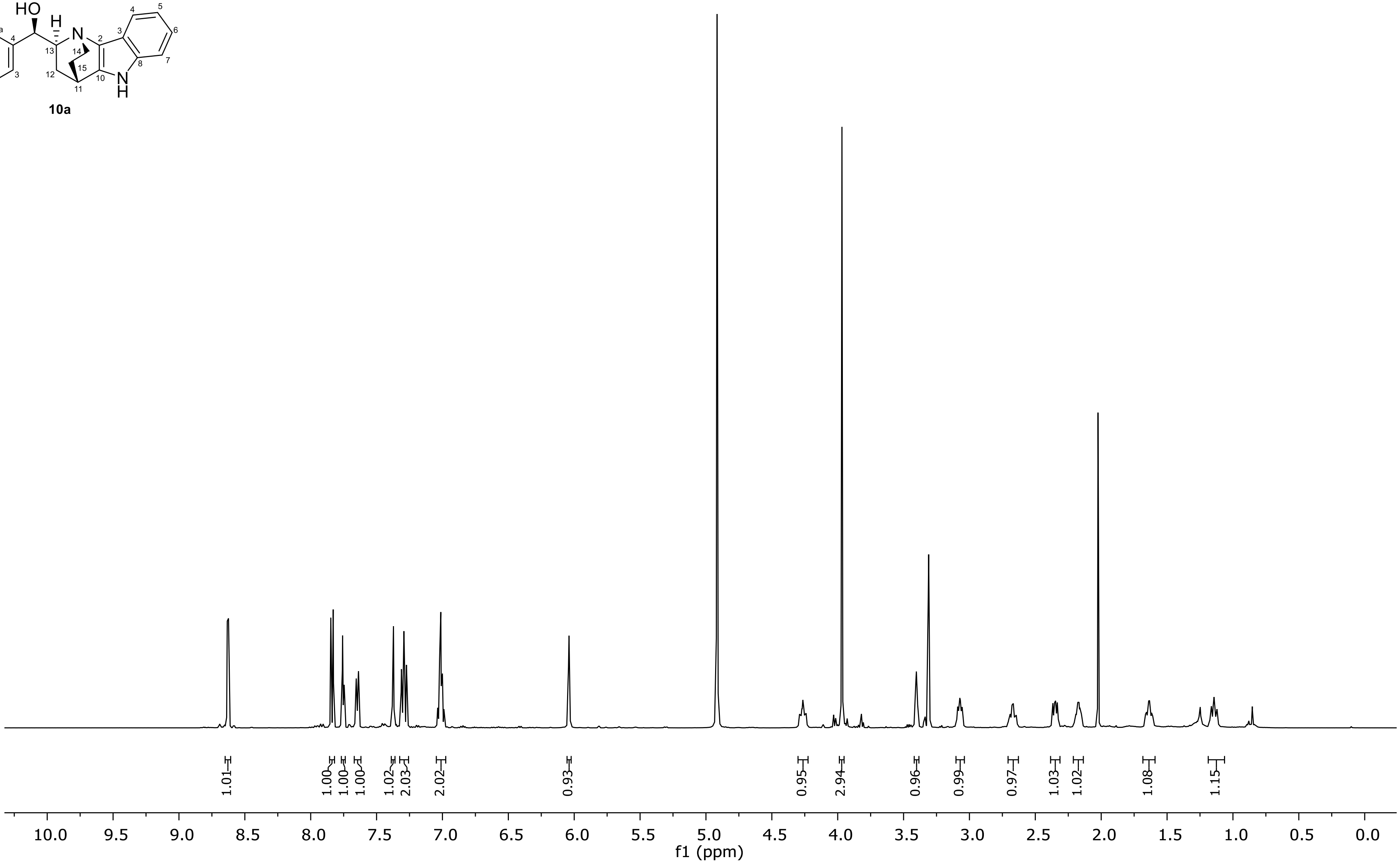

1.01
1.00
1.00
1.00
1.02
2.03
2.02
0.93
0.95
2.94
0.96
0.99
0.97
1.03
1.02
1.08
1.15
10.0 9.5 9.0 8.5 8.0 7.5 7.0 6.5 6.0 5.5 5.0 4.5 4.0 3.5 3.0 2.5 2.0 1.5 1.0 0.5 0.0
f1 (ppm)

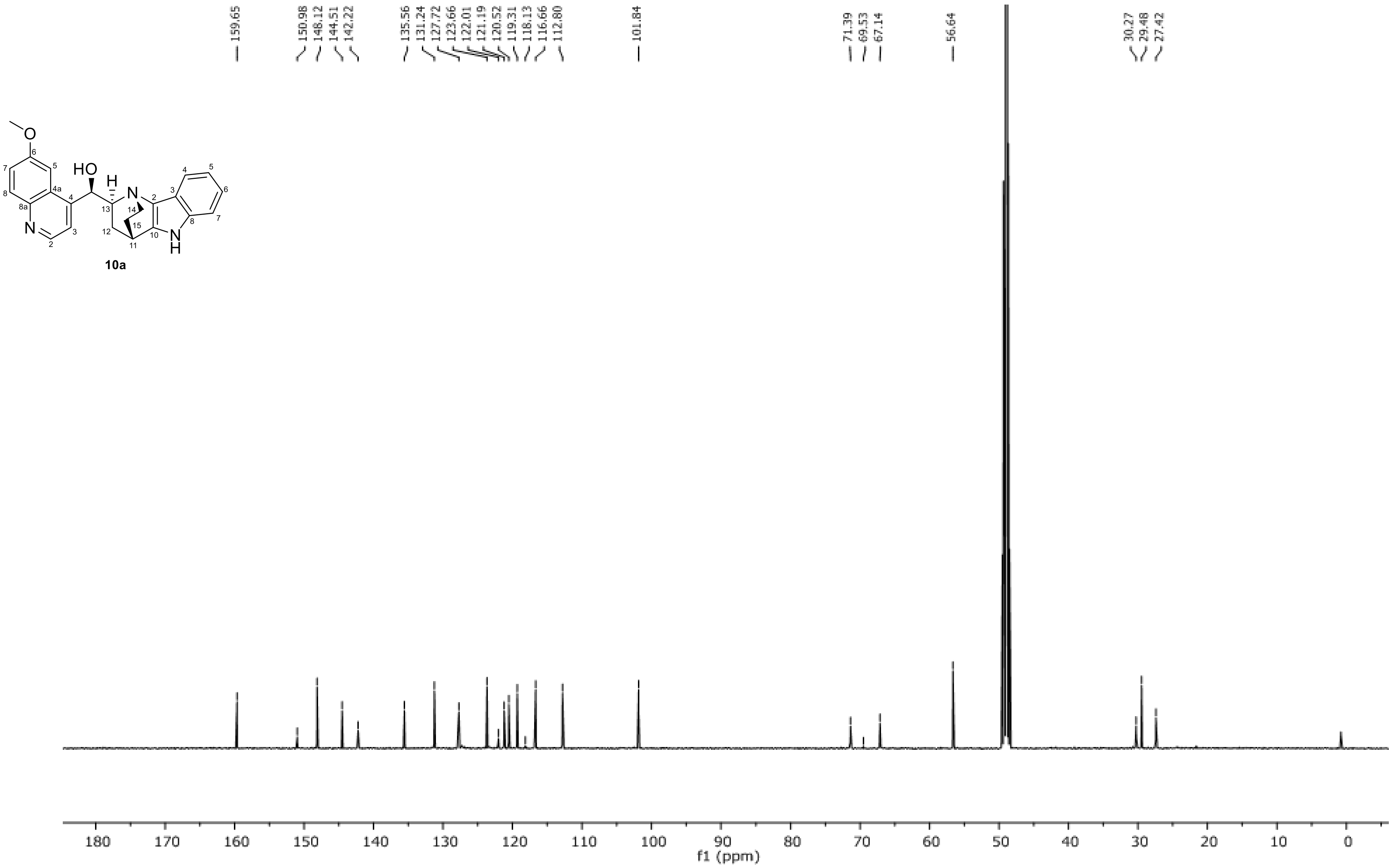
HO
H
N
N
H
O
10a
159.65
150.98
148.12
144.51
142.22
135.56
131.24
127.72
123.66
122.01
121.19
120.52
119.31
118.13
116.66
112.80
101.84
71.39
69.53
67.14
56.64
30.27
29.48
27.42
180 170 160 150 140 130 120 110 100 90 80 70 60 50 40 30 20 10 0
f1 (ppm)

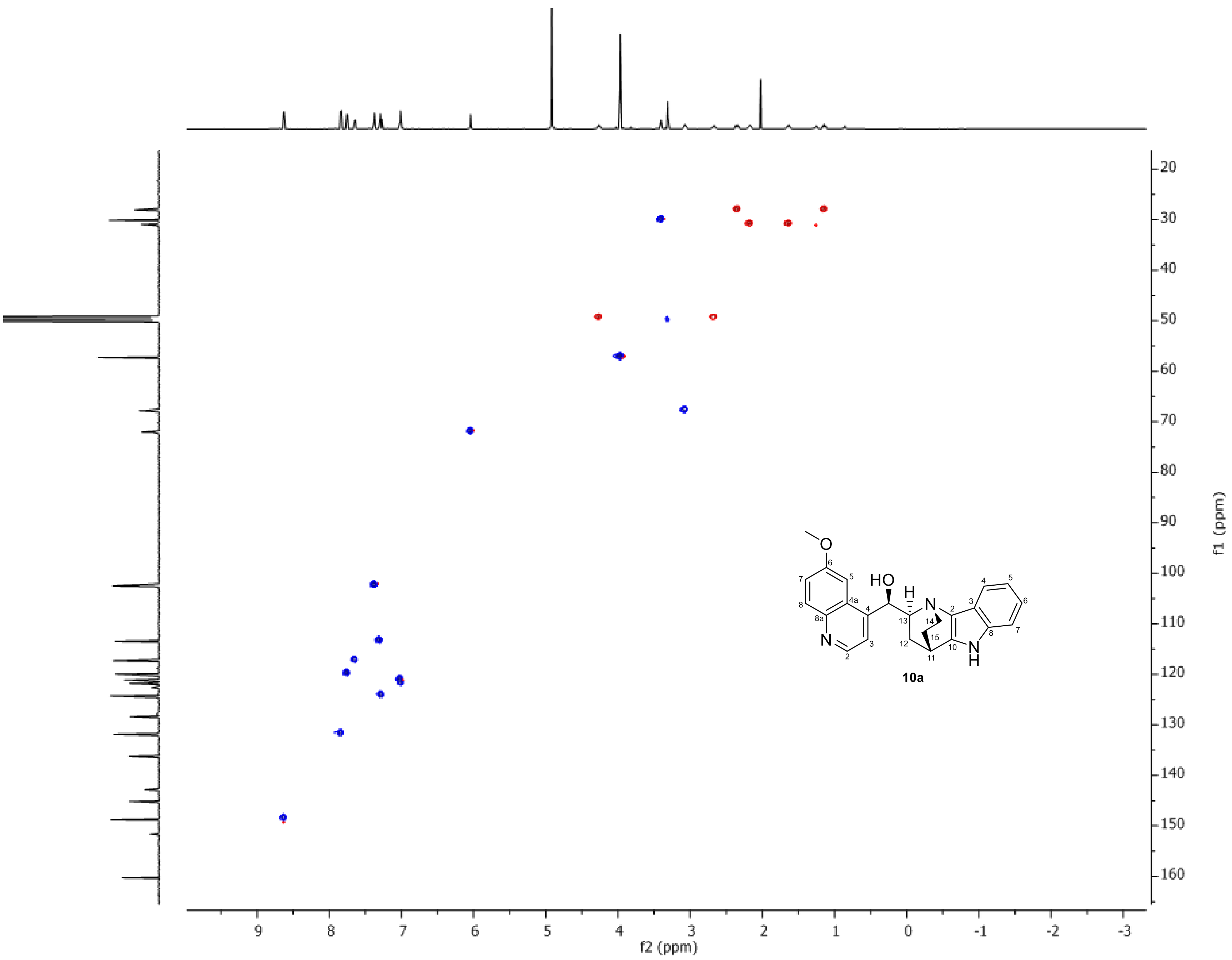

f2 (ppm)
f1 (ppm)
HO
H
N
O
N
H
10a

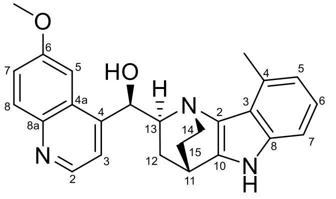

10b

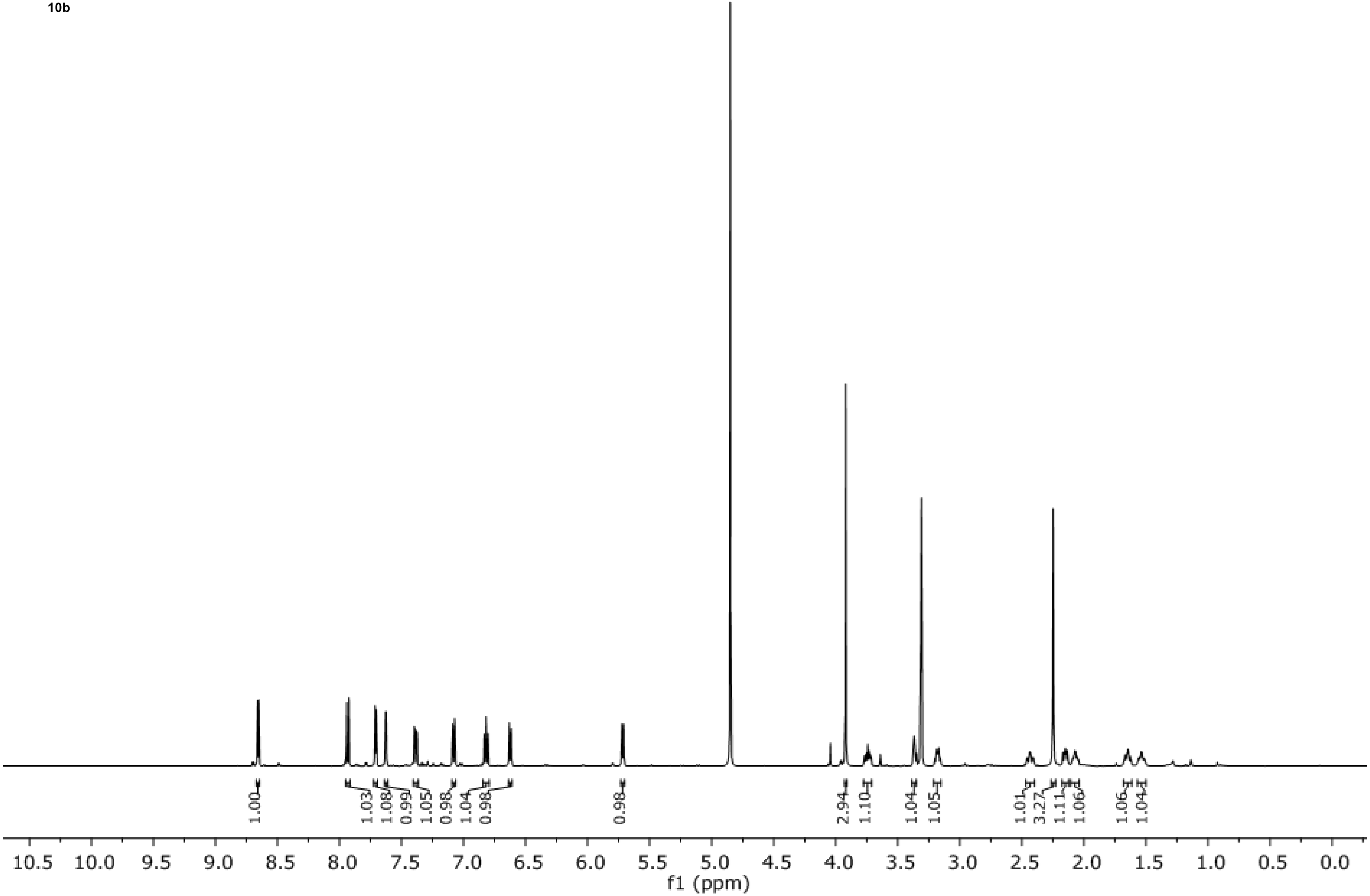
1.00
1.03
1.08
0.99
1.05
0.98
1.04
0.98
0.98
2.94
1.10
1.04
1.05
1.01
3.27
1.11
1.06
1.06
1.04
10.5 10.0 9.5 9.0 8.5 8.0 7.5 7.0 6.5 6.0 5.5 5.0 4.5 4.0 3.5 3.0 2.5 2.0 1.5 1.0 0.5 0.0
f1 (ppm)

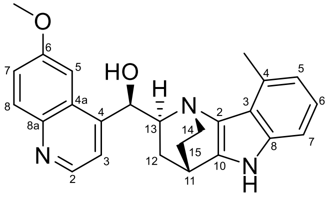

10b

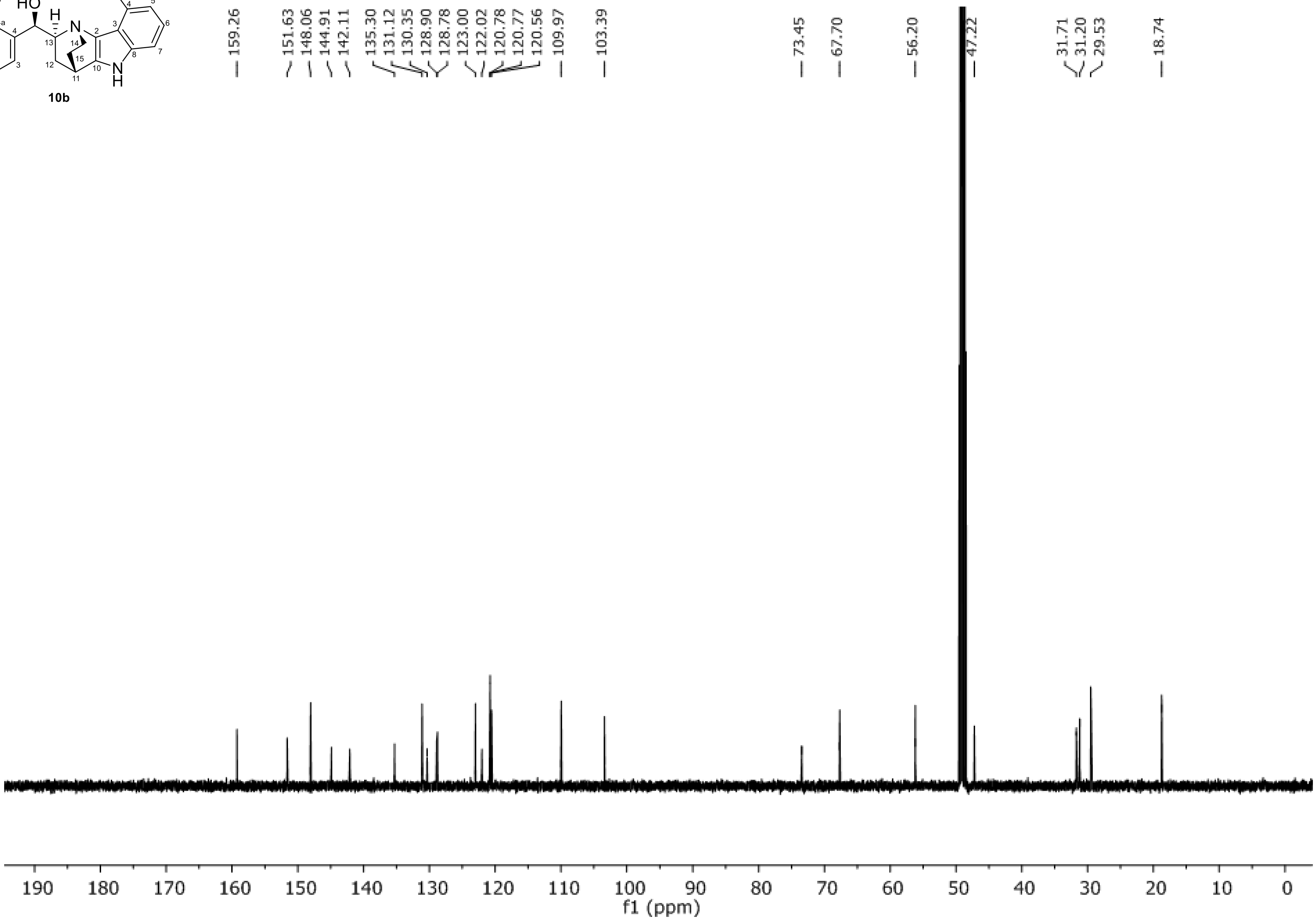
159.26
151.63
148.06
144.91
142.11
135.30
131.12
130.35
128.90
128.78
123.00
122.02
120.78
120.77
120.56
109.97
103.39
73.45
67.70
56.20
47.22
31.71
31.20
29.53
18.74
190 180 170 160 150 140 130 120 110 100 90 80 70 60 50 40 30 20 10 0
f1 (ppm)

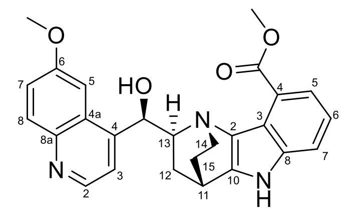

10c

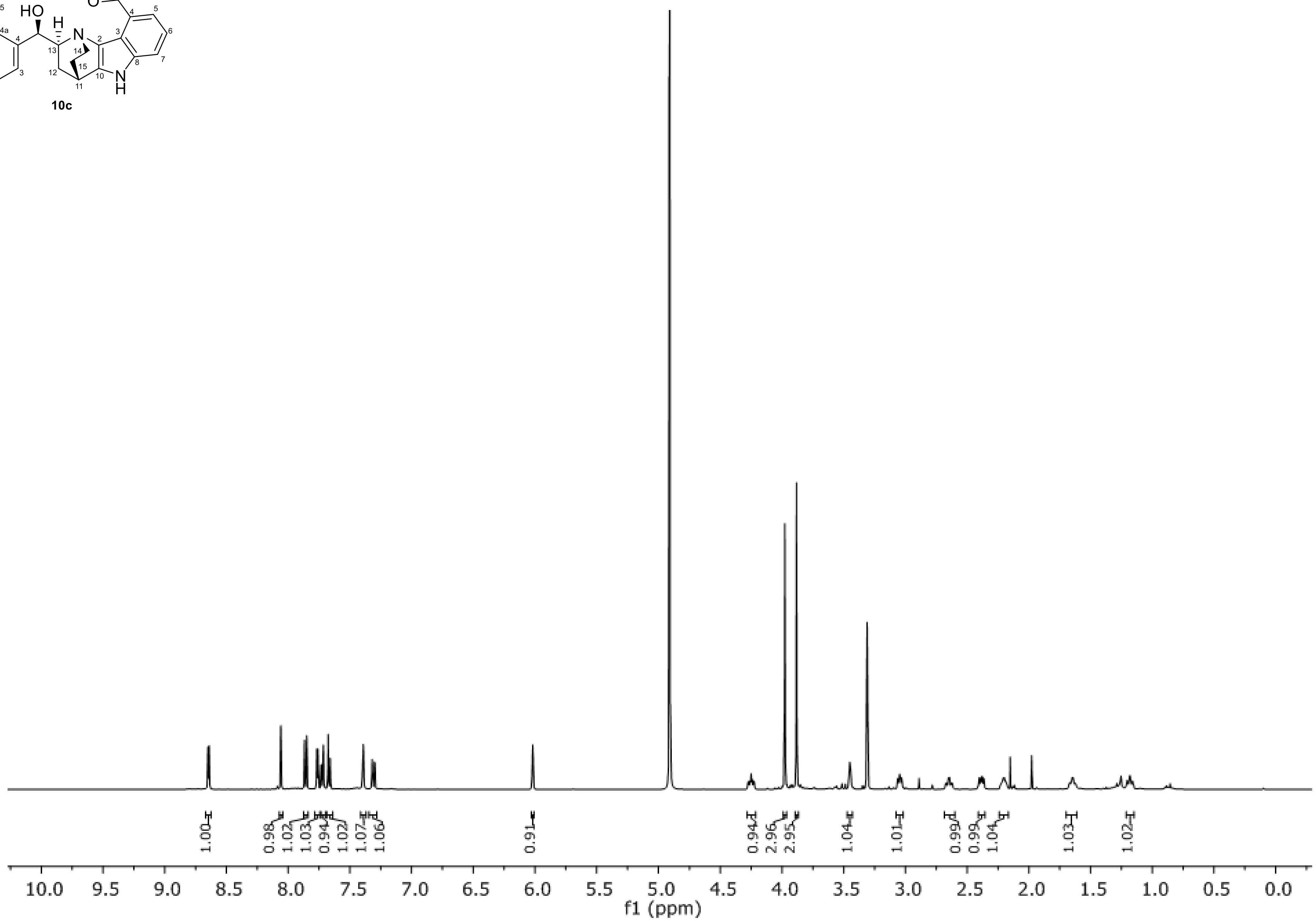
1.00
0.98
1.02
1.03
0.94
1.02
1.07
1.06
0.91
0.94
2.96
2.95
1.04
1.01
0.99
0.99
1.04
1.03
1.02
10.0 9.5 9.0 8.5 8.0 7.5 7.0 6.5 6.0 5.5 5.0 4.5 4.0 3.5 3.0 2.5 2.0 1.5 1.0 0.5 0.0
f1 (ppm)

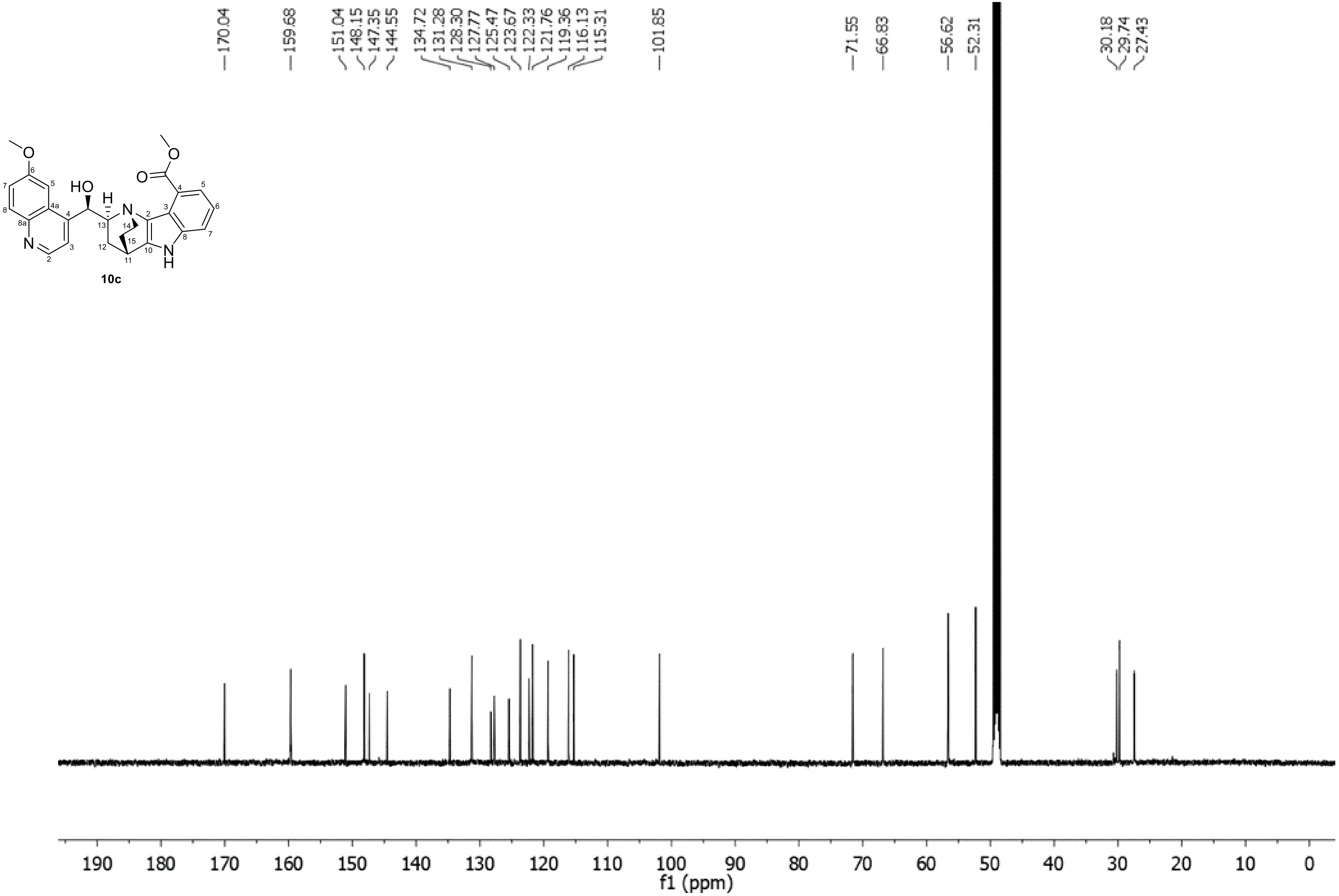
170.04
159.68
151.04
148.15
147.35
144.55
134.72
131.28
128.30
127.77
125.47
123.67
122.33
121.76
119.36
116.13
115.31
101.85
71.55
66.83
56.62
52.31
30.18
29.74
27.43
190 180 170 160 150 140 130 120 110 100 90 80 70 60 50 40 30 20 10 0
f1 (ppm)
10c

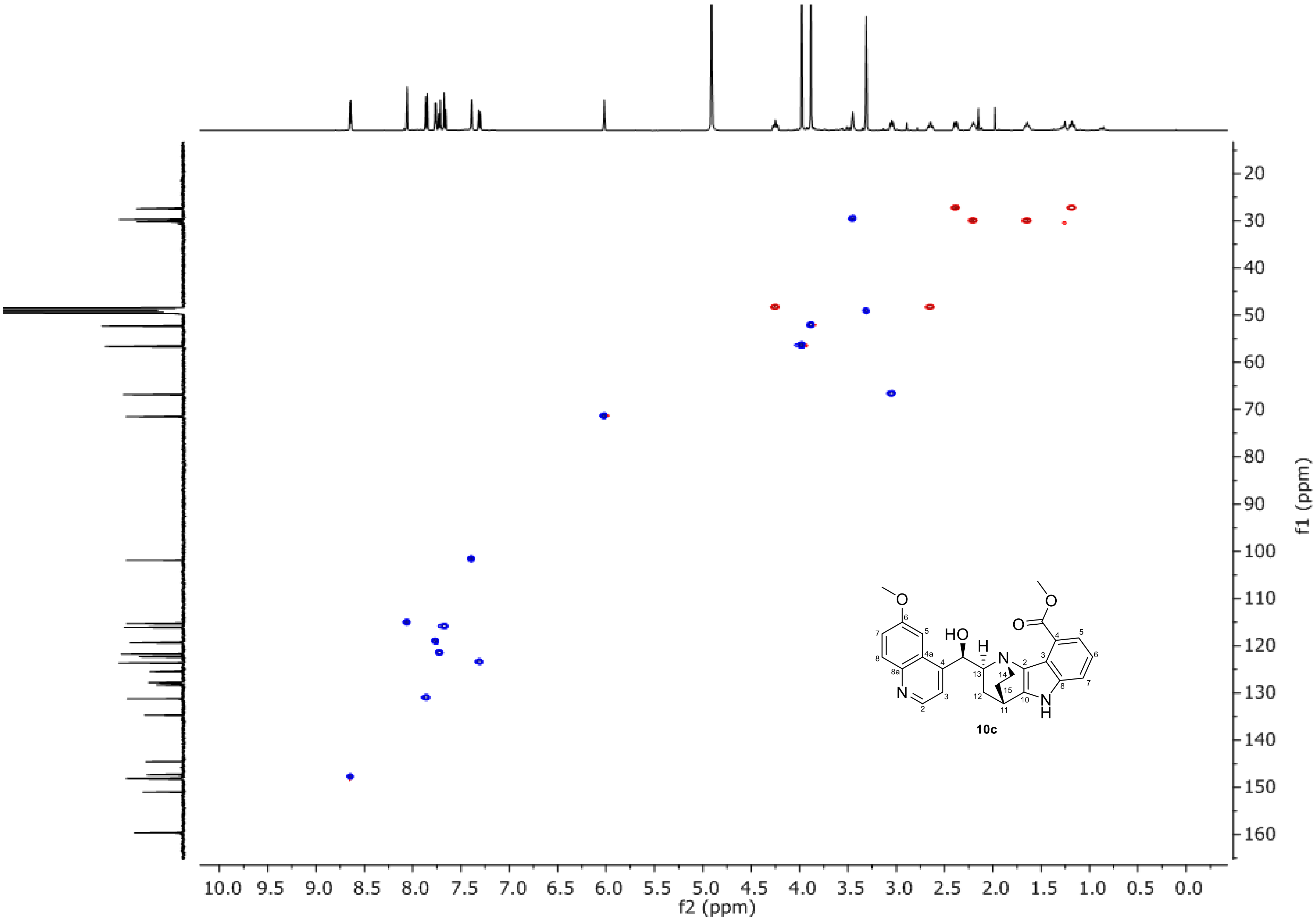
f1 (ppm)
f2 (ppm)
HO
H
N
O
O
O
N
NH
10c

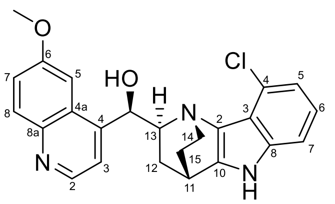
Cl
HO
H
N
N
H
O

10d

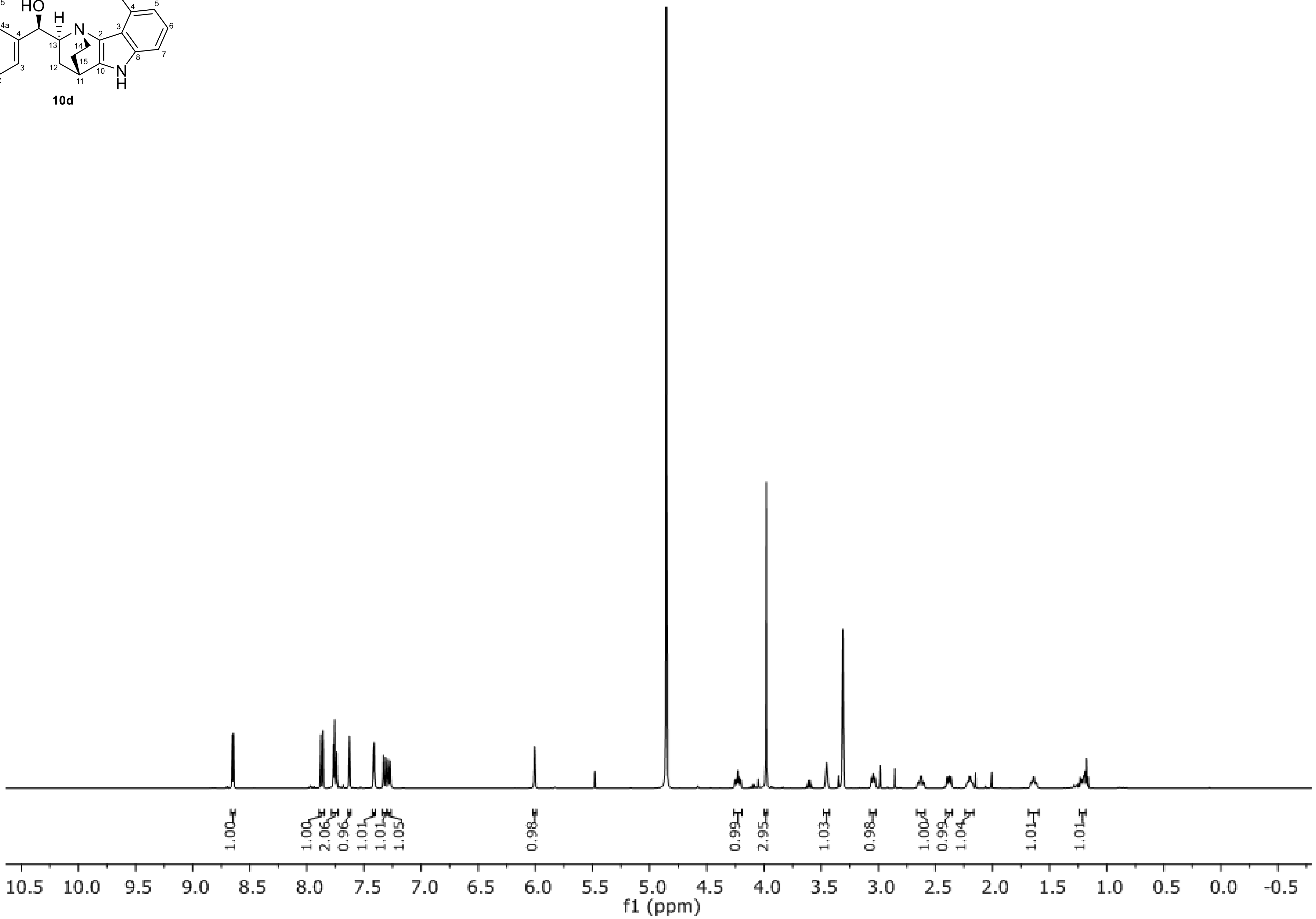
1.00
1.00
2.06
0.96
1.01
1.01
1.05
0.98
0.99
2.95
1.03
0.98
1.00
0.99
1.04
1.01
1.01
10.5 10.0 9.5 9.0 8.5 8.0 7.5 7.0 6.5 6.0 5.5 5.0 4.5 4.0 3.5 3.0 2.5 2.0 1.5 1.0 0.5 0.0 -0.5
f1 (ppm)

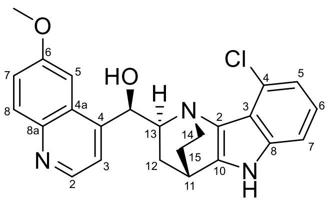

10d

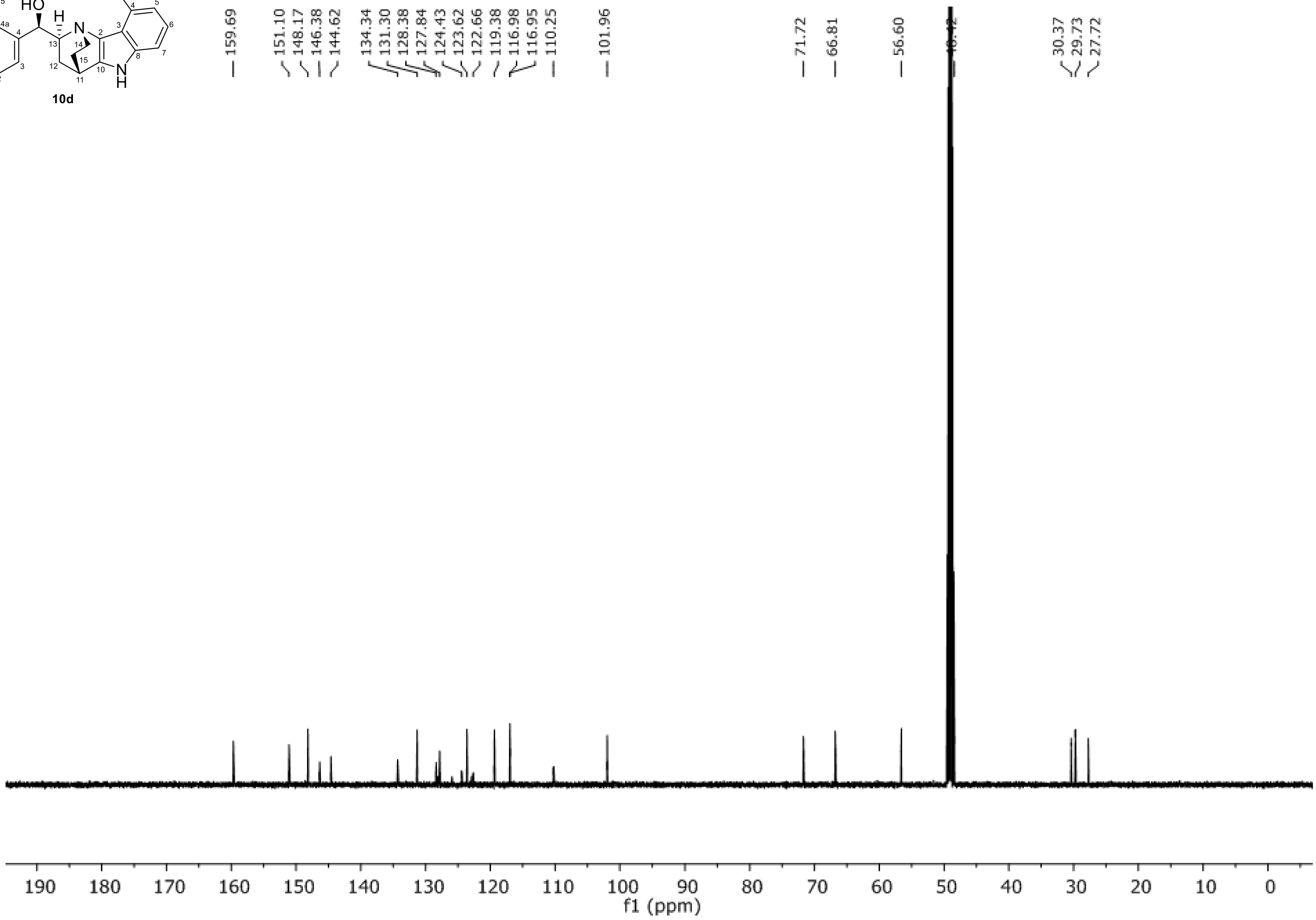
159.69
151.10
148.17
146.38
144.62
134.34
131.30
128.38
127.84
124.43
123.62
122.66
119.38
116.98
116.95
110.25
101.96
71.72
66.81
56.60
30.37
29.73
27.72
190 180 170 160 150 140 130 120 110 100 90 80 70 60 50 40 30 20 10 0
f1 (ppm)

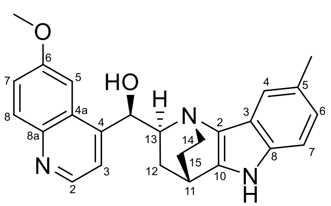

10e

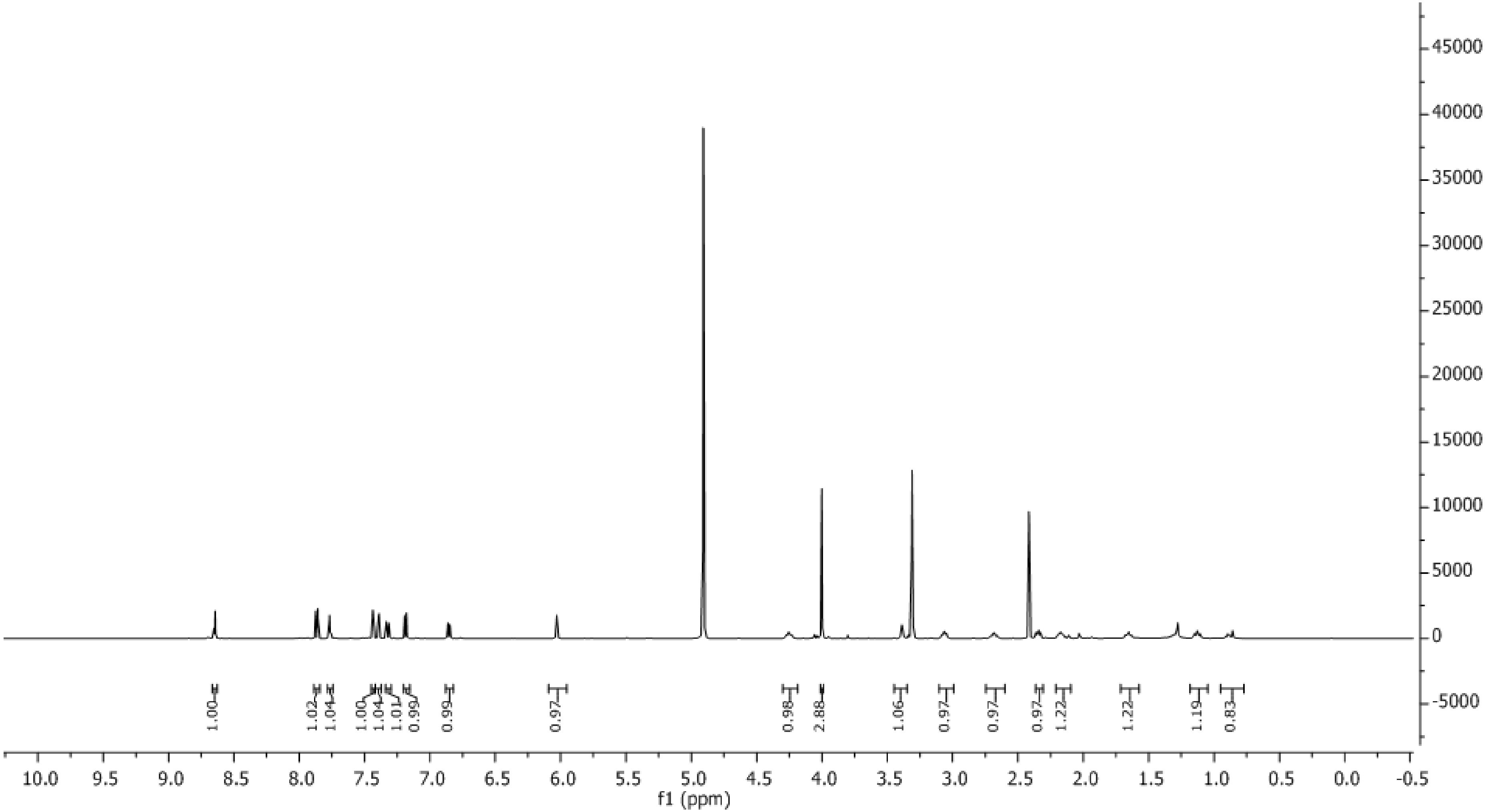
45000
40000
35000
30000
25000
20000
15000
10000
5000
0
-5000
1.00
1.02
1.04
1.00
1.04
1.01
0.99
0.99
0.97
0.98
2.88
1.06
0.97
0.97
0.97
1.22
1.22
1.19
0.83
10.0 9.5 9.0 8.5 8.0 7.5 7.0 6.5 6.0 5.5 5.0 4.5 4.0 3.5 3.0 2.5 2.0 1.5 1.0 0.5 0.0 -0.5
f1 (ppm)

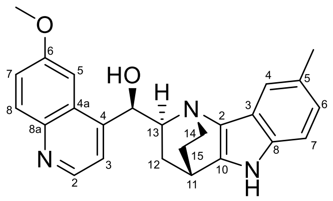
HO
H
N
NH
10e

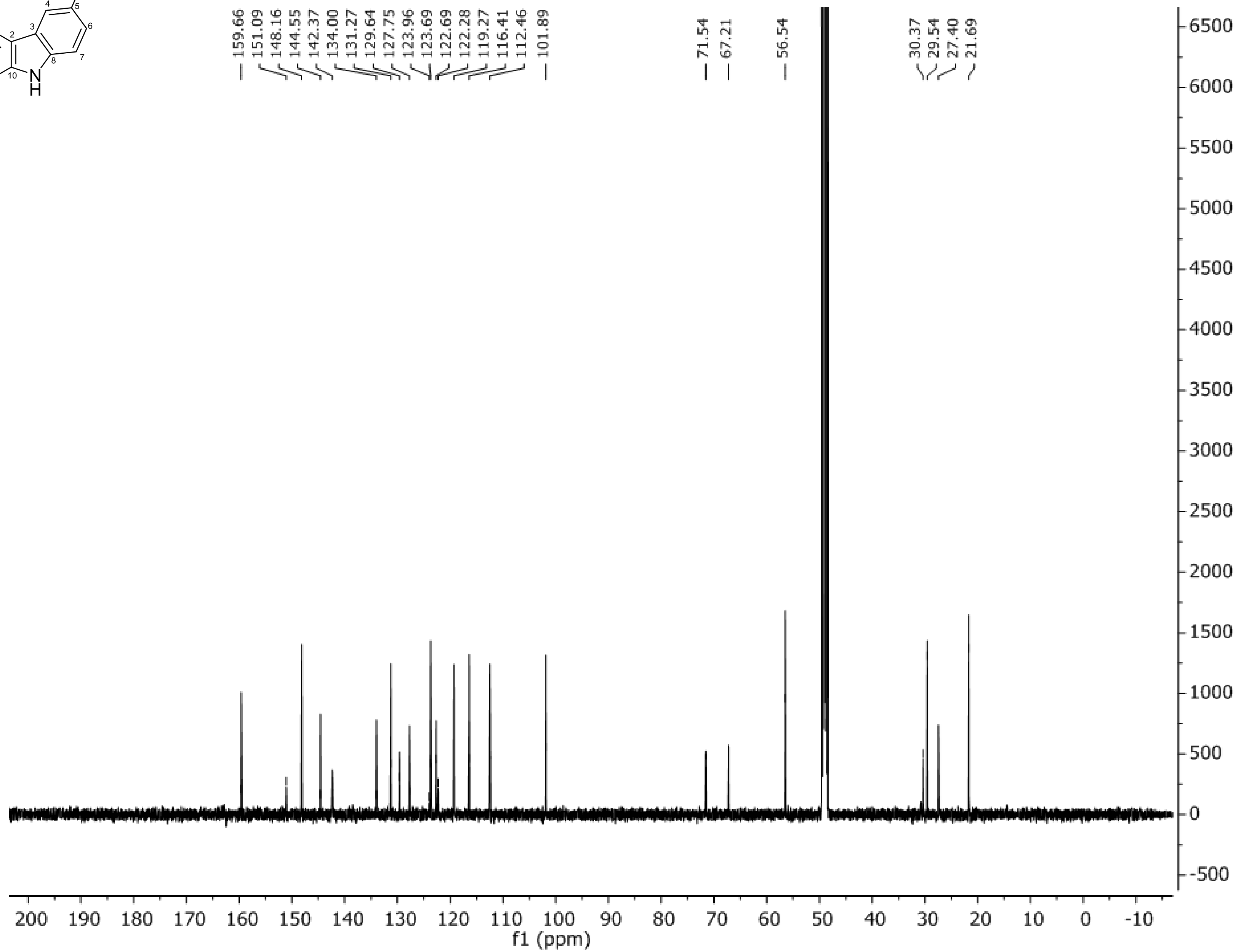
159.66
151.09
148.16
144.55
142.37
134.00
131.27
129.64
127.75
123.96
123.69
122.69
122.28
119.27
116.41
112.46
101.89
71.54
67.21
56.54
30.37
29.54
27.40
21.69
f1 (ppm)

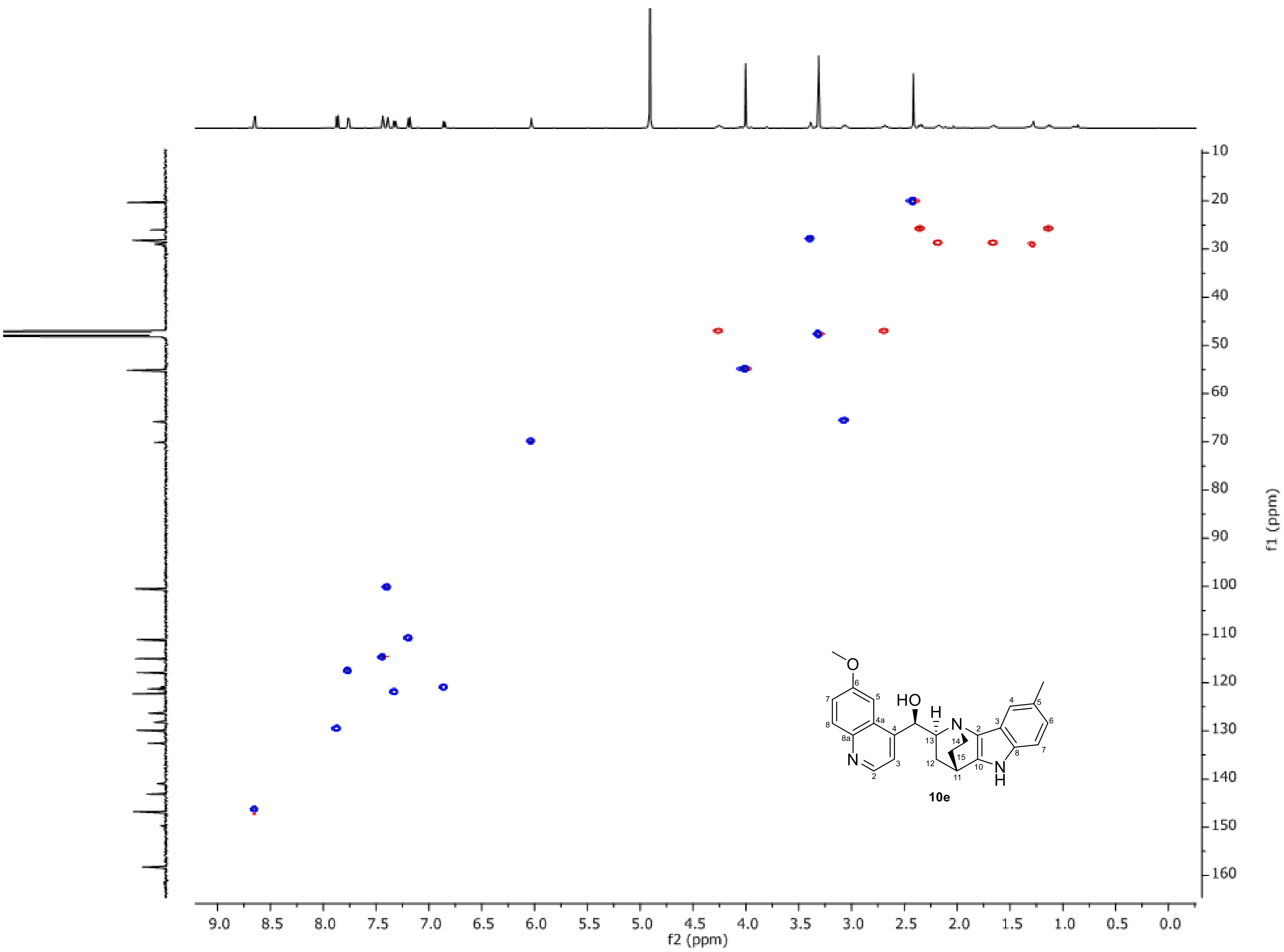
f2 (ppm)
f1 (ppm)
10e

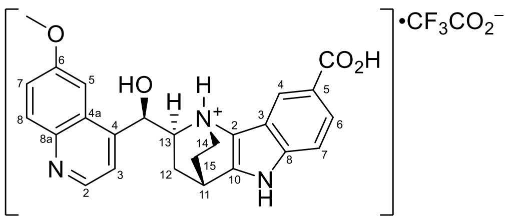
•CF3CO2–
CO2H
HO
N+
NH
N

10f

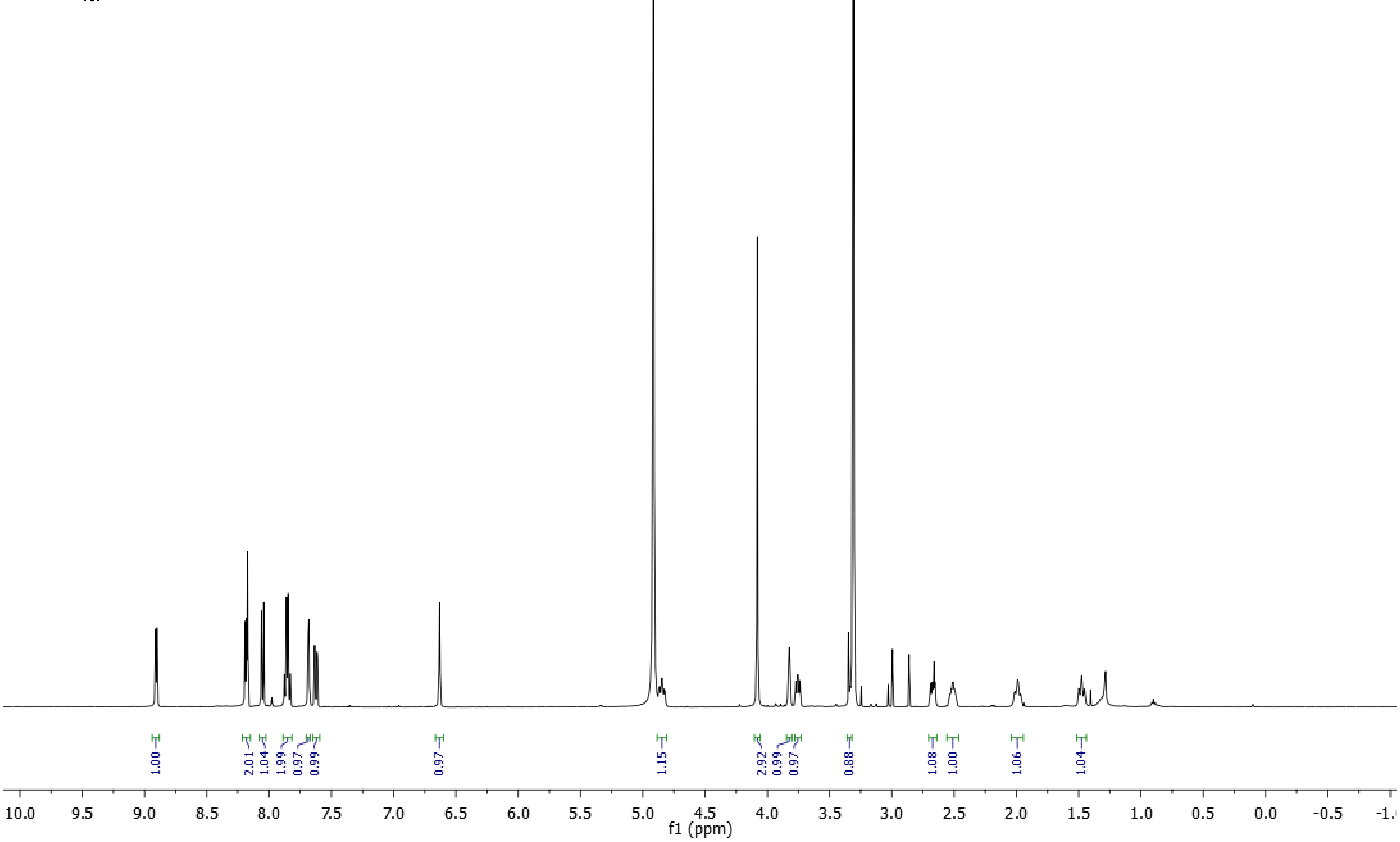
1.00
2.01
1.04
1.99
0.97
0.99
0.97
1.15
2.92
0.99
0.97
0.88
1.08
1.00
1.06
1.04
10.0 9.5 9.0 8.5 8.0 7.5 7.0 6.5 6.0 5.5 5.0 4.5 4.0 3.5 3.0 2.5 2.0 1.5 1.0 0.5 0.0 -0.5
f1 (ppm)

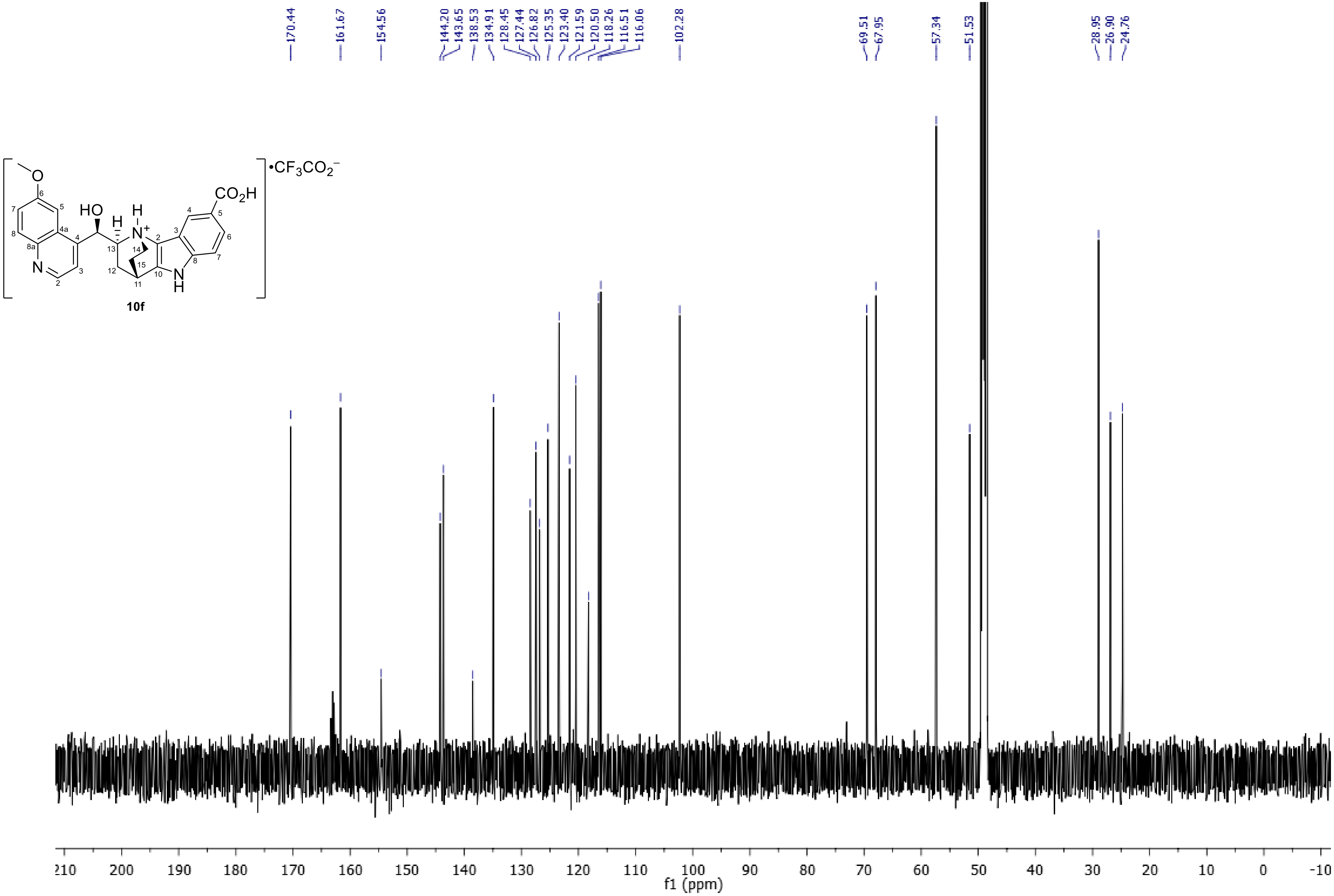
170.44
161.67
154.56
144.20
143.65
138.53
134.91
128.45
127.44
126.82
125.35
123.40
121.59
120.50
118.26
116.51
116.06
102.28
69.51
67.95
57.34
51.53
28.95
26.90
24.76
•$CF_3CO_2^-$
CO_2H
HO
10f
f1 (ppm)

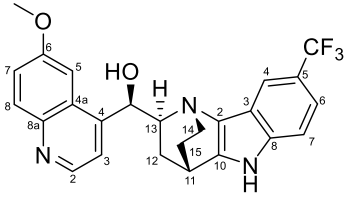

CF3
HO
H
N
H

10h

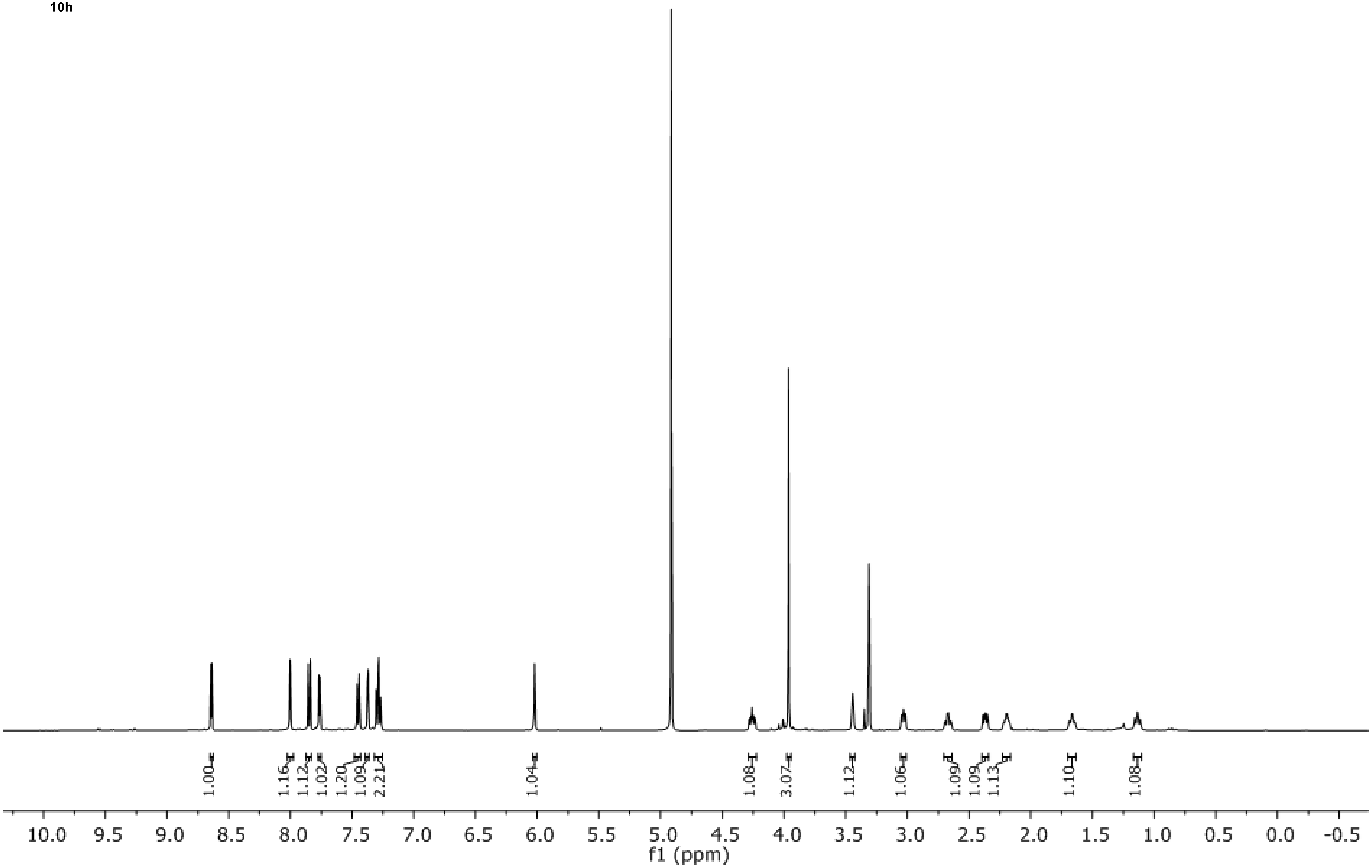

1.00
1.16
1.12
1.02
1.20
1.09
2.21
1.04
1.08
3.07
1.12
1.06
1.09
1.09
1.13
1.10
1.08
10.0 9.5 9.0 8.5 8.0 7.5 7.0 6.5 6.0 5.5 5.0 4.5 4.0 3.5 3.0 2.5 2.0 1.5 1.0 0.5 0.0 -0.5
f1 (ppm)

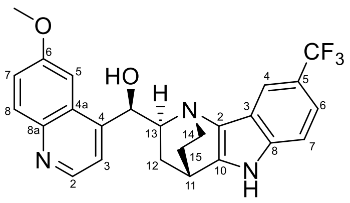
HO
CF3
N
H

10h

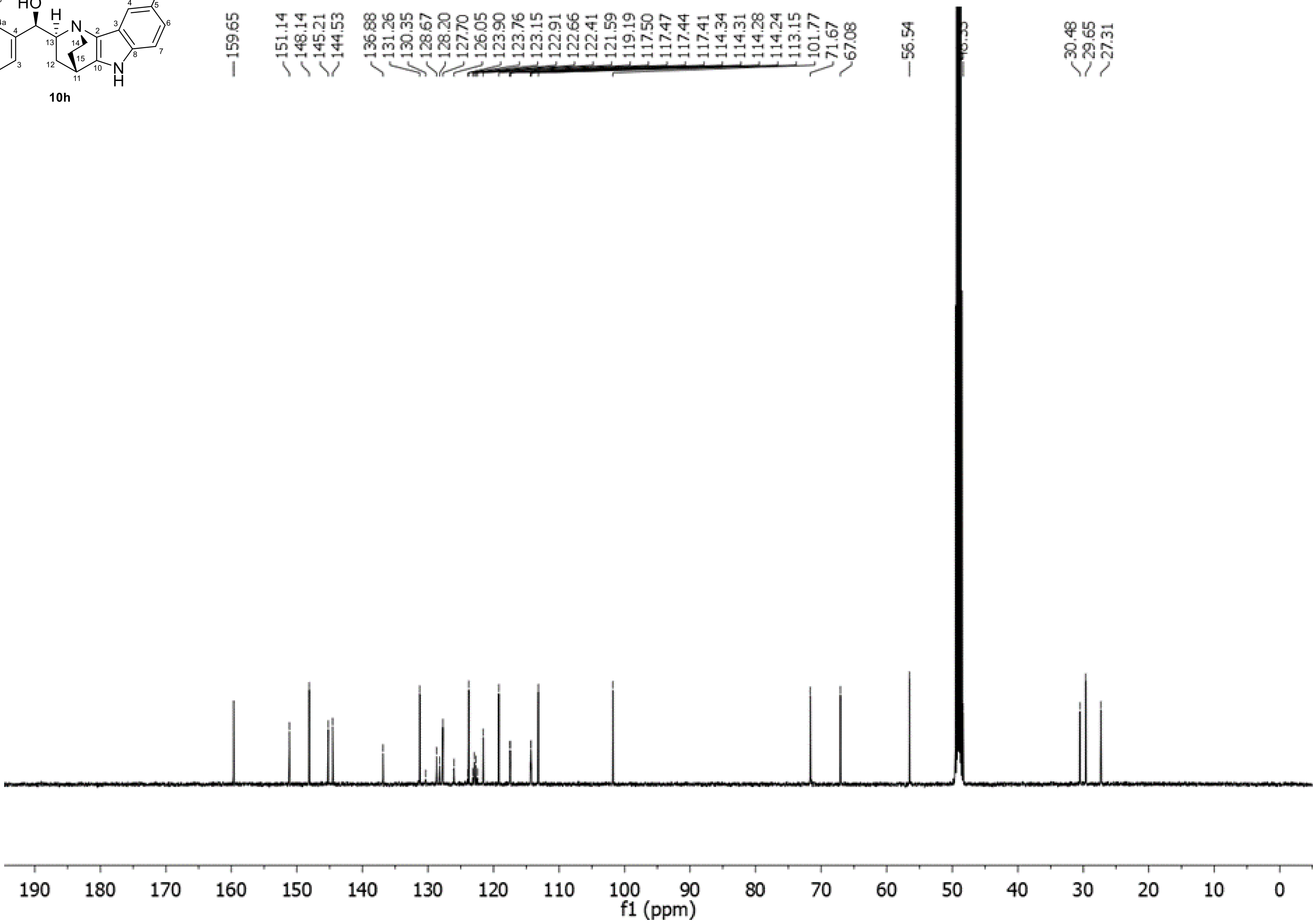
159.65
151.14
148.14
145.21
144.53
136.88
131.26
130.35
128.67
128.20
127.70
126.05
123.90
123.76
123.15
122.91
122.66
122.41
121.59
119.19
117.50
117.47
117.44
117.41
114.34
114.31
114.28
114.24
113.15
101.77
71.67
67.08
56.54
30.48
29.65
27.31
190 180 170 160 150 140 130 120 110 100 90 80 70 60 50 40 30 20 10 0
f1 (ppm)

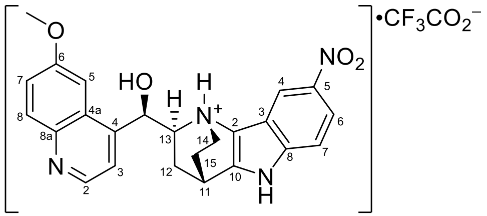
•CF3CO2−
NO2
HO
H
H
N+
N
H
N
O

10i

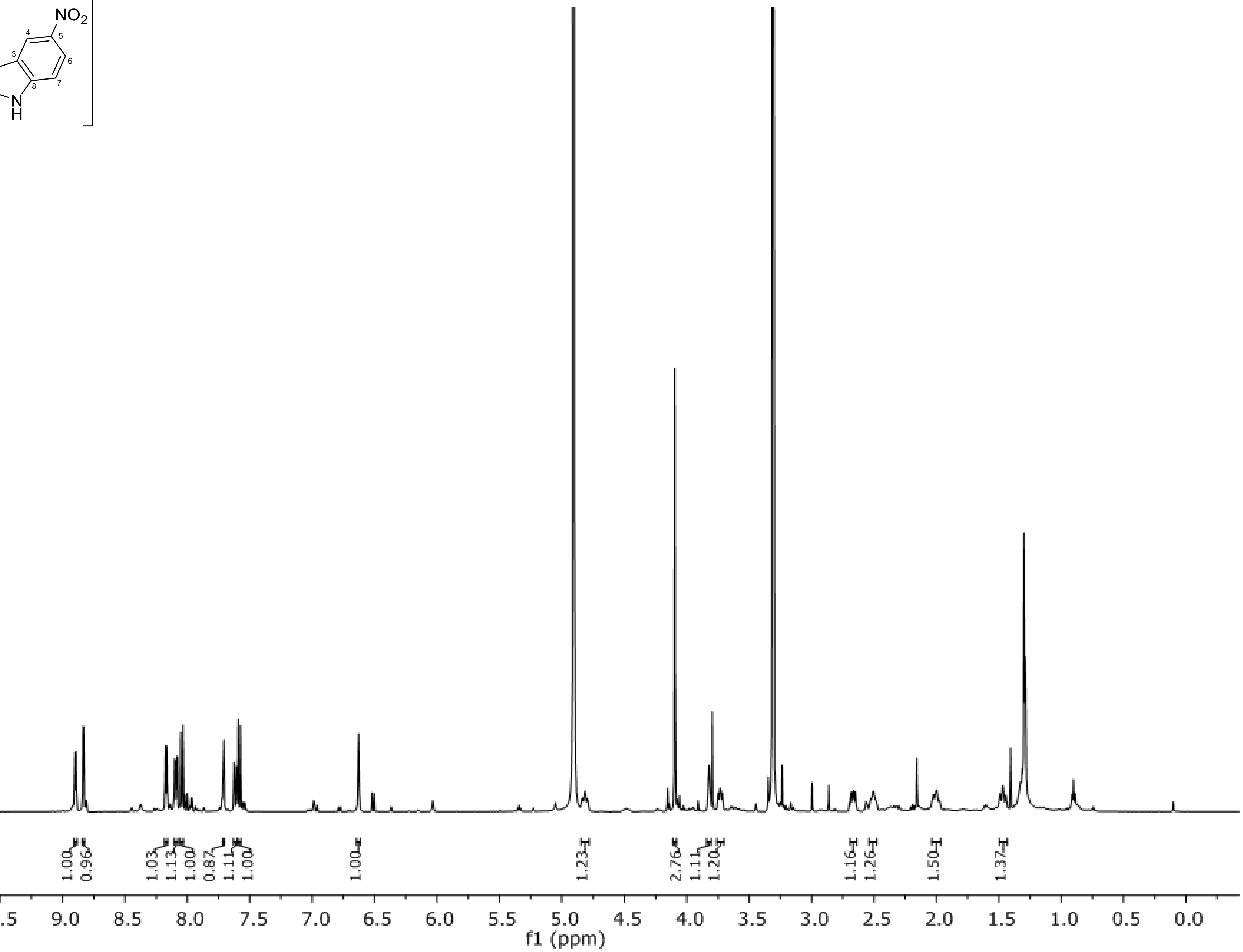
1.00
0.96
1.03
1.13
1.00
0.87
1.11
1.00
1.00
1.23
2.76
1.11
1.20
1.16
1.26
1.50
1.37
10.0 9.5 9.0 8.5 8.0 7.5 7.0 6.5 6.0 5.5 5.0 4.5 4.0 3.5 3.0 2.5 2.0 1.5 1.0 0.5 0.0
f1 (ppm)

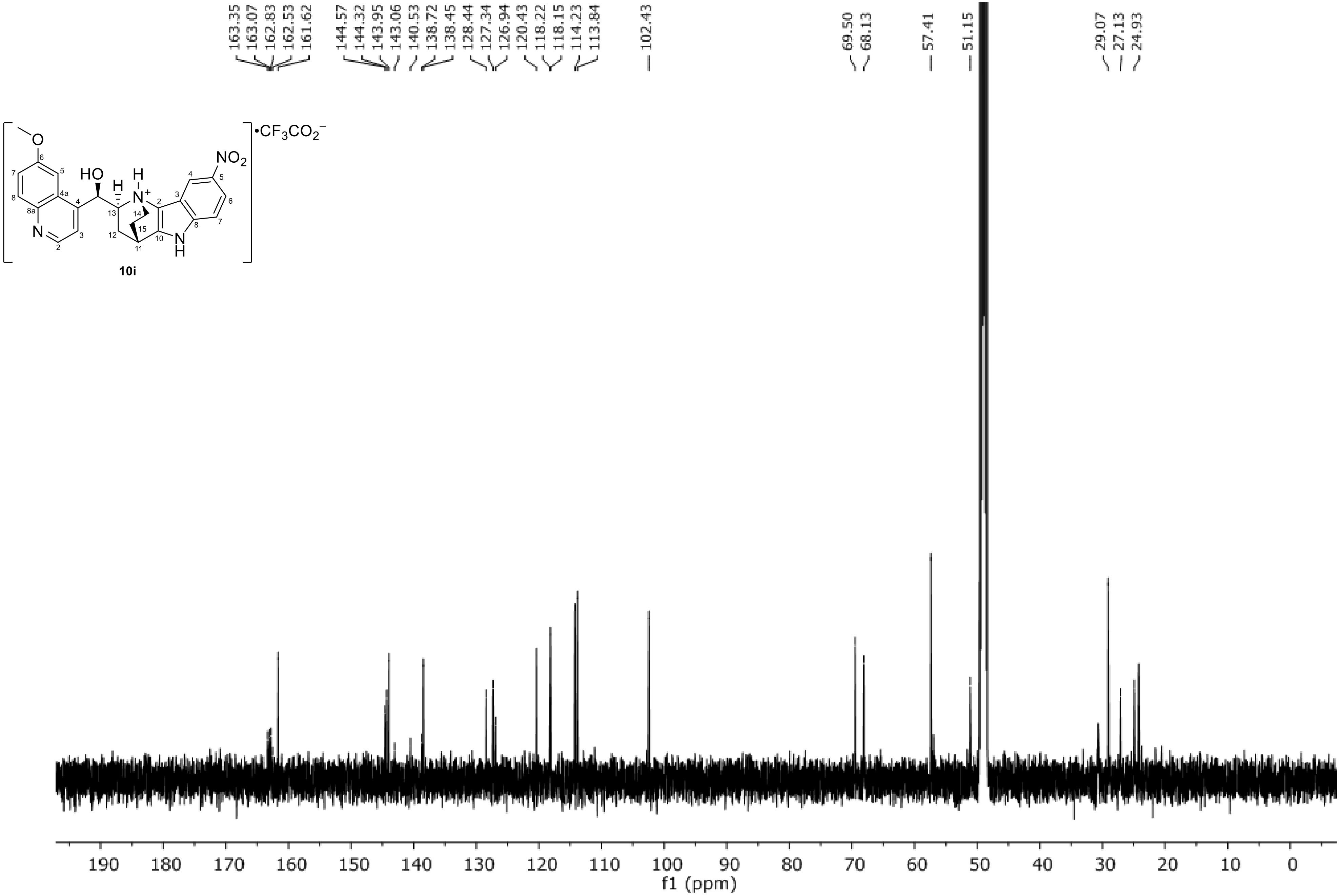

10i

163.35
163.07
162.83
162.53
161.62
144.57
144.32
143.95
143.06
140.53
138.72
138.45
128.44
127.34
126.94
120.43
118.22
118.15
114.23
113.84
102.43
69.50
68.13
57.41
51.15
29.07
27.13
24.93
190
180
170
160
150
140
130
120
110
100
90
80
70
60
50
40
30
20
10
0
f1 (ppm)

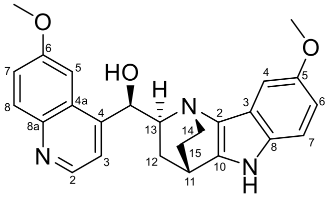

10j

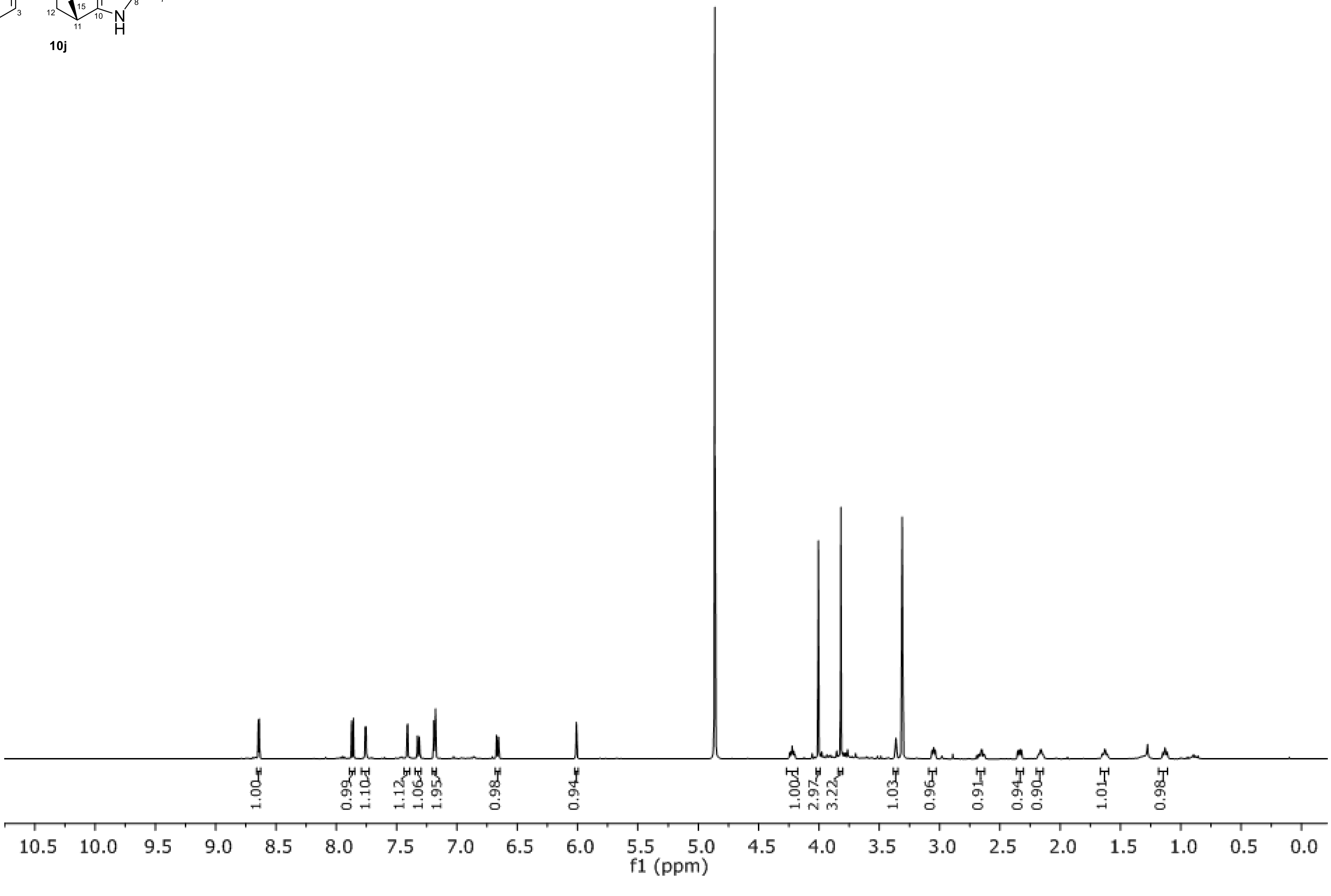

1.00
0.99
1.10
1.12
1.06
1.95
0.98
0.94
1.00
2.97
3.22
1.03
0.96
0.91
0.94
0.90
1.01
0.98
10.5 10.0 9.5 9.0 8.5 8.0 7.5 7.0 6.5 6.0 5.5 5.0 4.5 4.0 3.5 3.0 2.5 2.0 1.5 1.0 0.5 0.0
f1 (ppm)

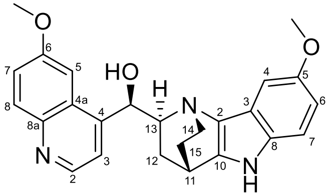

HO
H
N
NH
O
O
N

10j

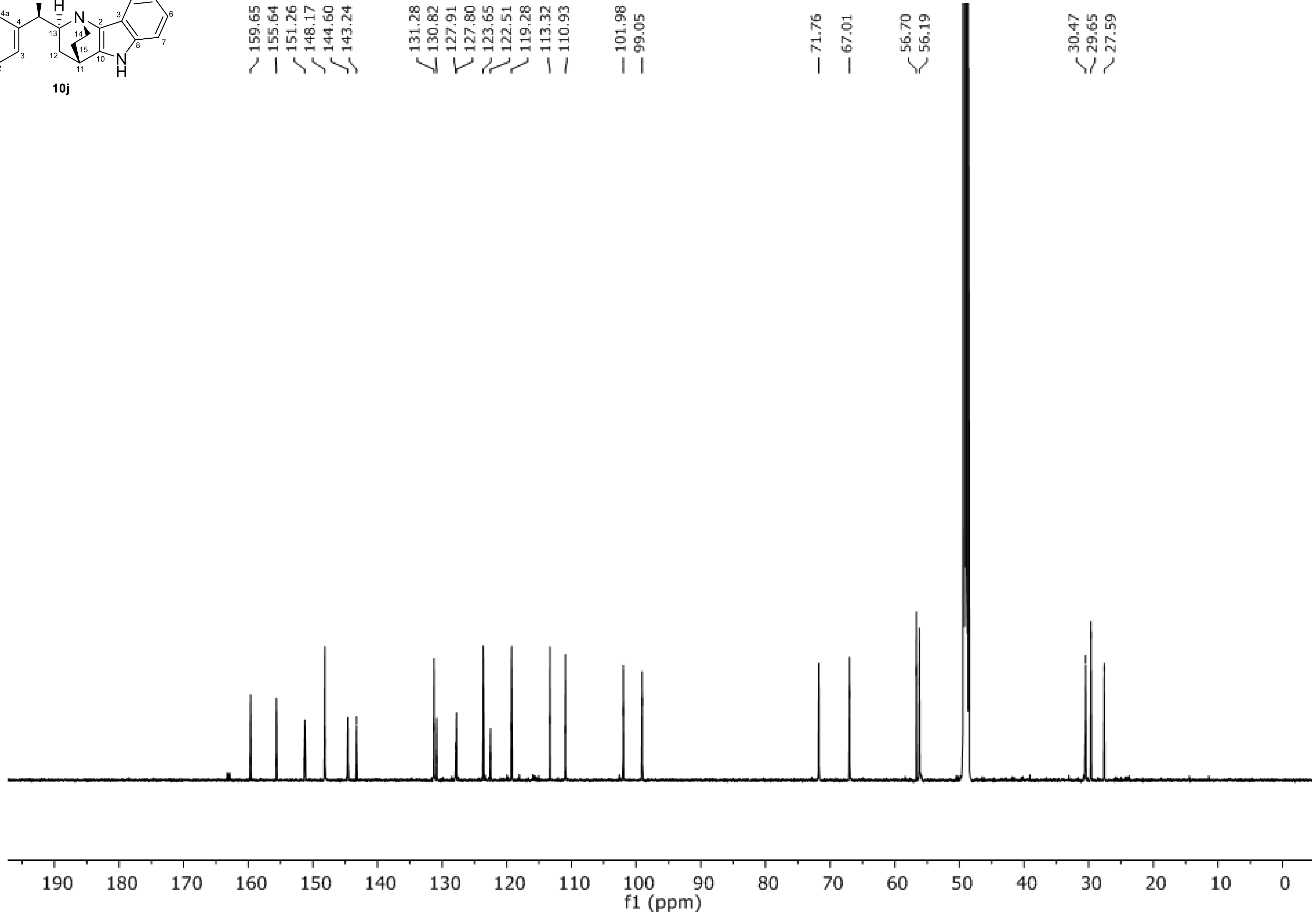

159.65
155.64
151.26
148.17
144.60
143.24
131.28
130.82
127.91
127.80
123.65
122.51
119.28
113.32
110.93
101.98
99.05
71.76
67.01
56.70
56.19
30.47
29.65
27.59
190 180 170 160 150 140 130 120 110 100 90 80 70 60 50 40 30 20 10 0
f1 (ppm)

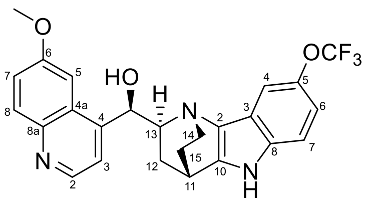

10k

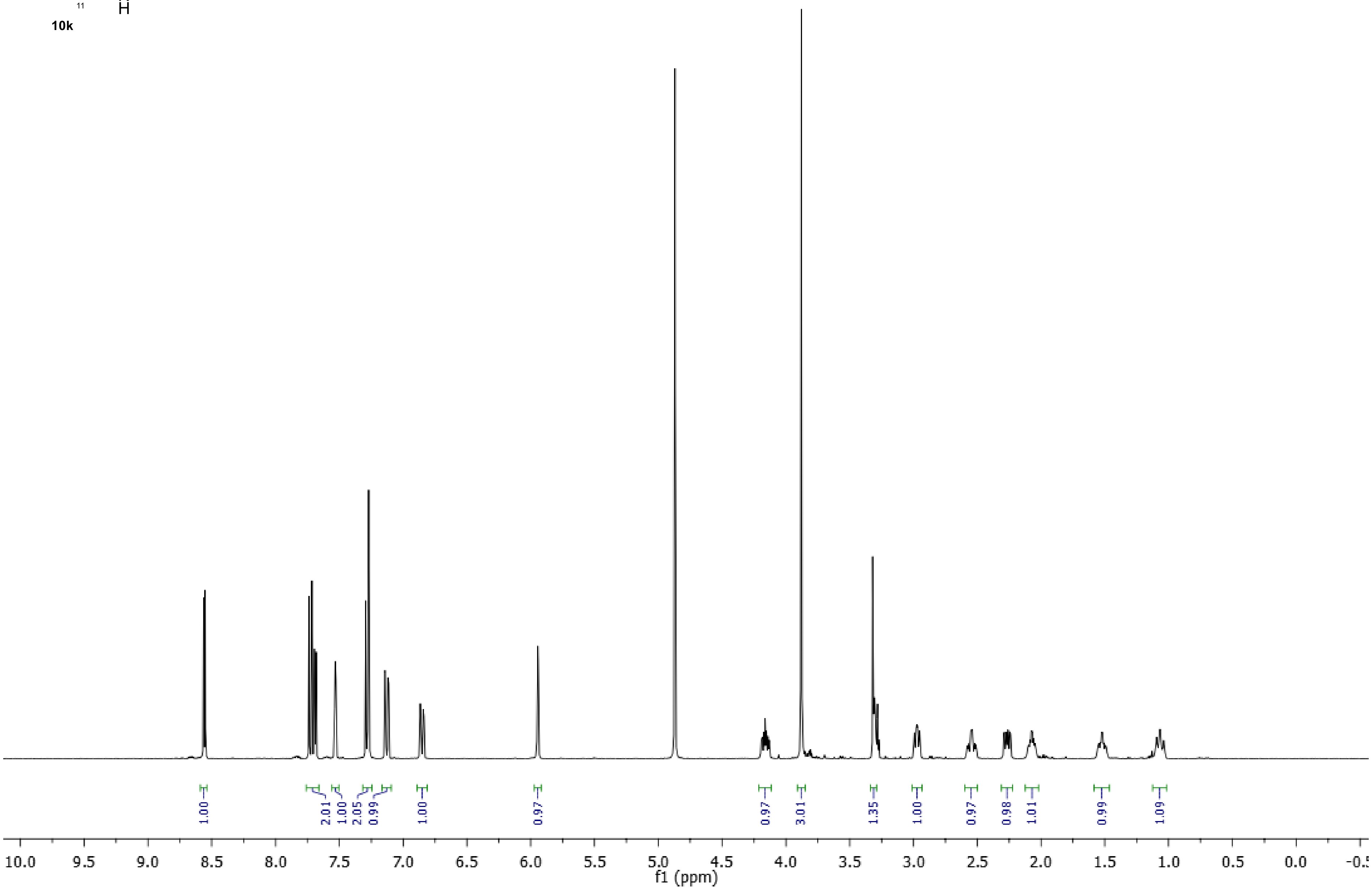

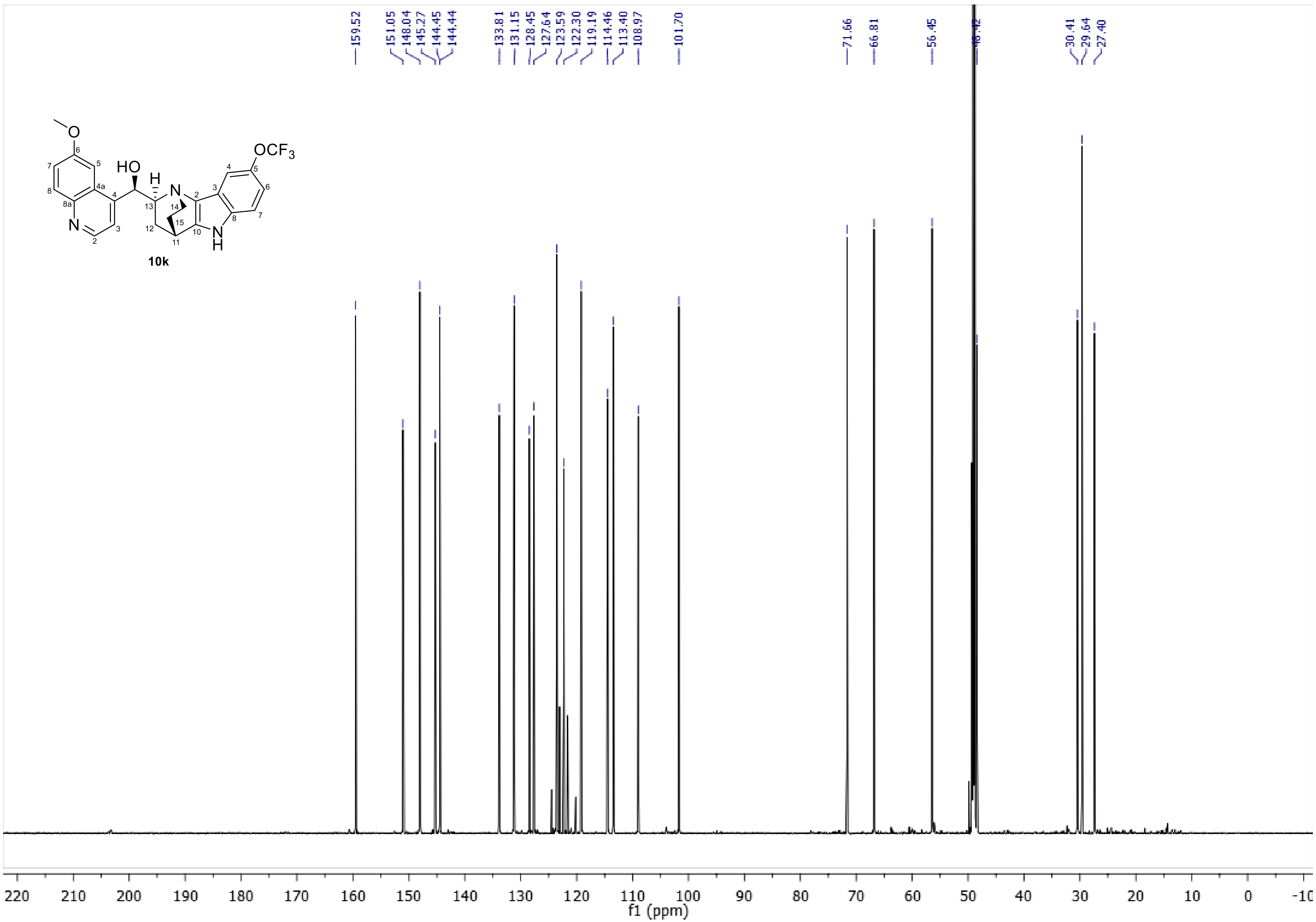

159.52
151.05
148.04
145.27
144.45
144.44
133.81
131.15
128.45
127.64
123.59
122.30
119.19
114.46
113.40
108.97
101.70
71.66
66.81
56.45
48.42
30.41
29.64
27.40
HO
N
OCF3
N
H
10k
f1 (ppm)

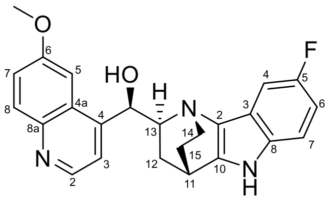

10l

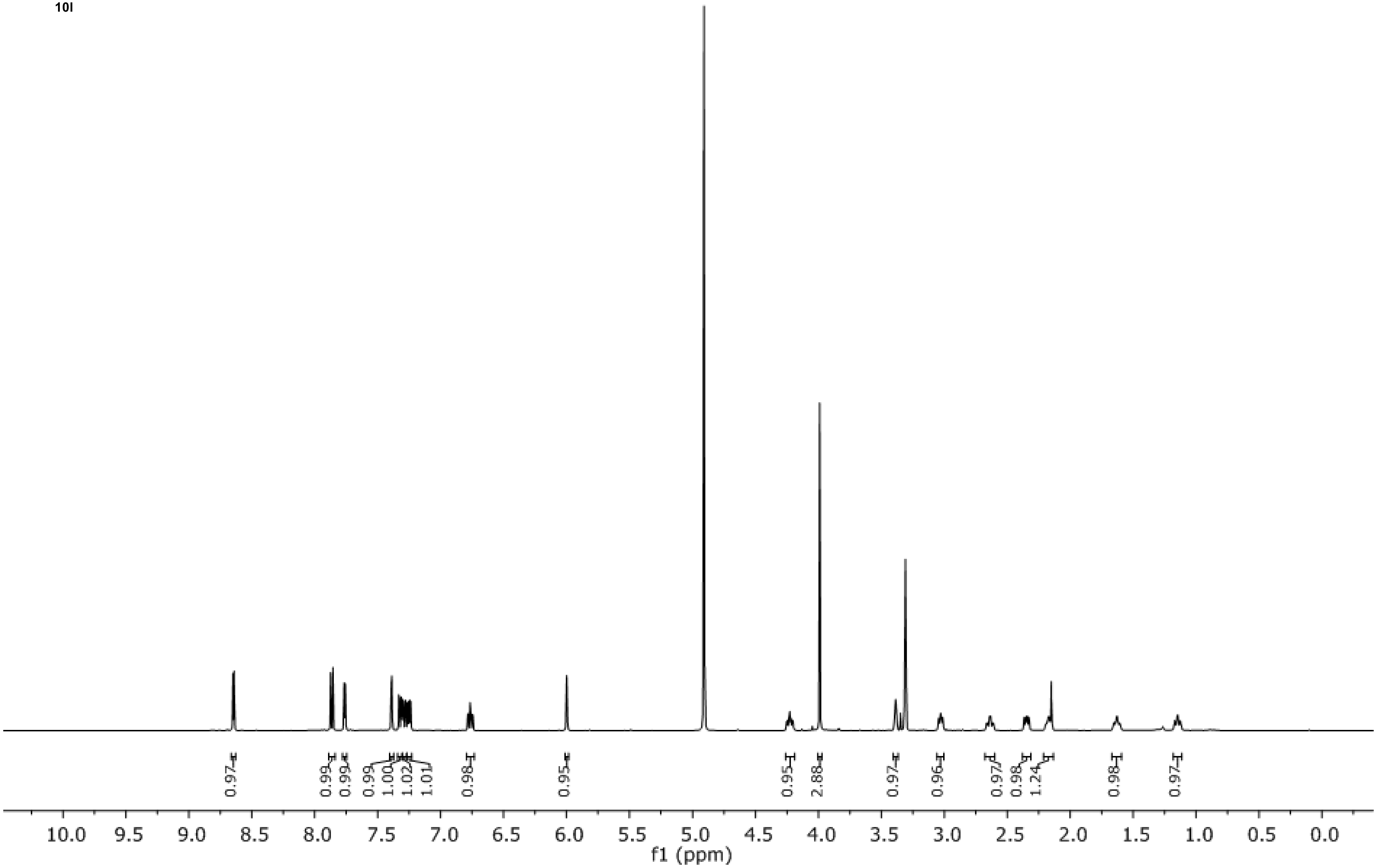
0.97
0.99
0.99
0.99
1.00
1.02
1.01
0.98
0.95
0.95
2.88
0.97
0.96
0.97
0.98
1.24
0.98
0.97
10.0 9.5 9.0 8.5 8.0 7.5 7.0 6.5 6.0 5.5 5.0 4.5 4.0 3.5 3.0 2.5 2.0 1.5 1.0 0.5 0.0
f1 (ppm)

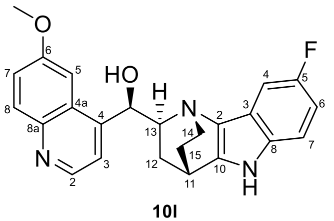

10l

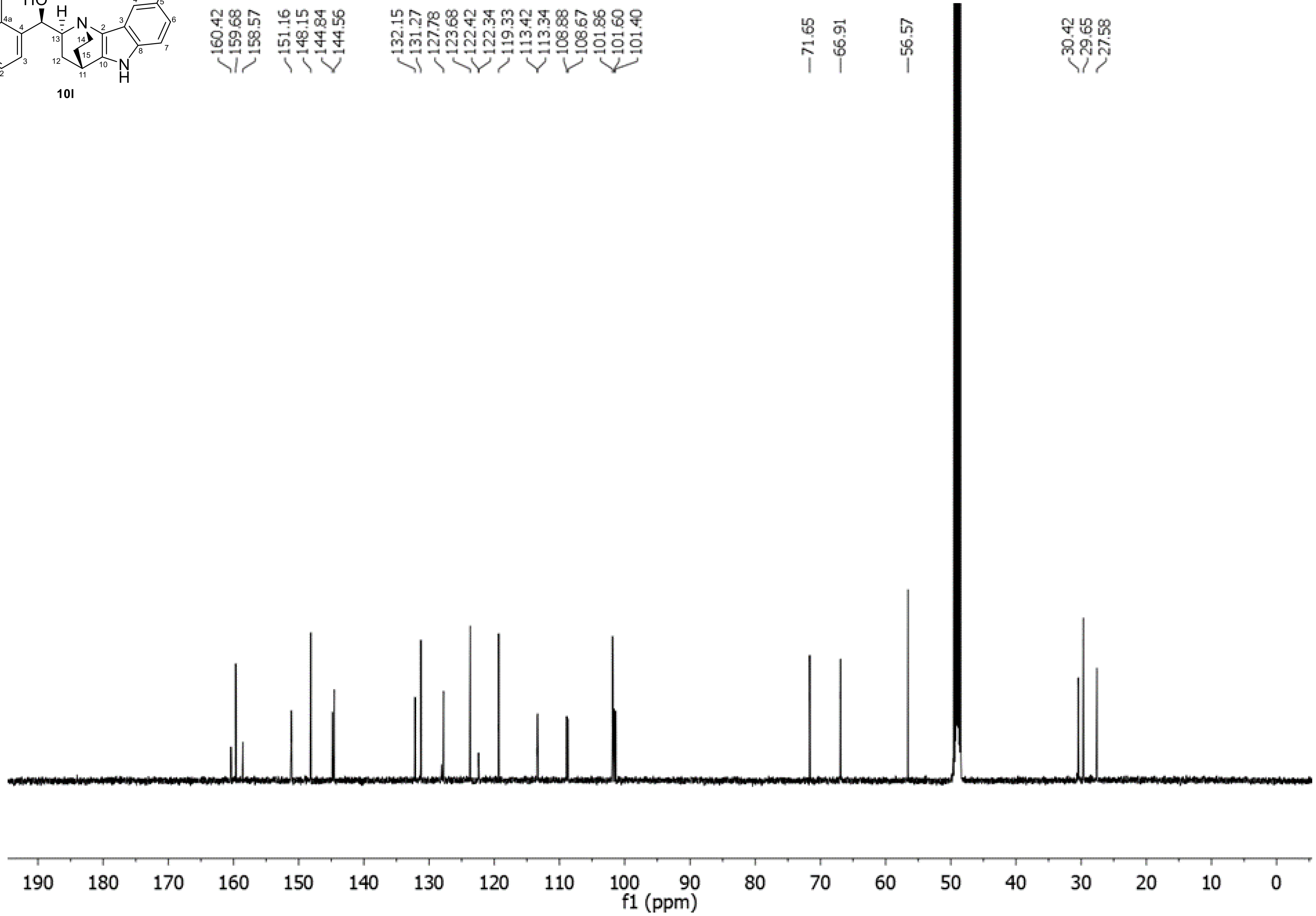
160.42
159.68
158.57
151.16
148.15
144.84
144.56
132.15
131.27
127.78
123.68
122.42
122.34
119.33
113.42
113.34
108.88
108.67
101.86
101.60
101.40
71.65
66.91
56.57
30.42
29.65
27.58
190 180 170 160 150 140 130 120 110 100 90 80 70 60 50 40 30 20 10 0
f1 (ppm)

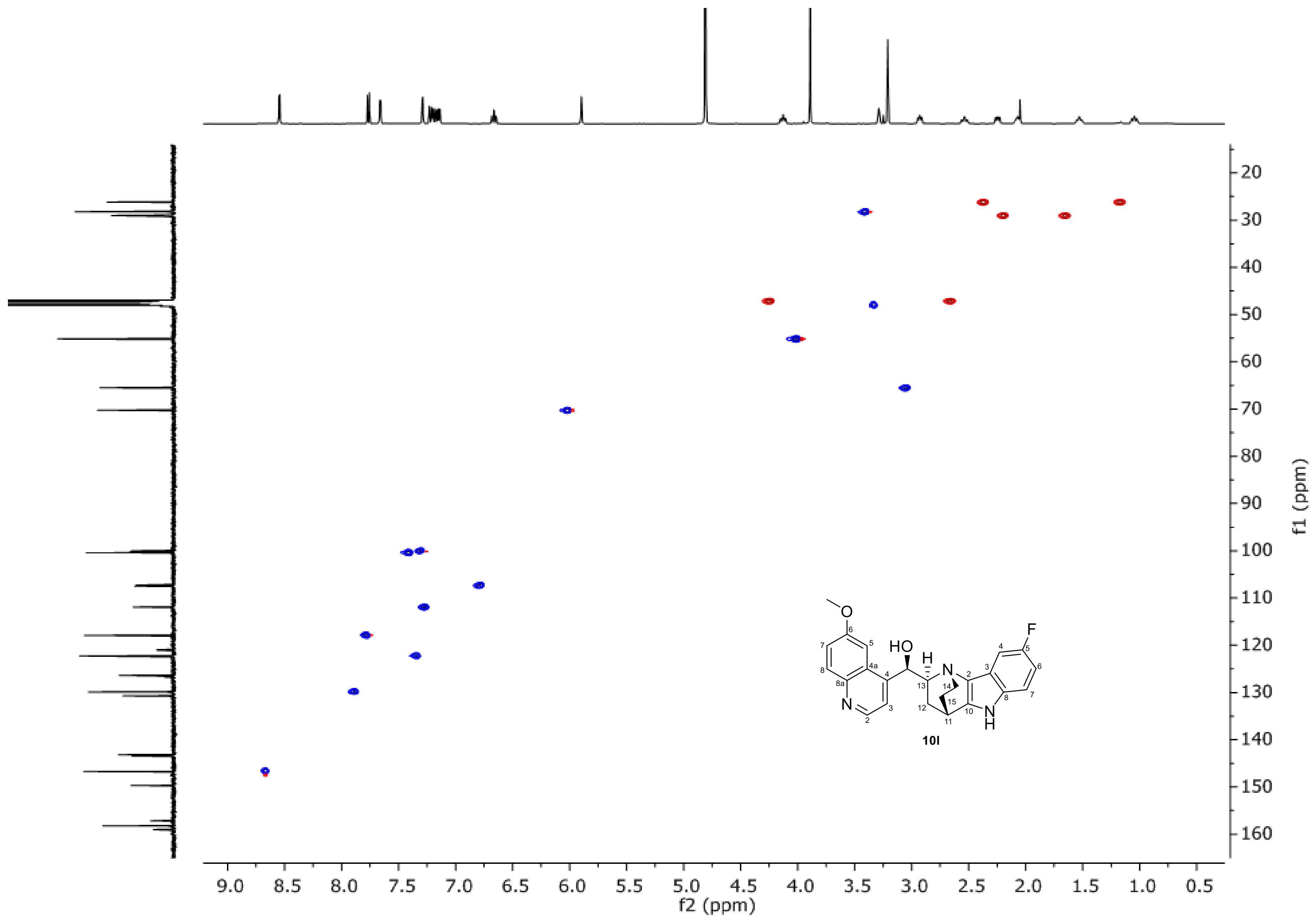
f2 (ppm)
f1 (ppm)
10l

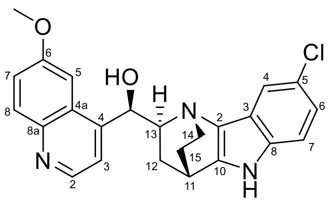
HO
H
N
Cl
N
H
OMe numbering: 2, 3, 4, 4a, 5, 6, 7, 8, 8a, 10, 11, 12, 13, 14, 15

10m

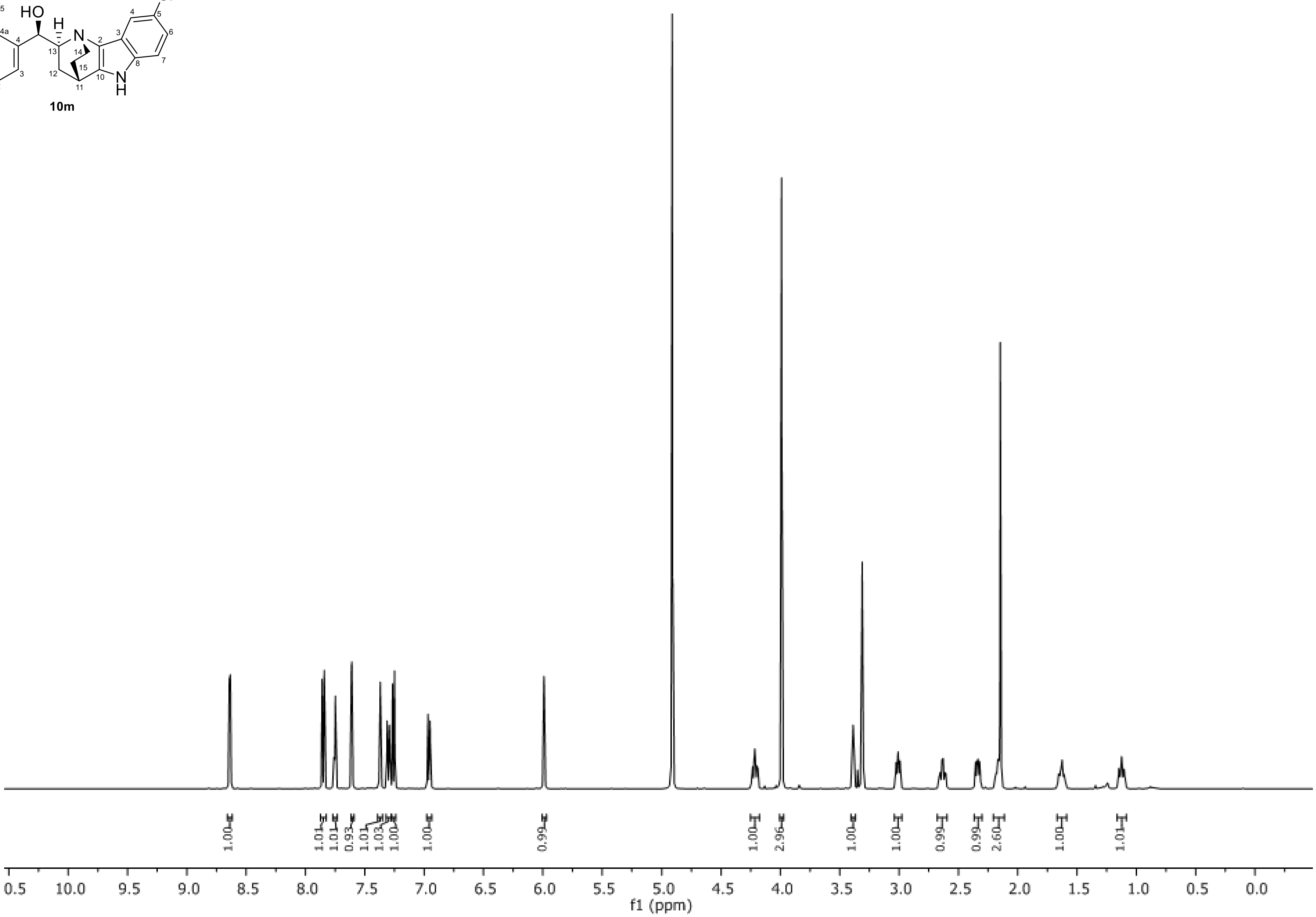
1.00
1.01
1.01
0.93
1.01
1.03
1.00
1.00
0.99
1.00
2.96
1.00
1.00
0.99
0.99
2.60
1.00
1.01
0.5 10.0 9.5 9.0 8.5 8.0 7.5 7.0 6.5 6.0 5.5 5.0 4.5 4.0 3.5 3.0 2.5 2.0 1.5 1.0 0.5 0.0
f1 (ppm)

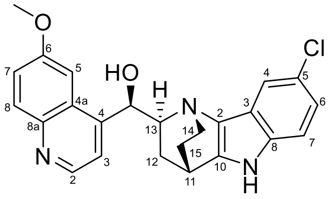

O
HO
H
N
Cl
N
H
N

10m

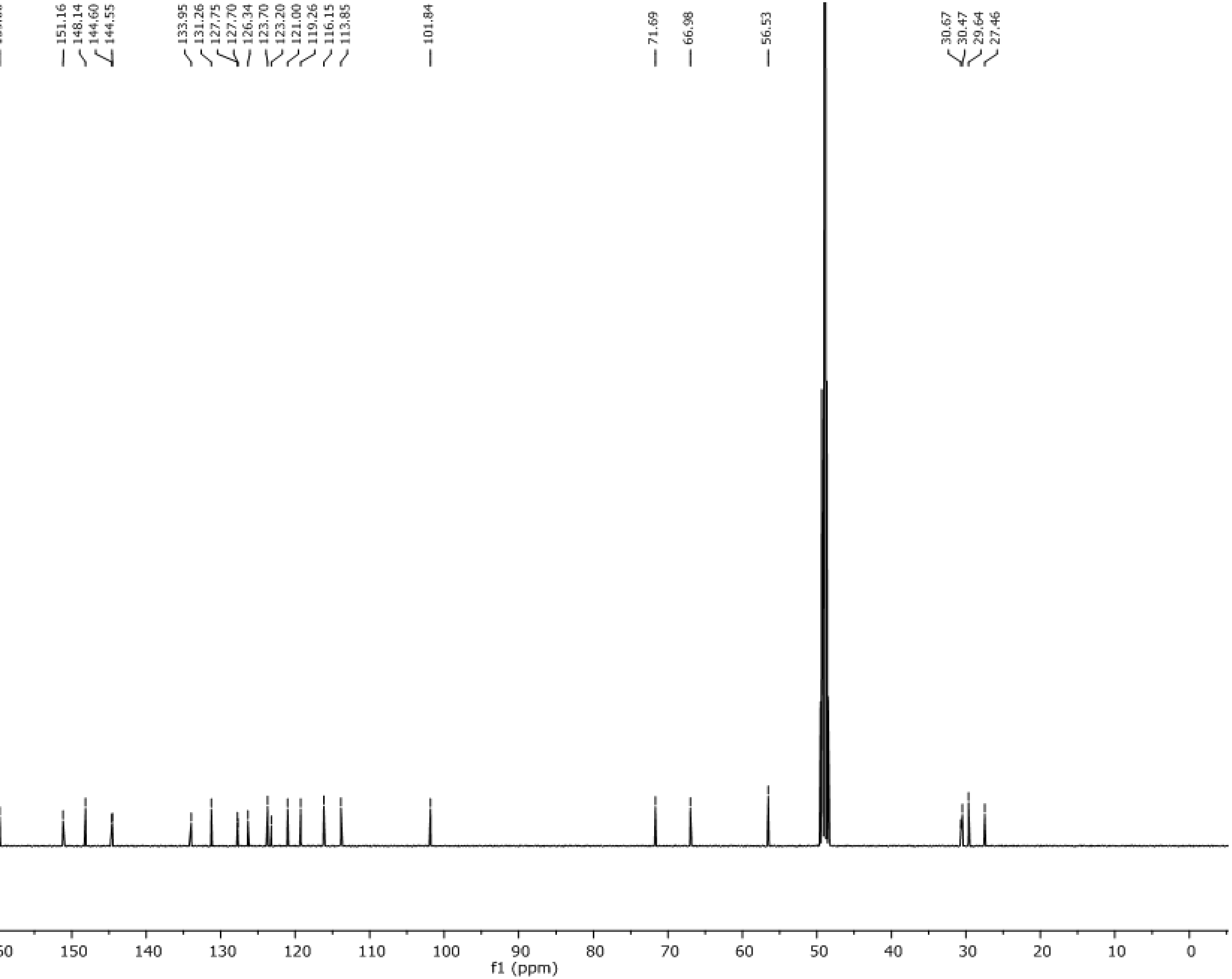

159.66
151.16
148.14
144.60
144.55
133.95
131.26
127.75
127.70
126.34
123.70
123.20
121.00
119.26
116.15
113.85
101.84
71.69
66.98
56.53
30.67
30.47
29.64
27.46
180 170 160 150 140 130 120 110 100 90 80 70 60 50 40 30 20 10 0
f1 (ppm)

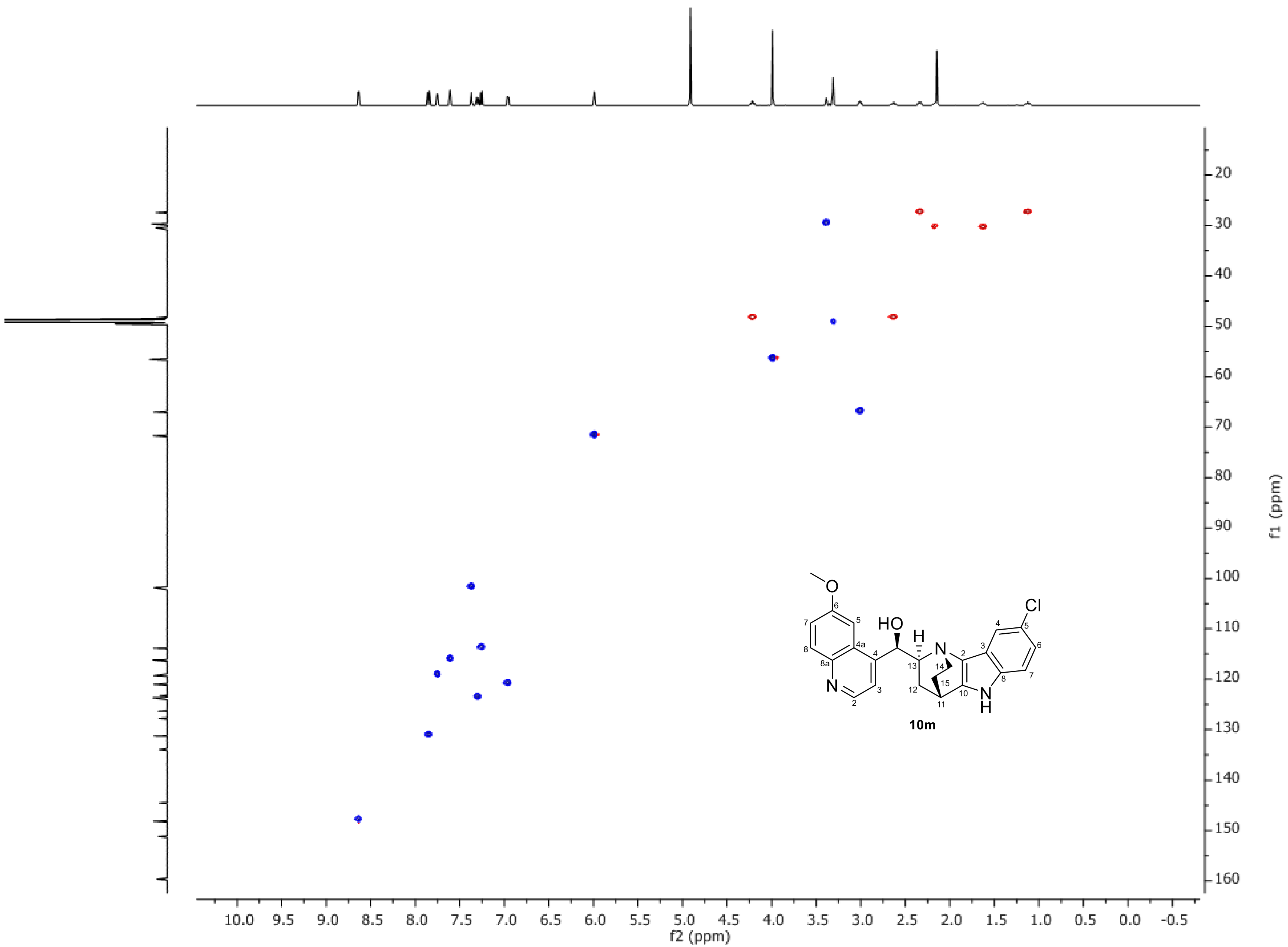
f2 (ppm)
f1 (ppm)
HO
H
N
Cl
N
H
10m

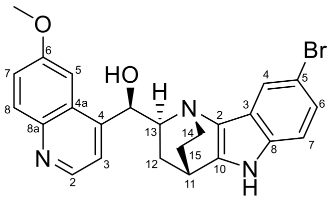
O
HO
Br
N
NH

10n

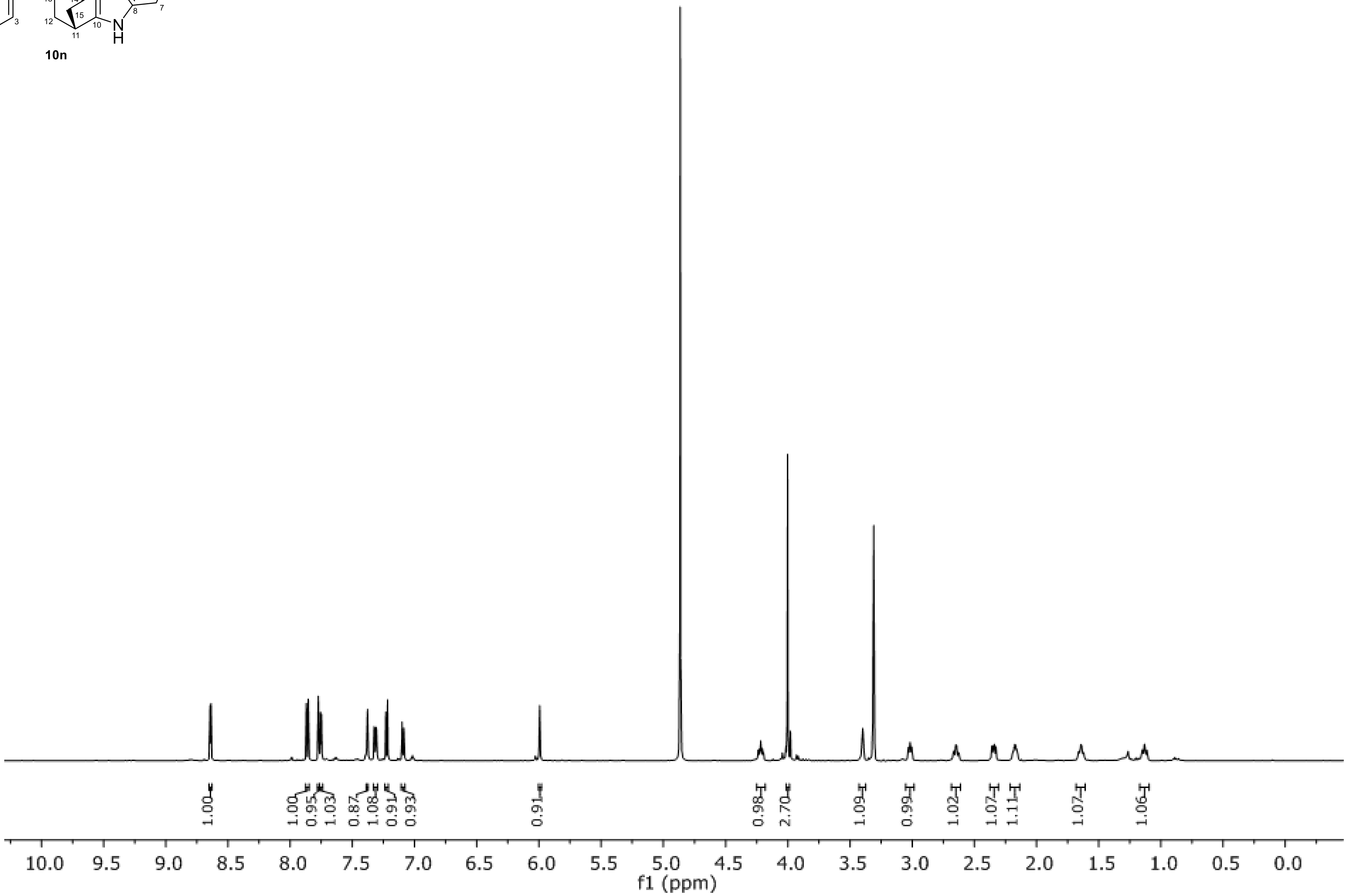
1.00
1.00
0.95
1.03
0.87
1.08
0.91
0.93
0.91
0.98
2.70
1.09
0.99
1.02
1.07
1.11
1.07
1.06
10.0 9.5 9.0 8.5 8.0 7.5 7.0 6.5 6.0 5.5 5.0 4.5 4.0 3.5 3.0 2.5 2.0 1.5 1.0 0.5 0.0
f1 (ppm)

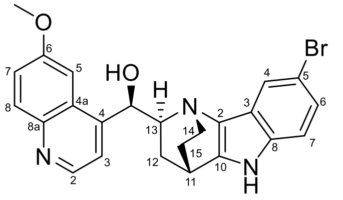
HO
H
N
Br
N
H

10n

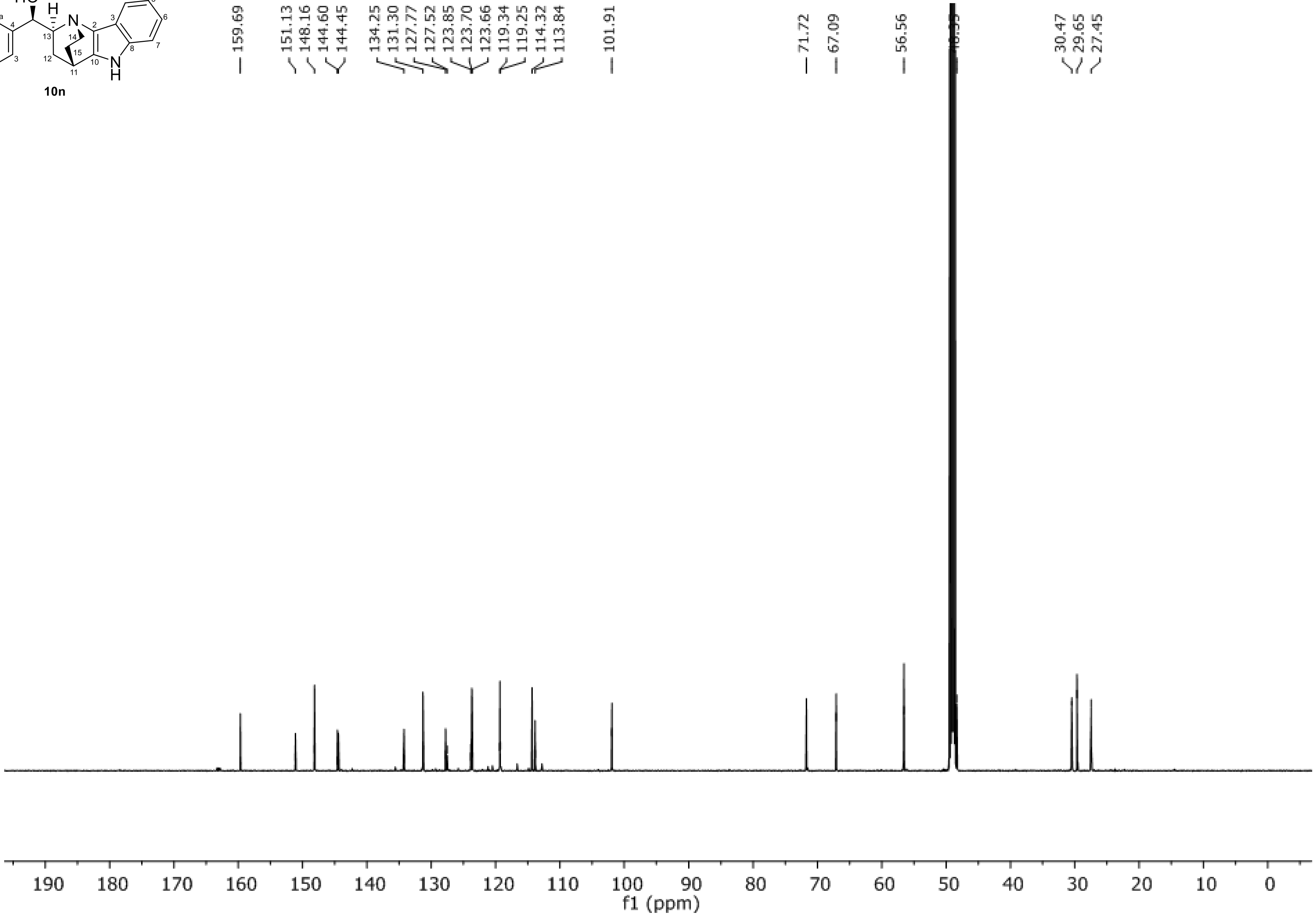
159.69
151.13
148.16
144.60
144.45
134.25
131.30
127.77
127.52
123.85
123.70
123.66
119.34
119.25
114.32
113.84
101.91
71.72
67.09
56.56
30.47
29.65
27.45
190 180 170 160 150 140 130 120 110 100 90 80 70 60 50 40 30 20 10 0
f1 (ppm)

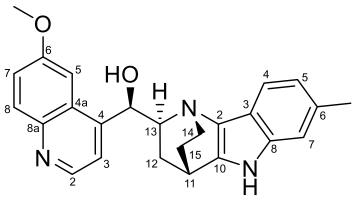

10o

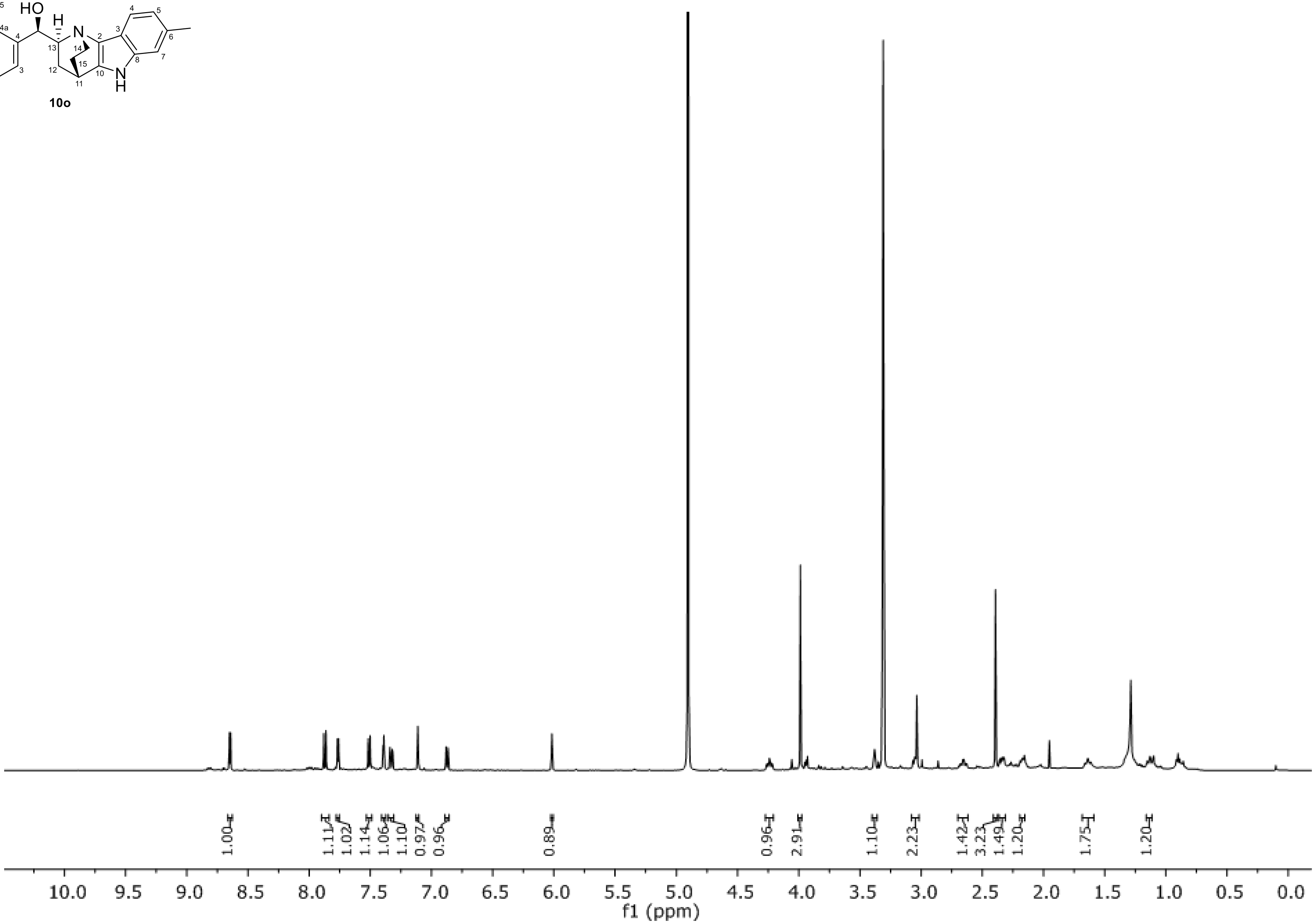
1.00
1.11
1.02
1.14
1.06
1.10
0.97
0.96
0.89
0.96
2.91
1.10
2.23
1.42
3.23
1.49
1.20
1.75
1.20
10.0 9.5 9.0 8.5 8.0 7.5 7.0 6.5 6.0 5.5 5.0 4.5 4.0 3.5 3.0 2.5 2.0 1.5 1.0 0.5 0.0
f1 (ppm)

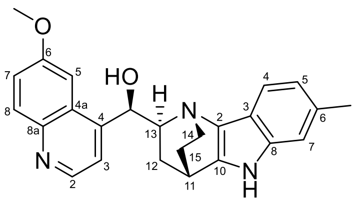

10o

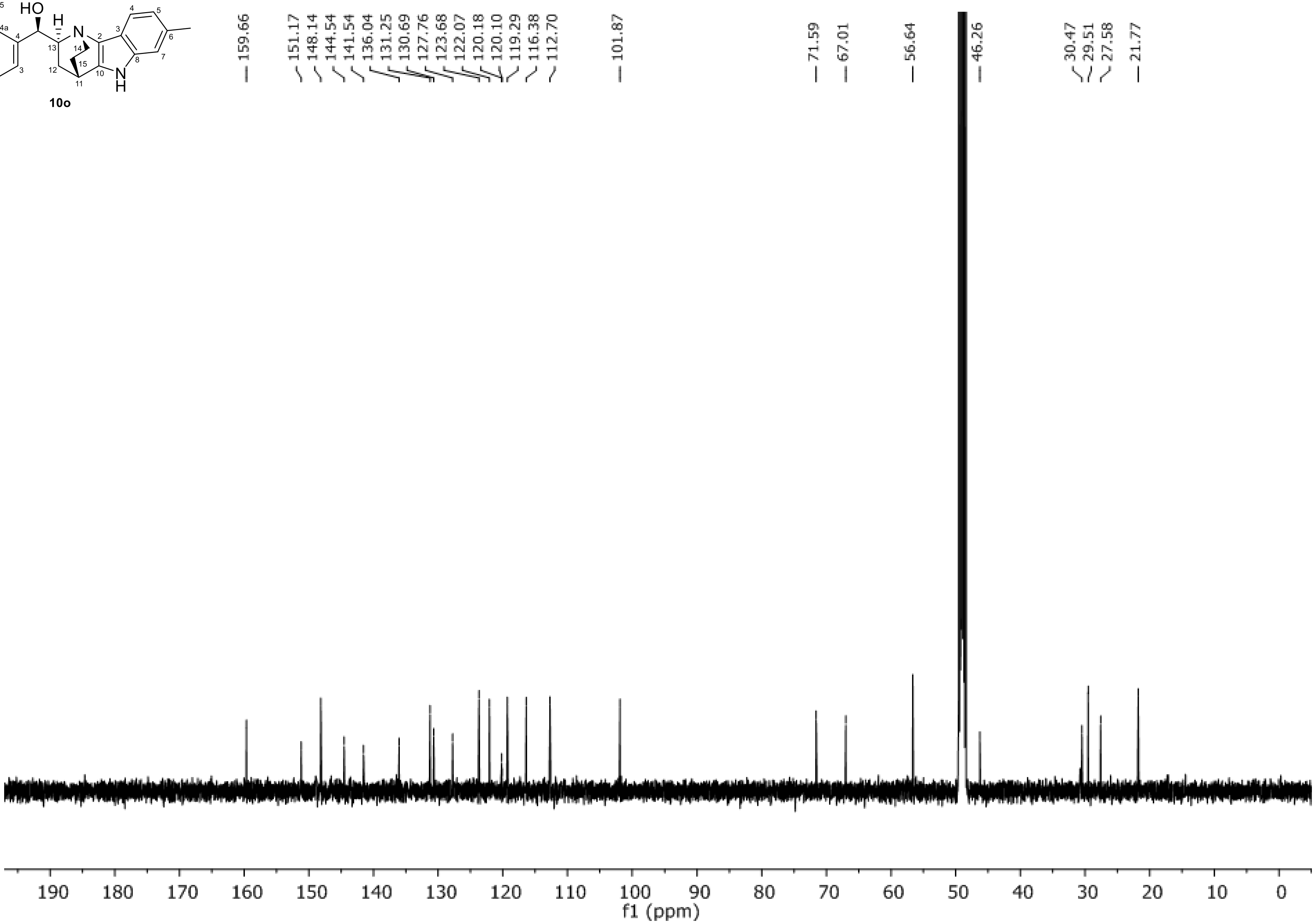
159.66
151.17
148.14
144.54
141.54
136.04
131.25
130.69
127.76
123.68
122.07
120.18
120.10
119.29
116.38
112.70
101.87
71.59
67.01
56.64
46.26
30.47
29.51
27.58
21.77
190 180 170 160 150 140 130 120 110 100 90 80 70 60 50 40 30 20 10 0
f1 (ppm)

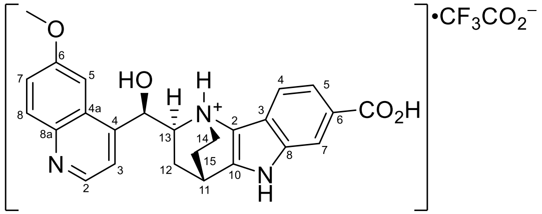

10p

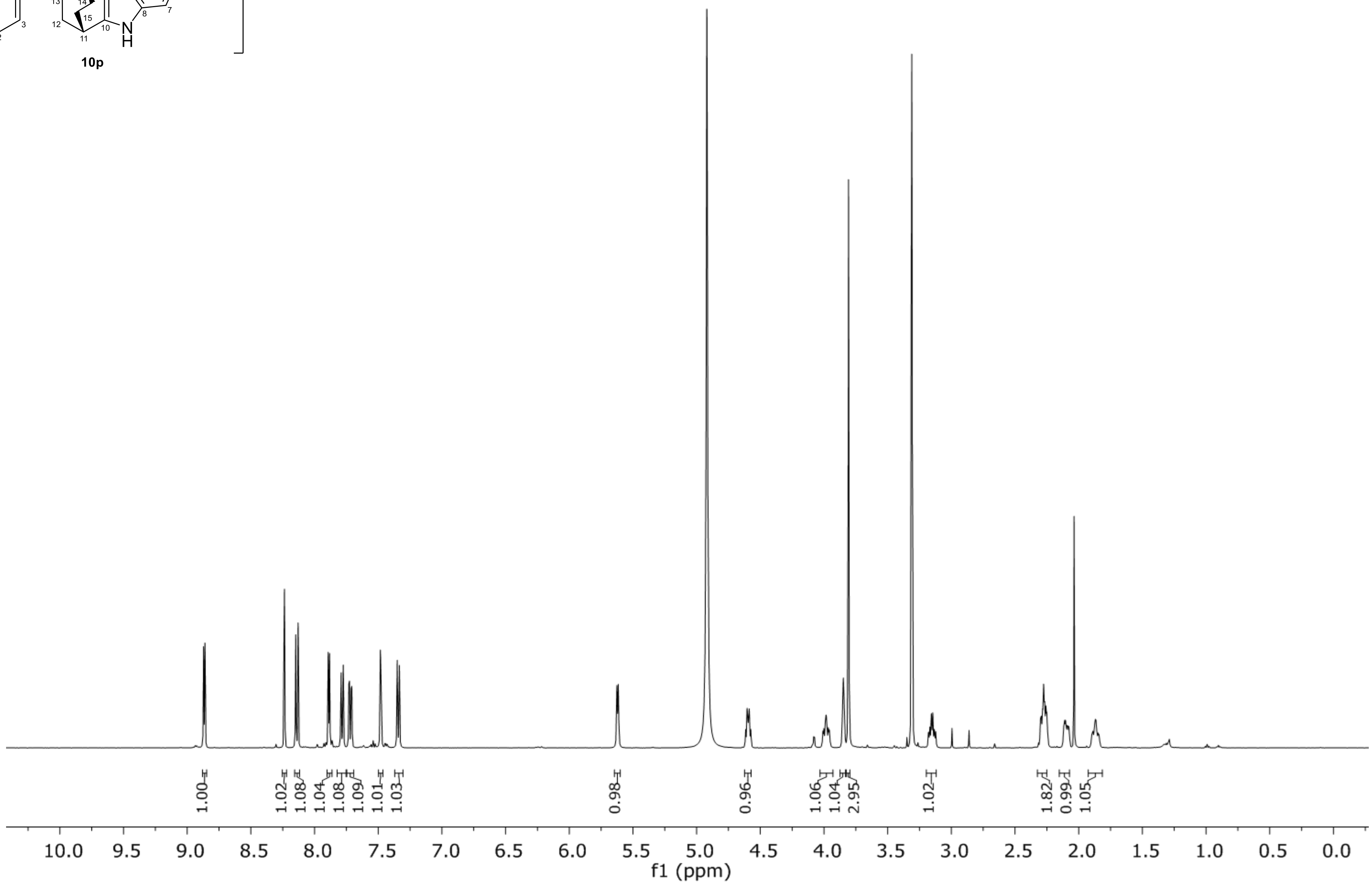

1.00
1.02
1.08
1.04
1.08
1.09
1.01
1.03
0.98
0.96
1.06
1.04
2.95
1.02
1.82
0.99
1.05
10.0 9.5 9.0 8.5 8.0 7.5 7.0 6.5 6.0 5.5 5.0 4.5 4.0 3.5 3.0 2.5 2.0 1.5 1.0 0.5 0.0
f1 (ppm)

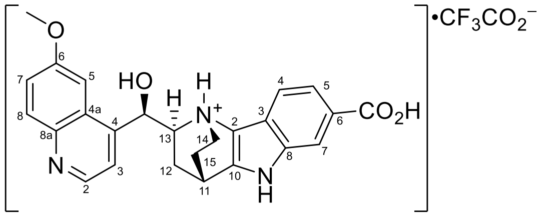

•$CF_3CO_2^-$
HO
H
N+
CO2H
N
NH

10p

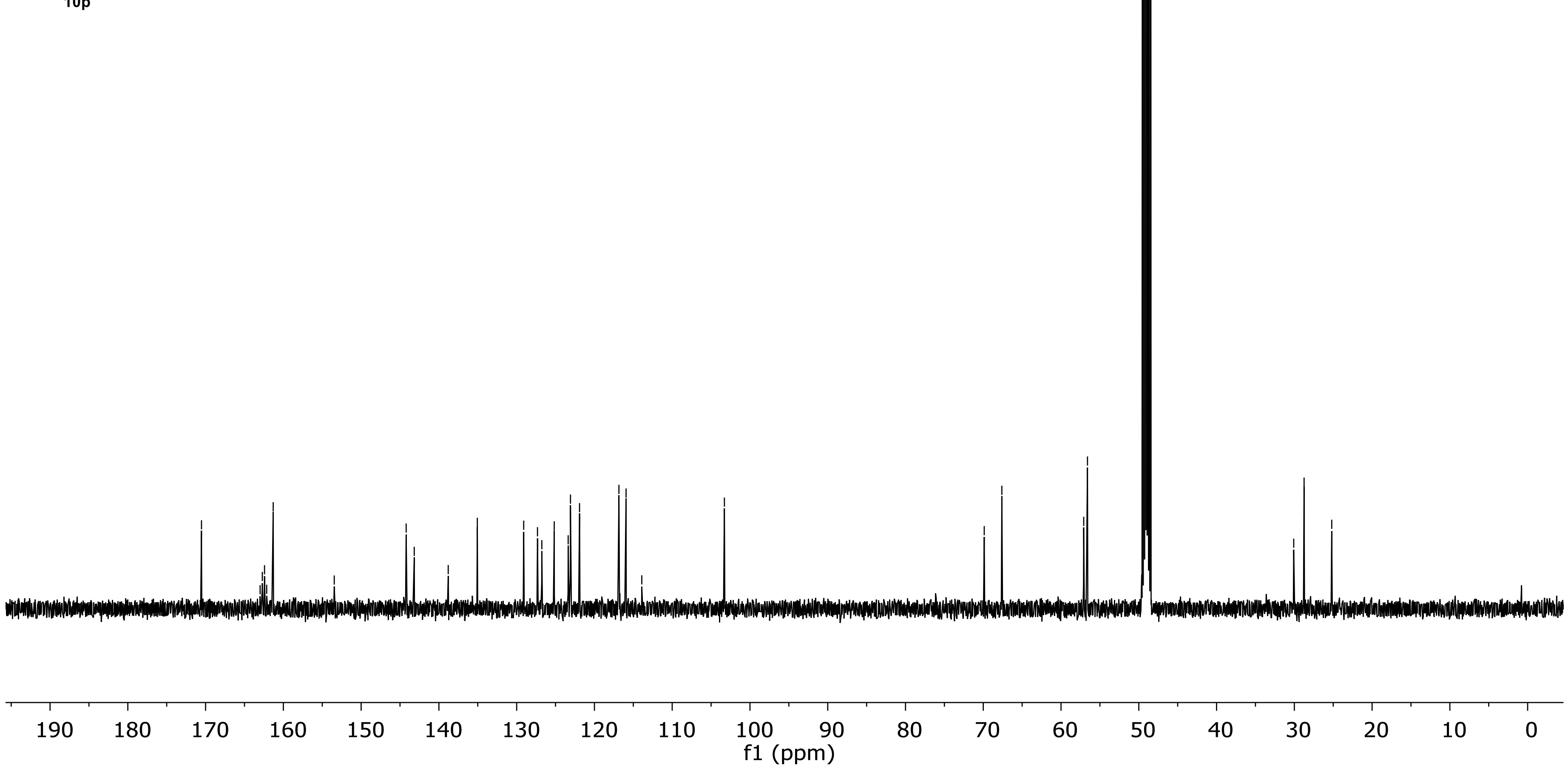

170.53
162.99
162.71
162.42
162.14
161.31
153.46
144.21
143.17
138.80
135.06
129.09
127.32
126.76
125.17
123.38
123.08
121.92
116.85
115.93
113.91
103.29
69.88
67.61
57.08
56.60
30.08
28.75
25.19
190 180 170 160 150 140 130 120 110 100 90 80 70 60 50 40 30 20 10 0
f1 (ppm)

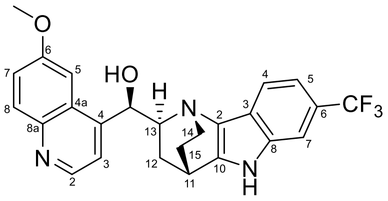

10r

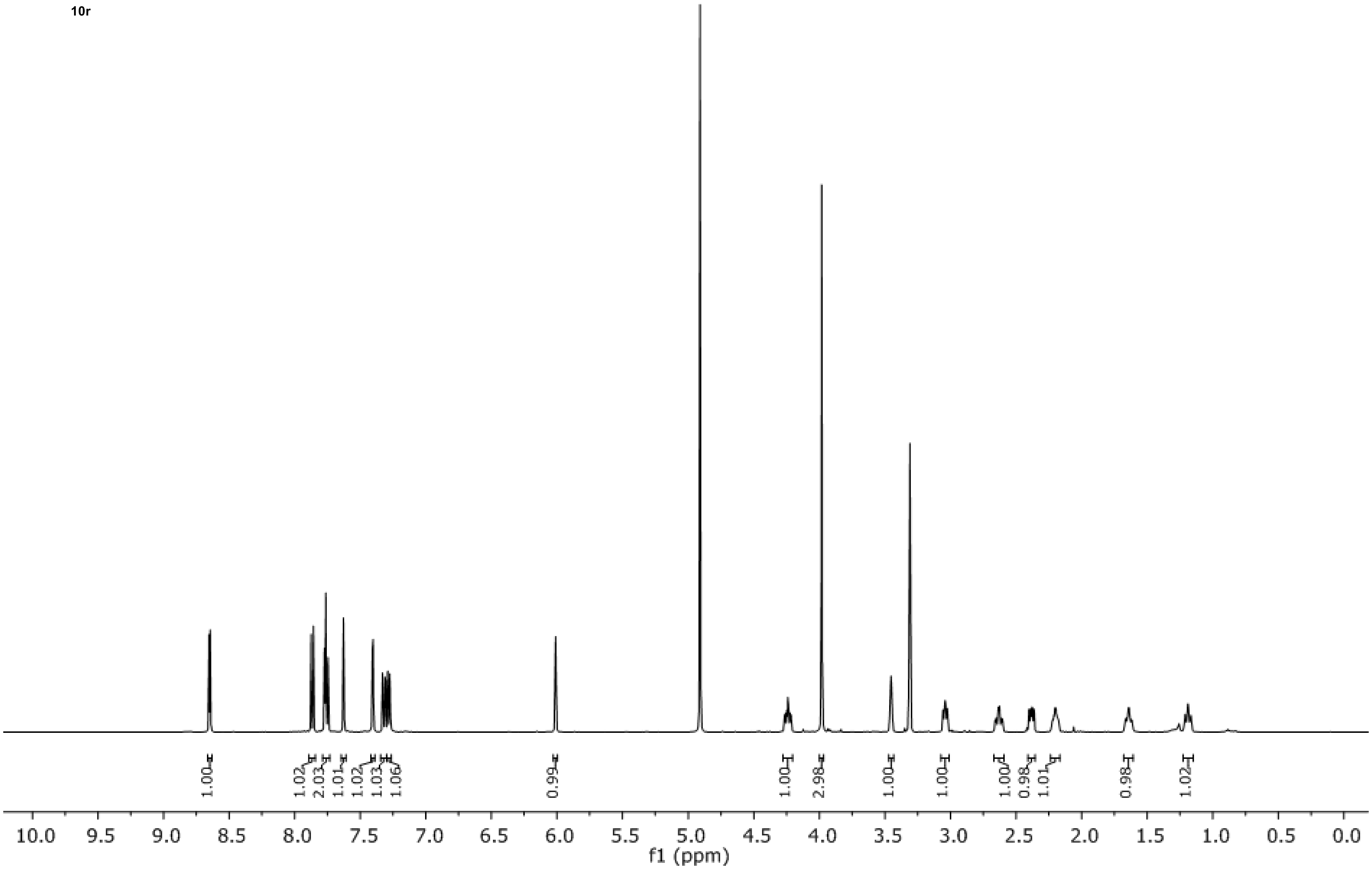
1.00
1.02
2.03
1.01
1.02
1.03
1.06
0.99
1.00
2.98
1.00
1.00
1.00
0.98
1.01
0.98
1.02
10.0 9.5 9.0 8.5 8.0 7.5 7.0 6.5 6.0 5.5 5.0 4.5 4.0 3.5 3.0 2.5 2.0 1.5 1.0 0.5 0.0
f1 (ppm)

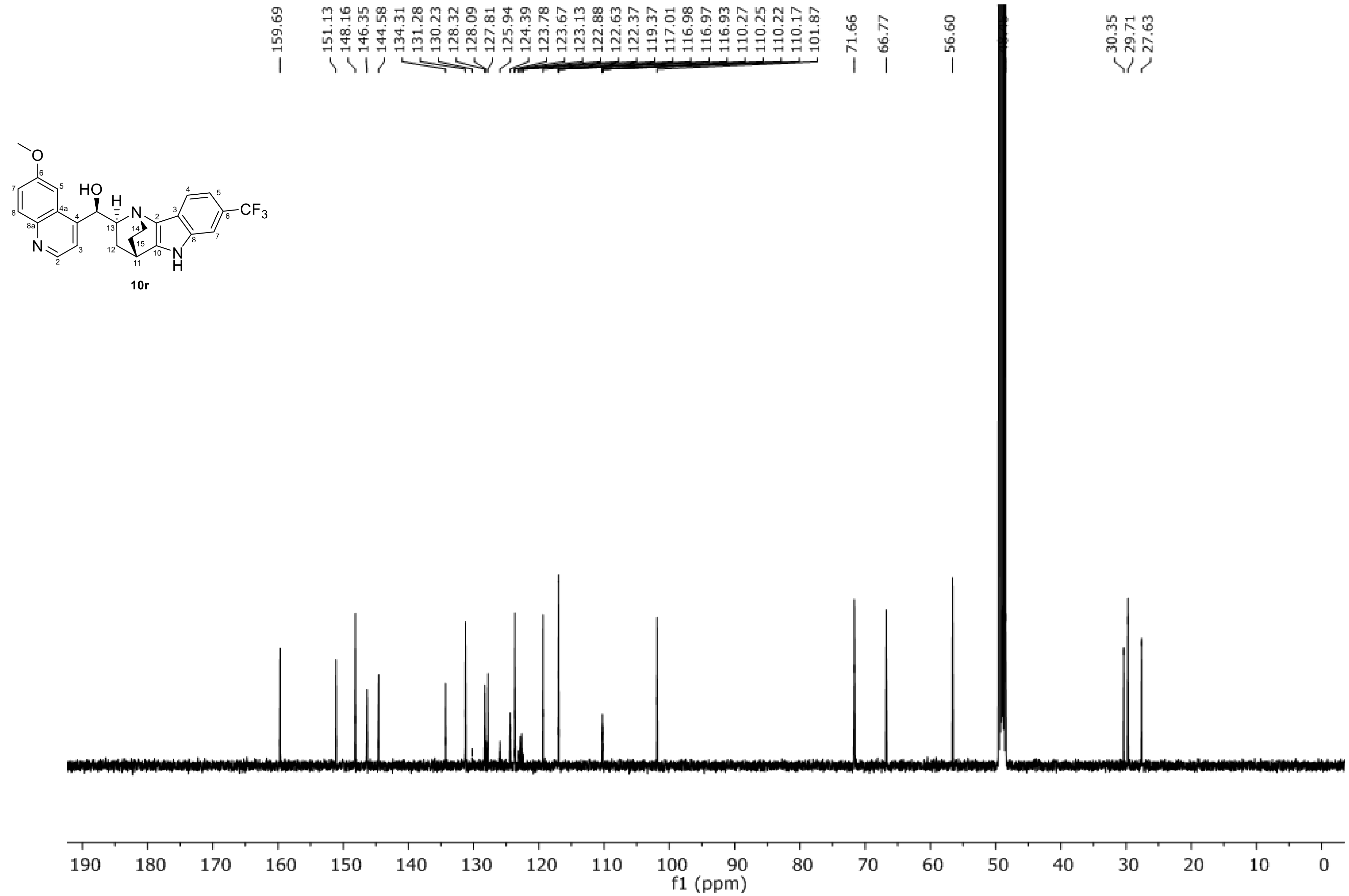

159.69
151.13
148.16
146.35
144.58
134.31
131.28
130.23
128.32
128.09
127.81
125.94
124.39
123.78
123.67
123.13
122.88
122.63
122.37
119.37
117.01
116.98
116.97
116.93
110.27
110.25
110.22
110.17
101.87
71.66
66.77
56.60
30.35
29.71
27.63
HO
H
N
CF3
NH
10r
f1 (ppm)

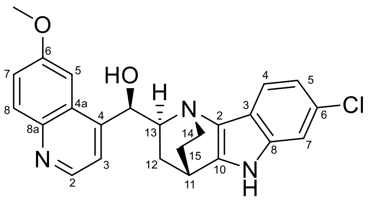
O
6
7
5
HO
H
N
4a
4
13
14
2
3
4
5
6
Cl
8
8a
N
2
3
12
15
11
10
8
7
N
H

10s

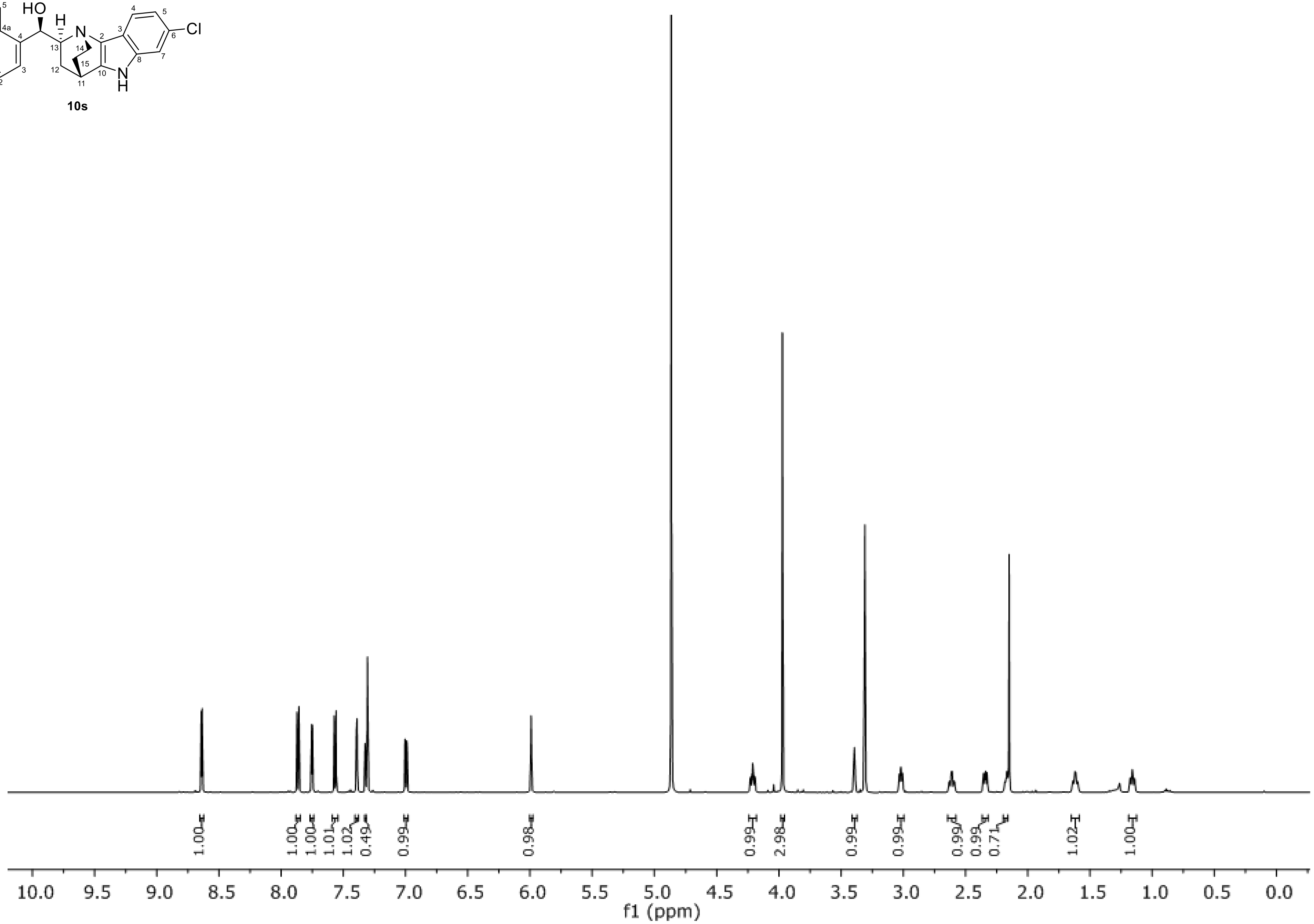
1.00
1.00
1.00
1.01
1.02
0.49
0.99
0.98
0.99
2.98
0.99
0.99
0.99
0.99
0.71
1.02
1.00
10.0 9.5 9.0 8.5 8.0 7.5 7.0 6.5 6.0 5.5 5.0 4.5 4.0 3.5 3.0 2.5 2.0 1.5 1.0 0.5 0.0
f1 (ppm)

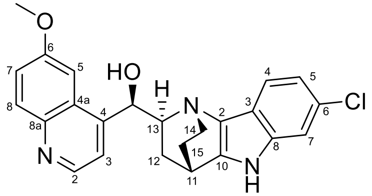

10s

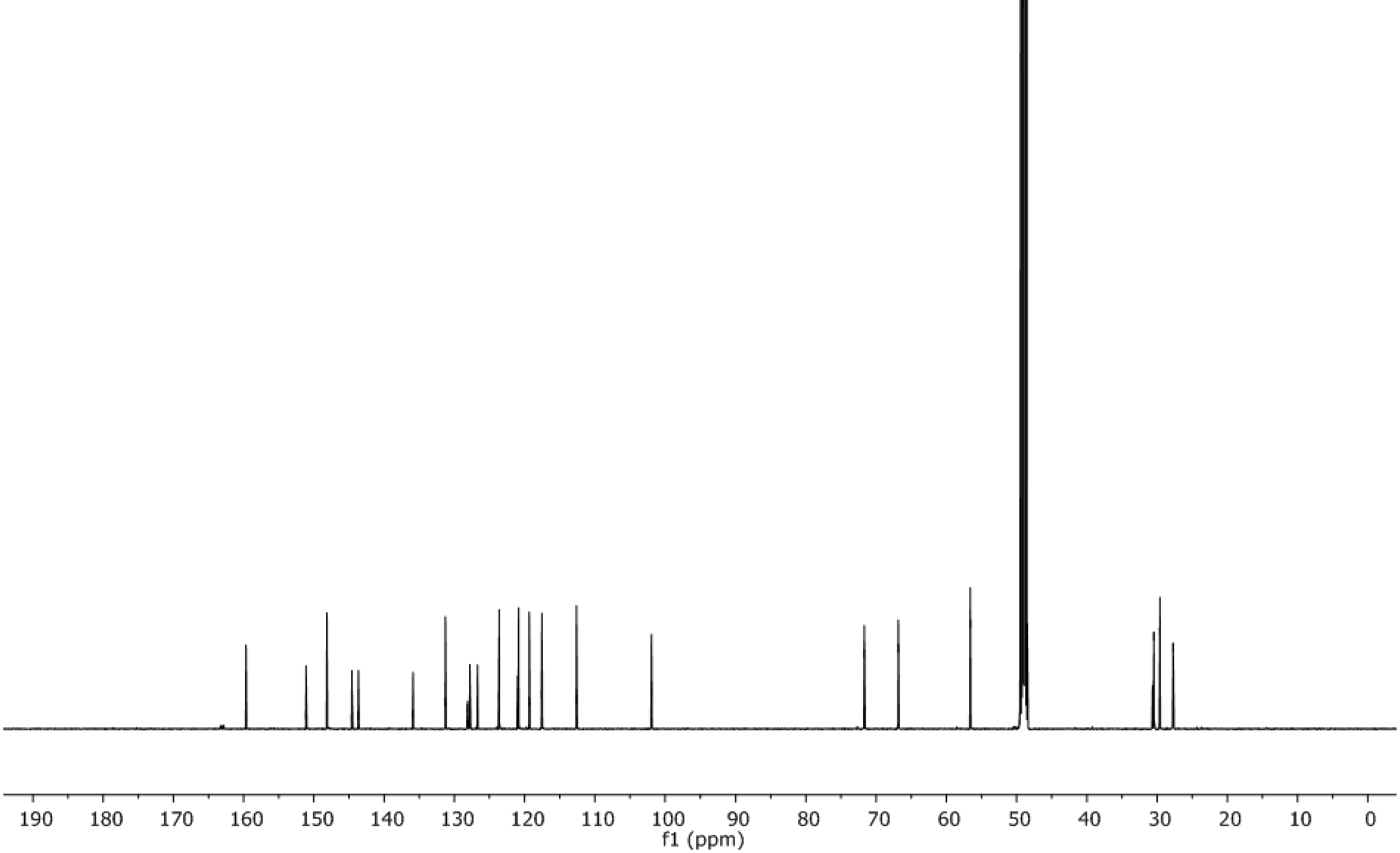
159.67
151.14
148.16
144.60
143.68
135.90
131.28
128.13
127.81
126.71
123.63
121.01
120.86
119.35
117.55
112.62
101.95
71.69
66.86
56.60
30.46
29.60
27.72
190 180 170 160 150 140 130 120 110 100 90 80 70 60 50 40 30 20 10 0
f1 (ppm)

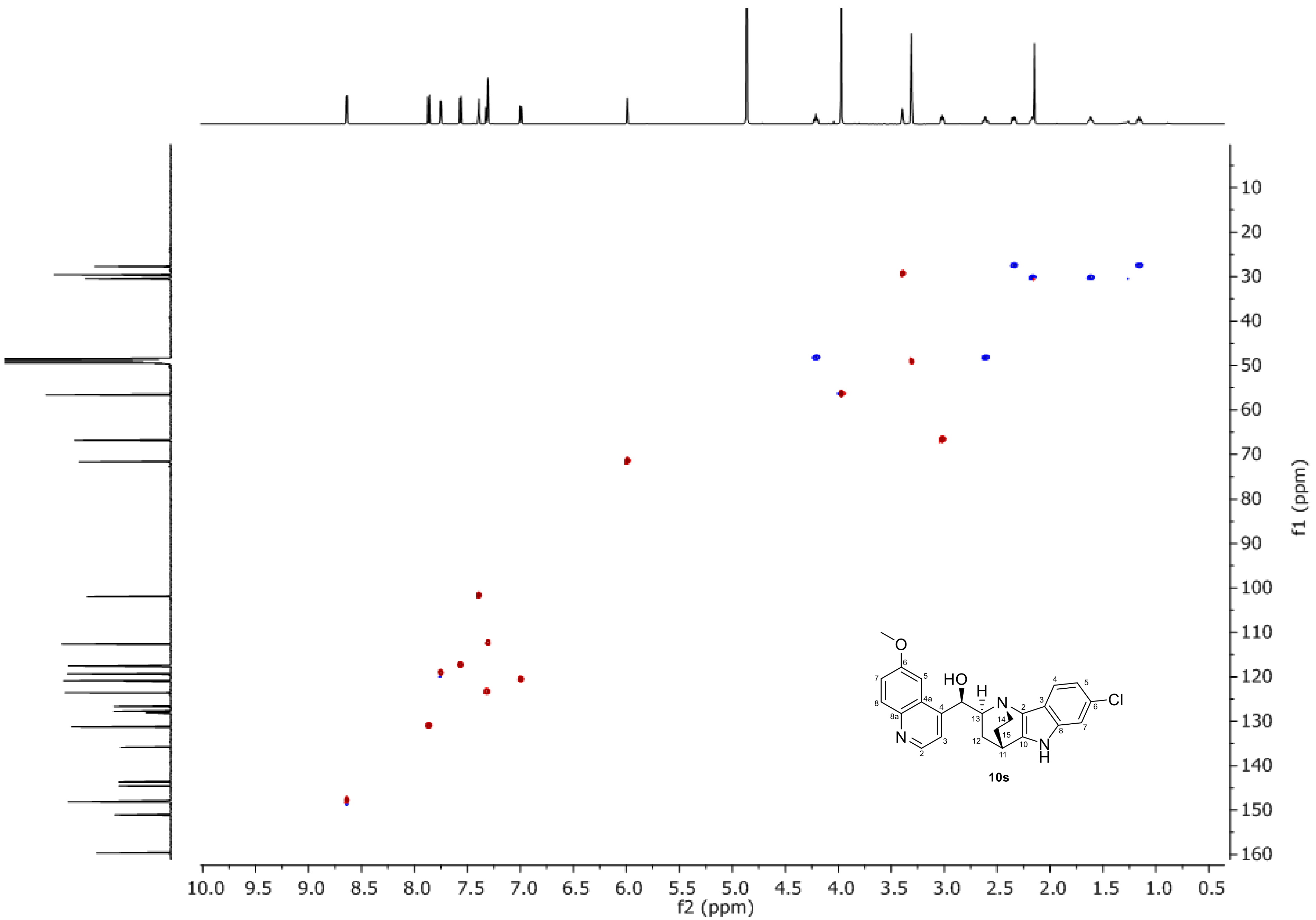
HO
H
N
Cl
N
H
O
10s
f2 (ppm)
f1 (ppm)

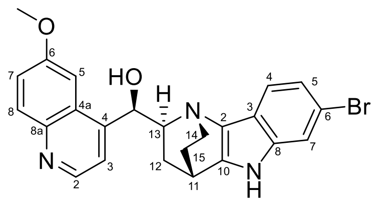

10t

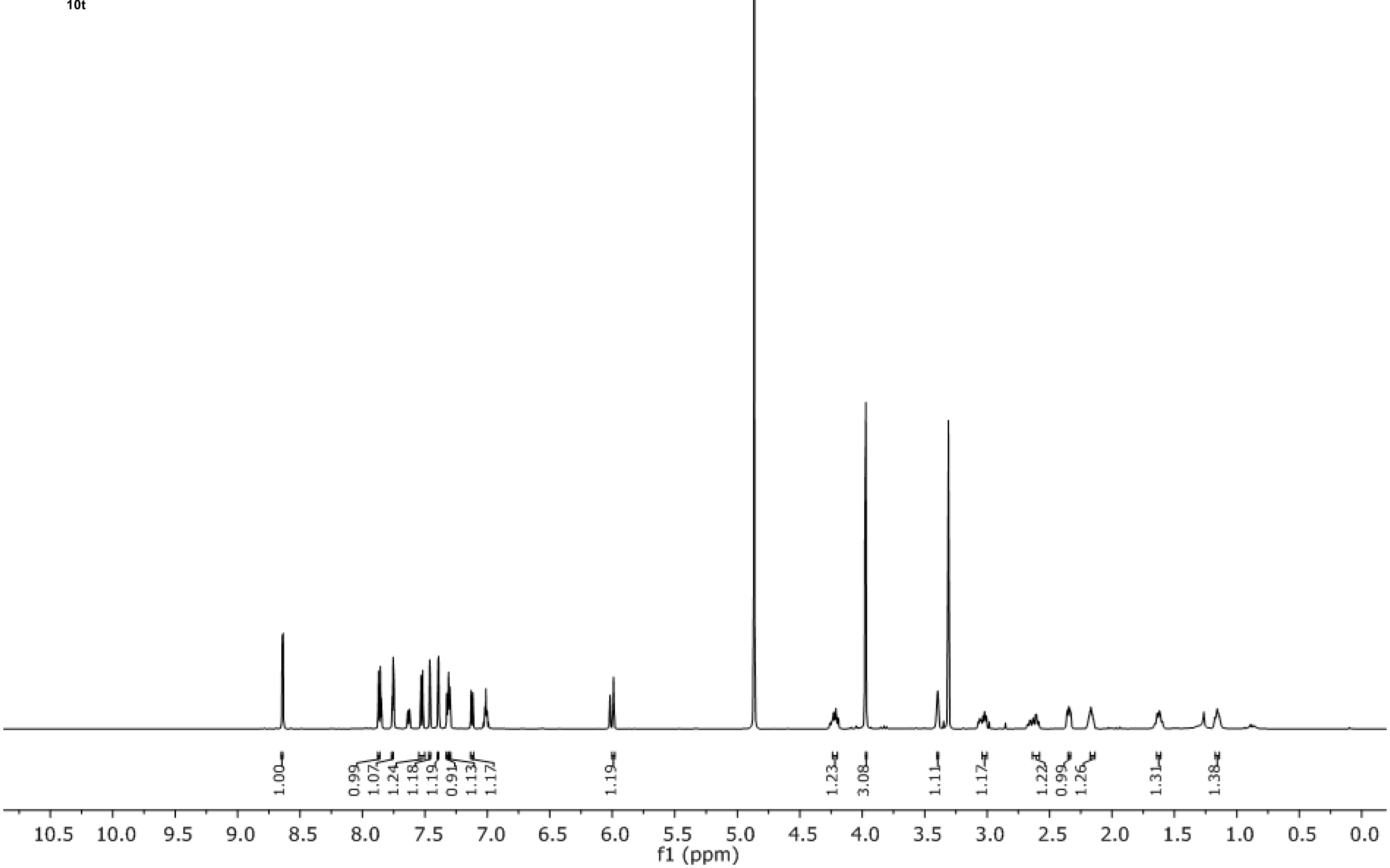
1.00
0.99
1.07
1.24
1.18
1.19
0.91
1.13
1.17
1.19
1.23
3.08
1.11
1.17
1.22
0.99
1.26
1.31
1.38
10.5
10.0
9.5
9.0
8.5
8.0
7.5
7.0
6.5
6.0
5.5
5.0
4.5
4.0
3.5
3.0
2.5
2.0
1.5
1.0
0.5
0.0
f1 (ppm)

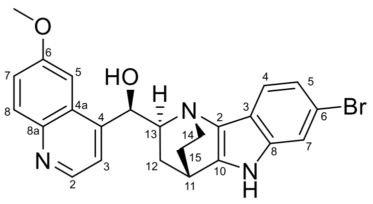
HO
N
Br
N
H

10t

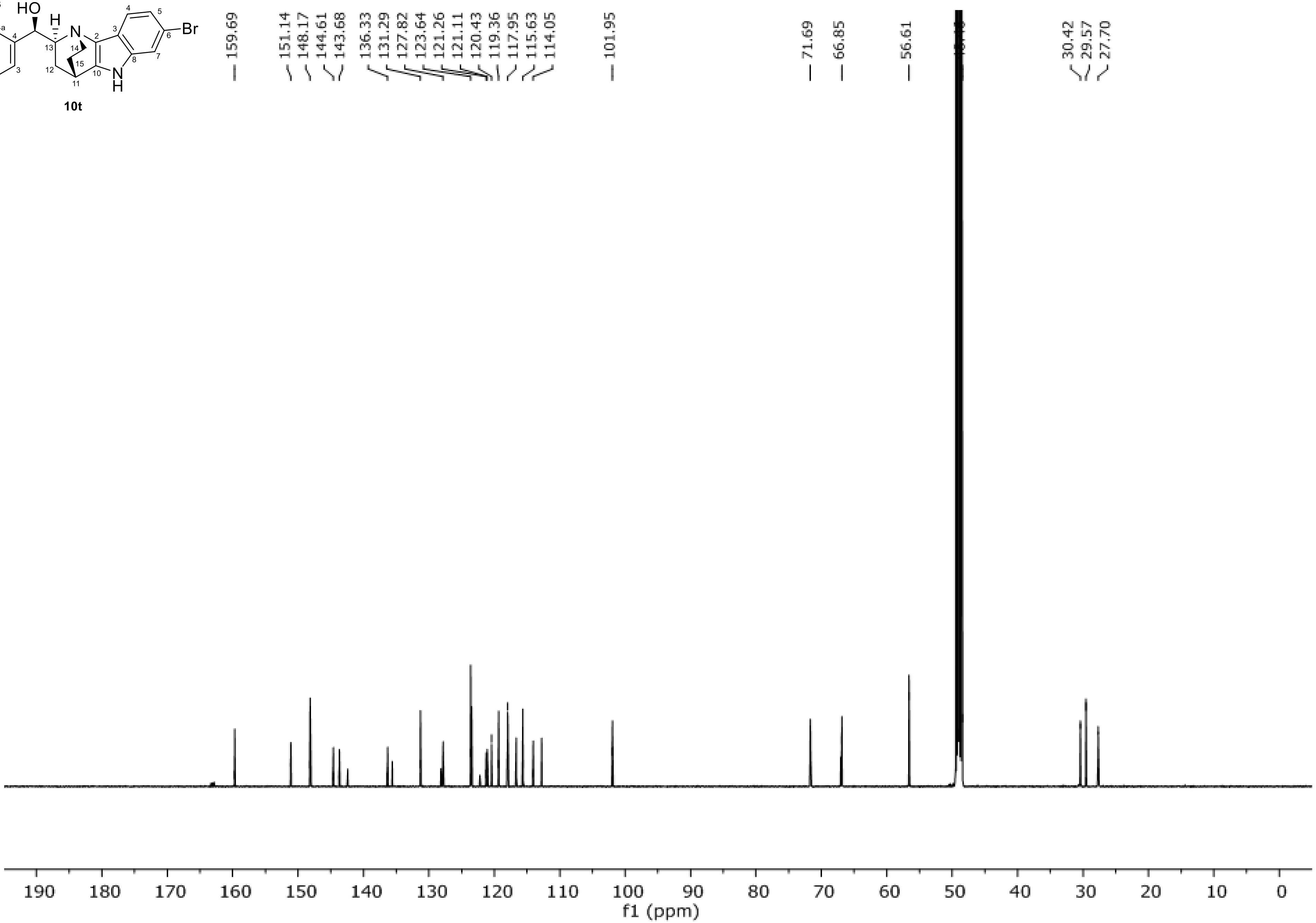
159.69
151.14
148.17
144.61
143.68
136.33
131.29
127.82
123.64
121.26
121.11
120.43
119.36
117.95
115.63
114.05
101.95
71.69
66.85
56.61
30.42
29.57
27.70
190 180 170 160 150 140 130 120 110 100 90 80 70 60 50 40 30 20 10 0
f1 (ppm)

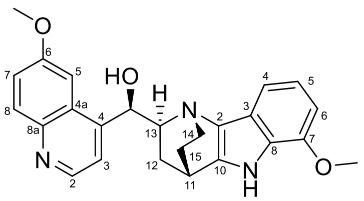

10u

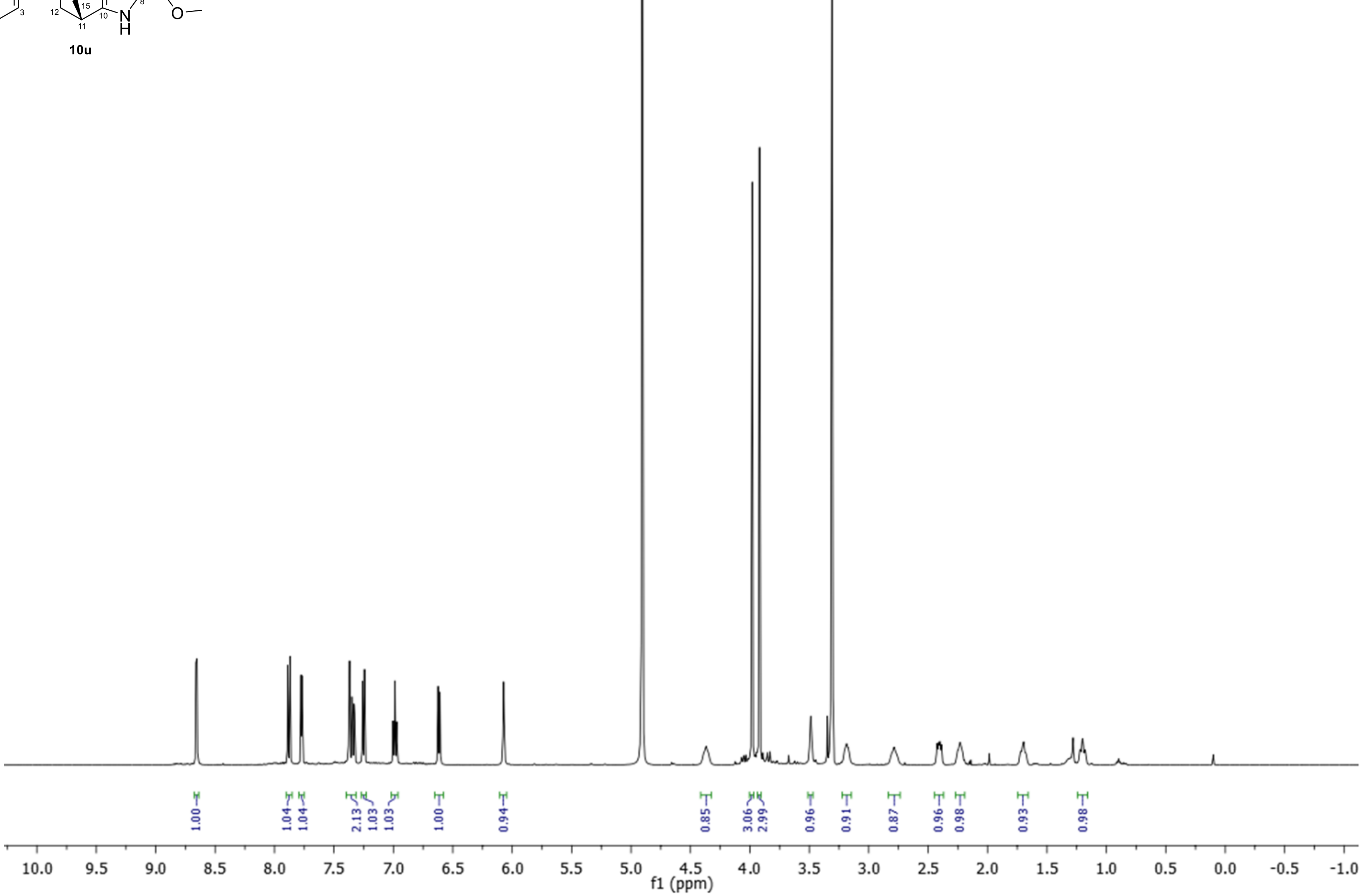
1.00
1.04
1.04
2.13
1.03
1.03
1.00
0.94
0.85
3.06
2.99
0.96
0.91
0.87
0.96
0.98
0.93
0.98
10.0 9.5 9.0 8.5 8.0 7.5 7.0 6.5 6.0 5.5 5.0 4.5 4.0 3.5 3.0 2.5 2.0 1.5 1.0 0.5 0.0 -0.5 -1.0
f1 (ppm)

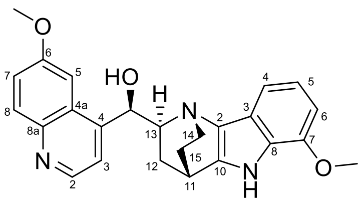

10u

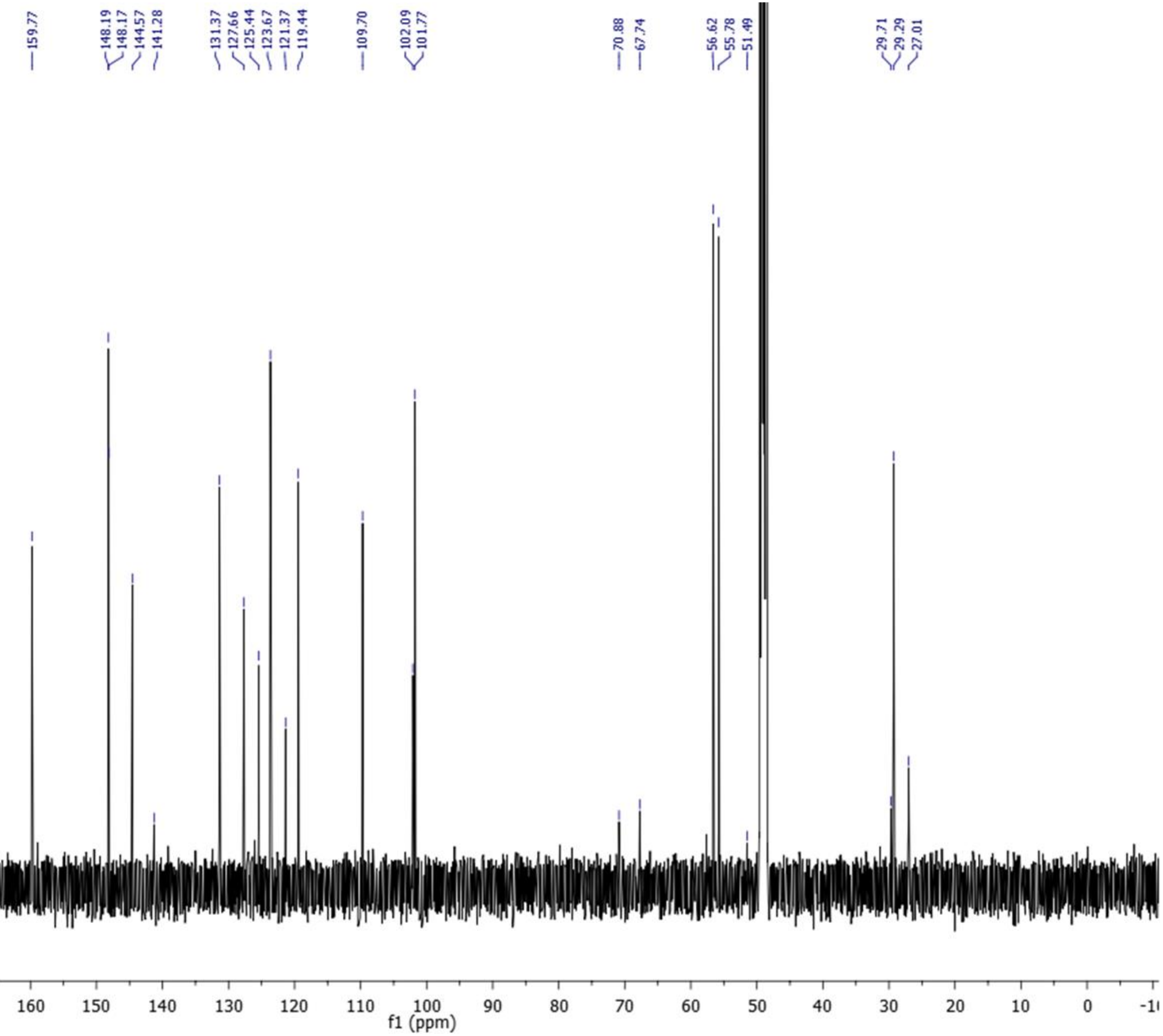
159.77
148.19
148.17
144.57
141.28
131.37
127.66
125.44
123.67
121.37
119.44
109.70
102.09
101.77
70.88
67.74
56.62
55.78
51.49
29.71
29.29
27.01
210
200
190
180
170
160
150
140
130
120
110
100
90
80
70
60
50
40
30
20
10
0
f1 (ppm)

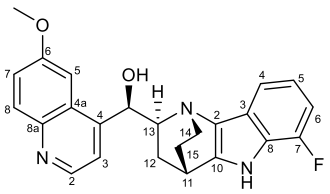

O
OH
H
N
NH
F

10x

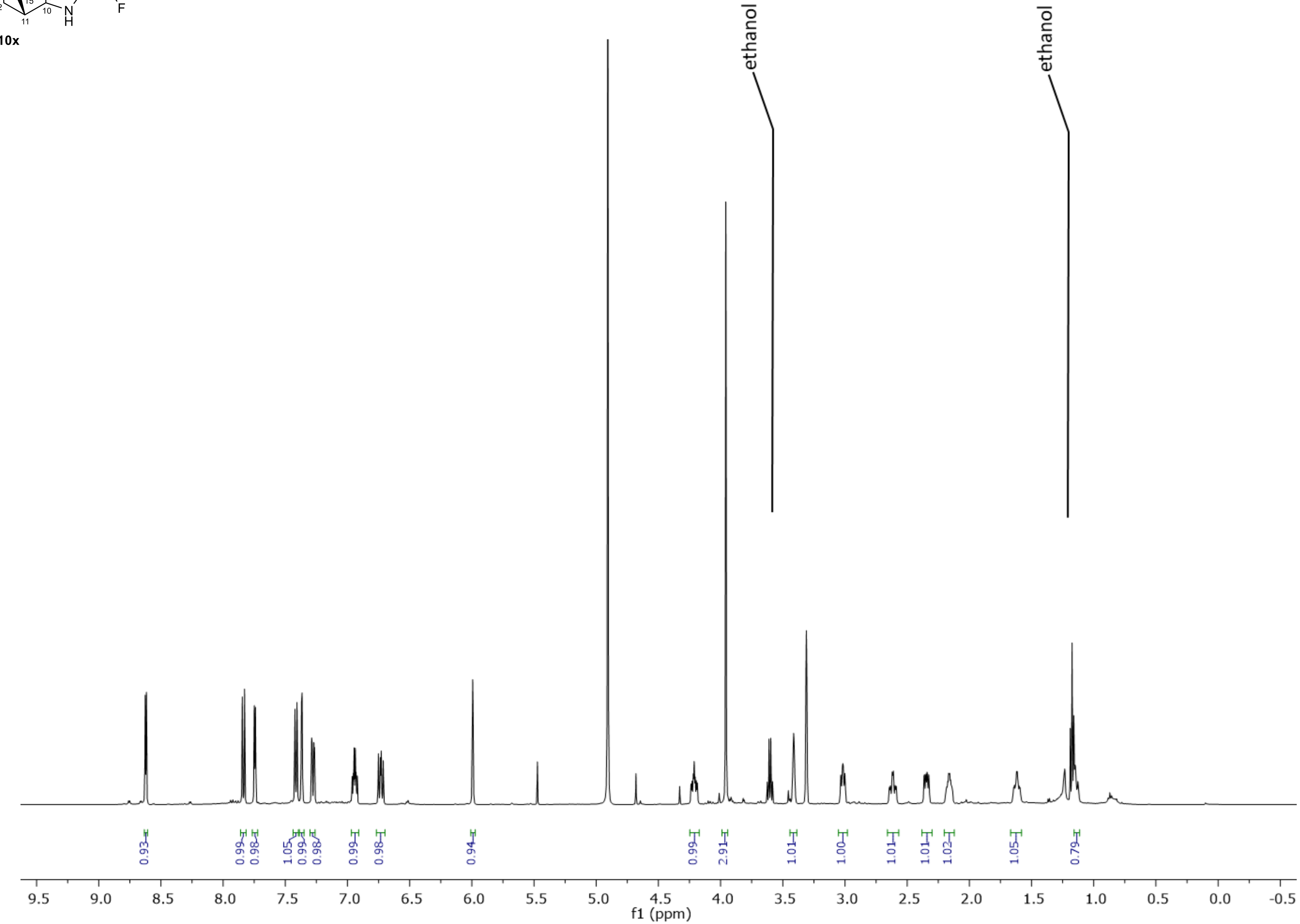

ethanol
ethanol
0.93
0.99
0.98
1.05
0.99
0.98
0.99
0.98
0.94
0.99
2.91
1.01
1.00
1.01
1.01
1.02
1.05
0.79
9.5 9.0 8.5 8.0 7.5 7.0 6.5 6.0 5.5 5.0 4.5 4.0 3.5 3.0 2.5 2.0 1.5 1.0 0.5 0.0 -0.5
f1 (ppm)

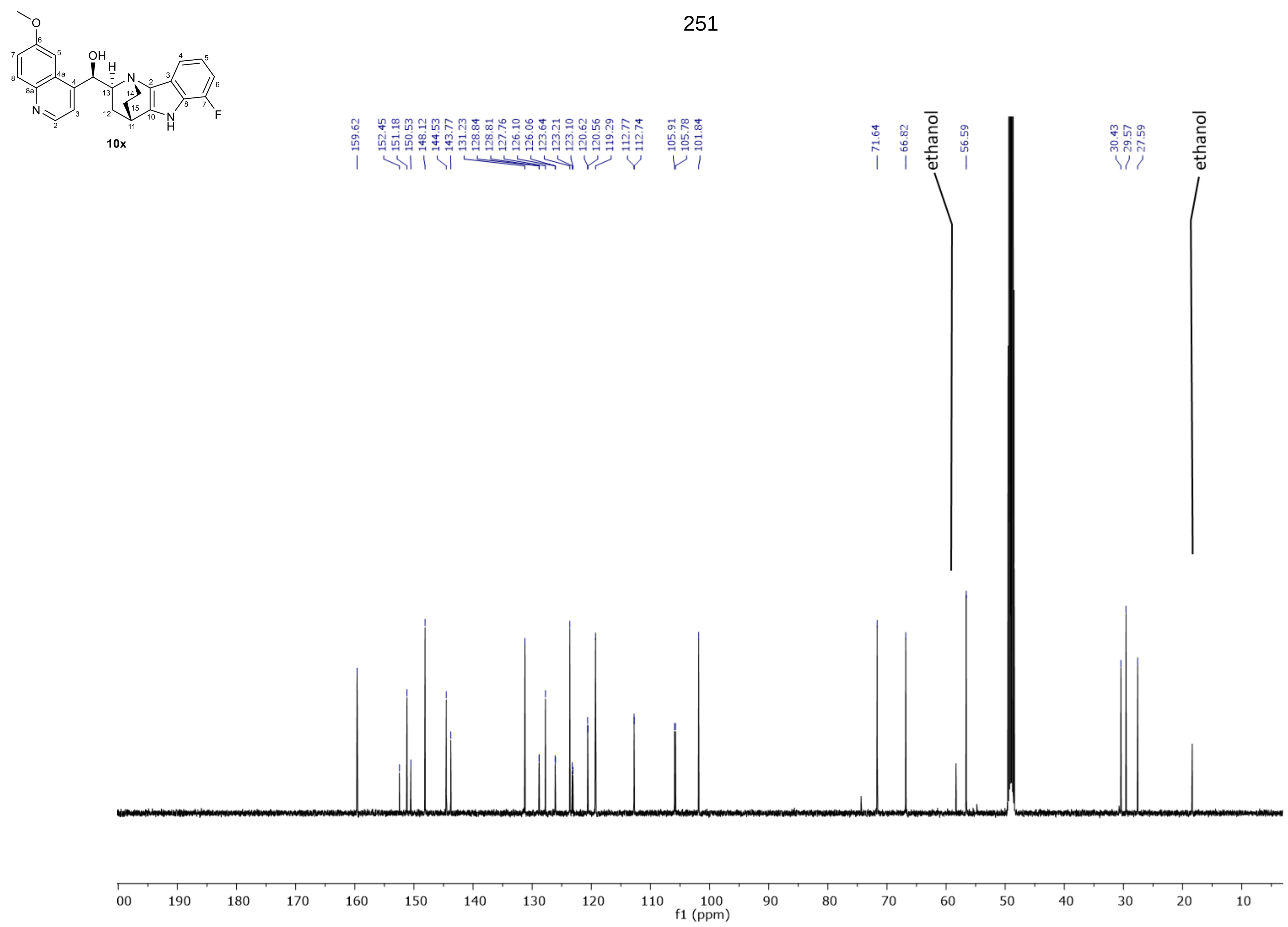
10x
159.62
152.45
151.18
150.53
148.12
144.53
143.77
131.23
128.84
128.81
127.76
126.10
126.06
123.64
123.21
123.10
120.62
120.56
119.29
112.77
112.74
105.91
105.78
101.84
71.64
66.82
ethanol
56.59
30.43
29.57
27.59
ethanol
f1 (ppm)

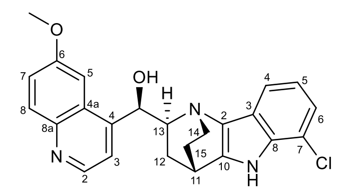

10y

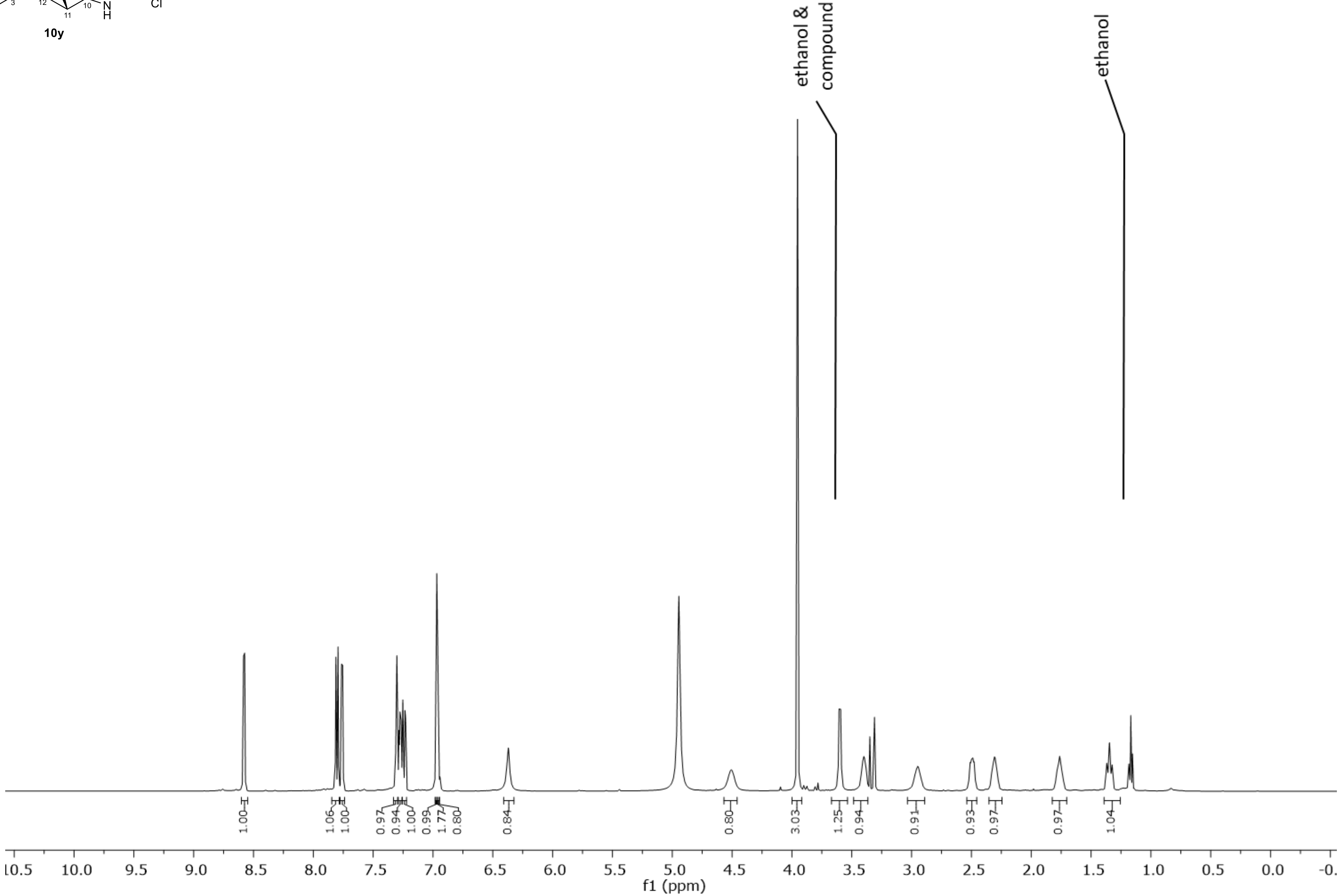
ethanol &
compound
ethanol
1.00
1.06
1.00
0.97
0.94
1.00
0.99
1.77
0.80
0.84
0.80
3.03
1.25
0.94
0.91
0.93
0.97
0.97
1.04
f1 (ppm)

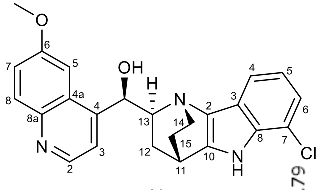
OH
H
N
Cl
N
H

10y

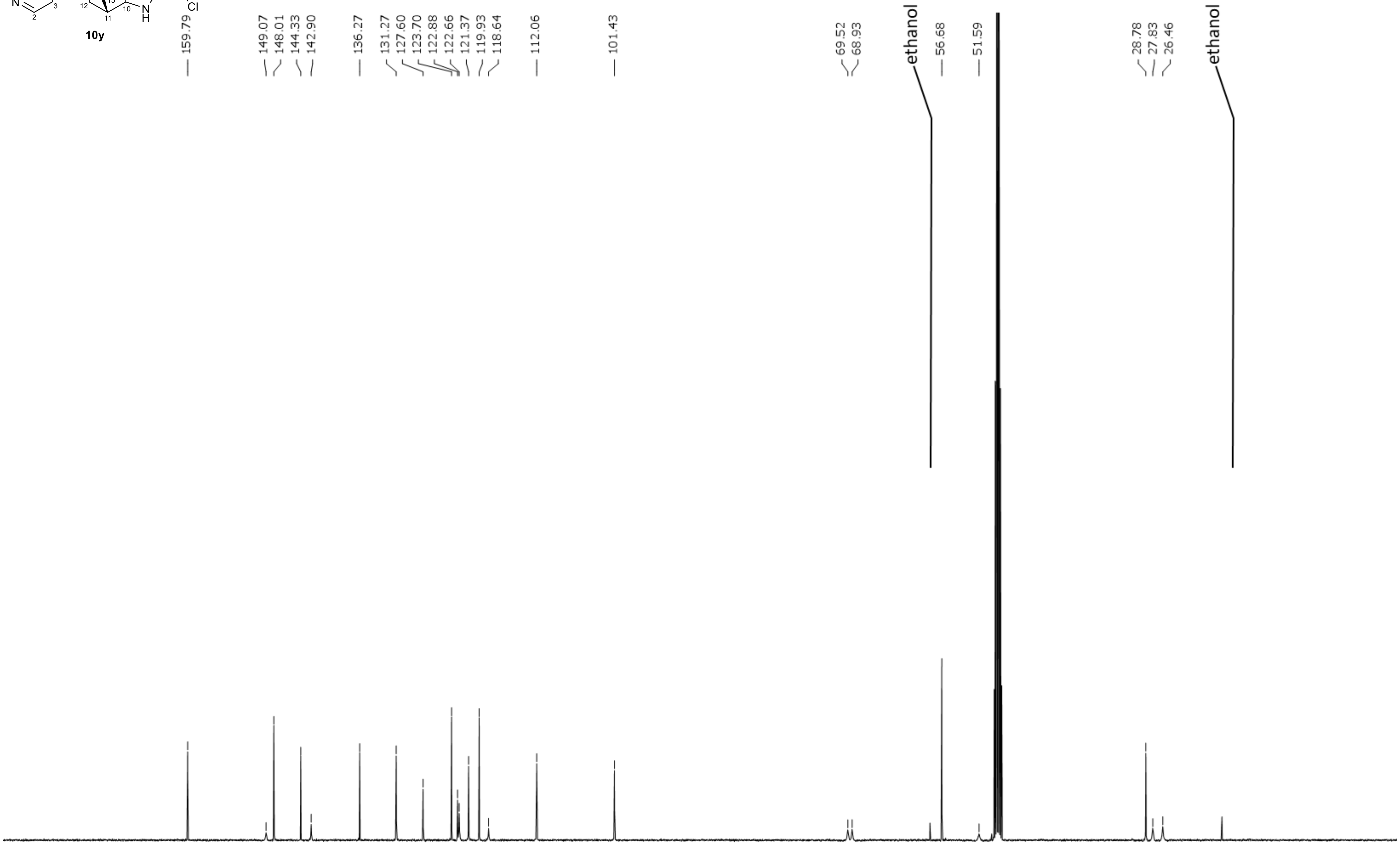
159.79
149.07
148.01
144.33
142.90
136.27
131.27
127.60
123.70
122.88
122.66
121.37
119.93
118.64
112.06
101.43
69.52
68.93
ethanol
56.68
51.59
28.78
27.83
26.46
ethanol

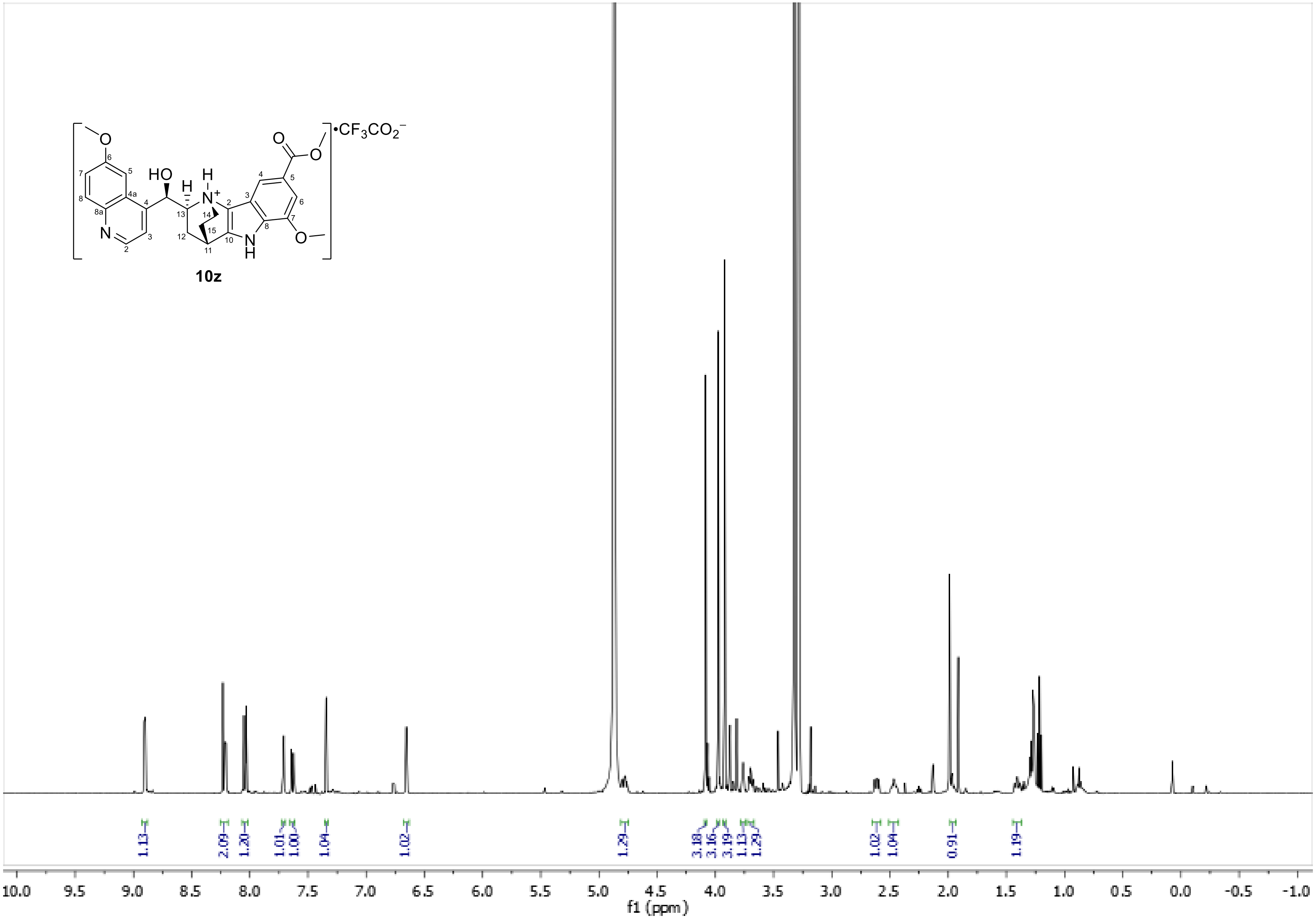
•CF3CO2−
HO
H
H
N+
N
H
O
O
O
O
N
10z
1.13
2.09
1.20
1.01
1.00
1.04
1.02
1.29
3.18
3.16
3.19
1.13
1.29
1.02
1.04
0.91
1.19
10.0
9.5
9.0
8.5
8.0
7.5
7.0
6.5
6.0
5.5
5.0
4.5
4.0
3.5
3.0
2.5
2.0
1.5
1.0
0.5
0.0
-0.5
-1.0
f1 (ppm)

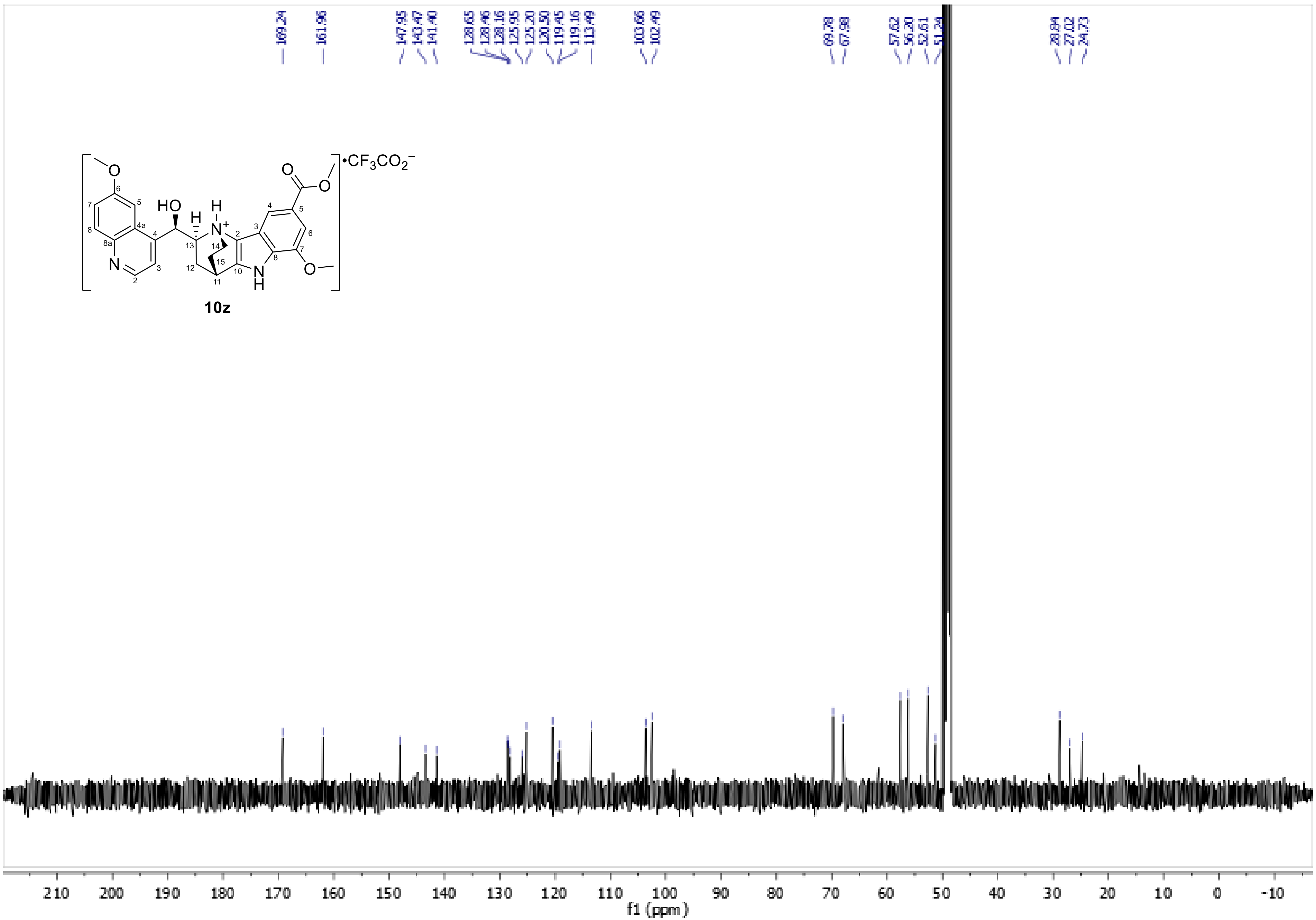
169.24
161.96
147.95
143.47
141.40
128.65
128.46
128.16
125.95
125.20
120.50
119.45
119.16
113.49
103.66
102.49
69.78
67.98
57.62
56.20
52.61
•CF3CO2−
HO
H
N+
NH
O
N
10z
28.84
27.02
24.73
210
200
190
180
170
160
150
140
130
120
110
100
90
80
70
60
50
40
30
20
10
0
-10
f1 (ppm)

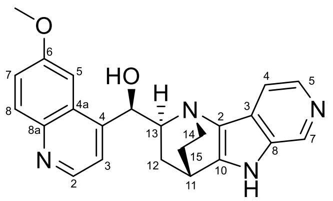
HO
H
N
N
NH
N
2
3
4
4a
5
6
7
8
8a
10
11
12
13
14
15
7
8
5
4
3
2

10v

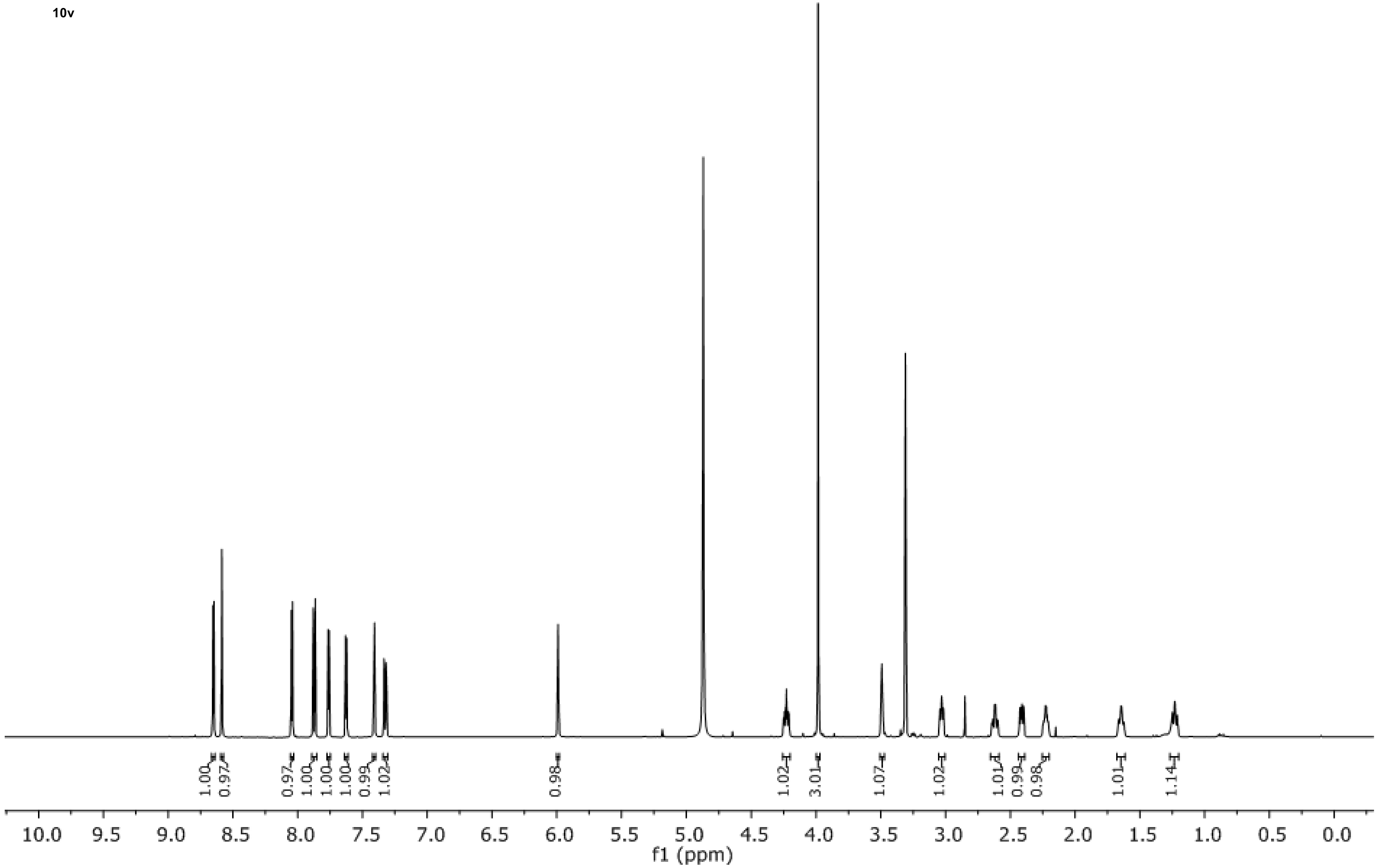
1.00
0.97
0.97
1.00
1.00
1.00
0.99
1.02
0.98
1.02
3.01
1.07
1.02
1.01
0.99
0.98
1.01
1.14
10.0
9.5
9.0
8.5
8.0
7.5
7.0
6.5
6.0
5.5
5.0
4.5
4.0
3.5
3.0
2.5
2.0
1.5
1.0
0.5
0.0
f1 (ppm)

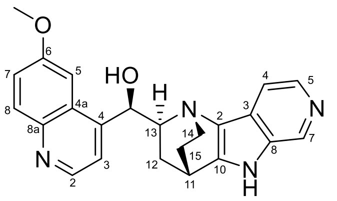

10v

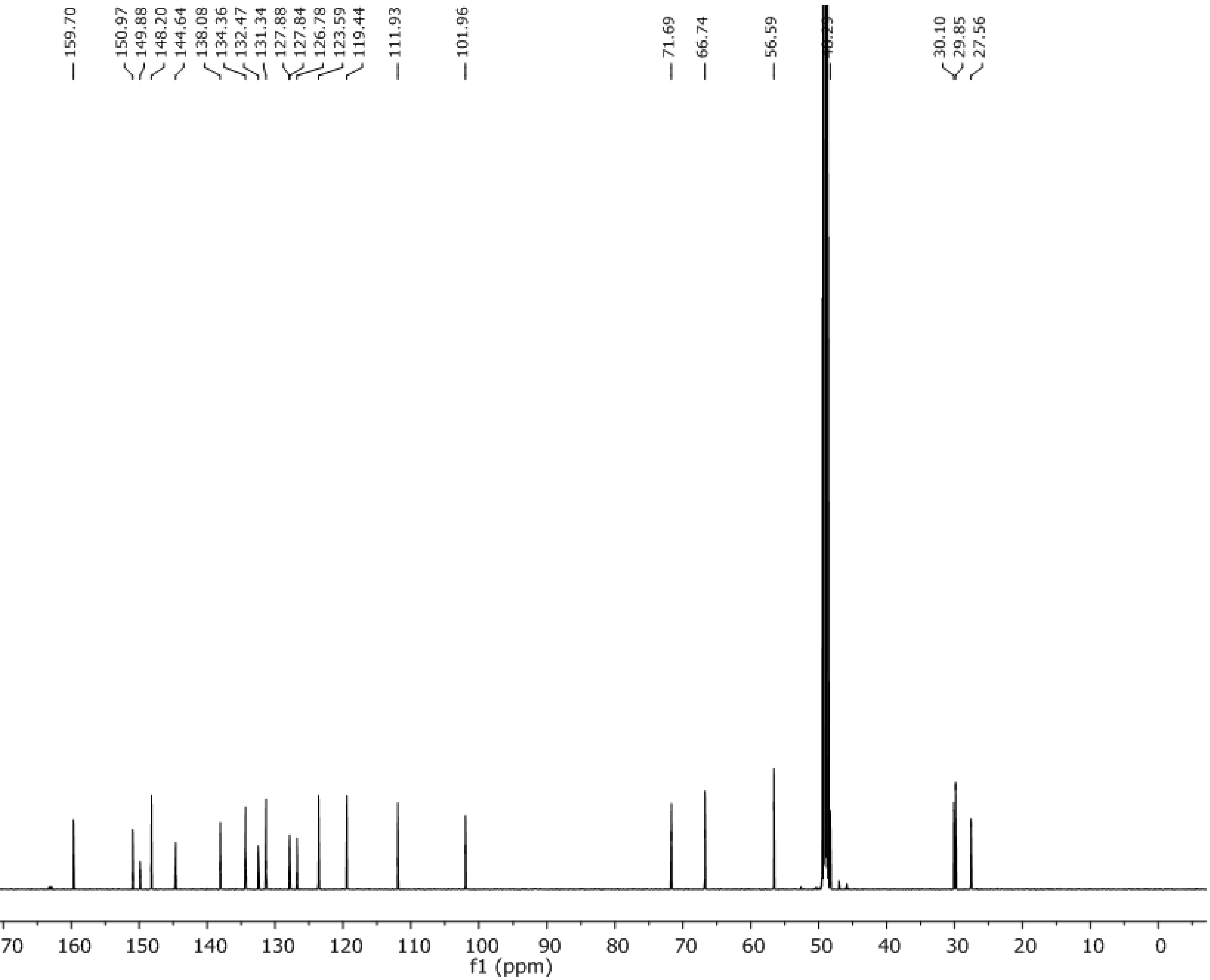
159.70
150.97
149.88
148.20
144.64
138.08
134.36
132.47
131.34
127.88
127.84
126.78
123.59
119.44
111.93
101.96
71.69
66.74
56.59
30.10
29.85
27.56
190 180 170 160 150 140 130 120 110 100 90 80 70 60 50 40 30 20 10 0
f1 (ppm)

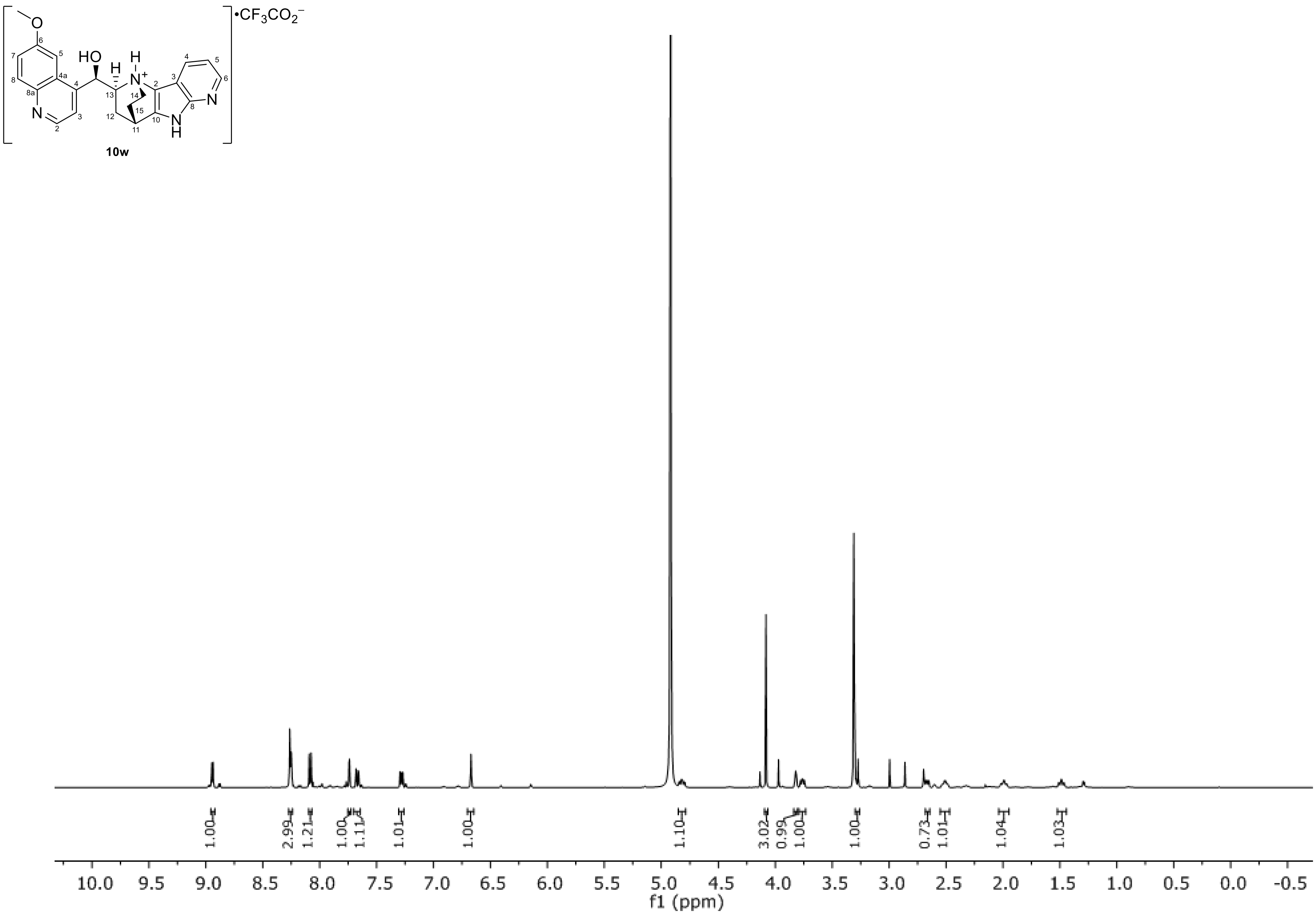
•CF3CO2−
HO
H
N+
N
NH
N
1.00
2.99
1.21
1.00
1.11
1.01
1.00
1.10
3.02
0.99
1.00
1.00
0.73
1.01
1.04
1.03
10.0 9.5 9.0 8.5 8.0 7.5 7.0 6.5 6.0 5.5 5.0 4.5 4.0 3.5 3.0 2.5 2.0 1.5 1.0 0.5 0.0 -0.5
f1 (ppm)

10w

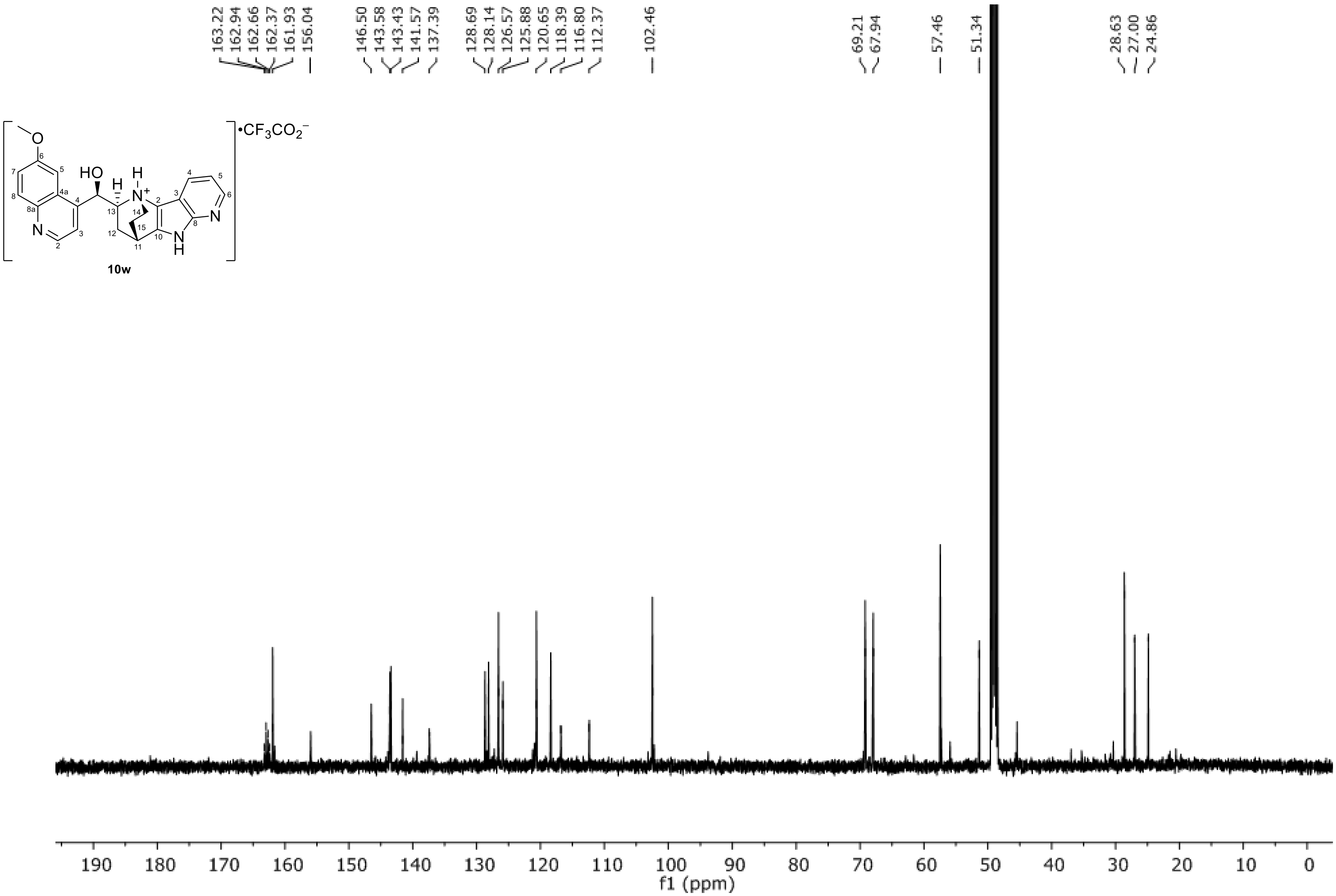
163.22
162.94
162.66
162.37
161.93
156.04
146.50
143.58
143.43
141.57
137.39
128.69
128.14
126.57
125.88
120.65
118.39
116.80
112.37
102.46
69.21
67.94
57.46
51.34
28.63
27.00
24.86
O
HO
H
H
N+
N
N
NH
•CF3CO2−
190
180
170
160
150
140
130
120
110
100
90
80
70
60
50
40
30
20
10
0
f1 (ppm)

10w

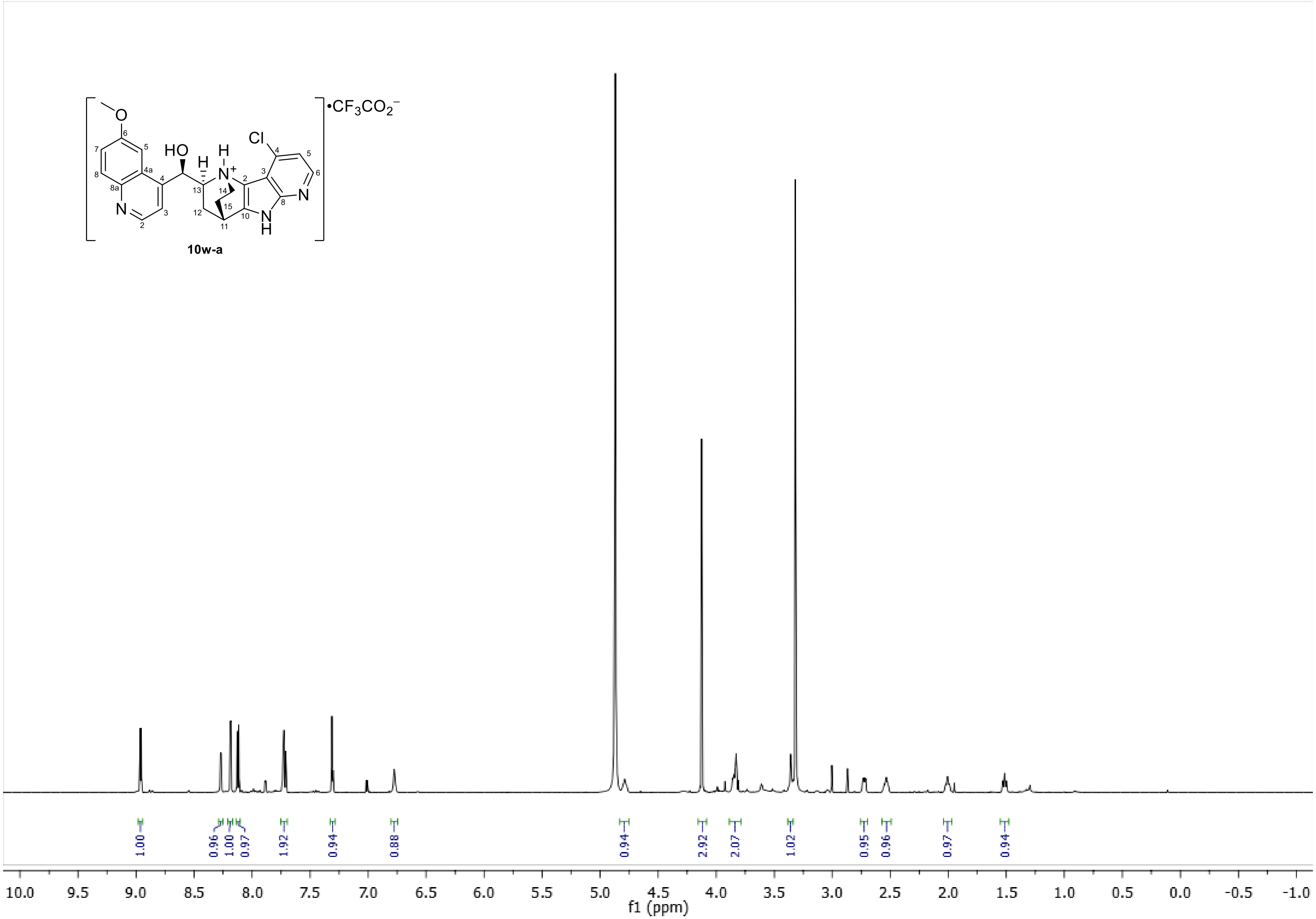
•CF3CO2−
10w-a
1.00
0.96
1.00
0.97
1.92
0.94
0.88
0.94
2.92
2.07
1.02
0.95
0.96
0.97
0.94
10.0 9.5 9.0 8.5 8.0 7.5 7.0 6.5 6.0 5.5 5.0 4.5 4.0 3.5 3.0 2.5 2.0 1.5 1.0 0.5 0.0 -0.5 -1.0
f1 (ppm)

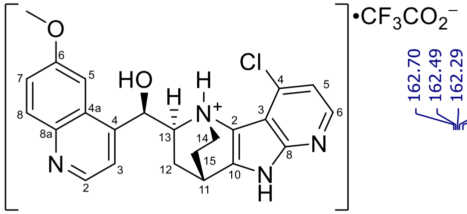
HO
Cl
•CF3CO2−

10w-a

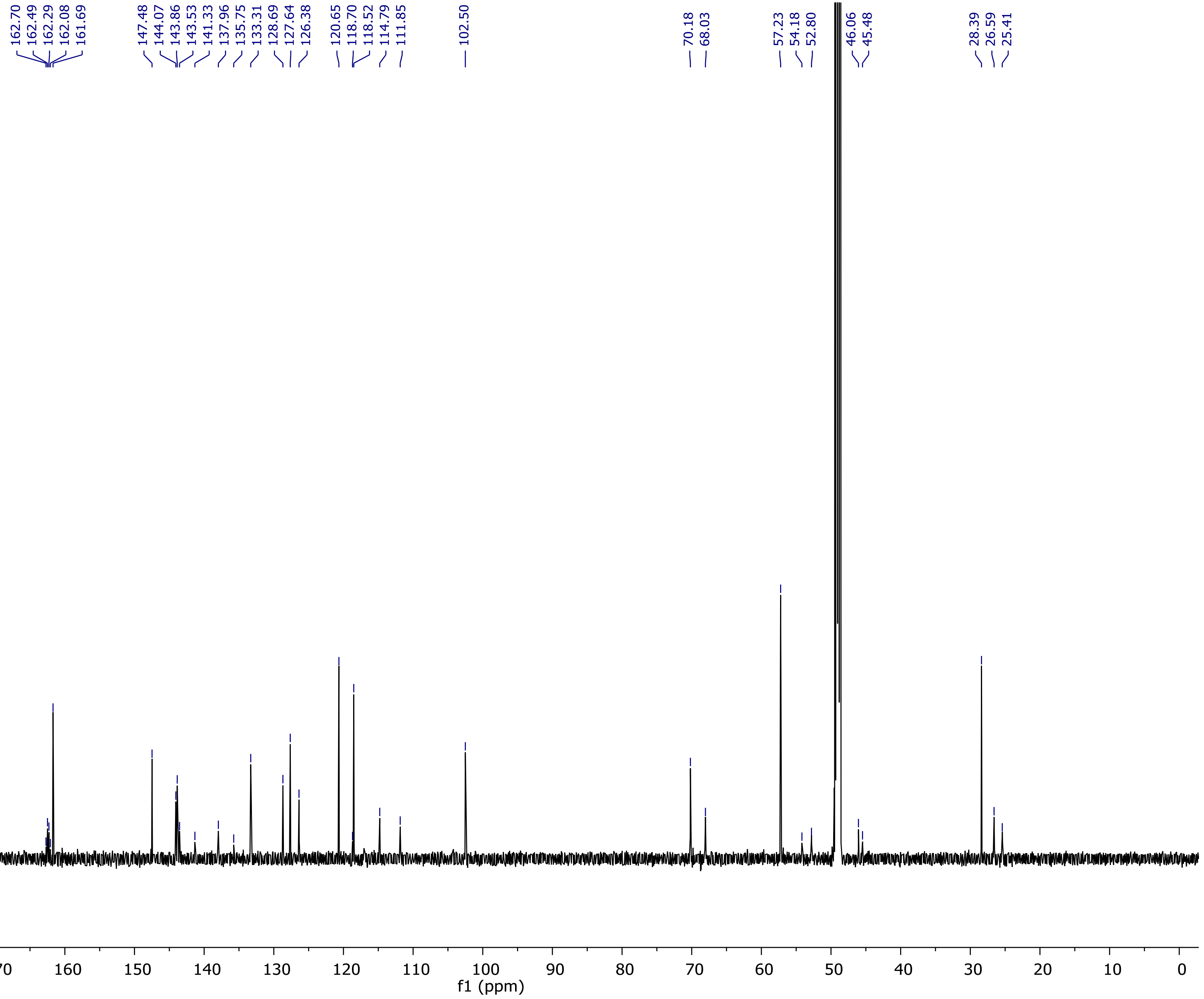
162.70
162.49
162.29
162.08
161.69
147.48
144.07
143.86
143.53
141.33
137.96
135.75
133.31
128.69
127.64
126.38
120.65
118.70
118.52
114.79
111.85
102.50
70.18
68.03
57.23
54.18
52.80
46.06
45.48
28.39
26.59
25.41
200 190 180 170 160 150 140 130 120 110 100 90 80 70 60 50 40 30 20 10 0
f1 (ppm)

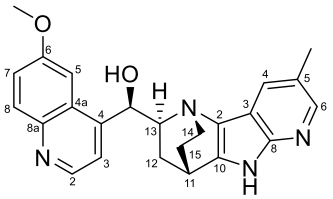

10w-b

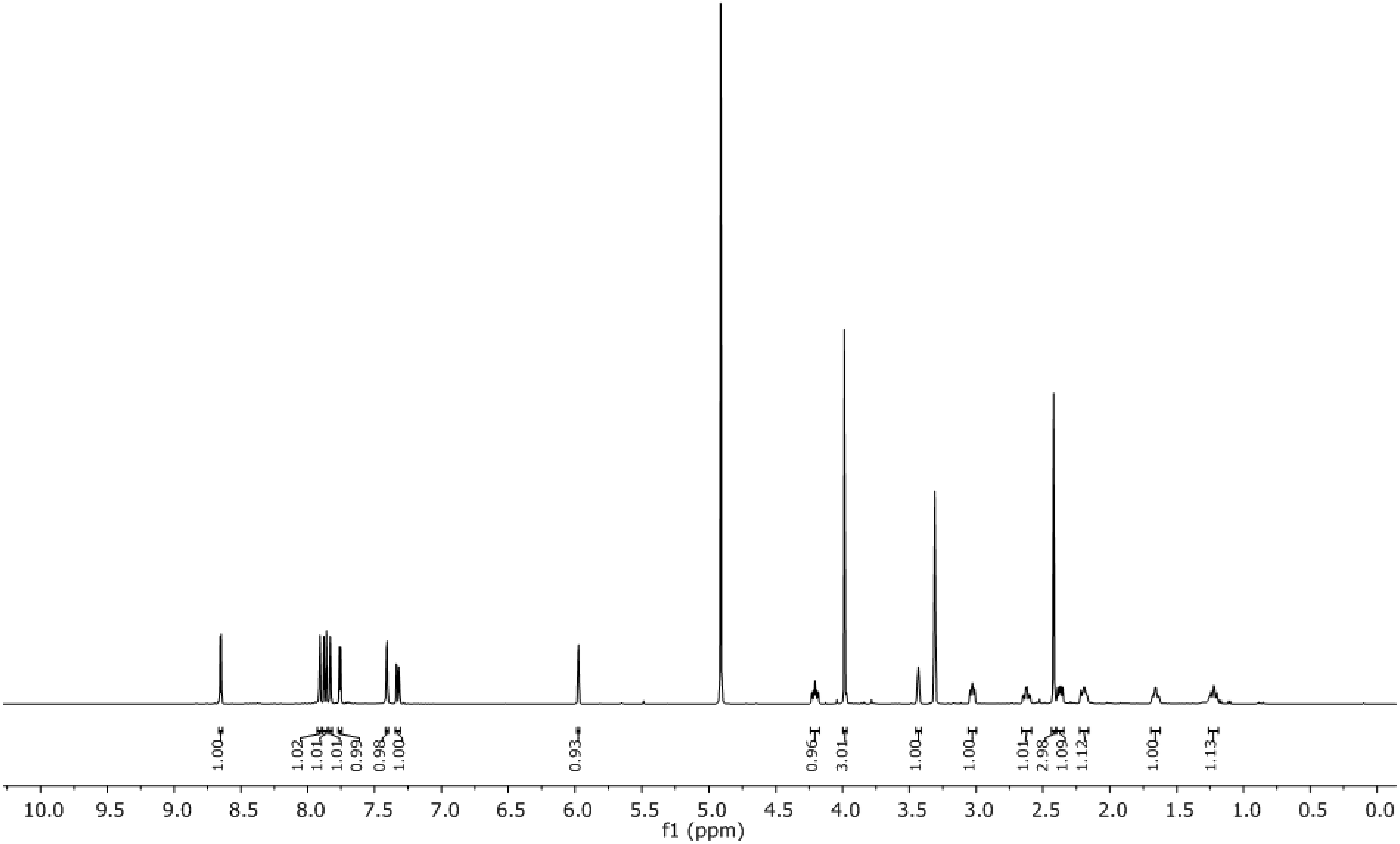
1.00
1.02
1.01
1.01
0.99
0.98
1.00
0.93
0.96
3.01
1.00
1.00
1.01
2.98
1.09
1.12
1.00
1.13
10.0 9.5 9.0 8.5 8.0 7.5 7.0 6.5 6.0 5.5 5.0 4.5 4.0 3.5 3.0 2.5 2.0 1.5 1.0 0.5 0.0
f1 (ppm)

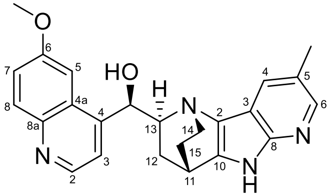

10w-b

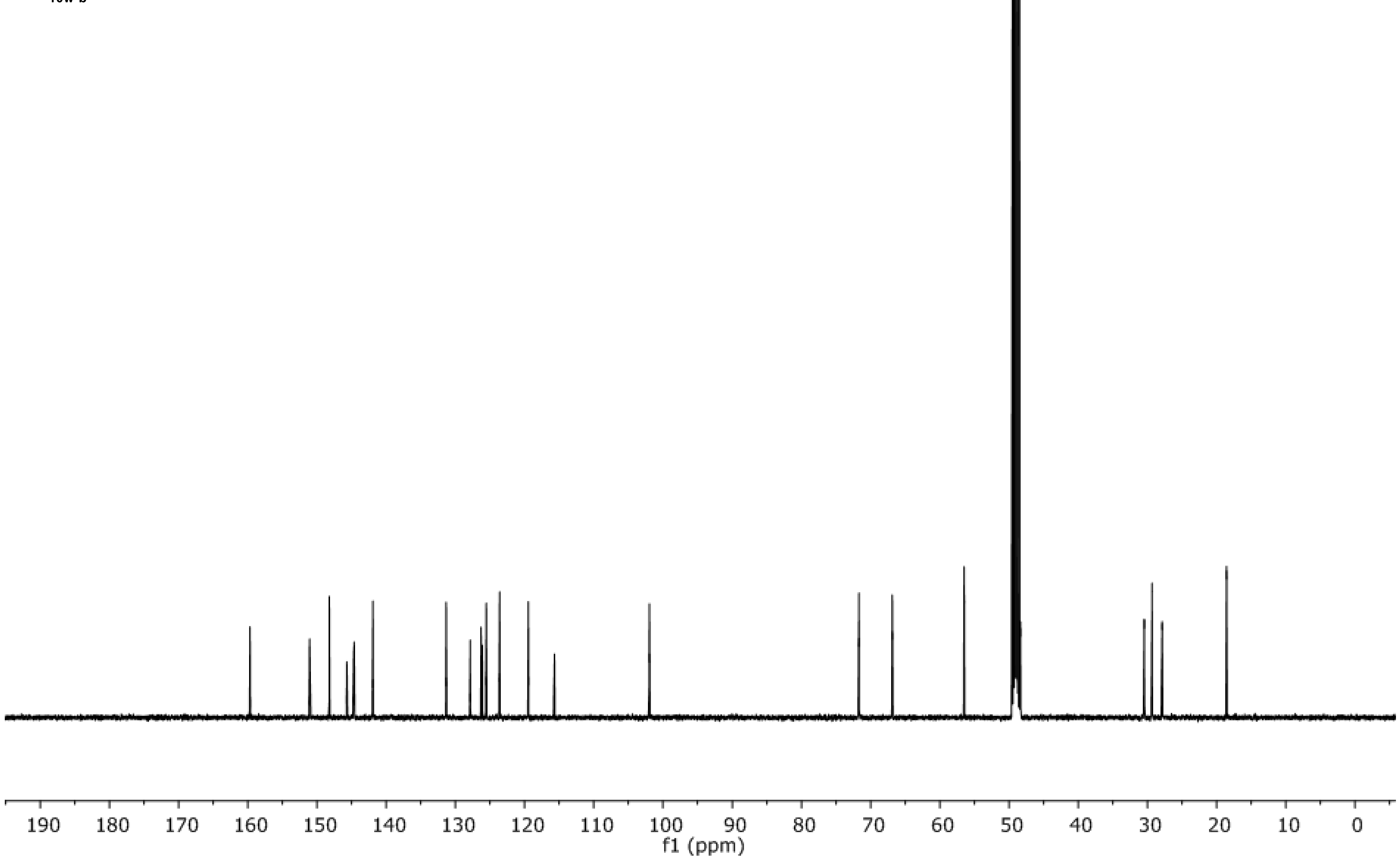
159.66
151.06
148.20
145.67
144.68
144.61
141.90
131.31
127.84
126.26
126.07
125.52
123.59
119.43
115.65
101.95
71.70
66.88
56.49
30.46
29.33
27.86
18.56
190 180 170 160 150 140 130 120 110 100 90 80 70 60 50 40 30 20 10 0
f1 (ppm)

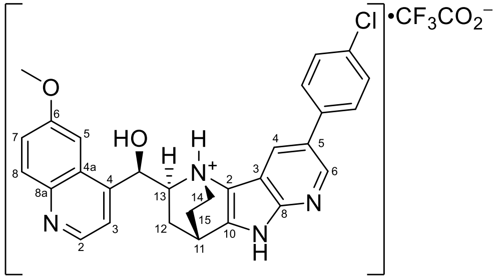

10w-c

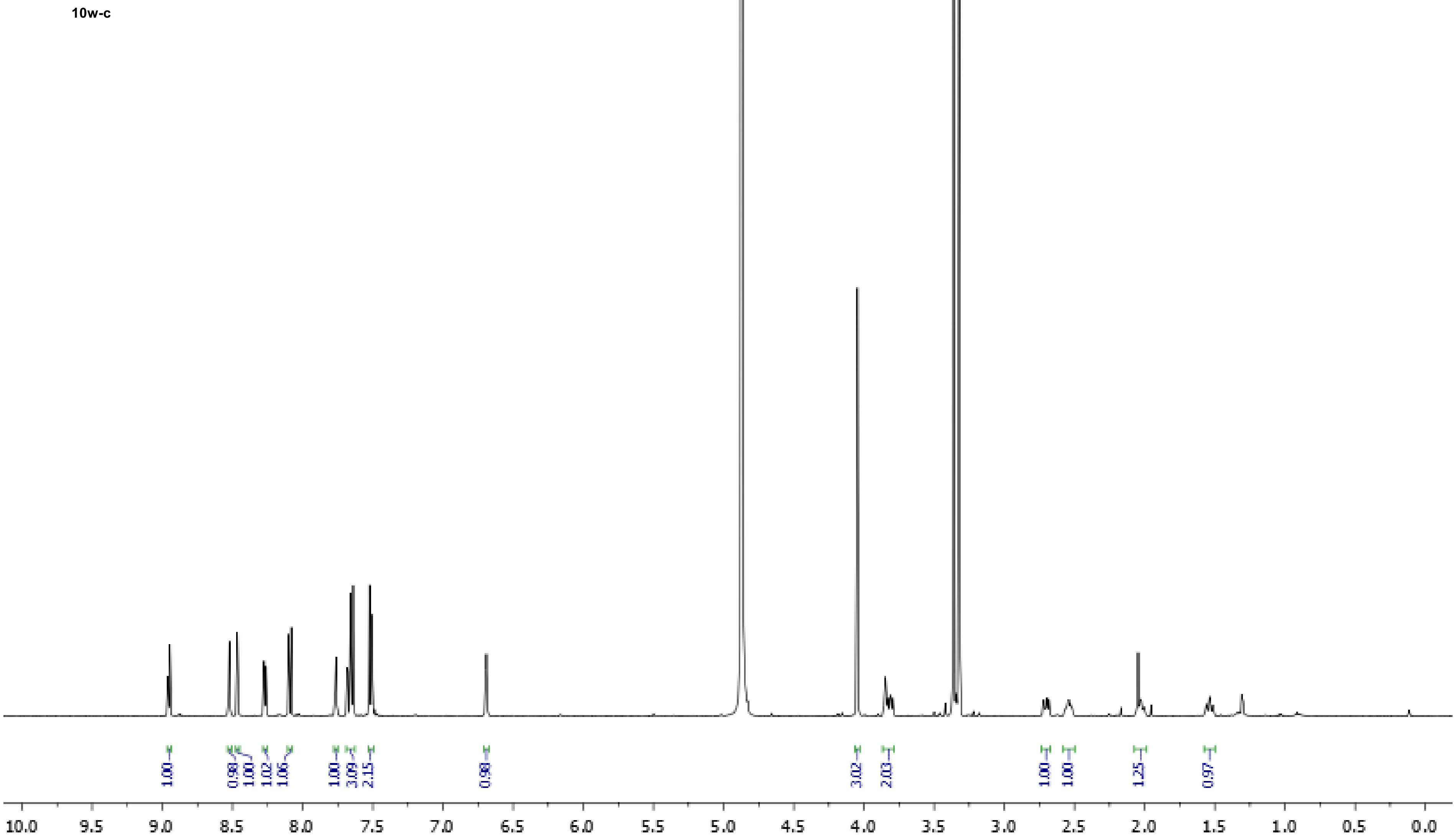

1.00
0.98
1.00
1.02
1.06
1.00
3.09
2.15
0.98
3.02
2.03
1.00
1.00
1.25
0.97
10.0 9.5 9.0 8.5 8.0 7.5 7.0 6.5 6.0 5.5 5.0 4.5 4.0 3.5 3.0 2.5 2.0 1.5 1.0 0.5 0.0

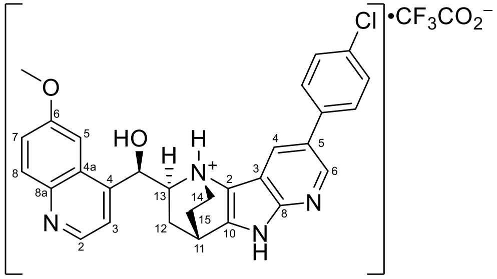

10w-c

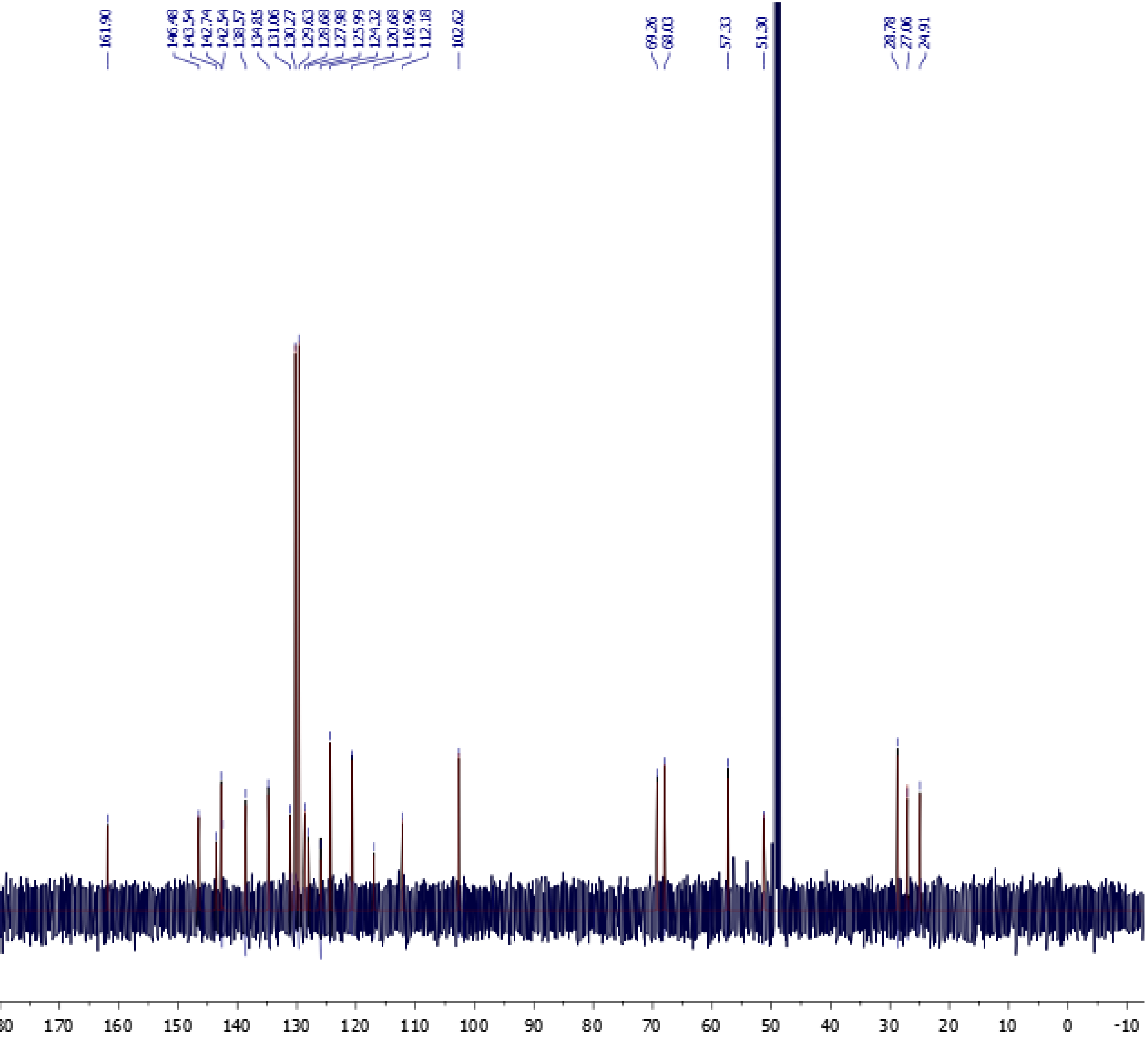
161.90
146.48
143.54
142.74
142.54
138.57
134.85
131.06
130.27
129.63
128.68
127.98
125.99
124.32
120.68
116.96
112.18
102.62
69.26
68.03
57.33
51.30
28.78
27.06
24.91
230 220 210 200 190 180 170 160 150 140 130 120 110 100 90 80 70 60 50 40 30 20 10 0 -10

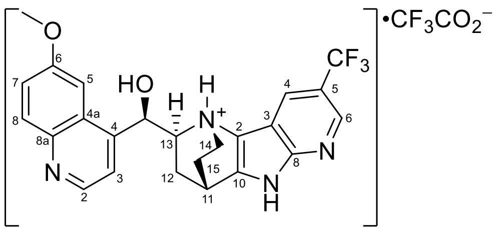

•CF3CO2−
CF3
HO
H
H
N+
N
N
N
H

10w-d

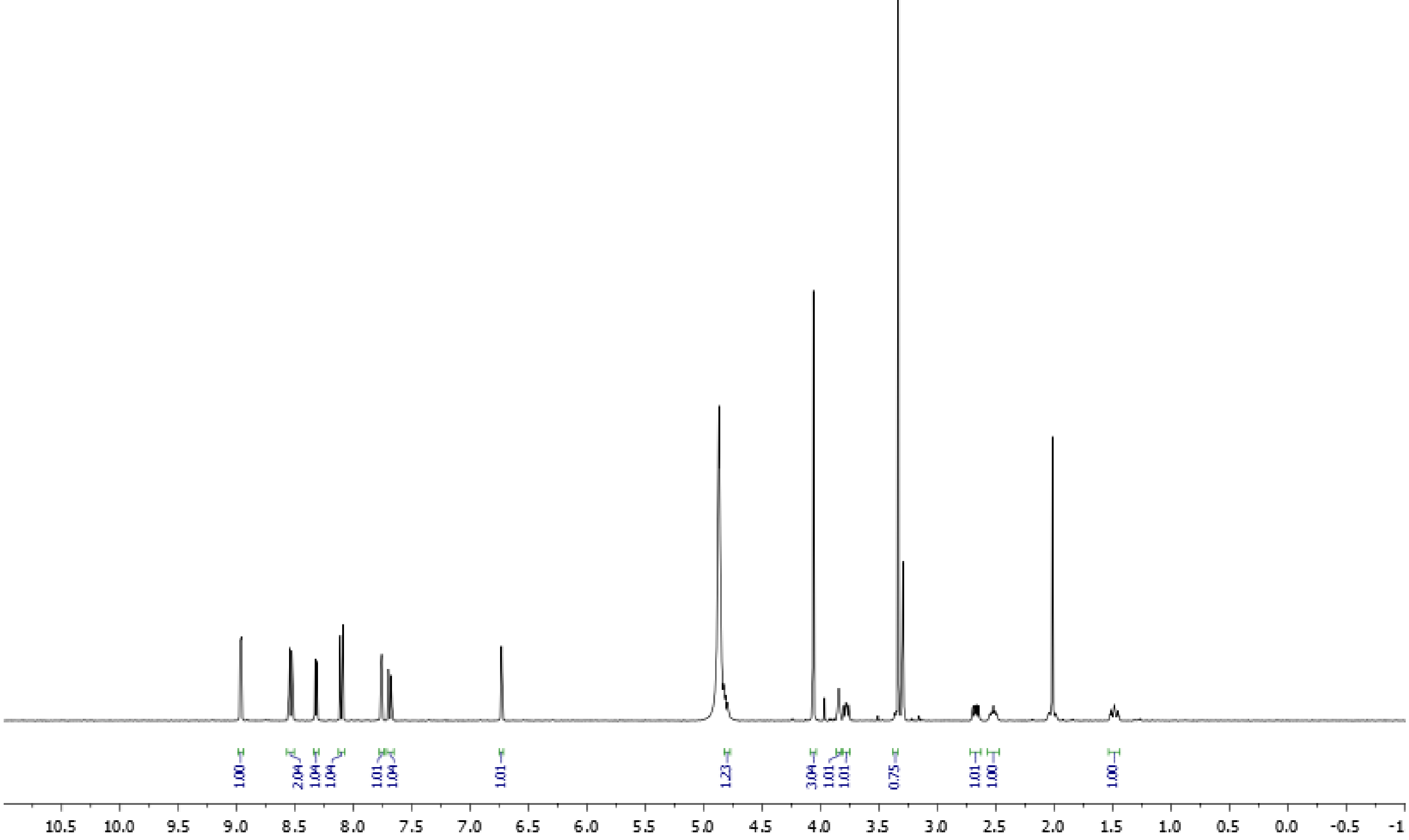

1.00
2.04
1.04
1.04
1.01
1.04
1.01
1.23
3.04
1.01
1.01
0.75
1.01
1.00
1.00
10.5
10.0
9.5
9.0
8.5
8.0
7.5
7.0
6.5
6.0
5.5
5.0
4.5
4.0
3.5
3.0
2.5
2.0
1.5
1.0
0.5
0.0
-0.5
-1

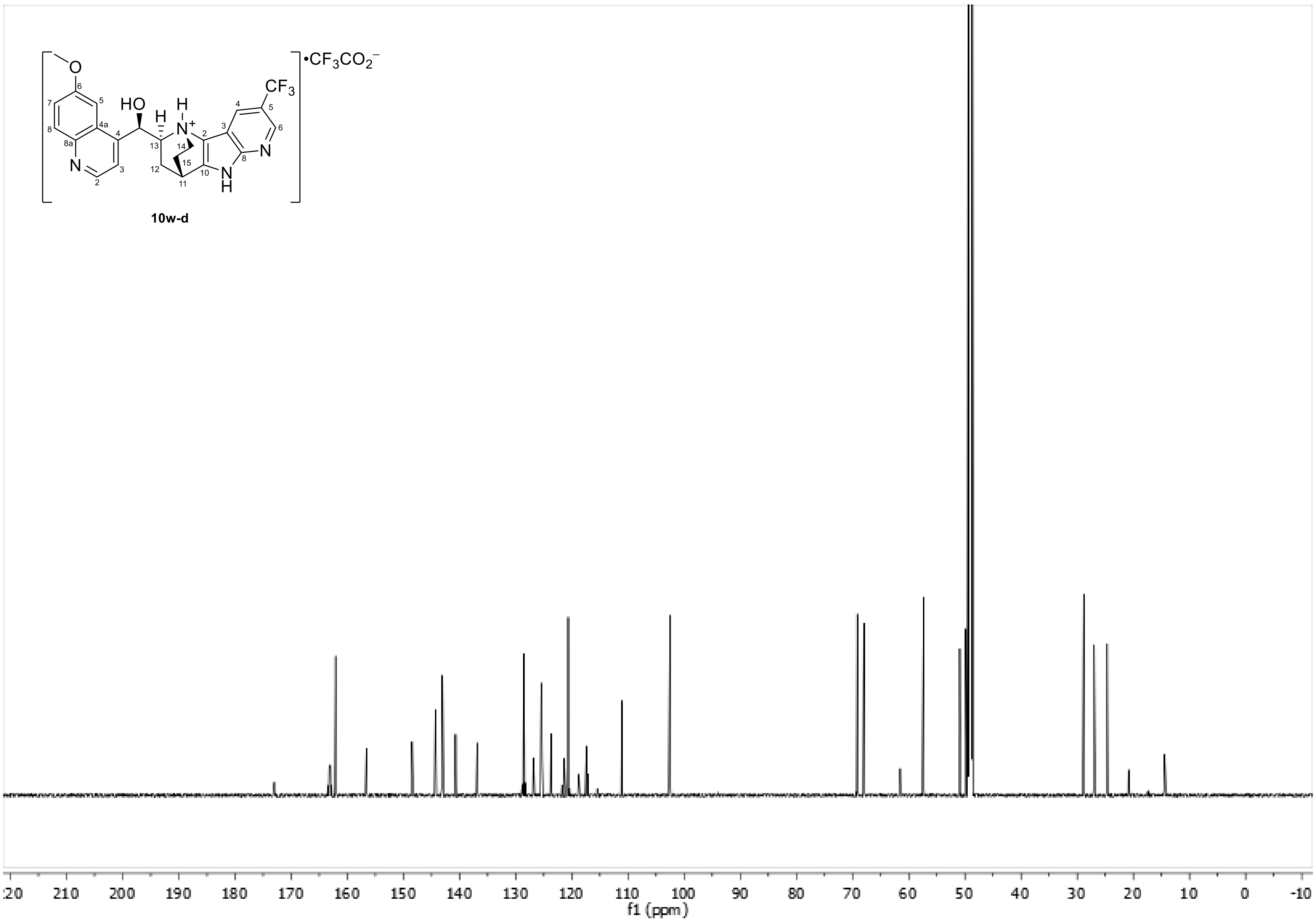
•CF3CO2−
CF3
HO
10w-d
f1 (ppm)
220 210 200 190 180 170 160 150 140 130 120 110 100 90 80 70 60 50 40 30 20 10 0 -10

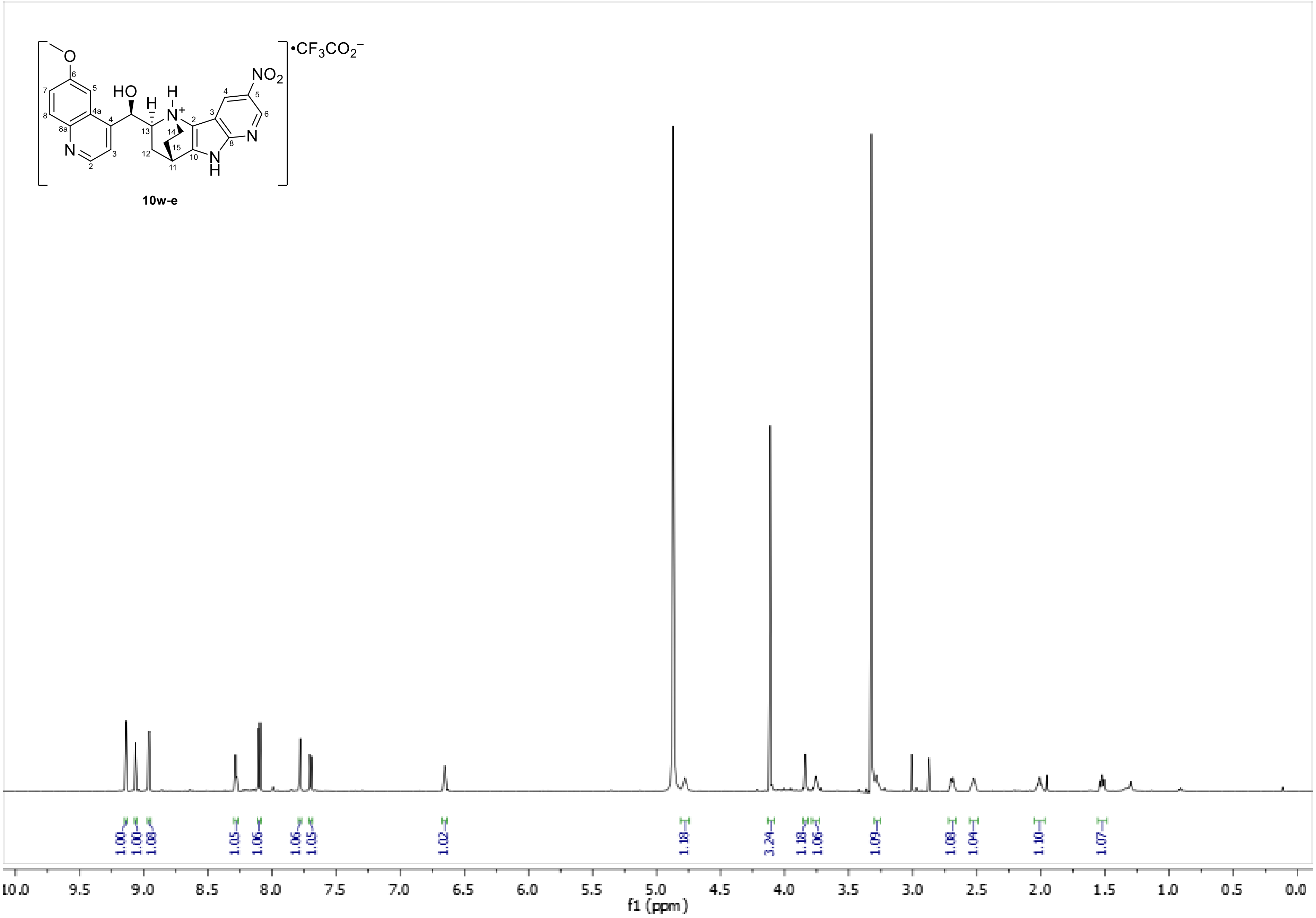
•CF3CO2−
HO
H
N+
NO2
N
NH
10w-e
1.00
1.00
1.08
1.05
1.06
1.06
1.05
1.02
1.18
3.24
1.18
1.06
1.09
1.08
1.04
1.10
1.07
10.0
9.5
9.0
8.5
8.0
7.5
7.0
6.5
6.0
5.5
5.0
4.5
4.0
3.5
3.0
2.5
2.0
1.5
1.0
0.5
0.0
f1 (ppm)

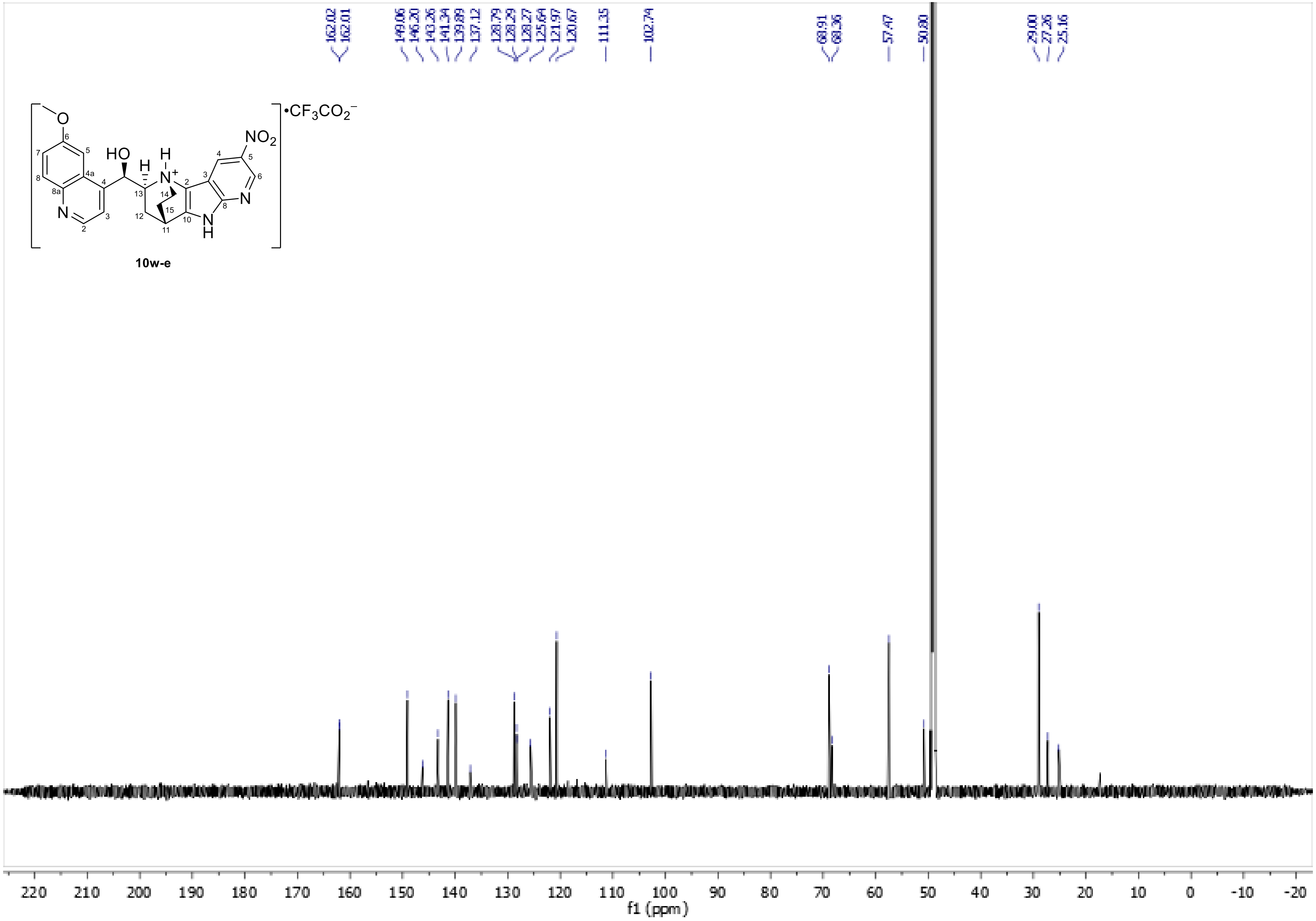
162.02
162.01
149.06
146.20
143.26
141.34
139.89
137.12
128.79
128.29
128.27
125.64
121.97
120.67
111.35
102.74
68.91
68.36
57.47
50.80
29.00
27.26
25.16
•CF3CO2−
NO2
HO
H
N+
N
NH
O
10w-e
f1 (ppm)

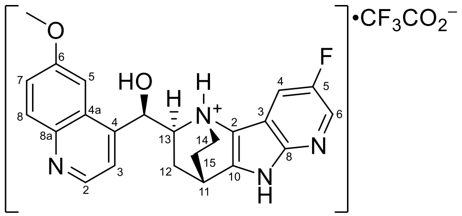
•CF3CO2−
F
HO
H
N+
N
NH
O

10w-f

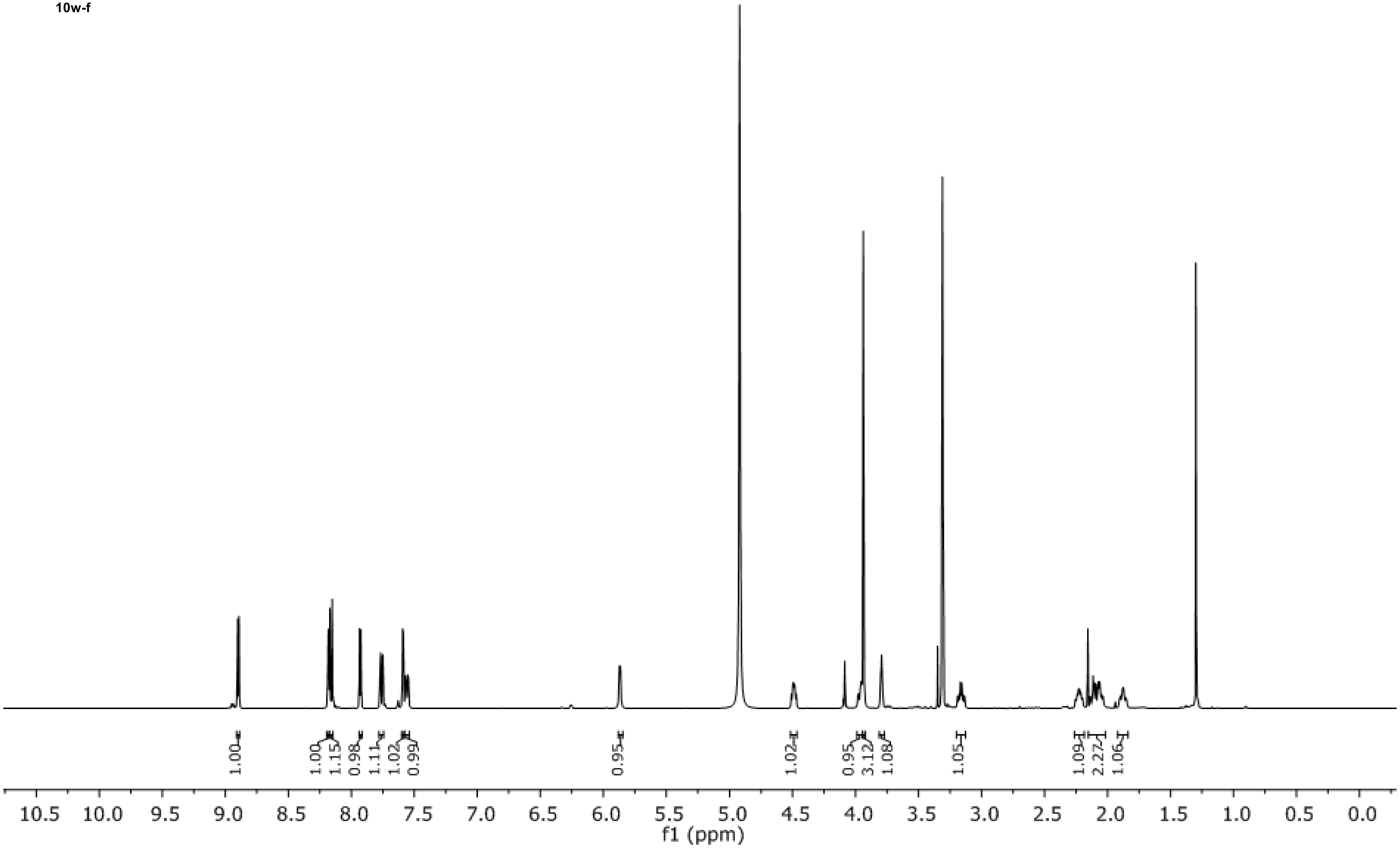
1.00
1.00
1.15
0.98
1.11
1.02
0.99
0.95
1.02
0.95
3.12
1.08
1.05
1.09
2.27
1.06
10.5 10.0 9.5 9.0 8.5 8.0 7.5 7.0 6.5 6.0 5.5 5.0 4.5 4.0 3.5 3.0 2.5 2.0 1.5 1.0 0.5 0.0
f1 (ppm)

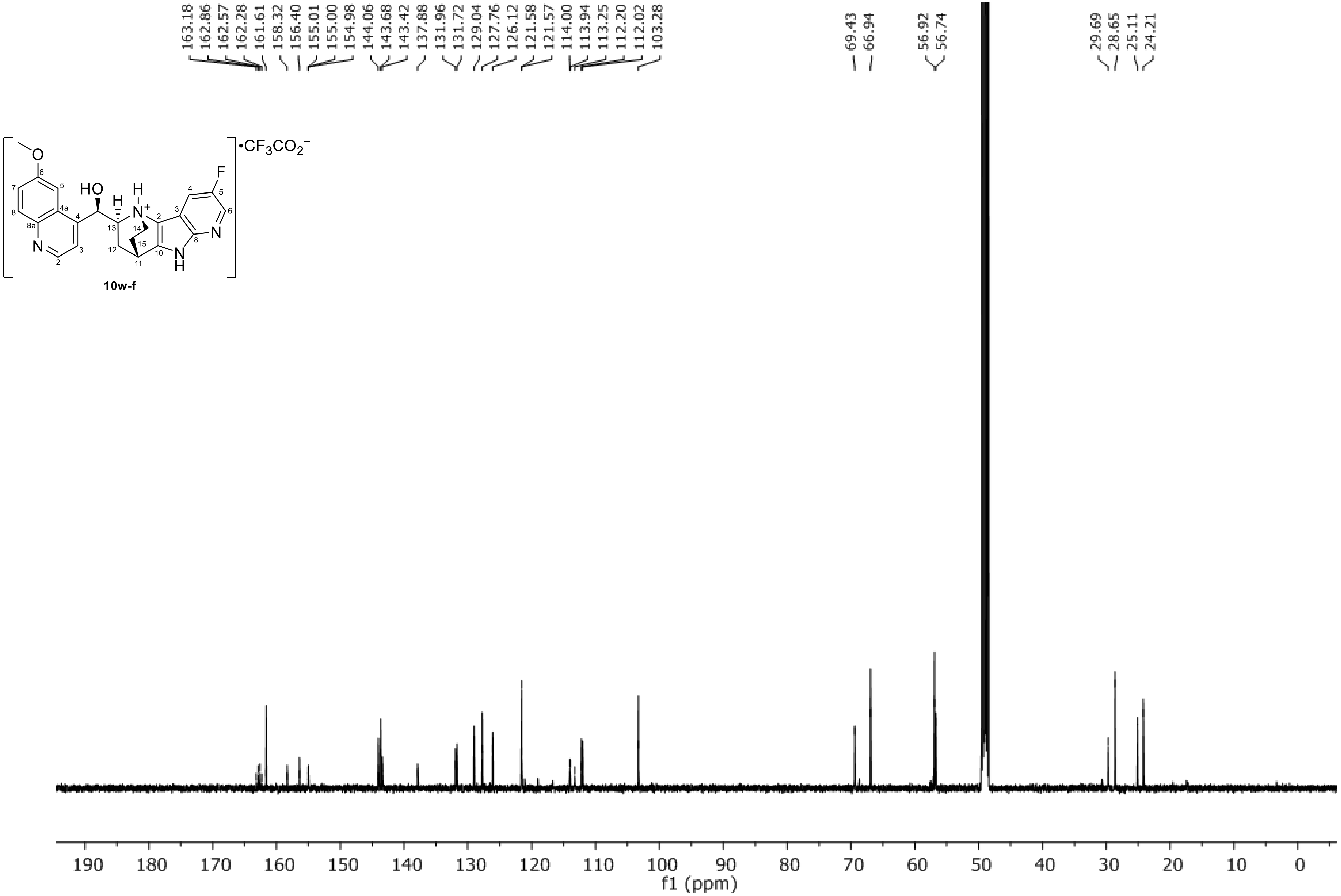
163.18
162.86
162.57
162.28
161.61
158.32
156.40
155.01
155.00
154.98
144.06
143.68
143.42
137.88
131.96
131.72
129.04
127.76
126.12
121.58
121.57
114.00
113.94
113.25
112.20
112.02
103.28
69.43
66.94
56.92
56.74
29.69
28.65
25.11
24.21
•CF3CO2⁻
10w-f
190 180 170 160 150 140 130 120 110 100 90 80 70 60 50 40 30 20 10 0
f1 (ppm)

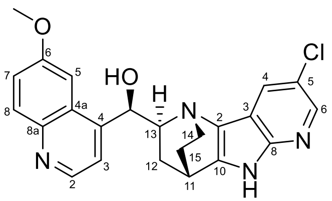

O
HO
H
N
Cl
N
NH
N

10w-g

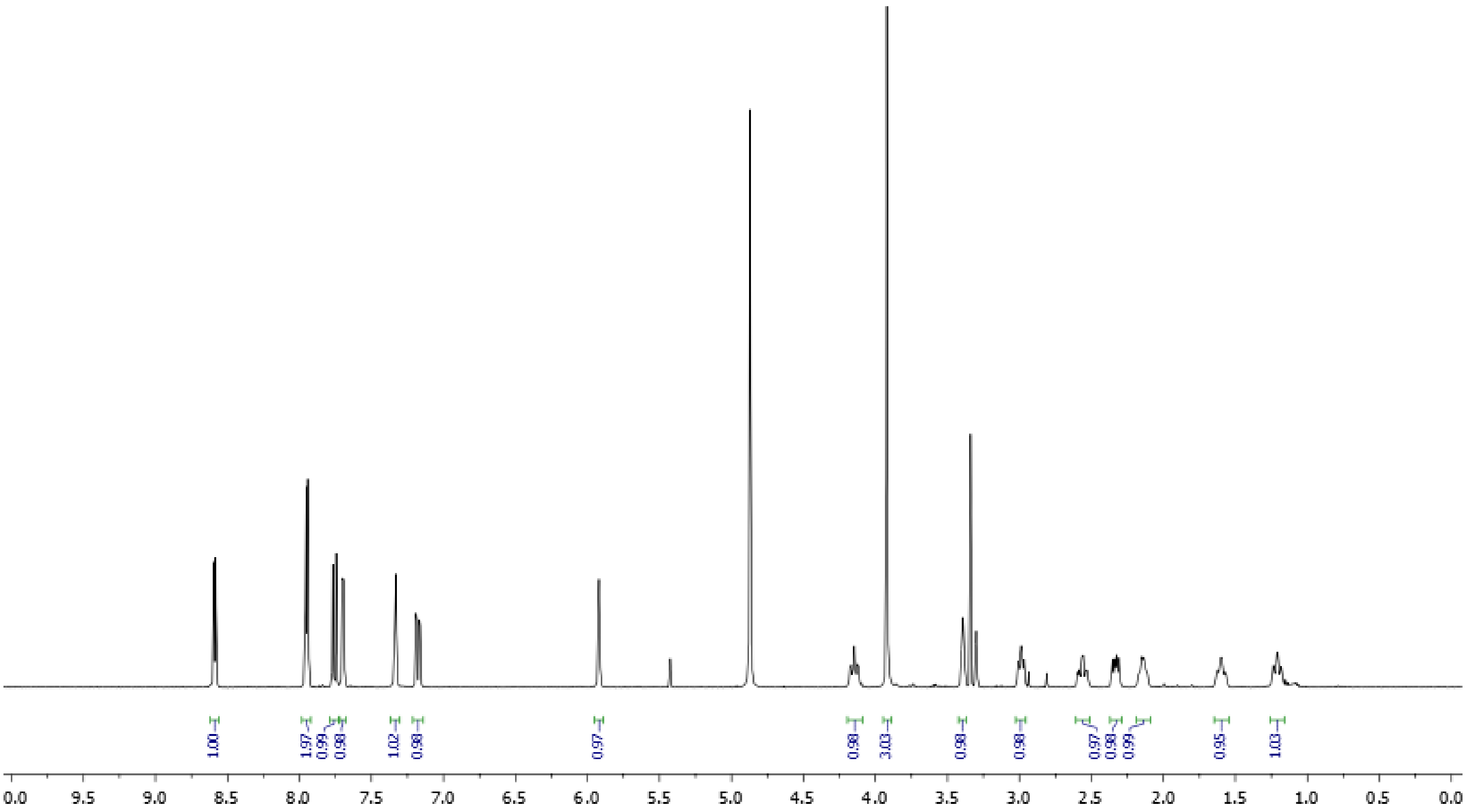

1.00
1.97
0.99
0.98
1.02
0.98
0.97
0.98
3.03
0.98
0.98
0.97
0.98
0.99
0.95
1.03
0.0 9.5 9.0 8.5 8.0 7.5 7.0 6.5 6.0 5.5 5.0 4.5 4.0 3.5 3.0 2.5 2.0 1.5 1.0 0.5 0.0

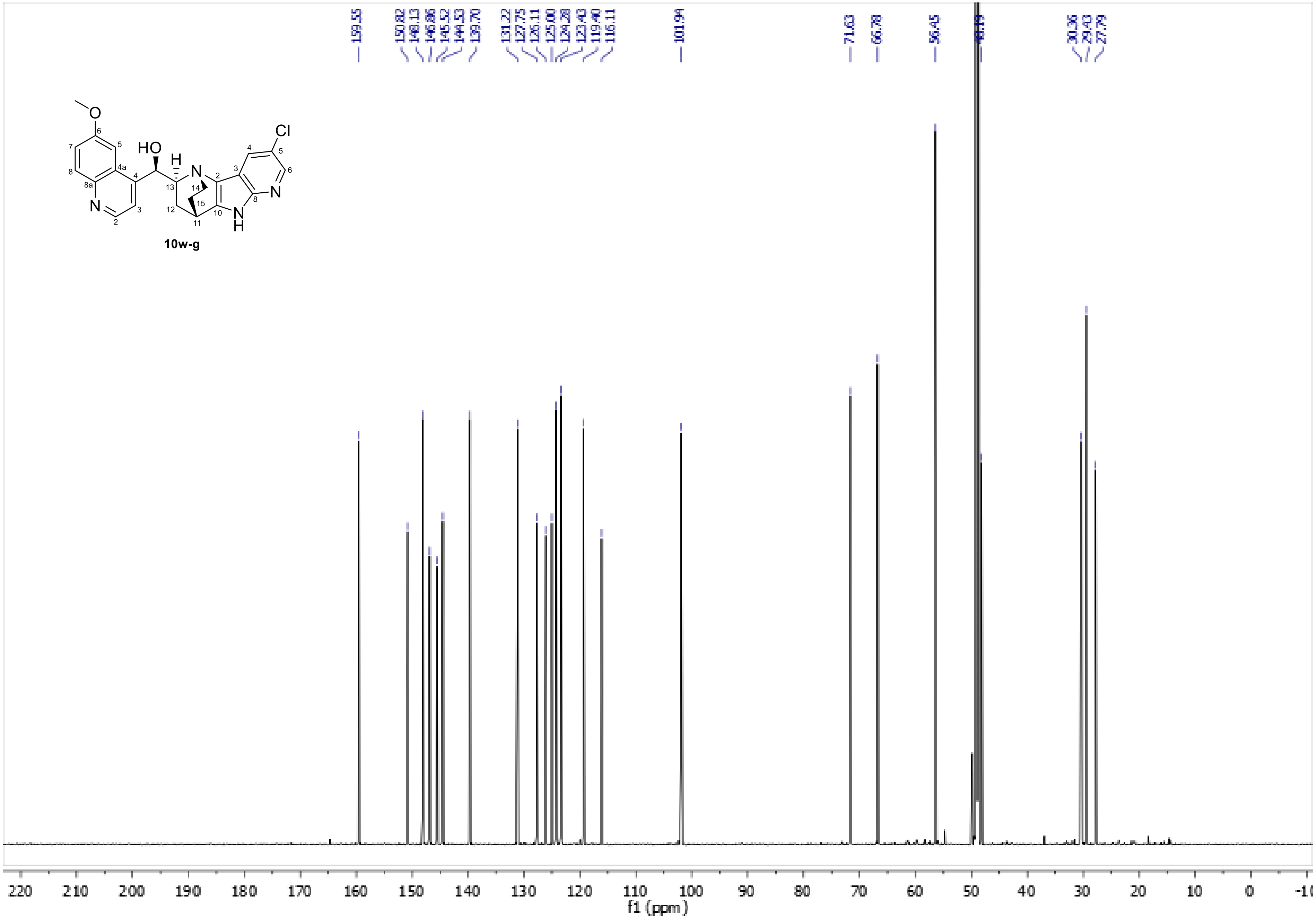

159.55
150.82
148.13
146.86
145.52
144.53
139.70
131.22
127.75
126.11
125.00
124.28
123.43
119.40
116.11
101.94
71.63
66.78
56.45
30.36
29.43
27.79
10w-g
f1 (ppm)

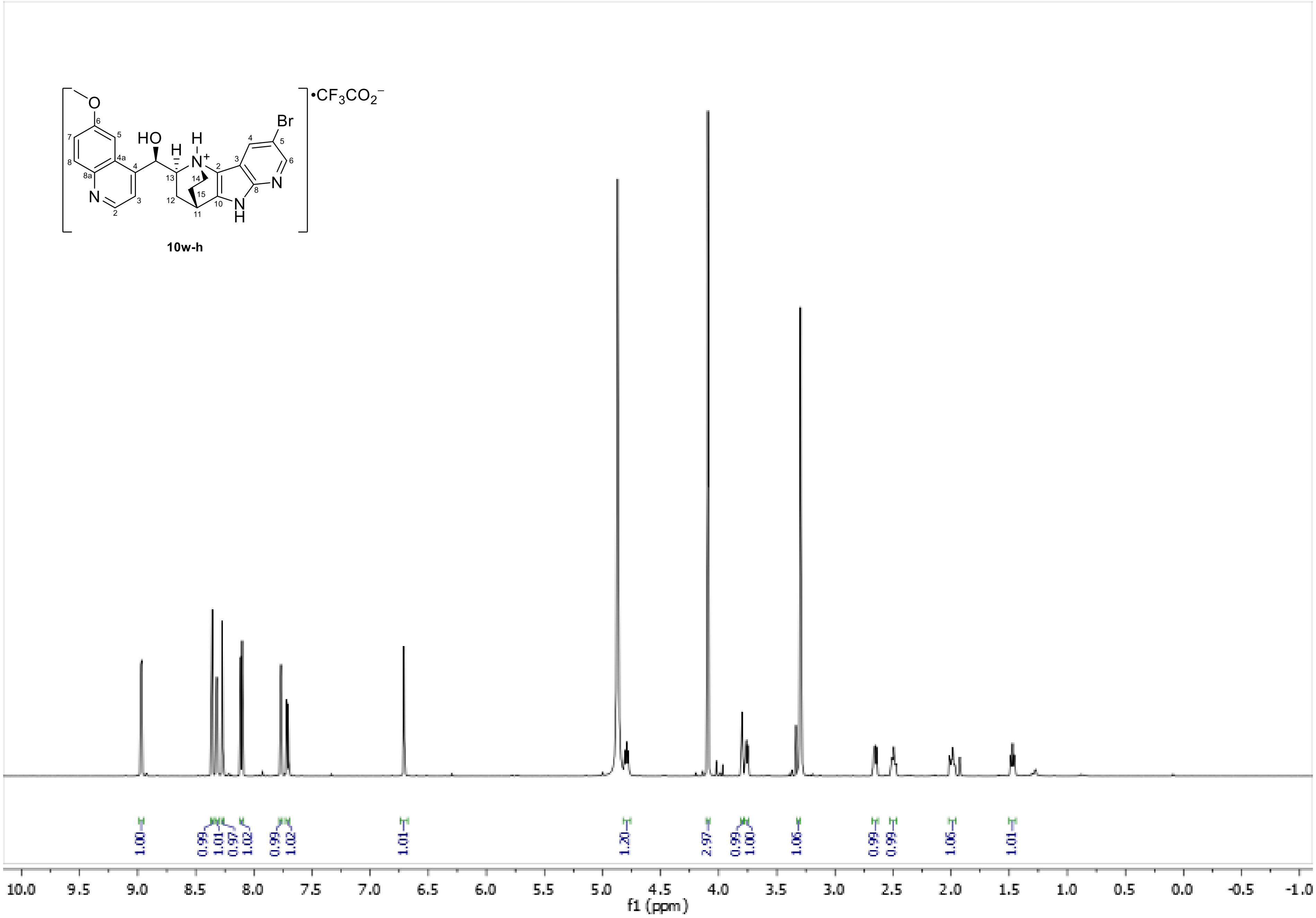
•CF3CO2−
Br
HO
10w-h
1.00
0.99
1.01
0.97
1.02
0.99
1.02
1.01
1.20
2.97
0.99
1.00
1.06
0.99
0.99
1.06
1.01
10.0
9.5
9.0
8.5
8.0
7.5
7.0
6.5
6.0
5.5
5.0
4.5
4.0
3.5
3.0
2.5
2.0
1.5
1.0
0.5
0.0
-0.5
-1.0
f1 (ppm)

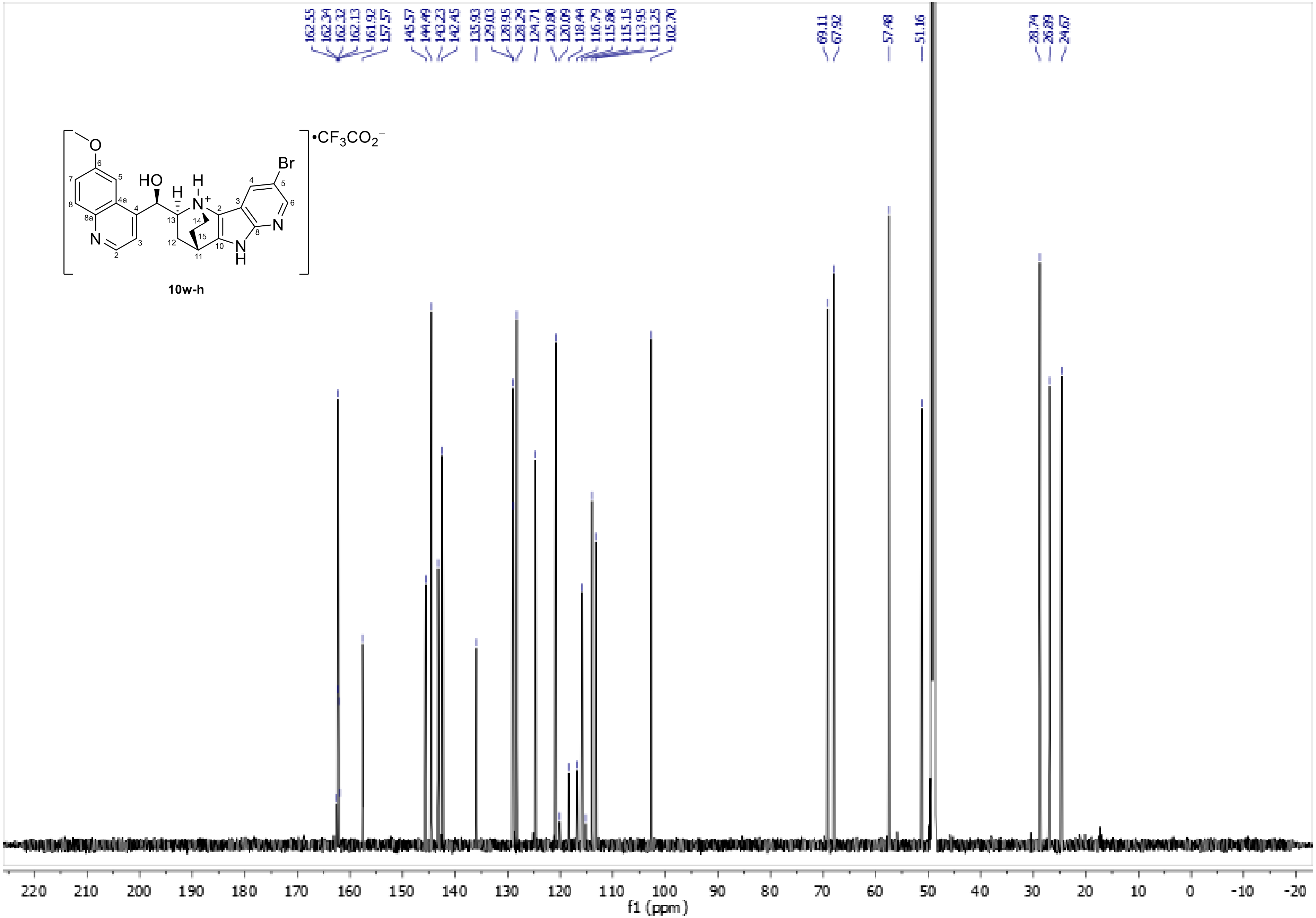
162.55
162.34
162.32
162.13
161.92
157.57
145.57
144.49
143.23
142.45
135.93
129.03
128.95
128.29
124.71
120.80
120.09
118.44
116.79
115.86
115.15
113.95
113.25
102.70
69.11
67.92
57.48
51.16
28.74
26.89
24.67
•CF3CO2−
Br
HO
H
H
N+
N
N
NH
O
10w-h
f1 (ppm)

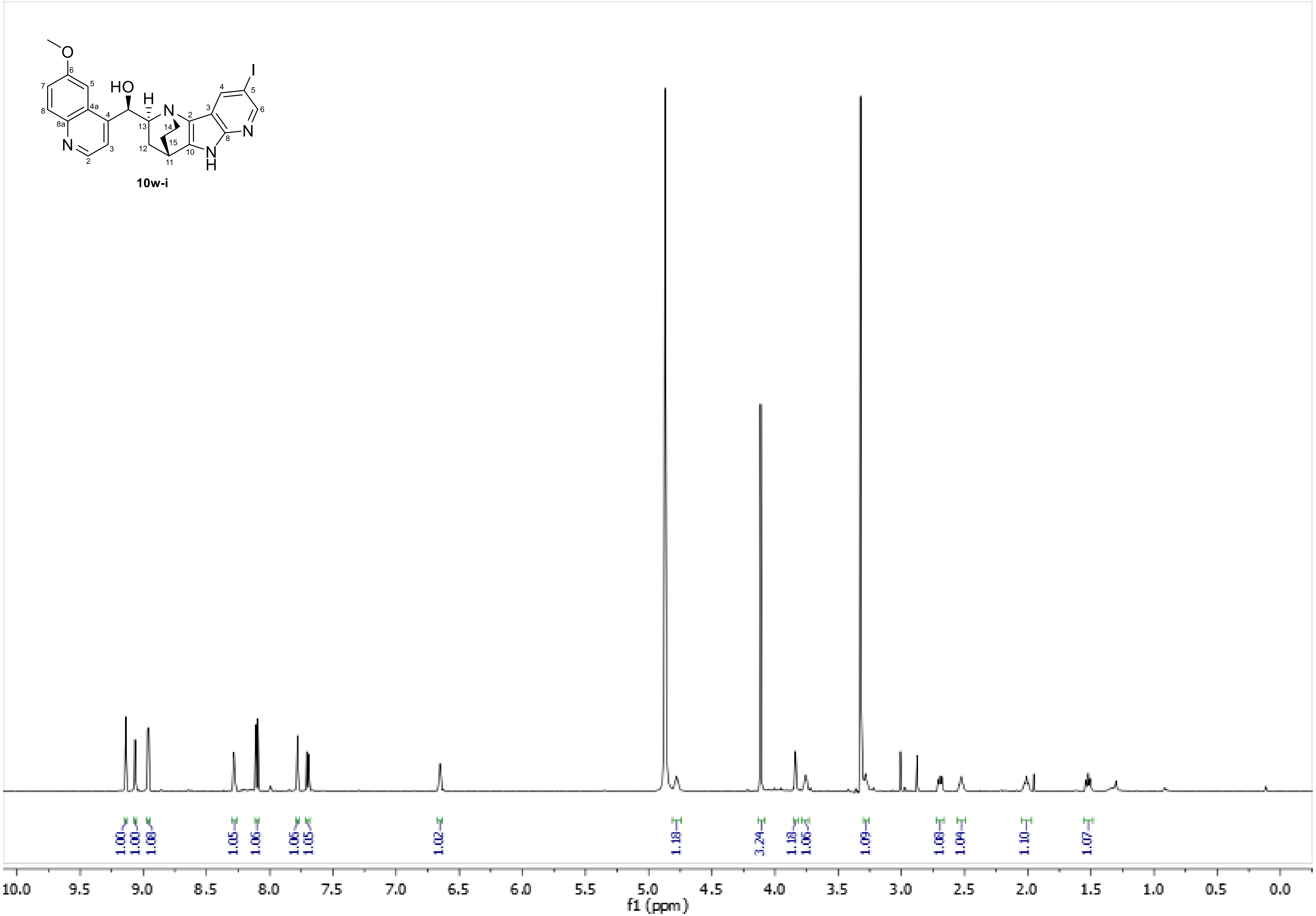

10w-i
1.00
1.00
1.08
1.05
1.06
1.06
1.05
1.02
1.18
3.24
1.18
1.06
1.09
1.08
1.04
1.10
1.07
10.0 9.5 9.0 8.5 8.0 7.5 7.0 6.5 6.0 5.5 5.0 4.5 4.0 3.5 3.0 2.5 2.0 1.5 1.0 0.5 0.0
f1 (ppm)

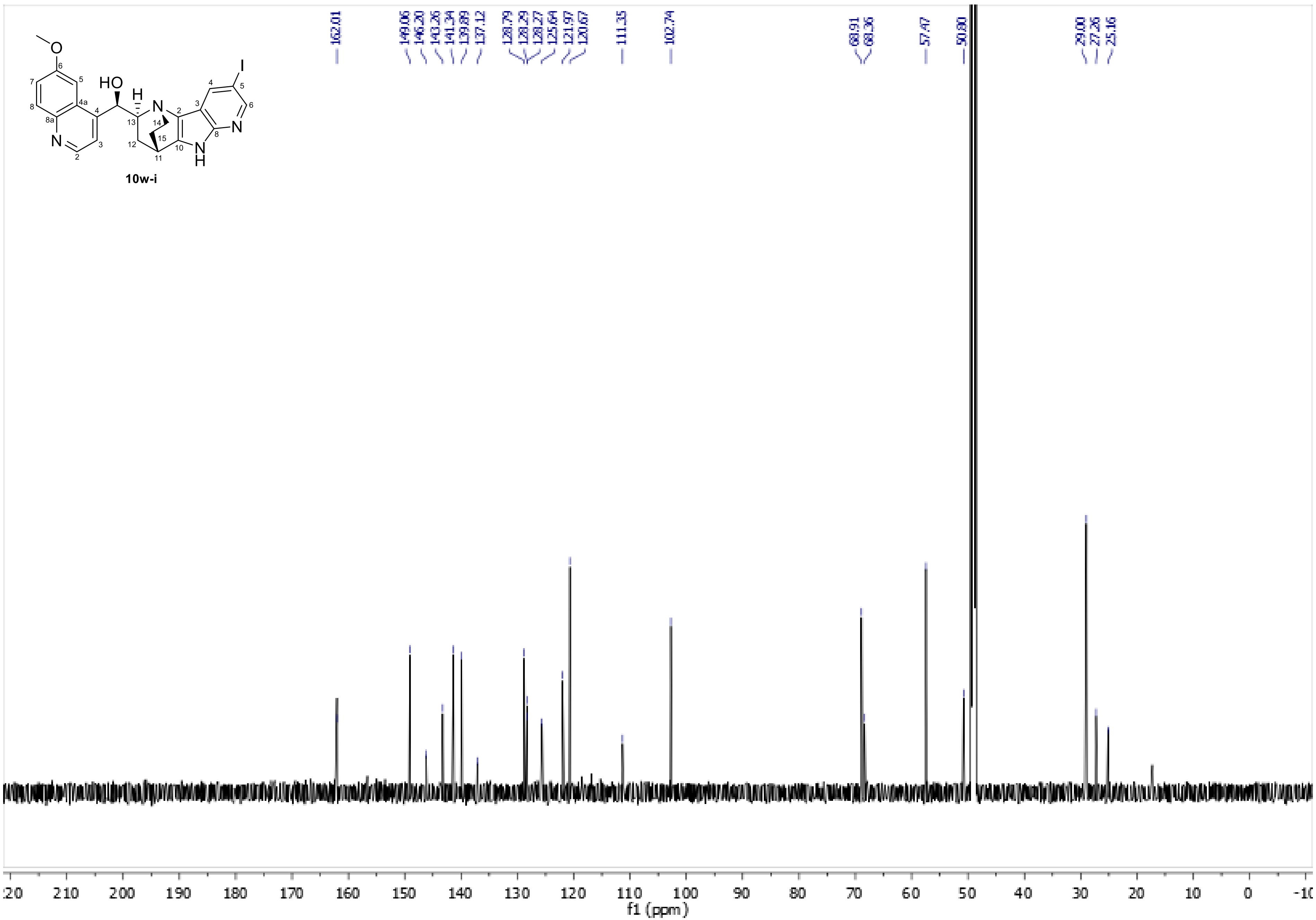
10w-i
162.01
149.06
146.20
143.26
141.34
139.89
137.12
128.79
128.29
128.27
125.64
121.97
120.67
111.35
102.74
68.91
68.36
57.47
50.80
29.00
27.26
25.16
f1 (ppm)

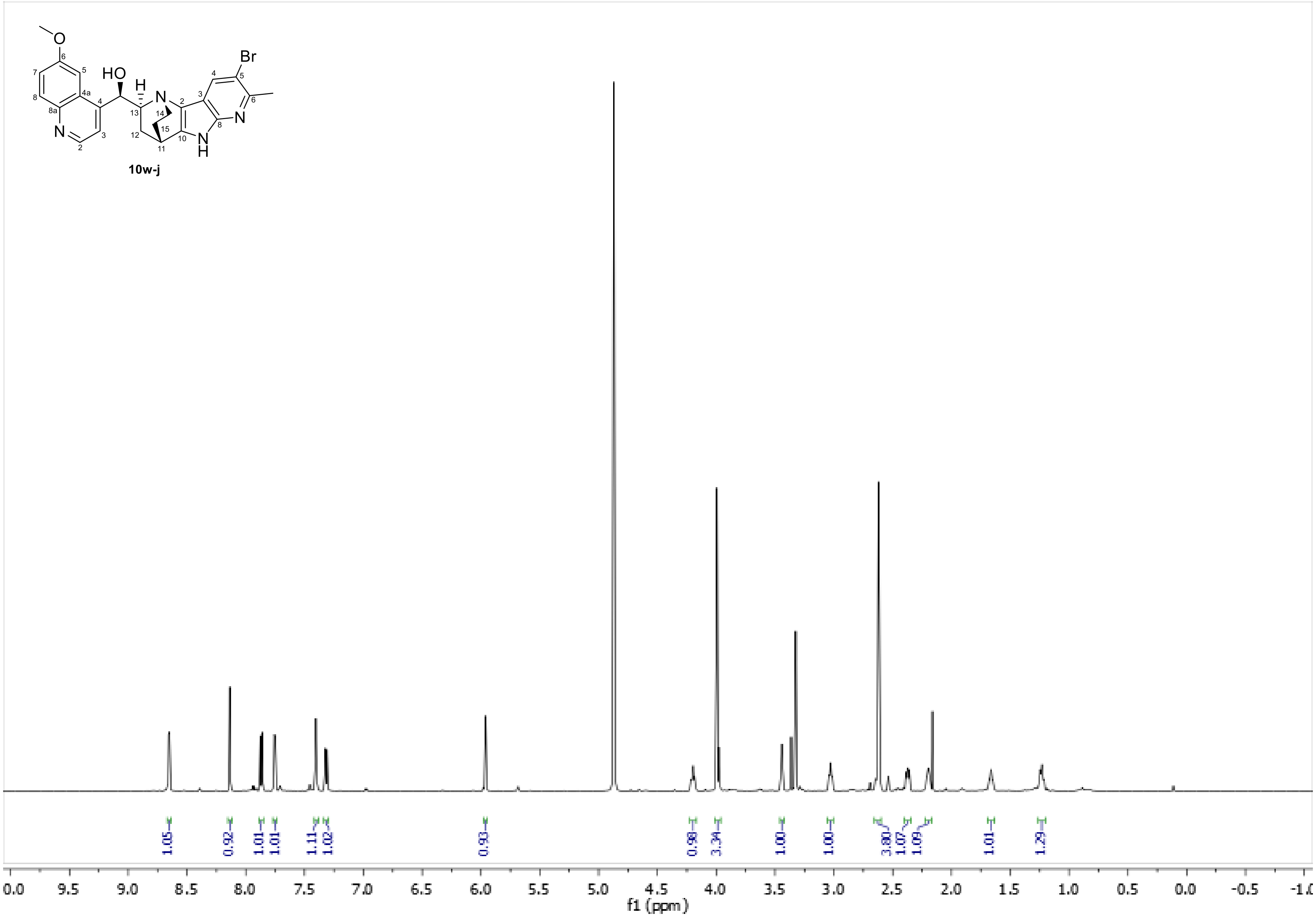
10w-j
1.05
0.92
1.01
1.01
1.11
1.02
0.93
0.98
3.34
1.00
1.00
3.80
1.07
1.09
1.01
1.29
f1 (ppm)

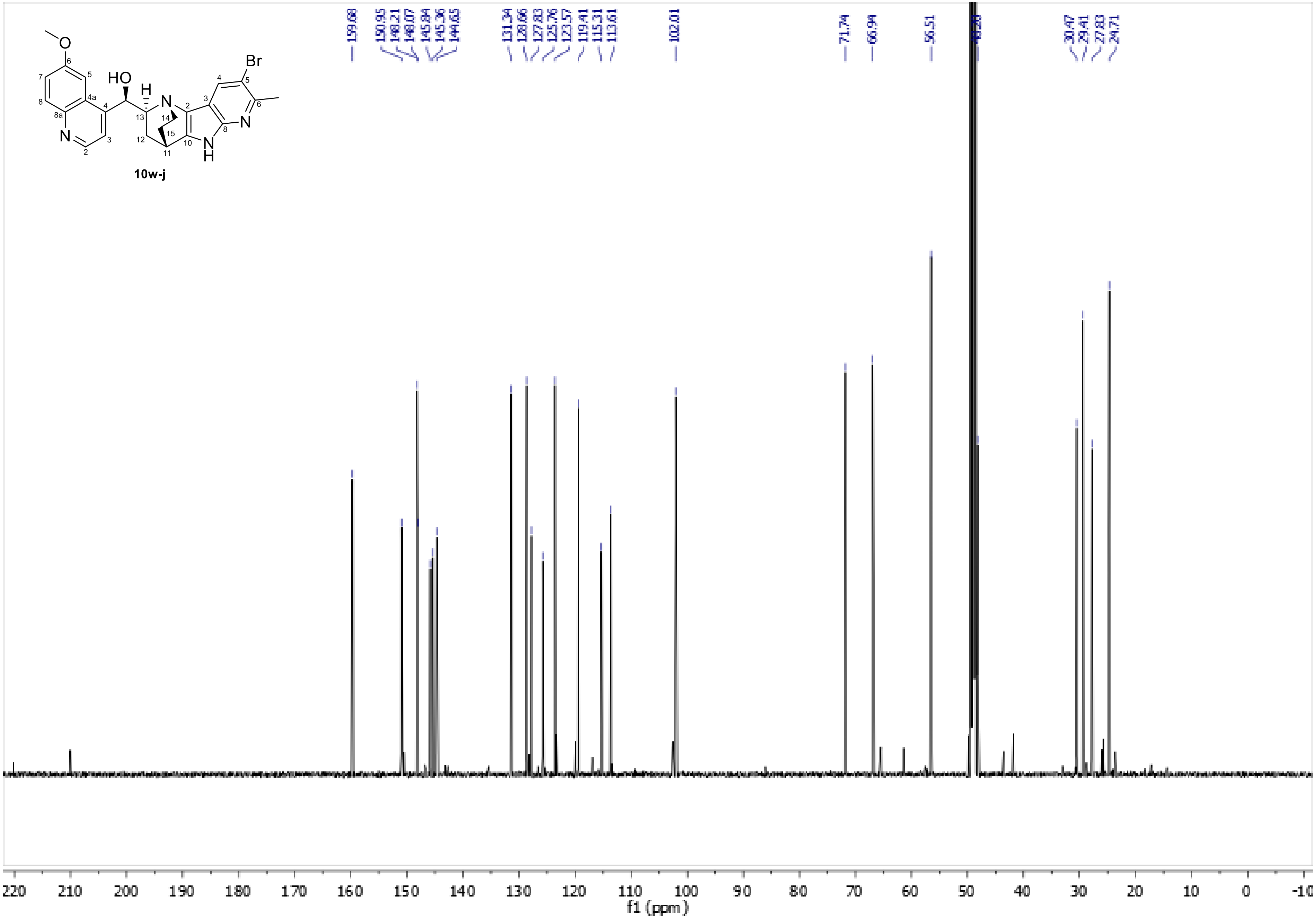

HO
Br
10w-j
159.68
150.95
148.21
148.07
145.84
145.36
144.65
131.34
128.66
127.83
125.76
123.57
119.41
115.31
113.61
102.01
71.74
66.94
56.51
30.47
29.41
27.83
24.71
f1 (ppm)

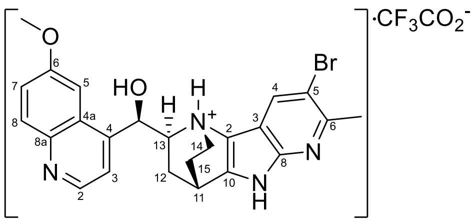
·CF3CO2-
HO
H
N+
Br
N
N
H
N

10w-j

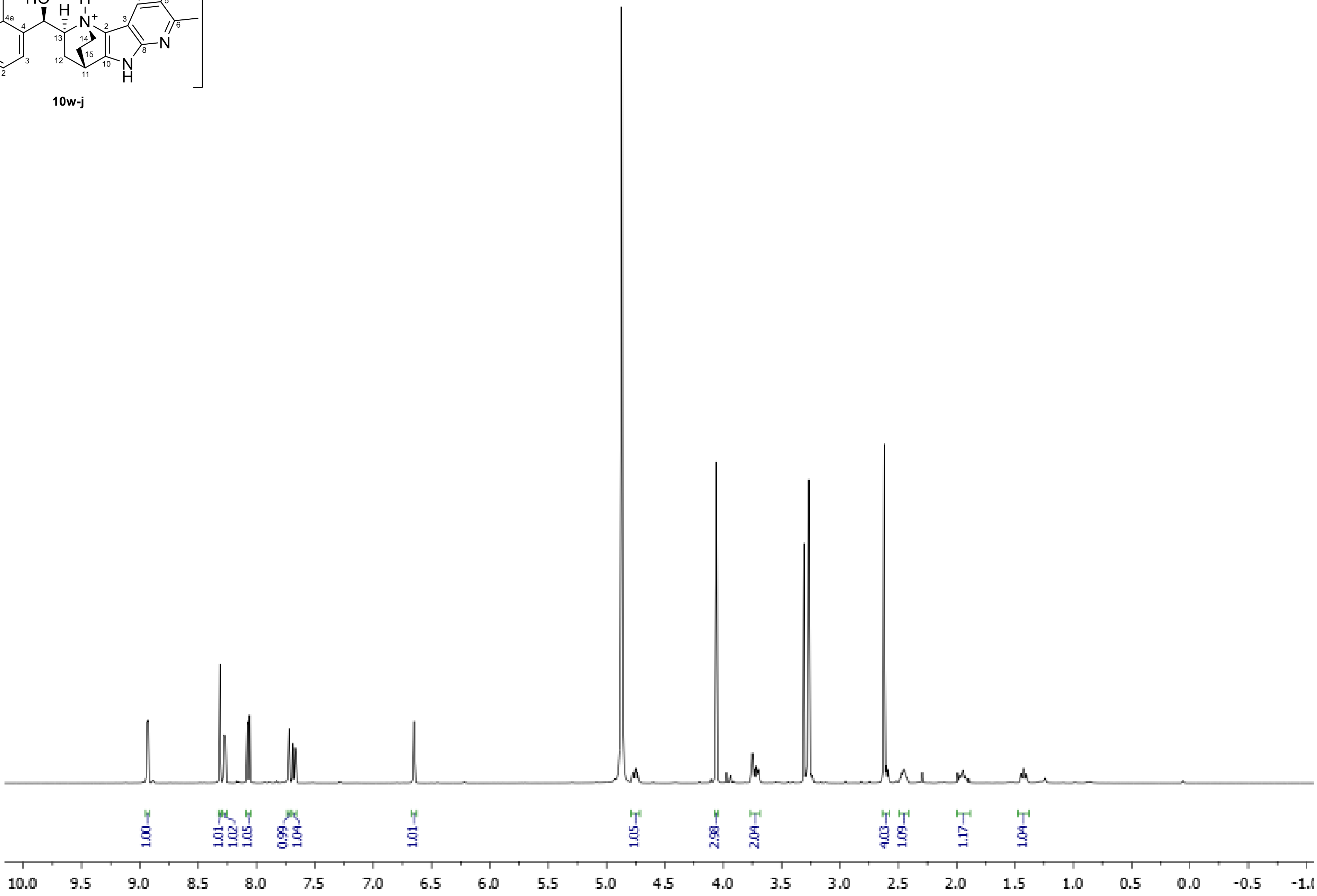
1.00
1.01
1.02
1.05
0.99
1.04
1.01
1.05
2.98
2.04
4.03
1.09
1.17
1.04
10.0 9.5 9.0 8.5 8.0 7.5 7.0 6.5 6.0 5.5 5.0 4.5 4.0 3.5 3.0 2.5 2.0 1.5 1.0 0.5 0.0 -0.5

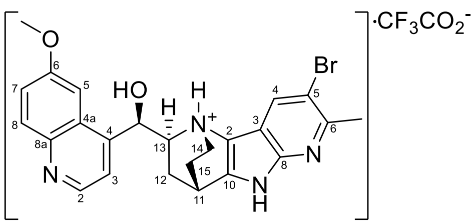
·$CF_3CO_2^-$
Br
HO
H
N+
NH
N

10w-j

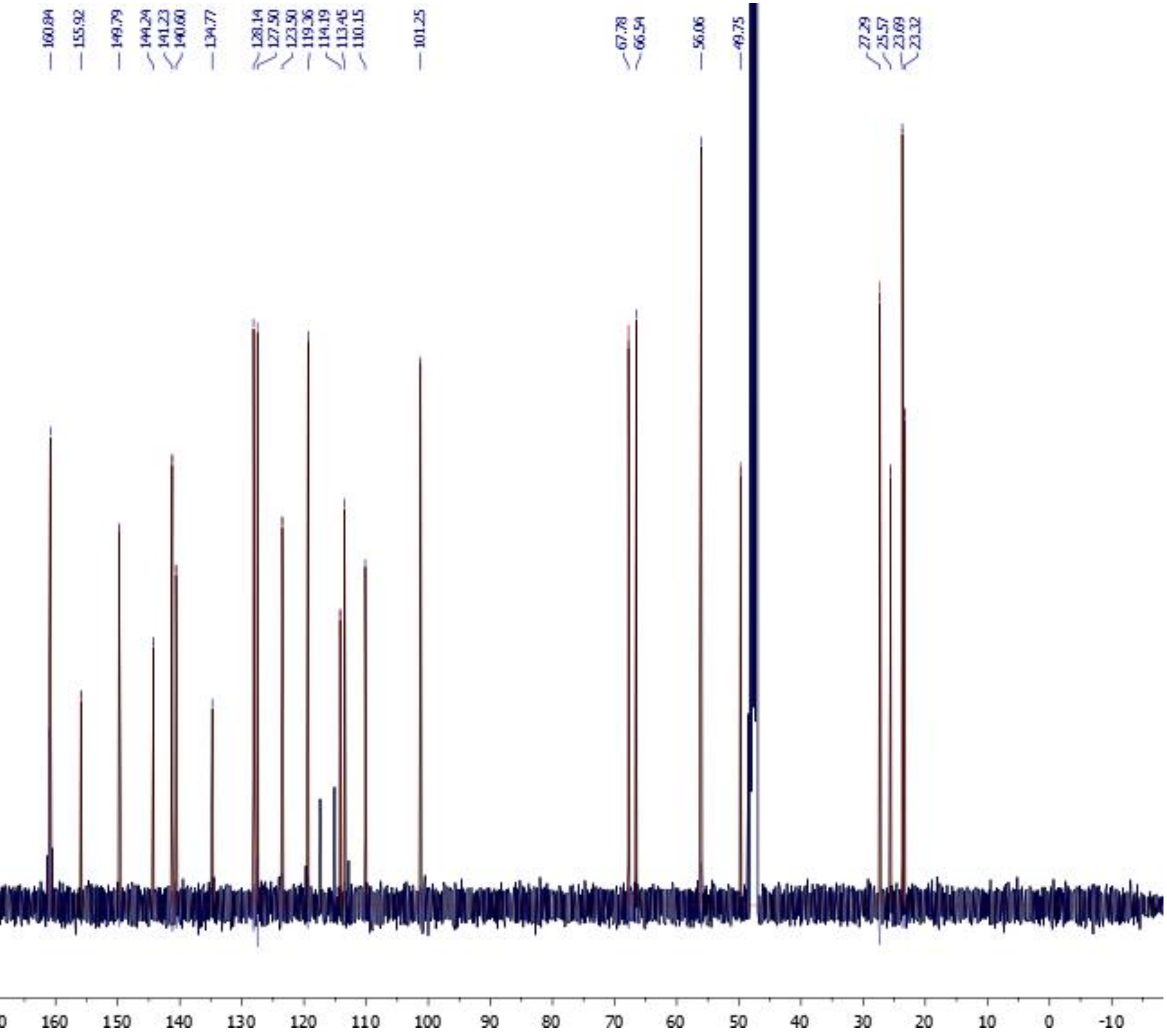
160.84
155.92
149.79
144.24
141.23
140.60
134.77
128.14
127.50
123.50
119.36
114.19
113.45
110.15
101.25
67.78
66.54
56.06
49.75
27.29
25.57
23.69
23.32
210 200 190 180 170 160 150 140 130 120 110 100 90 80 70 60 50 40 30 20 10 0 -10

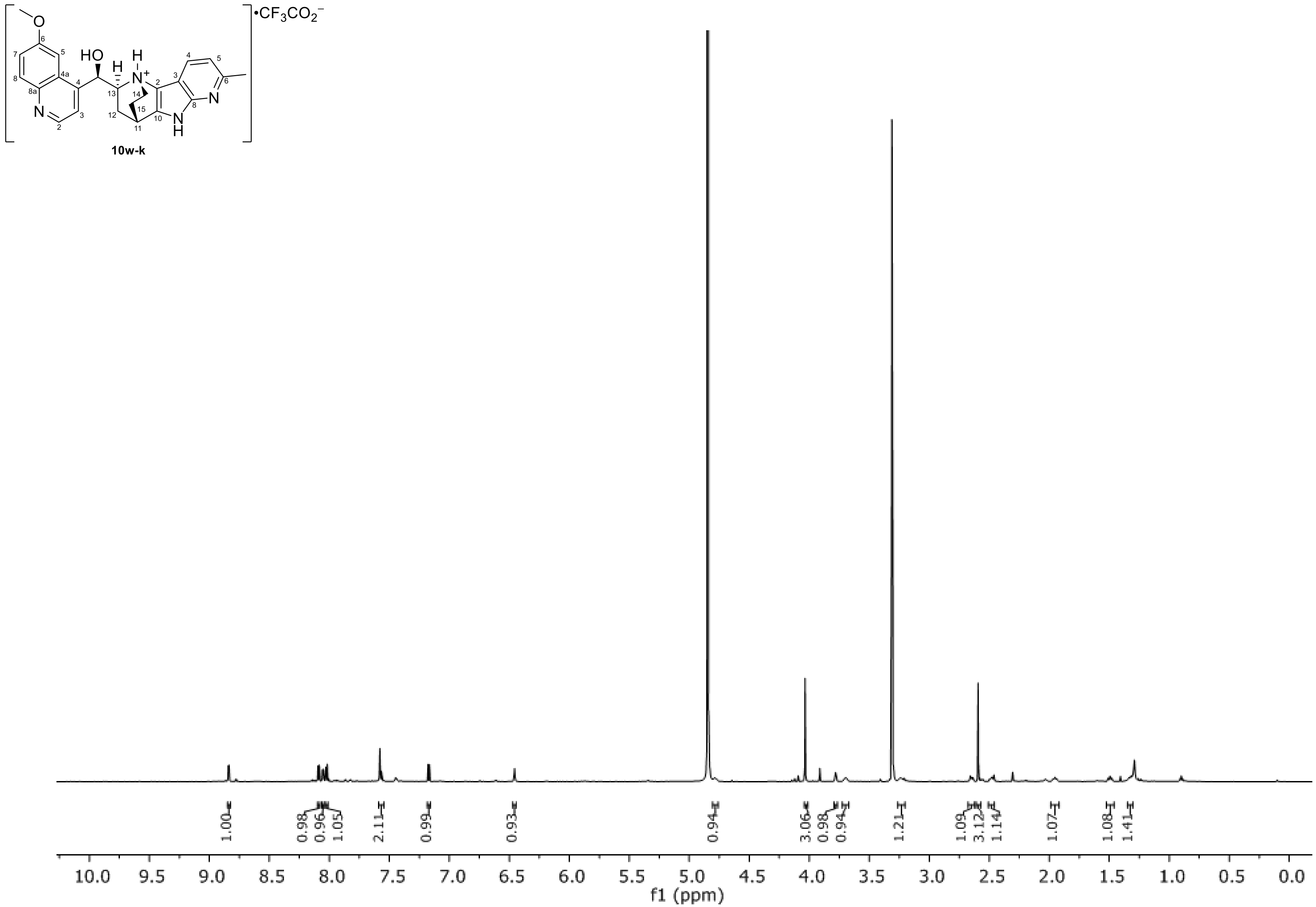
•CF3CO2−
10w-k
1.00
0.98
0.96
1.05
2.11
0.99
0.93
0.94
3.06
0.98
0.94
1.21
1.09
3.12
1.14
1.07
1.08
1.41
f1 (ppm)

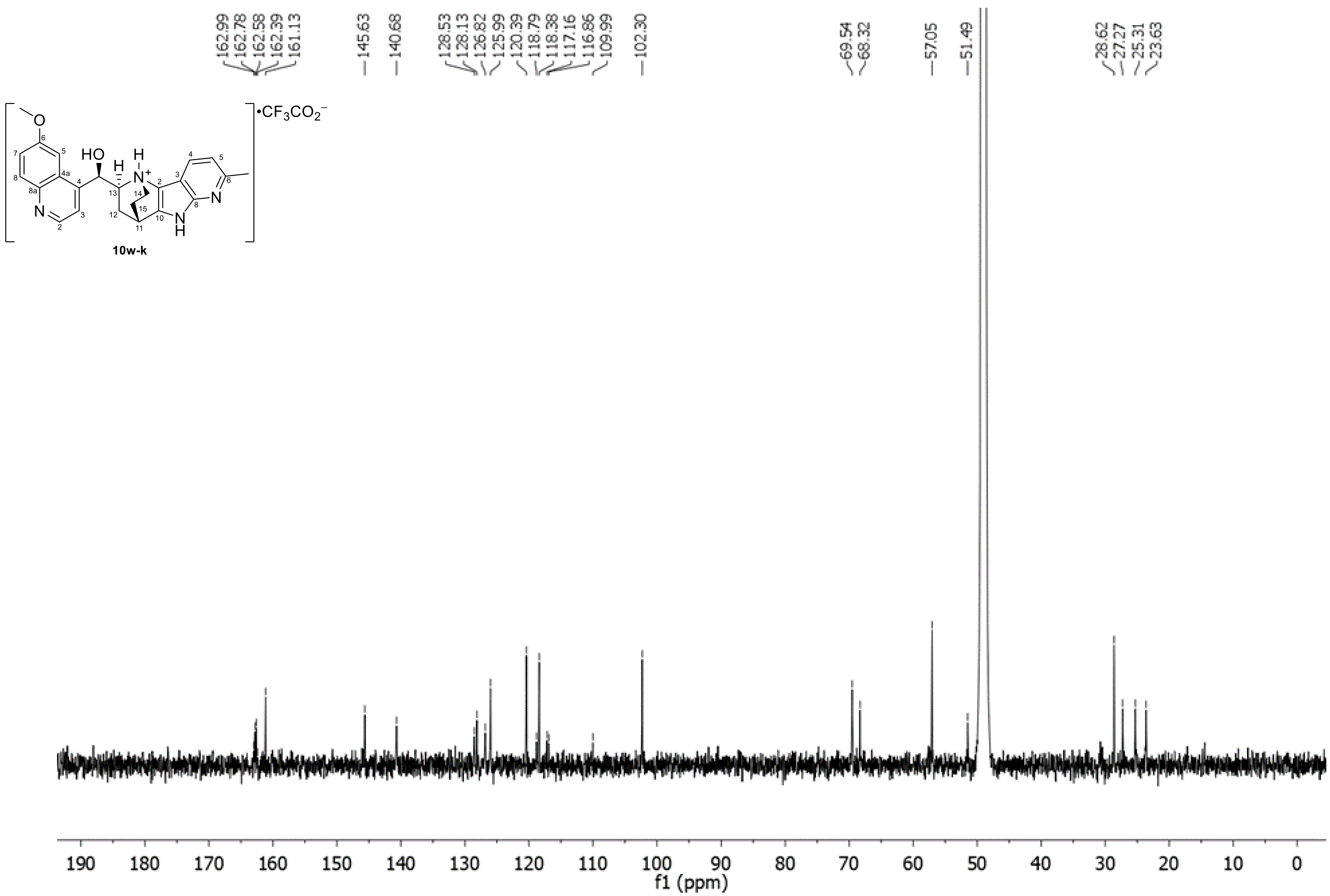
162.99
162.78
162.58
162.39
161.13
145.63
140.68
128.53
128.13
126.82
125.99
120.39
118.79
118.38
117.16
116.86
109.99
102.30
69.54
68.32
57.05
51.49
28.62
27.27
25.31
23.63
•CF3CO2−
HO
10w-k
f1 (ppm)

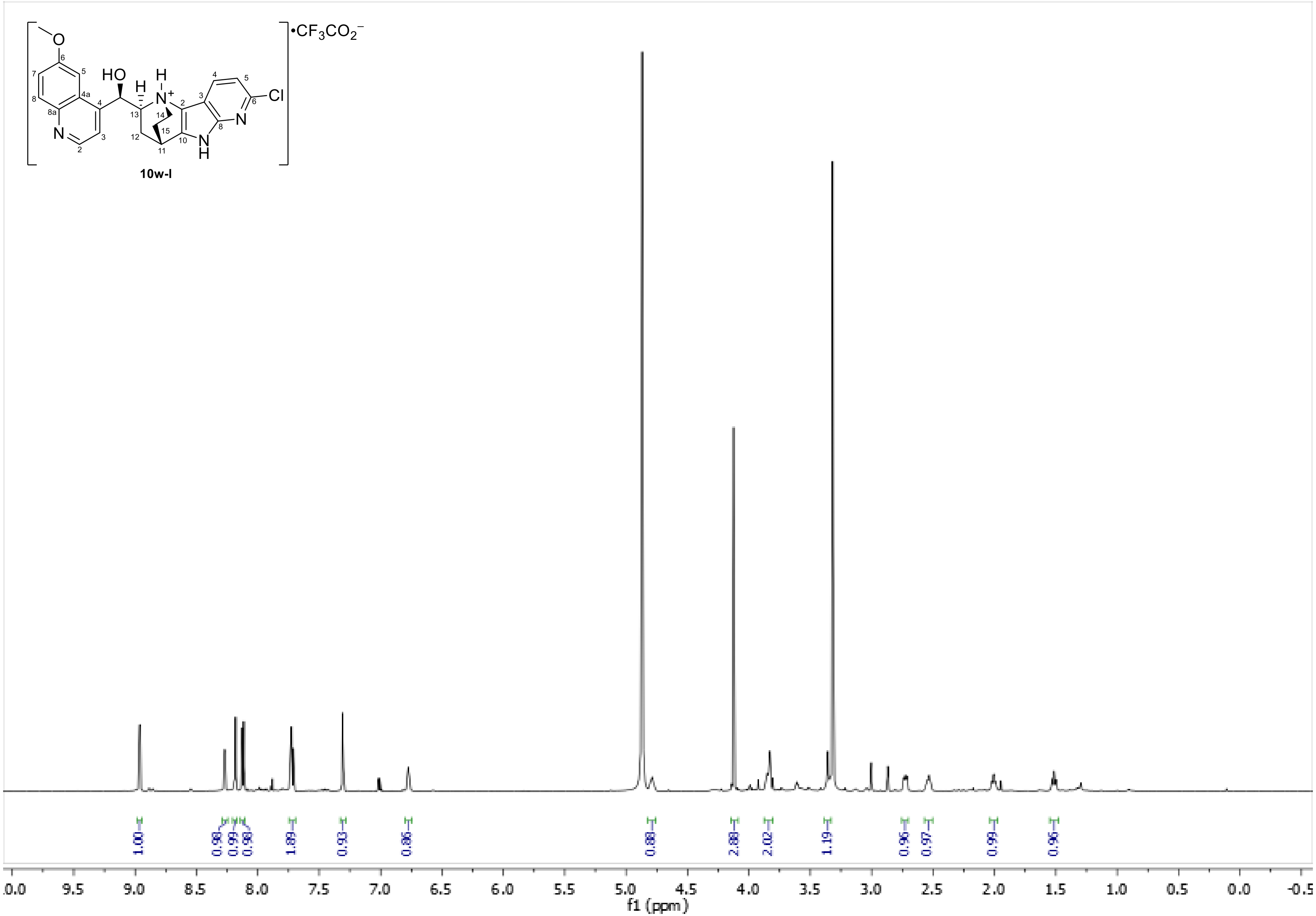
•CF3CO2−
HO
H
N+
Cl
N
NH
10w-I
1.00
0.98
0.99
0.98
1.89
0.93
0.86
0.88
2.88
2.02
1.19
0.96
0.97
0.99
0.96
10.0
9.5
9.0
8.5
8.0
7.5
7.0
6.5
6.0
5.5
5.0
4.5
4.0
3.5
3.0
2.5
2.0
1.5
1.0
0.5
0.0
-0.5
f1 (ppm)

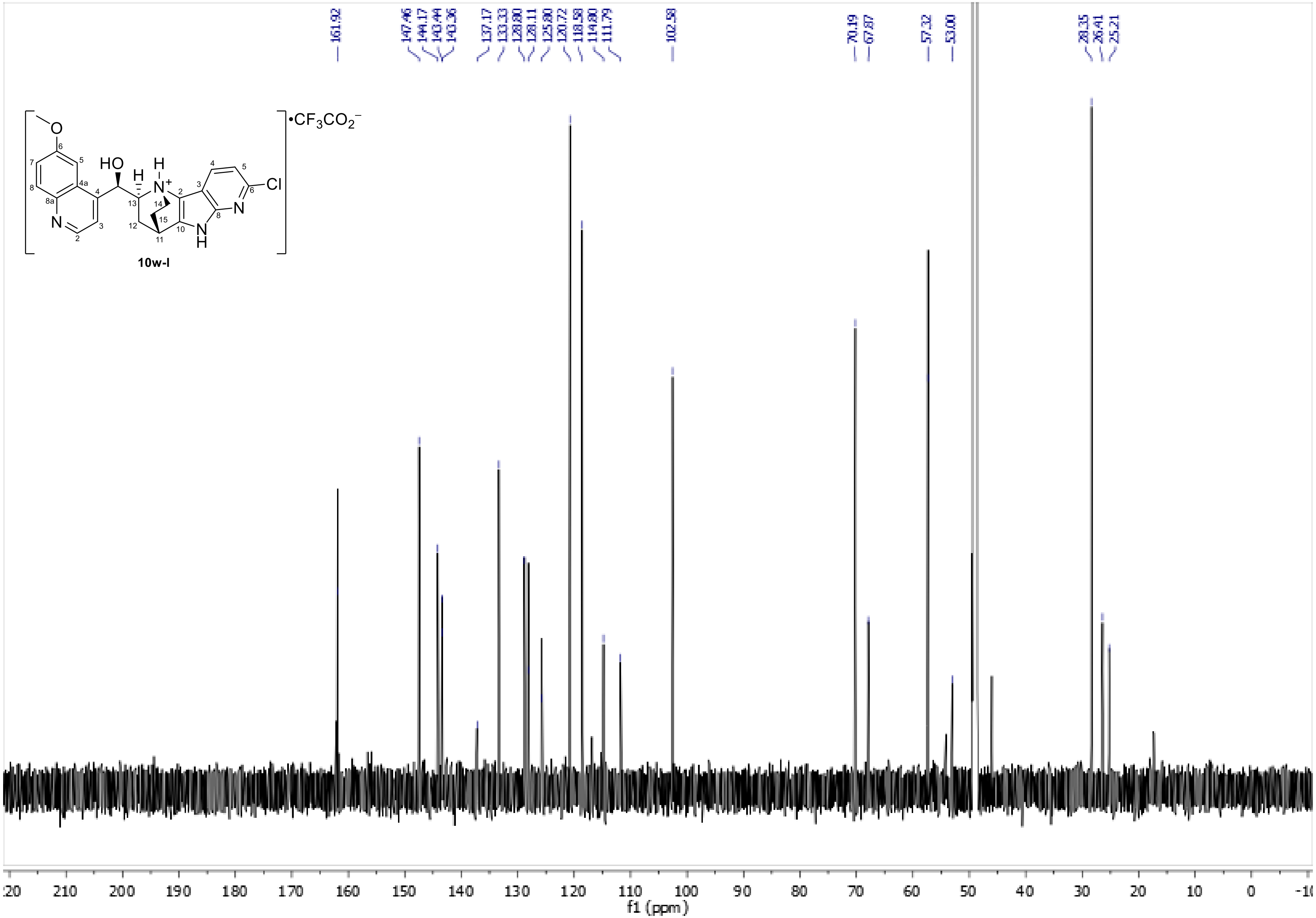
161.92
147.46
144.17
143.44
143.36
137.17
133.33
128.80
128.11
125.80
120.72
118.58
114.80
111.79
102.58
70.19
67.87
57.32
53.00
28.35
26.41
25.21
•CF3CO2–
HO
H
N+
Cl
N
NH
O
10w-I
f1 (ppm)

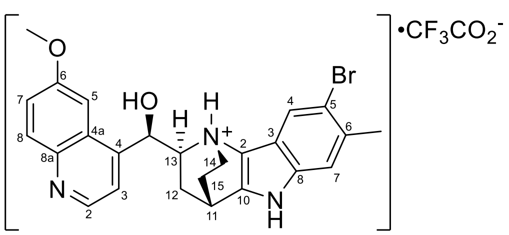
•CF3CO2-
Br
HO
H
N+
N
H
N

S-1

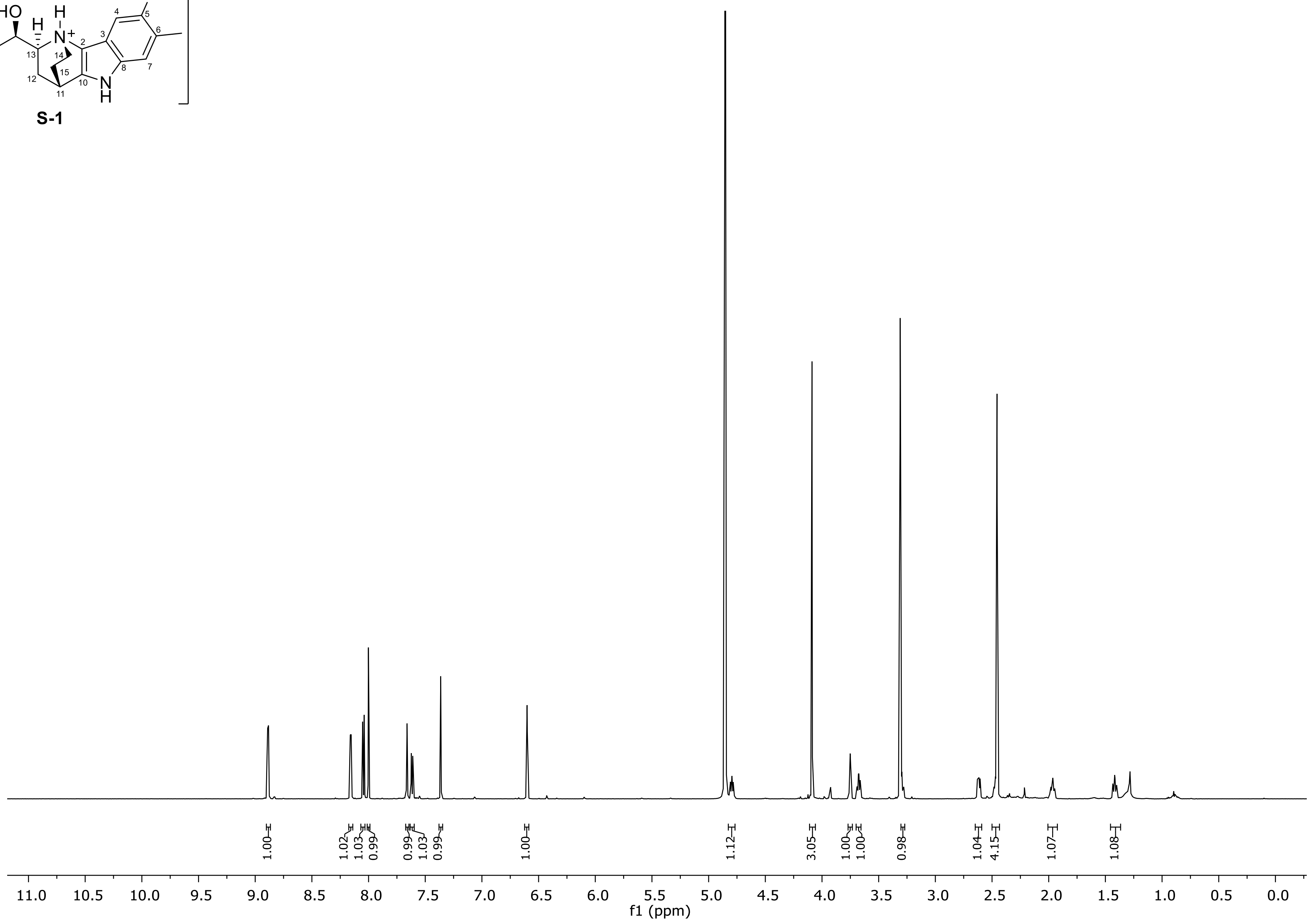
1.00
1.02
1.03
0.99
0.99
1.03
0.99
1.00
1.12
3.05
1.00
1.00
0.98
1.04
4.15
1.07
1.08
11.0 10.5 10.0 9.5 9.0 8.5 8.0 7.5 7.0 6.5 6.0 5.5 5.0 4.5 4.0 3.5 3.0 2.5 2.0 1.5 1.0 0.5 0.0
f1 (ppm)

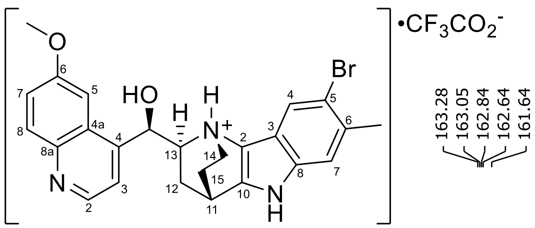
•$CF_3CO_2^-$
Br
HO
H
N+
NH

S-1

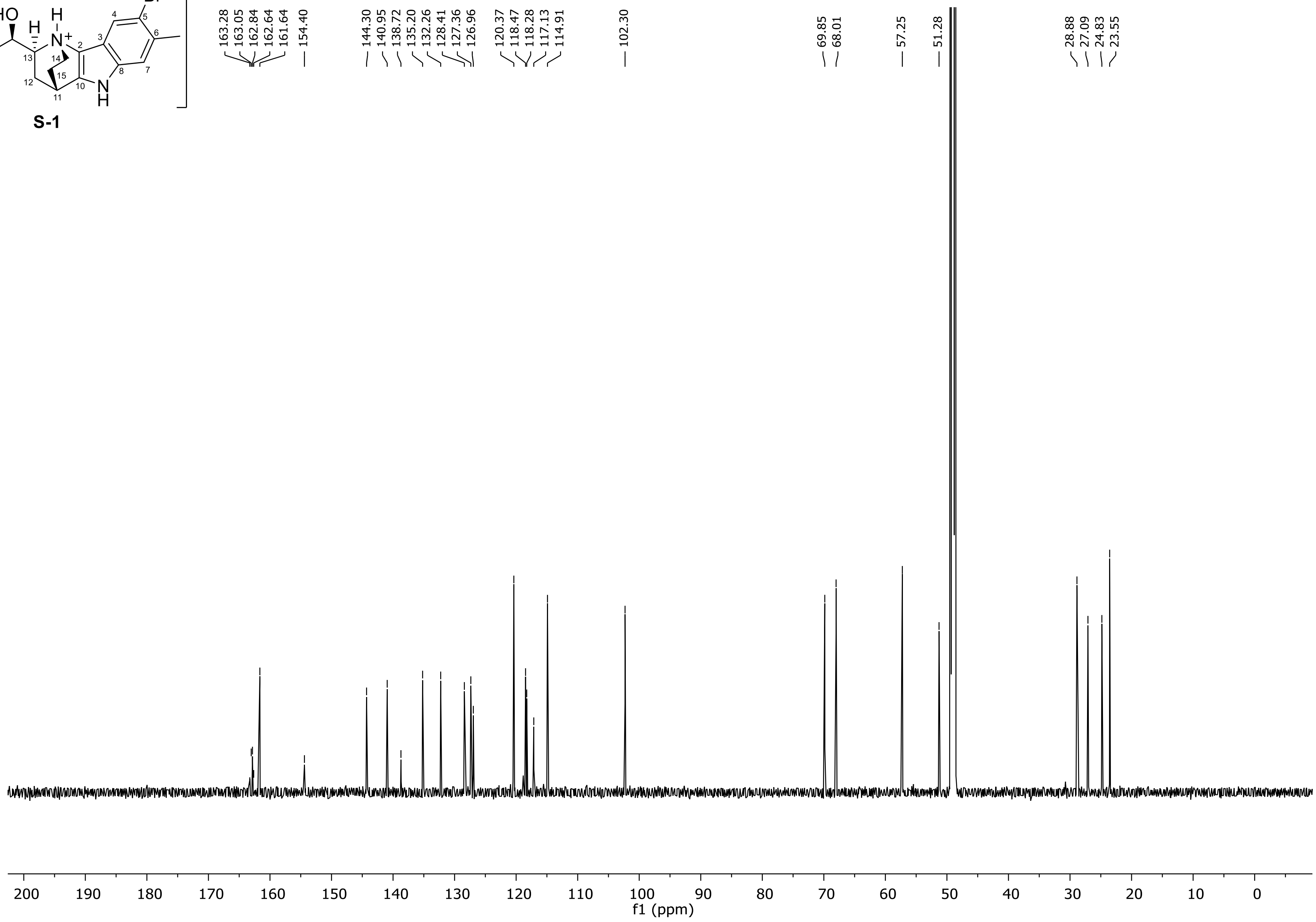
163.28
163.05
162.84
162.64
161.64
154.40
144.30
140.95
138.72
135.20
132.26
128.41
127.36
126.96
120.37
118.47
118.28
117.13
114.91
102.30
69.85
68.01
57.25
51.28
28.88
27.09
24.83
23.55
200 190 180 170 160 150 140 130 120 110 100 90 80 70 60 50 40 30 20 10 0
f1 (ppm)

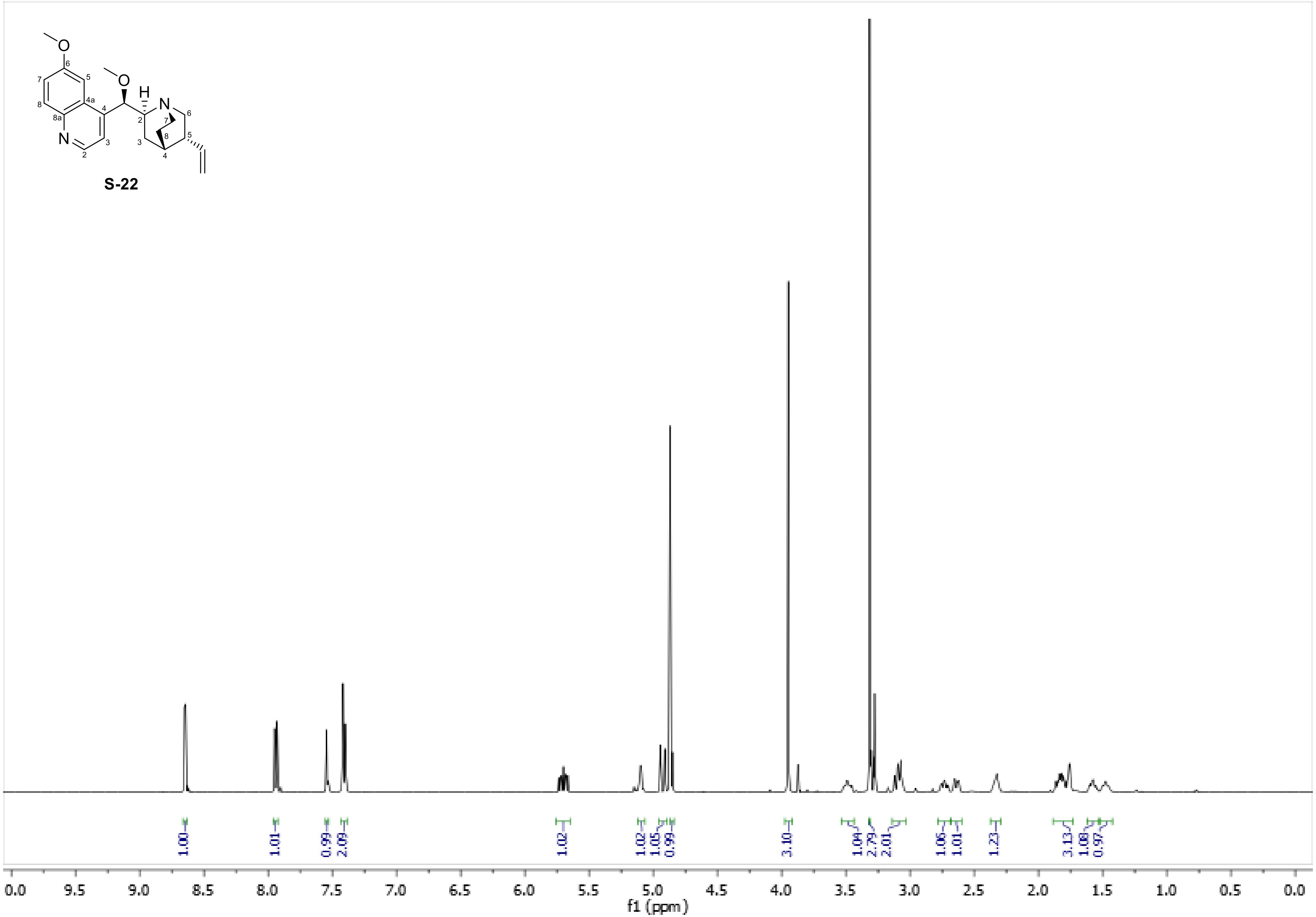
S-22
1.00
1.01
0.99
2.09
1.02
1.02
1.05
0.99
3.10
1.04
2.79
2.01
1.06
1.01
1.23
3.13
1.08
0.97
f1 (ppm)

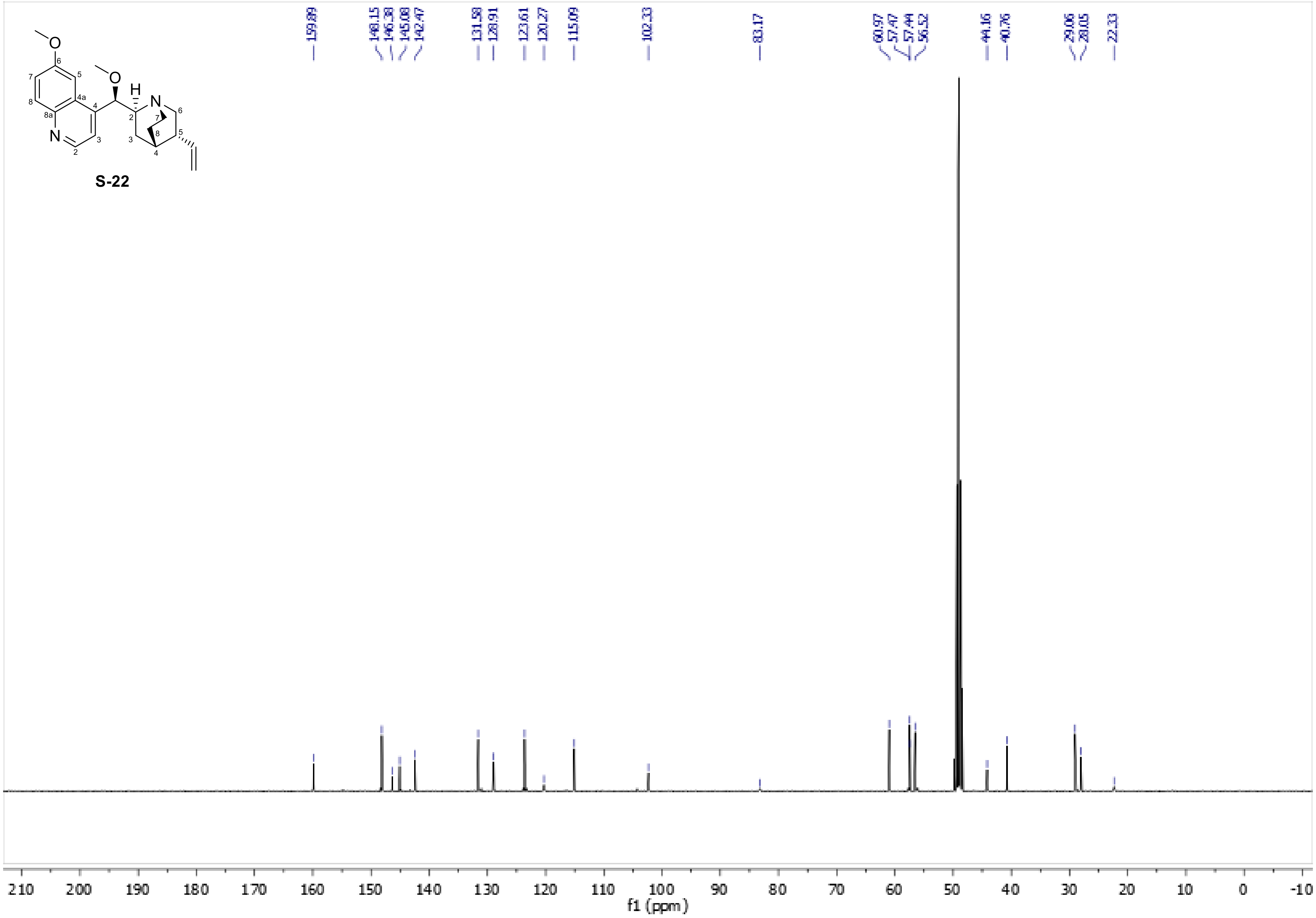
S-22
159.89
148.15
146.38
145.08
142.47
131.58
128.91
123.61
120.27
115.09
102.33
83.17
60.97
57.47
57.44
56.52
44.16
40.76
29.06
28.05
22.33
210 200 190 180 170 160 150 140 130 120 110 100 90 80 70 60 50 40 30 20 10 0 -10
f1 (ppm)

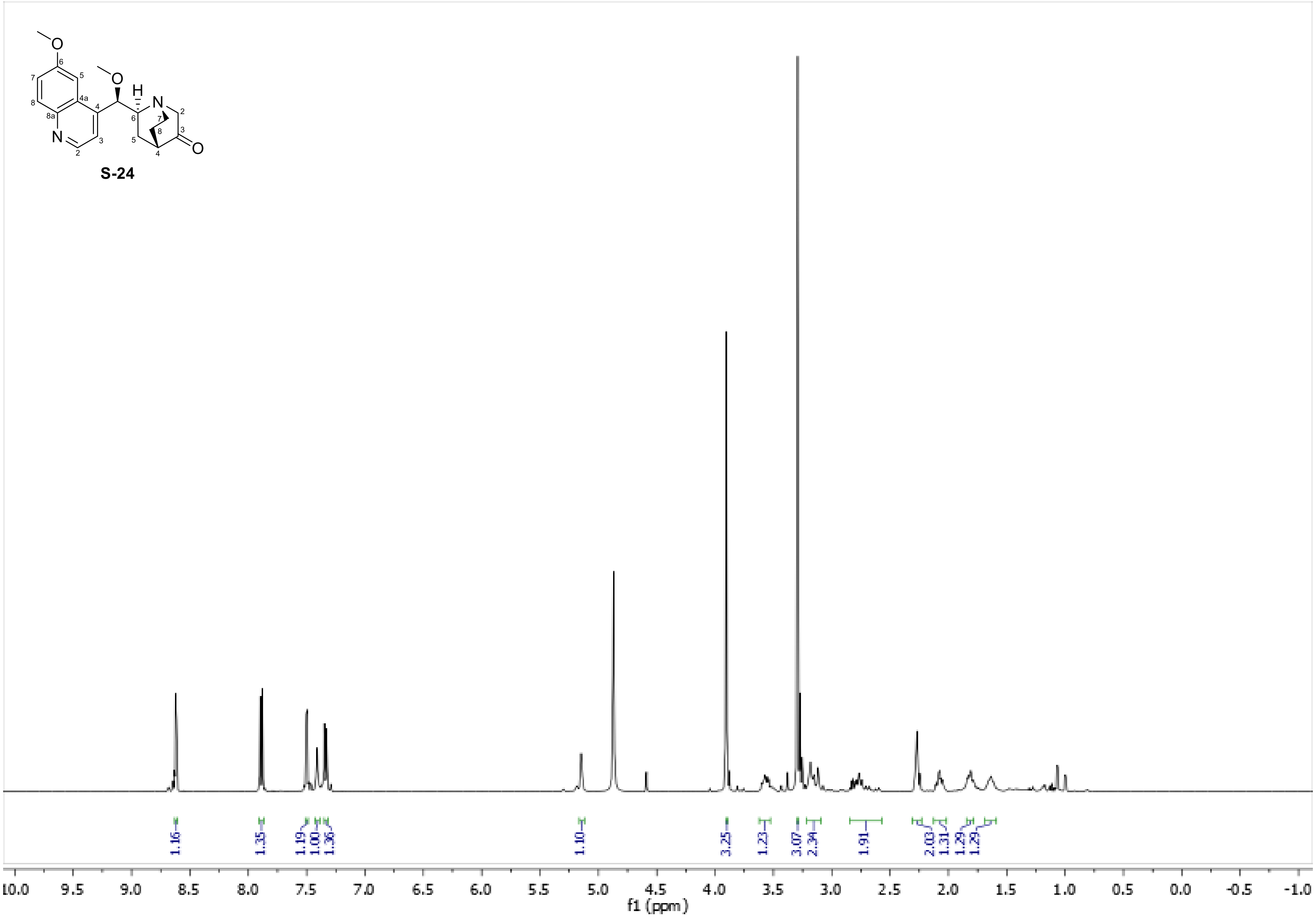
S-24
1.16
1.35
1.19
1.00
1.36
1.10
3.25
1.23
3.07
2.34
1.91
2.03
1.31
1.29
1.29
10.0 9.5 9.0 8.5 8.0 7.5 7.0 6.5 6.0 5.5 5.0 4.5 4.0 3.5 3.0 2.5 2.0 1.5 1.0 0.5 0.0 -0.5 -1.0
f1 (ppm)

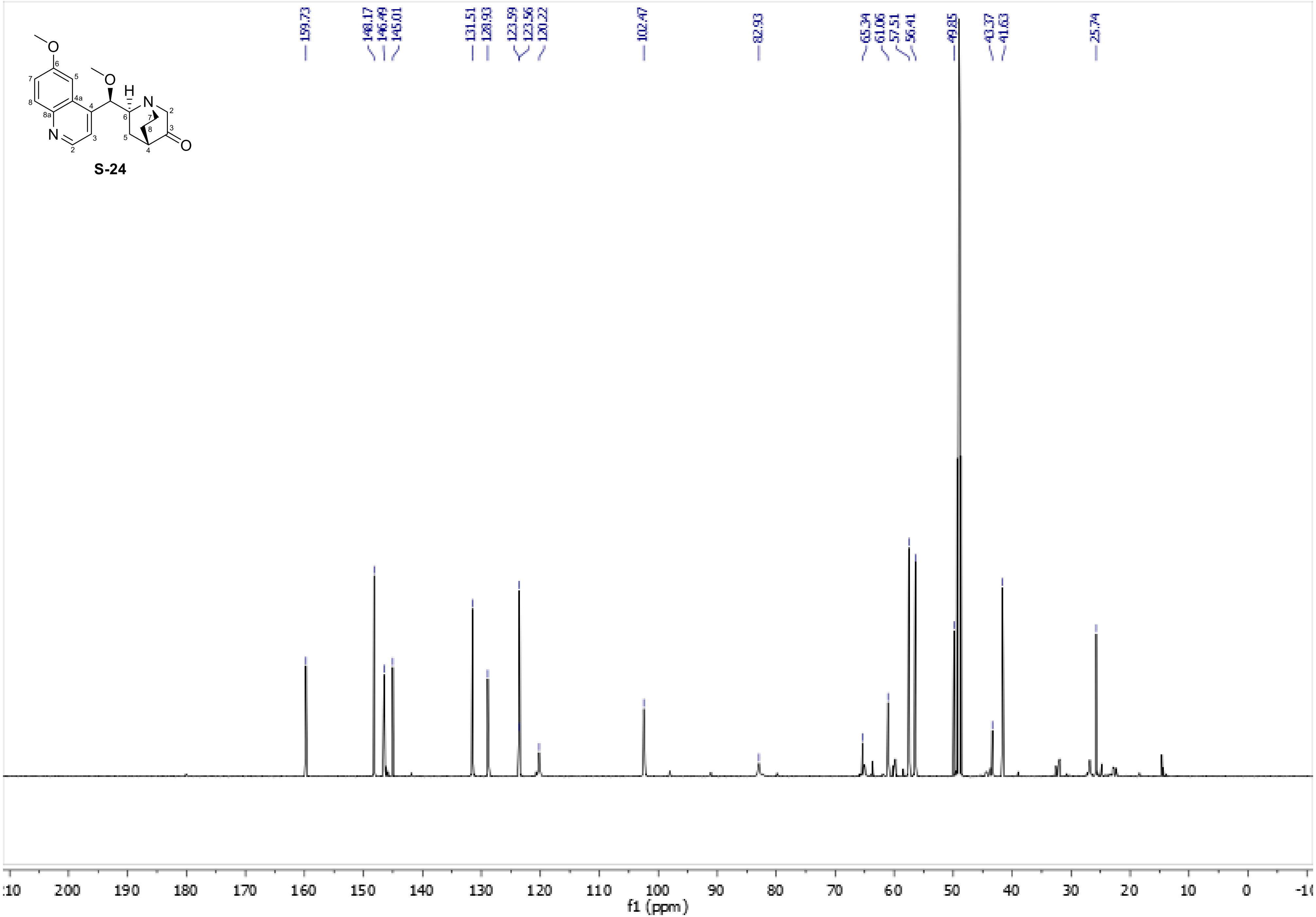
S-24
159.73
148.17
146.49
145.01
131.51
128.93
123.59
123.56
120.22
102.47
82.93
65.34
61.06
57.51
56.41
49.85
43.37
41.63
25.74
f1 (ppm)

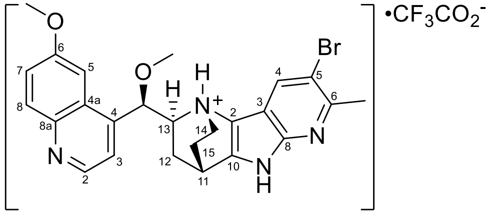
•$CF_3CO_2^-$
Br
N
NH
H
O

S-2

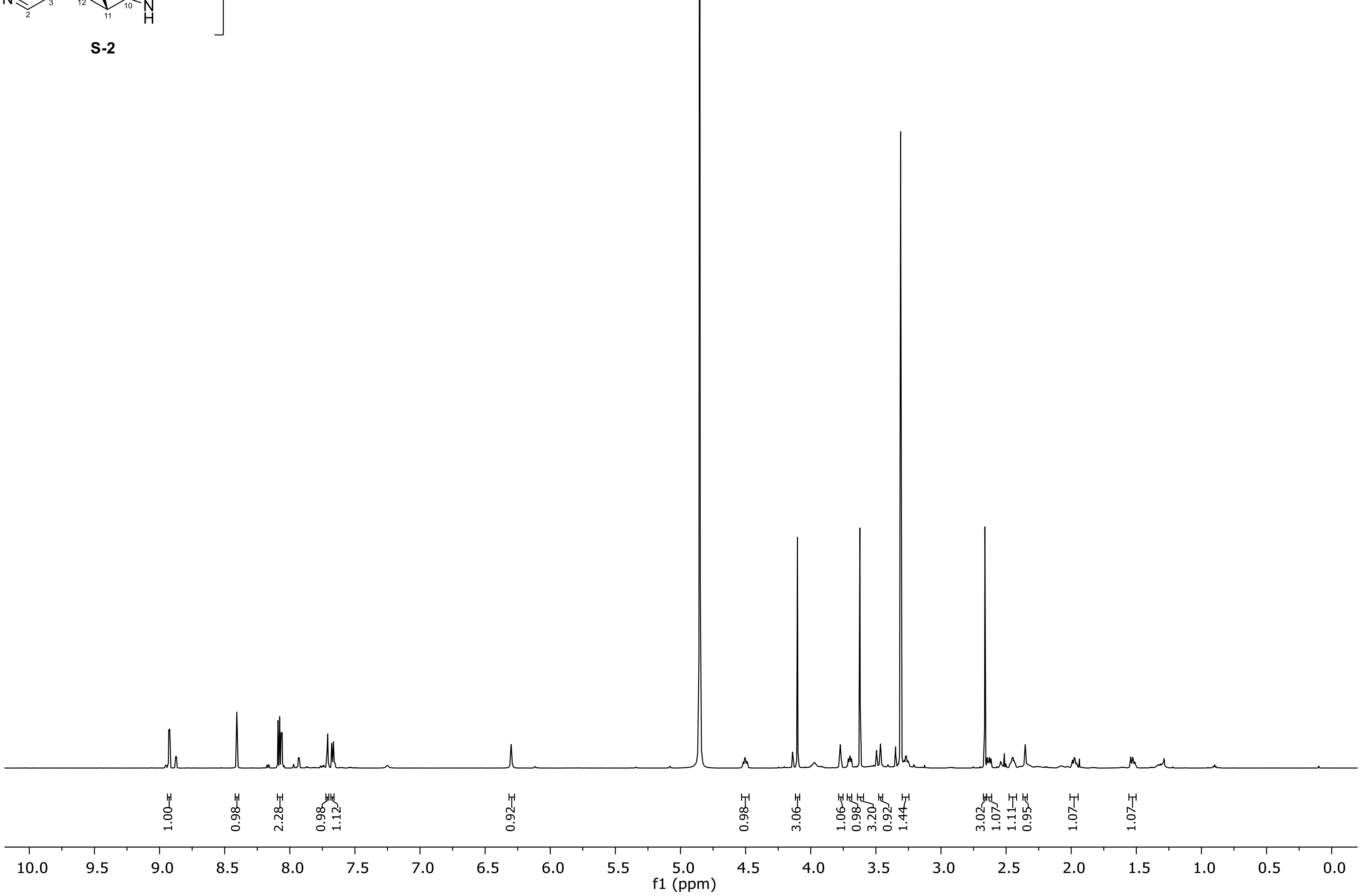
1.00
0.98
2.28
0.98
1.12
0.92
0.98
3.06
1.06
0.98
3.20
0.92
1.44
3.02
1.07
1.11
0.95
1.07
1.07
10.0 9.5 9.0 8.5 8.0 7.5 7.0 6.5 6.0 5.5 5.0 4.5 4.0 3.5 3.0 2.5 2.0 1.5 1.0 0.5 0.0
f1 (ppm)

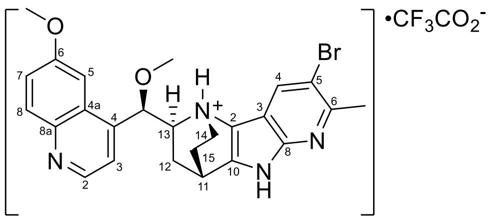
•$CF_3CO_2^-$
Br
N
NH
H
O

S-2

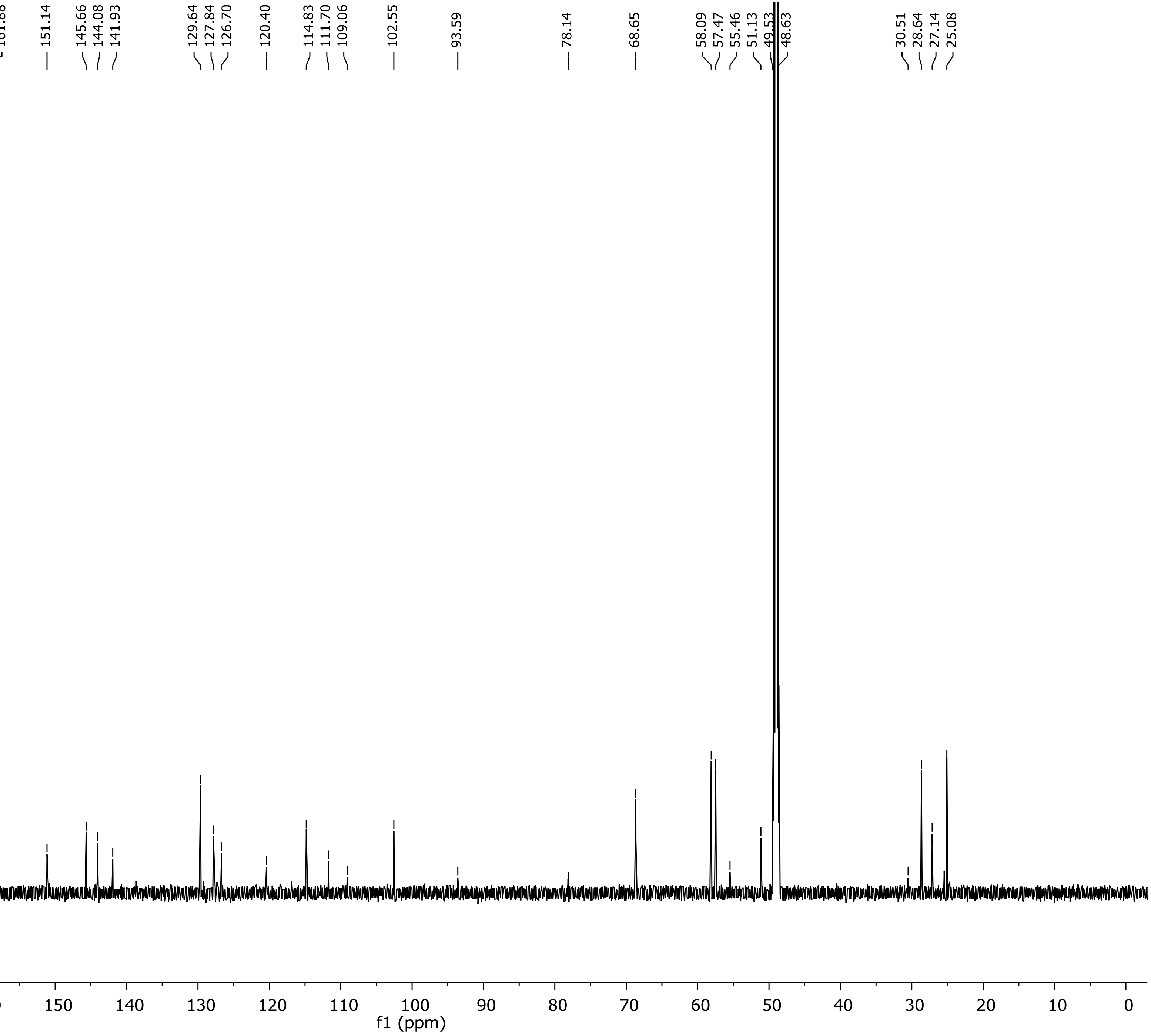
162.54
162.35
162.14
161.92
161.88
151.14
145.66
144.08
141.93
129.64
127.84
126.70
120.40
114.83
111.70
109.06
102.55
93.59
78.14
68.65
58.09
57.47
55.46
51.13
49.53
48.63
30.51
28.64
27.14
25.08
200 190 180 170 160 150 140 130 120 110 100 90 80 70 60 50 40 30 20 10 0
f1 (ppm)

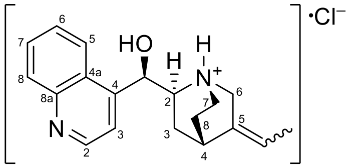

S-25

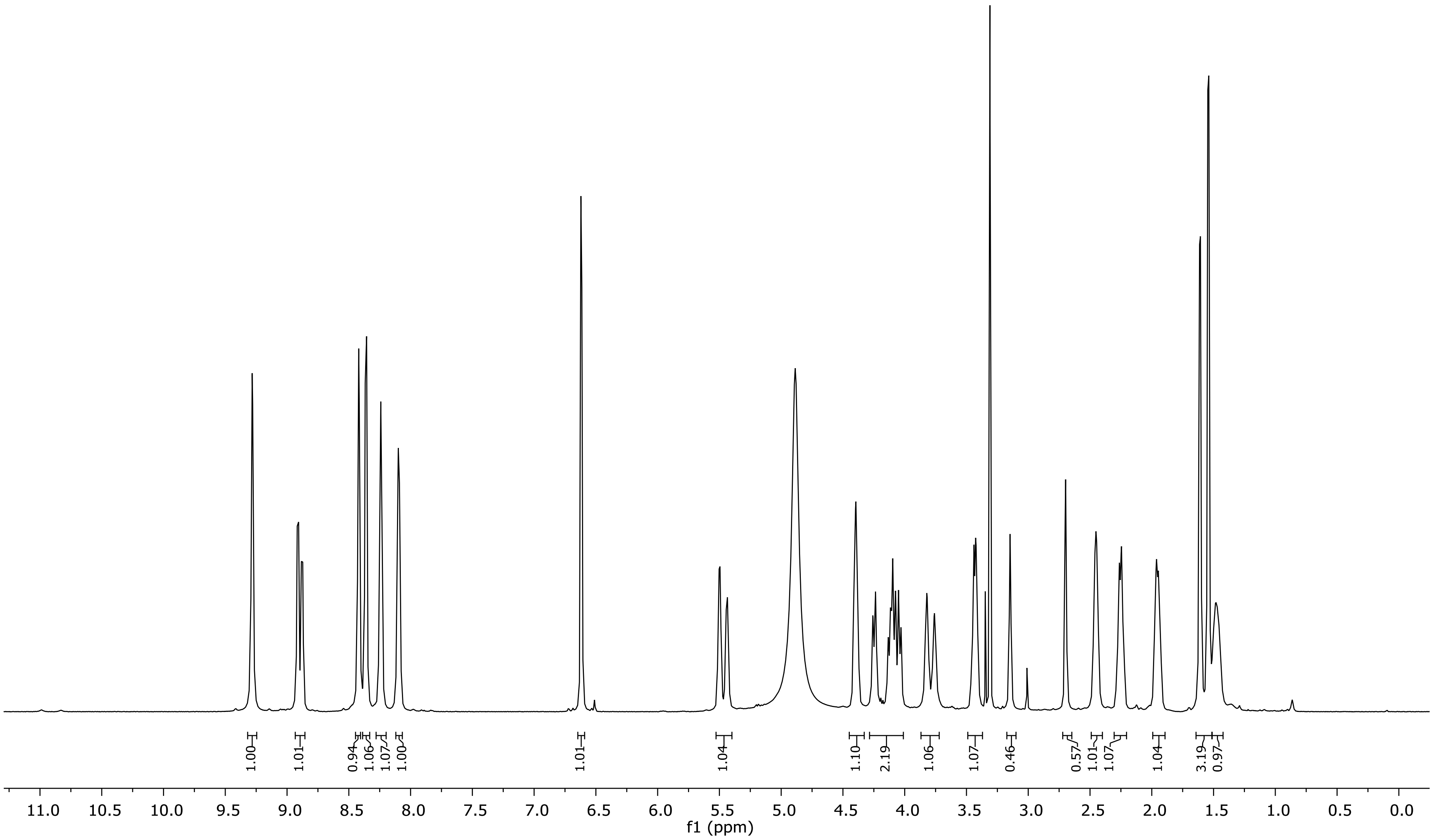

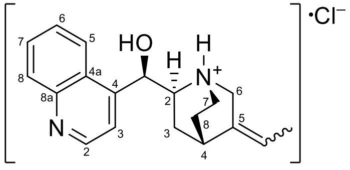

S-25

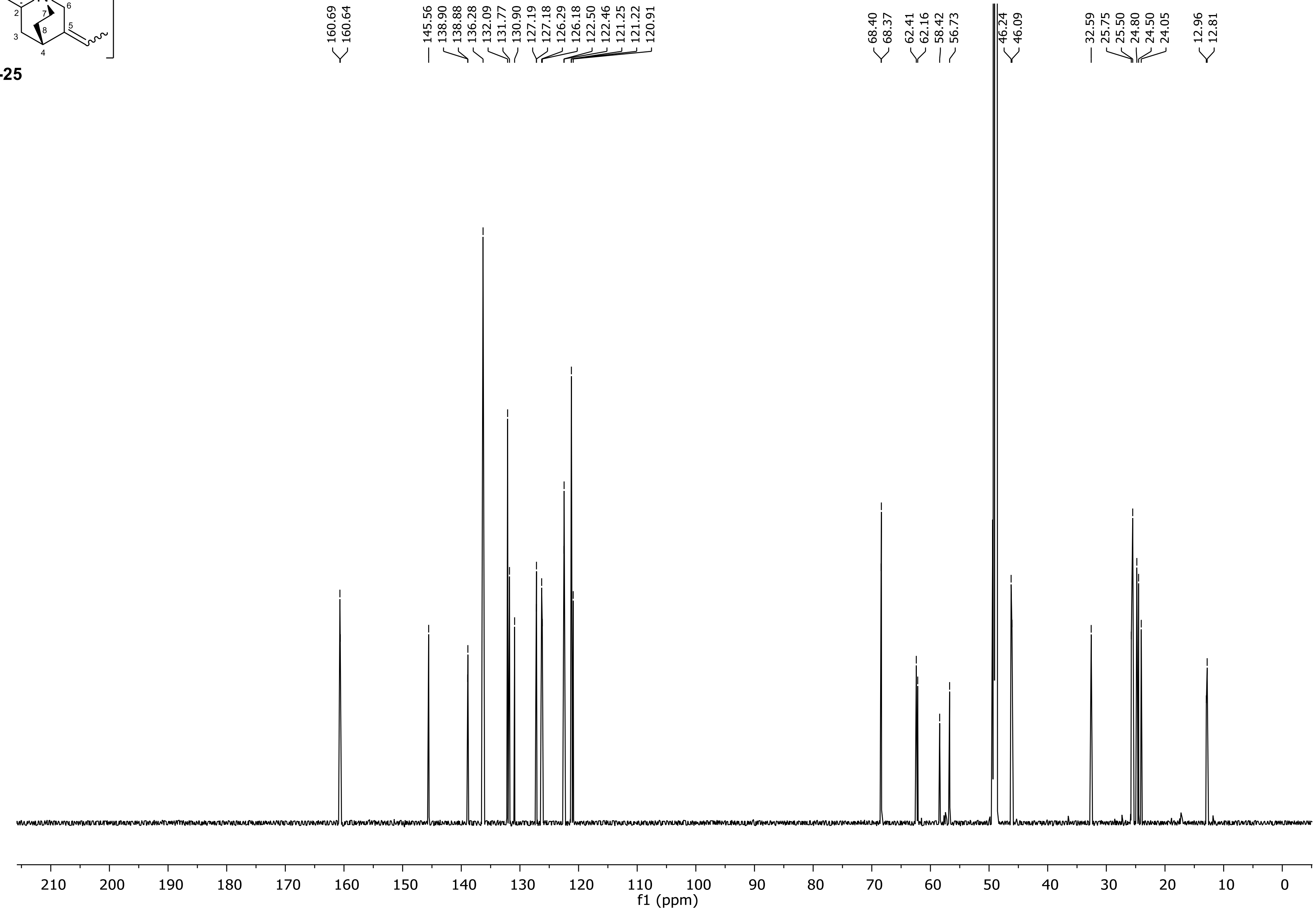
160.69
160.64
145.56
138.90
138.88
136.28
132.09
131.77
130.90
127.19
127.18
126.29
126.18
122.50
122.46
121.25
121.22
120.91
68.40
68.37
62.41
62.16
58.42
56.73
46.24
46.09
32.59
25.75
25.50
24.80
24.50
24.05
12.96
12.81
210 200 190 180 170 160 150 140 130 120 110 100 90 80 70 60 50 40 30 20 10 0
f1 (ppm)

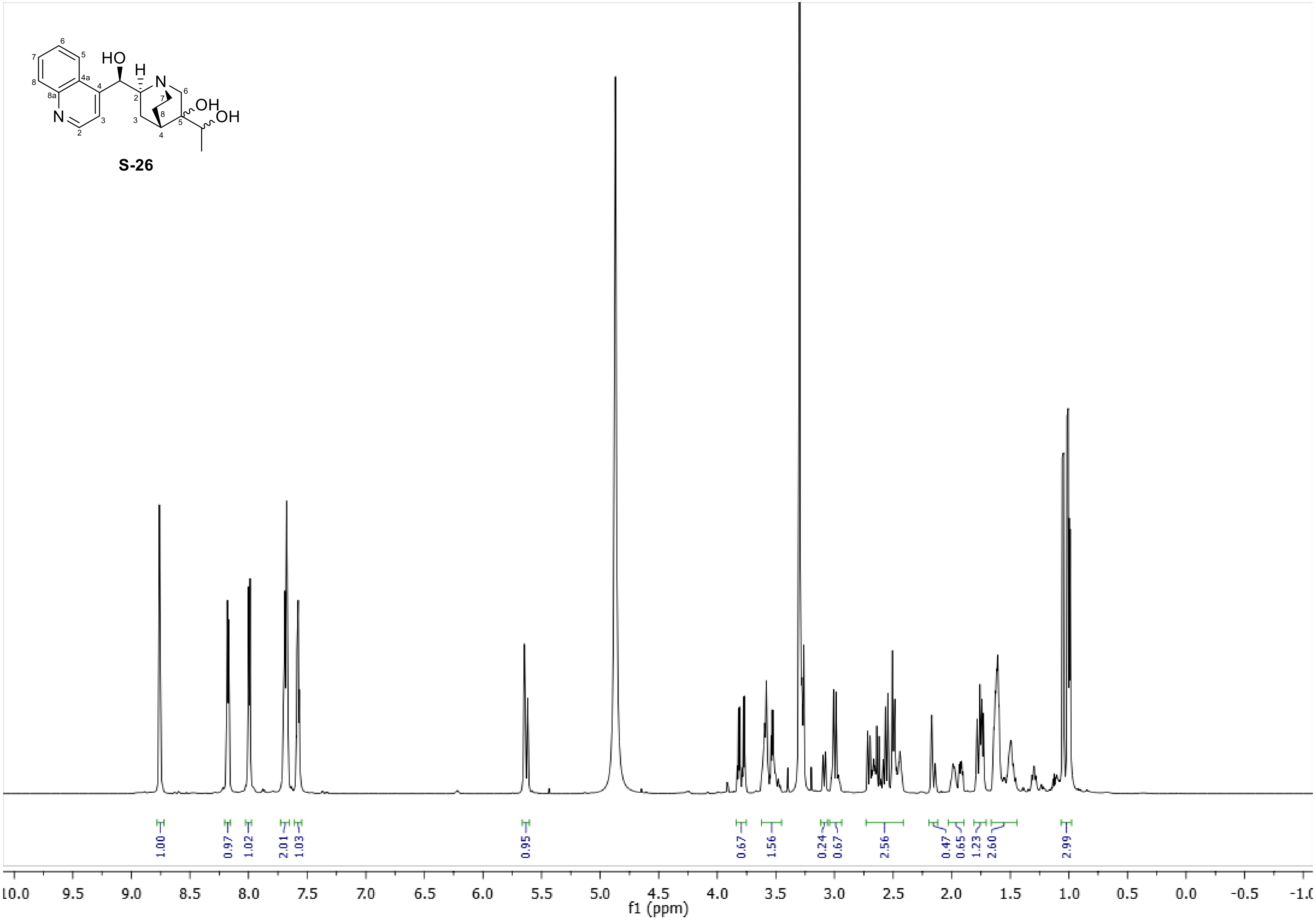
HO
H
N
OH
OH
S-26
1.00
0.97
1.02
2.01
1.03
0.95
0.67
1.56
0.24
0.67
2.56
0.47
0.65
1.23
2.60
2.99
10.0 9.5 9.0 8.5 8.0 7.5 7.0 6.5 6.0 5.5 5.0 4.5 4.0 3.5 3.0 2.5 2.0 1.5 1.0 0.5 0.0 -0.5
f1 (ppm)

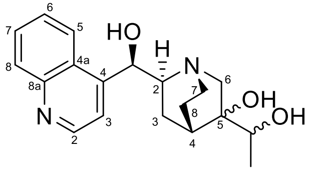
HO
H
N
OH
OH
2
3
4
4a
5
6
7
8
8a

S-26

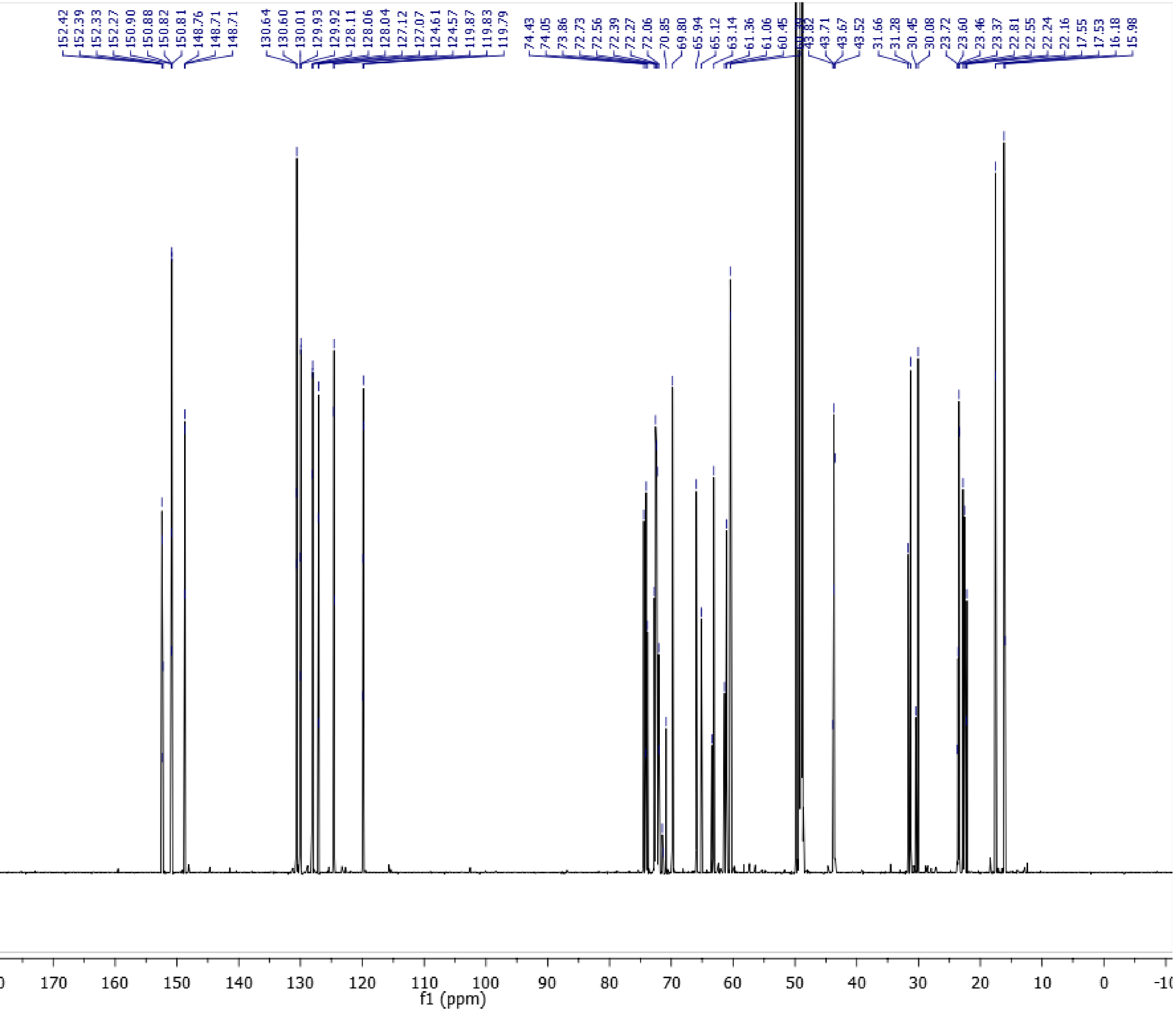
152.42
152.39
152.33
152.27
150.90
150.88
150.82
150.81
148.76
148.71
148.71
130.64
130.60
130.01
129.93
129.92
128.11
128.06
128.04
127.12
127.07
124.61
124.57
119.87
119.83
119.79
74.43
74.05
73.86
72.73
72.56
72.39
72.27
72.06
70.85
69.80
65.94
65.12
63.14
61.36
61.06
60.45
43.82
43.71
43.67
43.52
31.66
31.28
30.45
30.08
23.72
23.60
23.46
23.37
22.81
22.55
22.24
22.16
17.55
17.53
16.18
15.98
220 210 200 190 180 170 160 150 140 130 120 110 100 90 80 70 60 50 40 30 20 10 0
f1 (ppm)

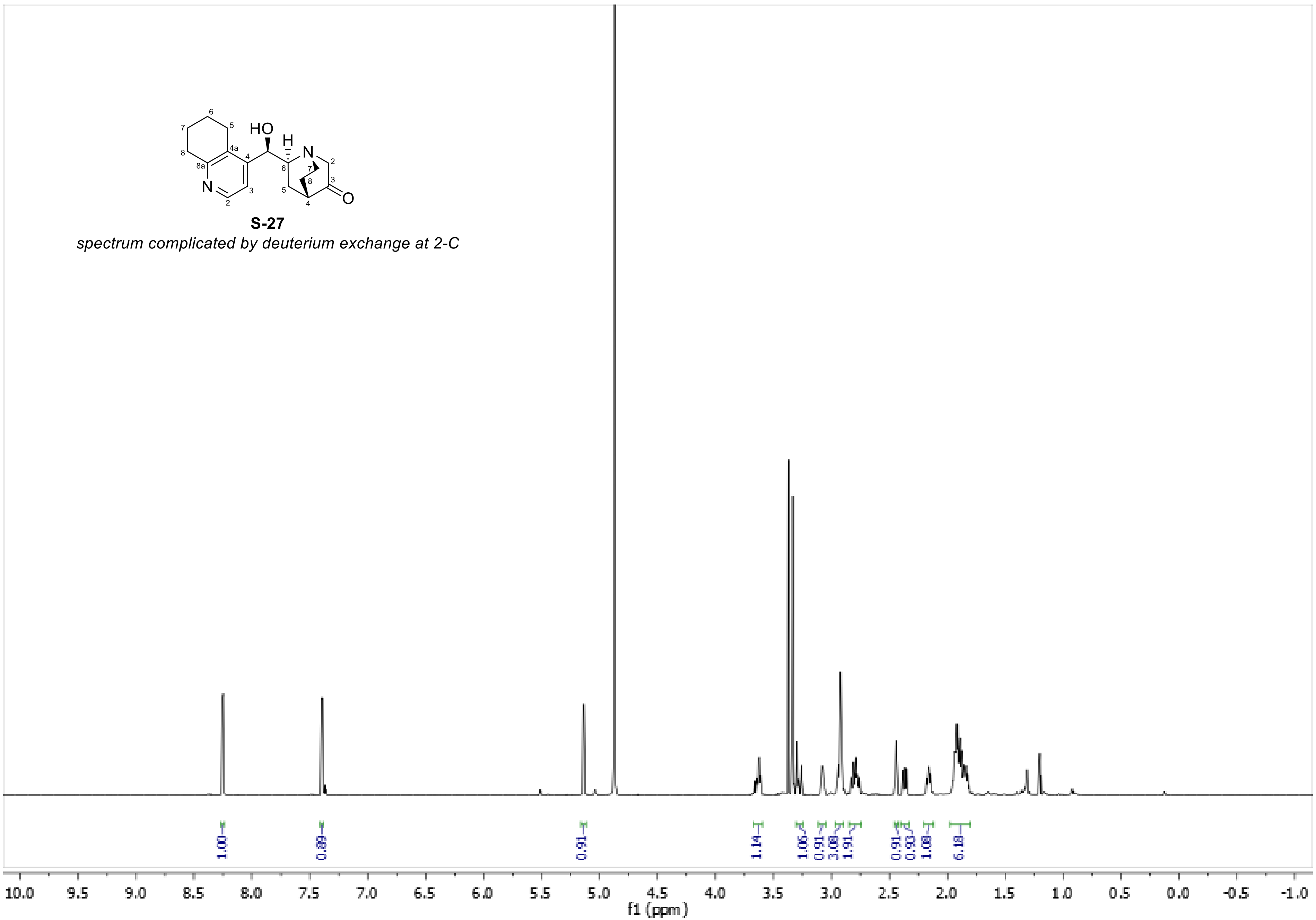
HO
H
N
S-27
spectrum complicated by deuterium exchange at 2-C
1.00
0.89
0.91
1.14
1.06
0.91
3.08
1.91
0.91
0.93
1.08
6.18
10.0 9.5 9.0 8.5 8.0 7.5 7.0 6.5 6.0 5.5 5.0 4.5 4.0 3.5 3.0 2.5 2.0 1.5 1.0 0.5 0.0 -0.5 -1.0
f1 (ppm)

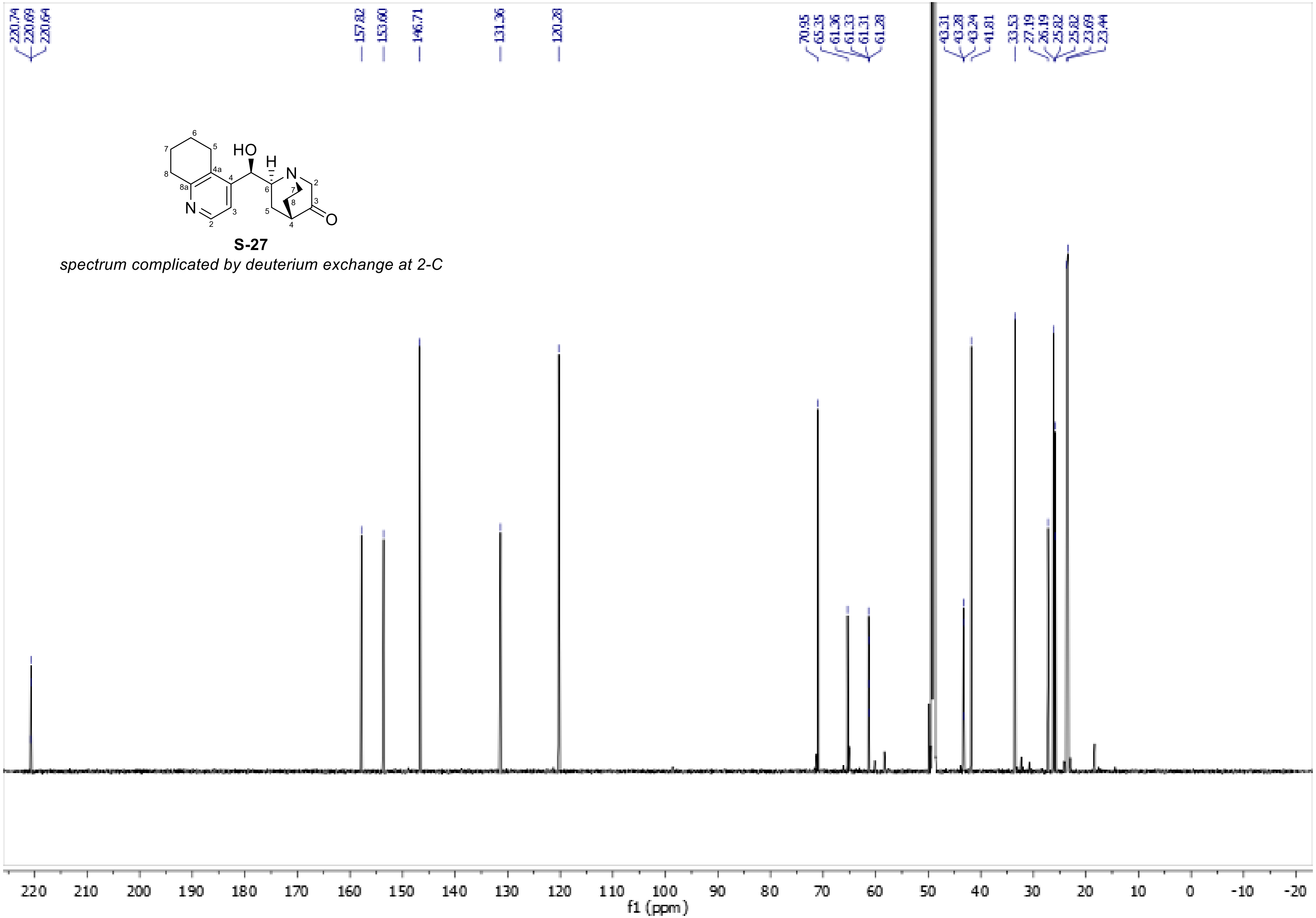
220.74
220.69
220.64
157.82
153.60
146.71
131.36
120.28
70.95
65.35
61.36
61.33
61.31
61.28
43.31
43.28
43.24
41.81
33.53
27.19
26.19
25.82
25.82
23.69
23.44
HO
H
N
S-27
spectrum complicated by deuterium exchange at 2-C
220 210 200 190 180 170 160 150 140 130 120 110 100 90 80 70 60 50 40 30 20 10 0 -10 -20
f1 (ppm)

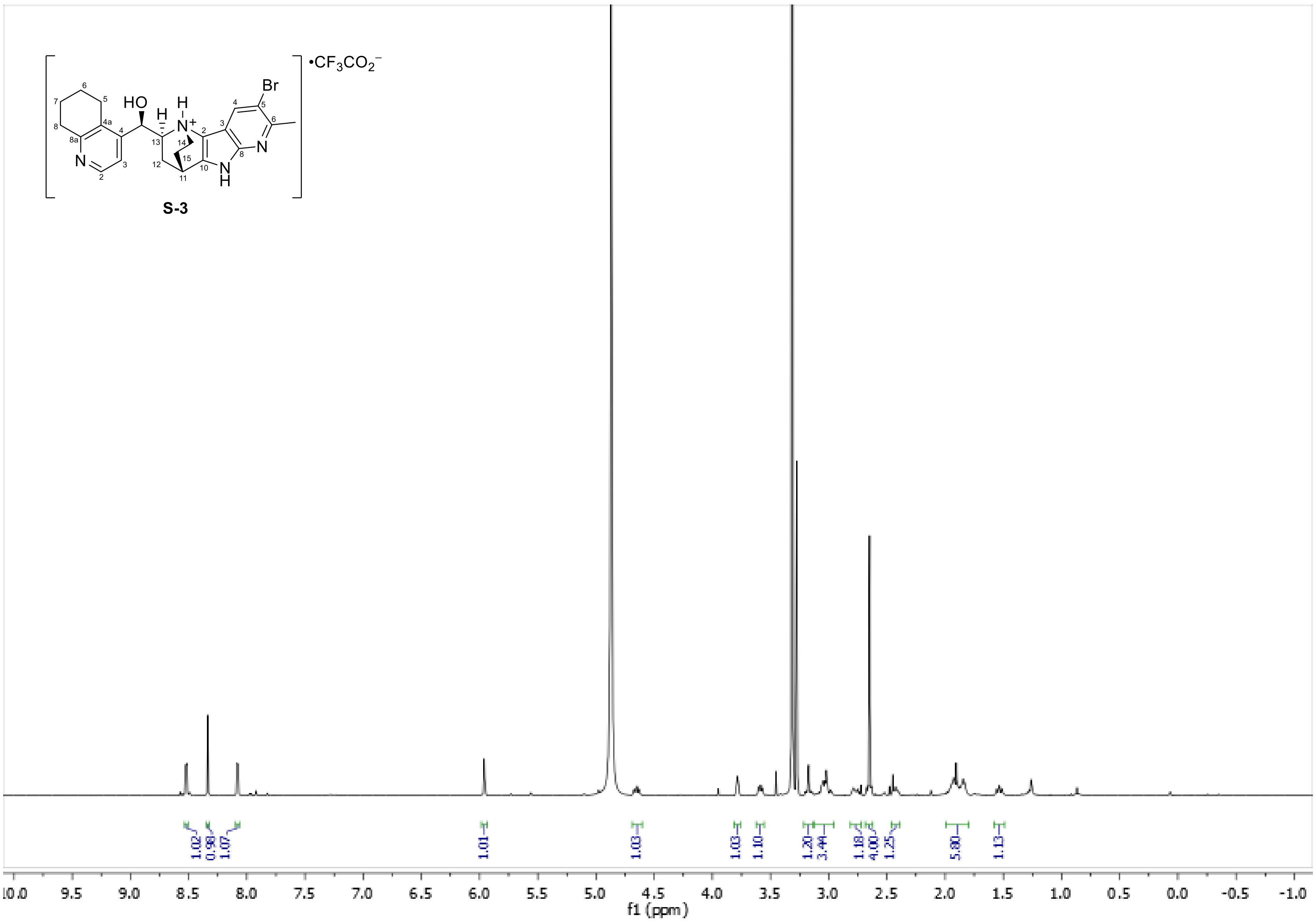
•CF3CO2−
S-3
1.02
0.98
1.07
1.01
1.03
1.03
1.10
1.20
3.44
1.18
4.00
1.25
5.80
1.13
f1 (ppm)

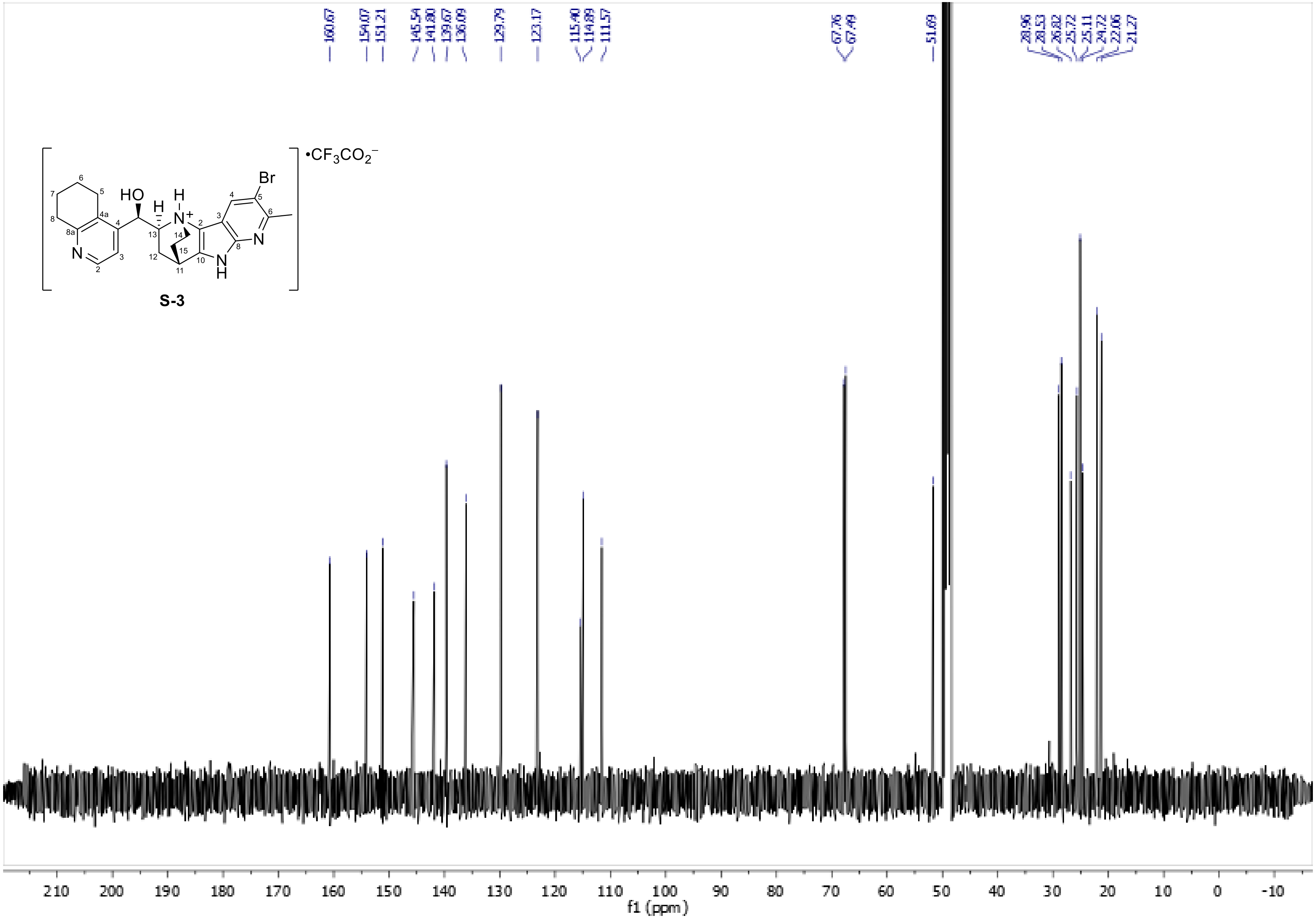

160.67
154.07
151.21
145.54
141.80
139.67
136.09
129.79
123.17
115.40
114.89
111.57
67.76
67.49
51.69
28.96
28.53
26.82
25.72
25.11
24.72
22.06
21.27
•CF3CO2−
S-3
210 200 190 180 170 160 150 140 130 120 110 100 90 80 70 60 50 40 30 20 10 0 -10
f1 (ppm)

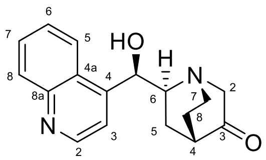

S-28

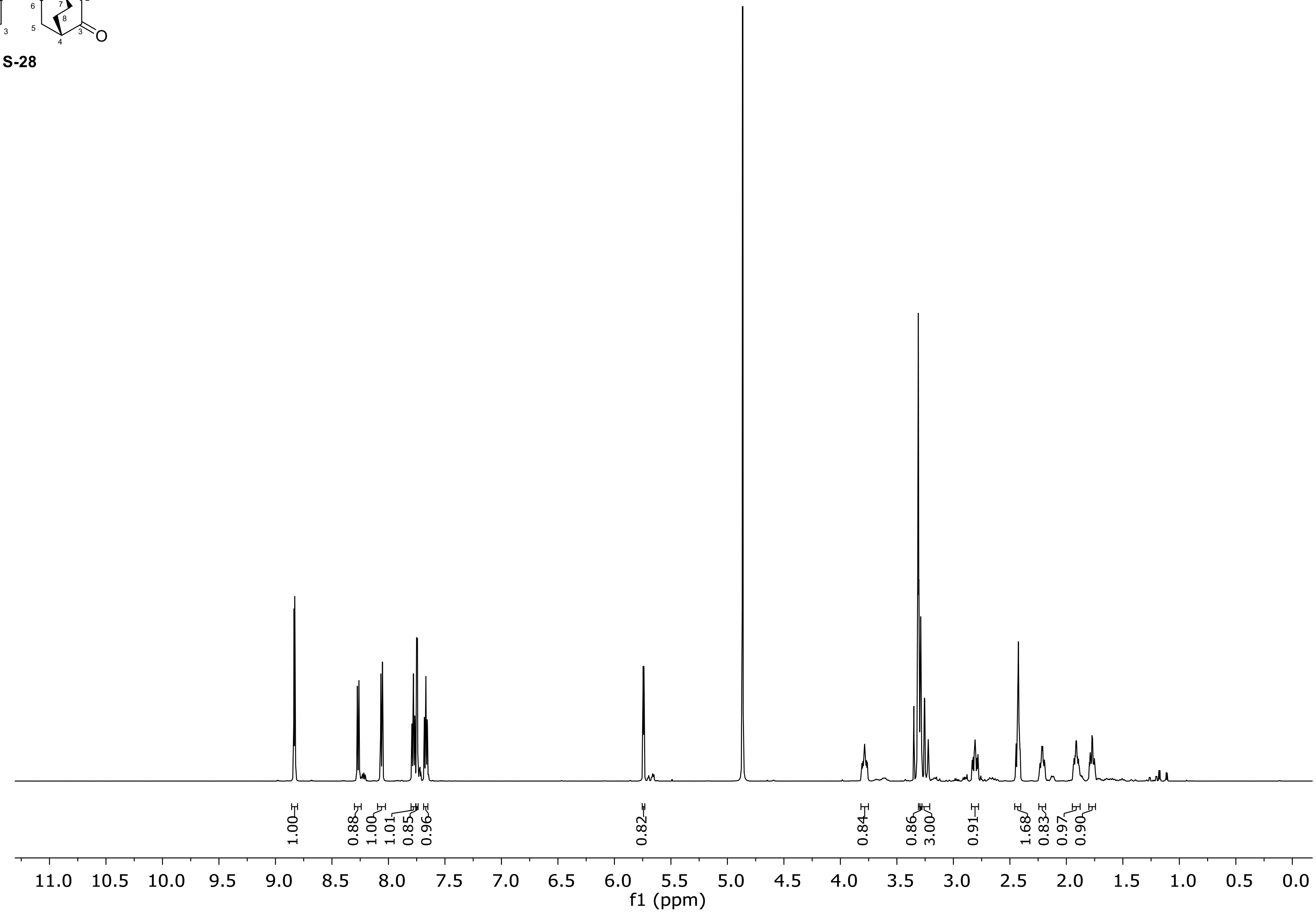
1.00
0.88
1.00
1.01
0.85
0.96
0.82
0.84
0.86
3.00
0.91
1.68
0.83
0.97
0.90
11.0 10.5 10.0 9.5 9.0 8.5 8.0 7.5 7.0 6.5 6.0 5.5 5.0 4.5 4.0 3.5 3.0 2.5 2.0 1.5 1.0 0.5 0.0
f1 (ppm)

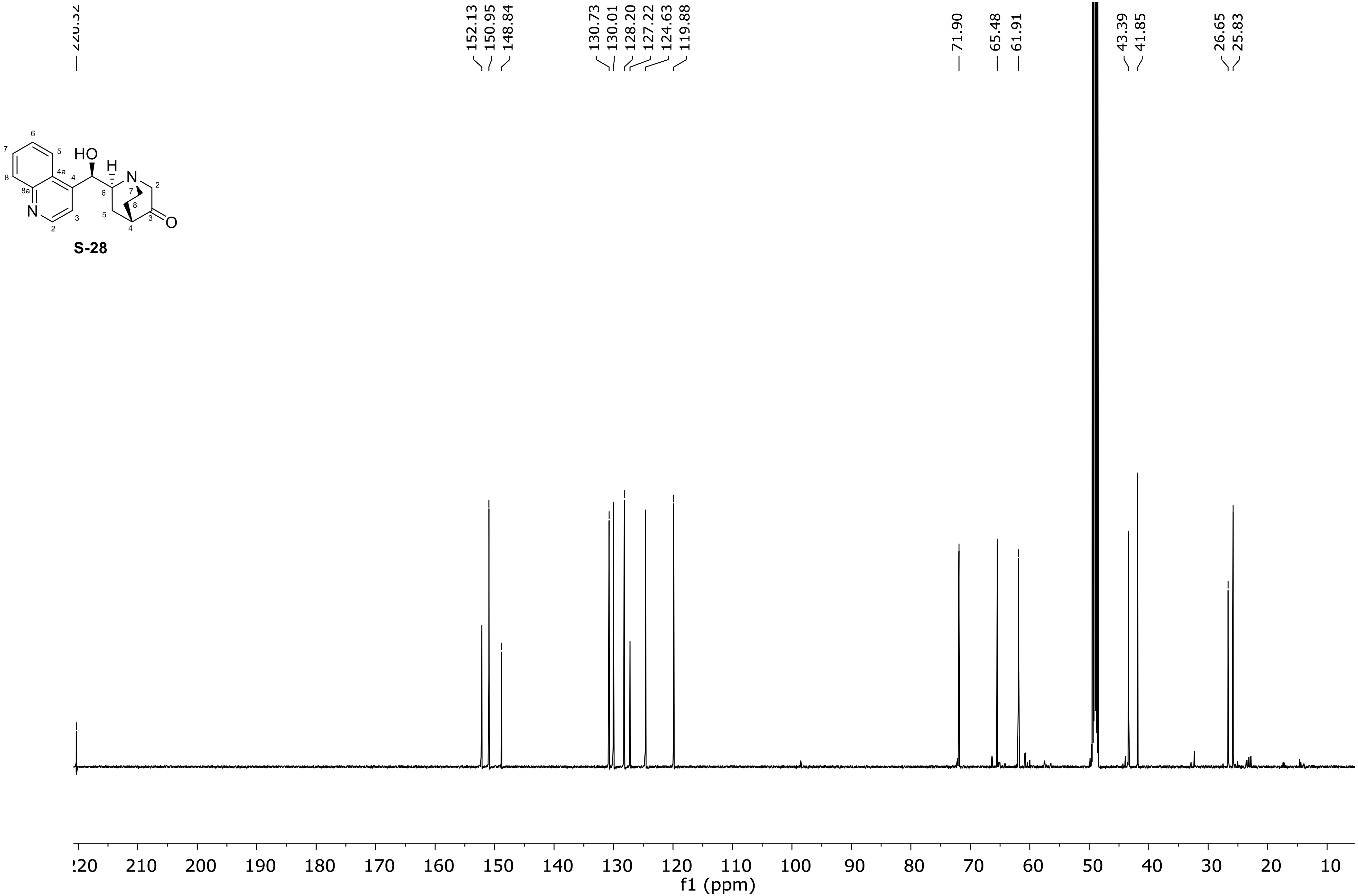
HO
H
N
O
S-28
152.13
150.95
148.84
130.73
130.01
128.20
127.22
124.63
119.88
71.90
65.48
61.91
43.39
41.85
26.65
25.83
f1 (ppm)

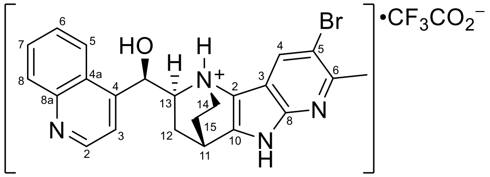
HO
H
H
N+
Br
•$CF_3CO_2^-$
N
N
NH

S-4

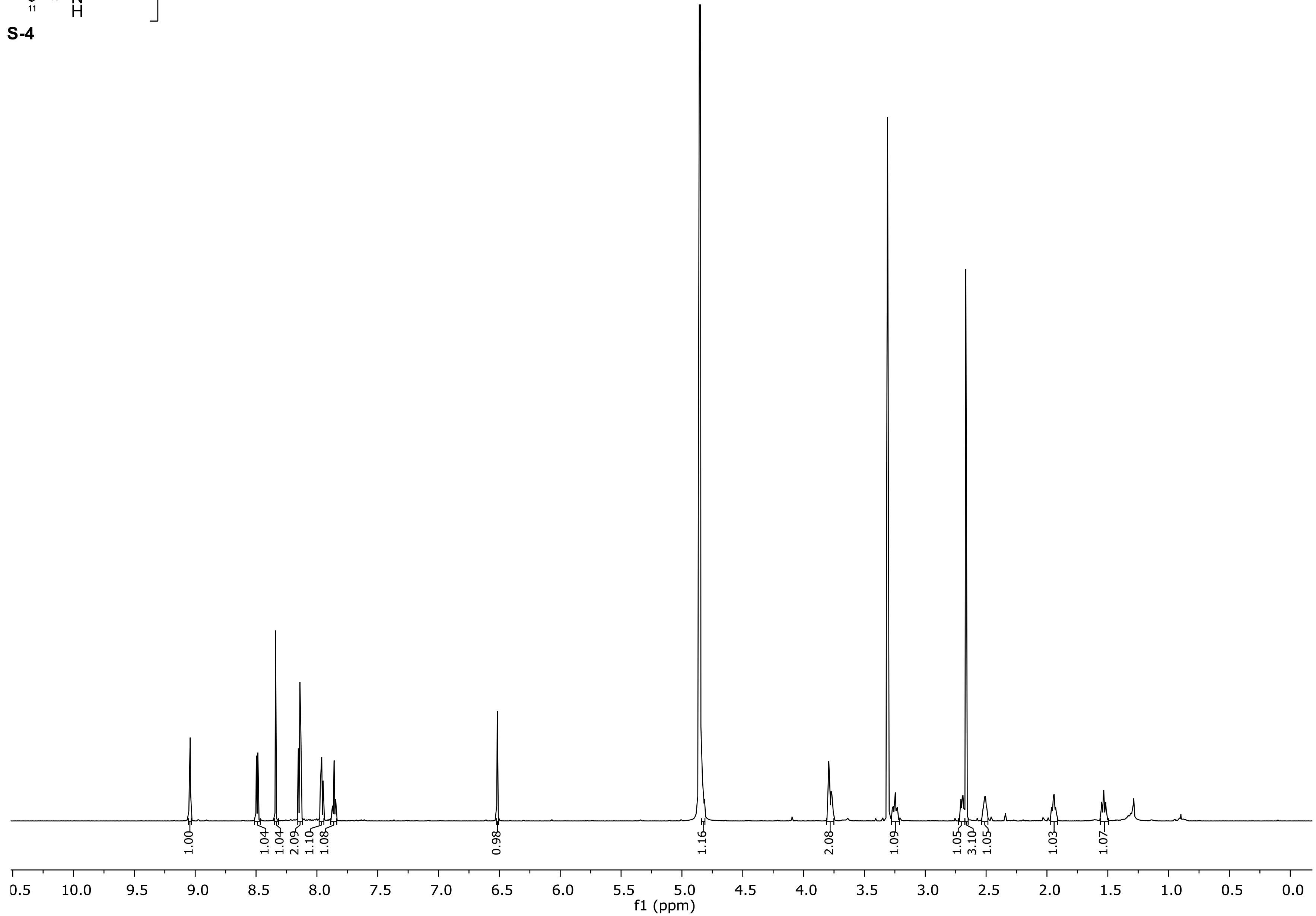
1.00
1.04
1.04
2.09
1.10
1.08
0.98
1.16
2.08
1.09
1.05
3.10
1.05
1.03
1.07
0.5
10.0
9.5
9.0
8.5
8.0
7.5
7.0
6.5
6.0
5.5
5.0
4.5
4.0
3.5
3.0
2.5
2.0
1.5
1.0
0.5
0.0
f1 (ppm)

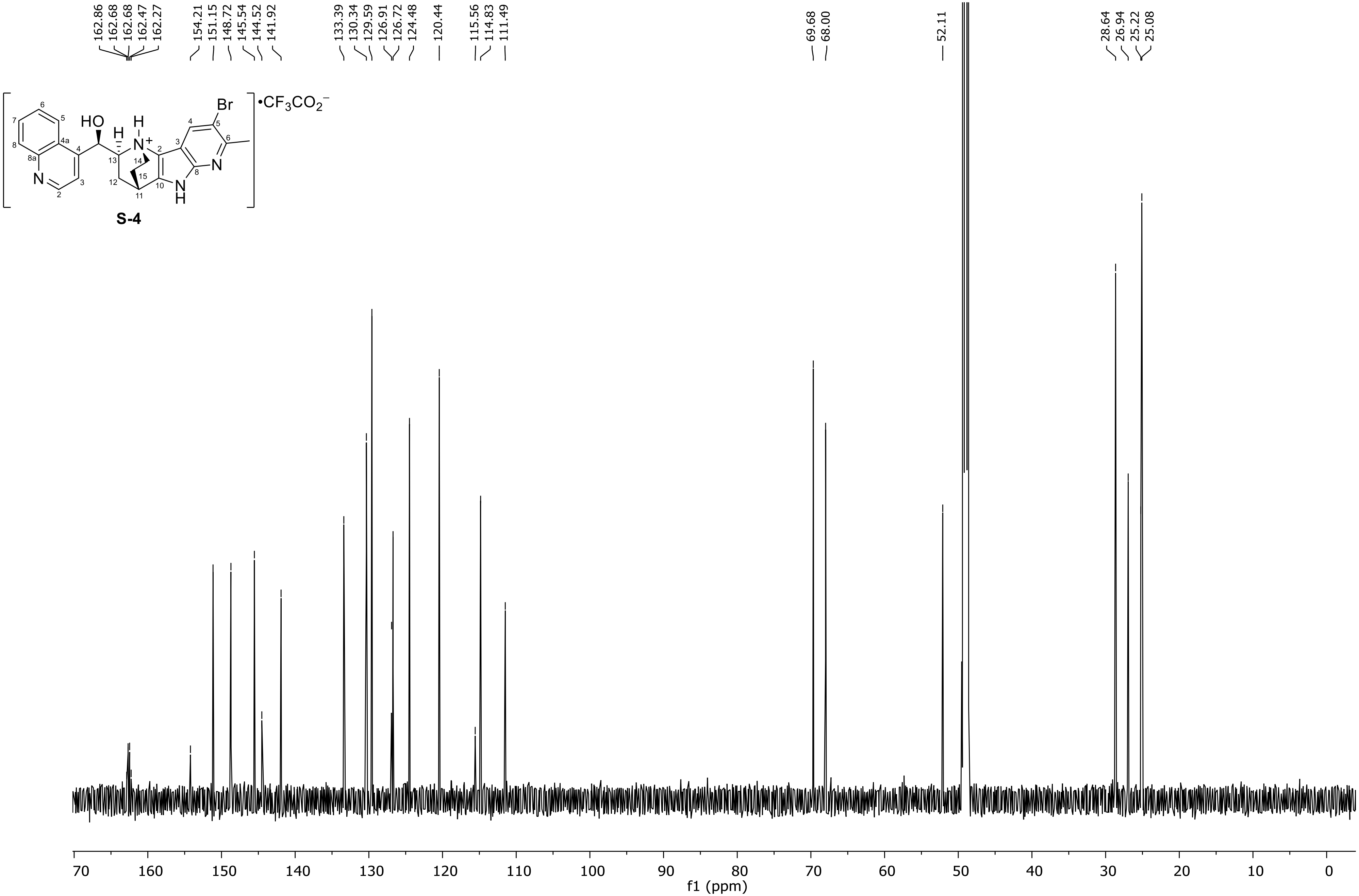
162.86
162.68
162.68
162.47
162.27
154.21
151.15
148.72
145.54
144.52
141.92
133.39
130.34
129.59
126.91
126.72
124.48
120.44
115.56
114.83
111.49
69.68
68.00
52.11
28.64
26.94
25.22
25.08
•$CF_3CO_2^-$
HO
Br
N
NH
S-4
170 160 150 140 130 120 110 100 90 80 70 60 50 40 30 20 10 0
f1 (ppm)

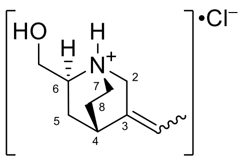
HO
H
H
N+
•Cl−
2
3
4
5
6
7
8

S-29

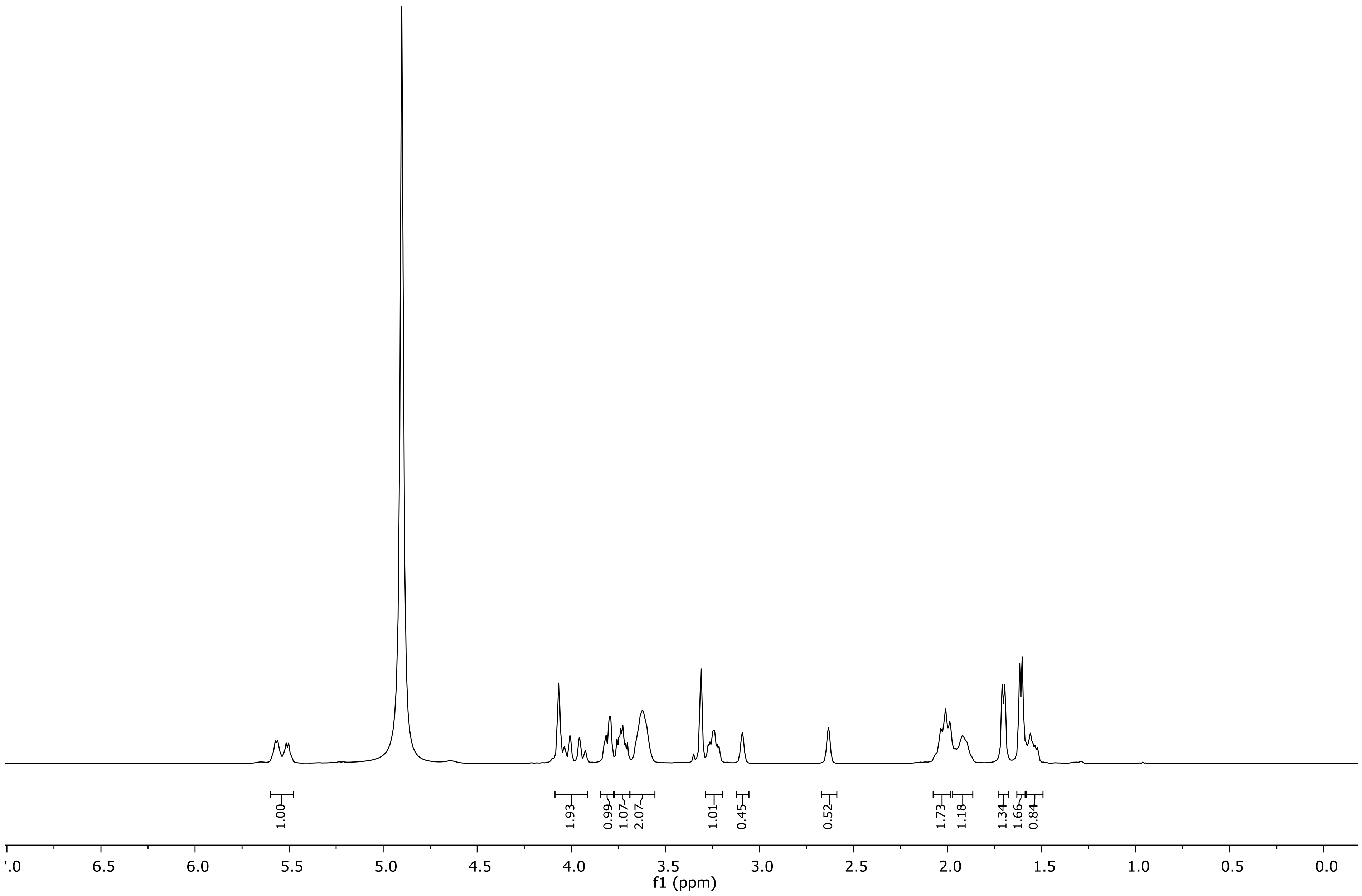
1.00
1.93
0.99
1.07
2.07
1.01
0.45
0.52
1.73
1.18
1.34
1.66
0.84
6.5
6.0
5.5
5.0
4.5
4.0
3.5
3.0
2.5
2.0
1.5
1.0
0.5
0.0
f1 (ppm)

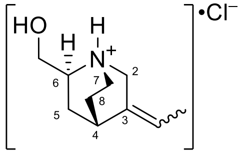
HO
H
H
N+
•Cl−
2
3
4
5
6
7
8

S-29

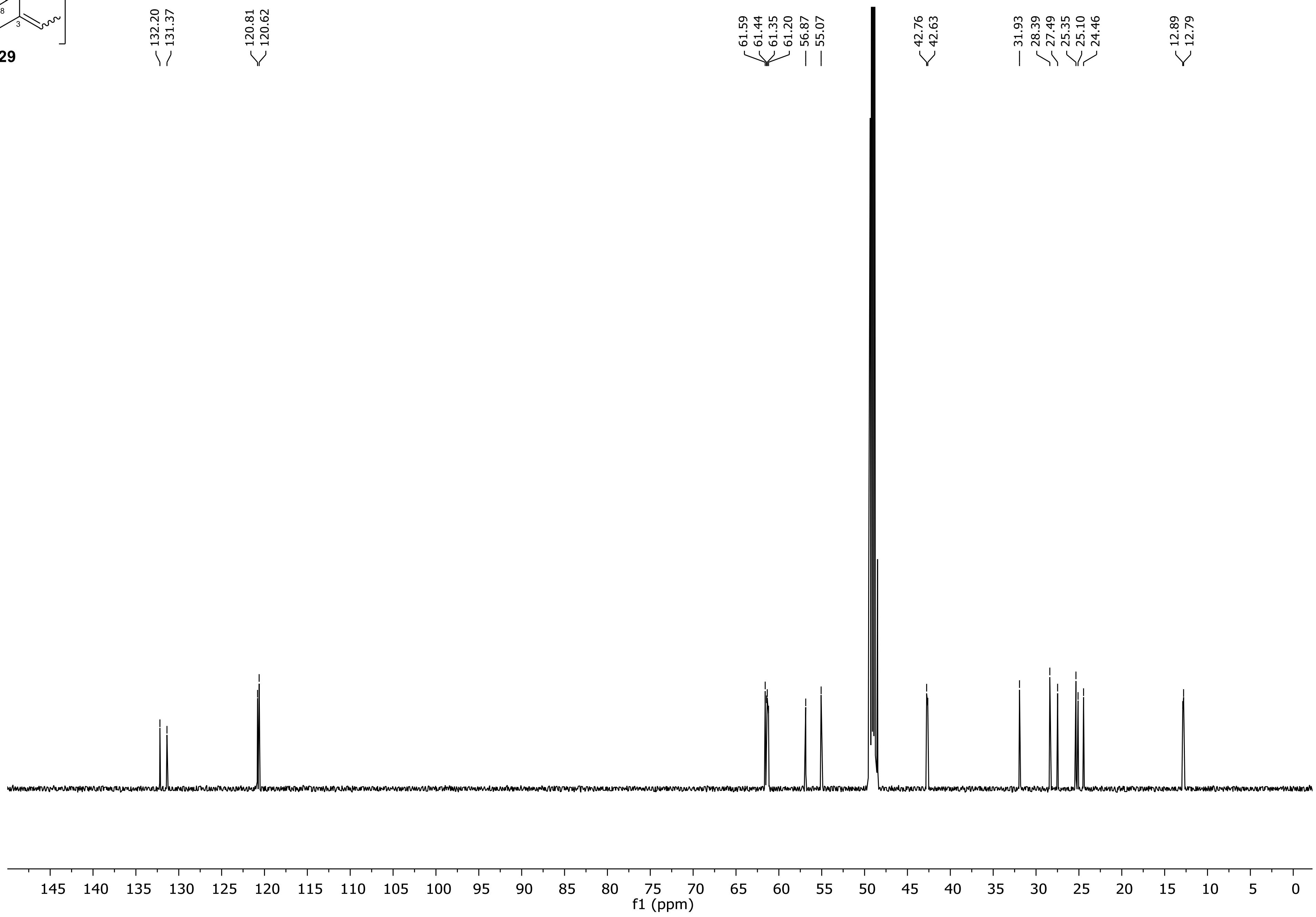
132.20
131.37
120.81
120.62
61.59
61.44
61.35
61.20
56.87
55.07
42.76
42.63
31.93
28.39
27.49
25.35
25.10
24.46
12.89
12.79
145 140 135 130 125 120 115 110 105 100 95 90 85 80 75 70 65 60 55 50 45 40 35 30 25 20 15 10 5 0
f1 (ppm)

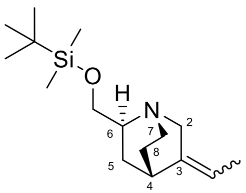
Si
O
H
N
2
6
7
8
5
3
4

S-30

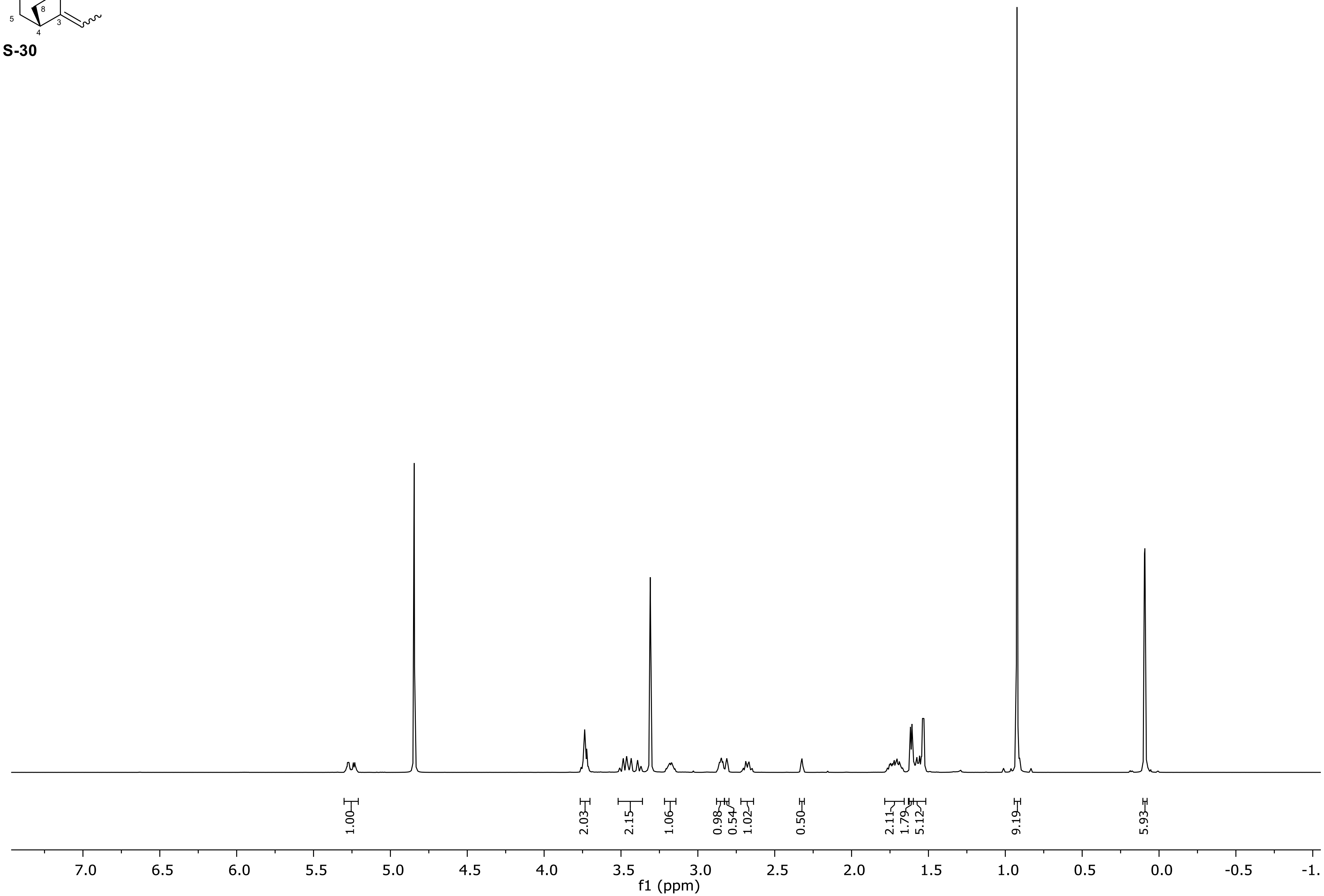
1.00
2.03
2.15
1.06
0.98
0.54
1.02
0.50
2.11
1.79
5.12
9.19
5.93
7.0
6.5
6.0
5.5
5.0
4.5
4.0
3.5
3.0
2.5
2.0
1.5
1.0
0.5
0.0
-0.5
-1.
f1 (ppm)

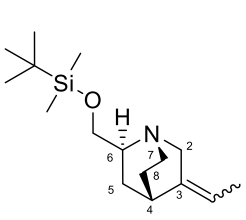

S-30

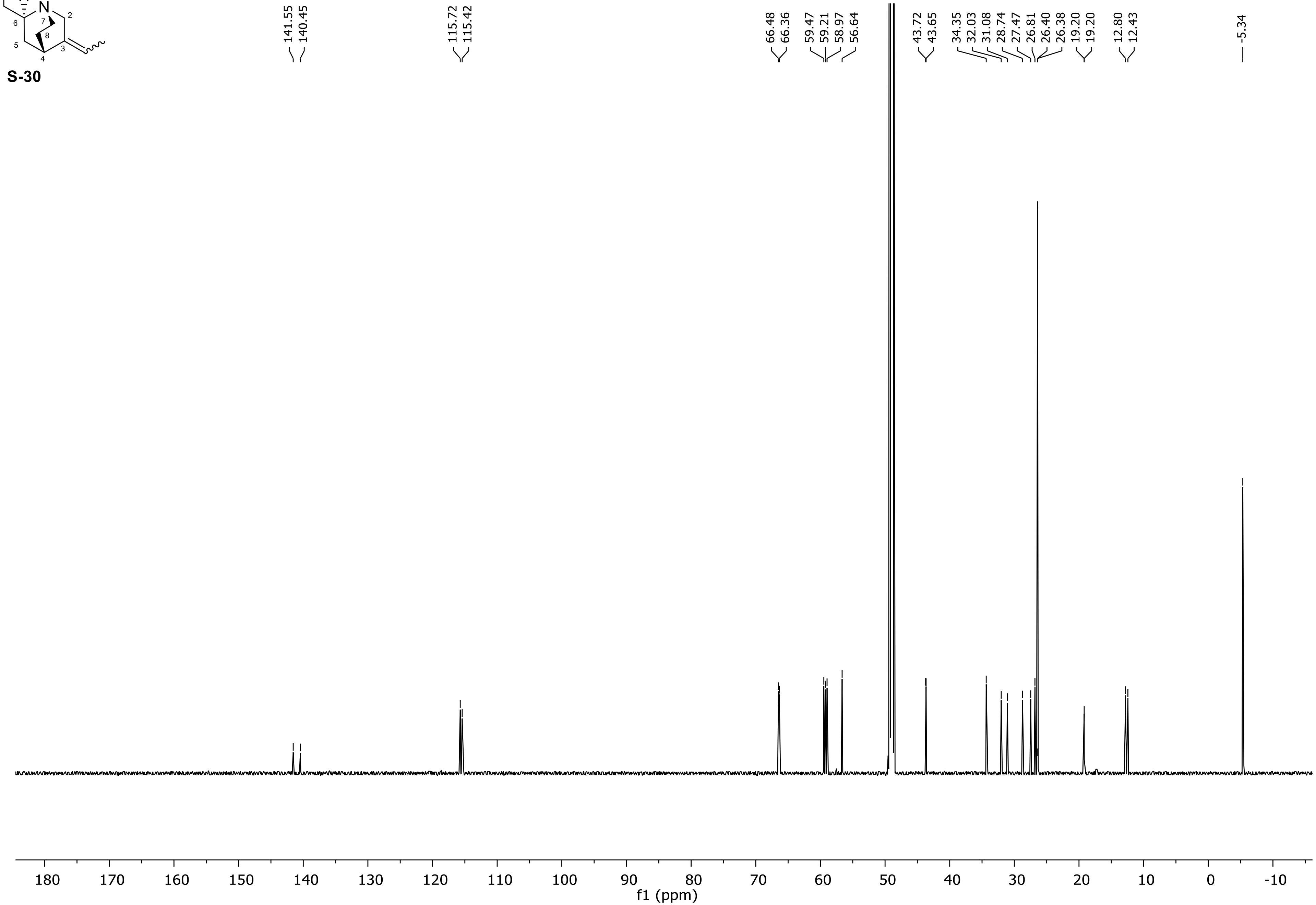
141.55
140.45
115.72
115.42
66.48
66.36
59.47
59.21
58.97
56.64
43.72
43.65
34.35
32.03
31.08
28.74
27.47
26.81
26.40
26.38
19.20
19.20
12.80
12.43
-5.34
180
170
160
150
140
130
120
110
100
90
80
70
60
50
40
30
20
10
0
-10
f1 (ppm)

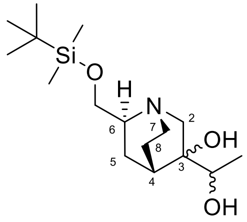
Si
O
H
N
OH
OH
2
3
4
5
6
7
8

S-31

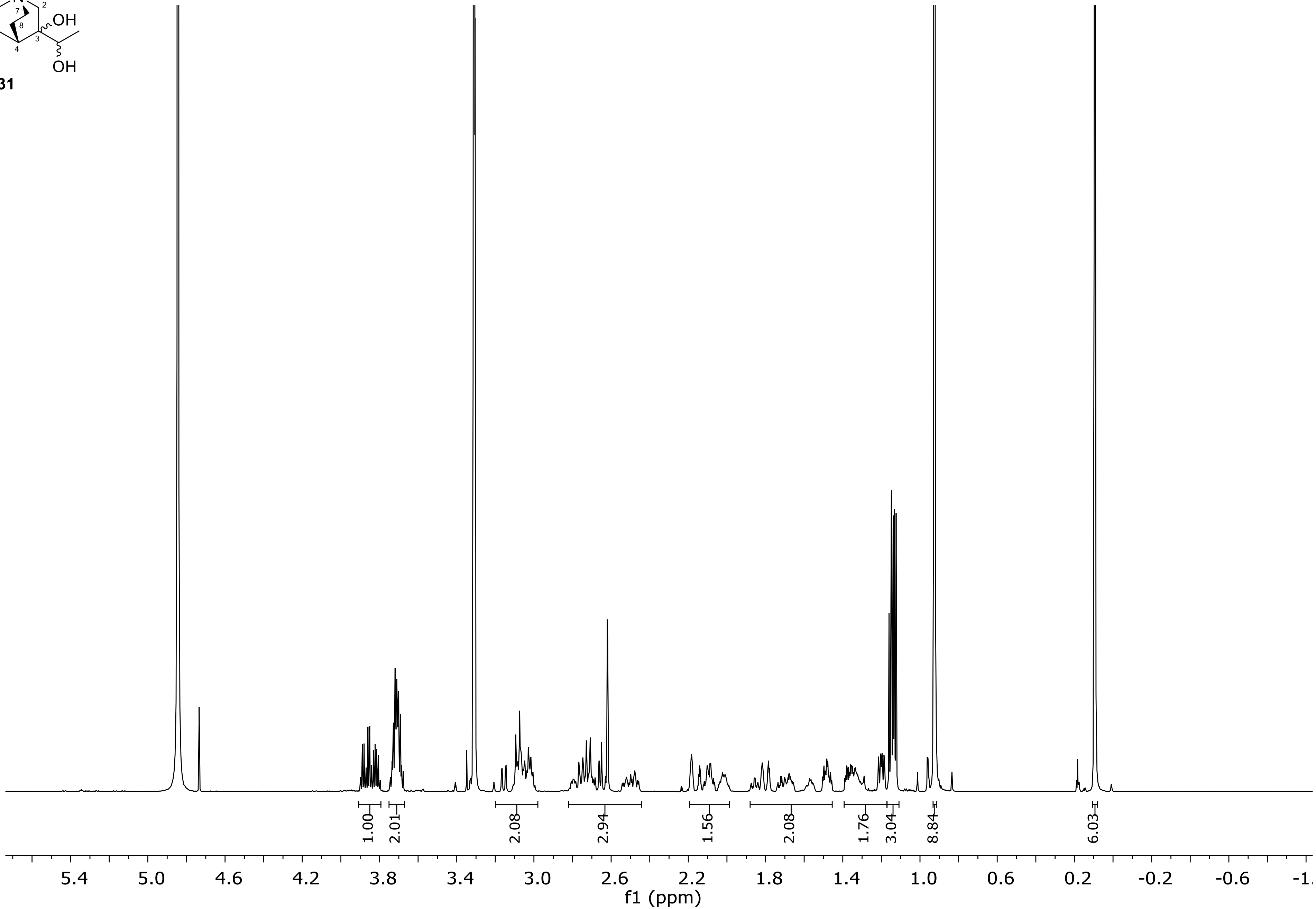
1.00
2.01
2.08
2.94
1.56
2.08
1.76
3.04
8.84
6.03
5.4
5.0
4.6
4.2
3.8
3.4
3.0
2.6
2.2
1.8
1.4
1.0
0.6
0.2
-0.2
-0.6
f1 (ppm)

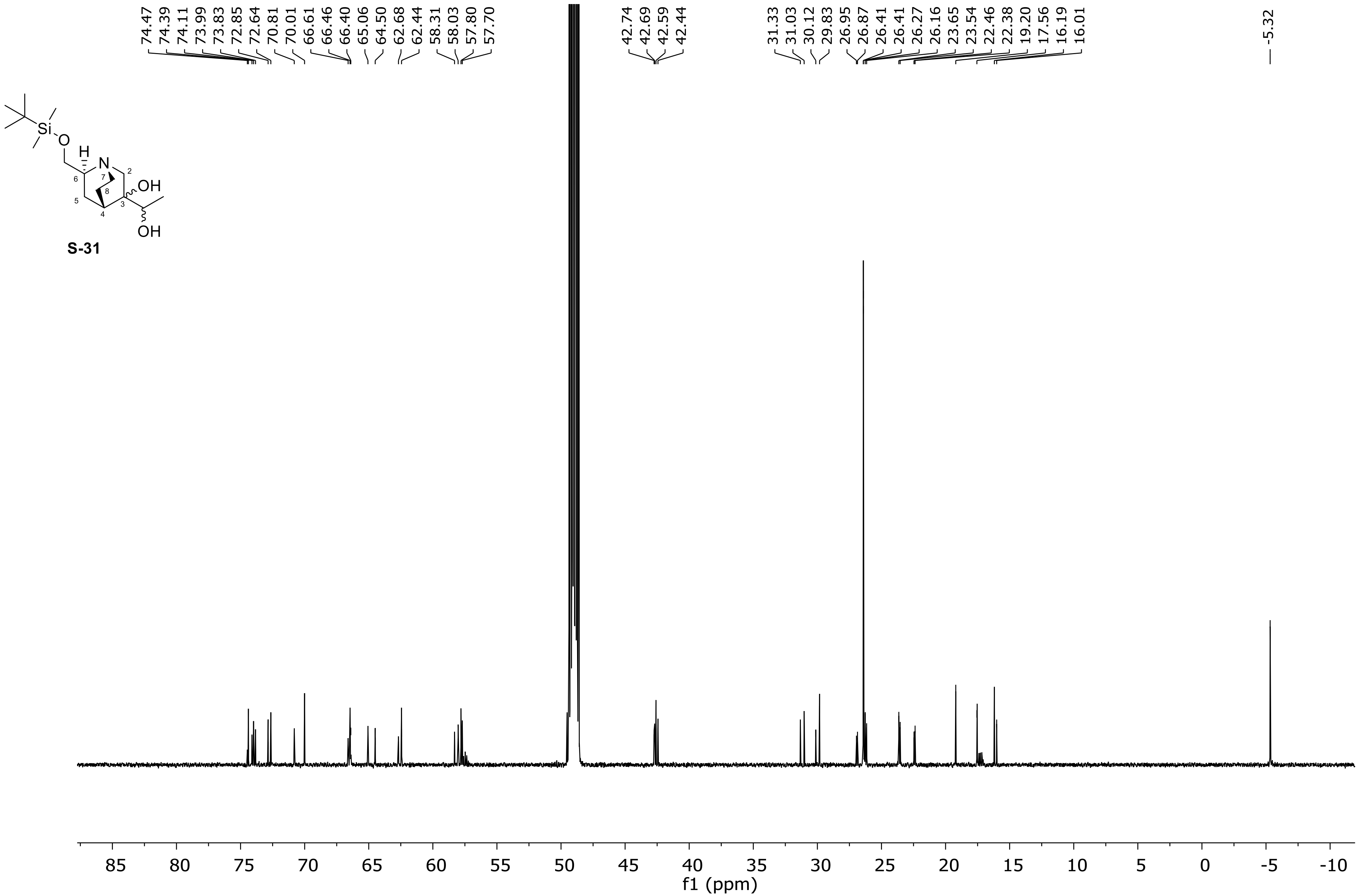
Si
O
H
N
OH
OH
2
3
4
5
6
7
8
S-31
74.47
74.39
74.11
73.99
73.83
72.85
72.64
70.81
70.01
66.61
66.46
66.40
65.06
64.50
62.68
62.44
58.31
58.03
57.80
57.70
42.74
42.69
42.59
42.44
31.33
31.03
30.12
29.83
26.95
26.87
26.41
26.41
26.27
26.16
23.65
23.54
22.46
22.38
19.20
17.56
16.19
16.01
-5.32
85
80
75
70
65
60
55
50
45
40
35
30
25
20
15
10
5
0
-5
-10
f1 (ppm)

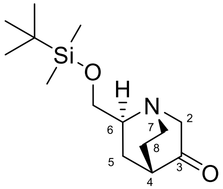
Si
O
H
N
2
6
7
8
5
3
4
O

S-32

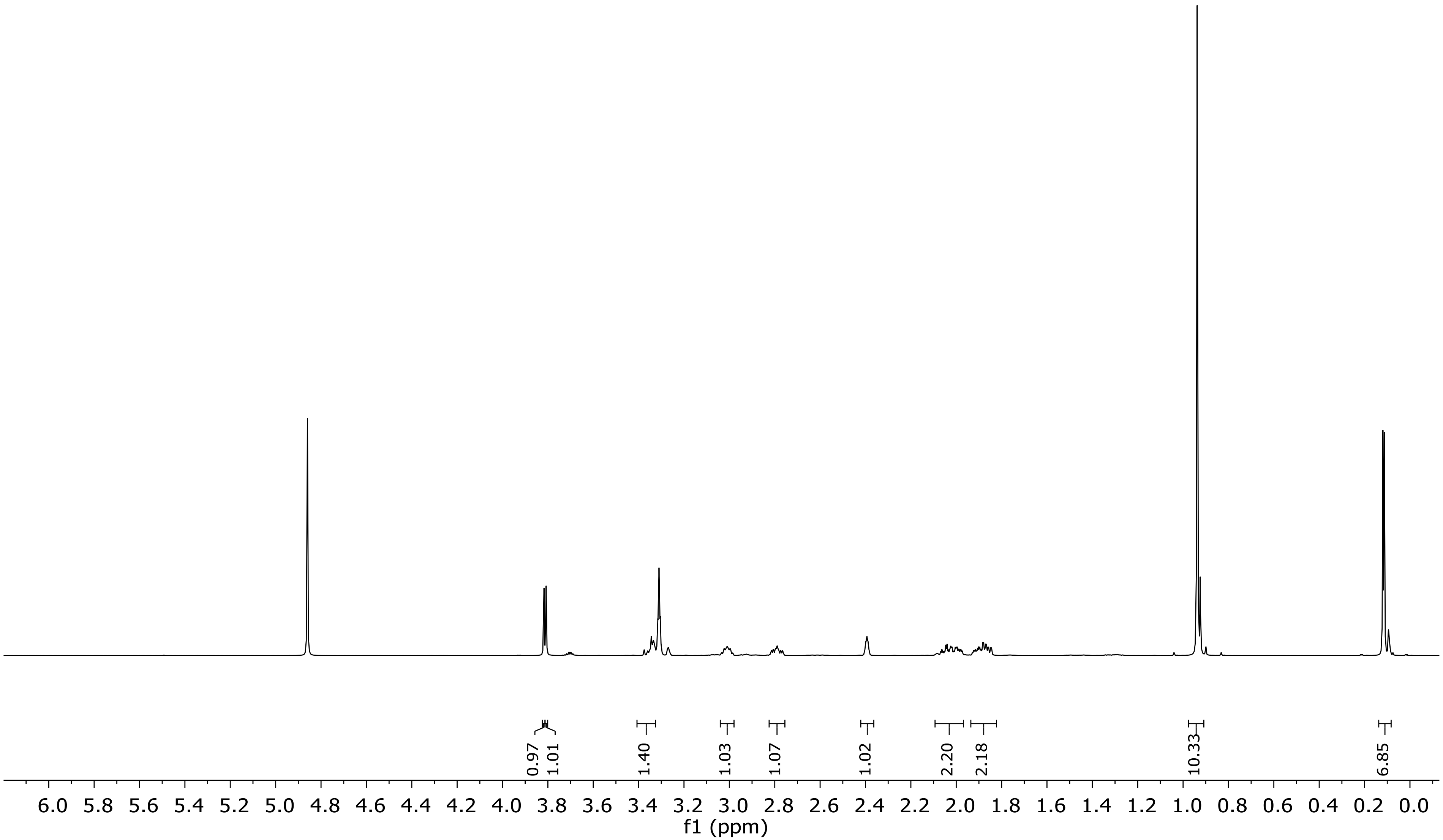
0.97
1.01
1.40
1.03
1.07
1.02
2.20
2.18
10.33
6.85
6.0 5.8 5.6 5.4 5.2 5.0 4.8 4.6 4.4 4.2 4.0 3.8 3.6 3.4 3.2 3.0 2.8 2.6 2.4 2.2 2.0 1.8 1.6 1.4 1.2 1.0 0.8 0.6 0.4 0.2 0.0
f1 (ppm)

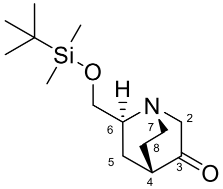
Si
O
H
N
2
3
4
5
6
7
8
O

S-32

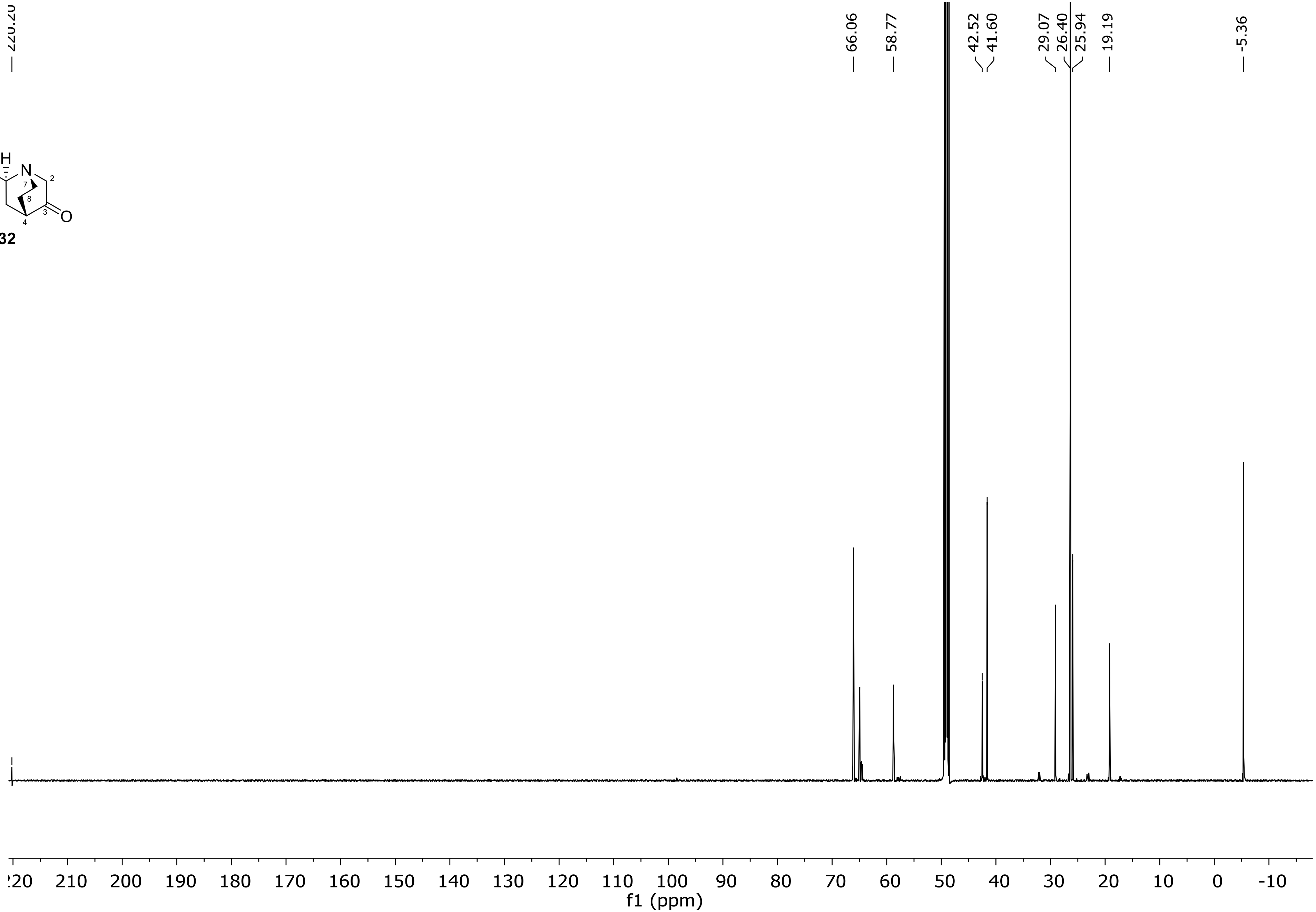
220.20
66.06
58.77
42.52
41.60
29.07
26.40
25.94
19.19
-5.36
220
210
200
190
180
170
160
150
140
130
120
110
100
90
80
70
60
50
40
30
20
10
0
-10
f1 (ppm)

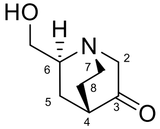
HO
H
N
2
6
7
8
5
4
3
O

S-33

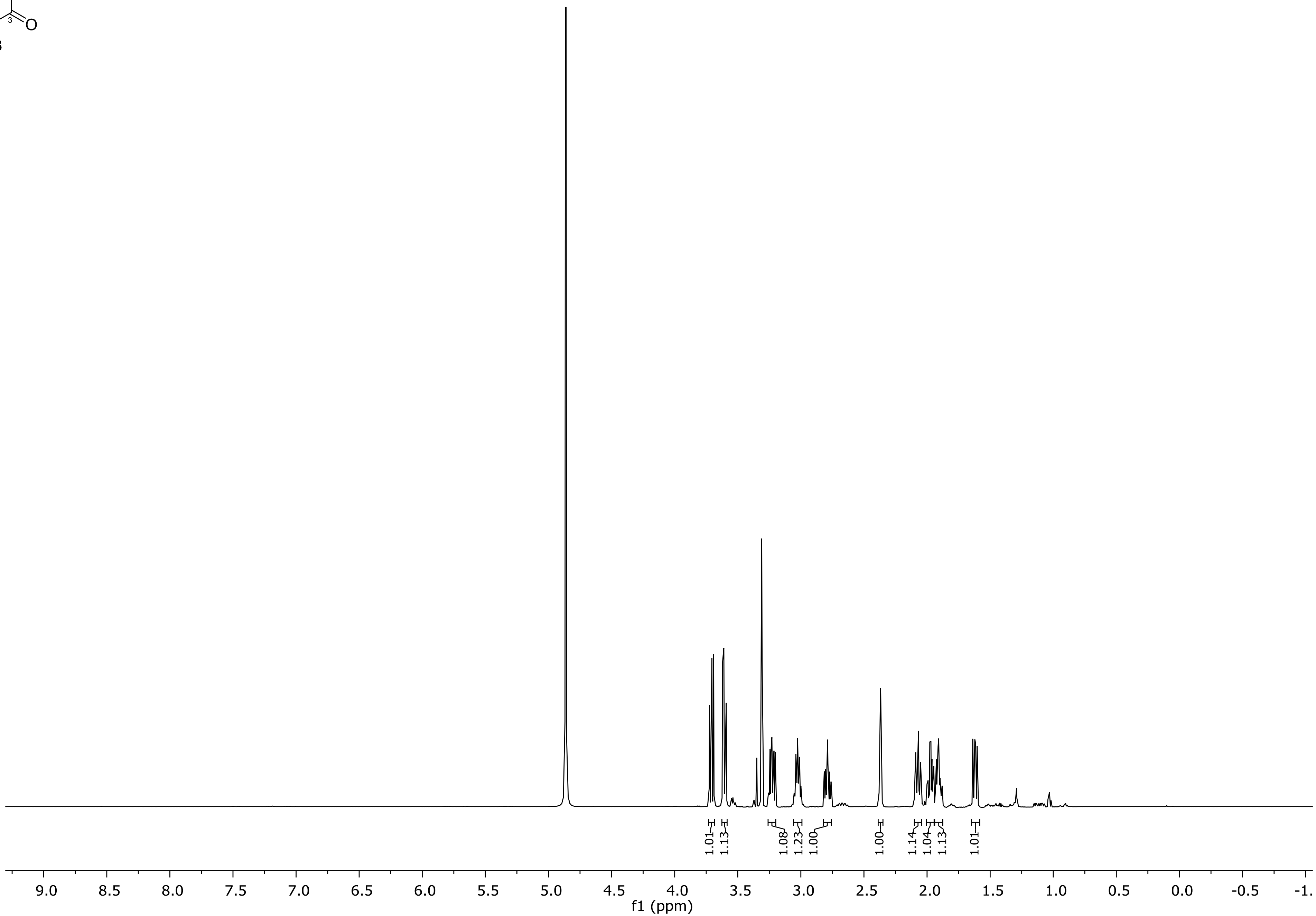
1.01
1.13
1.08
1.23
1.00
1.00
1.14
1.04
1.13
1.01
9.0 8.5 8.0 7.5 7.0 6.5 6.0 5.5 5.0 4.5 4.0 3.5 3.0 2.5 2.0 1.5 1.0 0.5 0.0 -0.5 -1.
f1 (ppm)

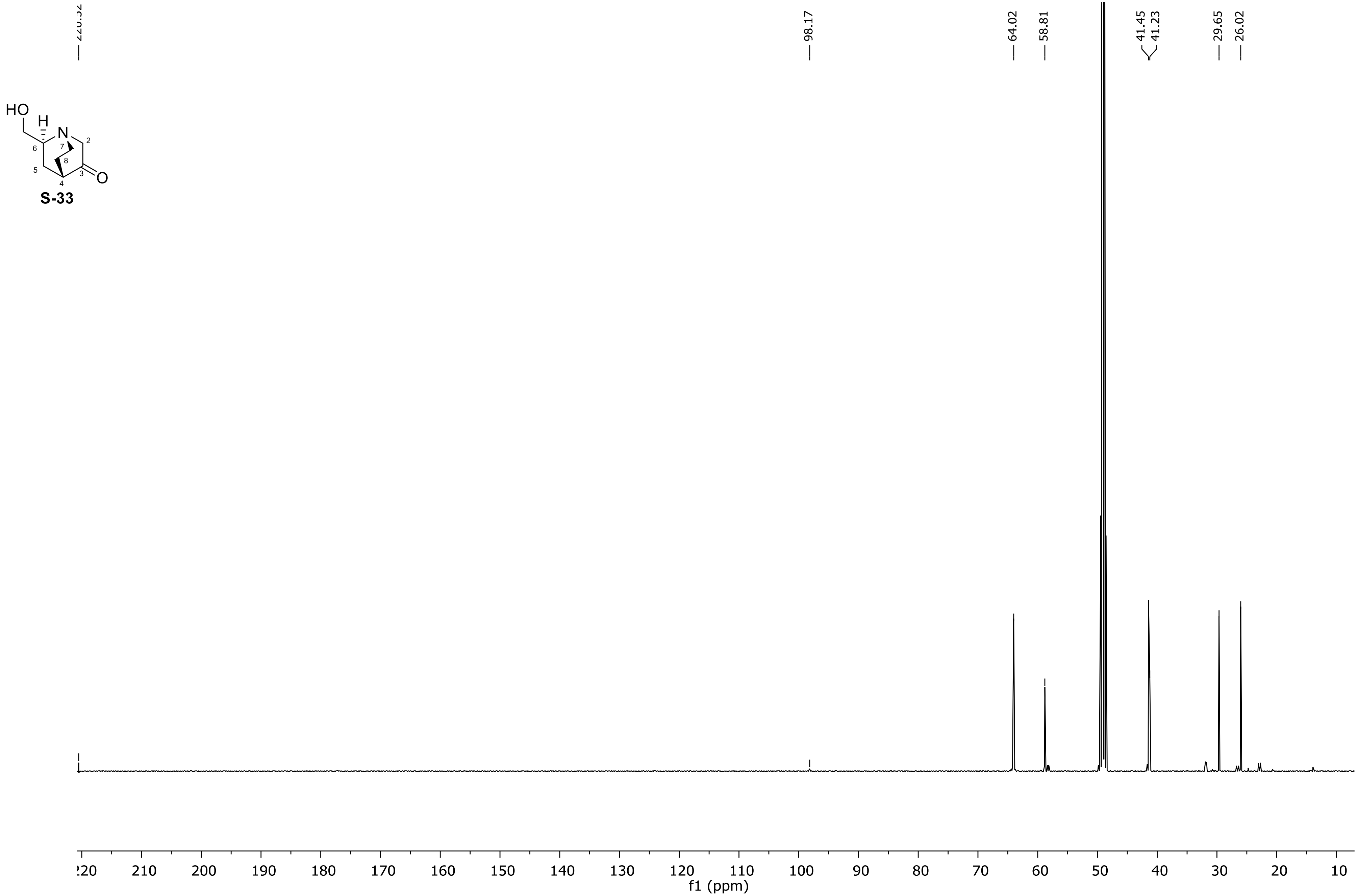
98.17
64.02
58.81
41.45
41.23
29.65
26.02
HO
H
N
2
3
4
5
6
7
8
O
S-33
220 210 200 190 180 170 160 150 140 130 120 110 100 90 80 70 60 50 40 30 20 10
f1 (ppm)

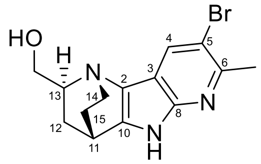
HO
H
N
Br
N
N
H

S-5

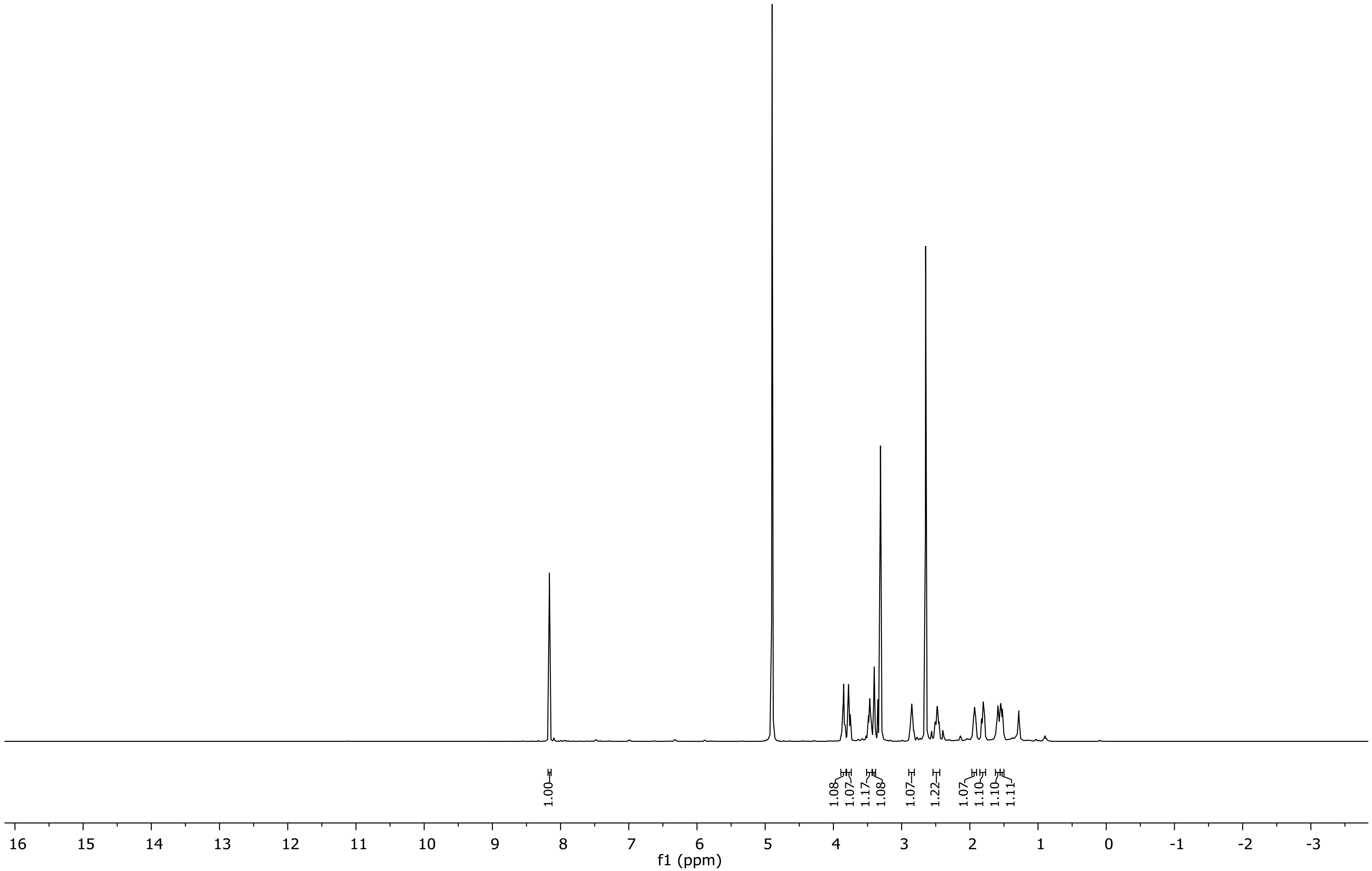
1.00
1.08
1.07
1.17
1.08
1.07
1.22
1.07
1.10
1.10
1.11
16 15 14 13 12 11 10 9 8 7 6 5 4 3 2 1 0 -1 -2 -3
f1 (ppm)

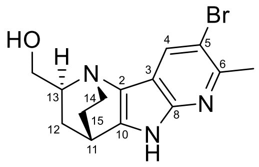
HO
H
N
Br
N
N
H
2
3
4
5
6
8
10
11
12
13
14
15

S-5

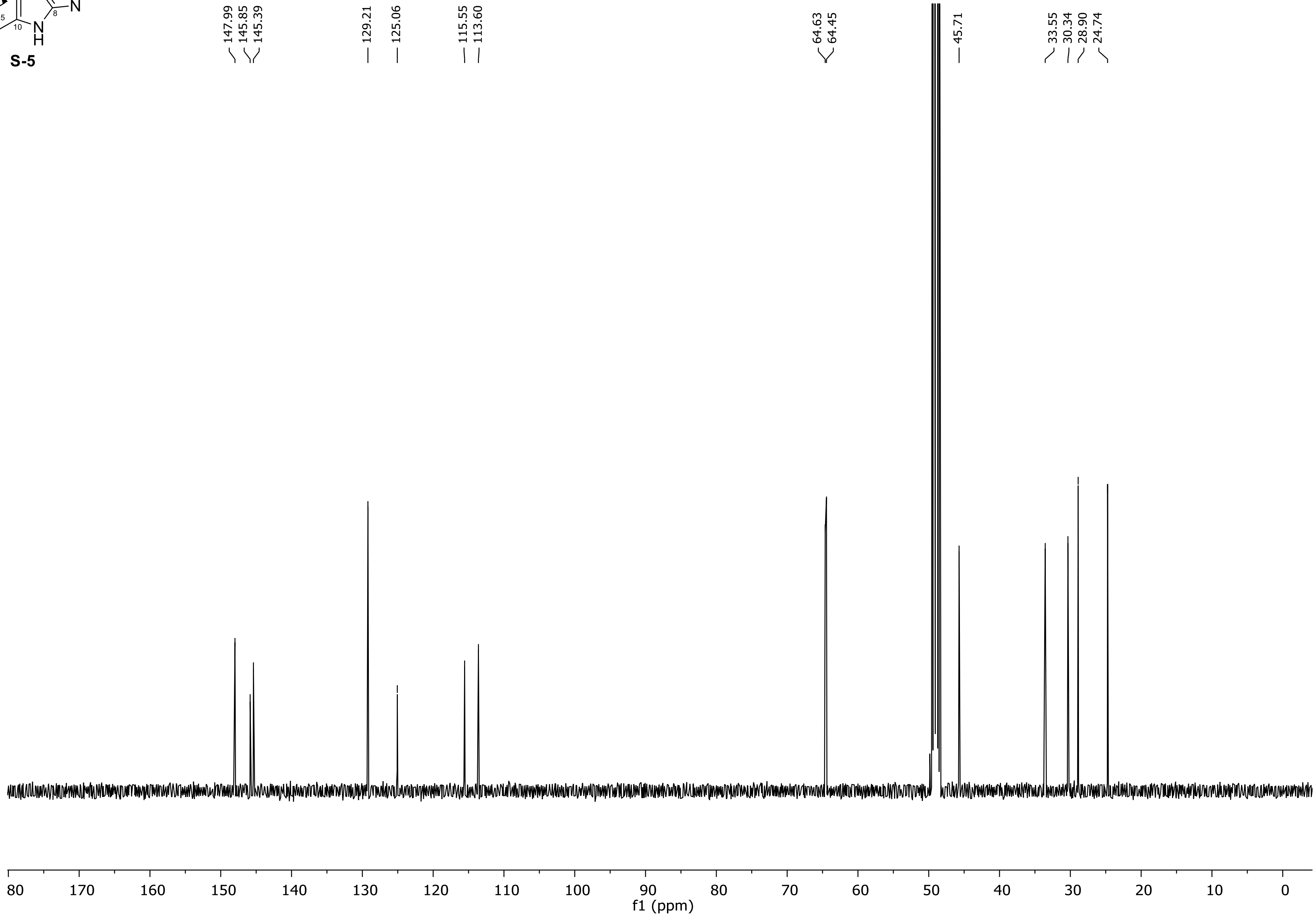
147.99
145.85
145.39
129.21
125.06
115.55
113.60
64.63
64.45
45.71
33.55
30.34
28.90
24.74
80
170
160
150
140
130
120
110
100
90
80
70
60
50
40
30
20
10
0
f1 (ppm)

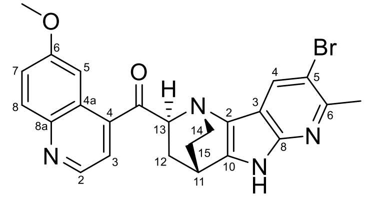
Br
O
N
H

S-34

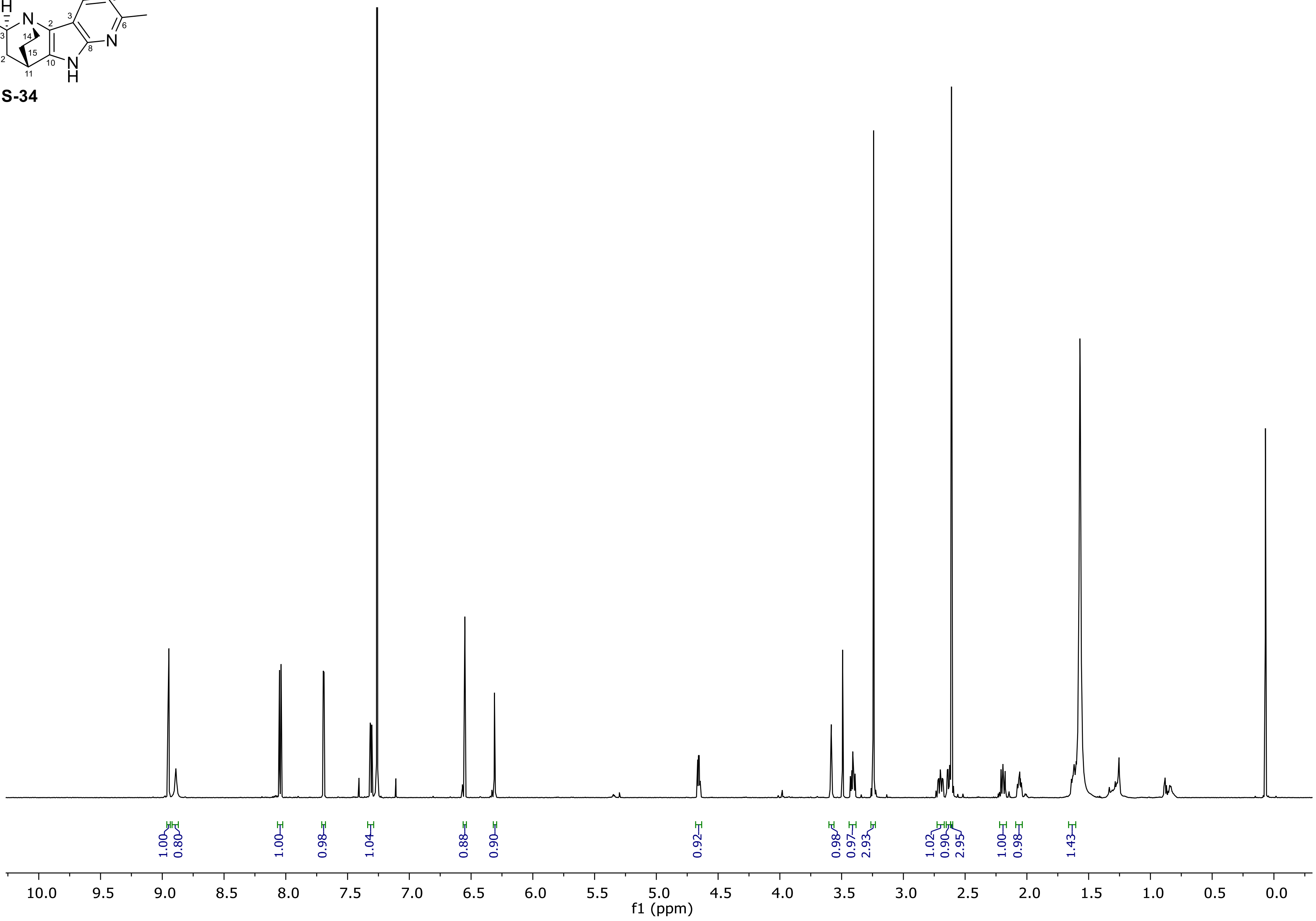
1.00
0.80
1.00
0.98
1.04
0.88
0.90
0.92
0.98
0.97
2.93
1.02
0.90
2.95
1.00
0.98
1.43
10.0 9.5 9.0 8.5 8.0 7.5 7.0 6.5 6.0 5.5 5.0 4.5 4.0 3.5 3.0 2.5 2.0 1.5 1.0 0.5 0.0
f1 (ppm)

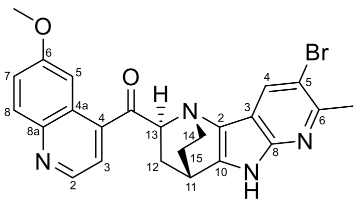
O
Br
O
H
N
N
N
H
5
6
7
8
8a
4a
4
3
2
13
12
14
15
11
10
8
2
3
4
5
6

S-34

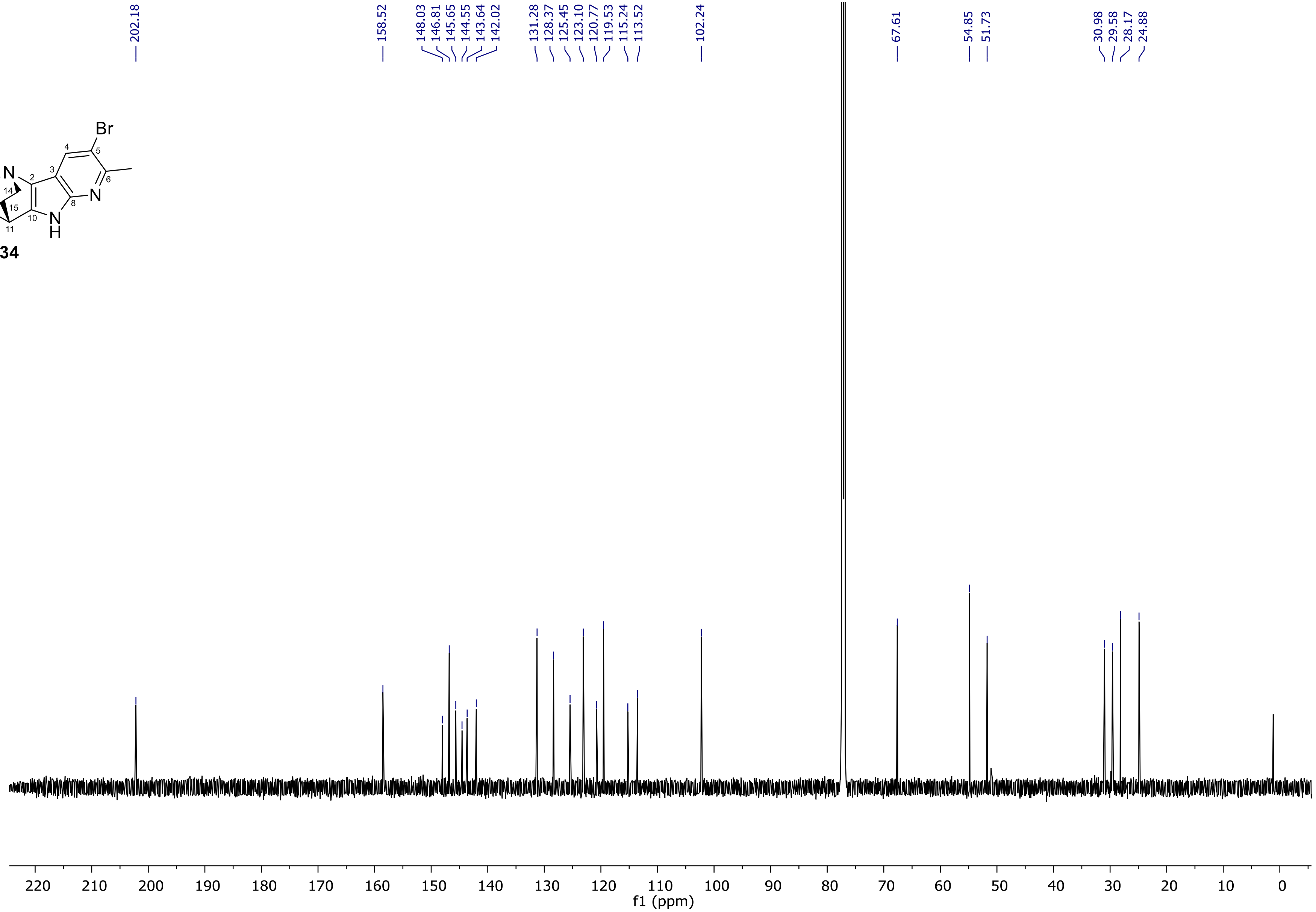
202.18
158.52
148.03
146.81
145.65
144.55
143.64
142.02
131.28
128.37
125.45
123.10
120.77
119.53
115.24
113.52
102.24
67.61
54.85
51.73
30.98
29.58
28.17
24.88
220 210 200 190 180 170 160 150 140 130 120 110 100 90 80 70 60 50 40 30 20 10 0
f1 (ppm)

8.0 References

[1] L. Kremer, E. Hennes, A. Brause, A. Ursu, L. Robke, H. T. Matsubayashi, Y. Nihongaki, J. Flegel, I. Mejdrová, J. Eickhoff, et al., *Angew. Chem. Int. Ed.* **2019**, *58*, 16617–16628.

[2] L. Robke, Y. Futamura, G. Konstantinidis, J. Wilke, H. Aono, Z. Mahmoud, N. Watanabe, Y.-W. Wu, H. Osada, L. Laraia, et al., *Chem. Sci.* **2018**, *9*, 3014–3022.

[3] A. S. Rosenthal, C. Tanega, M. Shen, B. T. Mott, J. M. Bougie, D.-T. Nguyen, T. Misteli, D. S. Auld, D. J. Maloney, C. J. Thomas, *An Inhibitor of the Cdc2-like Kinase 4 (Clk4)*, National Center For Biotechnology Information (US), **2010**. https://www.ncbi.nlm.nih.gov/books/NBK56236/

[4] "AID 2710 - Kinase Inhibition Profile Study on Inhibitors of CDC-like Kinase 4 - PubChem," can be found under https://pubchem.ncbi.nlm.nih.gov/bioassay/2710.

[5] M. Muraki, B. Ohkawara, T. Hosoya, H. Onogi, J. Koizumi, T. Koizumi, K. Sumi, J. Yomoda, M. V. Murray, H. Kimura, et al., *J. Biol. Chem.* **2004**, *279*, 24246–24254.

[6] A. Foucourt, D. Hédou, C. Dubouilh-Benard, A. Girard, T. Taverne, A. S. Casagrande, L. Désiré, B. Leblond, T. Besson, *Molecules* **2014**, *19*, 15411–15439.

[7] K. J. French, Y. Zhuang, L. W. Maines, P. Gao, W. Wang, V. Beljanski, J. J. Upson, C. L. Green, S. N. Keller, C. D. Smith, *J. Pharmacol. Exp. Ther.* **2010**, *333*, 129–39.

[8] M. E. Schnute, M. D. McReynolds, T. Kasten, M. Yates, G. Jerome, J. W. Rains, T. Hall, J. Chrencik, M. Kraus, C. N. Cronin, et al., *Biochem. J.* **2012**, *444*, 79–88.

[9] C. Sadhu, B. Masinovsky, K. Dick, C. G. Sowell, D. E. Staunton, *J. Immunol.* **2003**, *170*, 2647–54.

[10] B. J. Lannutti, S. A. Meadows, S. E. M. Herman, A. Kashishian, B. Steiner, A. J. Johnson, J. C. Byrd, J. W. Tyner, M. M. Loriaux, M. Deininger, et al., *Blood* **2011**, *117*, 591–4.

[11] K. Down, A. Amour, I. R. Baldwin, A. W. J. Cooper, A. M. Deakin, L. M. Felton, S. B. Guntrip, C. Hardy, Z. A. Harrison, K. L. Jones, et al., *J. Med. Chem.* **2015**, *58*, 7381–7399.

[12] P. Ertl, S. Roggo, A. Schuffenhauer, *J. Chem. Inf. Model.* **2008**, *48*, 68–74.

[13] I. Colomer, C. J. Empson, P. Craven, Z. Owen, R. G. Doveston, I. Churcher, S. P. Marsden, A. Nelson, *Chem. Commun.* **2016**, *52*, 7209–7212.

[14] M. A. Bray, S. Singh, H. Han, C. T. Davis, B. Borgeson, C. Hartland, M. Kost-Alimova, S. M. Gustafsdottir, C. C. Gibson, A. E. Carpenter, *Nat. Protoc.* **2016**, *11*, 1757–1774.

[15] M. H. Woehrmann, W. M. Bray, J. K. Durbin, S. C. Nisam, A. K. Michael, E. Glassey, J. M. Stuart, R. S. Lokey, *Mol. Biosyst.* **2013**, *9*, 2604–2617.

[16] A. Pahl, S. Sievers, in *Methods Mol. Biol.*, Humana Press, New York, NY, **2019**, pp. 115–126.

[17] https://github.com/mpimp-comas/cellpainting. Last visited 18/02/2020.

[18] L. Laraia, K. Ohsawa, G. Konstantinidis, L. Robke, Y. W. Wu, K. Kumar, H. Waldmann,

Angew. Chem. Int. Ed. **2017**, *56*, 2145–2150.

[19] H. Waldmann, L. Laraia, G. Garivet, D. J. Foley, N. Kaiser, S. Müller, S. Zinken, T. Pinkert, J. Wilke, D. Corkery, et al., *Angew. Chem. Int. Ed.* **2019**, DOI 10.1002/anie.201913712.

[20] L. Robke, L. Laraia, M. A. Carnero Corrales, G. Konstantinidis, M. Muroi, A. Richters, M. Winzker, T. Engbring, S. Tomassi, N. Watanabe, et al., *Angew. Chem. Int. Ed.* **2017**, *56*, 8153–8157.

[21] L. Robke, T. Rodrigues, P. Schröder, D. J. Foley, G. J. L. Bernardes, L. Laraia, H. Waldmann, *Tetrahedron* **2018**, *74*, DOI 10.1016/j.tet.2018.07.021.

[22] L. Laraia, A. Friese, D. P. Corkery, G. Konstantinidis, N. Erwin, W. Hofer, H. Karatas, L. Klewer, A. Brockmeyer, M. Metz, et al., *Nat. Chem. Biol.* **2019**, *15*, 710–720.

[23] F. De Chaumont, S. Dallongeville, N. Chenouard, N. Hervé, S. Pop, T. Provoost, V. Meas-Yedid, P. Pankajakshan, T. Lecomte, Y. Le Montagner, et al., *Nat. Methods* **2012**, *9*, 690–696.

[24] D. E. Portlock, D. Naskar, L. West, W. L. Seibel, T. Gu, H. J. Krauss, X. S. Peng, P. M. Dybas, E. G. Soyke, S. B. Ashton, et al., *Tetrahedron Lett.* **2003**, *44*, 5365–5368.

[25] F. I. Carroll, P. Abraham, K. Gaetano, S. W. Mascarella, R. A. Wohl, J. Lind, K. Petzoldt, *J. Chem. Soc. Perkin Trans. 1* **1991**, 3017–3026.

[26] C. Chen, D. R. Lieberman, R. D. Larsen, T. R. Verhoeven, P. J. Reider, *J. Org. Chem.* **1997**, *62*, 2676–2677.
